# Merging Hydrogen‐Atom‐Transfer and the Truce‐Smiles Rearrangement for Synthesis of β‐Arylethylamines from Unactivated Allylsulfonamides

**DOI:** 10.1002/anie.202418869

**Published:** 2025-04-21

**Authors:** Hanqi Zhou, Danijela Lunic, Nil Sanosa, Diego Sampedro, Ignacio Funes‐Ardoiz, Christopher J. Teskey

**Affiliations:** ^1^ Institute of Organic Chemistry RWTH Aachen University Landoltweg 1 52074 Aachen Germany; ^2^ Institute of Organic Chemistry TU Braunschweig Hagenring 30 38106 Braunschweig Germany; ^3^ Department of Chemistry Instituto de Investigación en Química de la Universidad de La Rioja (IQUR) Universidad de La Rioja Madre de Dios 53 26004 Logroño Spain

**Keywords:** Arylation, Cobalt, Density functional calculations, Hydrogen‐atom‐transfer, Reaction mechanisms

## Abstract

Arylethylamines are crucial elements in pharmaceutical molecules, making methods for their synthesis highly significant. The Truce–Smiles rearrangement is a well‐developed strategy to synthesize arylethylamine motifs via aryl migration. However, most examples require amide substrates to activate the alkene to attack by a radical precursor. This strategy both limits the product scope to amide‐containing compounds as well as necessitating the incorporation of specific functional groups arising from the initial radical addition. In this work, we overcome these limitations, delivering a hydrogen‐atom transfer from a cobalt catalyst to unactivated alkenes to yield β‐arylethylamines with simple alkyl chains. DFT studies reveal that increasing the steric hindrance in at least one of the *ortho* positions on the migrating aromatic group promotes *ipso* over *ortho* addition, a selectivity that contrasts with previous methods.

## Graphical abstract

A mild, photoinduced radical hydroarylation of unactivated *N*‐allylamine derivatives is reported for the synthesis of β‐arylethylamines with simple alkyl chains. DFT studies reveal that *ipso* attack is more favourable than *ortho* attack when one of the *ortho* positions at the aromatic group attached to sulfur was blocked.

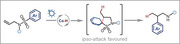

The β‐arylethylamine motif is prevalent in pharmaceutical molecules due to its distinctive biological activity and synthetic versatility (Scheme 1a).^[^ ^1^, ^2^ ^]^ Conventional synthetic approaches include reductive amination,^[^ ^3^ ^]^ aminoarylation^[^ ^4^, ^5^, ^6^, ^7^, ^8^, ^9^, ^10^, ^11^ ^]^ and hydroamination.^[^ ^12^ ^]^ An alternative strategy is radical‐mediated hydroarylation which has emerged as a potentially efficient strategy for accessing a variety of β‐arylethylamines under mild conditions.^[^ ^13^ ^]^ Markovnikov‐selective radical hydroarylation may occur via catalytic hydrogen‐atom‐transfer from a high‐valent, first‐row transition metal (MHAT catalysis) either using a dual‐catalytic, cross‐coupling strategy^[^ ^14^, ^15^, ^16^ ^]^ or via radical cycloisomerisation onto the *ortho*‐position of a pendant arene, followed by subsequent rearomatisation (Scheme 1b).^[^ ^17^, ^18^, ^19^ ^]^ In the second case, (hetero‐)cyclic compounds are formed, precluding this as a strategy for linear arylethylamine synthesis.

**Scheme 1:**
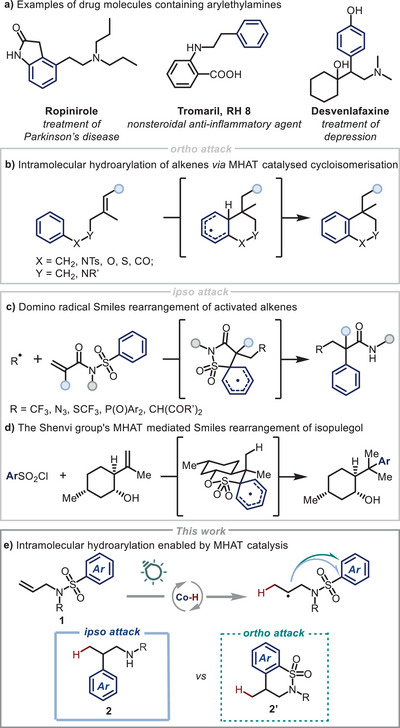
The importance of β‐arylethylamine motifs in drug molecules. Hydroarylation via MHAT and radical Truce‐Smiles reactions.

An alternative strategy that we proposed would involve the design of a suitable substrate class for MHAT catalysis whereby the inherent preference for *ortho*‐cyclisation is supplanted by *ipso*‐substitution. This type of radical Truce‐Smiles rearrangement,^[^ ^20^, ^21^ ^]^ has been extensively investigated in recent years,^[^ ^22^, ^23^ ^]^ though rarely in combination with transition metal catalysis.^[^ ^24^ ^]^ An early report from the Stephenson group described a photoredox‐catalysed Smiles rearrangement via the formation of a carbon‐centered radical, formed upon single electron reduction of difluoroalkylbromides.^[^ ^25^ ^]^ Further exploitation of this concept has allowed a variety of different moieties to be formed.^[^ ^26^, ^27^, ^28^, ^29^ ^]^ This includes two component domino reactions, for instance, developed by the Nevado group, where electrophilic radicals can react with *N*‐arylsulfonyl acrylamides, leading to the formation of corresponding arylethylamides (Scheme 1c).^[^ ^30^, ^31^, ^32^, ^33^ ^]^ However, most of these examples rely on olefins that are amide or styrene‐derived.^[^ ^6^ ^]^ In contrast, there are fewer examples with simple, unactivated *N*‐allylsulfonamides,^[^ ^34^, ^35^, ^36^ ^]^ which limits the generality of this approach to arylethylamines.^[^ ^10^, ^11^, ^33^ ^]^

Taking inspiration from the approaches described above–and work from the Shenvi group on a hydroarylative Truce‐Smiles rearrangement employing a stoichiometric manganese reagent for HAT (Scheme 1d)^[^ ^37^ ^]^—we sought to develop a catalytic MHAT platform for hydroarylation. We rationalized that the use of the reductive MHAT catalytic system,^[^ ^38^ ^]^ that our group^[^ ^39^, ^40^, ^41^, ^42^ ^]^ and others^[^ ^43^, ^44^, ^45^, ^46^, ^47^, ^48^, ^49^, ^50^, ^51^ ^]^ have developed in the last years, may enable the formation of β‐arylethylamines via the following sequence: polarity‐matched HAT from a Co^III^–H species (formed by reduction and protonation of a Co^II^ catalyst); followed by radical aryl migration and reduction and protonation of the resulting *N*‐centered radical to form the product. However, we were aware that obtaining the desired products from *ipso* substitution could prove challenging due to the competitive, previously reported *ortho*‐cyclization pathway (Scheme 1e). Herein, we report our studies in the development of a photoinduced hydroarylation strategy of *N*‐arylsulfonyl allylanilines, demonstrating how substrate design can yield desired products with highly sterically substituted arene rings.

To evaluate the feasibility of the proposal, we began exploring the reactivity of *N*‐phenylsulfonyl allylanilines **1** as a model substrate class (Scheme 2a). However, only a moderate yield could be obtained with substrate **1a** after extensive screening of conditions, due to the generation of undesired six‐ and five‐membered heterocyclic products **2a**′ and **2a**″. As shown in the mechanistic proposal, once the HAT from Co^III^–H species occurs, the generated radical can evolve towards three different products via *ipso* addition (**2a**) or *ortho* addition to the phenyl group attached to sulfur (**2a**′) and nitrogen (**2a**″), respectively.^[^ ^52^ ^]^ To suppress this undesired, previously reported radical *ortho* attack, we prepared two additional substrates with di‐*ortho* and *ortho* substitution (**1b** and **1c**,). In both cases, the reaction selectivity shifts towards the generation of products **2b** & **2c**, respectively (Scheme 2a).

**Scheme 2:**
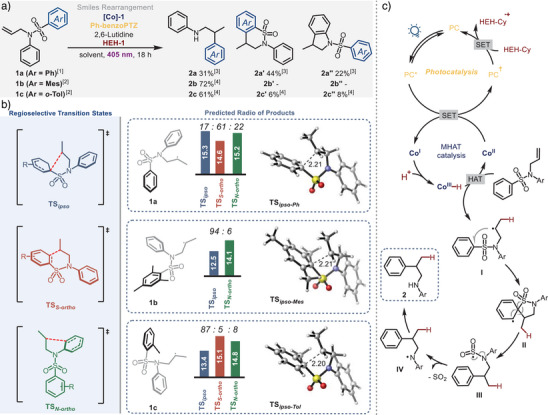
Reaction selectivity a), computational studies b), and proposed mechanism c).^[^ ^1^ ^]^ Conditions: [Co]‐1 (3.0 mol%), Ph‐benzoPTZ (1.0 mol%), HEH‐Cy (1.5 equiv), 2,6‐lutidine (1.0 equiv), benzene:HFIP (0.03 m, 35:1), 405 nm, 18 h;^[^ ^2^ ^]^ [Co]‐1 (2.0 mol%), Ph‐benzoPTZ (2.0 mol%), HEH‐Cy (3.0 equiv), 2,6‐lutidine (1.0 equiv), toluene:HFIP (0.05 m, 20:1), 405 nm, 18 h;^[^ ^3^ ^]^ Yield was determined by 1H‐NMR analysis with 1,3,5‐trimethoxybenzene as an internal standard;^[^ ^4^ ^]^ Isolated yield.

Based on these results, we selected to evaluate the influence of *N*‐arylsulfonyl allylaniline with various substituents at the *ortho* position of the migrating aryl group by a density‐functional theory (DFT) computational study at the SMD(C_6_H_6_) ωB97XD/Def2TZVPP//ωB97XD/Def2SVP level of theory (see further computational details in the Supporting Information), including quasi‐harmonic corrections with the Goodvibes program.^[^ ^53^ ^]^ For all three substrates, **1a**, **1b,** and **1c**, we observed that the formation of the first transition state was the rate‐determining step for all possible reaction pathways (Scheme 2b, see the complete free energy profiles in the Supporting Information). For substrate **1a**, the least stable and highest energy transition state occurs for *ipso* addition at the arene attached to sulfur (15.3 kcal mol^−1^), while the most stable transition state arises from *ortho* addition at the same aromatic group (14.6 kcal mol^−1^). The transition state energy for attack at the aromatic ring bonded directly to N has an energy of 15.2 kcal mol^−1^. By analyzing the energies of these three transition states, the predicted ratio of the products is calculated to be: **2a**:**2a**′:**2a**″ = 17:61:22 (Scheme 2b).

However, when both *ortho* positions are substituted (substrate **1b**), the *ortho* addition at the mesityl group is suppressed due to the lack of protons for rearomatisation. In contrast, the transition state formed by *ipso* addition to sulfur (12.5 kcal mol^−1^) was more stable than the *ortho* addition to the aromatic group attached to nitrogen (14.1 kcal mol^−1^). No *ortho*‐addition product was isolated experimentally which aligns well with this computational prediction (Scheme 2b). To further explore the influence of *ortho* substituents, a substrate with only one *ortho* position occupied (**1c**) was also investigated. Surprisingly, the transition state of *ipso* addition is still the most stable (13.4 kcal mol^−1^) compared to the two *ortho* additions (14.8, *N*‐aryl and 15.1 R_2_NSO_2_‐aryl, in kcal mol^−1^), corresponding again to the experimental results where aryl‐migration is the predominant product (Scheme 2b). Our analysis suggests that an increase in steric hindrance at the *ortho* position destabilizes the substrate by twisting the aromatic ring. This steric strain thus increases the ground state energy bringing it closer to the transition state energy for *ipso* attack where the *ipso* carbon becomes sp^3^ hybridized. Radical attack at the *ortho* positions is therefore not favorable when at least one *ortho* substituent on the aromatic group is present.

Based on our investigations and our previous work, we proposed the following mechanism (Scheme 2c). Initially, the photocatalyst, Ph‐benzoPTZ, is excited with 405 nm light. The excited state PC^*^ (E_ox_^*^ = −1.97 V vs SCE) can undergo single electron transfer (SET) with the commercially available cobalt(II) salen (E_red_ = −1.60 V vs SCE) to form the corresponding radical cation PC^•+^ and Co^I^ species.^[^ ^47^ ^]^ It was verified by the Stern–Volmer quenching experiment that PC* can be quenched by Co^II^ (see Supporting Information). The PC^•+^ can then be reduced by the cyclohexyl‐substituted Hantzsch ester (HEH‐Cy) to restore the ground state of the photocatalyst, completing the photocatalysis cycle and yielding radical cation, HEH‐Cy^•+^ (which fragments to form a cyclohexyl radical that is a spectator in most reactions–see Scheme 4). Meanwhile, the key Co^III^–H species is generated through the protonation of Co^I^. Following hydrogen atom transfer (HAT) from Co^III^–H to starting material **1**, an alkyl radical intermediate **I** is formed together with Co^II^, completing the MHAT catalysis cycle. Then, in the selectivity determining step, the alkyl radical reacts at the *ipso* position of the sulfonyl aromatic ring, giving aryl radical **II**. Rapid radical C─S bond breaking (**III**), followed by *N*‐desulfonylation, generates a nitrogen‐centred radical **IV,** which undergoes reduction and protonation to yield the final product. Without the addition of 2,6‐lutidine and HFIP, yields were diminished and only starting material was recovered in the absence of the photocatalyst, cobalt catalyst, Hantzsch ester, or light (see Supporting Information for more details).

Next, we probed the possible variation in the migrating aromatic group (Scheme 3a), starting with monosubstituted sulfones bearing electron‐withdrawing and electron‐donating groups at the *ortho*‐position. A range of functional groups including halogens, trifluoromethyl, and ester groups were all well tolerated to deliver the corresponding β‐arylethylamines in moderate to good yields (**2c**‐**2h**, **2k**, **2l**). Substrates where the migrating aryl group is di‐*ortho* substituted also performed well, giving the products good yields (**2b**, **2i**, **2j**, **2m**, **2n**). Notably, substrates with large steric groups, such as **1m** and **1n**, were also suitable for this reaction providing access to sterically congested products straightforwardly. Substrates containing a fused ring and heterocyclic ring were compatible with the reaction system to form **2r** and **2s** in 42% and 17% respectively. The low yield of **2s** was due to incomplete consumption of the starting material. Substrates without *ortho* position substituents were also evaluated (**2a**, **2o**‐**2q**), demonstrating that a *para* electron‐withdrawing group could improve the reaction efficiency to some extent.

**Scheme 3:**
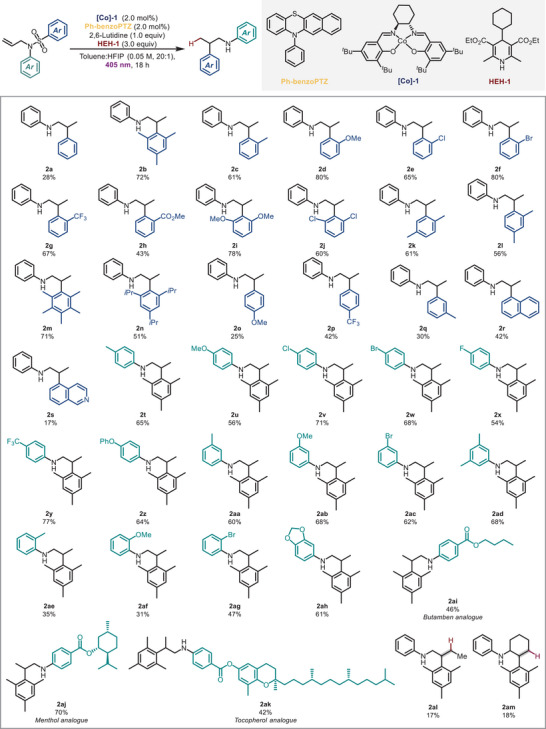
Substrate scope. Reactions performed on a 0.1 mmol scale, isolated yields.

Then we turned our attention to substrates with diverse aniline moieties. Substrates bearing different groups at *meta*‐ and *para‐*sites on the aniline (**2t**‐**2ad**) furnished the targeted products in good yields. However, substrates with *ortho* substituents (**2ae**‐**2ag**) showed decreased yields due to unreacted starting material. Benzo[d][1,3]dioxol‐5‐amine could also undergo the sequential process to give **2ah** in 61% yield. The reaction also proceeded smoothly with more complex substrates bearing drug moieties to deliver analogues of Butamben (**2ai**), Menthol (**2aj**), and Tocopherol (**2ak**). Finally, we investigated the reaction with internal alkene‐containing substrates. In these cases, the starting material was not completely consumed, likely due to the slower rate of HAT from the metal catalyst.^[^ ^54^ ^]^ However, low product yields of **2al** and **2am** were nevertheless isolated.

Next, we investigated the electronics of the alkene. MHAT from weak field ligand catalysts occurs more slowly with electron‐deficient alkenes.^[^ ^55^ ^]^ Therefore, it was interesting to observe that with the amide starting material, **1an**, product **2an**′ was isolated (Scheme 4a). This likely arises from the addition of the nucleophilic cyclohexyl radical (from fragmentation of HEH‐Cy^•+^) which is polarity matched with the electron‐poor olefin, in contrast to the electrophilic Co^III^–H. The same reactions without cobalt catalyst and without both cobalt catalyst and photocatalyst gave the same outcome but in slightly lower yield. However, by switching to the parent Hantzsch Ester and carrying out the reaction at a slightly higher temperature, we were able to isolate product **2an** from sequential MHAT and Smiles‐rearrangement (Scheme 4b). Finally, we demonstrated that the product from our methodology can be further arylated under photoredox conditions to yield product **3**.^[^ ^56^ ^]^

**Scheme 4:**
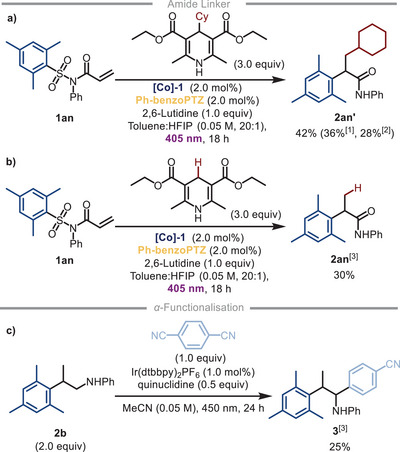
Truce‐Smiles rearrangement with amide substrate and α‐functionalisation of amines.^[^ ^1^ ^]^ Without [Co]‐1;^[^ ^2^ ^]^ without [Co]‐1 and Ph‐benzoPTZ;^[^ ^3^ ^]^ without a cooling fan.

In conclusion, we have developed a novel synthesis of β‐arylethylamines that uses MHAT catalysis to trigger aryl migration of *N*‐arylsulfonyl allylanilines. This hydroarylation protocol uses completely unactivated substrates to yield a series of valuable β‐arylethylamines, including the construction of products with high steric‐hindrance at the *ortho*‐positions of the aromatic ring. Indeed, computational analysis demonstrates the requirement of *ortho*‐aryl substitution to favor *ipso* radical addition and overcome the competitive *ortho*‐addition.

## Conflict of Interests

The authors declare no conflict of interest.

## Supporting information



Supporting Information

## Data Availability

The data that support the findings of this study are available in the supplementary material of this article.

## References

[1] A. Zhang , J. L. Neumeyer , R. J. Baldessarini , Chem. Rev. 2007, 107, 274–302. 17212477 10.1021/cr050263h

[2] R. R. Gainetdinov , M. C. Hoener , M. D. Berry , Pharmacol. Rev. 2018, 70, 549–620. 29941461 10.1124/pr.117.015305

[3] O. I. Afanasyev , E. Kuchuk , D. L. Usanov , D. Chusov , Chem. Rev. 2019, 119, 11857–11911. 31633341 10.1021/acs.chemrev.9b00383

[4] P. T. G. Rabet , S. Boyd , M. F. Greaney , Angew. Chem. Int. Ed. 2017, 56, 4183–4186. 10.1002/anie.201612445 28294505

[5] D. Wang , L. Wu , F. Wang , X. Wan , P. Chen , Z. Lin , G. Liu , J. Am. Chem. Soc. 2017, 139, 6811–6814. 28471665 10.1021/jacs.7b02455

[6] T. M. Monos , R. C. McAtee , C. R. J. Stephenson , Science 2018, 361, 1369–1373. 30262501 10.1126/science.aat2117 PMC9320120

[7] H. Jiang , X. Yu , C. G. Daniliuc , A. Studer , Angew. Chem. Int. Ed. 2021, 60, 14399–14404. 10.1002/anie.202101775 PMC8252614 33871137

[8] A. Bunescu , Y. Abdelhamid , M. J. Gaunt , Nature 2021, 598, 597–603. 34517408 10.1038/s41586-021-03980-8

[9] A. R. Allen , J.‐F. Poon , R. C. McAtee , N. B. Watson , D. A. Pratt , C. R. J. Stephenson , ACS Catal. 2022, 12, 8511–8526. 36312445 10.1021/acscatal.2c02577 PMC9608985

[10] E. A. Noten , C. H. Ng , R. M. Wolesensky , C. R. J. Stephenson , Nat. Chem. 2024, 16, 599–606. 38228850 10.1038/s41557-023-01404-w

[11] C. Hervieu , M. S. Kirillova , Y. Hu , S. Cuesta‐Galisteo , E. Merino , C. Nevado , Nat. Chem. 2024, 16, 607–614. 38228849 10.1038/s41557-023-01414-8 PMC10997517

[12] S. Ma , J. F. Hartwig , Acc. Chem. Res. 2023, 56, 1565–1577. 37272995 10.1021/acs.accounts.3c00141 PMC11620761

[13] A. J. Boyington , M.‐L. Y. Riu , N. T. Jui , J. Am. Chem. Soc. 2017, 139, 6582–6585. 28472584 10.1021/jacs.7b03262

[14] S. A. Green , J. L. M. Matos , A. Yagi , R. A. Shenvi , J. Am. Chem. Soc. 2016, 138, 12779–12782. 27623023 10.1021/jacs.6b08507

[15] S. L. Shevick , C. Obradors , R. A. Shenvi , J. Am. Chem. Soc. 2018, 140, 12056–12068. 30153002 10.1021/jacs.8b06458 PMC6329606

[16] S. A. Green , S. Vásquez‐Céspedes , R. A. Shenvi , J. Am. Chem. Soc. 2018, 140, 11317–11324. 30048124 10.1021/jacs.8b05868 PMC6245942

[17] H. Shigehisa , T. Ano , H. Honma , K. Ebisawa , K. Hiroya , Org. Lett. 2016, 18, 3622–3625. 27415770 10.1021/acs.orglett.6b01662

[18] J. L. M. Matos , S. A. Green , Y. Chun , V. Q. Dang , R. G. Dushin , P. Richardson , J. S. Chen , D. W. Piotrowski , B. M. Paegel , R. A. Shenvi , Angew. Chem. Int. Ed. 2020, 59, 12998–13003. 10.1002/anie.202003948 PMC7500246 32285542

[19] Y. Yamaguchi , Y. Seino , A. Suzuki , Y. Kamei , T. Yoshino , M. Kojima , S. Matsunaga , Org. Lett. 2022, 24, 2441–2445. 35312335 10.1021/acs.orglett.2c00700

[20] J. J. Köhler , W. N. Speckamp , Tetrahedron Lett. 1977, 18, 631–634.

[21] W. B. Motherwell , A. M. K. Pennell , J. Chem. Soc. Chem. Commun. 1991, 13, 877–879.

[22] A. R. Allen , E. A. Noten , C. R. J. Stephenson , Chem. Rev. 2022, 122, 2695–2751. 34672526 10.1021/acs.chemrev.1c00388 PMC9272681

[23] D. M. Whalley , M. F. Greaney , Synthesis 2022, 54, 1908–1918.

[24] N. Gillaizeau‐Simonian , E. Barde , A. Guérinot , J. Cossy , Chem. Eur. J. 2021, 27, 4004–4008. 33296109 10.1002/chem.202005129

[25] J. J. Douglas , H. Albright , M. J. Sevrin , K. P. Cole , C. R. J. Stephenson , Angew. Chem. Int. Ed. 2015, 54, 14898–14902. 10.1002/anie.201507369 PMC4725294 26474077

[26] D. Alpers , K. P. Cole , C. R. J. Stephenson , Angew. Chem. Int. Ed. 2018, 57, 12167–12170. 10.1002/anie.201806659 30025192

[27] C. Faderl , S. Budde , G. Kachkovskyi , D. Rackl , O. Reiser , J. Org. Chem. 2018, 83, 12192–12206. 30153021 10.1021/acs.joc.8b01538

[28] D. M. Whalley , H. A. Duong , M. F. Greaney , Chem. Commun. 2020, 56, 11493–11496. 10.1039/d0cc05049k 32857086

[29] D. M. Whalley , J. Seayad , M. F. Greaney , Angew. Chem. Int. Ed. 2021, 60, 22219–22223. 10.1002/anie.202108240 34370898

[30] W. Kong , M. Casimiro , N. Fuentes , E. Merino , C. Nevado , Angew. Chem. Int. Ed. 2013, 52, 13086–13090. 10.1002/anie.201307377 24174281

[31] W. Kong , M. Casimiro , E. Merino , C. Nevado , J. Am. Chem. Soc. 2013, 135, 14480–14483. 24047140 10.1021/ja403954g

[32] W. Kong , E. Merino , C. Nevado , Angew. Chem. Int. Ed. 2014, 53, 5078–5082. 10.1002/anie.201311241 24692217

[33] C. Hervieu , M. S. Kirillova , T. Suárez , M. Müller , E. Merino , C. Nevado , Nat. Chem. 2021, 13, 327–334. 33833448 10.1038/s41557-021-00668-4

[34] X. Wang , J. Liu , Z. Yu , M. Guo , X. Tang , G. Wang , Org. Lett. 2018, 20, 6516–6519. 30284833 10.1021/acs.orglett.8b02840

[35] D. M. Whalley , H. A. Duong , M. F. Greaney , Chem. Eur. J. 2019, 25, 1927–1930. 30536854 10.1002/chem.201805712

[36] E. A. Noten , R. C. McAtee , C. R. J. Stephenson , Chem. Sci. 2022, 13, 6942–6949. 35774166 10.1039/d2sc01228f PMC9200115

[37] S. W. M. Crossley , R. M. Martinez , S. Guevara‐Zuluaga , R. A. Shenvi , Org. Lett. 2016, 18, 2620–2623. 27175746 10.1021/acs.orglett.6b01047 PMC5871529

[38] S. Jana , V. J. Mayerhofer , C. J. Teskey , Angew. Chem. Int. Ed. 2023, 62, e202304882. 10.1002/anie.202304882 37184388

[39] E. Bergamaschi , V. J. Mayerhofer , C. J. Teskey , ACS Catal. 2022, 2, 14806–14811.

[40] J. Qin , M. Barday , S. Jana , N. Sanosa , I. Funes‐Ardoiz , C. J. Teskey , Angew. Chem. Int. Ed. 2023, 62, e202310639. 10.1002/anie.202310639 37676106

[41] V. J. Mayerhofer , M. Lippolis , C. J. Teskey , Angew. Chem. Int. Ed. 2024, 63, e202314870. 10.1002/anie.202314870 37947372

[42] D. Lunic , N. Vystavkin , J. Qin , C. J. Teskey , Angew. Chem. Int. Ed. 2024, 63, e202409388. 10.1002/anie.202409388 38977417

[43] Y. Kamei , Y. Seino , Y. Yamaguchi , T. Yoshino , S. Maeda , M. Kojima , S. Matsunaga , Nat. Commun. 2021, 12, 966. 33574227 10.1038/s41467-020-20872-z PMC7878493

[44] V. van der Puyl , R. O. McCourt , R. A. Shenvi , Tetrahedron Lett. 2021, 72, 153047. 37841701 10.1016/j.tetlet.2021.153047 PMC10573012

[45] X. Wu , C. N. Gannett , J. Liu , R. Zeng , L. F. T. Novaes , H. Wang , H. D. Abruña , S. Lin , J. Am. Chem. Soc. 2022, 144, 17783–17791. 36137298 10.1021/jacs.2c08278

[46] J. Liu , J. Rong , D. P. Wood , Y. Wang , S. H. Liang , S. Lin , J. Am. Chem. Soc. 2024, 146, 4380–4392. 38300825 10.1021/jacs.3c10989 PMC11219133

[47] M. Nakagawa , Y. Matsuki , K. Nagao , H. Ohmiya , J. Am. Chem. Soc. 2022, 144, 7953–7959. 35476545 10.1021/jacs.2c00527

[48] S. Shibutani , K. Nagao , H. Ohmiya , J. Am. Chem. Soc. 2024, 146, 4375–4379. 38300804 10.1021/jacs.3c10133

[49] H. Lindner , W. M. Amberg , T. Martini , D. M. Fischer , E. Moore , E. M. Carreira , Angew. Chem. Int. Ed. 2024, 63, e202319515. 10.1002/anie.202319515 38415968

[50] H. Yan , Q. Liao , Y. Chen , G. G. Gurzadyan , B. Lu , C. Wu , L. Shi , Angew. Chem. Int. Ed. 2023, 62, e202302483. 10.1002/anie.202302483 37042236

[51] S. Gnaim , A. Bauer , H.‐J. Zhang , L. Chen , C. Gannett , C. A. Malapit , D. E. Hill , D. Vogt , T. Tang , R. A. Daley , W. Hao , R. Zeng , M. Quertenmont , W. D. Beck , E. Kandahari , J. C. Vantourout , P.‐G. Echeverria , H. D. Abruna , D. G. Blackmond , S. D. Minteer , S. E. Reisman , M. S. Sigman , P. S. Baran , Nature 2022, 605, 687–695. 35614246 10.1038/s41586-022-04595-3 PMC9206406

[52] M. Tada , H. Shijima , M. Nakamura , Org. Biomol. Chem. 2003, 1, 2499–2505. 12956067 10.1039/b303728b

[53] G. Luchini , J. V. Alegre‐Requena , I. Funes‐Ardoiz , R. S. Paton , F1000Research 2020, 9, 291.

[54] Y.‐N. Yin , B.‐S. Zhao , H.‐Y. Liu , R.‐Q. Sheng , D.‐C. Ouyang , R. Zhu , Chem 2024, 10, 14806–14811.

[55] S. L. Shevick , C. V. Wilson , S. Kotesova , D. Kim , P. L. Holland , R. A. Shenvi , Chem. Sci. 2020, 11, 12401–12422. 33520153 10.1039/d0sc04112b PMC7810138

[56] H. Zhao , D. Leonori , Angew. Chem. Int. Ed. 2021, 60, 7669–7674. 10.1002/anie.202100051 PMC8048505 33459469

