# Supplementary material for: Merging Hydrogen‐Atom‐Transfer and the Truce‐Smiles Rearrangement for Synthesis of β‐Arylethylamines from Unactivated Allylsulfonamides
Source: Angew Chem Int Ed Engl. 2025 Apr 21;64(19):e202418869. doi: 10.1002/anie.202418869 (PMC12051773; doi:10.1002/anie.202418869)

Supporting Information

Merging Hydrogen-Atom-Transfer and the Truce-Smiles Rearrangement for Synthesis of β-Arylethylamines from Unactivated Allylsulfonamines

Hanqi Zhou,[a,b] Danijela Lunic,[a,c] Nil Sanosa,[c] Diego Sampedro,[c]

Ignacio Funes-Ardoiz*,[c] and Christopher J. Teskey*,[b]

[a] Institute of Organic Chemistry, RWTH Aachen University, Landoltweg 1, 52074 Aachen, Germany

[b] Institute of Organic Chemistry, Technische Universität Braunschweig, Hagenring 30, 38106 Braunschweig, Germany

[c] Department of Chemistry, Instituto de Investigación en Química de la Universidad de La Rioja (IQUR)Centro de Investigación en Síntesis Química (CISQ), Universidad de La Rioja, Madre de Dios 53, 26006 Logroño, Spain

*Email: Christopher.teskey@tu-braunschweig.de

Table of Contents

1. Materials and Methods 1
2. Selected Optimization Reactions 2
2.1 Sulfonamide (Phenyl-substituted) 2
2.1.1 Hantzsch ester screening 2
2.1.2 Solvent screening 2
2.1.3 Cobalt catalyst screening 3
2.1.4 Photocatalyst screening 4
2.1.5 Photocatalyst loading screening 5
2.1.6 Ratio of solvent screening 5
2.1.7 Hantzsch ester loading screening 5
2.1.8 Cobalt catalyst loading screening 6
2.1.9 Hydrogen source screening 6
2.1.10 Cosolvent screening 6
2.1.11 Additive screening 7
2.2 Sulfonamide (Mesityl-substituted) 8
2.2.1 Cobalt catalyst loading screening 8
2.2.2 Photocatalyst loading screening 8
2.2.3 Hantzsch ester loading screening 8
2.2.4 Base loading screening 9
2.2.5 Solvent screening 9
2.2.6 Ratio of solvent screening 9
2.2.7 Concentration screening 10
2.3 Control reactions 10
3. Synthesis of Starting Materials 11
3.1 General procedure of Sulfonamide 11
4. General procedure and characterization data of Smile rearrangement products 23
4.1 General procedure 1 of Smile rearrangement of Sulfonamide 23
4.2 General procedure 2 of Smile rearrangement of Sulfonamide 36
5. General procedure and characterization data of photoinduced α-functionalisation of 2b 37
6. Mechanistic experiments 38
6.1 UV-Vis experiments 38
6.2 Quenching experiments 39
6.3 Deuteration experiments 41
6.4 Trapping with TEMPO 43
7. Computational details 44
7.1 Free energy profiles for the reaction pathways of 1a 44
7.2. Benchmarking study of selectivity determining transition states of 1a 46
7.3 Energy profiles for the reaction pathways of 1b 47
7.4 Benchmarking study of selectivity determining transition states of 1b 48
7.5 Energy profiles for the reaction pathways of 1c 49
7.6 Benchmarking study of selectivity determining transition states of 1c 51

7.7 XYZ Coordinates and energies of the calculated species 52
8. References.. 93
9. NMR Spectra 95

1. Materials and Methods

Unless otherwise stated, all reactions were performed utilizing standard Schlenk techniques. All reagents and starting materials were purchased at reagent grade and used as received. Anhydrous solvents were dried using an Innovative Technology PS-MD-5 solvent purification system. Thin layer chromatography (TLC) was performed on Merck Kieselgel 60 F254 aluminum plates with unmodified silica and visualized either under UV light or stained with potassium permanganate or vanillin. Column chromatography was performed with Merck silica gel 60 (35 – 70 mesh).

All ^{1}H, ^{13}C and ^{19}F NMR spectra were recorded at ambient temperature on either Varian V-NMRS 600, Varian V-NMRS 400, Bruker AV-400, Bruker AV-600 or Varian Mercury 300 spectrometers. Chemical shifts (δ/ppm) were referenced to the residual solvent peak in ^{1}H (7.26 ppm for Chloroform-d) and ^{13}C spectra (77.16 ppm for Chloroform-d). Coupling constants (J) are given in Hz. Signals are described as br = broad, s = singlet, d = doublet, dd = doublet of doublets, t = triplet, q = quartet, p = quintet, h = sextet and m = multiplet.

High-resolution mass spectrometry (HRMS) was performed using a Thermo Scientific LTQ Orbitrap XL spectrometer. Infrared (IR) spectra were recorded on a Perkin Elmer Spektrum 100 FT-IR spectrometer Spectrum 100 spectrometer with an UATR Diamond/KRS-5 crystal with attenuated total reflectance (ATR) and signals reported as wavenumbers in reciprocal centimeters. Gas chromatography coupled with mass spectrometry (GC-MS) was performed on an Agilent Technologies 5975 series MSD mass spectrometer under electrospray ionization (EI) mode coupled with an Agilent Technologies 7820A gas chromatograph employing an Agilent 19091s-433 HP-5MS column (30 m x 0.250 µm x 0.250 µm). The fluorescence quenching experiments have been carried out on an Agilent Cary Eclipse. The UV-Vis measurements have been carried out on an Agilent Cary 60.

All the photochemical reactions were carried out in an EvoluChem™ PhotoRedOxBox Duo equipped with two EvoluChem 405PF LED lamps (λ_{max} = 405 nm, 18 W).

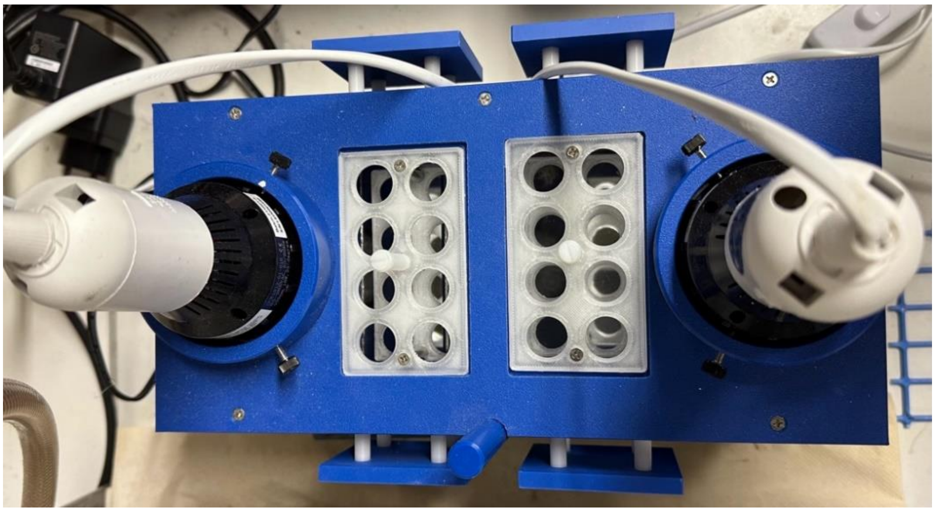

2. Selected Optimization Reactions

2.1 Sulfonamide (Phenyl-substituted)

Unless otherwise stated, the optimization has been carried out on a 0.1 mmol scale.

2.1.1 Hantzsch ester screening

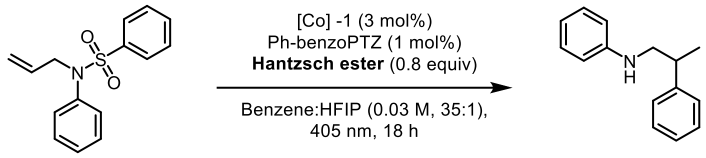

Entry	Hantzsch ester	Product (%)[a]	Starting material (%)[a]
1	HEH-1	22	42
2	HEH-2	11	53
3	HEH-3	0	11
4	HEH-4	0	100
5	HEH-5	11	63
6	HEH-6	9	73

[a]yield determined by ^{1}H-NMR analysis with 1,3,5-trimethoxybenzene as an internal standard.

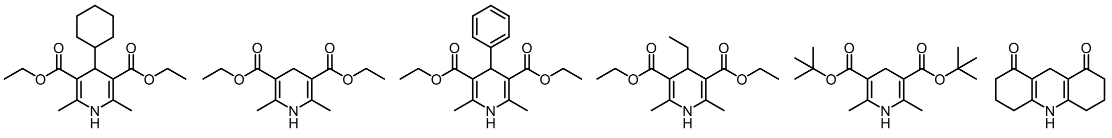

HEH-1 HEH-2 HEH-3 HEH-4 HEH-5 HEH-6

2.1.2 Solvent screening

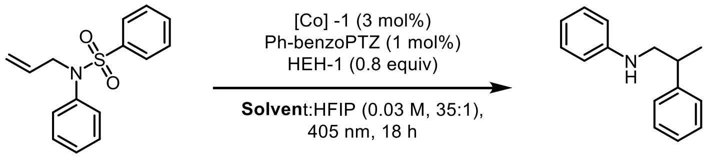

Entry	Solvent	Product (%)[a]	Starting material (%)[a]
1	MeCN	24	42
2	Toluene	24	31
3	DMSO	27	51
4	^{i}PrOH	15	38
5	THF	19	57
6	DMF	0	45
7	Acetone	18	24
8	DCM	3	77
9	1,4-dioxane	13	42
10	Et_2O	22	16
11	DME	20	27

12	EA	0	49
13	Benzene + 1-Octanol (1:1)	22	27
14	Benzene + Cyclohexanone (1:1)	17	40
15	Benzene + Dimethoxyethane (1:1)	18	38
16	HFIP	0	0
17	PhCl	16	45
18	PhOMe	14	41
19	Benzene	22	42
20	$PhCF_3$	0	87

[a]yield determined by ^{1}H-NMR analysis with 1,3,5-trimethoxybenzene as an internal standard.

2.1.3 Cobalt catalyst screening

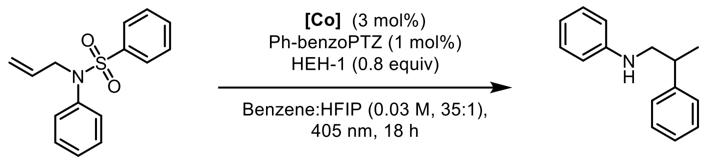

Entry	[Co] Cat.	Product (%)[a]	Starting material (%)[a]
1	[Co] -1	22	42
2	[Co] -2	17	46
3	[Co] -3	22	40
4	[Co] -4	15	53
5	[Co] -5	12	62
6	[Co] -6	0	99
7	[Co] -7	23	26
8	[Co] -8	23	31
9	[Co] -9	1	2
10	[Co] -10	17	58
11	[Co] -11	24	25
12	[Co] -12	0	99
13	[Co] -13	0	67
14	[Co] -14	0	88
15	[Co] -15	0	4
16	[Co] -16	0	80
17	$Co(acac)_2$	0	85

[a]yield determined by ^{1}H-NMR analysis with 1,3,5-trimethoxybenzene as an internal standard.

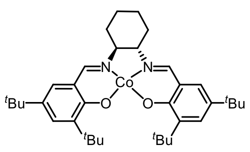

[Co] -1

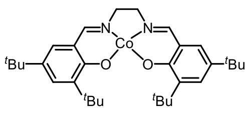

[Co] -2

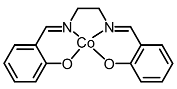

[Co] -3

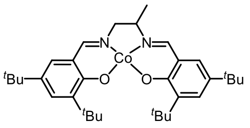

[Co] -4

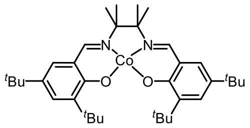

[Co] -5

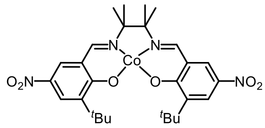

[Co] -6

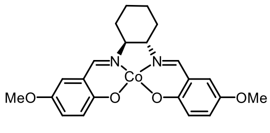

[Co] -7

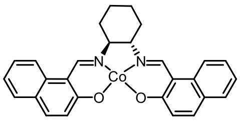

[Co] -8

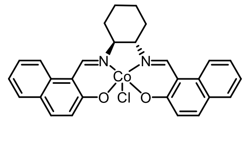

[Co] -9

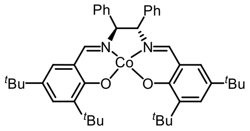

[Co] -10

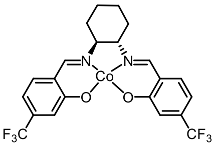

[Co] -11

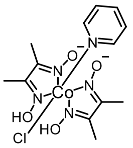

[Co] -12

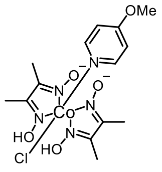

[Co] -13

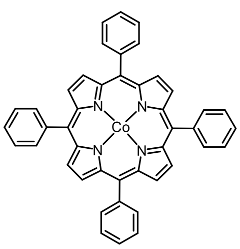

[Co] -14

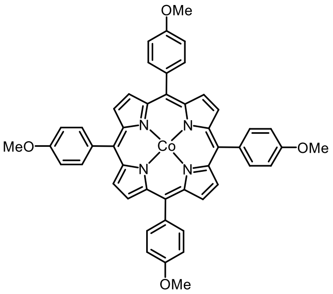

[Co] -15

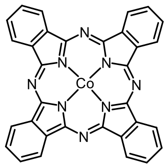

[Co] -16

2.1.4 Photocatalyst screening

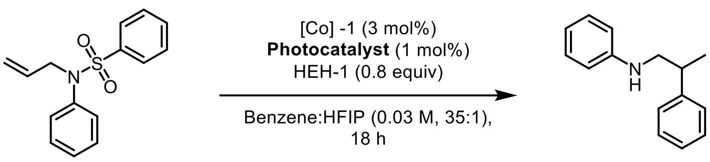

Entry	Photocatalyst	Product (%)[a]	Starting material (%)[a]
1	Ph-benzoPTZ	22	42
2	4CzIPN (405nm)	23	34
3	Mes-Acr-Ph-BF_4 (405 nm)	22	30
4	Ir(ppy)$_3$ (405 nm)	20	35
5	Ir(*p*F-ppy)$_3$ (405 nm)	21	35
6	4CzIPN (450 nm)	24	29
7	Mes-Ar (450 nm)	19	47
8	Ru(bmp)$_3$Cl$_2$ (450 nm)	5	86
9	Eosin Y (450 nm)	7	72
10	[Ir(dFCF$_3$ppy)$_2$(bpy)]PF$_6$ (450 nm)	11	64
11	Mes-(^{t}Bu)$_2$Acr-Ph-BF_4 (450 nm)	13	60
12	Ph-PTZ (405 nm)	0	100
13	PC 1 (450 nm)	26	27
14	PC 2 (450 nm)	28	19

[a]yield determined by ^{1}H-NMR analysis with 1,3,5-trimethoxybenzene as an internal standard.

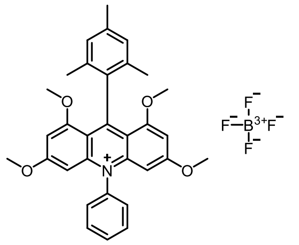

PC 1

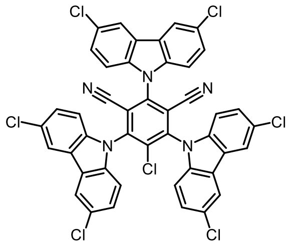

PC 2

2.1.5 Photocatalyst loading screening

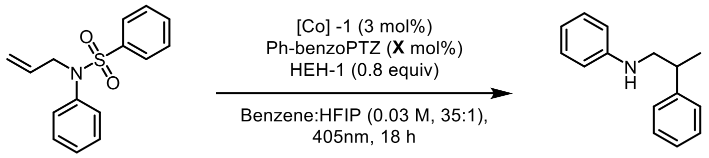

Entry	Ph-benzoPTZ (mol%)	Product (%)[a]	Starting material (%)[a]
1	1	22	42
2	2	21	34
3	5	21	31

[a]yield determined by ^{1}H-NMR analysis with 1,3,5-trimethoxybenzene as an internal standard.

2.1.6 Ratio of solvent screening

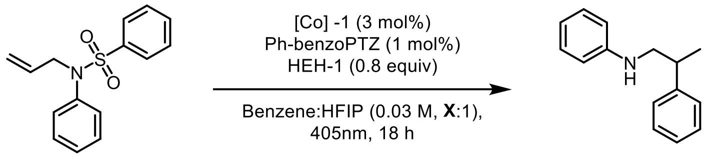

Entry	Benzene: HFIP	Product (%)[a]	Starting material (%)[a]
1	2:1	0	5
2	5:1	20	50
3	10:1	22	52
4	35:1	22	42
5	70:1	18	43

[a]yield determined by ^{1}H-NMR analysis with 1,3,5-trimethoxybenzene as an internal standard.

2.1.7 Hantzsch ester loading screening

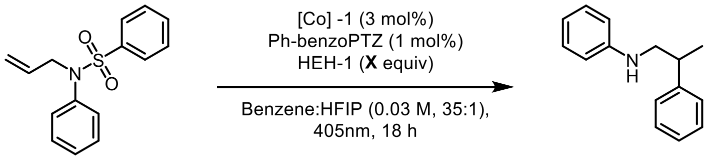

Entry	HEH-1 (equiv)	Product (%)[a]	Starting material (%)[a]
1	0.5	12	62
2	0.8	22	42
3	1.0	20	39
4	1.3	28	0

5	1.5	31	0
6	2.0	34	0
7	3.0	35	0
8	3.0 (HEH)	7	74

[a]yield determined by ^{1}H-NMR analysis with 1,3,5-trimethoxybenzene as an internal standard.

2.1.8 Cobalt catalyst loading screening

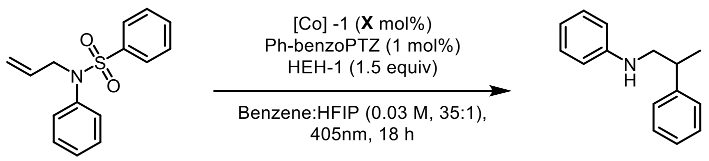

Entry	Co-Salen-1 (mol%)	Product (%)[a]	Starting material (%)[a]
1	1	19	36
2	2	29	9
4	3	31	0
3	5	25	18

[a]yield determined by ^{1}H-NMR analysis with 1,3,5-trimethoxybenzene as an internal standard.

2.1.9 Hydrogen source screening

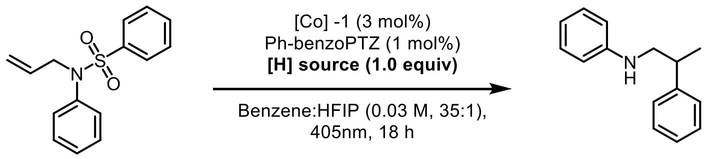

Entry	[H] Source	Product (%)[a]	Starting material (%)[a]
1	HEH-1	20	39
2	$PhSiH_3$	20	33
3	$AscH_2$	0	100
4	Et_3SiH	0	100
5	^{i}PrOH	0	100
6	PivOH	0	100

[a]yield determined by ^{1}H-NMR analysis with 1,3,5-trimethoxybenzene as an internal standard.

2.1.10 Cosolvent screening

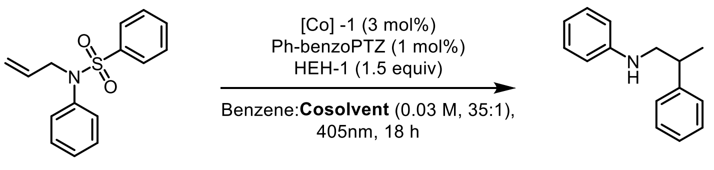

Entry	Cosolvent	Product (%)[a]	Starting material (%)[a]
1	^{i}PrOH	17	39
2	$PhCF_3$	13	52
3	Cosolvent 1	14	40
4	TFE	17	43

5	HFIP	31	0

[a]yield determined by ^{1}H-NMR analysis with 1,3,5-trimethoxybenzene as an internal standard.

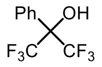

cosolvent 1

2.1.11 Additive screening

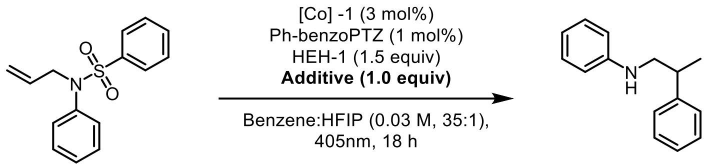

Entry	Additive	Product (%)[a]	Starting material (%)[a]
1	Pyridine	33	9
2	Cs_2CO_3	31	0
3	$Na_2S_2O_8$	29	26
4	TsOH•H_2O	0	88
5	2,4,6-collidine	37	0
6	2,4,6-collidine (0.015 M)	33	0
7	2,6-Dichloropyridine	21	31
8	2,6-Di-tert-butylpyridine	26	23
9	2,6-Lutidine	39(28)[b]	0
10	2,6-Lutidine (0.5 equiv)	34	0
11	2,6-Lutidine (2.0 equiv)	36	0
12	2,6-Lutidine (450 nm)	3	95
13	2,6-Lutidine (380 nm)	6	64
14	Quinoline	14	62
15	DMAP	35	0
16	2,2’-Bipyridine	0	96
17	2,6-Diacetylpyridine	6	80
18	Et_3N	31	0
19	5,6,7,8-Tetrahydroquinoline	32	0
20	2-Phenylpyridine	31	14
21	2-Benzylpyridine	27	21

[a]yield determined by ^{1}H-NMR analysis with 1,3,5-trimethoxybenzene as an internal standard. [b]isolated yield.

2.2 Sulfonamide (Mesityl-substituted)

2.2.1 Cobalt catalyst loading screening

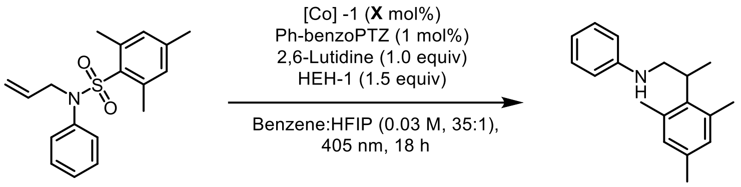

Entry	[Co] -1 (mol%)	Product (%)[a]	Starting material (%)[a]
1	1	59	33
2	2	63	27
3	4	47	42
4	6	29	59
5	8	17	73

[a]yield determined by ^{1}H-NMR analysis with 1,3,5-trimethoxybenzene as an internal standard.

2.2.2 Photocatalyst loading screening

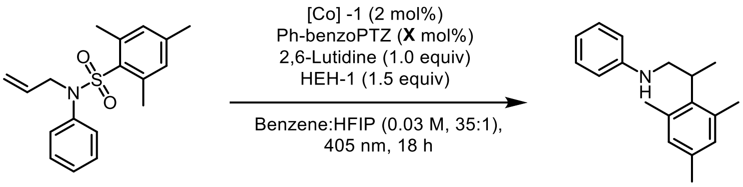

Entry	Ph-benzoPTZ (mol%)	Product (%)[a]	Starting material (%)[a]
1	1	63	27
2	2	65	34
3	3	63	34
4	4	57	28

[a]yield determined by ^{1}H-NMR analysis with 1,3,5-trimethoxybenzene as an internal standard.

2.2.3 Hantzsch ester loading screening

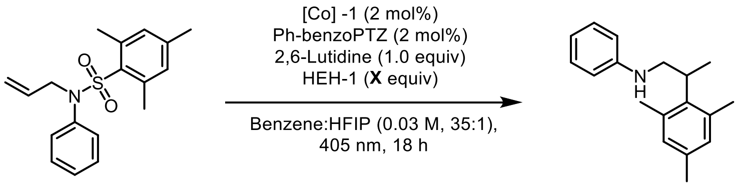

Entry	HEH-1 (equiv)	Product (%)[a]	Starting material (%)[a]
1	1	40	49
2	1.5	65	34
3	2	74	11
4	3	81	0

[a]yield determined by ^{1}H-NMR analysis with 1,3,5-trimethoxybenzene as an internal standard.

2.2.4 Base loading screening

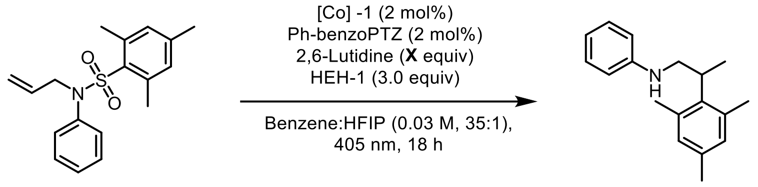

Entry	2,6-Lutidine (equiv)	Product (%)[a]	Starting material (%)[a]
1	0.5	64	12
2	1.0	81	0
3	1.5	80	0
4	2.0	79	0

[a]yield determined by ^{1}H-NMR analysis with 1,3,5-trimethoxybenzene as an internal standard.

2.2.5 Solvent screening

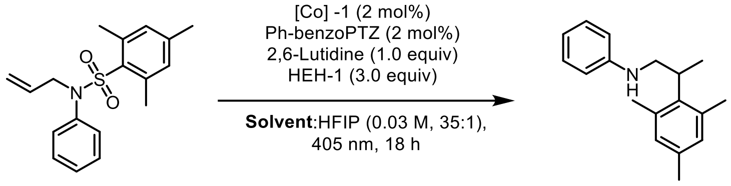

Entry	Solvent	Product (%)[a]	Starting material (%)[a]
1	Benzene	81	0
2	Toluene	81	0
3	CH_3CN	60	39
4	THF	69	7
5	PhCl	65	0

[a]yield determined by ^{1}H-NMR analysis with 1,3,5-trimethoxybenzene as an internal standard.

2.2.6 Ratio of solvent screening

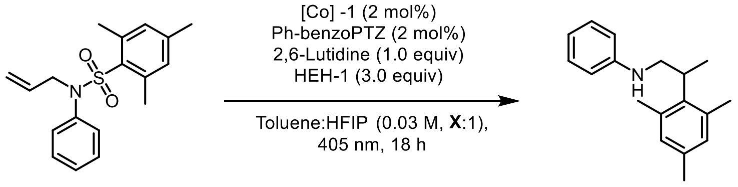

Entry	Toluene: HFIP	Product (%)[a]	Starting material (%)[a]
1	35:1	81	0
2	20:1	83	0
3	10:1	78	0

[a]yield determined by ^{1}H-NMR analysis with 1,3,5-trimethoxybenzene as an internal standard.

2.2.7 Concentration screening

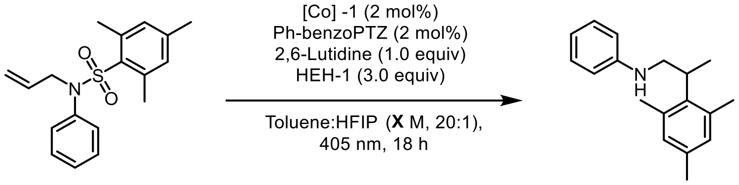

Entry	Concentration (M)	Product (%)[a]	Starting material (%)[a]
1	0.1	75	5
2	0.05	84(72[b])	0
3	0.03	83	0
4	0.02	83	0

[a]yield determined by ^{1}H-NMR analysis with 1,3,5-trimethoxybenzene as an internal standard. [b]isolated yield.

2.3 Control reactions

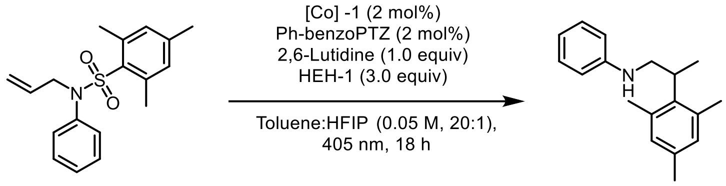

Entry	Variation	Product (%)[a]	Starting material (%)[a]
1	none	84(72) [b]	0
2	w/o Ph-benzoPTZ	0	67
3	w/o [Co]-1	0	63
4	w/o HEH-1	0	8
5	w/o 2,6-Lutidine	29	47
6	w/o HFIP	31	38
7	w/o 2,6-Lutidine and HFIP	20	63
8	w/o Light	0	71
9	Under air	41	18

[a]yield determined by ^{1}H-NMR analysis with 1,3,5-trimethoxybenzene as an internal standard. [b]isolated yield.

3. Synthesis of Starting Materials

3.1 General procedure of Sulfonamide

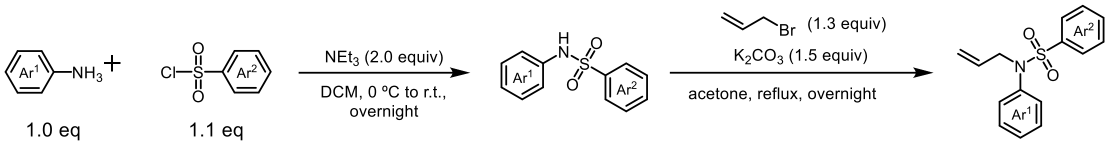

A solution of the respective aniline (1.0 equiv, 5.0 mmol) in DCM (10 mL) was cooled to 0 °C, triethylamine (2.0 equiv, 10.0 mmol) was then added dropwise, followed by respective aryl sulfonyl chloride (1.1 equiv, 5.5 mmol). The reaction mixture was warmed to room temperature and stirred overnight, then diluted with water (10.0 mL) and extracted with DCM (3 x 15 mL). The organic extracts were dried with sodium sulfate and concentrated under vacuum to obtain the crude aryl sulfamide.

The crude aryl sulfamide was dissolved in acetone (30 mL) and potassium carbonate (1.5 equiv, 7.5 mmol) was added, followed by allyl bromide (1.3 equiv, 6.5 mmol). The reaction mixture was heated to reflux at 60 °C and stirred overnight. After the reaction mixture cooling down, the mixture was filtered and concentrated under vacuum. The crude reaction was then purified using flash column chromatography. [1]

***N*-allyl-*N*-phenylbenzenesulfonamide (1a)**

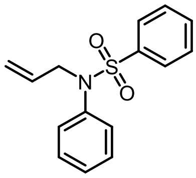

Prepared following general procedure of sulfonamide. **1a** was obtained as a white solid in 67% yield (0.92g, 3.35 mmol). **^{1}H NMR** (600 MHz, $CDCl_3$) δ 7.54 (dd, J = 8.4, 1.3 Hz, 1H), 7.52 – 7.48 (m, 1H), 7.43 – 7.34 (m, 1H), 7.23 – 7.18 (m, 2H), 6.96 (dd, J = 7.8, 1.9 Hz, 1H), 5.67 (ddt, J = 16.6, 10.1, 6.3 Hz, 1H), 5.05 – 4.93 (m, 1H), 4.12 (d, J = 6.3 Hz, 1H). **^{13}C NMR** (151 MHz, $CDCl_3$) δ 139.1, 138.6, 132.9, 132.8, 129.0, 129.0, 128.9, 128.0, 127.8, 119.0, 53.8. **IR (neat)**: v = 3060, 2323, 1589, 1488, 1448, 1337, 1284, 1218, 1158, 1076, 1021, 990, 925, 864, 768, 732, 687, 628, 556 cm^{-1}. **HRMS(ESI)**: calculated for $C_{15}H_{16}O_2NS$ $[M+H]^+$: 274.0824, found: 274.0898.

***N*-allyl-2,4,6-trimethyl-*N*-phenylbenzenesulfonamide (1b)**

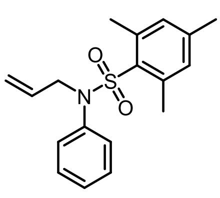

Prepared following general procedure of sulfonamide. **1b** was obtained as a white solid in 79% yield (1.24g, 3.95 mmol). **^{1}H NMR** (600 MHz, $CDCl_3$) δ 7.20 – 7.12 (m, 3H), 7.06 – 7.01 (m, 2H), 6.79 (s, 2H), 5.72 (ddt, J = 16.2, 10.8, 6.5 Hz, 1H), 5.06 – 4.88 (m, 2H), 4.23 (dt, J = 6.5, 1.3 Hz, 2H), 2.36 (s, 6H), 2.19 (s, 3H). **^{13}C NMR** (151 MHz, $CDCl_3$) δ 142.6, 140.5, 139.1, 133.3, 132.9, 131.9, 129.8, 129.1, 128.0, 119.0, 53.4, 23.0, 21.1. **IR (neat)**: v = 3854, 3063, 3007, 2671, 2330, 2166, 1994, 1905, 1775, 1591, 1490, 1447, 1338, 1289, 1219, 1158, 1086, 1067, 988, 927, 866, 766, 734, 689 cm^{-1}. **HRMS(ESI)**: calculated for $C_{18}H_{21}O_2NNaS$ $[M+Na]^+$: 338.1185, found: 338.1180.

N-allyl-2-methyl-N-phenylbenzenesulfonamide (1c)

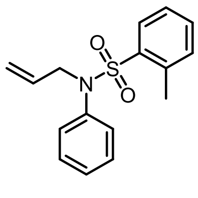

Prepared following general procedure of sulfonamide. **1c** was obtained as a yellow oil in 53% yield (0.76g, 2.65 mmol). **^{1}H NMR** (600 MHz, $CDCl_3$) δ 7.72 (d, J = 7.9 Hz, 1H), 7.33 (t, J = 7.5 Hz, 1H), 7.20 – 7.12 (m, 5H), 7.03 (d, J = 7.0 Hz, 2H), 5.69 (ddt, J = 16.7, 10.2, 6.3 Hz, 1H), 5.11 – 4.72 (m, 2H), 4.17 (d, J = 6.3 Hz, 2H), 2.29 (s, 3H). **^{13}C NMR** (151 MHz, $CDCl_3$) δ 139.0, 138.1, 137.1, 133.1, 132.9, 132.7, 130.4, 129.1, 129.0, 127.9, 126.1, 119.0, 53.8, 20.7. **IR (neat)**: v = 3064, 2930, 2325, 2096, 1870, 1712, 1644, 1593, 1490, 1454, 1336, 1218, 1160, 1066, 992, 926, 857, 806, 763, 732, 695 cm^{-1}. **HRMS(ESI)**: calculated for $C_{16}H_{17}O_2NNaS$ $[M+Na]^+$: 310.0872, found: 310.0864.

N-allyl-2-methoxy-N-phenylbenzenesulfonamide (1d)

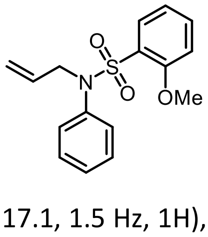

Prepared following general procedure of sulfonamide. **1d** was obtained as a yellow oil in 56% yield (0.85g, 2.80 mmol). **^{1}H NMR** (600 MHz, $CDCl_3$) δ 7.70 (dd, J = 7.9, 1.8 Hz, 1H), 7.48 (ddd, J = 8.8, 7.5, 1.7 Hz, 1H), 7.26 – 7.20 (m, 2H), 7.20 – 7.16 (m, 1H), 7.15 – 7.09 (m, 2H), 7.02 (d, J = 8.3 Hz, 1H), 6.89 (t, J = 7.6 Hz, 1H), 5.84 (ddt, J = 16.5, 10.2, 6.1 Hz, 1H), 5.12 (dd, J = 17.1, 1.5 Hz, 1H), 5.06 (d, J = 10.2 Hz, 1H), 4.45 (d, J = 6.1 Hz, 2H), 3.90 (s, 3H). **^{13}C NMR** (151 MHz, $CDCl_3$) δ 156.7, 139.2, 134.6, 134.0, 131.6, 128.8, 128.5, 127.5, 127.3, 120.2, 118.0, 112.0, 55.9, 54.6. **IR (neat)**: v = 3072, 2978, 2941, 2842, 2332, 2087, 1877, 1735, 1644, 1589, 1480, 1436, 1335, 1280, 1250, 1222, 1156, 1071, 1018, 925, 861, 802, 759, 695 cm^{-1}. **HRMS(ESI)**: calculated for $C_{16}H_{17}O_3NNaS$ $[M+Na]^+$: 326.0821, found: 326.0819.

N-allyl-2-chloro-N-phenylbenzenesulfonamide (1e)

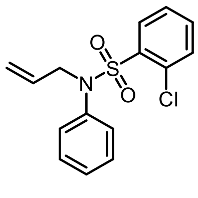

Prepared following general procedure of sulfonamide. **1e** was obtained as a yellow oil in 58% yield (0.89g, 2.90 mmol). **^{1}H NMR** (600 MHz, $CDCl_3$) δ 7.83 (dd, J = 8.0, 1.7 Hz, 1H), 7.53 (dd, J = 7.9, 1.3 Hz, 1H), 7.45 (td, J = 7.7, 1.7 Hz, 1H), 7.29 – 7.21 (m, 4H), 7.20 – 7.15 (m, 2H), 5.88 (ddt, J = 16.6, 10.0, 6.3 Hz, 1H), 5.32 – 4.76 (m, 2H), 4.50 (d, J = 6.3 Hz, 2H). **^{13}C NMR** (151 MHz, $CDCl_3$) δ 138.4, 137.0, 133.8, 133.5, 132.7, 132.2, 131.9, 129.3, 129.1, 128.0, 126.9, 118.8, 55.2. **IR (neat)**: v = 3070, 2923, 2329, 2109, 1875, 1644, 1579, 1491, 1452, 1430, 1339, 1255, 1220, 1161, 1069, 1042, 993, 927, 861, 759, 696, 663 cm^{-1}. **HRMS(ESI)**: calculated for $C_{15}H_{14}O_2NClNaS$ $[M+Na]^+$: 330.0326, found: 330.0323.

N-allyl-2-bromo-N-phenylbenzenesulfonamide (1f)

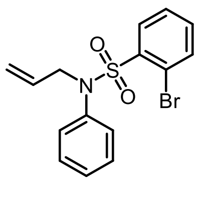

Prepared following general procedure of sulfonamide. **1f** was obtained as a yellow oil in 57% yield (1.00g, 2.85 mmol). **^{1}H NMR** (600 MHz, $CDCl_3$) δ 7.86 (dd, J = 7.9, 1.7 Hz, 1H), 7.73 (dd, J = 7.9, 1.3 Hz, 1H), 7.33 (td, J = 7.6, 1.7 Hz, 1H), 7.27 (td, J = 7.7, 1.4 Hz, 1H), 7.25 – 7.22 (m, 2H), 7.22 – 7.18 (m, 3H), 5.89 (ddt, J = 16.7, 10.1, 6.3 Hz, 1H), 5.31 – 4.85 (m, 2H), 4.51 (d, J = 6.3 Hz, 2H). **^{13}C NMR** (151 MHz, $CDCl_3$) δ 138.5, 138.3, 135.4, 133.7, 133.4, 133.0, 129.3, 129.1, 128.0, 127.4, 120.3, 118.9, 55.4. **IR (neat)**: v = 3068, 2923, 2326, 2107, 1993, 1875, 1735, 1643,

1573, 1490, 1446, 1426, 1336, 1253, 1219, 1161, 1068, 1026, 993, 927, 861, 759, 696 cm^{-1}. **HRMS(ESI)**: calculated for $C_{15}H_{14}O_2NBrNaS$ $[M+Na]^+$: 373.9821, found: 373.9819.

_N_-allyl-_N_-phenyl-2-(trifluoromethyl) benzenesulfonamide (1g)

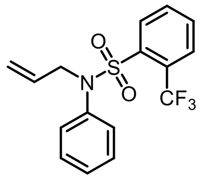

Prepared following general procedure of sulfonamide. **1g** was obtained as a yellow oil in 50% yield (0.85g, 2.50 mmol). **^{1}H NMR** (600 MHz, $CDCl_3$) δ 7.78 (d, J = 7.9 Hz, 1H), 7.66 (d, J = 8.0 Hz, 1H), 7.54 (t, J = 7.7 Hz, 1H), 7.41 (t, J = 7.7 Hz, 1H), 7.21 – 7.09 (m, 3H), 7.07 – 6.87 (m, 2H), 5.72 (ddt, J = 16.8, 10.2, 6.4 Hz, 1H), 5.12 – 4.85 (m, 2H), 4.26 (d, J = 6.4 Hz, 2H). **^{13}C NMR** (151 MHz, $CDCl_3$) δ 138.3, 138.3, 133.1, 132.9, 132.7, 131.9, 129.6, 129.2, 128.2 (q, J =7.6 Hz), 128.2, 127.8 (q, J = 33.2 Hz), 122.6 (q, J = 274.2 Hz), 119.2, 54.6 (d, J = 2.2 Hz). **^{19}F NMR** (376 MHz, $CDCl_3$) δ -57.50. **IR (neat)**: v = 3079, 2926, 2325, 2090, 1880, 1646, 1593, 1491, 1440, 1356, 1306, 1270, 1219, 1161, 1115, 1033, 994, 928, 862, 771, 737, 699 cm^{-1}. **HRMS(ESI)**: calculated for $C_{16}H_{14}O_2NF_3NaS$ $[M+Na]^+$: 364.0590, found: 364.0585.

methyl 2-(_N_-allyl-_N_-phenylsulfamoyl) benzoate (1h)

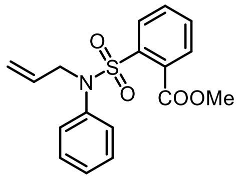

Prepared following general procedure of sulfonamide. **1h** was obtained as a yellow oil in 45% yield (0.74g, 2.25 mmol). **^{1}H NMR** (600 MHz, $CDCl_3$) δ 7.57 (td, J = 7.5, 1.3 Hz, 1H), 7.50 (dd, J = 7.7, 1.4 Hz, 1H), 7.46 (dd, J = 8.1, 1.3 Hz, 1H), 7.41 (ddd, J = 8.0, 7.3, 1.4 Hz, 1H), 7.35 – 7.25 (m, 3H), 7.20 – 7.13 (m, 2H), 5.81 (ddt, J = 17.1, 10.1, 6.2 Hz, 1H), 5.16 – 4.94 (m, 2H), 4.34 (d, J = 6.3 Hz, 2H), 3.89 (s, 3H). **^{13}C NMR** (151 MHz, $CDCl_3$) δ 168.4, 138.8, 136.7, 133.4, 133.2, 132.4, 130.1, 129.9, 129.6, 129.1, 128.3, 128.1, 118.8, 54.3, 53.2. **IR (neat)**: v = 3456, 3069, 3013, 2952, 2327, 2112, 1993, 1902, 1734, 1645, 1592, 1490, 1432, 1348, 1293, 1258, 1222, 1164, 1118, 1063, 994, 928, 862, 831, 765, 696, 657 cm^{-1}. **HRMS(ESI)**: calculated for $C_{17}H_{17}O_4NNaS$ $[M+Na]^+$: 354.0771, found: 354.0767.

_N_-allyl-2,6-dimethoxy-_N_-phenylbenzenesulfonamide (1i)

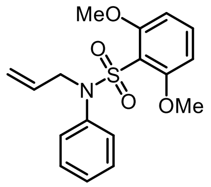

Prepared following general procedure of sulfonamide. **1i** was obtained as a pale yellow solid in 35% yield (0.58g, 1.75 mmol). **^{1}H NMR** (600 MHz, $CDCl_3$) δ 7.29 (t, J = 8.4 Hz, 1H), 7.19 – 7.11 (m, 4H), 7.10 – 7.06 (m, 1H), 6.49 (d, J = 8.4 Hz, 2H), 5.80 (ddt, J = 17.2, 10.2, 5.9 Hz, 1H), 5.07 (dd, J = 17.2, 1.5 Hz, 1H), 5.00 (dd, J = 10.2, 1.4 Hz, 1H), 4.35 (d, J = 6.0 Hz, 2H), 3.70 (s, 6H). **^{13}C NMR** (151 MHz, $CDCl_3$) δ 159.6, 140.1, 134.6, 134.1, 128.8, 127.4, 126.6, 117.8, 117.5, 105.3, 56.7, 54.3. **IR (neat)**: v = 3099, 3060, 2990, 2945, 2846, 2553, 2302, 2118, 1988, 1921, 1824, 1647, 1581, 1475, 1431, 1333, 1254, 1150, 1101, 1047, 982, 909, 860, 773, 723, 694, 655 cm^{-1}. **HRMS(ESI)**: calculated for $C_{17}H_{19}O_4NNaS$ $[M+Na]^+$: 356.0927, found: 356.0922.

_N_-allyl-2,6-dichloro-_N_-phenylbenzenesulfonamide (1j)

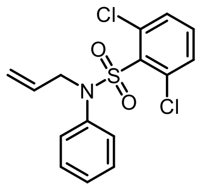

Prepared following general procedure of sulfonamide. **1j** was obtained as a brown oil in 25% yield (0.43g, 1.25 mmol). **^{1}H NMR** (600 MHz, $CDCl_3$) δ 7.30 (d, J = 8.0 Hz, 1H), 7.22 – 7.16 (m, 2H), 7.16 – 7.12 (m, 1H), 5.78 (ddt, J = 16.6, 10.2, 6.3 Hz, 1H), 5.11 – 4.93 (m, 1H), 4.43 (d, J = 6.3 Hz, 1H). **^{13}C NMR** (151 MHz, $CDCl_3$) δ 138.0, 136.3, 134.9, 133.2, 132.7, 131.5, 129.3, 129.1,

128.2, 119.1, 55.2. **IR (neat)**: *v* = 3073, 2983, 2925, 2327, 2079, 1993, 1877, 1644, 1592, 1562, 1491, 1423, 1352, 1170, 1064, 992, 927, 861, 778, 745, 695 cm^{-1}. **HRMS(ESI)**: calculated for $C_{15}H_{13}O_2NCl_2KS$ $[M+K]^+$: 379.9676, found: 379.9674.

N-allyl-2,5-dimethyl-*N*-phenylbenzenesulfonamide (1k)

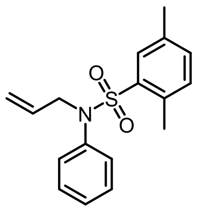

Prepared following general procedure of sulfonamide. **1k** was obtained as a pale yellow oil in 68% yield (1.02g, 3.40 mmol). **^{1}H NMR** (600 MHz, $CDCl_3$) δ 7.55 – 7.51 (m, 1H), 7.19 – 7.14 (m, 3H), 7.14 – 7.11 (m, 1H), 7.05 – 7.01 (m, 3H), 5.68 (ddt, J = 17.2, 10.2, 6.3 Hz, 1H), 5.13 – 4.83 (m, 2H), 4.15 (dt, J = 6.3, 1.3 Hz, 2H), 2.21 (s, 6H). **^{13}C NMR** (151 MHz, $CDCl_3$) δ 139.0, 136.5, 136.0, 134.9, 133.6, 133.1, 132.6, 130.7, 129.0, 129.0, 127.8, 118.9, 53.7, 20.8, 20.2. **IR (neat)**: *v* = 3062, 2978, 2926, 2867, 2326, 2083, 1993, 1870, 1644, 1593, 1490, 1452, 1389, 1333, 1217, 1154, 1066, 992, 926, 882, 854, 823, 771, 730,697 cm^{-1}. **HRMS(ESI)**: calculated for $C_{17}H_{19}O_2NKS$ $[M+K]^+$: 340.0768, found: 340.0764.

N-allyl-2,4-dimethyl-*N*-phenylbenzenesulfonamide (1l)

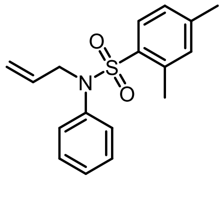

Prepared following general procedure of sulfonamide. **1l** was obtained as a pale yellow oil in 50% yield (0.75g, 2.50 mmol). **^{1}H NMR** (600 MHz, $CDCl_3$) δ 7.59 (d, J = 8.0 Hz, 1H), 7.20 – 7.11 (m, 3H), 7.07 – 7.01 (m, 2H), 6.99 – 6.91 (m, 2H), 5.68 (ddt, J = 16.7, 10.1, 6.3 Hz, 1H), 5.10 – 4.81 (m, 2H), 4.15 (d, J = 6.4 Hz, 2H), 2.25 (s, 3H), 2.23 (s, 3H). **^{13}C NMR** (151 MHz, $CDCl_3$) δ 143.5, 139.1, 138.0, 134.0, 133.3, 133.1, 130.5, 129.0, 129.0, 127.7, 126.8, 118.9, 53.7, 21.3, 20.6. **IR (neat)**: *v* = 3065, 3018, 2979, 2925, 2864, 2323, 2090, 1992, 1914, 1644, 1597, 1490, 1450, 1333, 1218, 1161, 1061, 993, 927, 856, 822, 772, 734, 695, 659 cm^{-1}. **HRMS(ESI)**: calculated for $C_{17}H_{19}O_2NNaS$ $[M+Na]^+$: 324.1029, found: 324.1027.

N-allyl-2,3,4,5,6-pentamethyl-*N*-phenylbenzenesulfonamide (1m)

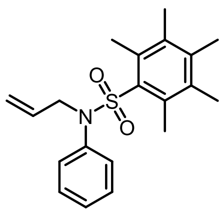

Prepared following general procedure of sulfonamide. **1m** was obtained as a yellow solid in 48% yield (0.82g, 2.40 mmol). **^{1}H NMR** (600 MHz, $CDCl_3$) δ 7.32 – 7.24 (m, 3H), 7.24 – 7.20 (m, 2H), 5.84 (ddt, J = 16.9, 10.5, 6.5 Hz, 1H), 5.13 – 5.02 (m, 2H), 4.33 (d, J = 6.2 Hz, 2H), 2.43 (s, 6H), 2.29 (s, 3H), 2.22 (s, 6H). **^{13}C NMR** (151 MHz, $CDCl_3$) δ 140.2, 139.2, 136.1, 134.8, 134.7, 133.4, 130.0, 129.1, 128.0, 119.0, 53.1, 18.9, 18.0, 17.2. **IR (neat)**: *v* = 3068, 2924, 2318, 2108, 1859, 1739, 1644, 1592, 1549, 1488, 1452, 1413, 1297, 1268, 1216, 1143, 1069, 1027, 985, 925, 844, 749, 727, 696 cm^{-1}. **HRMS(ESI)**: calculated for $C_{20}H_{25}O_2NNaS$ $[M+Na]^+$: 366.1498, found: 366.1490.

N-allyl-2,4,6-triisopropyl-*N*-phenylbenzenesulfonamide (1n)

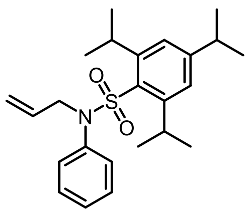

Prepared following general procedure of sulfonamide. **1n** was obtained as a yellow oil in 40% yield (0.80g, 2.00 mmol). **^{1}H NMR** (600 MHz, $CDCl_3$) δ 7.21 – 7.13 (m, 3H), 7.11 – 7.08 (m, 2H), 7.01 (s, 2H), 5.71 (ddt, J = 18.8, 9.8, 6.5 Hz, 1H), 4.99 – 4.90 (m, 2H), 4.22 (d, J = 6.6 Hz, 2H), 3.85 (hept, J = 6.8 Hz, 2H), 2.80 (hept, J = 6.9 Hz, 1H), 1.15 (d, J = 7.0 Hz, 6H), 1.05 (d, J = 6.8 Hz, 12H). **^{13}C NMR** (151 MHz, $CDCl_3$) δ 153.2, 151.5,

139.0, 133.3, 131.9, 130.4, 129.2, 128.1, 123.8, 119.1, 53.2, 34.2, 29.6, 24.8, 23.6. **IR (neat)**: *v* = 3414, 2959, 2870, 2326, 2112, 1871, 1715, 1644, 1598, 1562, 1491, 1459, 1424, 1364, 1318, 1261, 1215, 1156, 1048, 992, 927, 880, 851, 771, 729, 695, 664 cm^{-1}. **HRMS(ESI)**: calculated for $C_{24}H_{33}O_2NNaS$ $[M+Na]^+$: 422.2124, found: 422.2117.

***N*-allyl-4-methoxy-*N*-phenylbenzenesulfonamide (1o)**

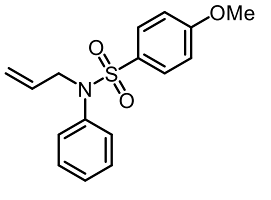

Prepared following general procedure of sulfonamide. **1o** was obtained as a white solid in 46% yield (0.70g, 2.30 mmol). **^{1}H NMR** (600 MHz, $CDCl_3$) δ 7.46 (d, J = 8.9 Hz, 2H), 7.27 – 7.12 (m, 3H), 6.98 (d, J = 6.7 Hz, 2H), 6.85 (d, J = 8.8 Hz, 2H), 5.67 (ddt, J = 16.6, 10.0, 6.3 Hz, 1H), 5.10 – 4.83 (m, 2H), 4.10 (d, J = 6.0 Hz, 2H), 3.79 (s, 3H). **^{13}C NMR** (151 MHz, $CDCl_3$) δ 163.0, 139.4, 133.0, 130.3, 130.0, 129.0, 129.0, 127.9, 118.9, 114.1, 55.7, 53.6. **IR (neat)**: *v* = 3074, 3007, 2965, 2934, 2836, 2687, 2507, 2294, 2159, 2056, 1992, 1887, 1729, 1641, 1591, 1492, 1456, 1423, 1342, 1305, 1251, 1216, 1154, 1089, 1055, 1027, 997, 939, 838, 803, 778, 734, 700, 668 cm^{-1}. **HRMS(ESI)**: calculated for $C_{16}H_{17}O_3NNaS$ $[M+Na]^+$: 326.0821, found: 326.0825.

***N*-allyl-*N*-phenyl-4-(trifluoromethyl)benzenesulfonamide (1p)**

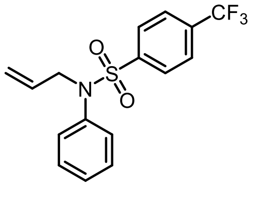

Prepared following general procedure of sulfonamide. **1p** was obtained as a white solid in 62% yield (1.06g, 3.10 mmol). **^{1}H NMR** (600 MHz, $CDCl_3$) δ 7.66 (s, 4H), 7.28 – 7.20 (m, 3H), 6.98 – 6.77 (m, 2H), 5.67 (ddt, J = 16.6, 10.2, 6.3 Hz, 1H), 5.13 – 4.90 (m, 2H), 4.14 (d, J = 6.3 Hz, 2H). **^{13}C NMR** (151 MHz, $CDCl_3$) δ 142.3, 138.6, 134.5 (d, J = 33.2 Hz), 132.4, 129.3, 129.0, 128.4, 128.3, 126.1 (q, J = 3.8 Hz), 123.4 (d, J = 273.1 Hz), 119.5, 54.0. **^{19}F NMR** (376 MHz, $CDCl_3$) δ -63.08. **IR (neat)**: *v* = 3072, 2925, 2323, 2158, 1994, 1809, 1687, 1642, 1593, 1493, 1453, 1405, 1350, 1320, 1212, 1161, 1127, 1059, 1016, 932, 849, 775, 734, 699 cm^{-1}. **HRMS(ESI)**: calculated for $C_{16}H_{14}O_2NF_3NaS$ $[M+Na]^+$: 364.0590, found: 364.0596.

***N*-allyl-3-methyl-*N*-phenylbenzenesulfonamide (1q)**

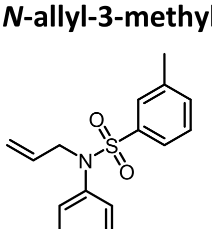

Prepared following general procedure of sulfonamide. **1q** was obtained as a yellow solid in 60% yield (0.86g, 3.00 mmol). **^{1}H NMR** (600 MHz, $CDCl_3$) δ 7.45 – 7.38 (m, 3H), 7.37 – 7.34 (m, 1H), 7.33 – 7.27 (m, 3H), 7.10 – 7.04 (m, 2H), 5.76 (ddt, J = 17.1, 10.2, 6.3 Hz, 1H), 5.27 – 4.89 (m, 2H), 4.21 (d, J = 6.3 Hz, 2H), 2.39 (s, 3H).**^{13}C NMR** (151 MHz, $CDCl_3$) δ 139.2, 139.1, 138.3, 133.5, 132.9, 129.0, 128.9, 128.8, 128.1, 127.9, 124.9, 118.9, 53.7, 21.4. **IR (neat)**: *v* = 3061, 2919, 2322, 2113, 1881, 1805, 1763, 1645, 1593, 1489, 1456, 1423, 1338, 1272, 1211, 1151, 1087, 1058, 1000, 931, 877, 795, 771, 732, 689 cm^{-1}. **HRMS(ESI)**: calculated for $C_{16}H_{17}O_2NNaS$ $[M+Na]^+$: 310.0872, found: 310.0868.

***N*-allyl-*N*-phenylnaphthalene-1-sulfonamide (1r)**

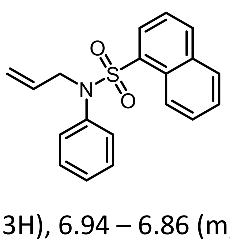

Prepared following general procedure of sulfonamide. **1r** was obtained as a yellow oil in 35% yield (0.57g, 1.75 mmol). **^{1}H NMR** (600 MHz, $CDCl_3$) δ 8.32 (dd, J = 8.5, 1.1 Hz, 1H), 7.96 (dd, J = 7.4, 1.3 Hz, 1H), 7.86 (d, J = 8.2 Hz, 1H), 7.79 – 7.71 (m, 1H), 7.38 (ddd, J = 8.1, 6.8, 1.2 Hz, 1H), 7.33 (ddd, J = 8.4, 6.8, 1.5 Hz, 1H), 7.27 (dd, J = 8.2, 7.3 Hz, 1H), 7.07 – 7.01 (m, 3H), 6.94 – 6.86 (m, 2H), 5.62 (ddt, J = 16.7, 10.2, 6.3 Hz, 1H), 5.00 – 4.76 (m, 2H), 4.15 (dt, J = 6.4, 1.3 Hz, 2H). **^{13}C NMR** (151 MHz, $CDCl_3$) δ 138.7, 134.4, 134.1, 134.1, 132.9, 130.7, 129.3, 128.8, 128.7, 128.7, 127.8, 127.7, 126.7, 125.3, 124.0, 118.9, 53.6. **IR (neat)**: *v* = 3062, 2921, 2865, 2323, 2093, 1881, 1714, 1643, 1592, 1492, 1452, 1338, 1205, 1159, 1134, 1062, 1029, 986, 926, 858, 805, 769, 733, 694 cm^{-1}. **HRMS(ESI)**: calculated for $C_{19}H_{17}O_2NNaS$ [M+Na]$^+$: 346.0872, found: 346.0862.

***N*-allyl-*N*-phenylquinoline-8-sulfonamide (1s)**

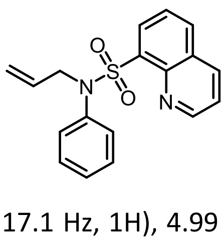

Prepared following general procedure of sulfonamide. **1s** was obtained as a brown solid in 32% yield (0.52g, 1.60 mmol). **^{1}H NMR** (600 MHz, $CDCl_3$) δ 9.10 – 8.95 (m, 1H), 8.17 (t, J = 7.9 Hz, 2H), 7.90 (d, J = 8.1 Hz, 1H), 7.48 (dd, J = 8.4, 4.2 Hz, 1H), 7.38 (t, J = 7.8 Hz, 1H), 7.06 – 6.99 (m, 3H), 6.93 – 6.80 (m, 2H), 5.86 (ddt, J = 16.5, 11.0, 6.1 Hz, 1H), 5.06 (d, J = 17.1 Hz, 1H), 4.99 (d, J = 10.2 Hz, 1H), 4.77 (d, J = 6.2 Hz, 2H). **^{13}C NMR** (151 MHz, $CDCl_3$) δ 151.2, 144.2, 139.4, 137.6, 136.7, 134.9, 133.6, 133.5, 129.0, 128.9, 127.5, 125.5, 122.2, 117.8, 56.3. **IR (neat)**: *v* = 3074, 3028, 2981, 2919, 2861, 2327, 2086, 1992, 1899, 1836, 1695, 1645, 1593, 1558, 1489, 1447, 1383, 1328, 1282, 1208, 1138, 1070, 1027, 994, 914, 866, 829, 785, 734, 699, 699 cm^{-1}. **HRMS(ESI)**: calculated for $C_{18}H_{16}O_2N_2NaS$ [M+Na]$^+$: 347.0825, found: 347.0820.

***N*-allyl-2,4,6-trimethyl-*N*-(*p*-tolyl)benzenesulfonamide (1t)**

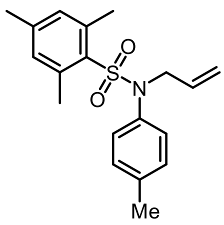

Prepared following general procedure of sulfonamide. **1t** was obtained as a white solid in 73% yield (1.20g, 3.65 mmol). **^{1}H NMR** (600 MHz, $CDCl_3$) δ 7.08 – 7.04 (m, 2H), 7.03 – 7.00 (m, 2H), 6.88 (s, 2H), 5.81 (ddt, *J* = 16.8, 10.2, 6.5 Hz, 1H), 5.08 (dq, *J* = 9.4, 1.3 Hz, 1H), 5.07 – 5.05 (m, 1H), 4.30 (dt, *J* = 6.5, 1.3 Hz, 2H), 2.47 (s, 6H), 2.30 (s, 3H), 2.28 (s, 3H). **^{13}C NMR** (151 MHz, $CDCl_3$) δ 142.5, 140.5, 137.9, 136.3, 133.5, 133.0, 131.8, 129.7, 129.6, 118.9, 53.5, 23.1, 21.2, 21.1. **IR (neat)**: *v* = 2975, 2923, 2865, 2739, 2322, 2165, 1909, 1645, 1602, 1565, 1508, 1451, 1378, 1316, 1226, 1189, 1151, 1080, 1050, 991, 927, 858, 793, 706, 663 cm^{-1}. **HRMS(ESI)**: calculated for $C_{19}H_{23}O_2NNaS$ [M+Na]$^+$: 352.1342, found: 352.1340.

***N*-allyl-*N*-(4-methoxyphenyl)-2,4,6-trimethylbenzenesulfonamide (1u)**

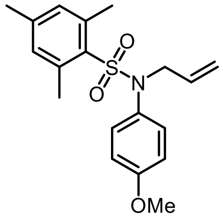

Prepared following general procedure of sulfonamide. **1u** was obtained as a yellow solid in 76% yield (1.31g, 3.80 mmol). **^{1}H NMR** (600 MHz, $CDCl_3$) δ 6.95 – 6.91 (m, 2H), 6.79 (s, 2H), 6.72 – 6.63 (m, 2H), 5.72 (ddt, *J* = 16.9, 10.4, 6.6 Hz, 1H), 5.03 – 4.91 (m, 2H), 4.19 (dt, *J* = 6.5, 1.3 Hz, 2H), 3.69 (s, 3H), 2.36 (s, 6H), 2.20 (s, 3H). **^{13}C NMR** (151 MHz, $CDCl_3$) δ 159.2, 142.5, 140.4, 133.5, 133.0, 131.8, 131.4, 131.3, 119.0, 114.2, 55.5, 53.7, 23.1,

21.1. **IR (neat)**: *v* = 2981, 2935, 2842, 2322, 2089, 1888, 1744, 1646, 1603, 1505, 1456, 1319, 1299, 1242, 1152, 1102, 1064, 1030, 923, 853, 791, 733, 702, 661 cm^{-1}. **HRMS(ESI)**: calculated for $C_{19}H_{23}O_3NNaS$ [M+Na]$^+$: 368.1291, found: 368.1291.

***N*-allyl-*N*-(4-chlorophenyl)-2,4,6-trimethylbenzenesulfonamide (1v)**

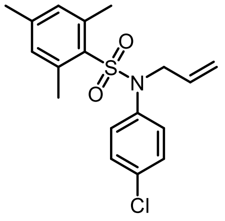

Prepared following general procedure of sulfonamide. **1v** was obtained as a yellow solid in 80% yield (1.40g, 4.00 mmol). **^{1}H NMR** (600 MHz, $CDCl_3$) *δ* 7.16 – 7.13 (m, 2H), 6.99 – 6.95 (m, 2H), 6.81 (s, 2H), 5.69 (ddt, *J* = 16.8, 10.2, 6.5 Hz, 1H), 5.02 – 4.81 (m, 2H), 4.18 (d, *J* = 6.5 Hz, 2H), 2.37 (s, 6H), 2.20 (s, 3H). **^{13}C NMR** (151 MHz, $CDCl_3$) *δ* 142.9, 140.4, 137.7, 133.8, 133.0, 132.5, 132.0, 131.0, 129.3, 119.5, 53.4, 23.1, 21.1. **IR (neat)**: *v* = 3409, 3083, 2981, 2936, 2877, 2316, 2091, 1861, 1646, 1599, 1565, 1487, 1407, 1378, 1316, 1266, 1207, 1155, 1085, 1045, 1013, 993, 925, 856, 820, 755, 717, 661 cm^{-1}. **HRMS(ESI)**: calculated for $C_{18}H_{20}O_2NClNaS$ [M+Na]$^+$: 372.0796, found: 372.0798.

***N*-allyl-*N*-(4-bromophenyl)-2,4,6-trimethylbenzenesulfonamide (1w)**

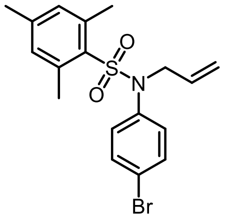

Prepared following general procedure of sulfonamide. **1w** was obtained as a yellow solid in 72% yield (1.41g, 3.60 mmol). **^{1}H NMR** (600 MHz, $CDCl_3$) *δ* 7.32 – 7.28 (m, 2H), 6.93 – 6.89 (m, 2H), 6.81 (s, 2H), 5.68 (ddt, *J* = 16.8, 10.2, 6.5 Hz, 1H), 5.05 – 4.89 (m, 2H), 4.18 (d, *J* = 6.5 Hz, 2H), 2.37 (s, 6H), 2.20 (s, 3H). **^{13}C NMR** (151 MHz, $CDCl_3$) *δ* 142.9, 140.4, 138.3, 132.9, 132.5, 132.3, 132.0, 131.3, 121.9, 119.5, 53.4, 23.1, 21.1. **IR (neat)**: *v* = 3402, 3081, 2981, 2935, 2878, 2316, 2120, 1861, 1646, 1598, 1565, 1483, 1402, 1317, 1265, 1205, 1155, 1102, 1047, 1007, 926, 858, 818, 748, 712, 660 cm^{-1}. **HRMS(ESI)**: calculated for $C_{18}H_{20}O_2NBrNaS$ [M+Na]$^+$: 416.0290, found: 416.0292.

***N*-allyl-*N*-(4-fluorophenyl)-2,4,6-trimethylbenzenesulfonamide (1x)**

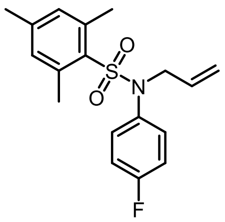

Prepared following general procedure of sulfonamide. **1x** was obtained as a white solid in 85% yield (1.42g, 4.25 mmol). **^{1}H NMR** (600 MHz, $CDCl_3$) *δ* 7.09 – 7.03 (m, 1H), 6.96 – 6.89 (m, 1H), 6.87 (s, 1H), 5.77 (ddt, *J* = 16.8, 10.1, 6.6 Hz, 1H), 5.12 – 5.00 (m, 1H), 4.26 (dt, *J* = 6.6, 1.3 Hz, 1H), 2.43 (s, 3H), 2.27 (s, 1H). **^{13}C NMR** (151 MHz, $CDCl_3$) *δ* 162.0 (d, *J* = 248.3 Hz), 142.8, 140.4, 134.9 (d, *J* = 3.2 Hz), 133.1, 132.5, 131.9, 131.8 (d, *J* = 8.8 Hz), 119.4, 116.0 (d, *J* = 22.4 Hz), 53.6, 23.0, 21.1. **^{19}F NMR** (564 MHz, $CDCl_3$) *δ* -112.93 – -113.00 (m). **IR (neat)**: *v* = 3082, 2974, 2933, 2871, 2321, 2112, 1855, 1733, 1646, 1598, 1564, 1501, 1460, 1410, 1381, 1343, 1307, 1268, 1219, 1197, 1150, 1091, 1036, 995, 923, 867, 807, 700, 661 cm^{-1}. **HRMS(ESI)**: calculated for $C_{18}H_{20}O_2NFNaS$ [M+Na]$^+$: 356.1091, found: 356.1084.

_N_-allyl-2,4,6-trimethyl-_N_-(4-(trifluoromethyl)phenyl)benzenesulfonamide (1y)

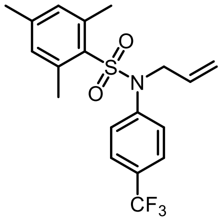

Prepared following general procedure of sulfonamide. **1y** was obtained as a white solid in 29% yield (0.56g, 1.45 mmol). **^{1}H NMR** (600 MHz, $CDCl_3$) δ 7.52 (d, J = 8.2 Hz, 2H), 7.28 – 7.24 (m, 2H), 6.90 (s, 2H), 5.75 (ddt, J = 16.8, 10.3, 6.4 Hz, 1H), 5.14 – 4.98 (m, 2H), 4.29 (d, J = 6.5 Hz, 2H), 2.46 (s, 6H), 2.28 (s, 3H). **^{13}C NMR** (151 MHz, $CDCl_3$) δ 143.2, 142.7, 140.4, 132.7, 132.5, 132.2, 129.6 (d, J = 33.0 Hz), 129.1, 126.2 (q, J = 3.8 Hz), 119.7, 53.2, 23.1, 21.1. **^{19}F NMR** (564 MHz, $CDCl_3$) δ -62.57. **IR (neat)**: v = 3076, 2979, 2940, 2335, 2162, 1744, 1605, 1515, 1459, 1414, 1323, 1215, 1159, 1129, 1066, 1016, 930, 867, 781, 749, 722, 658 cm^{-1}. **HRMS(ESI)**: calculated for $C_{19}H_{20}O_2NF_3NaS$ $[M+Na]^+$: 406.1059, found: 406.1061.

_N_-allyl-2,4,6-trimethyl-_N_-(4-phenoxyphenyl)benzenesulfonamide (1z)

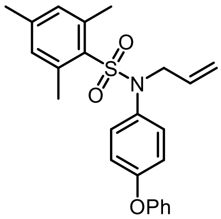

Prepared following general procedure of sulfonamide. **1z** was obtained as a white solid in 74% yield (1.51g, 3.70 mmol). **^{1}H NMR** (600 MHz, $CDCl_3$) δ 7.28 – 7.24 (m, 2H), 7.06 – 7.02 (m, 1H), 6.99 – 6.94 (m, 2H), 6.90 – 6.87 (m, 2H), 6.82 – 6.75 (m, 4H), 5.73 (ddt, J = 17.7, 9.5, 6.5 Hz, 1H), 5.05 – 4.95 (m, 2H), 4.20 (d, J = 6.5 Hz, 2H), 2.38 (s, 6H), 2.20 (s, 3H). **^{13}C NMR** (151 MHz, $CDCl_3$) δ 157.0, 156.8, 142.7, 140.5, 133.8, 133.3, 132.8, 131.9, 131.4, 130.0, 123.8, 119.3, 119.2, 118.9, 53.6, 23.1, 21.1. **IR (neat)**: v = 3069, 3037, 2940, 2322, 2028, 1872, 1732, 1643, 1589, 1489, 1460, 1335, 1292, 1242, 1209, 1156, 1068, 1016, 984, 930, 856, 797, 763, 724, 691, 656 cm^{-1}. **HRMS(ESI)**: calculated for $C_{24}H_{25}O_3NNaS$ $[M+Na]^+$: 430.1447, found: 430.1444.

_N_-allyl-2,4,6-trimethyl-_N_-(_m_-tolyl)benzenesulfonamide (1aa)

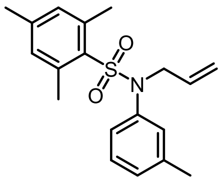

Prepared following general procedure of sulfonamide. **1aa** was obtained as a yellow oil in 50% yield (0.82g, 2.50 mmol). **^{1}H NMR** (600 MHz, $CDCl_3$) δ 7.13 (t, J = 7.7 Hz, 1H), 7.07 – 7.03 (m, 1H), 6.99 (d, J = 2.0 Hz, 1H), 6.92 – 6.86 (m, 3H), 5.81 (ddt, J = 16.7, 10.2, 6.4 Hz, 1H), 5.11 – 5.04 (m, 2H), 4.30 (d, J = 6.5 Hz, 2H), 2.48 (s, 6H), 2.28 (s, 3H), 2.27 (s, 3H). **^{13}C NMR** (151 MHz, $CDCl_3$) δ 142.5, 140.4, 138.9, 138.8, 133.3, 132.9, 131.7, 130.1, 128.7, 128.6, 126.4, 118.8, 53.3, 23.0, 21.2, 21.0. **IR (neat)**: v = 2979, 2933, 2864, 2328, 2163, 2082, 1943, 1782, 1736, 1644, 1601, 1485, 1451, 1323, 1244, 1155, 1058, 991, 923, 897, 853, 811, 695 cm^{-1}. **HRMS(ESI)**: calculated for $C_{19}H_{23}O_2NNaS$ $[M+Na]^+$: 352.1342, found: 352.1340.

_N_-allyl-_N_-(3-methoxyphenyl)-2,4,6-trimethylbenzenesulfonamide (1ab)

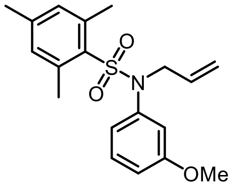

Prepared following general procedure of sulfonamide. **1ab** was obtained as a colorless oil in 61% yield (1.05g, 3.05 mmol). **^{1}H NMR** (600 MHz, $CDCl_3$) δ 7.03 (t, J = 8.1 Hz, 1H), 6.77 (s, 2H), 6.69 – 6.65 (m, 1H), 6.64 – 6.61 (m, 1H), 6.55 (t, J = 2.3 Hz, 1H), 5.70 (ddt, J = 16.7, 10.1, 6.4 Hz, 1H), 5.01 – 4.91 (m, 2H), 4.19 (dt, J = 6.4, 1.3 Hz, 2H), 3.55 (s, 3H), 2.36 (s, 6H), 2.15 (s, 3H). **^{13}C NMR** (151 MHz, $CDCl_3$) δ 159.8, 142.5, 140.3, 140.1, 133.2, 132.8, 131.8, 129.5, 121.4, 118.8, 114.9, 113.7, 55.2, 53.2, 22.9, 20.9. **IR (neat)**: v = 3196, 3081, 2939, 2839, 2319, 2162, 2085, 2013, 1968, 1736, 1645, 1597, 1485, 1455, 1320, 1285, 1256, 1194, 1154, 1042,

992, 924, 899, 854, 806, 720, 691 cm^{-1}. **HRMS(ESI)**: calculated for $C_{19}H_{23}O_3NNaS$ $[M+Na]^+$: 368.1291, found: 368.1291.

***N*-allyl-*N*-(3-bromophenyl)-2,4,6-trimethylbenzenesulfonamide (1ac)**

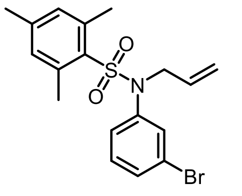

Prepared following general procedure of sulfonamide. **1ac** was obtained as a pale yellow oil in 75% yield (1.47g, 3.75 mmol). **^{1}H NMR** (400 MHz, $CDCl_3$) *δ* 7.36 – 7.22 (m, 2H), 7.13 – 6.97 (m, 2H), 6.86 (s, 2H), 5.79 – 5.66 (m, 1H), 5.09 – 4.98 (m, 2H), 4.23 (d, *J* = 6.4 Hz, 2H), 2.43 (s, 6H), 2.24 (s, 3H). **^{13}C NMR** (101 MHz, $CDCl_3$) *δ* 143.0, 140.5, 140.3, 132.7, 132.3, 131.9, 130.9, 130.2, 129.0, 127.9, 122.1, 119.4, 53.2, 23.0, 21.0. **IR (neat)**: *v* = 3080, 2979, 2937, 2862, 2743, 2323, 2187, 2071, 1873, 1735, 1644, 1569, 1470, 1415, 1324, 1216, 1155, 1057, 997, 926, 857, 788, 757, 691 cm^{-1}. **HRMS(ESI)**: calculated for $C_{18}H_{20}O_2NBrNaS$ $[M+Na]^+$: 416.0290, found: 416.0291.

***N*-allyl-*N*-(3,5-dimethylphenyl)-2,4,6-trimethylbenzenesulfonamide (1ad)**

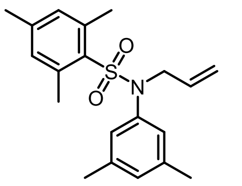

Prepared following general procedure of sulfonamide. **1ae** was obtained as a yellow solid in 30% yield (0.51g, 1.50 mmol). **^{1}H NMR** (400 MHz, $CDCl_3$) *δ* 6.88 – 6.80 (m, 3H), 6.71 (s, 2H), 5.75 (ddt, *J* = 16.9, 12.1, 6.4 Hz, 1H), 5.10 – 4.97 (m, 2H), 4.21 (d, *J* = 6.4 Hz, 2H), 2.45 (s, 6H), 2.25 (s, 3H), 2.18 (s, 6H). **^{13}C NMR** (101 MHz, $CDCl_3$) *δ* 142.5, 140.5, 138.9, 138.6, 133.5, 133.0, 131.8, 129.6, 127.0, 118.6, 53.3, 23.1, 21.2, 21.0. **IR (neat)**: *v* = 2918, 2860, 2736, 2465, 2307, 2003, 1870, 1746, 1644, 1600, 1463, 1379, 1312, 1150, 1066, 1030, 993, 928, 854, 782, 688 cm^{-1}. **HRMS(ESI)**: calculated for $C_{20}H_{25}O_2NNaS$ $[M+Na]^+$: 366.1498, found: 366.1495.

***N*-allyl-2,4,6-trimethyl-*N*-(*o*-tolyl)benzenesulfonamide (1ae)**

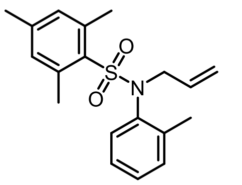

Prepared following general procedure of sulfonamide. **1ae** was obtained as a white solid in 35% yield (0.58g, 1.75 mmol). **^{1}H NMR** (600 MHz, $CDCl_3$) *δ* 7.20 (t, *J* = 7.3 Hz, 1H), 7.13 (q, *J* = 7.3 Hz, 3H), 6.87 (s, 2H), 5.86 (ddd, *J* = 17.0, 10.2, 5.3 Hz, 1H), 5.05 (dd, *J* = 19.7, 13.5 Hz, 2H), 4.34 (d, *J* = 6.9 Hz, 2H), 2.34 (s, 6H), 2.29 (s, 3H), 1.98 (s, 3H). **^{13}C NMR** (101 MHz, $CDCl_3$) *δ* 157.2, 141.8, 140.6, 134.2, 133.8, 131.3, 129.8, 126.2, 120.5, 118.3, 111.7, 55.0, 52.8, 23.2, 21.0. **IR (neat)**: *v* = 3064, 2978, 2932, 2735, 2464, 2321, 2192, 2044, 1985, 1944, 1871, 1739, 1642, 1601, 1566, 1491, 1457, 1377, 1315, 1271, 1216, 1187, 1158, 1113, 1029, 988, 927, 855, 799, 766, 713, 661 cm^{-1}. **HRMS(ESI)**: calculated for $C_{19}H_{23}O_2NNaS$ $[M+Na]^+$: 352.1342, found: 352.1340.

***N*-allyl-*N*-(2-methoxyphenyl)-2,4,6-trimethylbenzenesulfonamide (1af)**

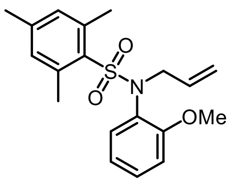

Prepared following general procedure of sulfonamide. **1af** was obtained as a yellow solid in 52% yield (0.90g, 2.60 mmol). **^{1}H NMR** (400 MHz, $CDCl_3$) *δ* 7.34 (dt, *J* = 7.6, 1.7 Hz, 1H), 7.25 – 7.15 (m, 1H), 6.91 – 6.82 (m, 1H), 6.79 (s, 2H), 6.68 (d, *J* = 8.3 Hz, 1H), 5.91 – 5.72 (m, 1H), 5.12 – 4.92 (m, 2H), 4.34 (s, 2H), 3.37 (s, 3H), 2.37 (s, 6H), 2.22 (s, 3H). **^{13}C NMR** (151 MHz, $CDCl_3$) *δ* 157.2, 141.8, 140.5, 134.1, 134.1, 133.7, 131.3, 129.8, 126.1, 120.5, 118.2, 111.6, 55.0, 52.7, 23.1, 20.9. **IR (neat)**: *v* = 3077, 2981, 2937, 2842, 2324, 2222, 2160, 2074, 1995,

1912, 1862, 1798, 1740, 1646, 1596, 1495, 1456, 1380, 1311, 1283, 1256, 1215, 1145, 1113, 1069, 1024, 991, 927, 856, 784, 758, 729, 676, 656 cm^{-1}. **HRMS(ESI)**: calculated for $C_{19}H_{23}O_3NNaS$ $[M+Na]^+$: 368.1291, found: 368.1290.

***N*-allyl-*N*-(2-bromophenyl)-2,4,6-trimethylbenzenesulfonamide (1ag)**

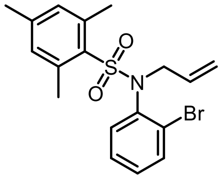

Prepared following general procedure of sulfonamide. **1ag** was obtained as a white solid in 56% yield (1.14g, 2.80 mmol). **^{1}H NMR** (600 MHz, $CDCl_3$) *δ* 7.57 (dd, *J* = 8.0, 1.6 Hz, 1H), 7.50 (dd, *J* = 8.0, 1.5 Hz, 1H), 7.32 (td, *J* = 7.6, 1.5 Hz, 1H), 7.17 (td, *J* = 7.6, 1.6 Hz, 1H), 6.87 (s, 2H), 5.98 (ddt, *J* = 16.9, 10.1, 6.8 Hz, 1H), 5.13 – 5.01 (m, 2H), 4.73 – 4.55 (m, 1H), 4.23 – 4.12 (m, 1H), 2.41 (s, 6H), 2.29 (s, 3H). **^{13}C NMR** (151 MHz, $CDCl_3$) *δ* 142.5, 140.4, 137.1, 135.0, 134.0, 133.8, 133.3, 131.9, 129.8, 127.9, 125.4, 119.6, 54.2, 23.7, 21.1. **IR (neat)**: *ν* = 3070, 2976, 2931, 2873, 2734, 2311, 2113, 1995, 1866, 1734, 1641, 1601, 1566, 1467, 1428, 1376, 1316, 1276, 1210, 1158, 1052, 1028, 990, 925, 850, 770, 748, 717, 661 cm^{-1}. **HRMS(ESI)**: calculated for $C_{18}H_{20}O_2NBrNaS$ $[M+Na]^+$: 416.0290, found: 416.0289.

***N*-allyl-*N*-(benzo[*d*][1,3]dioxol-5-yl)-2,4,6-trimethylbenzenesulfonamide (1ah)**

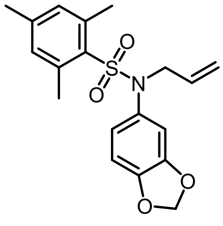

Prepared following general procedure of sulfonamide. **1ah** was obtained as a yellow solid in 57% yield (1.02g, 2.85 mmol). **^{1}H NMR** (600 MHz, $CDCl_3$) *δ* 6.90 (s, 2H), 6.69 – 6.63 (m, 2H), 6.56 (dd, *J* = 8.3, 2.1 Hz, 1H), 5.96 (s, 2H), 5.80 (ddt, *J* = 16.6, 9.9, 6.5 Hz, 1H), 5.30 – 4.97 (m, 2H), 4.25 – 4.23 (m, 2H), 2.49 (s, 6H), 2.30 (s, 3H). **^{13}C NMR** (151 MHz, $CDCl_3$) *δ* 147.8, 147.3, 142.6, 140.4, 133.3, 132.8, 131.9, 123.8, 119.2, 110.7, 108.1, 101.7, 53.8, 23.1, 21.1. **IR (neat)**: *ν* = 3072, 2978, 2900, 2781, 2296, 2110, 1994, 1846, 1756, 1646, 1602, 1560, 1480, 1337, 1238, 1194, 1157, 1098, 1072, 1038, 987, 936, 880, 838, 722, 681, 658 cm^{-1}. **HRMS(ESI)**: calculated for $C_{19}H_{21}O_4NNaS$ $[M+Na]^+$: 382.1084, found: 382.1071.

Butyl 4-((*N*-allyl-2,4,6-trimethylphenyl)sulfonamido)benzoate (1ai)

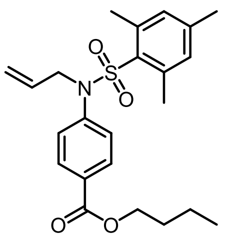

Prepared following general procedure of sulfonamide. **1ai** was obtained as a yellow oil in 18% yield (0.37g, 0.90 mmol). **^{1}H NMR** (600 MHz, $CDCl_3$) *δ* 7.85 (d, *J* = 8.5 Hz, 2H), 7.13 (d, *J* = 8.5 Hz, 2H), 6.80 (s, 2H), 5.69 (ddt, *J* = 16.8, 10.2, 6.4 Hz, 1H), 5.09 – 4.79 (m, 2H), 4.24 (d, *J* = 6.3 Hz, 2H), 4.21 (t, *J* = 6.6 Hz, 2H), 2.38 (s, 6H), 2.19 (s, 3H), 1.72 – 1.57 (m, 2H), 1.46 – 1.30 (m, 2H), 0.89 (t, *J* = 7.4 Hz, 3H). **^{13}C NMR** (151 MHz, $CDCl_3$) *δ* 166.0, 143.6, 143.0 140.3, 132.8, 132.1, 130.4, 129.3, 128.3, 119.4, 65.1, 52.9, 30.8, 23.0, 21.0, 19.3, 13.8. **IR (neat)**: *ν* = 3417, 2959, 2872, 2325, 2085, 1991, 1923, 1716, 1645, 1603, 1566, 1506, 1457, 1410, 1385, 1327, 1273, 1157, 1107, 1056, 1020, 927, 851, 775, 739, 701, 666 cm^{-1}. **HRMS(ESI)**: calculated for $C_{23}H_{29}O_4NNaS$ $[M+Na]^+$: 438.1710, found: 438.1697.

(*E*)-*N*-(but-2-en-1-yl)-2,4,6-trimethyl-*N*-phenylbenzenesulfonamide (1al)

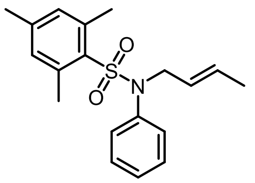

Prepared following general procedure of sulfonamide. **1al** was obtained as a yellow oil in 69% yield (1.14g, 3.45 mmol). **^{1}H NMR** (600 MHz, $CDCl_3$) δ 7.27 – 7.20 (m, 3H), 7.14 – 7.09 (m, 2H), 6.90 – 6.83 (m, 2H), 5.48 – 5.43 (m, 2H), 4.29 – 4.20 (m, 2H), 2.45 (s, 6H), 2.27 (s, 3H), 1.64 – 1.54 (m, 3H). **^{13}C NMR** (151 MHz, $CDCl_3$) *δ* 142.4, 140.3, 139.1, 133.0, 131.7, 130.4, 129.8, 128.9, 127.8, 125.9, 52.8, 22.9, 21.0, 17.7. **IR (neat)**: *v* = 3024, 2973, 2929, 2859, 2321, 2076, 1736, 1597, 1486, 1447, 1320, 1206, 1153, 1033, 966, 918, 849, 770, 693, 582 cm^{-1}. **HRMS(ESI)**: calculated for $C_{19}H_{24}O_2NS$ $[M+H]^+$: 330.1450, found: 330.1523.

N-(cyclohex-2-en-1-yl)-2,4,6-trimethyl-*N*-phenylbenzenesulfonamide(1am)

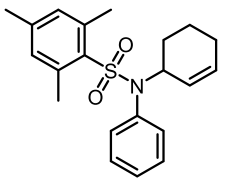

Prepared following general procedure of sulfonamide. **1am** was obtained as a yellow solid in 56% yield (0.99g, 2.80 mmol). **^{1}H NMR** (600 MHz, $CDCl_3$) *δ* 7.32 – 7.20 (m, 3H), 7.16 – 7.09 (m, 2H), 6.87 (s, 2H), 5.86 – 5.81 (m, 1H), 5.79 – 5.73 (m, 1H), 5.09 – 4.98 (m, 1H), 2.44 (s, 6H), 2.28 (s, 3H), 2.08 – 1.98 (m, 1H), 1.92 – 1.83 (m, 1H), 1.82 – 1.73 (m, 1H), 1.68 – 1.54 (m, 2H), 1.50 (tdd, *J* = 12.6, 9.7, 3.3 Hz, 1H). **^{13}C NMR** (151 MHz, $CDCl_3$) *δ* 142.3, 140.2, 136.1, 133.8, 132.6, 131.7, 131.2, 129.2, 128.6, 128.4, 55.7, 28.9, 24.4, 22.9, 21.5, 21.1. **IR (neat)**: *v* = 3065, 3022, 2936, 2869, 2322, 2189, 2031, 1912, 1596, 1560, 1484, 1446, 1400, 1309, 1218, 1146, 931, 700, 585 cm^{-1}. **HRMS(ESI)**: calculated for $C_{21}H_{25}O_2NNaS$ $[M+Na]^+$: 378.1606, found: 378.1499.

3.2 General procedure of *N*-arylsulfonyl acrylamides 1an.

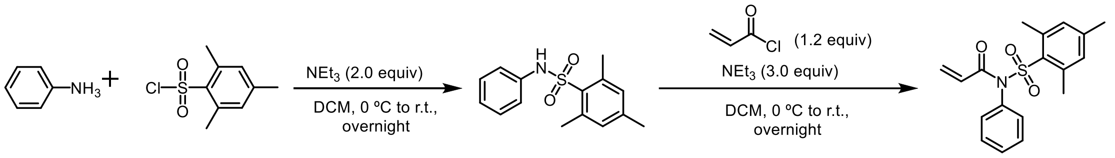

1.0 eq 1.1 eq **1an**

A solution of the aniline (1.0 equiv, 10.0 mmol) in DCM (20 mL) was cooled to 0 °C, triethylamine (2.0 equiv, 20.0 mmol) was then added dropwise, followed by 2,4,6-trimethylbenzenesulfonyl chloride (1.1 equiv, 11.0 mmol). The reaction mixture was warmed to room temperature and stirred overnight, then diluted with water (20.0 mL) and extracted with DCM (3 x 30 mL). The organic extracts were dried with sodium sulfate and concentrated under vacuum. The crude reaction was then purified using flash column chromatography.[1]

A solution of 2,4,6-trimethyl-N-phenylbenzenesulfonamide (1.0 equiv, 5 mmol) in DCM (10 mL) was added triethylamine (3.0 equiv, 15.0 mmol) and acryloyl chloride (1.2 equiv, 15.0 mmol) at 0 °C. The reaction mixture was warmed to room temperature and stirred overnight, then diluted with water (10.0 mL) and extracted with DCM (3 x 15 mL). The organic extracts were dried with sodium sulfate and concentrated under vacuum. The crude reaction was then purified using flash column chromatography. [2]

N-(mesitylsulfonyl)-*N*-phenylacrylamide (1an)

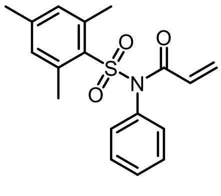

Prepared following reported procedure,[1-2] **1an** was obtained as a white solid in 52% yield (0.86g, 2.60 mmol). **^{1}H NMR** (600 MHz, $CDCl_3$) δ 7.53 – 7.46 (m, 3H), 7.45 – 7.36 (m, 2H), 6.98 (s, 2H), 6.38 (dd, *J* = 16.8, 1.5 Hz, 1H), 5.99 (dd, *J* = 16.8, 10.4 Hz, 1H), 5.61 (dd, *J* = 10.5, 1.5 Hz, 1H), 2.70 (s, 6H), 2.31 (s, 3H). **^{13}C NMR** (151 MHz, $CDCl_3$) δ 165.8, 143.7, 141.0, 135.0, 134.0, 132.1, 131.4, 130.8, 130.0, 129.8, 128.5, 23.1, 21.2. **IR (neat)**: *v* = 3254, 2980, 2932, 2544, 2166, 1967, 1897, 1684, 1598, 1482, 1455, 1402, 1295, 1185, 1034, 974, 927, 852, 695, 654 cm^{-1}. **HRMS(ESI)**: calculated for $C_{18}H_{20}O_3NS$ $[M+H]^+$: 330.1086, found: 330.1160.

3.3 General procedure of drug molecules 1aj and 1ak.

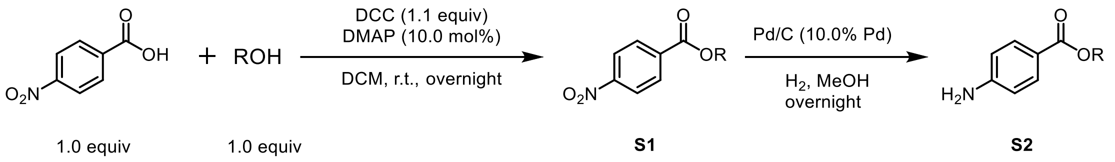

A mixture of the respective alcohol (1.0 equiv), 4-Nitrobenzoic acid (1.0 equiv), *N*,*N*′-Dicyclohexylcarbodiimide (1.1 equiv) and 4-Dimethylaminopyridine (10.0 mol%) in DCM (0.5 M) was stirred overnight under the room temperature. After the reaction was complete, the reaction mixture was filtered and concentrated under vacuum to obtain the crude intermediate **S1** was directly used in the next step without further purification.

To the menthol (0.5 M) solution of intermediate S1, 10% palladium on active carbon was added under argon atmosphere. Hydrogen gas was bubbled into the suspension with stirring at room temperature overnight. After the palladium on carbon was removed by filtration, the solvent was removed under vacuum, to afford the corresponding **S2**.[3]

Corresponding drug molecules analogues (**1aj**, **1ak**) were synthesized by General procedure of Sulfonamide from corresponding **S2**.[1-2]

(1*R*,2*S*,5*R*)-2-isopropyl-5-methylcyclohexyl4-((*N*-allyl-2,4,6-trimethylphenyl) sulfonamido) benzoate (1aj)

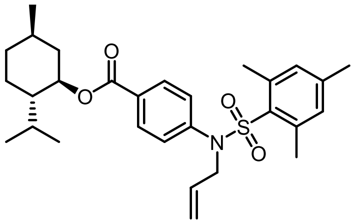

Prepared following general procedure of drug molecules. **1aj** was obtained as a yellow solid in 20% yield (0.50g, 1.00 mmol). **^{1}H NMR** (600 MHz, $CDCl_3$) δ 7.90 – 7.82 (m, 2H), 7.19 – 7.10 (m, 2H), 6.82 (s, 2H), 5.69 (ddt, *J* = 16.7, 10.2, 6.4 Hz, 1H), 5.05 – 4.95 (m, 2H), 4.82 (td, *J* = 10.9, 4.4 Hz, 1H), 4.23 (d, *J* = 6.4 Hz, 2H), 2.40 (s, 6H), 2.21 (s, 3H), 2.06 – 1.98 (m, 1H), 1.91 – 1.77 (m, 1H), 1.65 (dt, *J* = 11.7, 2.9 Hz, 2H), 1.54 – 1.39 (m, 2H), 1.10 – 0.94 (m, 2H), 0.84 (t, *J* = 6.1 Hz, 7H), 0.70 (d, *J* = 6.9 Hz, 3H). **^{13}C NMR** (151 MHz, $CDCl_3$) δ 165.5, 143.5, 143.0, 140.4, 132.1, 130.4, 129.7, 128.2, 119.4, 75.2, 53.0, 47.4, 41.1, 34.4, 31.6, 26.6, 23.1, 22.2, 20.9, 16.6.

IR (neat): *v* = 3401, 3072, 2950, 2867, 2322, 2198, 2077, 1988, 1951, 1705, 1643, 1603, 1564, 1506, 1456, 1413, 1340, 1267, 1158, 1112, 1072, 1019, 983, 960, 932, 878, 847, 778, 739, 702, 658 cm^{-1}. **HRMS(ESI)**: calculated for $C_{29}H_{39}O_4NNaS$ $[M+Na]^+$: 520.2492, found: 520.2476.

(*R*)-2,8-dimethyl-2-((4*R*,8*R*)-4,8,12-trimethyltridecyl)chroman-6-yl 4-((*N*-allyl-2,4,6-trimethylphenyl)sulfonamido)-2-methylbenzoate (1ak)

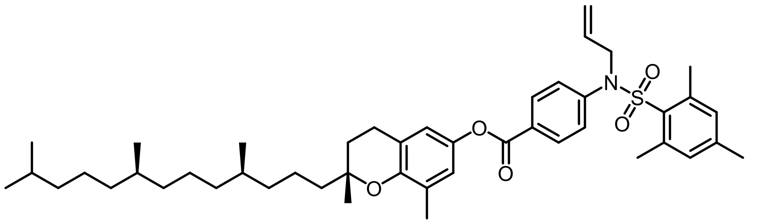

Prepared following general procedure of drug molecules. **1ak** was obtained as a yellow solid in 11% yield (0.42g, 0.55 mmol). **^{1}H NMR** (600 MHz, $CDCl_3$) δ 8.07 (d, *J* = 8.6 Hz, 2H), 7.27 (d, *J* = 3.6 Hz, 1H), 6.91 (s, 2H), 6.77 (d, *J* = 2.8 Hz, 1H), 6.72 (d, *J* = 2.8 Hz, 1H), 5.87 – 5.70 (m, 1H), 5.19 – 5.10 (m, 2H), 4.36 (d, *J* = 6.4 Hz, 2H), 2.75 (q, *J* = 6.8 Hz, 2H), 2.49 (s, 6H), 2.30 (s, 2H), 2.17 (s, 2H), 1.83 (dt, *J* = 13.9, 7.0 Hz, 1H), 1.75 (dt, *J* = 13.3, 6.4 Hz, 1H), 1.64 – 1.50 (m, 4H), 1.44 – 1.35 (m, 3H), 1.34 – 1.22 (m, 12H), 1.18 – 1.11 (m, 3H), 1.11 – 1.03 (m, 4H), 0.91 – 0.78 (m, 13H). **^{13}C NMR** (151 MHz, $CDCl_3$) δ 165.2, 150.1, 144.1, 143.1, 142.7, 140.4, 132.9, 132.7, 132.2, 132.0, 131.0, 128.7, 128.3, 127.6, 121.2, 121.2, 119.5, 119.2, 76.4, 53.0, 40.3, 39.5, 37.6, 37.6, 37.4, 33.0, 32.9, 31.1, 28.1, 25.0, 24.6, 24.4, 23.1, 22.9, 22.8, 22.6, 21.1, 19.9, 19.8, 16.3, 14.4, 13.1. **IR (neat)**: *v* = 2925, 2864, 2322, 2077, 1737, 1647, 1603, 1505, 1465, 1377, 1318, 1262, 1224, 1150, 1072, 1016, 925, 893, 865, 773, 745, 696 cm^{-1}. **HRMS(ESI)**: calculated for $C_{47}H_{67}O_5NNaS$ $[M+Na]^+$: 766.4632, found: 766.4607.

4. General procedure and characterization data of Smile rearrangement products

4.1 General procedure 1 of Smile rearrangement of Sulfonamide

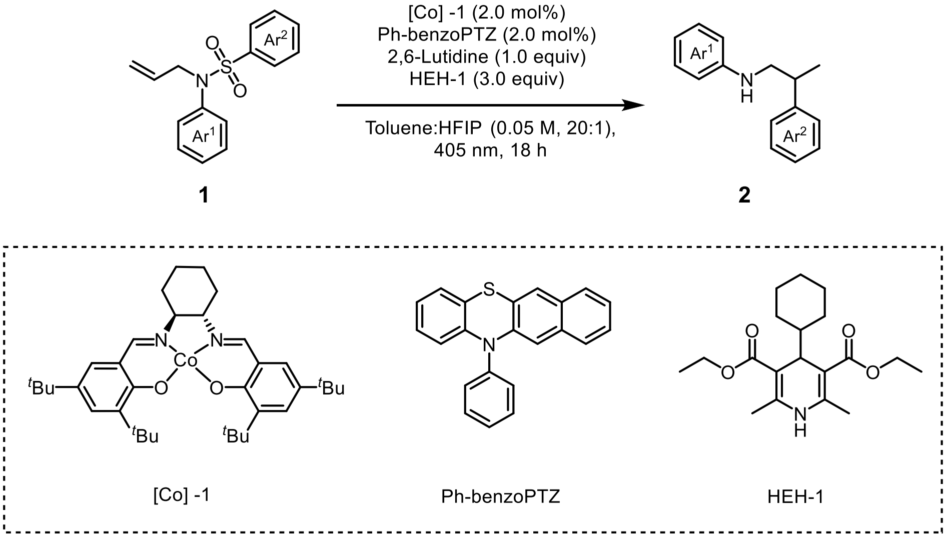

In an oven-dried 4 mL vial equipped with a magnetic stirring bar were added the sulfonamide **1** (0.10 mmol, 1.0 equiv), [Co] -1 (1.2 mg, 2.0 µmol, 2.0 mol%), Ph-benzoPTZ (0.7 mg, 2.0 µmol, 2.0 mol%) and HEH-1 (100.6 mg, 0.3 mmol, 3.0 equiv). A plastic cap with rubber septum was used to close the vial and the system was purged with an argon balloon for 15 minutes. Toluene (2 mL, 0.05 M) was added followed by HFIP (0.1 mL) and 2,6-Lutidine (11.6µL, 0.1mmol, 1.0 equiv). The vial was then placed in the PhotoRedOx Box (see Materials and Methods for more details about the photochemical setup) and irradiated for 18 hours. The crude was then concentrated under reduced pressure and purified by flash column chromatography on silica gel with Pentane-Et_2O.

***N*-(2-phenylpropyl)aniline (2a)**

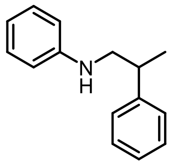

Prepared following general procedure 1, **2a** was obtained after purification by column chromatography (pentane: Et_2O 200:1) as a pale-yellow oil (5.9 mg, 28 µmol, 28%). Rf = 0.60 (pentane: EtOAc 20:1). **^{1}H NMR** (600 MHz, $CDCl_3$) δ 7.28 – 7.22 (m, 2H), 7.20 – 7.13 (m, 3H), 7.11 – 7.05 (m, 2H), 6.61 (t, *J* = 7.3 Hz, 1H), 6.50 (d, *J* = 8.6 Hz, 2H), 3.52 (s, 1H), 3.27 (dd, *J* = 12.4, 6.2 Hz, 1H), 3.16 (dd, *J* = 12.4, 8.3 Hz, 1H), 2.98 (h, *J* = 7.0 Hz, 1H), 1.26 (d, *J* = 7.0 Hz, 3H). **^{13}C NMR** (151 MHz, $CDCl_3$) δ 148.2, 144.7, 129.4, 128.8, 127.4, 126.8, 117.5, 113.1, 51.1, 39.4, 19.9. **IR (neat):** *v* = 3412, 3053, 2960, 2924, 2870, 2324, 2115, 1992, 1915, 1817, 1727, 1600, 1502, 1454, 1377, 1318, 1256, 1178, 1154, 1124, 1069, 1017, 910, 869, 748, 694 cm^{-1}. **HRMS(ESI)**: calculated for $C_{15}H_{18}N$ $[M+H]^+$: 212.1434, found: 212.1434.

***N*-(2-mesitylpropyl)aniline (2b)**

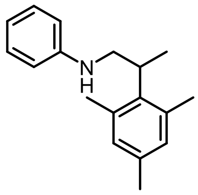

Prepared following general procedure 1, **2b** was obtained after purification by column chromatography (pentane: Et_2O 200:1) as a pale-yellow oil (18.2 mg, 72 µmol, 72%). Rf = 0.60 (pentane: EtOAc 20:1). **^{1}H NMR** (400 MHz, $CDCl_3$) δ 7.22 – 7.15 (m, 2H), 6.83 (s, 2H), 6.76 (tt, *J* = 7.4, 1.1 Hz, 1H), 6.68 (d, *J* = 7.5 Hz, 2H), 3.58 (p, *J* = 7.2 Hz, 1H), 3.53 – 3.41 (m, 2H), 2.50 – 2.19 (m, 9H), 1.39 (d, *J* = 7.1 Hz, 3H). **^{13}C NMR** (101 MHz, $CDCl_3$) δ 136.9, 135.8, 129.4, 118.7, 114.2, 49.4, 34.4, 21.5, 20.8, 17.4. **IR (neat):** *v* = 3409, 29660, 2921, 2867, 2313, 2130, 2062, 1916, 1720, 1656, 1600, 1500, 1374, 1316, 1027, 854, 746, 690, 584 cm^{-1}. **HRMS(ESI)**: calculated for $C_{18}H_{24}N$ $[M+H]^+$: 254.1831, found: 254.1905.

***N*-(2-(*o*-tolyl)propyl)aniline (2c)**

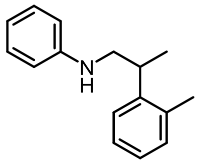

Prepared following general procedure 1, **2c** was obtained after purification by column chromatography (pentane: Et_2O 200:1) as a pale-yellow oil (13.7 mg, 61 µmol, 61%). Rf = 0.60 (pentane: EtOAc 20:1). **^{1}H NMR** (600 MHz, $CDCl_3$) δ 7.26 – 7.20 (m, 2H), 7.20 – 7.12 (m, 4H), 6.70 (t, *J* = 7.3 Hz, 1H), 6.59 (d, *J* = 7.3 Hz, 2H), 3.59 (s, 1H), 3.46 – 3.26 (m, 3H), 2.32 (s, 3H), 1.31 (d, *J* = 6.4 Hz, 3H). **^{13}C NMR** (151 MHz, $CDCl_3$) δ 148.4, 142.8, 136.3, 130.7, 129.4, 126.6, 126.3, 125.5, 117.5, 113.1, 50.3, 34.3, 19.7, 19.7. **IR (neat):** *v* = 3845, 3412, 3051, 3020, 2961, 2925,

2869, 2324, 2095, 1911, 1830, 1601, 1502, 1460, 1432, 1377, 1318, 1256, 1178, 1069, 1015, 990, 868, 750, 691 cm^{-1}. **HRMS(ESI)**: calculated for $C_{16}H_{20}N$ $[M+H]^+$: 226.1590, found: 226.1595.

N-(2-(2-methoxyphenyl)propyl)aniline (2d)

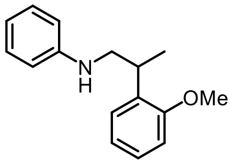

Prepared following general procedure 1, **2d** was obtained after purification by column chromatography (pentane: Et_2O 50:1) as a pale-yellow oil (19.3 mg, 80 µmol, 80%). Rf = 0.35 (pentane: EtOAc 20:1). **^{1}H NMR** (600 MHz, $CDCl_3$) δ 7.25 – 7.21 (m, 2H), 7.19 – 7.14 (m, 2H), 6.97 (td, *J* = 7.5, 1.2 Hz, 1H), 6.91 (dd, *J* = 8.6, 1.2 Hz, 1H), 6.68 (tt, *J* = 7.3, 1.1 Hz, 1H), 6.62 (dd, *J* = 8.7, 1.1 Hz, 2H), 3.85 (s, 3H), 3.77 (s, 1H), 3.57 (h, *J* = 7.0 Hz, 1H), 3.35 (dd, *J* = 12.0, 7.4 Hz, 1H), 3.23 (dd, *J* = 12.0, 6.8 Hz, 1H), 1.34 (d, *J* = 7.0 Hz, 3H). **^{13}C NMR** (151 MHz, $CDCl_3$) δ 157.4, 148.6, 132.8, 129.3, 127.5, 127.1, 120.9, 117.0, 112.9, 110.7, 55.5, 50.2, 32.2, 18.4. **IR (neat):** v = 3414, 3051, 2960, 2836, 2329, 2112, 1994, 1923, 1600, 1497, 1461, 1319, 1238, 1177, 1134, 1025, 929, 869, 800, 747, 691 cm^{-1}. **HRMS(ESI)**: calculated for $C_{16}H_{20}ON$ $[M+H]^+$: 242.1539, found: 242.1543.

N-(2-(2-chlorophenyl)propyl)aniline (2e)

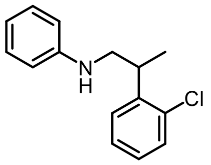

Prepared following general procedure 1, **2e** was obtained after purification by column chromatography (pentane: Et_2O 200:1) as a pale-yellow oil (15.9 mg, 65 µmol, 65%). Rf = 0.60 (pentane: EtOAc 20:1). **^{1}H NMR** (600 MHz, $CDCl_3$) δ 7.31 (dd, *J* = 7.9, 1.3 Hz, 1H), 7.21 (dd, *J* = 7.8, 1.8 Hz, 1H), 7.17 (ddd, *J* = 7.7, 6.9, 1.4 Hz, 1H), 7.12 – 7.05 (m, 3H), 6.61 (tt, *J* = 7.3, 1.1 Hz, 1H), 6.53 (dt, *J* = 7.7, 1.1 Hz, 2H), 3.62 – 3.52 (m, 2H), 3.31 (dd, *J* = 12.3, 7.3 Hz, 1H), 3.19 (dd, *J* = 12.3, 7.0 Hz, 1H), 1.26 (d, *J* = 6.9 Hz, 3H). **^{13}C NMR** (151 MHz, $CDCl_3$) δ 148.2, 141.9, 134.3, 129.9, 129.4, 127.8, 127.5, 127.4, 117.5, 113.0, 49.9, 35.4, 18.9. **IR (neat):** v = 3414, 3054, 2965, 2925, 2869, 2325, 2082, 1994, 1916, 1827, 1682, 1601, 1504, 1474, 1435, 1377, 1318, 1256, 1180, 1118, 1069, 1034, 943, 868, 748, 689 cm^{-1}. **HRMS(ESI)**: calculated for $C_{15}H_{17}NCl$ $[M+H]^+$: 246.1044, found: 246.1049.

N-(2-(2-bromophenyl)propyl)aniline (2f)

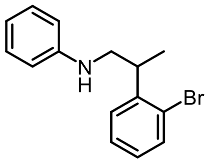

Prepared following general procedure 1, **2f** was obtained after purification by column chromatography (pentane: Et_2O 200:1) as a pale-yellow oil (23.1 mg, 80 µmol, 80%). Rf = 0.55 (pentane: EtOAc 20:1). **^{1}H NMR** (600 MHz, $CDCl_3$) δ 7.59 (dd, *J* = 8.0, 1.3 Hz, 1H), 7.37 – 7.27 (m, 2H), 7.18 (dd, *J* = 8.5, 7.2 Hz, 2H), 7.10 (ddd, *J* = 8.0, 6.9, 2.1 Hz, 1H), 6.71 (tt, *J* = 7.3, 1.1 Hz, 1H), 6.63 (dd, *J* = 8.6, 1.1 Hz, 2H), 3.69 – 3.58 (m, 2H), 3.39 (dd, *J* = 12.3, 7.4 Hz, 1H), 3.28 (dd, *J* = 12.3, 6.9 Hz, 1H), 1.34 (d, *J* = 6.9 Hz, 3H). **^{13}C NMR** (151 MHz, $CDCl_3$) δ 148.2, 143.6, 133.2, 129.3, 128.1, 128.0, 127.5, 125.2, 117.5, 113.0, 50.0, 38.1, 19.1. **IR (neat):** v = 3412, 3053, 2965, 2925, 2869, 2573, 1918, 1826, 1710, 1601, 1504, 1470, 1433, 1377, 1318, 1256, 1179, 1143, 1115, 1070, 1019, 943, 868, 748, 691, 661 cm^{-1}. **HRMS(ESI)**: calculated for $C_{15}H_{17}NBr$ $[M+H]^+$: 290.0539, found: 290.0549.

N-(2-(2-(trifluoromethyl)phenyl)propyl)aniline (2g)

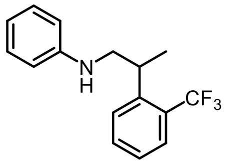

Prepared following general procedure 1, **2g** was obtained after purification by column chromatography (pentane: Et_2O 200:1) as a pale-yellow oil (18.7 mg, 67 µmol, 67%). Rf = 0.57 (pentane: EtOAc 20:1). **^{1}H NMR** (600 MHz, $CDCl_3$) *δ* 7.67 (dd, *J* = 8.0, 1.4 Hz, 1H), 7.57 – 7.52 (m, 1H), 7.49 (d, *J* = 7.9 Hz, 1H), 7.33 (t, *J* = 7.6 Hz, 1H), 7.20 – 7.12 (m, 2H), 6.70 (tt, *J* = 7.4, 1.2 Hz, 1H), 6.62 – 6.49 (m, 2H), 3.60 (s, 1H), 3.53 (h, *J* = 6.9 Hz, 1H), 3.41 (dd, *J* = 12.1, 7.7 Hz, 1H), 3.34 (dd, *J* = 12.1, 6.9 Hz, 1H), 1.36 (d, *J* = 6.8 Hz, 3H). **^{13}C NMR** (151 MHz, $CDCl_3$) *δ* 148.1, 144.1, 132.4, 129.3, 128.8 (q, *J* = 28.7 Hz), 127.6, 126.5, 126.0 (q, *J* = 5.9 Hz), 124.7 (q, *J* = 273.3 Hz), 117.5, 112.9, 50.6, 34.8, 20.8. **^{19}F NMR** (564 MHz, $CDCl_3$) *δ* -58.4. **IR (neat):** *v* = 3419, 3052, 2969, 2927, 2873, 2326, 2105, 1915, 1830, 1602, 1505, 1456, 1379, 1310, 1260, 1160, 1113, 1035, 956, 870, 750, 691 cm^{-1}. **HRMS(ESI)**: calculated for $C_{16}H_{17}NF_3$ $[M+H]^+$:280.1308, found: 280.1308.

Methyl 2-(1-(phenylamino)propan-2-yl)benzoate (2h)

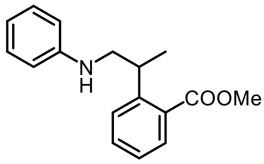

Prepared following general procedure 1, **2h** was obtained after purification by column chromatography (pentane: Et_2O 50:1) as a pale-yellow oil (11.6 mg, 43 µmol, 43%). Rf = 0.28 (pentane: EtOAc 20:1). **^{1}H NMR** (600 MHz, $CDCl_3$) *δ* 7.66 (dd, *J* = 7.9, 1.5 Hz, 1H), 7.41 (td, *J* = 7.6, 1.5 Hz, 1H), 7.35 (dd, *J* = 8.0, 1.3 Hz, 1H), 7.22 – 7.14 (m, 1H), 7.06 (dd, *J* = 8.6, 7.2 Hz, 2H), 6.57 (t, *J* = 7.3 Hz, 1H), 6.47 (d, *J* = 7.9 Hz, 2H), 3.96 (s, 1H), 3.89 – 3.79 (m, 1H), 3.76 (s, 3H), 3.41 – 3.06 (m, 2H), 1.27 (d, *J* = 6.9 Hz, 3H). **^{13}C NMR** (151 MHz, $CDCl_3$) *δ* 169.1, 148.5, 145.8, 132.1, 131.3, 130.0, 129.3, 126.8, 126.3, 117.0, 112.7, 52.4, 51.5, 34.5, 20.1. **IR (neat):** *v* = 3387, 3023, 2954, 2871, 2328, 2097, 1919, 1715, 1601, 1506, 1434, 1378, 1255, 1192, 1122, 1071, 1017, 958, 869, 826, 750, 718, 692,665 cm^{-1}. **HRMS(ESI)**: calculated for $C_{17}H_{19}O_2NNa$ $[M+Na]^+$:292.1308, found: 292.1305.

N-(2-(2,6-dimethoxyphenyl)propyl)aniline (2i)

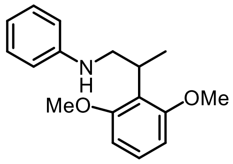

Prepared following general procedure 1, **2i** was obtained after purification by column chromatography (pentane: Et_2O 50:1) as a pale-yellow oil (21.2 mg, 78 µmol, 78%). Rf = 0.23 (pentane: EtOAc 20:1). **^{1}H NMR** (600 MHz, $CDCl_3$) *δ* 7.15 (q, *J* = 8.4 Hz, 3H), 6.70 – 6.62 (m, 1H), 6.57 (t, *J* = 8.3 Hz, 4H), 3.82 (q, *J* = 7.2 Hz, 1H), 3.79 (s, 6H), 3.48 (dd, *J* = 11.8, 8.0 Hz, 1H), 3.41 (dd, *J* = 11.9, 6.9 Hz, 1H), 1.36 (d, *J* = 7.0 Hz, 3H). **^{13}C NMR** (151 MHz, $CDCl_3$) *δ* 159.2, 148.9, 129.1, 127.5, 120.4, 116.7, 112.9, 104.5, 55.8, 48.3, 29.5, 16.9. **IR (neat):** *v* = 3410, 2932, 2837, 2333, 1593, 1504, 1464, 1366, 1319, 1246, 1186, 1111, 1026, 781, 738 cm^{-1}. **HRMS(ESI)**: calculated for $C_{17}H_{22}O_2N$ $[M+H]^+$:272.1572, found: 272.1647.

N-(2-(2,6-dichlorophenyl)propyl)aniline (2j)

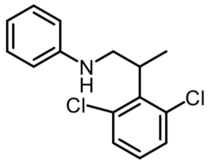

Prepared following general procedure 1, **2j** was obtained after purification by column chromatography (pentane: Et_2O 200:1) as a pale-yellow oil (16.7 mg, 60 µmol, 60%). Rf = 0.54 (pentane: EtOAc 20:1). **^{1}H NMR** (300 MHz, $CDCl_3$) δ 7.28 – 7.14 (m, 2H), 7.14 – 7.05 (m, 2H), 7.03 – 6.93 (m, 1H), 6.73 – 6.53 (m, 3H), 4.00 (h, J = 7.3 Hz, 1H), 3.67 (dd, J = 12.9, 7.5 Hz, 1H), 3.53 (dd, J = 12.9, 7.5 Hz, 1H), 1.41 (dd, J = 7.2, 0.7 Hz, 3H). **^{13}C NMR** (75 MHz, $CDCl_3$) δ 138.7, 130.4, 129.4, 128.8, 128.2, 118.5, 114.0, 47.9, 36.4, 16.0. **IR (neat):** v = 3410, 2961, 2924, 2862, 2323, 1921, 1598, 1503, 1430, 1373, 1318, 1257, 1184, 1075, 870, 744, 690, 554 cm^{-1}. **HRMS(ESI)**: calculated for $C_{15}H_{16}Cl_2N$ $[M+H]^+$:280.0582, found: 280.0658.

N-(2-(4-(trifluoromethyl)phenyl)propyl)aniline (2k)

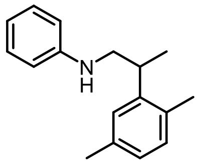

Prepared following general procedure 1, **2k** was obtained after purification by column chromatography (pentane: Et_2O 200:1) as a pale-yellow oil (14.6 mg, 61 µmol, 61%). Rf = 0.55 (pentane: EtOAc 20:1). **^{1}H NMR** (600 MHz, $CDCl_3$) δ 7.22 – 7.14 (m, 2H), 7.08 (d, J = 7.7 Hz, 1H), 7.05 (s, 1H), 6.97 (d, J = 7.7 Hz, 1H), 6.71 (t, J = 7.3 Hz, 1H), 6.68 – 6.50 (m, 2H), 3.63 (s, 1H), 3.49 – 3.21 (m, 3H), 2.34 (s, 3H), 2.28 (s, 3H), 1.30 (d, J = 5.6 Hz, 3H). **^{13}C NMR** (151 MHz, $CDCl_3$) δ 148.4, 142.6, 136.0, 133.1, 130.6, 129.4, 127.1, 126.2, 117.4, 113.1, 50.4, 34.3, 21.3, 19.8, 19.2. **IR (neat):** v = 3412, 3017, 2961, 2923, 2868, 2324, 2099, 1998, 1905, 1826, 1759, 1601, 1502, 1458, 1376, 1318, 1257, 1177, 1154, 1116, 1068, 1037, 985, 870, 809, 747, 691 cm^{-1}. **HRMS(ESI)**: calculated for $C_{17}H_{22}N$ $[M+H]^+$:240.1747, found: 240.1738.

N-(2-(2,4-dimethylphenyl)propyl)aniline (2l)

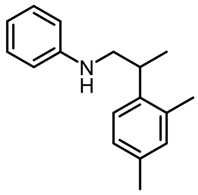

Prepared following general procedure 1, **2l** was obtained after purification by column chromatography (pentane: Et_2O 200:1) as a pale-yellow oil (13.4 mg, 56 µmol, 56%). Rf = 0.55 (pentane: EtOAc 20:1). **^{1}H NMR** (600 MHz, $CDCl_3$) δ 7.23 – 7.15 (m, 2H), 7.13 (d, J = 7.8 Hz, 1H), 7.09 – 6.97 (m, 2H), 6.70 (tt, J = 7.3, 1.1 Hz, 1H), 6.63 – 6.53 (m, 2H), 3.60 (s, 1H), 3.39 – 3.24 (m, 3H), 2.32 (s, 3H), 2.28 (s, 3H), 1.29 (d, J = 6.0 Hz, 3H). **^{13}C NMR** (151 MHz, $CDCl_3$) δ 148.4, 139.7, 136.2, 135.8, 131.5, 129.4, 127.3, 125.4, 117.4, 113.1, 50.4, 34.0, 21.0, 19.9, 19.6. **IR (neat):** v = 3411, 3015, 2960, 2923, 2868, 2325, 2114, 1994, 1910, 1729, 1601, 1502, 1456, 1376, 1318, 1256, 1177, 1118, 1067, 1022, 991, 931, 870, 817, 747, 691 cm^{-1}. **HRMS(ESI)**: calculated for $C_{17}H_{22}N$ $[M+H]^+$:240.1747, found: 240.1747.

***N*-(2-(2,3,4,5,6-pentamethylphenyl)propyl)aniline (2m)**

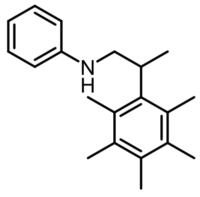

Prepared following general procedure 1, **2m** was obtained after purification by column chromatography (pentane: Et_2O 200:1) as a pale-yellow oil (20.0 mg, 71 µmol, 71%). Rf = 0.55 (pentane: EtOAc 20:1). **^{1}H NMR** (600 MHz, $CDCl_3$) δ 7.18 (dd, *J* = 8.6, 7.3 Hz, 2H), 6.71 (t, *J* = 7.3 Hz, 1H), 6.65 – 6.51 (m, 2H), 3.73 (h, *J* = 7.4 Hz, 1H), 3.66 (s, 1H), 3.59 – 3.45 (m, 2H), 2.48 – 2.11 (m, 15H), 1.45 (d, *J* = 7.3 Hz, 3H). **^{13}C NMR** (151 MHz, $CDCl_3$) δ 148.4, 137.8, 133.4, 129.3, 117.3, 113.0, 49.2, 35.4, 18.0, 17.2. **IR (neat):** *v* = 3407, 2922, 2327, 2113, 1993, 1909, 1601, 1503, 1379, 1317, 1257, 1178, 1152, 1118, 1062, 990, 867, 817, 746, 691 cm^{-1}. **HRMS(ESI)**: calculated for $C_{20}H_{28}N$ $[M+H]^+$:282.2216, found: 282.2212.

***N*-(2-(2,4,6-triisopropylphenyl)propyl)aniline (2n)**

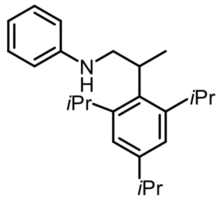

Prepared following general procedure 1, **2n** was obtained after purification by column chromatography (pentane: Et_2O 200:1) as a white solid (17.2 mg, 51 µmol, 51%). Rf = 0.50 (pentane: EtOAc 20:1). **^{1}H NMR** (600 MHz, $CDCl_3$) δ 7.21 – 7.13 (m, 2H), 7.02 (dd, *J* = 18.8, 2.1 Hz, 2H), 6.69 (tt, *J* = 7.3, 1.1 Hz, 1H), 6.65 – 6.49 (m, 2H), 3.70 (h, *J* = 7.4 Hz, 1H), 3.62 (s, 1H), 3.54 (dd, *J* = 12.2, 7.7 Hz, 1H), 3.41 (dd, *J* = 12.2, 7.6 Hz, 1H), 3.37 (q, *J* = 6.8 Hz, 1H), 3.23 (p, *J* = 6.8 Hz, 1H), 2.88 (p, *J* = 6.9 Hz, 1H), 1.46 (d, *J* = 7.3 Hz, 3H), 1.28 (d, *J* = 6.9 Hz, 9H), 1.24 (t, *J* = 6.8 Hz, 6H), 1.19 (d, *J* = 6.8 Hz, 3H). **^{13}C NMR** (151 MHz, $CDCl_3$) δ 148.3, 148.2, 147.1, 146.7, 135.1, 129.4, 123.2, 121.3, 117.4, 113.0, 49.8, 34.0, 33.0, 29.9, 29.8, 25.1, 25.0, 24.9, 24.1, 24.1, 24.1, 19.2. **IR (neat):** *v* = 3339, 3050, 2958, 2871, 2325, 2201, 2076, 1993, 1916, 1827, 1768, 1599, 1502, 1459, 1379, 1310, 1254, 1177, 1100, 1061, 1011, 930, 784, 745, 691 cm^{-1}. **HRMS(ESI)**: calculated for $C_{24}H_{36}N$ $[M+H]^+$:338.2842, found: 338.2849.

***N*-(2-(4-methoxyphenyl)propyl)aniline (2o)**

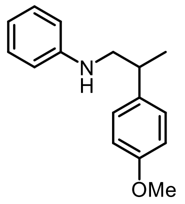

Prepared following general procedure 1, **2o** was obtained after purification by column chromatography (pentane: Et_2O 50:1) as a pale-yellow oil (6.0 mg, 25 µmol, 25%). Rf = 0.24 (pentane: EtOAc 20:1). **^{1}H NMR** (600 MHz, $CDCl_3$) δ 7.16 (dd, *J* = 8.5, 7.5 Hz, 4H), 6.90 – 6.83 (m, 2H), 6.69 (tt, *J* = 7.3, 1.1 Hz, 1H), 6.57 (dd, *J* = 8.7, 1.1 Hz, 2H), 3.81 (s, 3H), 3.57 (s, 1H), 3.32 (dd, *J* = 12.3, 6.1 Hz, 1H), 3.18 (dd, *J* = 12.3, 8.4 Hz, 1H), 3.01 (h, *J* = 7.0 Hz, 1H), 1.31 (d, *J* = 7.0 Hz, 3H). **^{13}C NMR** (151 MHz, $CDCl_3$) δ 158.4, 148.3, 136.7, 129.4, 128.3, 117.4, 114.2, 113.1, 55.4, 51.2, 38.5, 20.1. **IR (neat):** *v* = 3408, 3020, 2956, 2925, 2660, 2326, 2110, 1993, 1723, 1602, 1506, 1462, 1376, 1315, 1245, 1178, 1118, 1066, 1033, 870, 829, 748, 692 cm^{-1}. **HRMS(ESI)**: calculated for $C_{16}H_{20}ON$ $[M+H]^+$:242.1539, found: 242.1545.

***N*-(2-(4-(trifluoromethyl)phenyl)propyl)aniline (2p)**

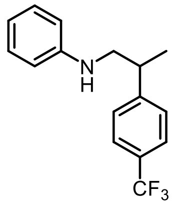

Prepared following general procedure 1, **2p** was obtained after purification by column chromatography (pentane: Et_2O 200:1) as a pale-yellow oil (11.7 mg, 42 µmol, 42%). Rf = 0.61 (pentane: EtOAc 20:1). **^{1}H NMR** (600 MHz, $CDCl_3$) δ 7.59 (d, *J* = 8.0 Hz, 2H), 7.34 (d, *J* = 8.0 Hz, 2H), 7.24 – 7.11 (m, 2H), 6.71 (tt, *J* = 7.4, 1.1 Hz, 1H), 6.58 (dt, *J* = 7.7, 1.1 Hz, 2H), 3.56 (s, 1H), 3.38 (dd, *J* = 12.8, 6.1 Hz, 1H), 3.27 (dd, *J* = 12.7, 8.2 Hz, 1H), 3.21 – 3.10 (m, 1H), 1.36 (d, *J* = 7.0

Hz, 3H). **^{13}C NMR** (151 MHz, $CDCl_3$) δ 148.9, 147.9, 129.5, 129.1 (q, J = 32.5 Hz), 127.8, 125.7 (q, J = 3.8 Hz), 124.4 (d, J = 271.6 Hz), 117.7, 113.1, 50.9, 39.3, 19.6. **^{19}F NMR** (564 MHz, $CDCl_3$) δ -62.41. **IR (neat):** v = 3415, 3052, 2964, 2929, 2874, 2310, 2069, 1920, 1724, 1602, 1505, 1422, 1379, 1322, 1257, 1162, 1117, 1067, 1015, 870, 838, 748, 692 cm^{-1}. **HRMS(ESI)**: calculated for $C_{16}H_{17}NF_3$ $[M+H]^+$:280.1308, found: 280.1312.

***N*-(2-(4-(trifluoromethyl)phenyl)propyl)aniline (2q)**

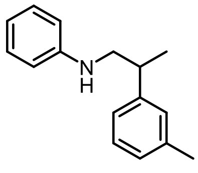

Prepared following general procedure 1, **2q** was obtained after purification by column chromatography (pentane: Et_2O 200:1) as a pale-yellow oil (6.8 mg, 30 µmol, 30%). Rf = 0.59 (pentane: EtOAc 20:1). **^{1}H NMR** (600 MHz, $CDCl_3$) δ 7.23 (t, J = 7.5 Hz, 1H), 7.19 – 7.13 (m, 2H), 7.11 – 7.00 (m, 3H), 6.69 (tt, J = 7.3, 1.1 Hz, 1H), 6.58 (dt, J = 7.6, 1.1 Hz, 2H), 3.58 (s, 1H), 3.33 (dd, J = 12.5, 6.1 Hz, 1H), 3.23 (dd, J = 12.3, 8.3 Hz, 1H), 3.02 (dt, J = 8.2, 6.7 Hz, 1H), 2.36 (s, 3H), 1.33 (d, J = 7.0 Hz, 3H). **^{13}C NMR** (151 MHz, $CDCl_3$) δ 148.3, 144.6, 138.4, 129.4, 128.7, 128.1, 127.5, 124.4, 117.4, 113.1, 51.0, 39.3, 21.6, 20.0. **IR (neat):** v = 3407, 3020, 2957, 2919, 2866, 2386, 1599, 1374, 1316, 1257, 1171, 1117, 909, 870, 785, 745, 566 cm^{-1}. **HRMS(ESI)**: calculated for $C_{16}H_{20}N$ $[M+H]^+$:226.1518, found: 226.1591.

***N*-(2-(naphthalen-1-yl)propyl)aniline (2r)**

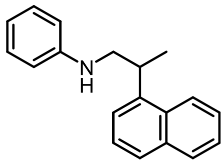

Prepared following general procedure 1, **2r** was obtained after purification by column chromatography (pentane: Et_2O 200:1) as a pale-yellow oil (11.0 mg, 42 µmol, 42%). Rf = 0.46 (pentane: EtOAc 20:1). **^{1}H NMR** (600 MHz, $CDCl_3$) δ 8.19 – 7.99 (m, 1H), 7.90 (dd, J = 6.9, 2.5 Hz, 1H), 7.77 (d, J = 8.0 Hz, 1H), 7.61 – 7.40 (m, 4H), 7.17 (t, J = 7.7 Hz, 2H), 6.71 (t, J = 7.3 Hz, 1H), 6.59 (d, J = 7.9 Hz, 2H), 3.98 (h, J = 6.9 Hz, 1H), 3.75 – 3.63 (m, 1H), 3.54 (dd, J = 12.2, 7.3 Hz, 1H), 3.48 (dd, J = 12.2, 6.5 Hz, 1H), 1.49 (d, J = 6.9 Hz, 3H). **^{13}C NMR** (151 MHz, $CDCl_3$) δ 148.3, 140.6, 134.2, 132.0, 129.4, 129.2, 127.2, 126.2, 125.8, 125.7, 123.1, 123.0, 117.5, 113.0, 50.4, 33.7, 20.2. **IR (neat):** v = 3409, 3048, 2963, 2925, 2869, 2323, 2088, 1993, 1921, 1813, 1727, 1600, 1504, 1430, 1391, 1318, 1255, 1176, 1123, 1066, 1023, 992, 865, 777, 747, 691 cm^{-1}. **HRMS(ESI)**: calculated for $C_{19}H_{20}N$ $[M+H]^+$:262.1590, found: 262.1594.

***N*-(2-(isoquinolin-5-yl)propyl)aniline (2s)**

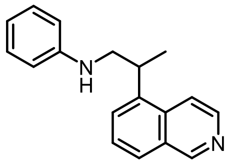

Prepared following general procedure 1, **2s** was obtained after purification by column chromatography (pentane: Et_2O 100:1) as a pale-yellow oil (4.5 mg, 17 µmol, 17%). Rf = 0.34 (pentane: EtOAc 20:1). **^{1}H NMR** (400 MHz, $CDCl_3$) δ 9.05 – 8.89 (m, 1H), 8.20 – 8.10 (m, 1H), 7.69 (d, J = 8.1 Hz, 1H), 7.64 (d, J = 7.2 Hz, 1H), 7.52 (t, J = 7.6 Hz, 1H), 7.41 (dd, J = 8.2, 4.1 Hz, 1H), 7.10 (t, J = 7.6 Hz, 2H), 6.61 (t, J = 7.3 Hz, 1H), 6.55 (d, J = 7.9 Hz, 2H), 4.57 (q, J = 7.1 Hz, 1H), 3.59 – 3.25 (m, 2H), 1.49 (d, J = 7.3 Hz, 3H). **^{13}C NMR** (151 MHz, $CDCl_3$) δ 149.4, 143.6, 136.8, 129.2, 128.6, 126.7, 126.5, 126.5, 121.1, 112.7, 51.6, 32.7, 19.3. **IR (neat):** v = 3400, 3048, 2925, 2856, 2324, 2071, 2007, 1928, 1680, 1600, 1501, 1369, 1318, 1258, 1177,

1135, 1069, 997, 829, 794, 749, 692 cm^{-1}. **HRMS(ESI)**: calculated for $C_{18}H_{19}N_2$ [M+H]$^+$:263.1543, found: 263.1540.

N-(2-mesitylpropyl)-4-methylaniline (2t)

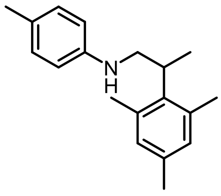

Prepared following general procedure 1, **2t** was obtained after purification by column chromatography (pentane: Et_2O 200:1) as a pale-yellow oil (17.4 mg, 65 µmol, 65%). Rf = 0.61 (pentane: EtOAc 20:1). **^{1}H NMR** (400 MHz, $CDCl_3$) δ 7.06 – 6.92 (m, 2H), 6.85 (s, 2H), 6.72 – 6.36 (m, 2H), 3.68 – 3.50 (m, 1H), 3.51 – 3.38 (m, 2H), 2.50 – 2.16 (m, 12H), 1.37 (dd, *J* = 7.3, 2.3 Hz, 3H). **^{13}C NMR** (101 MHz, $CDCl_3$) δ 146.1, 137.1, 135.7, 129.9, 126.6, 113.2, 48.9, 34.8, 21.6, 20.8, 20.5, 17.4. **IR (neat):** v = 3407, 2961, 2921, 2866, 2327, 2095, 1992, 1863, 1727, 1615, 1518, 1478, 1377, 1313, 1253, 1181, 1126, 1078, 1030, 918, 851, 806, 743, 701 cm^{-1}. **HRMS(ESI)**: calculated for $C_{19}H_{26}N$ [M+H]$^+$:268.2060, found: 268.2058.

N-(2-mesitylpropyl)-4-methoxyaniline (2u)

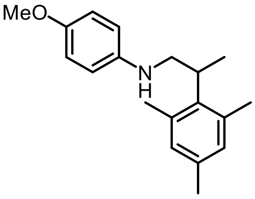

Prepared following general procedure 1, **2u** was obtained after purification by column chromatography (pentane: Et_2O 50:1) as a pale-yellow oil (15.9 mg, 56 µmol, 56%). Rf = 0.34 (pentane: EtOAc 20:1). **^{1}H NMR** (400 MHz, $CDCl_3$) δ 6.84 (s, 2H), 6.81 – 6.72 (m, 2H), 6.64 – 6.47 (m, 2H), 3.74 (s, 3H), 3.63 – 3.49 (m, 1H), 3.48 – 3.35 (m, 2H), 2.43 – 2.15 (m, 9H), 1.36 (dd, *J* = 7.2, 2.4 Hz, 3H). **^{13}C NMR** (101 MHz, $CDCl_3$) δ 152.2, 142.6, 137.1, 135.7, 115.1, 114.4, 56.0, 49.5, 34.8, 21.6, 20.8, 17.4. **IR (neat):** v = 3393, 2926, 2326, 2077, 1993, 1842, 1726, 1611, 1510, 1462, 1378, 1299, 1235, 1177, 1123, 1077, 1036, 851, 818, 726 cm^{-1}. **HRMS(ESI)**: calculated for $C_{19}H_{26}ON$ [M+H]$^+$:284.2009, found: 284.2008.

4-chloro-*N*-(2-mesitylpropyl)aniline (2v)

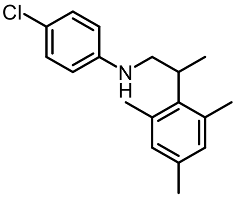

Prepared following general procedure 1, **2v** was obtained after purification by column chromatography (pentane: Et_2O 200:1) as a pale-yellow oil (20.4 mg, 71 µmol, 71%). Rf = 0.57 (pentane: EtOAc 20:1). **^{1}H NMR** (400 MHz, $CDCl_3$) δ 7.14 – 7.03 (m, 2H), 6.85 (s, 2H), 6.65 – 6.32 (m, 2H), 3.61 – 3.50 (m, 1H), 3.44 (d, *J* = 7.9 Hz, 2H), 2.50 – 1.99 (m, 9H), 1.52 – 1.28 (m, 3H). **^{13}C NMR** (101 MHz, $CDCl_3$) δ 146.9, 136.7, 135.9, 129.2, 121.9, 114.0, 48.5, 34.7, 21.5, 20.8, 17.4. **IR (neat):** v = 3409, 2963, 2923, 2871, 2730, 2328, 2162, 1862, 1730, 1600, 1497, 1379, 1316, 1254, 1175, 1088, 1029, 852, 813, 742, 698, 669 cm^{-1}. **HRMS(ESI)**: calculated for $C_{18}H_{22}NCl$ [M+H]$^+$:287.1435, found: 287.1433.

4-bromo-*N*-(2-mesitylpropyl)aniline (2w)

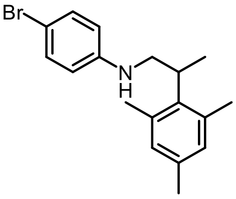

Prepared following general procedure 1, **2w** was obtained after purification by column chromatography (pentane: Et_2O 200:1) as a pale-yellow oil (22.5 mg, 68 µmol, 68%). Rf = 0.54 (pentane: EtOAc 20:1). **^{1}H NMR** (400 MHz, $CDCl_3$) δ 7.25 – 7.20 (m, 2H), 6.85 (s, 2H), 6.57 – 6.30 (m, 2H), 3.55 (h, *J* = 7.3 Hz, 1H), 3.43 (d, *J* = 7.9 Hz, 2H), 2.48 – 2.16 (m,

9H), 1.37 (dd, *J* = 7.2, 1.6 Hz, 3H). **^{13}C NMR** (101 MHz, $CDCl_3$) *δ* 147.3, 136.7, 135.9, 132.0, 114.5, 108.9, 48.4, 34.7, 21.5, 20.8, 17.4. **IR (neat):** *v* = 3408, 2962, 2922, 2870, 2327, 2084, 1997, 1862, 1731, 1594, 1494, 1381, 1316, 1254, 1176, 1125, 1071, 1029, 1003, 920, 852, 810, 742, 695 cm^{-1}. **HRMS(ESI)**: calculated for $C_{18}H_{23}NBr$ $[M+H]^+$:332.1008, found: 332.1009.

4-fluoro-*N*-(2-mesitylpropyl)aniline (2x)

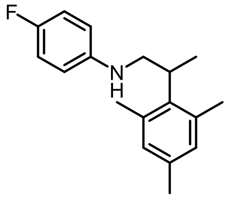

Prepared following general procedure 1, **2w** was obtained after purification by column chromatography (pentane: Et_2O 200:1) as a pale-yellow oil (14.6 mg, 54 µmol, 54%). Rf = 0.64 (pentane: EtOAc 20:1). **^{1}H NMR** (600 MHz, $CDCl_3$) *δ* 6.92 – 6.80 (m, 4H), 6.57 – 6.45 (m, 2H), 3.55 (h, *J* = 7.5 Hz, 1H), 3.49 – 3.37 (m, 2H), 2.63 – 1.98 (m, 9H), 1.37 (d, *J* = 7.2 Hz, 3H). **^{13}C NMR** (151 MHz, $CDCl_3$) *δ* 155.9 (d, *J* = 234.9 Hz), 144.7, 136.8, 135.8, 115.8 (d, *J* = 22.3 Hz), 113.9 (d, *J* = 7.4 Hz). 49.1, 34.7, 21.5, 20.8, 17.4. **^{19}F NMR** (565 MHz, $CDCl_3$) *δ* -128.28. **IR (neat):** *v* = 3406, 2963, 2924, 2871, 2731, 2157, 1847, 1728, 1611, 1509, 1404, 1378, 1316, 1255, 1217, 1156, 1119, 1079, 1029, 915, 852, 818, 775, 738, 695 cm^{-1}. **HRMS(ESI)**: calculated for $C_{18}H_{23}NF$ $[M+H]^+$:272.1809, found: 272.1812.

N-(2-mesitylpropyl)-4-(trifluoromethyl)aniline (2y)

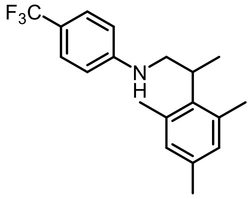

Prepared following general procedure 1, **2y** was obtained after purification by column chromatography (pentane: Et_2O 200:1) as a pale-yellow oil (24.7 mg, 77 µmol, 77%). Rf = 0.60 (pentane: EtOAc 20:1). **^{1}H NMR** (400 MHz, $CDCl_3$) *δ* 7.51 – 7.32 (m, 2H), 6.84 (s, 2H), 6.63 (d, *J* = 8.4 Hz, 2H), 3.62 – 3.53 (m, 1H), 3.53 – 3.48 (m, 2H), 2.43 – 2.24 (m, 9H), 1.39 (d, *J* = 7.0 Hz, 3H). **^{13}C NMR** (101 MHz, $CDCl_3$) *δ* 149.8, 136.3, 136.1, 126.8 (q, *J* = 3.8 Hz), 125.0 (q, *J* = 271.7 Hz), 112.9, 48.6, 34.5, 21.5, 20.8, 17.4. **^{19}F NMR** (377 MHz, $CDCl_3$) *δ* -61.52. **IR (neat):** *v* = 3407, 2962, 2927, 2866, 2333, 1729, 1613, 1532, 1269, 1106, 1062, 821, 739, 594 cm^{-1}. **HRMS(ESI)**: calculated for $C_{19}H_{23}NF_3$ $[M+H]^+$:322.1704, found: 322.1779.

N-(2-mesitylpropyl)-4-phenoxyaniline (2z)

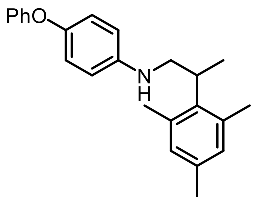

Prepared following general procedure 1, **2z** was obtained after purification by column chromatography (pentane: Et_2O 50:1) as a pale-yellow oil (22.1 mg, 64 µmol, 64%). Rf = 0.32 (pentane: EtOAc 20:1). **^{1}H NMR** (600 MHz, $CDCl_3$) *δ* 7.28 (dd, *J* = 8.7, 7.3 Hz, 2H), 7.01 (tt, *J* = 7.4, 1.1 Hz, 1H), 6.96 – 6.92 (m, 2H), 6.92 – 6.88 (m, 2H), 6.85 (s, 2H), 6.56 (d, *J* = 8.7 Hz, 2H), 3.59 (h, *J* = 7.5 Hz, 1H), 3.47 (d, *J* = 7.9 Hz, 2H), 2.52 – 2.21 (m, 9H), 1.39 (d, *J* = 7.2 Hz, 3H). **^{13}C NMR** (151 MHz, $CDCl_3$) *δ* 159.3, 147.6, 145.0, 136.9, 135.8, 129.6, 122.0, 121.4, 117.2, 114.1, 49.0, 34.7, 21.6, 20.8, 17.4. **IR (neat):** *v* = 3406, 2962, 2923, 2869, 2350, 2156, 2067, 1860, 1729, 1592, 1508, 1484, 1379, 1317, 1226, 1161, 1121, 1075, 1027, 958, 830, 750, 690 cm^{-1}. **HRMS(ESI)**: calculated for $C_{24}H_{28}ON$ $[M+H]^+$:346.2165, found: 346.2167.

***N*-(2-mesitylpropyl)-3-methylaniline (2aa)**

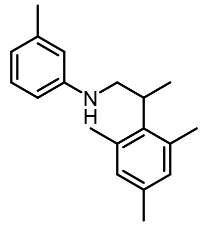

Prepared following general procedure 1, **2aa** was obtained after purification by column chromatography (pentane: Et_2O 200:1) as a pale-yellow oil (16.0 mg, 60 µmol, 60%). Rf = 0.58 (pentane: EtOAc 20:1). **^{1}H NMR** (600 MHz, $CDCl_3$) *δ* 7.06 (t, *J* = 7.9 Hz, 1H), 6.85 (s, 2H), 6.53 (d, *J* = 7.4 Hz, 1H), 6.44 – 6.35 (m, 2H), 3.57 (h, *J* = 7.4 Hz, 1H), 3.46 (d, *J* = 7.8 Hz, 2H), 2.50 – 2.22 (m, 12H), 1.38 (d, *J* = 7.2 Hz, 3H). **^{13}C NMR** (151 MHz, $CDCl_3$) *δ* 148.4, 139.1, 137.1, 135.7, 129.2, 118.3, 113.8, 110.2, 48.5, 34.8, 21.7, 21.6, 20.8, 17.4. **IR (neat):** *v* = 3409, 2961, 2920, 2868, 2729, 2329, 2195, 2111, 1992, 1907, 1807, 1723, 1603, 1483, 1376, 1324, 1266, 1174, 1214, 1174, 1119, 1083, 1032, 994, 958, 922, 850, 767, 691 cm^{-1}. **HRMS(ESI)**: calculated for $C_{19}H_{26}N$ $[M+H]^+$:268.2060, found: 268.2059.

***N*-(2-mesitylpropyl)-3-methoxyaniline (2ab)**

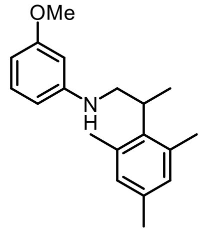

Prepared following general procedure 1, **2ab** was obtained after purification by column chromatography (pentane: Et_2O 50:1) as a pale-yellow oil (19.3 mg, 68 µmol, 68%). Rf = 0.38 (pentane: EtOAc 20:1). **^{1}H NMR** (600 MHz, $CDCl_3$) *δ* 7.07 (t, *J* = 8.1 Hz, 1H), 6.85 (s, 2H), 6.27 (dd, *J* = 8.1, 2.4 Hz, 1H), 6.19 (dd, *J* = 8.1, 2.1 Hz, 1H), 6.13 (t, *J* = 2.3 Hz, 1H), 3.77 (s, 3H), 3.69 – 3.52 (m, 2H), 3.46 (d, *J* = 7.8 Hz, 2H), 2.57 – 2.21 (m, 9H), 1.38 (d, *J* = 7.2 Hz, 3H). **^{13}C NMR** (151 MHz, $CDCl_3$) *δ* 161.0, 149.8, 137.0, 135.8, 130.1, 106.2, 102.6, 98.9, 55.2, 48.4, 34.8, 21.6, 20.8, 17.4. **IR (neat):** *v* =3409, 2958, 2870, 2730, 2326, 2198, 2081, 1989, 1896, 1725, 1608, 1500, 1457, 1378, 1338, 1301, 1268, 1206, 1160, 1120, 1084, 1041, 957, 848, 826, 755, 687 cm^{-1}. **HRMS(ESI)**: calculated for $C_{19}H_{26}ON$ $[M+H]^+$:284.2009, found: 284.2008.

3-bromo-*N*-(2-mesitylpropyl)aniline (2ac)

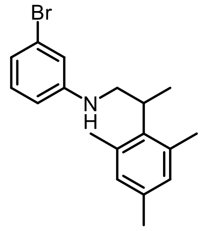

Prepared following general procedure 1, **2ac** was obtained after purification by column chromatography (pentane: Et_2O 200:1) as a pale-yellow oil (20.5 mg, 62 µmol, 62%). Rf = 0.58 (pentane: EtOAc 20:1). **^{1}H NMR** (600 MHz, $CDCl_3$) *δ* 6.99 (t, *J* = 8.0 Hz, 1H), 6.85 (s, 2H), 6.83 – 6.76 (m, 1H), 6.69 (t, *J* = 2.1 Hz, 1H), 6.45 (dd, *J* = 8.4, 2.3 Hz, 1H), 3.65 (s, 1H), 3.55 (h, *J* = 7.5 Hz, 1H), 3.43 (dd, *J* = 7.9, 2.1 Hz, 2H), 2.52 – 2.22 (m, 9H), 1.37 (d, *J* = 7.2 Hz, 3H). **^{13}C NMR** (151 MHz, $CDCl_3$) *δ* 149.6, 136.5, 135.9, 130.6, 123.4, 120.0, 115.4, 111.7, 48.2, 34.6, 21.6, 20.8, 17.4. **IR (neat):** *v* = 3409, 2963, 2922, 2869, 2729, 2337, 2166, 2067, 1994, 1712, 1594, 1480, 1417, 1378, 1321, 1249, 1165, 1119, 1068, 1031, 984, 847, 760, 680 cm^{-1}. **HRMS(ESI)**: calculated for $C_{18}H_{23}NBr$ $[M+H]^+$:332.1008, found: 332.1012.

3-bromo-*N*-(2-mesitylpropyl)aniline (2ad)

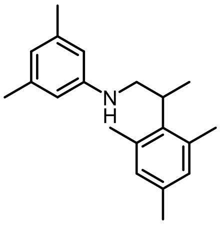

Prepared following general procedure 1, **2ad** was obtained after purification by column chromatography (pentane: Et_2O 200:1) as a pale-yellow oil (19.1 mg, 68 μmol, 68%). Rf = 0.55 (pentane: EtOAc 20:1). **^{1}H NMR** (600 MHz, $CDCl_3$) *δ* 6.85 (s, 2H), 6.37 (s, 1H), 6.22 (s, 2H), 3.56 (h, *J* = 7.4 Hz, 1H), 3.51 (s, 1H), 3.45 (d, *J* = 7.8 Hz, 2H), 2.36 (d, *J* = 21.1 Hz, 6H), 2.28 (s, 3H), 2.24 (s, 6H), 1.38 (d, *J* = 7.2 Hz, 3H). **^{13}C NMR** (151 MHz, $CDCl_3$) *δ* 148.4, 139.0, 137.1, 135.7, 119.3, 111.0, 48.5, 34.8, 21.6, 21.6, 20.8, 17.4. **IR (neat):** *v* = 3409, 2961, 2918, 2867, 2728, 2329, 2083, 1993, 1729, 1601, 1509, 1472, 1375, 1334, 1257, 1186, 1117, 1087, 1031, 956, 850, 820, 744, 690 cm^{-1}. **HRMS(ESI)**: calculated for $C_{20}H_{27}NNa$ $[M+Na]^+$:304.2036, found: 304.2043.

N-(2-mesitylpropyl)-2-methylaniline (2ae)

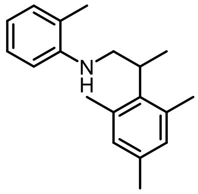

Prepared following general procedure 1, **2ae** was obtained after purification by column chromatography (pentane: Et_2O 200:1) as a pale-yellow oil (9.4 mg, 35 μmol, 35%). Rf = 0.55 (pentane: EtOAc 20:1). **^{1}H NMR** (600 MHz, $CDCl_3$) *δ* 7.12 (td, *J* = 7.7, 1.6 Hz, 1H), 7.01 (d, *J* = 7.2 Hz, 1H), 6.84 (s, 2H), 6.71 – 6.55 (m, 2H), 3.61 (h, *J* = 7.4 Hz, 1H), 3.55 – 3.45 (m, 2H), 3.43 (s, 1H), 2.50 – 2.19 (m, 9H), 1.91 (s, 3H), 1.40 (d, *J* = 7.2 Hz, 3H). **^{13}C NMR** (151 MHz, $CDCl_3$) *δ* 146.3, 136.9, 135.8, 130.2, 127.3, 122.2, 116.9, 109.9, 48.4, 34.6, 21.6, 20.8, 17.4, 17.3. **IR (neat):** *v* = 3409, 2963, 2921, 2867, 2730, 2327, 2081, 1992, 1876, 1605, 1509, 1475, 1447, 1377, 1315, 1258, 1216, 1159, 1134, 1030, 919, 850, 744, 716 cm^{-1}. **HRMS(ESI)**: calculated for $C_{19}H_{26}N$ $[M+H]^+$:268.2060, found: 268.2064.

N-(2-mesitylpropyl)-2-methoxyaniline (2af)

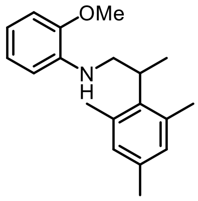

Prepared following general procedure 1, **2af** was obtained after purification by column chromatography (pentane: Et_2O 50:1) as a pale-yellow oil (8.8 mg, 35 μmol, 35%). Rf = 0.35 (pentane: EtOAc 20:1). **^{1}H NMR** (600 MHz, $CDCl_3$) *δ* 6.86 (td, *J* = 7.7, 1.4 Hz, 1H), 6.84 (s, 2H), 6.75 (dd, *J* = 7.9, 1.4 Hz, 1H), 6.65 (td, *J* = 7.7, 1.5 Hz, 1H), 6.61 (dd, *J* = 7.8, 1.5 Hz, 1H), 4.28 (s, 1H), 3.78 (s, 3H), 3.57 – 3.48 (m, 2H), 3.44 (dd, *J* = 11.8, 6.2 Hz, 1H), 2.49 – 2.20 (m, 9H), 1.39 (d, *J* = 6.9 Hz, 3H). **^{13}C NMR** (151 MHz, $CDCl_3$) *δ* 147.1, 138.4, 137.7, 135.6, 121.5, 116.4, 110.0, 109.8, 55.6, 48.4, 35.0, 20.8, 17.4. **IR (neat):** *v* = 3420, 2926, 2730, 2326, 2089, 1994, 1889, 1728, 1601, 1514, 1455, 1343, 1303, 1247, 1221, 1176, 1130, 1028, 957, 896, 850, 733 cm^{-1}. **HRMS(ESI)**: calculated for $C_{19}H_{25}ONNa$ $[M+Na]^+$:306.1828, found: 306.1831.

2-bromo-*N*-(2-mesitylpropyl)aniline (2ag)

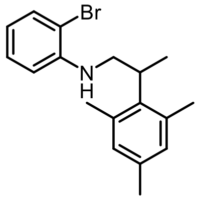

Prepared following general procedure 1, **2ag** was obtained after purification by column chromatography (pentane: Et_2O 200:1) as a pale-yellow oil (15.6 mg, 47 μmol, 47%). Rf = 0.55 (pentane: EtOAc 20:1). **^{1}H NMR** (600 MHz, $CDCl_3$) *δ* 7.39 (dd, *J* = 7.9, 1.5 Hz, 1H), 7.17 (ddd, *J* = 8.5, 7.3, 1.5 Hz, 1H), 6.85 (s, 2H), 6.63 (dd, *J* = 8.1, 1.5 Hz, 1H), 6.55 (td, *J* = 7.6, 1.5 Hz, 1H), 4.32 (t, *J* = 5.7 Hz, 1H), 3.58 (h, *J* = 7.3 Hz, 1H), 3.54 – 3.44 (m, 2H), 2.45 – 2.22 (m, 9H), 1.42 (d, *J* = 7.2 Hz, 3H). **^{13}C NMR** (151 MHz, $CDCl_3$) *δ* 145.0, 136.8, 135.9, 132.5,

129.7, 128.6, 117.7, 111.4, 110.0, 48.3, 34.7, 21.6, 20.8, 17.4. **IR (neat):** *v* = 3396, 3063, 2962, 2922, 2868, 2730, 2330, 2080, 2883, 1731, 1595, 1505, 1455, 1377, 1319, 1287, 1215, 1162, 1133, 1080, 1017, 921, 850, 739, 661 cm^{-1}. **HRMS(ESI)**: calculated for $C_{18}H_{22}ONBrNa$ $[M+Na]^+$:354.0828, found: 354.0832.

***N*-(2-mesitylpropyl)benzo[*d*][1,3]dioxol-5-amine (2ah)**

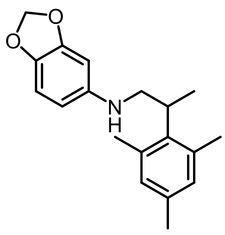

Prepared following general procedure 1, **2ah** was obtained after purification by column chromatography (pentane: Et_2O 50:1) as a pale-yellow oil (18.1 mg, 61 µmol, 61%). Rf = 0.30 (pentane: EtOAc 20:1). **^{1}H NMR** (400 MHz, $CDCl_3$) *δ* 6.83 (s, 2H), 6.65 (d, *J* = 8.3 Hz, 1H), 6.29 (d, *J* = 2.3 Hz, 1H), 6.11 (dd, *J* = 8.3, 2.3 Hz, 1H), 5.89 – 5.80 (m, 2H), 3.63 – 3.51 (m, 1H), 3.41 (dd, *J* = 7.7, 2.1 Hz, 2H), 2.44 – 2.19 (m, 9H), 1.37 (d, *J* = 7.2 Hz, 3H). **^{13}C NMR** (101 MHz, $CDCl_3$) *δ* 148.5, 142.4, 140.6, 136.8, 135.8, 131.3, 129.6, 108.7, 106.1, 100.8, 97.2, 50.4, 34.4, 21.5, 20.8, 17.5. **IR (neat):** *v* = 3412, 2958, 2911, 2874, 2162, 1620, 1489, 1199, 1034, 848, 814, 733, 577 cm^{-1}. **HRMS(ESI)**: calculated for $C_{19}H_{24}O_2N$ $[M+H]^+$:298.1729, found: 298.1803.

Butyl 4-((2-mesitylpropyl)amino)benzoate (2ai)

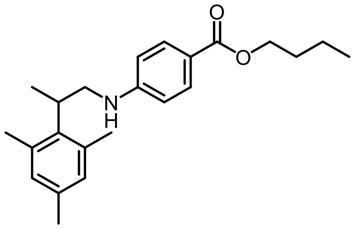

Prepared following general procedure 1, **2ai** was obtained after purification by column chromatography (pentane: Et_2O 50:1) as a pale-yellow oil (16.2 mg, 46 µmol, 46%). Rf = 0.30 (pentane: EtOAc 20:1). **^{1}H NMR** (500 MHz, $CDCl_3$) *δ* 7.89 – 7.85 (m, 2H), 6.83 (s, 2H), 6.61 (d, *J* = 7.7 Hz, 2H), 4.27 (t, *J* = 6.6 Hz, 2H), 3.61 – 3.55 (m, 1H), 3.54 – 3.50 (m, 2H), 2.41 – 2.23 (m, 9H), 1.77 – 1.68 (m, 2H), 1.53 – 1.42 (m, 2H), 1.39 (d, *J* = 6.9 Hz, 3H), 0.97 (t, *J* = 7.4 Hz, 3H). **^{13}C NMR** (126 MHz, $CDCl_3$) *δ* 166.9, 140.4, 136.3, 136.0, 132.0, 131.6, 130.6, 130.5, 128.3, 64.4, 34.4, 31.1, 23.1, 21.5, 20.8, 19.5, 17.4, 13.9. **IR (neat):** *v* = 3380, 2958, 2871, 2319, 1602, 1527, 1464, 1380, 1334, 1268, 1168, 1105, 1024, 842, 769, 734, 700, 580 cm-1. **HRMS(ESI)**: calculated for C23H32O2N [M+H]+ :354.2355, found: 354.2428.

(1*R*,2*S*,5*R*)-2-isopropyl-5-methylcyclohexyl 4-((2-mesitylpropyl)amino)benzoate (2aj)

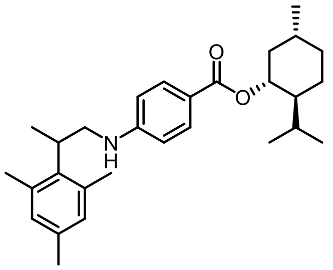

Prepared following general procedure 1, **2aj** was obtained after purification by column chromatography (pentane: Et_2O 30:1) as a pale-yellow oil (30.5 mg, 70 µmol, 70%). Rf = 0.26 (pentane: EtOAc 20:1). **^{1}H NMR** (600 MHz, $CDCl_3$) *δ* 7.88 – 7.85 (m, 2H), 6.83 (s, 2H), 6.64 – 6.57 (m, 2H), 4.88 (td, *J* = 10.8, 4.4 Hz, 1H), 3.60 – 3.54 (m, 1H), 3.54 – 3.50 (m, 2H), 2.41 – 2.23 (m, 9H), 2.14 – 2.06 (m, 1H), 1.99 – 1.90 (m, 1H), 1.75 – 1.67 (m, 2H), 1.58 – 1.49 (m, 2H), 1.38 (d, *J* = 7.0 Hz, 3H), 1.17 – 1.11 (m, 1H), 1.10 – 1.03 (m, 1H), 0.96 – 0.89 (m, 8H), 0.79 (dd, *J* = 7.0, 1.4 Hz, 3H). **^{13}C NMR** (151 MHz, $CDCl_3$) *δ* 166.3, 140.4, 136.4, 136.3, 136.0, 132.1, 131.6, 130.5, 128.3, 74.1, 47.5, 41.3, 34.6, 31.6, 26.6, 23.9, 22.2, 21.6, 20.9, 20.8, 17.4, 17.4, 16.7. **IR (neat):** *v* = 3382, 2951, 2925, 2865, 1603, 1526, 1457, 1334, 1269, 1169, 1105,

1033, 969, 842, 735, 700, 647 cm^{-1}. **HRMS(ESI)**: calculated for $C_{29}H_{42}O_2N$ $[M+H]^+$:436.3138, found: 436.3211.

(*R*)-2,8-dimethyl-2-((4*R*,8*R*)-4,8,12-trimethyltridecyl)chroman-6-yl4-((2-mesityl propyl)amino)benzoate (2ak)

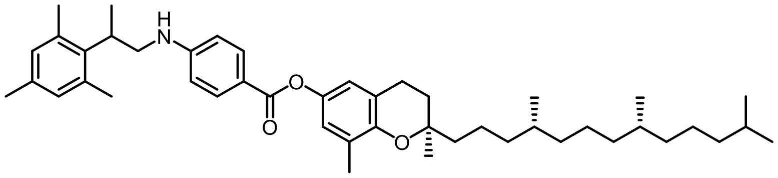

Prepared following general procedure 1, **2ak** was obtained after purification by column chromatography (pentane: Et_2O 25:1) as a pale-yellow oil (28.6 mg, 42 µmol, 42%). Rf = 0.22 (pentane: EtOAc 20:1). **^{1}H NMR** (600 MHz, $CDCl_3$) *δ* 8.02 – 7.98 (m, 2H), 6.84 (s, 2H), 6.81 – 6.67 (m, 4H), 3.64 – 3.52 (m, 3H), 2.79 – 2.70 (m, 2H), 2.42 – 2.22 (m, 9H), 2.16 (s, 3H), 1.86 – 1.78 (m, 1H), 1.78 – 1.72 (m, 1H), 1.62 – 1.49 (m, 3H), 1.44 – 1.39 (m, 3H), 1.40 – 1.35 (m, 2H), 1.33 – 1.19 (m, 13H), 1.17 – 1.11 (m, 3H), 1.11 – 1.02 (m, 3H), 0.90 – 0.80 (m, 12H). **^{13}C NMR** (76 MHz, $CDCl_3$) *δ* 166.1, 152.2, 149.7, 143.1, 136.3, 136.1, 132.3, 127.3, 121.6, 121.0, 119.5, 111.7, 76.2, 47.8, 40.3, 39.5, 37.6, 37.4, 34.7, 32.9, 32.9, 31.2, 28.1, 25.0, 24.6, 24.4, 22.9, 22.8, 22.6, 21.6, 21.1, 20.8, 19.9, 19.8, 17.4, 16.3. **IR (neat):** *v* = 3428, 2922, 2858, 2189, 2159, 1762, 1602, 1528, 1466, 1373, 1261, 1220, 1167, 1068, 901, 760, 731, 691, 588 cm^{-1}. **HRMS(ESI)**: calculated for $C_{46}H_{67}O_3NNa$ $[M+Na]^+$:704.5121, found: 704.5012.

***N*-(2-mesitylbutyl)aniline (2al)**

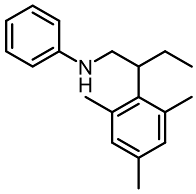

Prepared following general procedure 1, **2al** was obtained after purification by column chromatography (pentane: Et_2O 200:1) as a pale-yellow oil (4.5 mg, 17 µmol, 17%). Rf = 0.65 (pentane: EtOAc 20:1). **^{1}H NMR** (600 MHz, $CDCl_3$) δ 7.18 – 7.11 (m, 2H), 6.82 (d, *J* = 20.6 Hz, 2H), 6.71 – 6.64 (m, 1H), 6.55 (d, *J* = 7.7 Hz, 2H), 3.51 (dd, *J* = 11.8, 6.2 Hz, 1H), 3.47 – 3.34 (m, 2H), 2.36 (s, 3H), 2.25 (s, 3H), 2.23 (s, 3H), 1.84 (p, *J* = 7.4 Hz, 2H), 1.55 (s, 1H), 0.88 (t, *J* = 7.4 Hz, 3H). **^{13}C NMR** (151 MHz, $CDCl_3$) δ 138.4, 136.5, 135.7, 135.4, 131.4, 129.5, 129.4, 117.4, 113.1, 47.4, 42.1, 25.4, 22.1, 21.5, 20.8, 12.9. **IR (neat):** *v* = 3400, 2922, 2856, 2732, 2320, 2164, 2070, 2023, 1917, 1686, 1601, 1504, 1437, 1377, 1313, 1253, 1179, 1122, 1074, 1025, 989, 907, 852, 747, 691 cm^{-1}. **HRMS(ESI)**: calculated for $C_{19}H_{26}N$ $[M+H]^+$:268.2060, found: 268.2059.

***N*-(2-mesitylbutyl)aniline (2am)**

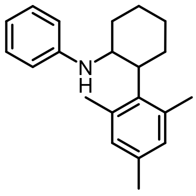

Prepared following general procedure 1, **2am** was obtained after purification by column chromatography (pentane: Et_2O 200:1) as a pale-yellow oil (5.3 mg, 18 µmol, 18%). Rf = 0.63 (pentane: EtOAc 20:1). **^{1}H NMR** (600 MHz, $CDCl_3$) δ 7.18 – 7.11 (m, 2H), 6.82 (d, *J* = 20.6 Hz, 2H), 6.71 – 6.64 (m, 1H), 6.55 (d, *J* = 7.7 Hz, 2H), 3.51 (dd, *J* = 11.8, 6.2 Hz, 1H), 3.47 – 3.34 (m, 2H), 2.36 (s, 3H), 2.25 (s, 3H), 2.23 (s, 3H), 1.84 (p, *J* = 7.4 Hz, 2H), 1.55 (s, 1H), 0.88 (t, *J* = 7.4 Hz, 3H). **^{13}C NMR** (76 MHz, $CDCl_3$) δ 135.3, 129.2, 129.0, 116.7, 113.4, 54.3, 46.3, 32.1, 28.2, 27.6, 21.1, 20.7. **IR (neat):** *v* = 3421, 2922, 2855, 2323, 1725, 1598, 1498, 1448, 1375, 1308, 1252, 1183, 1136, 1027, 914, 816, 745, 690, 589 cm^{-1}. **HRMS(ESI)**: calculated for $C_{21}H_{28}N$ $[M+H]^+$:294.2216, found: 294.2211.

4.2 General procedure 2 of Smile rearrangement of Sulfonamide

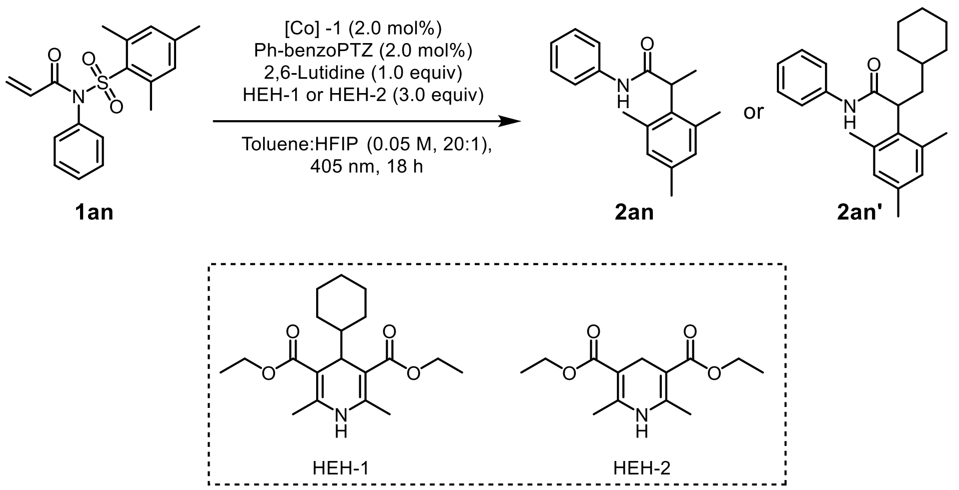

In an oven-dried 4 mL vial equipped with a magnetic stirring bar were added the sulfonamide **1an** (0.10 mmol, 1.0 equiv), [Co] -1 (1.2 mg, 2.0 µmol, 2.0 mol%), Ph-benzoPTZ (0.7 mg, 2.0 µmol, 2.0 mol%) and HEH-1 (100.6 mg, 0.3 mmol, 3.0 equiv) or HEH-2 (76.0 mg, 0.3 mmol, 3.0 equiv). A plastic cap with rubber septum was used to close the vial and the system was purged with an argon balloon for 15 minutes. Toluene (2 mL, 0.05 M) was added followed by HFIP (0.1 mL) and 2,6-Lutidine (11.6µL, 0.1mmol, 1.0 equiv). The vial was then placed in the PhotoRedOx Box (see Materials and Methods for more details about the photochemical setup) and irradiated for 18 hours with or without a cooling fan. The crude was then concentrated under reduced pressure and purified by flash column chromatography on silica gel with Pentane-Et_2O.

2-mesityl-*N*-phenylpropanamide (2an)

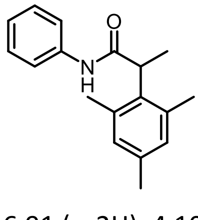

Prepared following general procedure 2 using HEH-2 and conducted without the cooling fan, **2an** was obtained after purification by column chromatography (pentane: EtOAc 50:1) as a white solid (8.0mg, 30 µmol, 30%). Rf = 0.50 (pentane: EtOAc 20:1). **^{1}H NMR** (600 MHz, $CDCl_3$) δ 7.37 (dd, *J* = 8.7, 1.2 Hz, 2H), 7.30 – 7.23 (m, 2H), 7.09 – 7.03 (m, 1H), 6.96 (s, 1H), 6.91 (s, 2H), 4.10 (q, *J* = 7.2 Hz, 1H), 2.55 – 2.07 (m, 9H), 1.51 (d, *J* = 7.2 Hz, 3H). **^{13}C NMR** (151 MHz, $CDCl_3$) δ 173.3, 138.1, 137.1, 137.0, 134.8, 130.5, 129.1, 124.2, 119.9, 42.1, 20.9, 14.9, 0.1. **IR (neat):** v = 3297, 2972, 2922, 2861, 2028, 1662, 1599, 1537, 1494, 1440, 1374, 1325, 1302, 1247, 1206, 1081, 1027, 913, 749, 695 cm^{-1}. **HRMS(ESI)**: calculated for $C_{18}H_{22}NO$ $[M+H]^+$:268.1623, found: 268.1697.

3-cyclohexyl-2-mesityl-*N*-phenylpropanamide (2an')

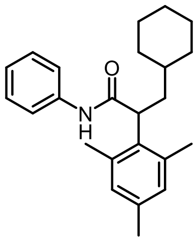

Prepared following general procedure 2 using HEH-1 and conducted with the cooling fan, **2an'** was obtained after purification by column chromatography (pentane: Et_2O 50:1) as a white solid (14.7 mg, 42 µmol, 42%). Rf = 0.55 (pentane: EtOAc 20:1).

The same reaction was carried out without [Co]-1 and the product was obtained in 36% yield.

The reaction was repeated again, without both [Co]-1 and Ph-benzoPTZ and the product was obtained in 28%.

^{1}H NMR (600 MHz, $CDCl_3$) *δ* 7.31 – 7.24 (m, 2H), 7.20 – 7.15 (m, 2H), 6.97 (tt, *J* = 7.3, 1.2 Hz, 1H), 6.82 (s, 2H), 6.80 (s, 1H), 3.97 (t, *J* = 6.4 Hz, 1H), 2.32 – 2.11 (m, 9H), 1.79 (d, *J* = 13.0 Hz, 1H), 1.69 – 1.51 (m, 4H), 1.36 (dt, *J* = 13.4, 6.4 Hz, 1H), 1.32 – 1.23 (m, 1H), 1.17 – 1.04 (m, 3H), 0.94 – 0.79 (m, 2H). **^{13}C NMR (151 MHz, $CDCl_3$)** *δ* 173.0, 138.2, 137.0, 134.7, 129.0, 124.2, 119.9, 45.1, 38.3, 36.4, 33.9, 26.7, 26.4, 26.4, 20.9. **IR (neat):** *v* = 3397, 3322, 2921, 2850, 2165, 1674, 1598, 1514, 1436, 1378, 1308, 1240, 1188, 1125, 1079, 1025, 967, 903, 850, 750, 691 cm^{-1}. **HRMS(ESI)**: calculated for $C_{24}H_{31}NNa$ $[M+Na]^+$:372.2295, found: 372.2298.

5. General procedure and characterization data of photoinduced *α*-functionalisation of 2b

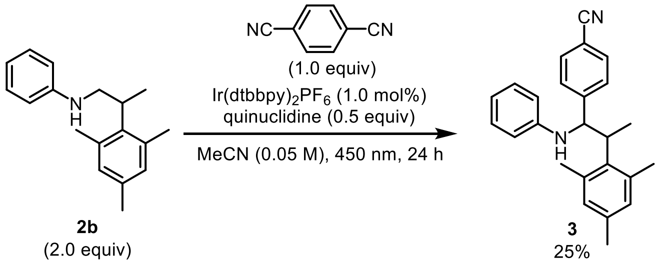

In an oven-dried 4 mL vial equipped with a magnetic stirring bar were added $Ir(dtbbpy)(ppy)_2PF_6$ (2 µmol, 1 mol%), 1,4-dicyanobenzene (0.2 mmol, 1.0 equiv), quinuclidine (0.10 mmol, 0.5 equiv) and **2b** (0.30 mmol, 1.5 equiv). A plastic cap with rubber septum was used to close the vial and the system was purged with an argon balloon for 15 minutes. MeCN (2 mL, 0.05 M) was added and the vial was then placed in the PhotoRedOx Box under 450nm without the cooling fan for 24 hours. The crude was then concentrated under reduced pressure and purified by flash column chromatography on silica gel with Pentane-EtOAc.[4]

4-(2-mesityl-1-(phenylamino)propyl)benzonitrile (3)

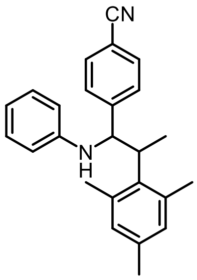

Prepared following reported procedure,[4] **3** was obtained after purification by column chromatography (pentane: EtOAc 20:1) as a yellow oil (8.9 mg, 25 µmol, 25%)i. Rf = 0.45 (pentane: EtOAc 10:1). **^{1}H NMR** (500 MHz, $CDCl_3$) δ 7.70 – 7.62 (m, 4H), 7.00 – 6.95 (m, 2H), 6.93 – 6.92 (m, 1H), 6.83 – 6.79 (m, 1H), 6.61 (tt, *J* = 7.3, 1.1 Hz, 1H), 6.27 – 6.20 (m, 2H), 4.64 (d, *J* = 10.5 Hz, 1H), 3.91 (s, 1H), 3.39 (dq, *J* = 10.5, 7.2 Hz, 1H), 2.39 (s, 3H), 2.35 (s, 3H), 2.27 (s, 3H), 1.18 (d, *J* = 7.2 Hz, 3kH). **^{13}C NMR** (126 MHz, $CDCl_3$) δ 151.0, 147.2, 137.4, 136.9, 136.6, 134.9, 132.7, 131.9, 129.1, 128.5, 119.1, 118.2, 113.7, 111.3, 62.6, 43.0, 21.9, 21.4, 20.8, 16.1. **IR (neat):** *v* = 3389, 2961, 2864, 2224, 2164, 2017, 1983, 1930, 1699, 1497, 1455, 1374, 1312, 1261, 1095, 1024, 743, 693, 571 cm^{-1}. **HRMS(ESI)**: calculated for $C_{25}H_{27}N_2$ $[M+H]^+$:355.2096, found: 355.2170.

6. Mechanistic experiments

6.1 UV-Vis experiments

The UV-Vis spectrum of Ph-benzoPTZ was recorded in toluene.

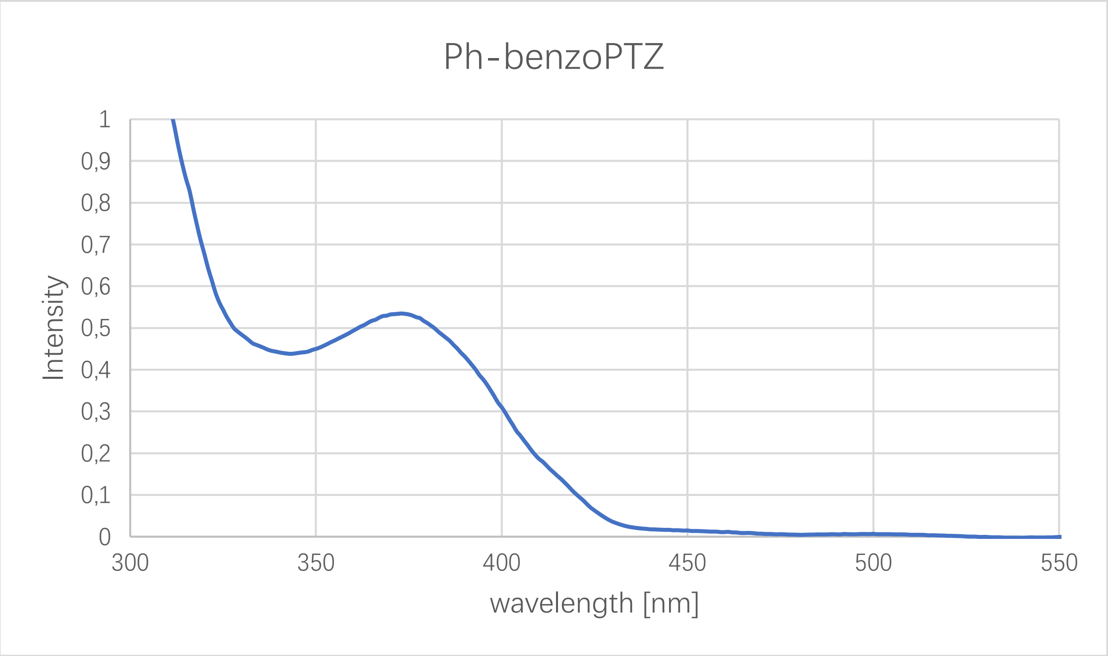

Figure S1 UV-Vis spectrum of Ph-benzoPTZ 0.1mM

The UV-Vis spectrum of Co-1 was recorded in toluene.

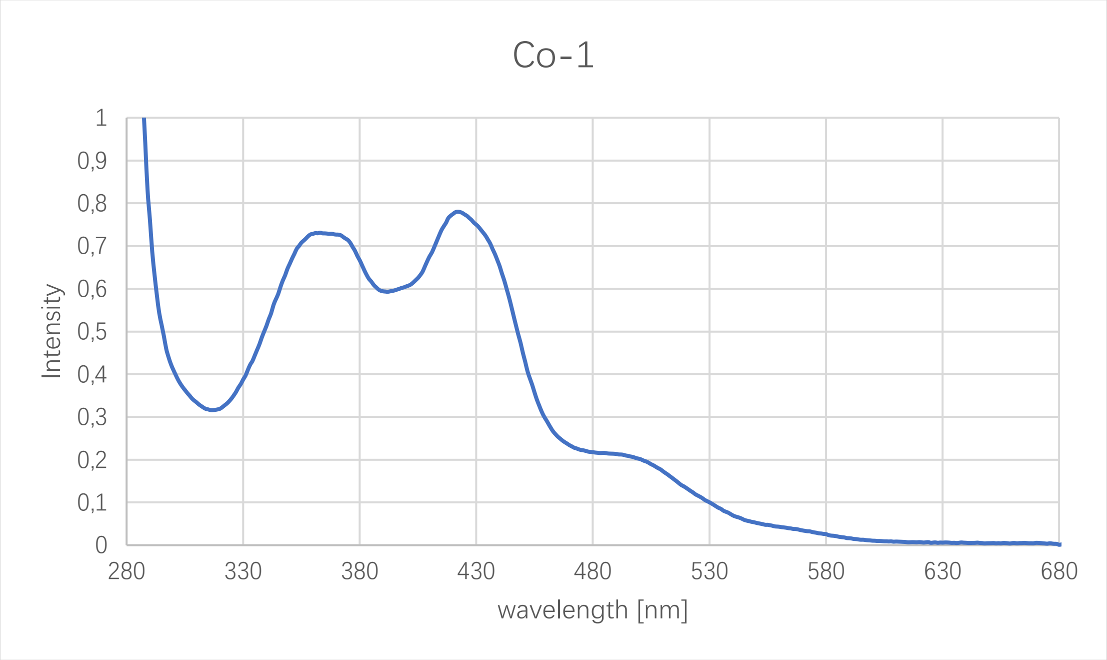

Figure S2 UV-Vis spectrum of Co-1 0.05mM

6.2 Quenching experiments

Excitation wavelength: 340 nm.
Excitation slit: 10 nm, emission slit: 10 nm.

Procedure 1: A 0.1 mM solution of Ph-benzoPTZ in degassed solvent (toluene: HFIP = 20: 1) was added Co-1 (quencher) of different concentration in degassed toluene (quencher concentration = 0.01 mM, 0.02 mM, 0.03 mM, 0.04 mM, 0.05 mM, 0.06 mM). Then, fluorescence of the solutions was measured.
The Co-1 has a broad absorption in visible light region (Figure S1). To consider the inner filter effect of Co-1, the quenching ratio I_0/I, based on the measured steady-state emission intensity (I at 426 nm) of the excited state of Ph-benzoPTZ has been corrected using the following formula.[5-7]

$$\left(\frac{\mathrm{I0}}{\mathrm{I}}\right)\mathrm{corr} = \left(\frac{\mathrm{I0}}{\mathrm{I}}\right)\frac{\mathrm{Abs(PC)}}{\mathrm{Abs(PC)} + \mathrm{Abs(Co)}}\frac{1 - 10^{-(\mathrm{Abs(PC)}+\mathrm{Abs(Co)})}}{1 - 10^{-\mathrm{Abs(PC)}}}$$

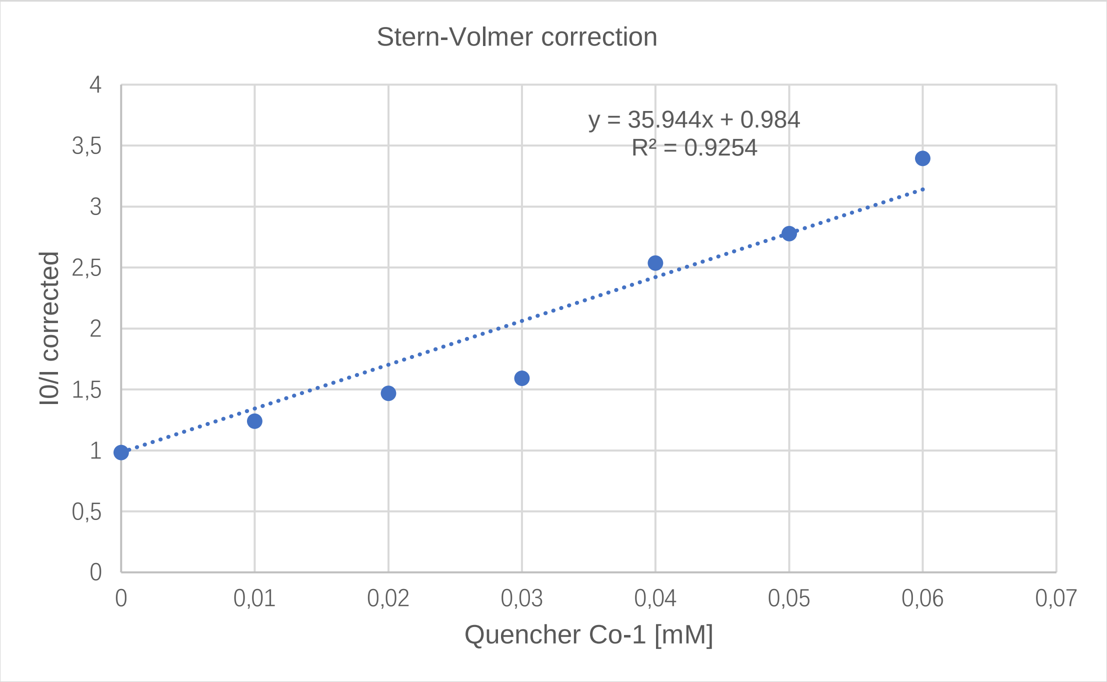

Figure S3 Corrected Stern-Volmer plot of Ph-benzoPTZ with [Co]-1

Excitation wavelength: 340 nm.
Excitation slit: 10 nm, emission slit: 10 nm.

Procedure 2: A 0.05 mM solution of Ph-benzoPTZ in degassed solvent (toluene: HFIP = 20: 1) was added HEH-1 (quencher) of different concentration in degassed toluene (quencher concentration = 0.005 mM, 0.01 mM, 0.02 mM, 0.03 mM, 0.04 mM). Then, fluorescence of the solutions was measured.

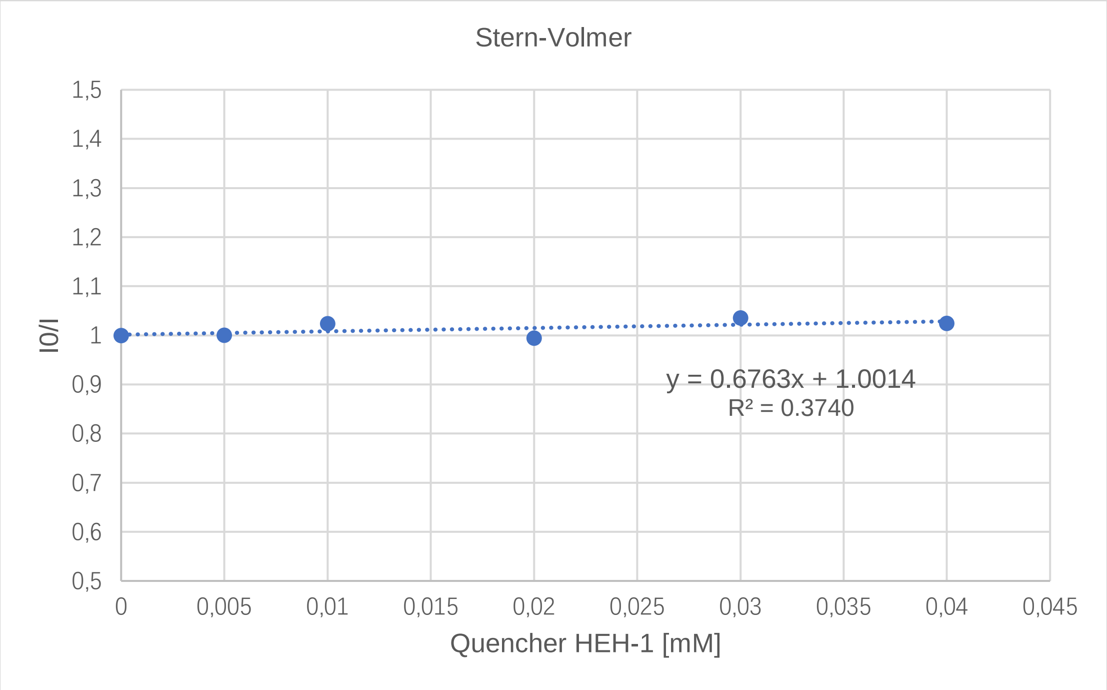

Figure S4 Stern-Volmer plot of Ph-benzoPTZ with HEH-1

6.3 Deuteration experiments

All reactions were conducted General procedure 1 of Smile rearrangement of Sulfonamide, using the different deuterated Hantzsch esters synthesized as reported procedures.[8]

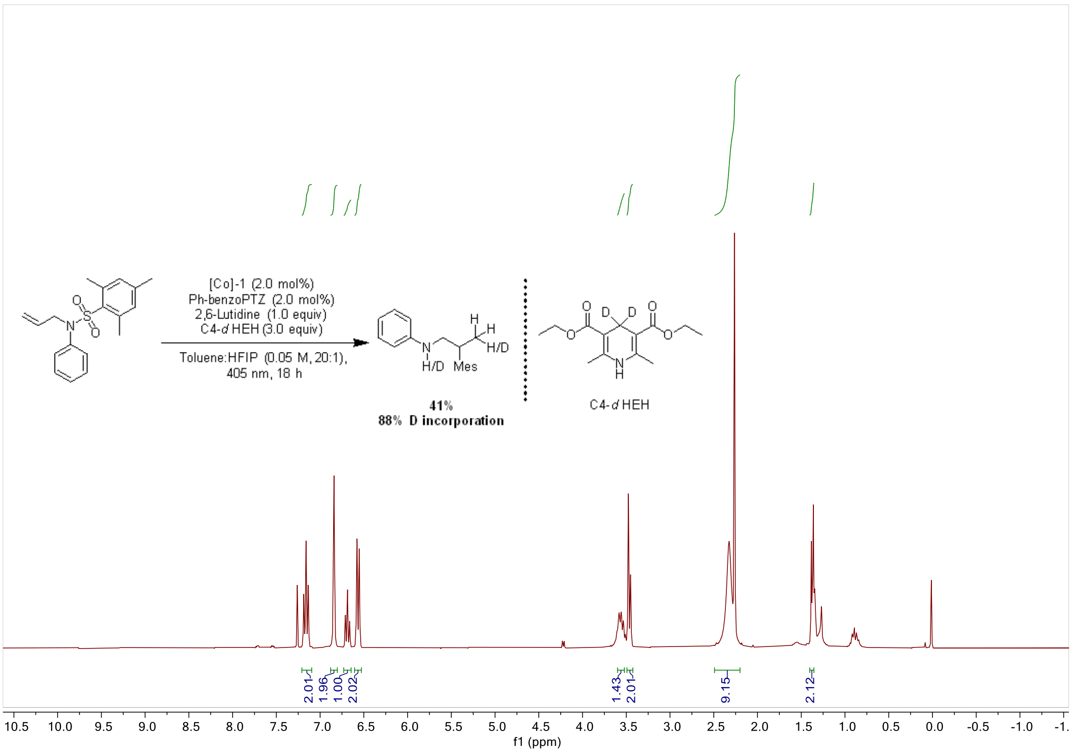

Figure S5 ^{1}H NMR of **2b** from the deuterated experiment with C4-*d* HEH.

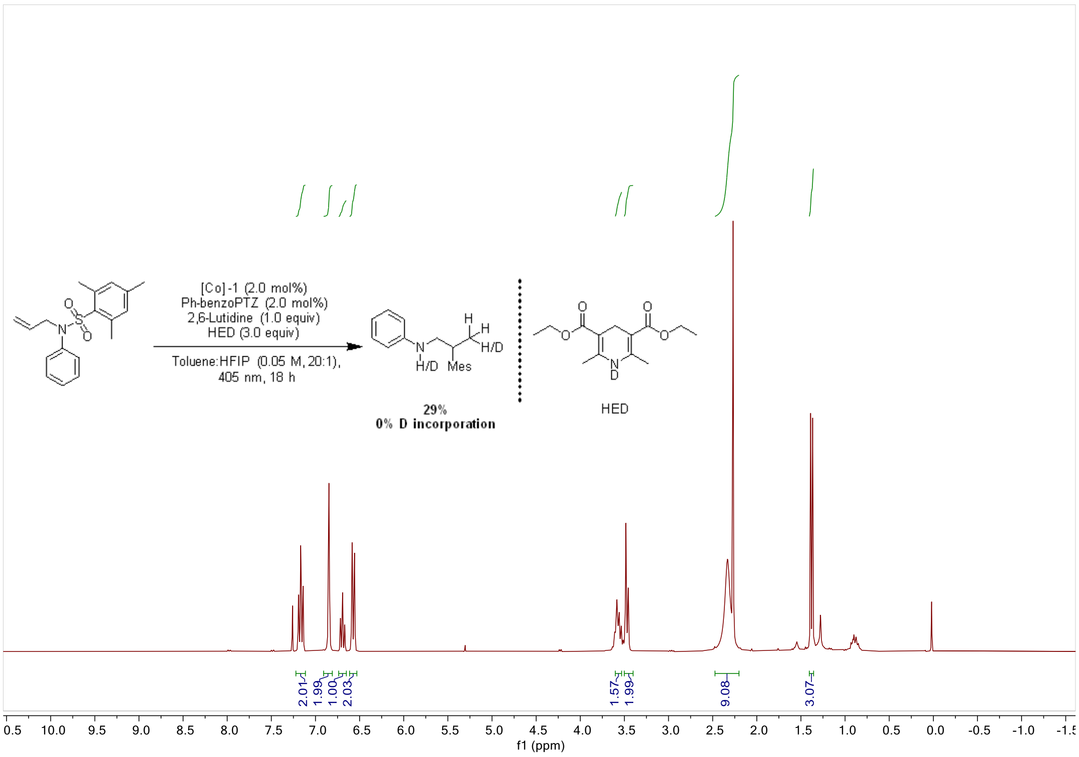

Figure S6 ^{1}H NMR of **2b** from the deuterated experiment with HED.

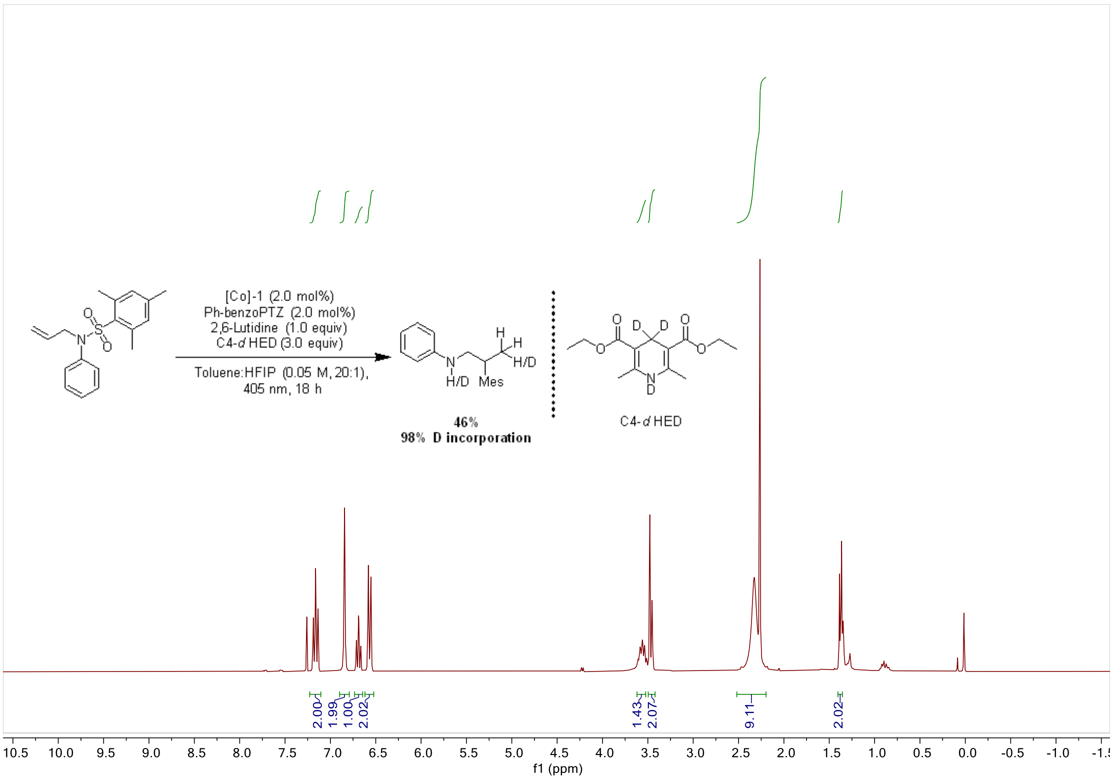

Figure S7 ^{1}H NMR of **2b** from the deuterated experiment with C4-*d* HED.

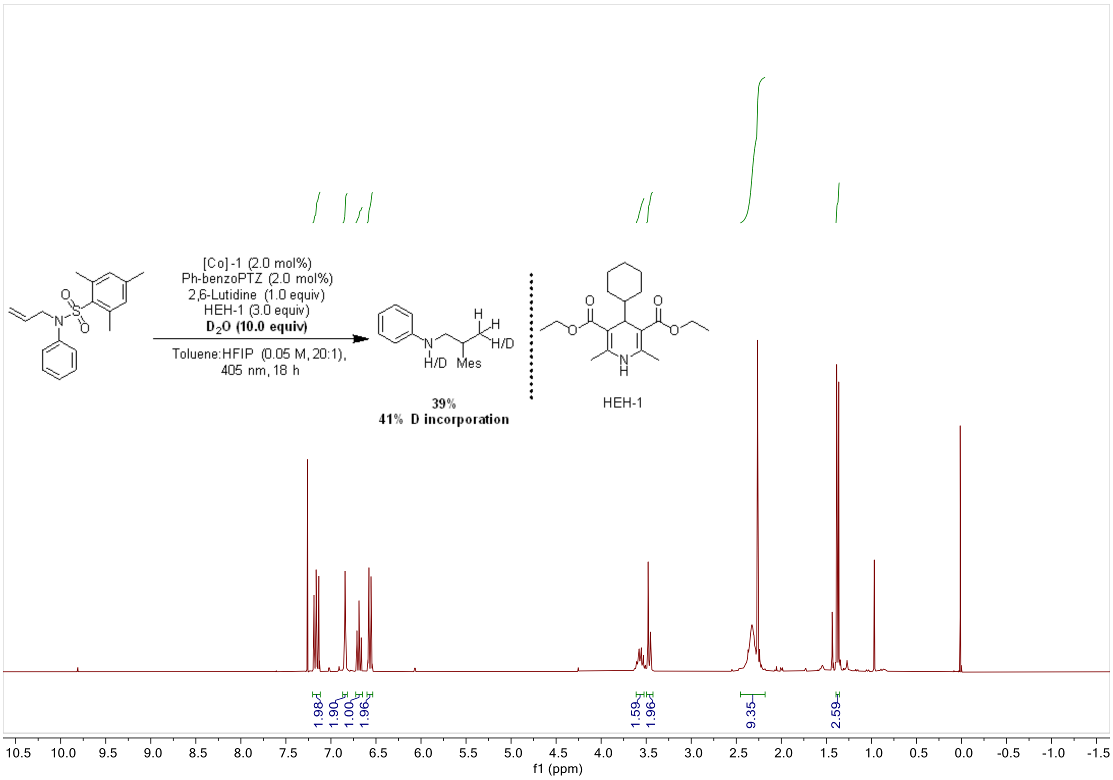

Figure S8 ^{1}H NMR of **2b** from the deuterated experiment with D_2O (10.0 equiv).

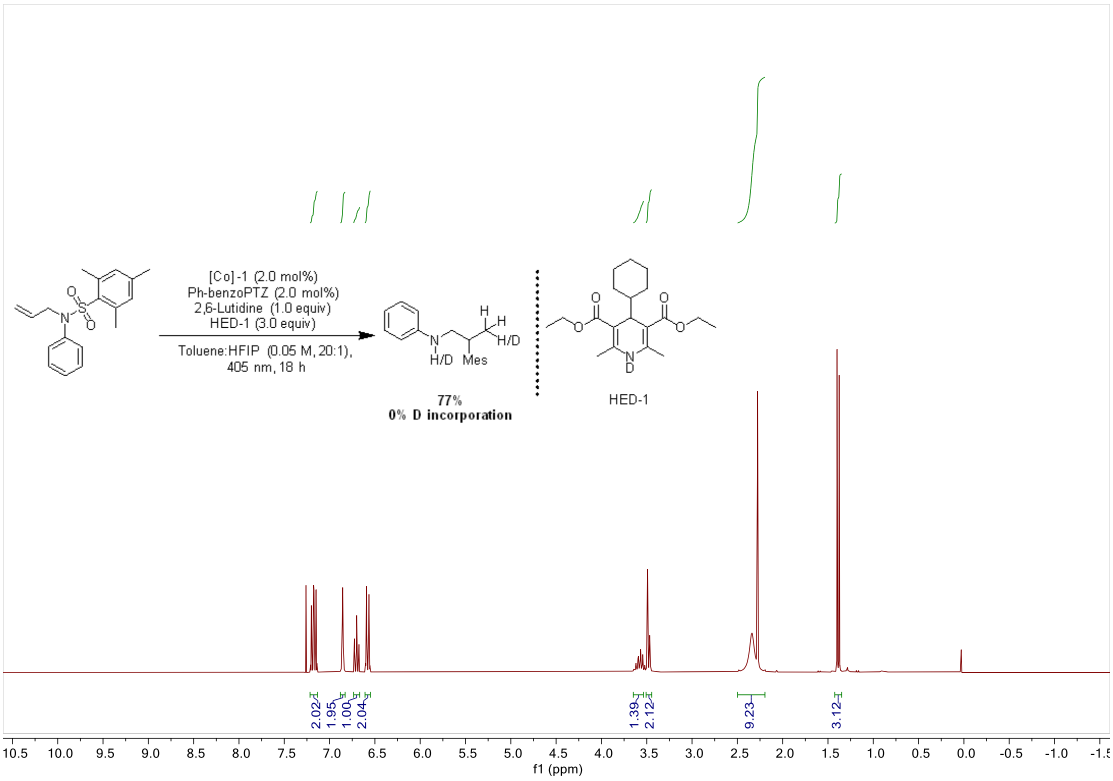

Figure S9 ^{1}H NMR of **2b** from the deuterated experiment with HED-1.

6.4 Trapping with TEMPO

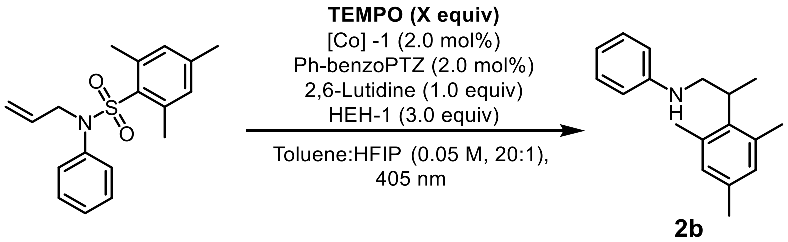

2b

Entry	TEMPO (X equiv)	2b (%)[a]
1	2.0	15
2	5.0	0

[a]yield determined by ^{1}H-NMR analysis with 1,3,5-trimethoxybenzene as an internal standard.

7. Computational details

DFT computational studies were carried out using Gaussian16 program package.[9] All the structures were optimized at the ωB97XD[10] using Def2SVP[11] as the small basis set (indicated as sbs). Frequency calculations were calculated at standard conditions and all the stationary points were characterized as minima (0 imaginary frequencies) or transition states (1 imaginary frequency). Relaxation to reactants and products and IRC calculations were performed when required to further characterize the PES. In addition, single point energy calculations were recalculated with the Def2TZVPP to further refine the potential energies. Solvation was included using the SMD implicit solvent model[12] (C_6H_6 as the solvent) in both optimizations and single point calculations. A benchmarking study was also performed using single point calculations with the B3LYP-D3 (BJ)[13-15], PBE1PBE-D3(BJ)[16] and the M062X[17] functionals (see below).

Additionally, quasi-harmonic (indicated as qh) were applied to the final energies of the key transition states using the Grimme's method[18] (cut-off 100 cm^{-1}) in the Goodvibes program (final energies in the main text[19]).

All the 3D structures were drawn using CYLview 1.0 software.[20]

7.1 Free energy profiles for the reaction pathways of 1a

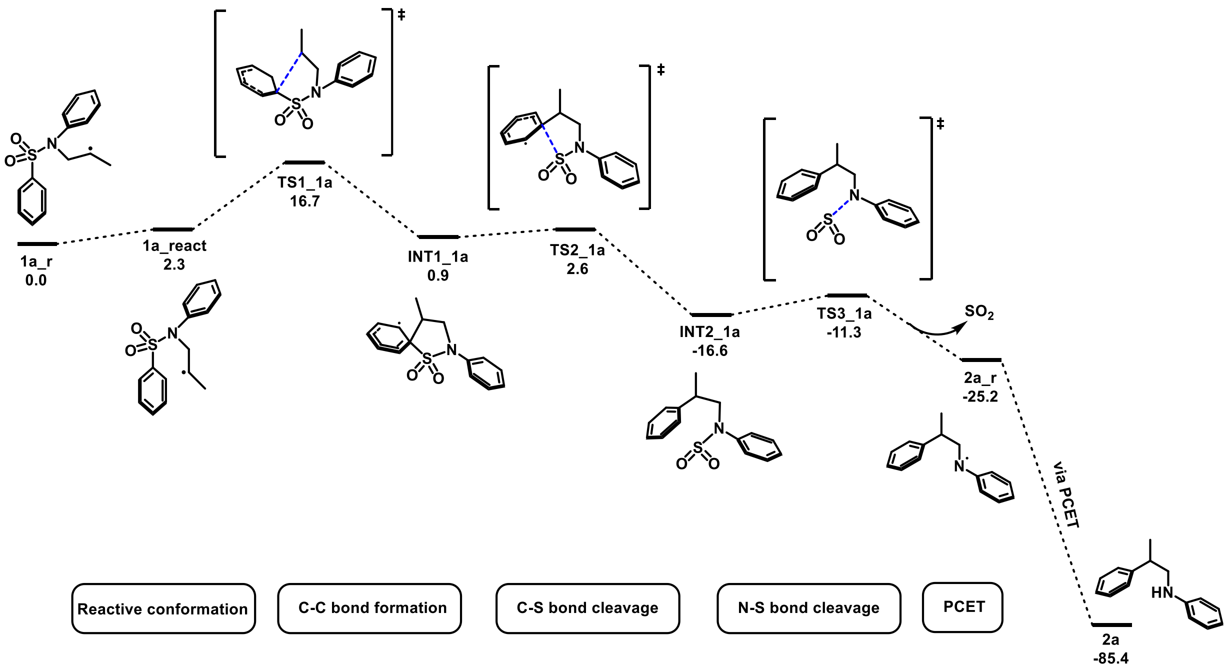

Figure S10 Free energy profile of **1a** for pathway 1: *ipso* attack. The energy is given in kcal/mol.

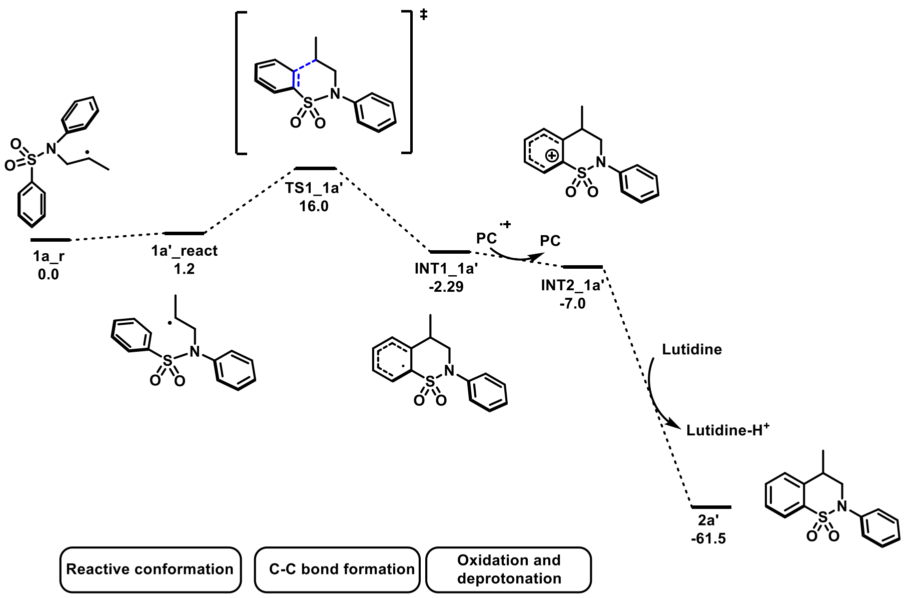

Figure S11 Free energy profile of **1a** for pathway 2: S-*ortho* attack. The energy is given in kcal/mol.

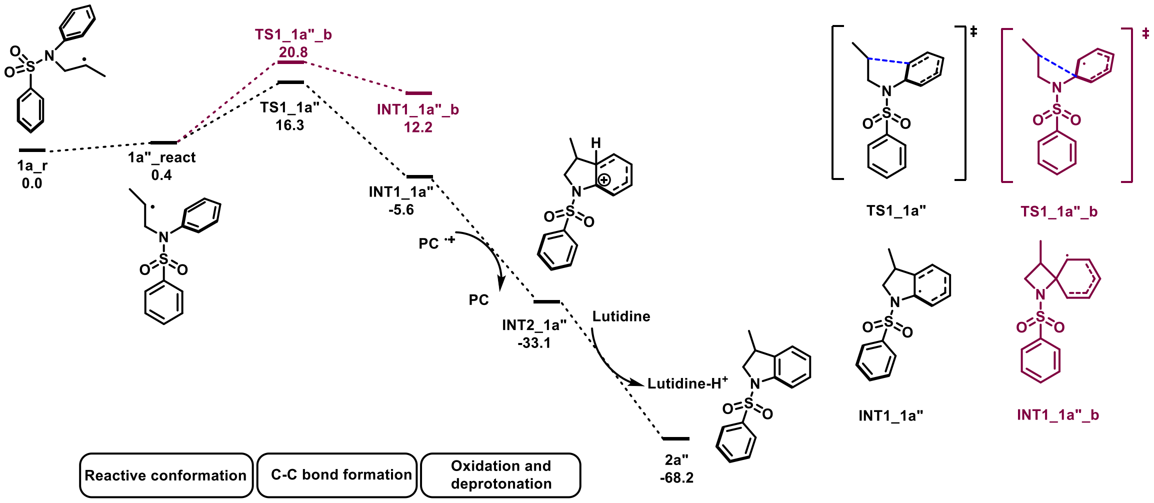

Figure S12 Free energy profile of **1a** for pathway 3: N-*ortho* attack. The energy is given in kcal/mol.

7.2. Benchmarking study of selectivity determining transition states of 1a

The first transition states in all three pathways are the rate determining steps, and their energies are compared: $\Delta G^{\#}_{ipso}$, $\Delta G^{\#}_{S\text{-}ortho}$ and $\Delta G^{\#}_{N\text{-}ortho,}$ for pathways 1, 2 and 3, respectively. The relative difference in Gibbs free energy $\Delta\Delta G^{\#}$ is calculated, where $\Delta\Delta G^{\#}_{o\text{-}i}$ presents the difference in energy between transitions states in pathway 1 and 2, and $\Delta\Delta G^{\#}_{nph\text{-}i}$ is the energy difference between transition states in and pathway 1 and 3. The values are given in kcal/mol. DFT computational studies were carried out on five levels of theory: UωB97XD (small basis set, indicated as sbs), UωB97XD, UB3LYP, UPBE1PBE and UM062X. Additionally, quasi harmonics (indicated as qh) are applied to UωB97XD and UM062X. By obtaining the energies, the predicted ratio of the products is calculated using the energies obtained from UωB97XD qh, and these values are indicated in the manuscript.

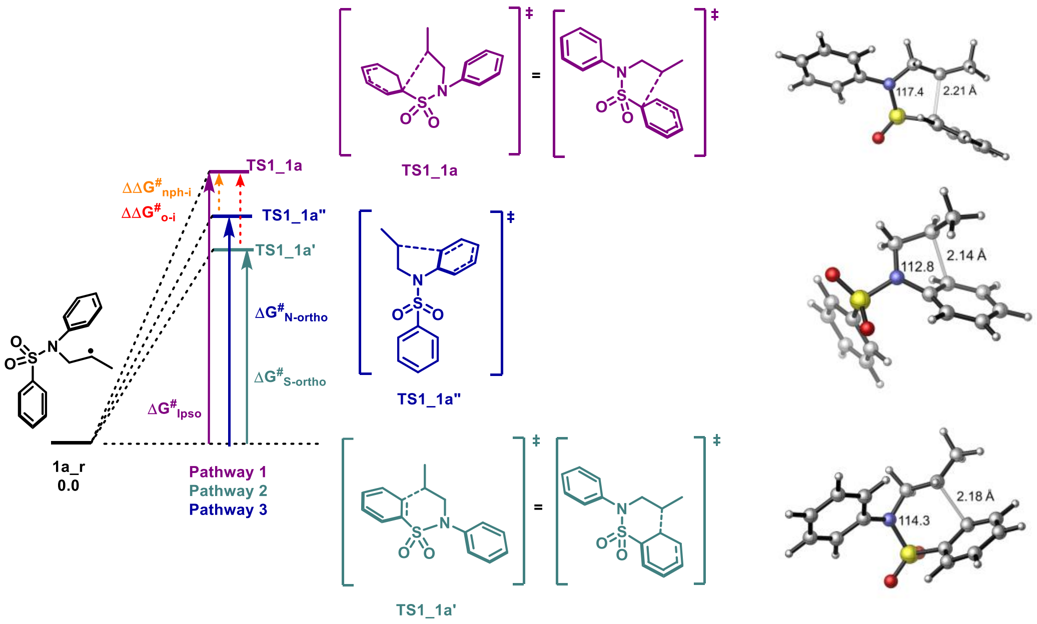

Figure S13 Comparison of the energies of the first transition states for **1a**.

Table S1 Values of Gibbs free energies for different pathways, relative differences of energy and calculated ratio of products. The values of energy are given in kcal/mol.

1a	$\Delta\Delta G^{\neq}_{o\text{-}i}$	$\Delta\Delta G^{\neq}_{nph\text{-}i}$	$\Delta G^{\neq}_{ipso}$	$\Delta G^{\neq}_{S\text{-}ortho}$	$\Delta G^{\neq}_{N\text{-}ortho}$	2a	2a'	2a"
**UωB97XD sbs**	0.06	1.42	14.9	15.0	16.3	50	45	5
**UωB97XD**	-0.70	-0.47	16.7	16.0	16.3	15	51	34
**UB3LYP**	-1.21	-0.49	16.1	14.9	15.6	9	70	21
**UPBE1PBE**	-0.57	-0.34	13.9	13.4	13.6	18	49	33
**UM062X**	-0.47	0.16	17.0	16.5	17.2	25	56	19
**UωB97XD qh**	-0.74	-0.13	15.3	14.6	15.2	17	61	22
**UM062X qh**	-0.50	0.50	15.6	15.1	16.1	26	62	11

7.3 Energy profiles for the reaction pathways of 1b

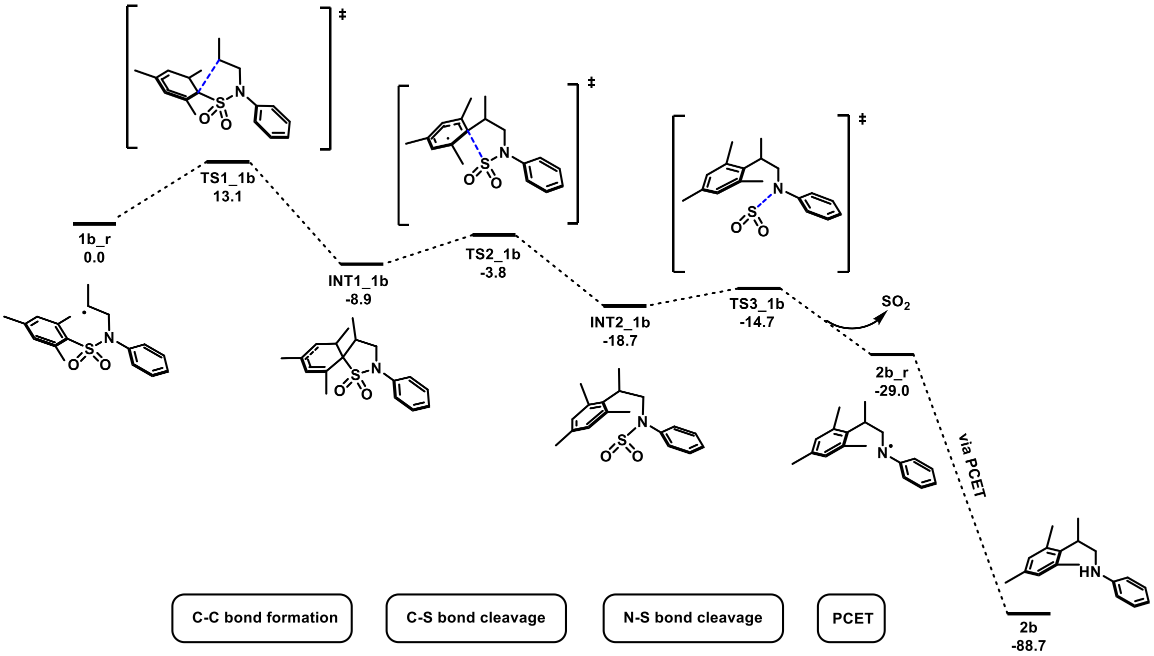

Figure S14 Free energy profile of **1b** for pathway 1: *Ipso* attack. The energy is given in kcal/mol.

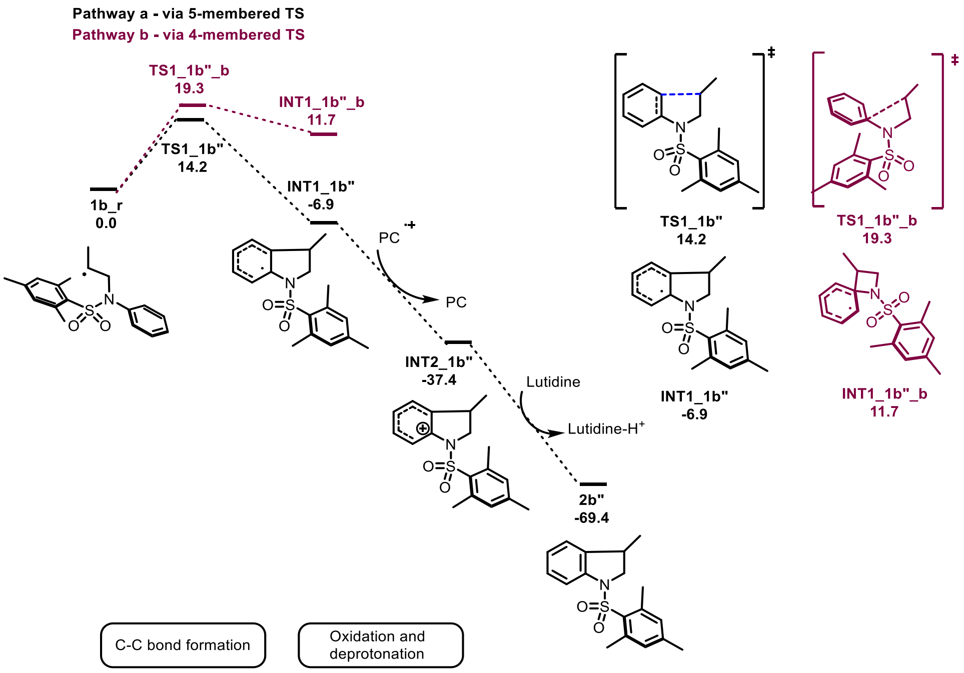

Figure S15 Free energy profile of **1b** for pathway 3: N-*ortho* attack. The energy is given in kcal/mol.

7.4 Benchmarking study of selectivity determining transition states of 1b

The first transition states in both pathways are the rate determining steps, and their energies are compared: $\Delta G^{\#}_{ipso}$ and $\Delta G^{\#}_{N\text{-}ortho,}$ for pathways 1 and 3, respectively. The relative difference in Gibbs free energy $\Delta\Delta G^{\#}$ is calculated: $\Delta\Delta G^{\#}_{nph\text{-}i}$ is the energy difference between transition states in and pathway 1 and 3. The values are given in kcal/mol. DFT computational studies were carried out on five levels of theory: UωB97XD (small basis set, indicated as sbs), UωB97XD, UB3LYP, UPBE1PBE and UM062X. Additionally, quasi harmonics (indicated as qh) are applied to UωB97XD and UM062X. By obtaining the energies, the predicted ratio of the products is calculated using the energies obtained from UωB97XD qh, and these values are indicated in the manuscript.

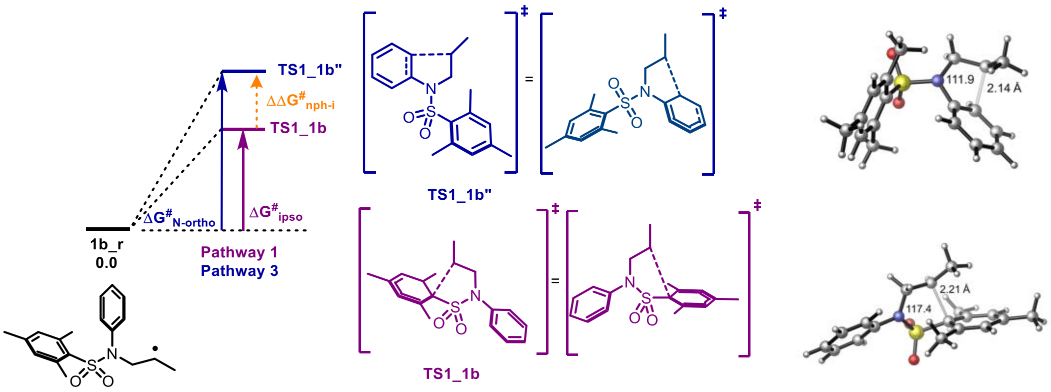

Figure S16 Comparison of the energies of the first transition states for **1b**.

Table S2 Values of Gibbs free energies for different pathways, relative differences of energy and calculated ratio of products. The values of energy are given in kcal/mol.

1b	$\Delta\Delta G^{\neq}_{nph\text{-}i}$	$\Delta G^{\neq}_{ipso}$	$\Delta G^{\neq}_{N\text{-}ortho}$	2b	2b"
UωB97XD sbs	2.39	11.0	13.4	98	2
UωB97XD	1.16	13.1	14.2	88	12
UB3LYP	1.09	12.4	13.5	86	14
UPBE1PBE	1.02	10.3	11.3	85	15
UM062X	1.69	13.5	15.2	95	5
UωB97XD qh	1.61	12.5	14.1	94	6
UM062X qh	2.14	12.9	15.0	97	3

7.5 Energy profiles for the reaction pathways of 1c

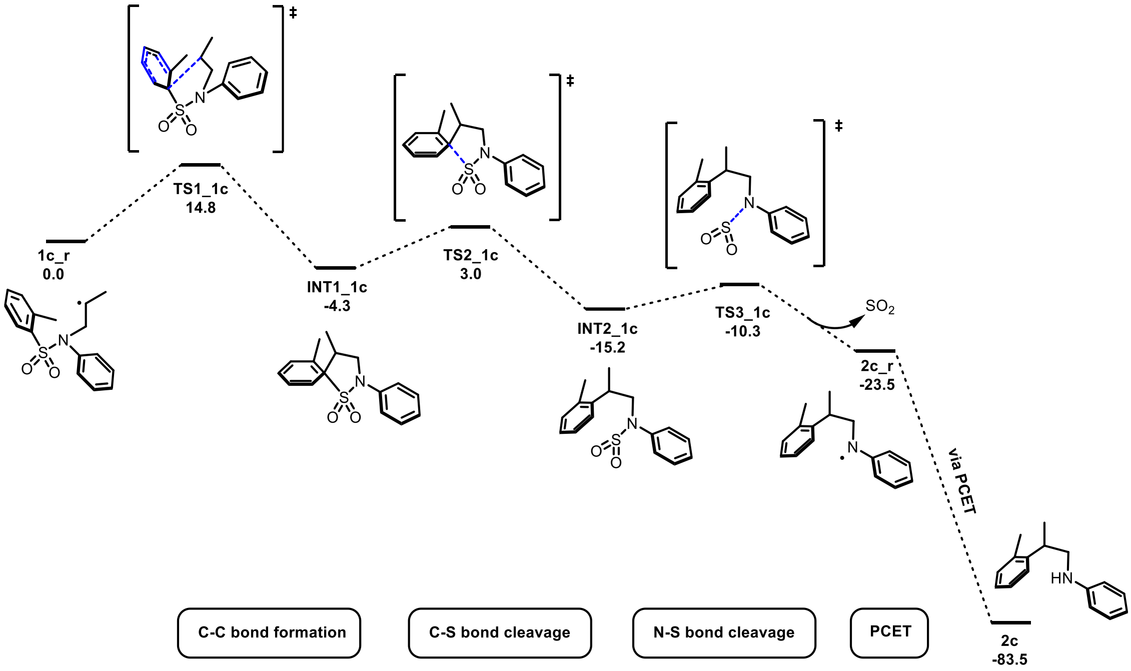

Figure S17 Free energy profile of **1c** for pathway 1: *Ipso* attack. The energy is given in kcal/mol.

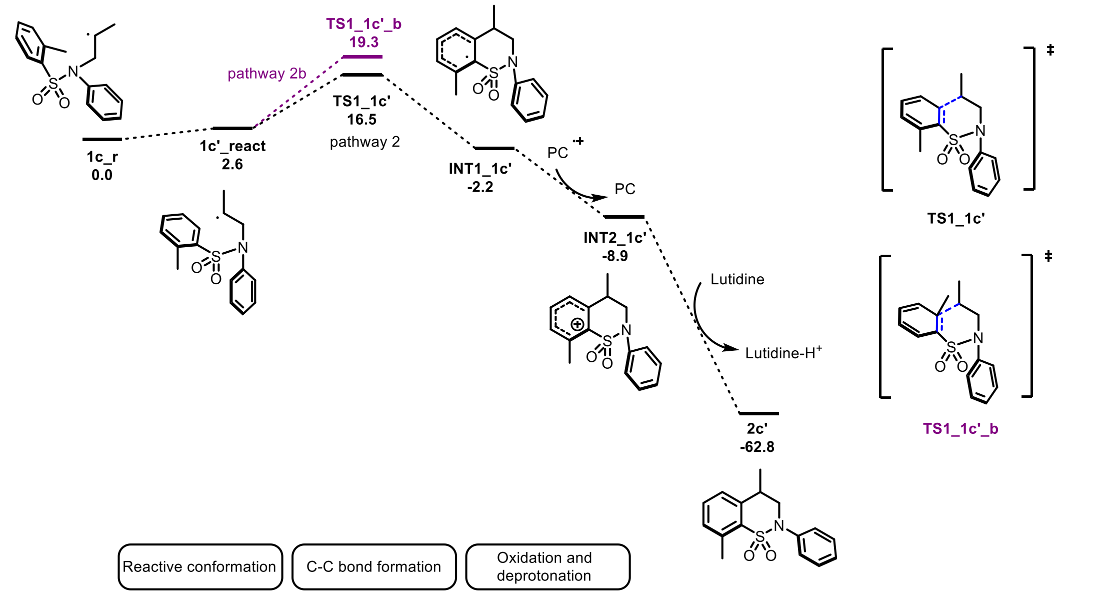

Figure S18 Free energy profile of **1c** for pathway 2: S-*ortho* attack. The energy is given in kcal/mol.

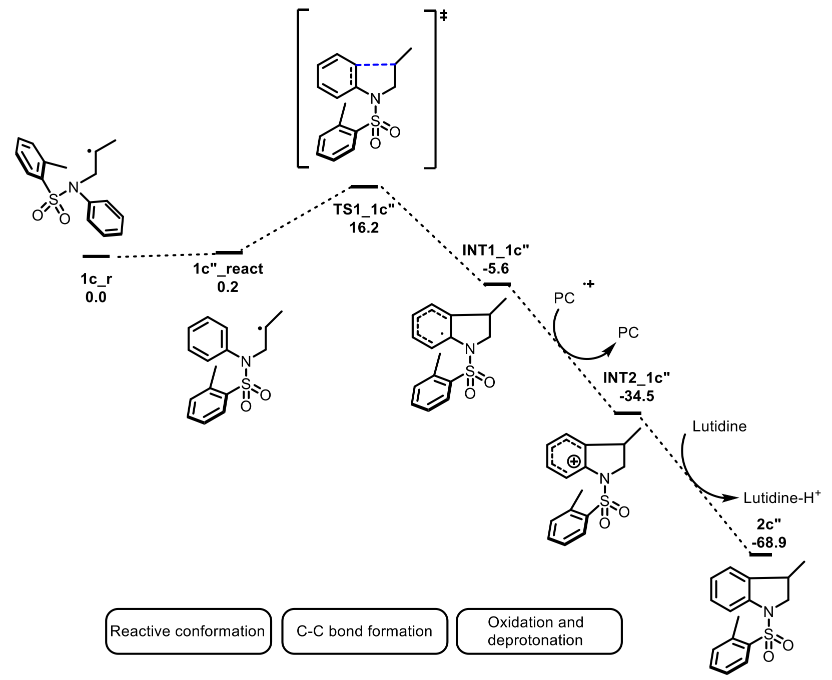

Figure S19 Free energy profile of **1c** for pathway 3: N-*ortho* attack. The energy is given in kcal/mol.

7.6 Benchmarking study of selectivity determining transition states of 1c

The first transition states in all three pathways are rate determining steps, and their energies are compared: $\Delta G^{\#}_{ipso}$, $\Delta G^{\#}_{S\text{-}ortho}$ and $\Delta G^{\#}_{N\text{-}ortho,}$ for pathways 1, 2 and 3, respectively. The relative difference in Gibbs free energy $\Delta\Delta G^{\#}$ is calculated, where $\Delta\Delta G^{\#}_{o\text{-}i}$ presents the difference in energy between transitions states in pathway 1 and 2, and $\Delta\Delta G^{\#}_{nph\text{-}i}$ is the energy difference between transition states in and pathway 1 and 3. The values are given in kcal/mol. DFT computational studies were carried out on five levels of theory: UωB97XD (small basis set, indicated as sbs), UωB97XD, UB3LYP, UPBE1PBE and UM062X. Additionally, quasi harmonics (indicated as qh) are applied to UωB97XD and UM062X. By obtaining the energies, the predicted ratio of the products is calculated using the energies obtained from UωB97XD qh, and these values are indicated in the manuscript.

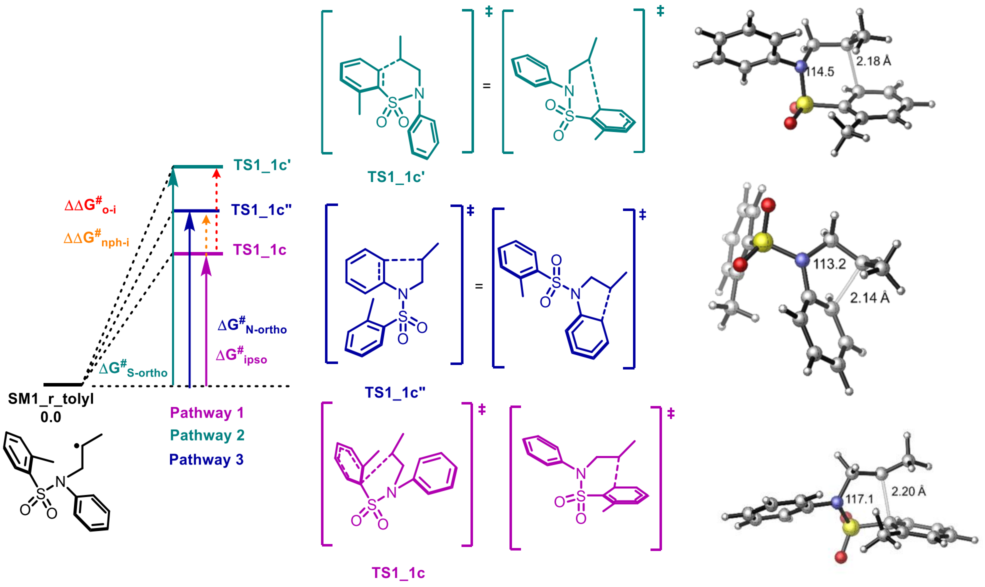

Figure S20 Comparison of the energies of the first transition states for **1c**.

Table S3 Values of Gibbs free energies for different pathways, relative differences of energy and calculated ratio of products. The values of energy are given in kcal/mol.

1c	$\Delta\Delta G^{\neq}_{o\text{-}i}$	$\Delta\Delta G^{\neq}_{nph\text{-}i}$	$\Delta G^{\neq}_{ipso}$	$\Delta G^{\neq}_{S\text{-}ortho}$	$\Delta G^{\neq}_{N\text{-}ortho}$	2c	2c'	2c"
UωB97XD sbs	1.71	3.08	12.8	14.6	15.9	94	5	1
UωB97XD	1.70	1.38	14.8	16.5	16.2	87	5	8
UB3LYP	1.23	0.99	14.5	15.8	15.5	76	10	14
UPBE1PBE	1.62	0.85	12.5	14.2	13.4	77	5	18
UM062X	1.48	1.87	15.3	16.8	17.2	89	7	4
UωB97XD qh	1.71	1.38	13.4	15.1	14.8	87	5	8
UM062X qh	1.49	1.87	13.9	15.4	15.8	89	7	4

7.7 XYZ Coordinates and energies of the calculated species

PC

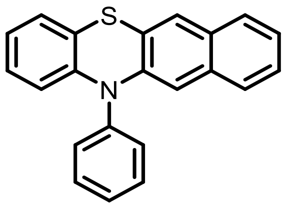

E (UωB97XD sbs) = -1299.284894
E (UωB97XD) = -1300.36595
E (UB3LYP) =-1300.74498
E (UPBE1PBE) = -1299.53483
E (UM062X) = -1300.30818
G_{corr} = 0.263368

0 1

C	-6.97309300	-1.04410900	3.33855500
C	-5.81144300	-1.25997400	2.60188400
C	-5.77429000	-0.94512300	1.24167700
C	-6.89161600	-0.34759100	0.64170100
C	-8.04851100	-0.10965900	1.38642800
C	-8.09311300	-0.47698500	2.72911700
C	-5.10578200	0.64830100	-1.08934400
C	-4.16313000	-0.06129300	-0.28799500
C	-2.85778500	0.36427300	-0.23831200
H	-2.14870500	-0.16116000	0.40499200
C	-2.41119000	1.45140400	-1.03560400
C	-3.34954900	2.13367500	-1.86388800
C	-4.70856700	1.71938600	-1.85248800
H	-6.99991200	-1.30887600	4.39754300
H	-4.92450600	-1.69470800	3.06689600
H	-8.90927700	0.36866100	0.91381100
H	-9.00175500	-0.29837900	3.30781200
H	-5.43885200	2.26797800	-2.45230500
C	-2.90210900	3.22543200	-2.65800000
H	-3.62375100	3.74748400	-3.29125800
C	-1.05629200	1.88324000	-1.02742000
H	-0.33963100	1.35885900	-0.39042000
C	-1.58618100	3.61787400	-2.63065300
H	-1.25312800	4.45682700	-3.24572100
C	-0.65363100	2.94035700	-1.80647400
H	0.38961100	3.26352100	-1.79333900
S	-6.79724800	0.10519000	-1.07377300
N	-4.61636000	-1.18620300	0.45286400
C	-4.25854100	-2.49922500	0.07882100
C	-4.91849100	-3.61231200	0.63042500
C	-3.24671400	-2.73847400	-0.86840900
C	-4.55927300	-4.90648800	0.26426000
H	-5.72935300	-3.47299700	1.34440000
C	-2.90055700	-4.03848200	-1.22643700
H	-2.72848000	-1.90651900	-1.34332100
C	-3.54471400	-5.13734200	-0.66204900
H	-5.09390000	-5.74727300	0.71314600
H	-2.11095800	-4.18663200	-1.96727300
H	-3.26679400	-6.15441800	-0.94494300

PC$^{\cdot+}$

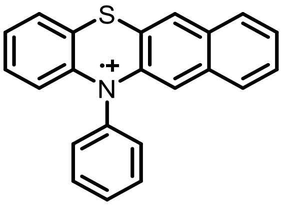

E (UωB97XD sbs) = -1299.063412
E (UωB97XD) = -1300.14631
E (UB3LYP) =-1300.53129
E (UPBE1PBE) = -1299.31999
E (UM062X) = -1300.08319
G_{corr} = 0.263543

1 2

C	-8.34301000	-1.80245400	0.88138500
C	-7.04735100	-1.95292800	0.40472600
C	-6.05919800	-1.05053100	0.80911600
C	-6.37900900	0.05016300	1.61986400
C	-7.68208500	0.17994400	2.10482500
C	-8.65213600	-0.75149100	1.74787200

C -4.29129200 1.21181700
0.46463900
C -4.15758900 -0.02401200 -
0.23376900
C -3.56226200 -0.07929900 -
1.47527600
H -3.52857300 -1.02368500 -
2.02309300
C -3.00255000 1.08612500 -
2.04912200
C -3.06851200 2.31415600 -
1.32398500
C -3.73750600 2.34877900 -
0.07119200
H -9.11786100 -2.50335700
0.56738800
H -6.79482100 -2.75961900 -
0.28498800
H -7.93883000 1.02771300
2.74300100
H -9.66969600 -0.63735100
2.12581300
H -3.82846500 3.30155700
0.45517800
C -2.49496100 3.48097900 -
1.89601700
H -2.54375500 4.42168700 -
1.34321000
C -2.37690100 1.06246600 -
3.32655900
H -2.33688200 0.12152000 -
3.88020700
C -1.89075800 3.42548500 -
3.12830000
H -1.45230300 4.32735800 -
3.56022200
C -1.83283100 2.20768200 -
3.85123700
H -1.35312400 2.18675200 -
4.83161700
S -5.13856800 1.24472600
2.01939400
N -4.70400100 -1.18771500
0.37834300
C -3.95126000 -2.30657600
0.63745200
C -4.52485000 -3.43928700
1.28773000
C -2.56076000 -2.33579400
0.31743600
C -3.75900300 -4.56133500
1.52666000
H -5.55941200 -3.41850500
1.62294700
C -1.81629600 -3.47110300
0.56536500
H -2.06964700 -1.45447500 -
0.09026000
C -2.40763100 -4.59646700
1.15456900
H -4.21116500 -5.41991100
2.02565600
H -0.75303700 -3.47719100
0.32060600
H -1.80968000 -5.48758200
1.35399200

PC*

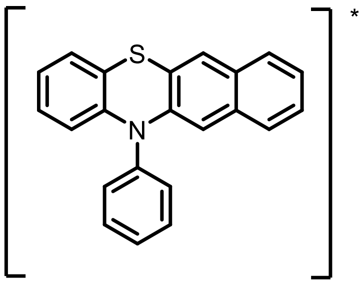

E (UωB97XD sbs) = -1299.185268
E (UωB97XD) = -1300.26623
E (UB3LYP) =-1300.65089
E (UPBE1PBE) = -1299.44096
E (UM062X) = -1300.20237
G_{corr} = 0.256013

0 3
C -7.05545800 -1.05070600
3.29681500
C -5.91116700 -1.28492800
2.53778700
C -5.88858300 -0.95845400
1.17911000
C -6.99250600 -0.30247600
0.61572800
C -8.13094600 -0.04863700
1.38190200
C -8.17010400 -0.44641600
2.71623300
C -5.16910900 0.65834700 -
1.09146300
C -4.29009500 -0.10631800 -
0.38595000
C -2.88415800 0.27322500 -
0.33184800
H -2.20747400 -0.31463100
0.29041000
C -2.39983700 1.38346300 -
1.06460400
C -3.33628000 2.17098600 -
1.84422600
C -4.71441200 1.80925600 -
1.83096500
H -7.06965300 -1.33037400
4.35218200
H -5.02623300 -1.73307300
2.99254800
H -8.98096200 0.47033700
0.93321100
H -9.06578200 -0.25653900
3.31115800
H -5.43491400 2.40514400 -
2.39464600
C -2.85806000 3.26227200 -
2.56646700
H -3.55848600 3.86362300 -
3.15101000
C -1.04977400 1.75261500 -
1.06205300
H -0.33979800 1.16754800 -
0.47261700
C -1.46821300 3.61423600 -
2.55208700
H -1.12959400 4.47616400 -
3.13059900
C -0.58568200 2.87605800 -
1.81531100

H 0.47491500 3.13469000 -
1.79124600
S -6.89117000 0.24380400 -
1.07312600
N -4.75077700 -1.21148500
0.36286900
C -4.30440600 -2.51151000
0.05873500
C -4.72719300 -3.63688800
0.78847900
C -3.43081600 -2.71797800 -
1.02568600
C -4.26278600 -4.90786600
0.46205500
H -5.43524200 -3.53180600
1.60869800
C -2.96416900 -3.99335700 -
1.33091700
H -3.12747300 -1.87914000 -
1.65266900
C -3.36846800 -5.10107200 -
0.58923900
H -4.61323900 -5.76188400
1.04676600
H -2.28469500 -4.11749200 -
2.17759500
H -3.00477400 -6.10049900 -
0.83545400

Lutidine

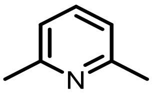

E (UωB97XD sbs) = -326.5894324
E (UωB97XD) = -326.94114
E (UB3LYP) =-327.070219
E (UPBE1PBE) = -326.671473
E (UM062X) = -326.904685
G_{corr} = 0.1117

0 1
C -2.62456100 -1.01072200
0.04248000
C -1.22773100 -1.00227900 -
0.04907500
C -0.57044000 0.22138900 -
0.12615300
C -1.31948200 1.39352900 -
0.10979400
C -2.71313600 1.29964000 -
0.01595200
N -3.33592000 0.12004300
0.05754800
H 0.51918200 0.26130800 -
0.19838400
H -0.67046400 -1.94110200 -
0.05916300
H -0.83574200 2.37051600 -
0.16879100
C -3.58900900 2.52174500
0.00629300
H -4.19733700 2.53506800
0.92347000
H -3.00377600 3.45008200 -
0.04229700
H -4.28888100 2.50161000 -
0.84318500
C -3.40446100 -2.29356400
0.12812000
H -4.00807800 -2.30915000
1.04838500
H -4.10549800 -2.36769000 -
0.71734600
H -2.74999700 -3.17581700
0.12073800

Lutidine-H

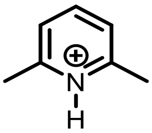

E (UωB97XD sbs) = -327.0258557
E (UωB97XD) = -327.376893
E (UB3LYP) =-327.503642
E (UPBE1PBE) = -327.104805
E (UM062X) = -327.331465
G_{corr} = 0.127396

1 1
C -2.57724900 -1.05491800
0.23262200
C -1.24902300 -1.01355800 -
0.16549400
C -0.64160400 0.21862500 -
0.39915000
C -1.35731100 1.40329400 -
0.23591600
C -2.68401800 1.34893000
0.16622400
N -3.22246500 0.12556400
0.37594900
H 0.40277200 0.25639200 -
0.71494600
H -0.70197000 -1.94795100 -
0.29037800
H -0.89296800 2.37333500 -
0.41364600
C -3.55334700 2.53792800
0.40172300
H -3.78093100 2.63555700
1.47513200
H -3.05023800 3.45340200
0.07147600
H -4.50533100 2.44301400 -
0.14176400
C -3.34790200 -2.30328900
0.50068600
H -3.80378400 -2.27628400
1.50182200
H -4.15425800 -2.42326200 -
0.23965500
H -2.69085400 -3.17829300
0.44214100
H -4.20012500 0.09121900
0.66840700

SO_2

E (UωB97XD sbs) = -548.2932125
E (UωB97XD) = -548.657163
E (UB3LYP) =-548.72779
E (UPBE1PBE) = -548.41676
E (UM062X) = -548.630663
G_{corr} = -0.017648

0 1
S -1.85196000 -0.17480800
0.00000000
O -0.43459000 0.13462900
0.00000000
O -2.24780100 -1.57050800
0.00000000

1a_r

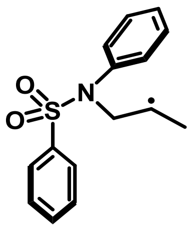

E (UωB97XD sbs) = -1183.561903
E (UwB97XD) = -1184.60903
E (UB3LYP) =-1184.93086
E (UPBE1PBE) = -1183.86215
E (UM062X) = -1184.5239
G_{corr} = 0.231805
$G_{corr(Goodvibes)}$ = 0.23623515

0 2
N -1.86290700 -1.63687000 -
0.92524500
C -1.02705800 -0.72124800 -
1.71209000
H -1.71985200 -0.05448600 -
2.25442100
H -0.40954000 -0.06665700 -
1.06372000
C -0.16292200 -1.46956600 -
2.66570700
H -0.61483900 -2.31767700 -
3.18877000
C 1.14678800 -0.92455400 -
3.10404700
H 1.75783400 -0.59378500 -
2.24541400
H 1.72645700 -1.66821600 -
3.66963000
H 1.03167600 -0.03746900 -
3.76005200
C -1.21874400 -2.69510800 -
0.20980000
C -1.78843400 -3.97124200 -
0.21504000
C -0.02943900 -2.46800600
0.49165900
C -1.17866700 -5.00735600
0.48741500
H -2.71402000 -4.13343100 -
0.76734400
C 0.58471800 -3.51309700
1.17828000
H 0.41619000 -1.47168900
0.50572400
C 0.01009700 -4.78337500
1.18220500
H -1.63167000 -6.00119200
0.48215400
H 1.51558300 -3.32953200
1.71951800
H 0.49103700 -5.60074300
1.72406200
S -3.23954700 -0.98257600 -
0.21717600
C -2.62334500 -0.10511700
1.20655600
C -2.27066700 1.23844700
1.07872300
C -2.44201300 -0.79811000
2.40411200
C -1.71536400 1.89645200
2.17430000
H -2.44533500 1.76050000
0.13633200
C -1.88409600 -0.12845100
3.49054900
H -2.74061200 -1.84486100
2.48028100
C -1.51955700 1.21340000
3.37497300
H -1.44073300 2.95000100
2.09085800
H -1.73753300 -0.65798300
4.43406600
H -1.08431200 1.73373400
4.23103200
O -3.76919100 -0.01105400 -
1.16033600
O -4.05648300 -2.08568600
0.25521700

1a_react

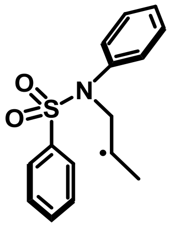

E (UωB97XD sbs) = -1183.561357
E (UωB97XD) = -1184.60751
E (UB3LYP) =-1184.92977
E (UPBE1PBE) = -1183.8602
E (UM062X) = -1184.52311
G_{corr} = 0.233891

0 2
N -0.51179000 -1.65093000
0.10712600
C 0.86132500 -1.44092600 -
0.39289600
H 1.55735500 -1.66438800
0.42875800
H 1.00619600 -2.22991000 -
1.15153600
C 1.15425100 -0.08808200 -
0.95407700
H 0.54333300 0.27852700 -
1.78482200
C 2.32107500 0.70734000 -
0.49216100
H 3.28050300 0.29748600 -
0.86775500
H 2.26489700 1.75194700 -
0.83198900
H 2.39109900 0.69832200
0.60953000

C	-1.49113100	-2.29200100	-0.70655100
C	-1.68209600	-1.90105800	-2.03740700
C	-2.26268200	-3.33201000	-0.17585700
C	-2.62945400	-2.54806300	-2.82815800
H	-1.09047100	-1.08485200	-2.45451300
C	-3.22430100	-3.95793200	-0.96478000
H	-2.10464000	-3.63187900	0.85972500
C	-3.40863200	-3.57247500	-2.29267600
H	-2.76640800	-2.23868500	-3.86673600
H	-3.82577100	-4.76449100	-0.53960500
H	-4.15759200	-4.07262800	-2.91062200
S	-1.02459400	-0.69171800	1.37670200
C	-1.70698300	0.77298900	0.62100500
C	-0.94307000	1.93771400	0.57537800
C	-2.98512400	0.71414000	0.06373500
C	-1.46640000	3.06300100	-0.05757700
H	0.04453700	1.95592900	1.03814100
C	-3.49289900	1.84379600	-0.57396800
H	-3.57468800	-0.20159300	0.13406800
C	-2.73494200	3.01365200	-0.63637400
H	-0.88120600	3.98420400	-0.09475900
H	-4.49015100	1.81112400	-1.01737900
H	-3.14028300	3.89773400	-1.13357400
O	0.17549900	-0.31518800	2.10777800
O	-2.11970500	-1.37706500	2.04203300

TS1_1a

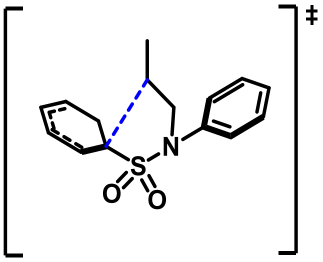

E (UωB97XD sbs) = -1183.542668
E (UωB97XD) = -1184.58689
E (UB3LYP) =-1184.90971
E (UPBE1PBE) = -1183.84447
E (UM062X) = -1184.50131
G_{corr} = 0.236315
$G_{corr(Goodvibes)}$ = 0.23847586
0 2

N	-0.87872500	-1.69809000	0.26900500
C	-1.08934400	-1.76801000	1.71075600
H	-2.17847800	-1.78807600	1.90361000
H	-0.66580300	-2.70111400	2.12550800
C	-0.45687600	-0.59125400	2.38238800
H	-0.84108600	0.38413200	2.06309400
C	-0.02348500	-0.70340400	3.80113800
H	0.58754300	0.16248900	4.09695500
H	-0.89694400	-0.73082100	4.47905000
H	0.55943300	-1.62108400	3.97776400
C	-1.60578800	-2.50821900	-0.62757800
C	-1.90721900	-2.06162500	-1.92266500
C	-2.06889600	-3.76373600	-0.21285300
C	-2.62678700	-2.87634900	-2.79037900
H	-1.58196300	-1.07338600	-2.24964700
C	-2.81052000	-4.56007700	-1.08377300
H	-1.84614900	-4.12837800	0.79091300
C	-3.08671400	-4.12727100	-2.37838900
H	-2.84449500	-2.51694400	-3.79866800
H	-3.16619500	-5.53529400	-0.74311200
H	-3.66116900	-4.75685500	-3.06089500
S	0.31399900	-0.64881700	-0.29013000
C	1.37796000	-0.60641400	1.15408100
C	1.97467200	0.63895300	1.47872200
C	2.05051000	-1.81058800	1.49098400
C	3.09081100	0.66353000	2.29867100
H	1.51871900	1.55964000	1.11067800
C	3.16199600	-1.75995800	2.31601900
H	1.67333700	-2.76658600	1.12095900
C	3.68004500	-0.52892900	2.73888000
H	3.52444600	1.62427500	2.58484600
H	3.65379500	-2.68903000	2.61279600
H	4.56328100	-0.49898100	3.37989600
O	-0.23333200	0.68651500	-0.49799100

O 0.99342300 -1.29810600 -
1.39960000

INT1_1a

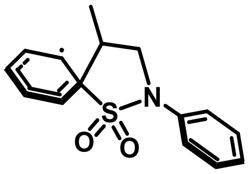

E (UωB97XD sbs) = -1183.574033
E (UωB97XD) = -1184.61453
E (UB3LYP) =-1184.93209
E (UPBE1PBE) = -1183.86988
E (UM062X) = -1184.52643
G_{corr} = 0.238768

0 2
N -0.92860500 -1.70857300
0.07525600
C 0.31530200 -1.08946000 -
0.36019300
H 0.97192300 -0.93343700
0.51343500
H 0.84660400 -1.76404500 -
1.04280200
C -0.03220900 0.24863700 -
1.02661200
H -0.61814500 0.01055700 -
1.93018800
C 1.18234800 1.06643200 -
1.43825900
H 1.76585900 0.52516900 -
2.19878400
H 0.87847200 2.03237400 -
1.86794200
H 1.84665300 1.27047500 -
0.58515900
C -1.58118000 -2.72194400 -
0.67042500
C -1.33159700 -2.92799800 -
2.03252100
C -2.49168800 -3.55515600 -
0.00774000
C -1.97791900 -3.95878100 -
2.71332600
H -0.63585400 -2.28506900 -
2.57479400
C -3.15161700 -4.56403500 -
0.70087800
H -2.67308500 -3.40643600
1.05808700
C -2.89428400 -4.77622600 -
2.05564800
H -1.76859300 -4.11255900 -
3.77445500
H -3.86358800 -5.20084900 -
0.17114700
H -3.40459600 -5.57688900 -
2.59500400
S -1.85482500 -0.50147900
0.84030000
C -0.99552700 1.00988600 -
0.07078600
C -0.32364700 1.79634000
0.99528000
C -2.07089400 1.75165100 -
0.77379700
C -0.72258400 3.06213900
1.32372800
H 0.50097300 1.32695700
1.53542400
C -2.44071900 3.01780800 -
0.41138400
H -2.58033600 1.23956200 -
1.59337900
C -1.78921100 3.69505200
0.64456800
H -0.20311100 3.59471100
2.12365100
H -3.24772600 3.51504800 -
0.95433400
H -2.09759400 4.70377600
0.92450500
O -1.49024800 -0.46615300
2.24920400
O -3.25369800 -0.64544900
0.46432200

TS2_1a

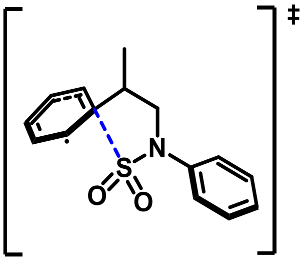

E (UωB97XD sbs) = -1183.573172
E (UωB97XD) = -1184.61191
E (UB3LYP) =-1184.93356
E (UPBE1PBE) = -1183.86829
E (UM062X) = -1184.52528
G_{corr} = 0.238861
$G_{corr(GoodVibes)}$ = 0.23847586

0 2
N -0.92303900 -1.69934800
0.11178500
C 0.32016000 -1.08326800 -
0.32760400
H 0.95808300 -0.89481400
0.55291000
H 0.86691500 -1.78275900 -
0.97209700
C 0.01071600 0.23378800 -
1.05909300
H -0.58348500 -0.02764000 -
1.95002200
C 1.27015100 0.95915200 -
1.51796900
H 1.83180800 0.33561300 -
2.23057600
H 1.02039800 1.90587000 -
2.01884200
H 1.93805600 1.18926900 -
0.67407400
C -1.58803300 -2.68881900 -
0.65223400
C -1.30918200 -2.90722000 -
2.00751400
C -2.53678400 -3.49882200 -
0.01228200

C -1.96449500 -3.92316000 -
2.70234500
H -0.58039900 -2.28918200 -
2.53453400
C -3.20314600 -4.49269100 -
0.71966100
H -2.74351200 -3.34410400
1.04781000
C -2.91785400 -4.71527800 -
2.06746900
H -1.73046100 -4.08579300 -
3.75698900
H -3.94332700 -5.11036700 -
0.20614500
H -3.43427400 -5.50446800 -
2.61777200
S -1.83121200 -0.48067700
0.90122900
C -0.89652300 1.11248600 -
0.17446700
C -0.26135200 1.87844700
0.89323600
C -2.01134100 1.77618800 -
0.83866800
C -0.80347400 3.05529000
1.34909400
H 0.63497900 1.47261000
1.36707500
C -2.53563100 2.95388800 -
0.36003200
H -2.45888000 1.28630100 -
1.70687200
C -1.95666800 3.59902000
0.74911300
H -0.32666700 3.58133100
2.17902800
H -3.39998100 3.40016300 -
0.85656700
H -2.38099200 4.53229800
1.12404100
O -1.39814900 -0.42539600
2.29370500
O -3.24734700 -0.64587100
0.59300400

INT2_1a

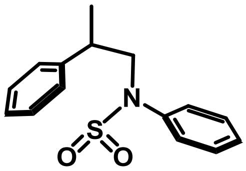

E (UωB97XD sbs) = -1183.602897
E (UωB97XD) = -1184.64092
E (UB3LYP) =-1184.96387
E (UPBE1PBE) = -1183.89095
E (UM062X) = -1184.5541
G_{corr} = 0.237304

0 2
N -1.09883700 -1.57137600
0.68182800
C 0.25767400 -1.11288500
0.39703100
H 0.65059400 -0.65482400
1.31441300
H 0.87369900 -2.00648300
0.20611900
C 0.37192700 -0.15248400 -
0.79710400
H 0.05182900 -0.70661200 -
1.69464600
C 1.83368300 0.25667900 -
0.98983700
H 2.47572500 -0.62625300 -
1.13570600
H 1.94433000 0.90582300 -
1.87039000
H 2.21325100 0.80867700 -
0.11546200
C -1.81581700 -2.28263900 -
0.32473400
C -2.96146200 -1.75601200 -
0.92833700
C -1.33463700 -3.53433300 -
0.72187400
C -3.62665800 -2.48632200 -
1.91031400
H -3.33116300 -0.77190100 -
0.63478800
C -1.99153600 -4.24710300 -
1.72131200
H -0.44976900 -3.94991100 -
0.23567500
C -3.14166500 -3.72844800 -
2.31562500
H -4.52629700 -2.07114500 -
2.36963900
H -1.60824600 -5.22328600 -
2.02628300
H -3.66051100 -4.29341300 -
3.09281500
S -1.99241200 -0.77649100
1.89044300
C -0.54865900 1.04564300 -
0.65454900
C -0.45435400 1.90932900
0.44505100
C -1.51897800 1.31198300 -
1.62672000
C -1.31320100 2.99949800
0.57273800
H 0.28817600 1.72342100
1.22496300
C -2.37787000 2.40426400 -
1.50470900
H -1.60691700 0.64716300 -
2.49046300
C -2.27886700 3.25065000 -
0.40178600
H -1.22876200 3.65534600
1.44225600
H -3.12906600 2.59278800 -
2.27540000
H -2.95309500 4.10413600 -
0.30062500
O -1.02311900 -0.33148500
2.90094900
O -3.10393500 -1.65995300
2.26120400

TS3_1a

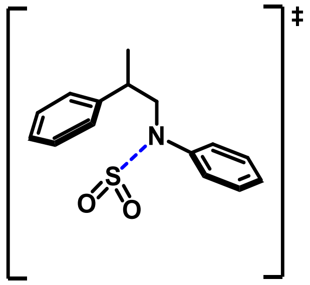

E (UωB97XD sbs) = -1183.597739
E (UωB97XD) = -1184.63129
E (UB3LYP) =-1184.96045
E (UPBE1PBE) = -1183.88466
E (UM062X) = -1184.54565
G_{corr} = 0.236109

0 2
N -1.28894600 -1.53204100
0.62183700
C 0.12035300 -1.20702600
0.48831300
H 0.43918800 -0.78859400
1.45174000
H 0.66629900 -2.15514200
0.35843500
C 0.49988700 -0.22788100 -
0.65364100
H 0.31590400 -0.73865500 -
1.61065500
C 1.99502000 0.07487600 -
0.56674400
H 2.58593400 -0.85007800 -
0.65599800
H 2.30344700 0.75395900 -
1.37468700
H 2.26306400 0.54473000
0.39196900
C -1.95329600 -2.22902700 -
0.38605100
C -3.36253600 -2.34967300 -
0.39856000
C -1.22408200 -2.88632600 -
1.40574900
C -3.99899000 -3.07435500 -
1.39734600
H -3.95491600 -1.86980400
0.37680500
C -1.87690800 -3.62332100 -
2.38456600
H -0.13631800 -2.85182100 -
1.42420000
C -3.26861900 -3.71639300 -
2.39695000
H -5.08893400 -3.14080600 -
1.38657600
H -1.28560500 -4.13142300 -
3.14933700
H -3.77760100 -4.28813800 -
3.17534100
S -2.19419700 -0.31158900
1.71284100
C -0.38181000 1.00556700 -
0.64694500
C -0.18583000 2.05015000
0.26458300
C -1.46342400 1.08871700 -
1.53266300
C -1.04661600 3.14563000
0.28839100
H 0.63700700 2.00387600
0.98088000
C -2.32823100 2.18195000 -
1.51006100
H -1.63292600 0.27912500 -
2.24858800
C -2.12156500 3.21486700 -
0.59748900
H -0.88005800 3.94812000
1.01057700
H -3.16730300 2.22511500 -
2.20831800
H -2.79761800 4.07264600 -
0.57493200
O -1.16692600 0.08743300
2.68430400
O -3.35779400 -1.03262800
2.25292600

2a_r

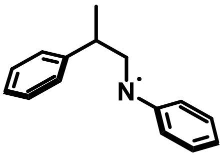

E (UωB97XD sbs) = - 635.2966656
E (UωB97XD) = -635.971773
E (UB3LYP) =-636.225705
E (UPBE1PBE) = -635.453579
E (UM062X) = -635.906247
G_{corr} = 0.229252

0 2
N 0.80625100 -1.41018700 -
0.87912100
C 0.15657500 -0.43478100 -
1.53431700
C -1.19590500 -0.48534800 -
2.00163600
C 0.89589600 0.76146900 -
1.79396300
C -1.74828500 0.58954200 -
2.67810100
H -1.80211100 -1.37613400 -
1.83157800
C 0.32752500 1.82745400 -
2.46681000
H 1.92724500 0.79532300 -
1.43691900
C -0.99860700 1.74965700 -
2.91465100
H -2.78089300 0.53166600 -
3.03048100
H 0.91290300 2.73108800 -
2.65132000
H -1.44793200 2.59060200 -
3.44737500
C 0.13755100 -2.64852800 -
0.59885300
H 0.60998600 -3.09411300
0.29285800
H -0.93647600 -2.52897700 -
0.36775100
C 0.28052800 -3.65255800 -
1.77260300

H -0.15940800 -3.16775200 -
2.65997600
C 1.74678600 -3.95722300 -
2.08188700
H 2.28771700 -3.03472200 -
2.33723000
H 2.25645300 -4.40863700 -
1.21608300
H 1.83051400 -4.65703800 -
2.92692600
C -0.53903300 -4.89556500 -
1.48658300
C -1.70738100 -5.15866200 -
2.21080800
C -0.16855900 -5.79431800 -
0.47720100
C -2.48347700 -6.28585800 -
1.94073300
H -2.01227500 -4.46945700 -
3.00378800
C -0.94155800 -6.92078500 -
0.20273100
H 0.73913100 -5.61405400
0.10491100
C -2.10281000 -7.17141600 -
0.93429800
H -3.38991100 -6.47293400 -
2.52153700
H -0.63424300 -7.60903000
0.58836800
H -2.70785800 -8.05541100 -
0.72006700

2a

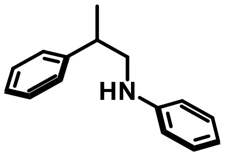

E (UωB97XD sbs) = -635.954787
E (UωB97XD) = -636.628688
E (UB3LYP) =-636.878973
E (UPBE1PBE) = -636.105633
E (UM062X) = -636.562055
G_{corr} = 0.24264

N 1.00721500 -1.55746500 -
1.18424600
C 0.41165100 -0.55020600 -
1.91143700
C -0.94848900 -0.58328100 -
2.27718300
C 1.16933200 0.57794600 -
2.29057900
C -1.51606300 0.47506300 -
2.98455100
H -1.57075800 -1.43808800 -
2.00792000
C 0.58936100 1.62430800 -
2.99623600
H 2.22785500 0.62293400 -
2.01866200
C -0.76080900 1.58657200 -
3.35240000
H -2.57435600 0.42209800 -
3.25318900
H 1.20450700 2.48418500 -
3.27378400
H -1.21374100 2.40943500 -
3.90867800
C 0.41910900 -2.85119600 -
0.95386900
H 0.99290400 -3.34335500 -
0.15203200
H -0.60290600 -2.72876400 -
0.56000700
C 0.38134200 -3.77986800 -
2.19015500
H -0.18049600 -3.24942800 -
2.97631000
C 1.78659000 -4.05047200 -
2.72641100
H 2.26898200 -3.11151600 -
3.03762000
H 2.43137000 -4.52169500 -
1.96677400
H 1.75626100 -4.71616500 -
3.60178500
C -0.39248900 -5.04037300 -
1.85786100
C -1.70103700 -5.21265500 -
2.32500500
C 0.15416800 -6.03968300 -
1.04135200
C -2.44151300 -6.34705200 -
1.99366900
H -2.14552800 -4.44472500 -
2.96489800
C -0.58215900 -7.17489600 -
0.70658300
H 1.17358900 -5.93490300 -
0.66044900
C -1.88379100 -7.33342700 -
1.18204200
H -3.45939800 -6.46083300 -
2.37434100
H -0.13527800 -7.94238600 -
0.06993300
H -2.46046900 -8.22394900 -
0.92158700
H 2.01094400 -1.49158800 -
1.08430800

1a'_react

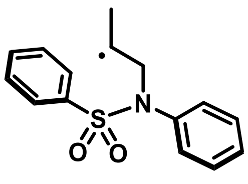

E (UωB97XD sbs) = -1183.561063
E (UωB97XD) = -1184.60718
E (UB3LYP) =-1184.92899
E (UPBE1PBE) = -1183.85918
E (UM062X) = -1184.52229
G_{corr} = 0.233602

N 0.57282300 -0.35487200
0.58534700
C 1.76823700 0.35605500
0.07256500
H 2.47961800 0.34203700
0.91545000

H 2.20631100 -0.24730600 -
0.73464900
C 1.54495200 1.75645300 -
0.39652900
H 1.60822400 1.95276000 -
1.46975600
C 1.16716800 2.85704800
0.52836400
H 1.83865400 2.90108700
1.40577400
H 1.19863200 3.83538600
0.02832800
H 0.14037000 2.73252500
0.92648400
C 0.45708100 -0.71643200
1.95959100
C 0.37020600 -2.06442400
2.32367800
C 0.45306400 0.26883100
2.95300800
C 0.27001000 -2.41794600
3.66669700
H 0.37151500 -2.82528600
1.54311900
C 0.37274900 -0.09158000
4.29681900
H 0.51128900 1.31959100
2.66656300
C 0.27558600 -1.43498000
4.65657600
H 0.19658100 -3.47244900
3.94171600
H 0.37606000 0.68454600
5.06545200
H 0.20523200 -1.71689200
5.70948300
S -0.71741000 -0.57527600 -
0.44181000
C -1.70622400 0.89891200 -
0.26372500
C -2.45569300 1.07401600
0.90071700
C -1.67993500 1.86412600 -
1.26718300
C -3.16977700 2.25801200
1.06836600
H -2.48171000 0.29227800
1.66228700
C -2.40535900 3.04090800 -
1.09090000
H -1.09450000 1.68707500 -
2.17045700
C -3.14156900 3.24034400
0.07667500
H -3.75741600 2.41105900
1.97587700
H -2.39539000 3.80498400 -
1.87099800
H -3.70510500 4.16600400
0.21298700
O -1.50798900 -1.68498000
0.06462300
O -0.17660800 -0.59195800 -
1.79215400

TS1_1a’

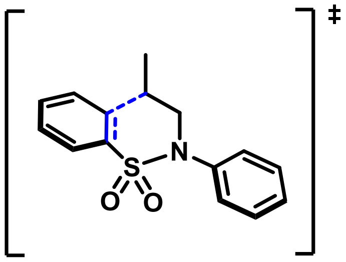

E (UωB97XD sbs) = -1183.544101
E (UωB97XD) = -1184.58954
E (UB3LYP) =-1184.91316
E (UPBE1PBE) = -1183.84691
E (UM062X) = -1184.50358
G_{corr} = 0.237843
$G_{corr(Goodvibes)}$ = 0.23994568

0 2
N -1.50202100 -2.47092800 -
2.10499600
C -0.58344600 -1.62141400 -
2.86660200
H 0.38505200 -2.14270900 -
2.93253100
H -0.97631500 -1.58057100 -
3.89855400
C -0.37016900 -0.21680500 -
2.35176700
H -1.28067400 0.33900700 -
2.10074300
C 0.70342500 0.57457000 -
3.02583000
H 1.66287900 0.03154700 -
3.03565700
H 0.43957600 0.78096300 -
4.08011800
H 0.85786500 1.54713700 -
2.53635800
C -2.88455600 -2.53393400 -
2.42713200
C -3.56528100 -3.75474200 -
2.32267100
C -3.57171600 -1.40934100 -
2.90117000
C -4.91297100 -3.83567300 -
2.65793600
H -3.02905100 -4.63758400 -
1.97657200
C -4.91530100 -1.50557700 -
3.26019700
H -3.06061900 -0.45079100 -
2.99469300
C -5.59486900 -2.71443400 -
3.13136800
H -5.43050000 -4.79297200 -
2.56367700
H -5.43459100 -0.62000000 -
3.63359800
H -6.64984200 -2.78549500 -
3.40453600
S -0.95440400 -2.87543000 -
0.56247000
C -0.80723300 -1.27848400
0.17270200
C 0.27051000 -0.47240800 -
0.28760000
C -1.83961100 -0.76599400
0.94362400

C 0.36542700 0.83726800
0.25511300
H 1.17032400 -0.96550500 -
0.66383000
C -1.77129900 0.56183200
1.37405000
H -2.68152500 -1.40610100
1.21440000
C -0.65927500 1.34866200
1.03522800
H 1.24264900 1.44691300
0.02929800
H -2.56788700 0.97361700
1.99630100
H -0.58942300 2.37111100
1.41423200
O -1.99039300 -3.63741800
0.11051200
O 0.38282300 -3.41985500 -
0.73344300

INT1_1a'

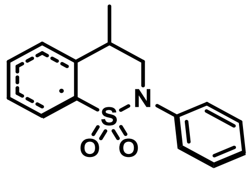

E (UωB97XD sbs) = -1183.578989
E (UωB97XD) = -1184.62144
E (UB3LYP) =-1184.93646
E (UPBE1PBE) = -1183.87614
E (UM062X) = -1184.53309
G_{corr} = 0.240578

0 2
N -1.24616600 -1.65711100 -
1.38704400
C -0.15051000 -1.09847100 -
2.19230500
H 0.62668400 -1.86432300 -
2.36764500
H -0.57815900 -0.83563300 -
3.16936300
C 0.45557000 0.15212400 -
1.54632500
H 1.29917600 0.45957900 -
2.18642800
C -0.55601000 1.29132900 -
1.47094500
H -1.41898300 1.01650100 -
0.84526900
H -0.10555700 2.19986700 -
1.04512800
H -0.93114000 1.54213600 -
2.47514200
C -2.33596900 -2.29716800 -
2.04954600
C -2.44552300 -3.68978400 -
2.11011000
C -3.29860800 -1.49553500 -
2.66991700
C -3.52475200 -4.27080000 -
2.77285100
H -1.68657800 -4.31389000 -
1.63479900
C -4.35909300 -2.08313200 -
3.35453700
H -3.20828400 -0.40918900 -
2.60274500
C -4.47875500 -3.47242800 -
3.40185100
H -3.61174100 -5.35904400 -
2.80635300
H -5.10639900 -1.45101900 -
3.83948900
H -5.31796000 -3.93279900 -
3.92782400
S -0.76124400 -2.26364300
0.10544000
C 0.00627200 -0.81294100
0.73769400
C 1.05646600 -0.20317100 -
0.14699500
C -0.37143100 -0.31302800
1.95086500
C 1.71104000 0.96974800
0.51709000
H 1.83085800 -0.97317800 -
0.34056600
C 0.28076000 0.82516000
2.47654300
H -1.17158900 -0.80357600
2.50952000
C 1.33037100 1.43810700
1.74024100
H 2.51956000 1.45979800 -
0.03262700
H -0.01640000 1.22153700
3.44847200
H 1.84249100 2.30325200
2.16829000
O -1.95046300 -2.60469500
0.86665800
O 0.28096700 -3.27072600 -
0.08555700

INT2_1a'

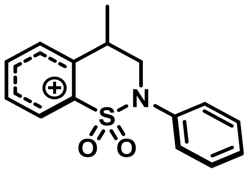

E (UωB97XD sbs) = -1183.366364
E (UωB97XD) = -1184.41282
E (UB3LYP) =-1184.7308
E (UPBE1PBE) = -1183.66793
E (UM062X) = -1184.32086
G_{corr} = 0.244339

1 1
N -1.03181800 -1.86206600 -
1.41003400
C 0.05171900 -1.25611300 -
2.18787700
H 0.89516700 -1.95927200 -
2.28383400
H -0.34121300 -1.06956200 -
3.19549300
C 0.51496400 0.07510400 -
1.58365900
H 1.39665100 0.40547100 -
2.15284400
C -0.56390400 1.15124400 -
1.63443600

H -1.44121500 0.88082600 -
1.02697700
H -0.18803200 2.12314500 -
1.28408800
H -0.90234600 1.28523100 -
2.67208200
C -2.23913700 -2.33635500 -
2.02151000
C -2.43909000 -3.70425700 -
2.21710200
C -3.19624300 -1.41182400 -
2.44172600
C -3.61192900 -4.14609200 -
2.82417800
H -1.67909500 -4.41911100 -
1.89490400
C -4.35642300 -1.86057100 -
3.06794800
H -3.02880400 -0.34631900 -
2.27511800
C -4.56771700 -3.22669000 -
3.25424400
H -3.77371700 -5.21583700 -
2.97025900
H -5.10464800 -1.13930900 -
3.40232400
H -5.48210100 -3.57644300 -
3.73769700
S -0.57519100 -2.43347100
0.07401800
C 0.01502800 -0.86902100
0.71635800
C 1.02816800 -0.17012600 -
0.09441600
C -0.46971500 -0.37309300
1.89211200
C 1.54663300 1.06234600
0.50578800
H 1.89061600 -0.86399300 -
0.21632800
C 0.06888300 0.83801600
2.37584000
H -1.24110300 -0.90399600
2.45310100
C 1.07591700 1.54837000
1.69749200
H 2.32220600 1.60145500 -
0.04577900
H -0.30575200 1.23065600
3.32503300
H 1.47098900 2.47091200
2.12367200
O -1.74021300 -2.86072900
0.81208500
O 0.61136500 -3.26467100 -
0.02119400

2a’

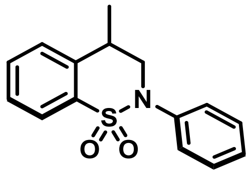

E (UωB97XD sbs) = -1183.024378
E (UωB97XD) = -1184.06808
E (UB3LYP) =-1184.38352
E (UPBE1PBE) = -1183.32484
E (UM062X) = -1183.98849
G_{corr} = 0.232808

0 1
N -1.28906200 -1.62478700 -
1.35727500
C -0.10682500 -1.13135500 -
2.06931300
H 0.72730300 -1.84813600 -
1.96788900
H -0.36202300 -1.07883900 -
3.13576800
C 0.29226900 0.25041200 -
1.55462600
H 1.25281800 0.50900500 -
2.02870200
C -0.74378400 1.31263200 -
1.93890500
H -1.71943600 1.08140600 -
1.48503500
H -0.43887200 2.31010800 -
1.59060200
H -0.86699700 1.35867300 -
3.03213900
C -2.34702100 -2.26655100 -
2.06649600
C -2.58749600 -3.63900600 -
1.94705900
C -3.14246800 -1.49307700 -
2.91743900
C -3.63333200 -4.22238100 -
2.65936600
H -1.95646800 -4.24707200 -
1.29696000
C -4.16597700 -2.08951600 -
3.64892600
H -2.95512000 -0.42027500 -
2.99515400
C -4.42004600 -3.45473300 -
3.51626300
H -3.82306500 -5.29259100 -
2.55152900
H -4.78067600 -1.47839900 -
4.31363000
H -5.23180900 -3.91941100 -
4.07999700
S -0.96849400 -2.11340300
0.20794600
C -0.07349700 -0.69102900
0.80427400
C 0.50719900 0.25310200 -
0.05175300
C 0.07204400 -0.62387700
2.19228500
C 1.25559400 1.27701400
0.54315300
C 0.81897600 0.40394400
2.75305200
H -0.40517200 -1.37403400
2.82645700
C 1.41650500 1.35294400
1.92229400
H 1.72183600 2.02893600 -
0.09908900
H 0.93537200 0.46408400
3.83671400
H 2.01127000 2.16046800
2.35455600
O -2.22996600 -2.24171200
0.91705700

O -0.04529800 -3.24469800
0.22190300

1a”_react

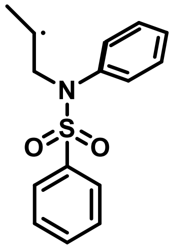

E (UωB97XD sbs) = -1183.561929
E (UωB97XD) = -1184.60904
E (UB3LYP) =-1184.93088
E (UPBE1PBE) = -1183.86217
E (UM062X) = -1184.52396
G_{corr} = 0.232521

0 2
N -2.19338900 -1.87567700
0.55260500
C -2.40829200 -1.82892900
2.00467800
H -2.84735600 -0.83977500
2.22245800
H -3.15496200 -2.57965900
2.33478000
C -1.12893300 -2.01033700
2.74371400
H -0.24752700 -1.49535000
2.34999100
C -1.10420300 -2.61759100
4.09840100
H -1.68542000 -3.55610900
4.13429100
H -0.07773900 -2.83825100
4.42497800
H -1.55004900 -1.94977800
4.86381200
C -1.50615900 -3.00448800
0.00536300
C -1.77209200 -4.29981600
0.46315500
C -0.55400400 -2.80508200 -
0.99815600
C -1.08685200 -5.38468900 -
0.07961600
H -2.51811200 -4.46284700
1.24311900
C 0.11474100 -3.89531400 -
1.54829200
H -0.35512500 -1.79017200 -
1.34227800
C -0.14615300 -5.18658700 -
1.08932200
H -1.29594100 -6.39248100
0.28611300
H 0.85445200 -3.73213700 -
2.33518000
H 0.38723800 -6.03864500 -
1.51651400
S -3.39038600 -1.18611600 -
0.40502700
C -4.70820200 -2.38600200 -
0.39074800
C -5.70552100 -2.28959700
0.57961600
C -4.68131800 -3.43532800 -
1.31042600
C -6.69077500 -3.27347000
0.63343000
H -5.71326600 -1.44439400
1.27008100
C -5.67119700 -4.41293100 -
1.24369000
H -3.89809200 -3.47716900 -
2.06912100
C -6.67081600 -4.33347200 -
0.27336000
H -7.48157800 -3.20720800
1.38351600
H -5.66344500 -5.23897200 -
1.95768600
H -7.44583200 -5.10181200 -
0.22780200
O -3.84776900 0.00265600
0.29614300
O -2.87491800 -1.10177500 -
1.75940900

TS1_1a”_b

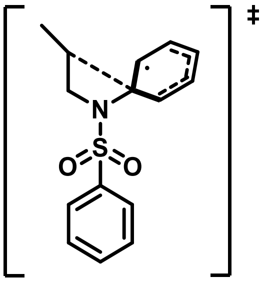

E (UωB97XD sbs) = -1183.531392
E (UωB97XD) = -1184.57891
E (UB3LYP) =-1184.8999
E (UPBE1PBE) = -1183.83371
E (UM062X) = -1184.48969
G_{corr} = 0.234849

0 2
N 2.56717000 0.99031100 -
1.64035500
C 1.89724400 2.15897400 -
2.18912700
H 2.56735400 2.78652500 -
2.79481200
H 1.42565700 2.79022000 -
1.41205200
C 0.87327000 1.35957200 -
2.96726800
H 1.22629800 1.01749500 -
3.94655700
C -0.58903900 1.63545300 -
2.87188200
H -0.86623100 2.50326200 -
3.49803600
H -1.17341600 0.77408600 -
3.23034300
H -0.90047100 1.85870100 -
1.84050500
C 1.58865400 -0.07960000 -
1.74915100

C 1.93658000 -1.23878300 -
2.51432200
C 0.60265500 -0.24801300 -
0.71825900
C 1.18309100 -2.39376000 -
2.42182600
H 2.78725400 -1.16680900 -
3.19446900
C -0.13924100 -1.41162500 -
0.64142100
H 0.41099200 0.57767800 -
0.02921100
C 0.12555200 -2.48642600 -
1.50396400
H 1.43257200 -3.25119800 -
3.05136000
H -0.92293000 -1.50119400
0.11484900
H -0.45949400 -3.40549200 -
1.43140700
S 3.79769900 1.03268600 -
0.53604200
O 4.56410300 2.22606300 -
0.86061000
O 4.40417600 -0.28722300 -
0.51729000
C 3.02840100 1.29505500
1.05253200
C 2.71084400 2.59525000
1.44658500
C 2.03707000 2.78940200
2.65083000
C 1.69707700 1.69461000
3.44655300
C 2.03521200 0.40101700
3.04725000
H 1.78482800 3.80176400
2.97331400
H 1.77806400 -0.45290000
3.67737700
H 3.00501200 3.44154700
0.82309700
H 1.17159500 1.85213200
4.39110600
H 2.97732700 -0.81053000
1.51358300
C 2.70463100 0.19311900
1.84354000

TS1_1a”

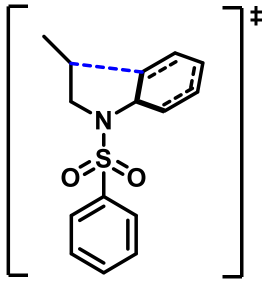

E (UωB97XD sbs) = -1183.539853
E (UωB97XD) = -1184.58708
E (UB3LYP) =-1184.90993
E (UPBE1PBE) = -1183.84445
E (UM062X) = -1184.5005
G_{corr} = 0.23576
$G_{corr(Goodvibes)}$ = 0.23845941

0 2
N 2.53035100 0.66191500 -
1.54041700
C 1.86101700 1.90553400 -
1.98737100
H 2.55332000 2.45335300 -
2.64519400
H 1.62369100 2.55815900 -
1.12838000
C 0.59337500 1.50529600 -
2.69844700
H -0.26000900 2.16930900 -
2.52606200
C 0.68825800 0.90772900 -
4.06546100
H -0.26877000 0.45825800 -
4.37064000
H 0.94549400 1.68006500 -
4.81388600
H 1.46621000 0.13023600 -
4.11890200
C 1.64645900 -0.44456000 -
1.47356500
C 2.00282700 -1.72787400 -
1.85851600
C 0.28081100 -0.07397800 -
1.28983100
C 1.00432800 -2.69121900 -
2.04942800
H 3.05408900 -1.95747900 -
2.03588300
C -0.70997200 -1.06680700 -
1.50835800
H 0.04143800 0.74679800 -
0.60692400
C -0.34700000 -2.34573000 -
1.90675800
H 1.28269900 -3.70538400 -
2.34320200
H -1.76028100 -0.82267900 -
1.33311000
H -1.11888900 -3.09965100 -
2.07910900
S 3.85750700 0.78374500 -
0.54455200
O 4.58303700 1.96332200 -
0.98547600
O 4.49194500 -0.52150900 -
0.49651600
C 3.16667500 1.12034700
1.06383300
C 2.68090000 0.05746200
1.82781100
C 2.07320200 0.33131400
3.05035500
C 1.96285400 1.64833700
3.49944900
C 2.46354700 2.69920500
2.73133600
H 1.68964900 -0.49007600
3.65893100
H 2.38714800 3.72732500
3.09119800
H 2.78697800 -0.96812300
1.47026000
H 1.48786900 1.85609300
4.46084700
H 3.48173200 3.24698100
0.89408400
C 3.07065600 2.44036200
1.50351400

INT1_1a”_b

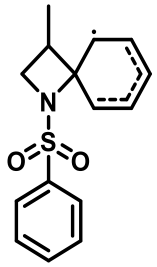

E (UωB97XD sbs) = -1183.548228
E (UωB97XD) = -1184.59508
E (UB3LYP) =-1184.90977
E (UPBE1PBE) = -1183.84714
E (UM062X) = -1184.5034
G_{corr} = 0.237273

0 2
N 2.58337700 1.01541000 -
1.69565300
C 1.93038100 2.21879500 -
2.19685700
H 2.56210600 2.84198500 -
2.84450400
H 1.48821100 2.84671400 -
1.40048400
C 0.89939200 1.29960700 -
2.88050800
H 1.21810100 1.08825900 -
3.91152300
C -0.56823100 1.65970000 -
2.84688800
H -0.76656500 2.51313800 -
3.51387900
H -1.18366000 0.81447200 -
3.19085000
H -0.90611700 1.93646900 -
1.83765300
C 1.43159400 0.10894700 -
1.96112700
C 1.79898100 -1.13990500 -
2.68961400
C 0.55217000 -0.14552100 -
0.77561100
C 1.26396900 -2.35721400 -
2.37918700
H 2.51982600 -1.02984000 -
3.50355500
C 0.04452000 -1.38199200 -
0.49464600
H 0.30384900 0.70734200 -
0.13920900
C 0.36800800 -2.50734800 -
1.29198000
H 1.54856400 -3.23509200 -
2.96431400
H -0.61178900 -1.50785600
0.37030600
H -0.04524000 -3.48843300 -
1.05122000
S 3.75510300 0.91458200 -
0.54563500
O 4.64534000 2.03684300 -
0.80276100
O 4.22861600 -0.45914200 -
0.54628200
C 2.96666200 1.21224800
1.02881200
C 2.71424000 2.52625300
1.42449000
C 2.03397300 2.75264500
2.61946200
C 1.62661400 1.67522300
3.40722700
C 1.90333500 0.36683300
3.00874100
H 1.83092800 3.77603700
2.94187500
H 1.59579900 -0.47398100
3.63401300
H 3.06369800 3.35822900
0.81036700
H 1.09747500 1.85767500
4.34526600
H 2.80018800 -0.88727900
1.48095000
C 2.57614200 0.12756500
1.81277500

INT1_1a”

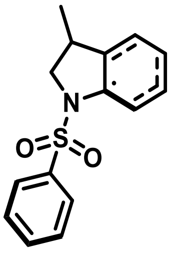

E (UωB97XD sbs) = -1183.58143
E (UωB97XD) = -1184.62614
E (UB3LYP) =-1184.94094
E (UPBE1PBE) = -1183.88064
E (UM062X) = -1184.53691
G_{corr} = 0.240067

0 2
N 2.41259000 -0.04160900 -
1.64503200
C 1.79772400 1.12777000 -
2.30515800
H 2.17617300 1.20819900 -
3.33580100
H 2.05885600 2.05530300 -
1.77791800
C 0.29448100 0.82223400 -
2.26486500
H -0.21285600 1.30506200 -
3.11304900
C -0.35104300 1.25941600 -
0.95424900
H -0.28889300 2.35140400 -
0.83037700
H -1.41305300 0.97411700 -
0.92286300
H 0.14112600 0.79145200 -
0.08611400
C 1.51445500 -1.11608900 -
1.60822000
C 0.31896400 -0.71467100 -
2.43297600
C 1.59304200 -2.31511600 -
0.94977900

C -0.89996900 -1.52188900 -
2.11693400
H 0.58278900 -0.90207400 -
3.49982300
C 0.44463100 -3.14523300 -
0.90171100
H 2.50879400 -2.60715900 -
0.43709400
C -0.79578600 -2.70046800 -
1.43393500
H -1.85955800 -1.19062800 -
2.52295600
H 0.50198200 -4.10596200 -
0.38690300
H -1.68648100 -3.31587900 -
1.28180600
S 3.67274500 0.20309700 -
0.56901100
O 4.45987400 1.28812600 -
1.12648500
O 4.26506200 -1.09050900 -
0.28583300
C 2.86255700 0.79764600
0.90014600
C 2.74300700 2.17384100
1.09617400
C 2.02186300 2.63814700
2.19369800
C 1.43306800 1.73343600
3.07722500
C 1.57471500 0.35965600
2.87797800
C 2.29696600 -0.11924700
1.78770400
H 1.92366800 3.71259200
2.36139200
H 1.12478400 -0.34602700
3.57899800
H 0.86610200 2.10197900
3.93486600
H 3.22167200 2.86618700
0.40174000
H 2.42341600 -1.19059500
1.62897600

INT2_1a”

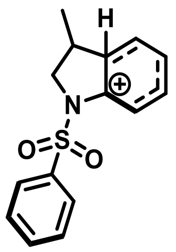

E (UωB97XD sbs) = -1183.410441
E (UωB97XD) = -1184.45474
E (UB3LYP) =-1184.77129
E (UPBE1PBE) =-1183.70878
E (UM062X) = -1184.36303
G_{corr} = 0.244516

1 1
N 2.22039600 0.01967700 -
1.47099300
C 1.70446600 1.15399200 -
2.26487100
H 2.23944300 1.18803100 -
3.22630200
H 1.87852100 2.09723600 -
1.73148800
C 0.21752800 0.80163600 -
2.42568500
H -0.16846800 1.19900900 -
3.37337000
C -0.62288400 1.30167200 -
1.25557100
H -0.62393500 2.40120600 -
1.23383200
H -1.66631900 0.96670000 -
1.34150100
H -0.22941400 0.94378800 -
0.28982700
C 1.43267800 -1.04082300 -
1.54111600
C 0.30749600 -0.74140000 -
2.47378200
C 1.50557600 -2.27398400 -
0.84760900
C -0.89075200 -1.60005100 -
2.31903300
H 0.71781900 -1.01894800 -
3.47453400
C 0.38474200 -3.06245500 -
0.87271200
H 2.39082800 -2.55261500 -
0.28029000
C -0.83163300 -2.72080500 -
1.56949900
H -1.78367000 -1.34357500 -
2.89429200
H 0.40295900 -4.00009000 -
0.31080100
H -1.68593900 -3.39431300 -
1.49449900
S 3.66434400 0.21159500 -
0.48627100
O 4.38693400 1.26653100 -
1.15376100
O 4.19774300 -1.11949800 -
0.31509800
C 2.94252600 0.80083700
1.01035300
C 2.78514300 2.17784900
1.18185800
C 2.15384800 2.63117300
2.33696400
C 1.69873500 1.71964700
3.29016500
C 1.88023000 0.34762600
3.10742900
C 2.51005100 -0.12645000
1.96086500
H 2.02491500 3.70302600
2.49703800
H 1.53691200 -0.35761000
3.86621300
H 1.20419900 2.08340800
4.19315900
H 3.16612300 2.87954900
0.43770300
H 2.67632700 -1.19506200
1.81499400

2a”

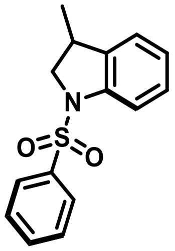

E (UωB97XD sbs) = -1183.032904
E (UωB97XD) = -1184.078
E (UB3LYP) =-1184.39343
E (UPBE1PBE) = -1183.33445
E (UM062X) = -1183.99685
G_{corr} = 0.232025

0 1
N 2.44651800 -0.11311300 -
1.65558900
C 1.77455000 1.04945300 -
2.27108400
H 2.04220100 1.08070000 -
3.33773100
H 2.12129100 1.98639800 -
1.81457500
C 0.26106900 0.81051500 -
2.07105800
H -0.27536200 1.05788300 -
2.99943600
C -0.33870900 1.61554200 -
0.91573300
H -0.26124900 2.69723600 -
1.10658600
H -1.40285400 1.37021500 -
0.77917500
H 0.18056500 1.39466300
0.02971400
C 1.49727400 -1.15639600 -
1.51550600
C 0.21397000 -0.67801700 -
1.80166500
C 1.72294200 -2.49242600 -
1.19205800
C -0.87437600 -1.53961000 -
1.75514100
C 0.61799300 -3.34562500 -
1.14071800
H 2.73073100 -2.84982900 -
0.98558100
C -0.66787100 -2.88148800 -
1.42153300
H -1.87984500 -1.17205800 -
1.97564800
H 0.77112100 -4.39765400 -
0.88932000
H -1.51457000 -3.57038800 -
1.38661000
S 3.65761200 0.15073200 -
0.53556400
O 4.48670800 1.20877600 -
1.08483000
O 4.21325100 -1.14658000 -
0.19488100
C 2.81189000 0.79381000
0.89521700
C 2.80496200 2.16946800
1.12139700
C 2.06135200 2.67393400
2.18655000
C 1.33777600 1.80813400
3.00609400
C 1.36406500 0.43159000
2.77543300
C 2.10823500 -0.08703700
1.71918200
H 2.04958100 3.74895500
2.37729800
H 0.80515600 -0.24448200
3.42546700
H 0.75295200 2.20812600
3.83725100
H 3.38226500 2.82912300
0.47155900
H 2.14348200 -1.16196600
1.53468900

1b_r

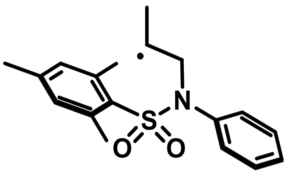

E (UωB97XD sbs) = -1301.39258
E (UωB97XD) = -1302.56201
E (UB3LYP) =-1302.92817
E (UPBE1PBE) = -1301.71009
E (UM062X) = -1302.45022
G_{corr} = 0.312175
$G_{corr(Goodvibes)}$ = 0.31670432

0 2
N -2.17420500 -1.85279500
0.59871900
C -2.32412900 -1.74079000
2.05335300
H -2.76845200 -0.74879400
2.24572800
H -3.03390200 -2.49200300
2.45341100
C -1.00635500 -1.86491800
2.73612400
H -0.16550600 -1.31382700
2.30464100
C -0.88472900 -2.51925100
4.06342000
H -1.34808800 -3.52267000
4.06377800
H 0.16568400 -2.62949500
4.36804600
H -1.39706900 -1.94489600
4.86188900
C -1.50758900 -3.00948100
0.08712800
C -1.89612700 -4.29397700
0.48086900
C -0.46551400 -2.84645300 -
0.82875500
C -1.24875400 -5.40821500 -
0.04806100
H -2.71592900 -4.42149900
1.19080800
C 0.17101600 -3.96392400 -
1.36274600
H -0.18042800 -1.83675900 -
1.12521300

C -0.21880300 -5.24602700 -
0.97440700
H -1.55765800 -6.40983300
0.25920000
H 0.97993800 -3.83186300 -
2.08461800
H 0.28352300 -6.12113000 -
1.39265700
S -3.34800500 -1.15899800 -
0.38557200
C -4.69841700 -2.35711000 -
0.42507200
C -5.70559200 -2.30545000
0.57253200
C -4.68941700 -3.38419200 -
1.39945900
C -6.66383600 -3.32139800
0.58610000
C -5.68312100 -4.36769100 -
1.31933000
C -6.66869000 -4.36644500 -
0.33771600
H -7.44499300 -3.28764600
1.35054600
H -5.67956600 -5.16575000 -
2.06624800
O -3.77989500 0.05151900
0.29433300
O -2.77291500 -1.04870800 -
1.71389100
C -5.86460200 -1.21498900
1.60625300
H -4.98842900 -1.10978600
2.25709400
H -6.01917100 -0.23753400
1.13242500
H -6.73158100 -1.43864900
2.24211300
C -7.72035500 -5.43927500 -
0.27454200
H -7.70280600 -5.95069800
0.70048600
H -8.72676700 -5.00923600 -
0.39697600
H -7.57618300 -6.19511500 -
1.05891000
C -3.71850900 -3.50470100 -
2.54939500
H -3.94409500 -4.41384900 -
3.12221000
H -3.79068600 -2.63901000 -
3.22005200
H -2.67376000 -3.56019000 -
2.22057500

TS1_1b

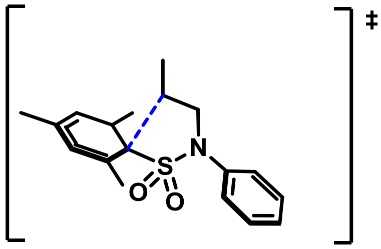

E (UωB97XD sbs) = -1301.37663
E (UωB97XD) = -1302.54276
E (UB3LYP) =-1302.90998
E (UPBE1PBE) = -1301.69528
E (UM062X) = -1302.43029
G_{corr} = 0.313732
$G_{corr(Goodvibes)}$ = 0.31730903

0 2
N -0.89039900 -1.72020900
0.24558900
C -1.27892000 -1.52857900
1.64133600
H -2.35117800 -1.25867400
1.68302100
H -1.15829900 -2.47631600
2.19305000
C -0.44574300 -0.46053800
2.28981600
H -0.71721800 0.56107200
2.00408000
C -0.00204600 -0.64337600
3.70021300
H 0.72241200 0.13303900
3.98914300
H -0.85581900 -0.56844800
4.39960000
H 0.46469600 -1.62897600
3.85584300
C -1.62883200 -2.56636000 -
0.61352100
C -2.00261800 -2.14956500 -
1.89803300
C -2.01342000 -3.83578600 -
0.16476600
C -2.72140400 -3.00692400 -
2.72604000
H -1.72971300 -1.15250300 -
2.24598200
C -2.75679000 -4.67550400 -
0.99110200
H -1.72528100 -4.17237100
0.83261500
C -3.10742800 -4.26961200 -
2.27754600
H -2.99661800 -2.67361700 -
3.72920700
H -3.05279200 -5.66195600 -
0.62673400
H -3.68216000 -4.93304100 -
2.92718600
S 0.28789800 -0.70343400 -
0.37341400
C 1.38576500 -0.59427600
1.06390900
C 2.02716200 0.66440000
1.34932700
C 2.03892100 -1.81658400
1.45905500
C 3.12135300 0.65686700
2.20670000
C 3.13089100 -1.73222500
2.31438000
C 3.68487100 -0.51510400
2.72445800
H 3.57324800 1.61769700
2.46914900
H 3.59244100 -2.66381400
2.65436100
O -0.30098300 0.59926100 -
0.66179400
O 0.95002800 -1.39854100 -
1.46833800
C 1.57399200 -3.19045300
1.04767300

H 0.63180500 -3.47071800
1.54315800
H 1.41343200 -3.26640300 -
0.03535800
H 2.32781500 -3.93581600
1.33468900
C 4.84321500 -0.46406600
3.67941300
H 4.48860900 -0.34397600
4.71729300
H 5.43967600 -1.38710500
3.64196600
H 5.50799500 0.38508900
3.46261200
C 1.55030000 1.99995600
0.84130000
H 1.61370300 2.07166300 -
0.25282900
H 0.49899700 2.19231600
1.09692200
H 2.16610100 2.79872500
1.27637100

INT1_1b

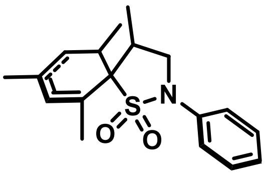

E (UωB97XD sbs) = -1301.41839
E (UωB97XD) = -1302.58053
E (UB3LYP) =-1302.94089
E (UPBE1PBE) = -1301.73078
E (UM062X) = -1302.46616
G_{corr} = 0.316579

0 2
N -1.47707000 -1.55468500 -
0.82446400
C -0.41354300 -0.99879900 -
1.64044600
H 0.55875800 -1.06436500 -
1.12725800
H -0.33018300 -1.57018700 -
2.57945100
C -0.77968400 0.45421600 -
1.95880200
H -1.53013500 0.39766800 -
2.75869500
C 0.38937900 1.27554700 -
2.48212700
H 0.82019600 0.79932100 -
3.37619500
H 0.05793900 2.28564400 -
2.76460200
H 1.18971000 1.38423500 -
1.73642100
C -1.52343700 -2.90572300 -
0.44614800
C -0.38463400 -3.71314500 -
0.58406300
C -2.70524500 -3.47671800
0.05569700
C -0.42805400 -5.05706600 -
0.21593800
H 0.54420300 -3.29732600 -
0.97539600
C -2.72708900 -4.81420900
0.43394100
H -3.61144200 -2.87697500
0.14397100
C -1.59331100 -5.61657600
0.30069200
H 0.46975200 -5.66874800 -
0.33265900
H -3.65431400 -5.23685200
0.82766400
H -1.62155100 -6.66822800
0.59252000
S -2.57076100 -0.41752900 -
0.20559800
C -1.50065000 1.10806300 -
0.75021900
C -0.66533800 1.44609300
0.45768700
C -2.45943700 2.19641300 -
1.16374200
C -0.97133900 2.55208200
1.20960500
C -2.69135700 3.26128500 -
0.33149900
C -1.99642600 3.46735000
0.88200400
H -0.38443000 2.73588800
2.11442600
H -3.42733000 4.00892000 -
0.64223600
O -2.64534800 -0.55163400
1.24362400
O -3.82674400 -0.48362400 -
0.95006800
C 0.42686600 0.52627200
0.92983100
H 1.26445900 0.45946800
0.21887900
H 0.83617700 0.88857900
1.88219600
H 0.05323300 -0.49445200
1.10395900
C -2.33586000 4.61239100
1.78637500
H -3.13922900 4.33051600
2.48970900
H -1.47040600 4.92318000
2.39014900
H -2.69471400 5.48455800
1.21934100
C -3.15803800 2.12245500 -
2.49241700
H -3.93992400 2.89154300 -
2.55267800
H -2.46028400 2.29945200 -
3.32890900
H -3.63306100 1.14380800 -
2.64952200

TS2_1b

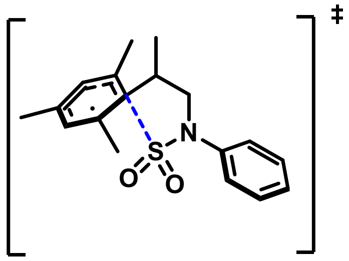

E (UωB97XD sbs) = -1301.41167
E (UωB97XD) = -1302.57313
E (UB3LYP) =-1302.93713
E (UPBE1PBE) = -1301.72355
E (UM062X) = -1302.45891
G_{corr} = 0.317174

0 2
N -0.85436700 -1.74270100
0.07038100
C 0.40683600 -1.18064700 -
0.38784000
H 1.10859600 -1.13502300
0.45959900
H 0.85662600 -1.85391800 -
1.12853000
C 0.16550900 0.21436900 -
0.99987100
H -0.38835600 0.01999200 -
1.92583400
C 1.45869000 0.91192900 -
1.40975700
H 1.93862000 0.35235600 -
2.22741900
H 1.26055600 1.93093500 -
1.77361600
H 2.18416200 0.97778800 -
0.58863600
C -1.60843500 -2.65404900 -
0.70667300
C -1.44350000 -2.77393300 -
2.09275300
C -2.54321300 -3.47277700 -
0.05828100
C -2.19325400 -3.70517600 -
2.81014600
H -0.73053900 -2.14183100 -
2.62498100
C -3.30435700 -4.38120300 -
0.78534100
H -2.66432000 -3.39063900
1.02275500
C -3.13061400 -4.50768800 -
2.16415300
H -2.04722900 -3.79197600 -
3.88933900
H -4.03197700 -5.00771700 -
0.26457500
H -3.72177800 -5.23009200 -
2.73077800
S -1.65318500 -0.51975600
0.96377400
C -0.78959100 1.06788100 -
0.11839000
C -0.21682700 1.92190600
0.94697000
C -1.95419800 1.64174300 -
0.83805100
C -0.88413400 3.06453400
1.33907700
C -2.54981800 2.79273100 -
0.38124800
C -2.05836500 3.52647300
0.72003200
H -0.46884700 3.64355600
2.16949100
H -3.43962400 3.16111900 -
0.90020300
O -1.10923800 -0.52627900
2.31935600
O -3.09568200 -0.64892900
0.77975000
C -2.53763300 0.93021400 -
2.02753200
H -3.52777300 1.33973400 -
2.26788600
H -1.90914400 1.04304000 -
2.92707400
H -2.66495300 -0.14779200 -
1.84456300
C -2.77810900 4.74687400
1.21074100
H -2.13730500 5.36593500
1.85479100
H -3.13296900 5.36876500
0.37435500
H -3.66681700 4.46446700
1.80103000
C 1.04679100 1.55641300
1.68085300
H 1.91791100 2.09049000
1.26665300
H 0.96238300 1.84397400
2.73820300
H 1.26454400 0.48352200
1.66013300

INT2_1b

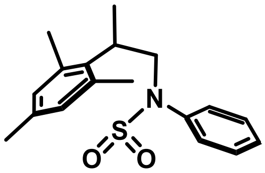

E (UωB97XD sbs) = -1301.43429
E (UωB97XD) = -1302.5955
E (UB3LYP) =-1302.96057
E (UPBE1PBE) = -1301.73971
E (UM062X) = -1302.48104
G_{corr} = 0.31587

0 2
N -0.98559400 -1.62381900
0.77112500
C 0.39274500 -1.26111800
0.45303700
H 0.86139300 -0.93244000
1.38691800
H 0.92493700 -2.18404500
0.17011600
C 0.55905700 -0.23063000 -
0.68559300
H 0.34616800 -0.78983900 -
1.60424100
C 2.02462000 0.20174900 -
0.78976700

H 2.67733400 -0.67323800 -
0.93917200
H 2.16797100 0.87591600 -
1.64696600
H 2.37406400 0.72517200
0.11109000
C -1.84811200 -2.10170500 -
0.25521600
C -3.06725000 -1.48072300 -
0.54547100
C -1.44003600 -3.20771400 -
1.00875500
C -3.87520600 -1.97677600 -
1.56400900
H -3.37897500 -0.59389700
0.00800800
C -2.24437800 -3.68314800 -
2.04167000
H -0.49501300 -3.70325300 -
0.77959100
C -3.46672100 -3.07386100 -
2.32093200
H -4.82545000 -1.48348000 -
1.77887600
H -1.91472600 -4.54631800 -
2.62398600
H -4.09826500 -3.45172400 -
3.12748700
S -1.68779800 -0.98787200
2.18339300
C -0.44255400 0.92013300 -
0.64492300
C -0.52874000 1.83051200
0.43016000
C -1.33974000 1.06832600 -
1.73241400
C -1.54645800 2.79561400
0.43550800
C -2.32716200 2.05364200 -
1.68911300
C -2.46521800 2.91940200 -
0.60203500
H -1.60842900 3.48175200
1.28555800
H -3.01624000 2.14684800 -
2.53402600
O -0.59685500 -0.81441000
3.15311700
O -2.84558200 -1.82531800
2.51714600
C 0.46743900 1.89931900
1.56536900
H 1.32525700 2.52874300
1.27632100
H 0.01045000 2.36154700
2.45149900
H 0.86374000 0.93062400
1.88270200
C -3.56715100 3.94451700 -
0.56695700
H -3.59847600 4.52969500 -
1.49896900
H -4.55144900 3.46174100 -
0.45420800
H -3.44187900 4.64545600
0.27052700
C -1.25836400 0.20299000 -
2.96854200
H -2.02346900 0.50238100 -
3.69797000
H -0.27872800 0.29428300 -
3.46421600
H -1.41798000 -0.86322900 -
2.74829700

TS3_1b

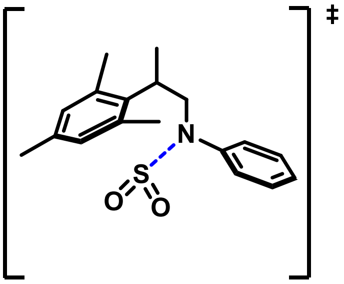

E (UωB97XD sbs) = -1301.42983
E (UωB97XD) = -1302.58729
E (UB3LYP) =-1302.95943
E (UPBE1PBE) = -1301.73513
E (UM062X) = -1302.47362
G_{corr} = 0.314025

0 2
N -1.61115000 -1.61455300
0.45954300
C -0.21400900 -1.23600300
0.58953400
H -0.08887100 -0.84870900
1.60722400
H 0.37891300 -2.16120100
0.53143500
C 0.32601000 -0.22661200 -
0.45845900
H 0.13264400 -0.68217600 -
1.43588700
C 1.85227500 -0.14347600 -
0.33959200
H 2.30866000 -1.09620200 -
0.65232700
H 2.25134700 0.64776300 -
0.99095400
H 2.19240200 0.05738500
0.68479300
C -2.02577300 -2.44096300 -
0.58321400
C -3.39241600 -2.75213800 -
0.77317700
C -1.08360700 -3.03703800 -
1.45699200
C -3.78639800 -3.59830600 -
1.80167400
H -4.13896100 -2.31573900 -
0.11434500
C -1.49485500 -3.89415900 -
2.46766900
H -0.01769100 -2.84863000 -
1.34249500
C -2.84857000 -4.17521400 -
2.65712000
H -4.84951500 -3.81289100 -
1.92959500
H -0.74396100 -4.34590400 -
3.11940800
H -3.16729800 -4.84174800 -
3.46089500

S -2.73294400 -0.33438000
1.23208700
C -0.40937300 1.11095500 -
0.46369600
C -0.27322300 2.06548600
0.56998800
C -1.27975500 1.40018200 -
1.54248600
C -1.01459700 3.25002200
0.51423900
C -1.99561700 2.60060900 -
1.55709200
C -1.88776000 3.53941000 -
0.53253100
H -0.90570700 3.97184900
1.32917000
H -2.66427100 2.80530500 -
2.39838600
O -1.97692700 0.07926000
2.42159100
O -4.02331700 -0.99501800
1.48395900
C -1.47267300 0.45750200 -
2.70839700
H -0.52475600 0.23335500 -
3.22289200
H -1.91274600 -0.50306600 -
2.40242200
H -2.14914800 0.90103200 -
3.45163400
C -2.70620600 4.80251400 -
0.53878700
H -3.65290400 4.65564000
0.00710900
H -2.17328600 5.63171800 -
0.05072900
H -2.96223500 5.11370700 -
1.56208900
C 0.65705800 1.89838600
1.74659800
H 0.63020800 0.88986400
2.17525800
H 1.69859800 2.11994500
1.46460300
H 0.38063600 2.59398800
2.55045900

2b_r

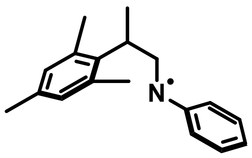

E (UωB97XD sbs) = -753.128809
E (UωB97XD) = -753.927724
E (UB3LYP) =-754.223656
E (UPBE1PBE) = -753.304003
E (UM062X) = -753.83455
G_{corr} = 0.30655

0 2
N -0.41450600 -1.44405500 -
2.65157000
C -0.36388200 -0.19497000 -
2.16122400
C -0.58377000 0.86953300 -
3.08773100
C -0.11081300 0.15667100 -
0.79859200
C -0.55229100 2.19172100 -
2.68222500
H -0.77686000 0.59461700 -
4.12669400
C -0.08261200 1.48574700 -
0.40874000
H 0.06087300 -0.62387400 -
0.05600100
C -0.30141700 2.50930700 -
1.34032300
H -0.72294900 2.98951300 -
3.40860700
H 0.11201500 1.73790400
0.63642600
H -0.27660900 3.55308800 -
1.01968100
C -0.19904000 -2.55928700 -
1.77167300
H 0.79890100 -2.47306300 -
1.30551200
H -0.92880300 -2.54670600 -
0.93741500
C -0.33974300 -3.87214900 -
2.55830000
H -1.21973100 -3.68723200 -
3.18698600
C 0.83228700 -4.08311300 -
3.52255600
H 0.97271400 -3.18425000 -
4.14264000
H 1.77601500 -4.28235800 -
2.99155300
H 0.64395700 -4.93610100 -
4.19203200
C -0.64198700 -5.11139900 -
1.72116500
C -1.71795900 -5.94305900 -
2.11769300
C 0.12494200 -5.49428700 -
0.60066800
C -2.01299600 -7.09789400 -
1.39080400
C -0.20669400 -6.66261900
0.09944300
C -1.27184400 -7.47861300 -
0.27054500
H -2.84992800 -7.72536400 -
1.71306300
H 0.40036400 -6.94239600
0.96601200
C -2.57065600 -5.62315700 -
3.32323300
H -3.12556900 -4.67980400 -
3.19721000
H -1.97045600 -5.52595300 -
4.24121600
H -3.30985100 -6.41780900 -
3.49570500
C -1.61910400 -8.72732600
0.49615600
H -2.62705600 -8.65566100
0.93536900
H -1.61448600 -9.61225200 -
0.15965300
H -0.90869200 -8.91061800
1.31495300
C 1.30114700 -4.69752400 -
0.08421500

H 1.95227900 -4.32908800 -
0.88798600
H 0.97288500 -3.82481000
0.50231900
H 1.91972100 -5.31633900
0.58106300

2b

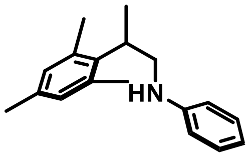

E (UωB97XD sbs) = -753.787971
E (UωB97XD) = -754.585591
E (UB3LYP) =-754.877363
E (UPBE1PBE) = -753.956308
E (UM062X) = -754.490409
G_{corr} = 0.32161

0 1
N 1.07494900 -2.06996200 -
0.82076400
C 0.02483800 -1.19856400 -
1.07293700
C -0.02239100 -0.39884100 -
2.22970300
C -1.03063300 -1.08592300 -
0.14839500
C -1.08488000 0.47690400 -
2.44259700
H 0.77794500 -0.45794300 -
2.96927100
C -2.08342000 -0.20546400 -
0.36919200
H -1.02198900 -1.71585600
0.74554000
C -2.12452900 0.58392600 -
1.52003600
H -1.09610700 1.08554000 -
3.35046700
H -2.89036900 -0.14442700
0.36543600
H -2.95559700 1.26995400 -
1.69509700
C 1.74233700 -2.77296800 -
1.89595900
H 2.20740400 -2.04870000 -
2.58373800
H 2.58082100 -3.33069600 -
1.45841400
C 0.81198900 -3.69533000 -
2.71351600
H 0.16988500 -3.00713300 -
3.27639100
C 1.58252500 -4.49587200 -
3.76743400
H 2.16543700 -3.82143900 -
4.41543700
H 2.28595600 -5.21759600 -
3.32685300
H 0.89092200 -5.06113600 -
4.40998200
C -0.12600700 -4.54485800 -
1.85701600
C -1.52214700 -4.43823500 -
2.07352400
C 0.33277300 -5.42701600 -
0.85526000
C -2.40729200 -5.19251900 -
1.30064300
C -0.59245300 -6.16645300 -
0.10536400
C -1.96609600 -6.06693900 -
0.30626200
H -3.48188600 -5.09233700 -
1.48192000
H -0.21619300 -6.84586000
0.66582100
C -2.10449200 -3.51465700 -
3.11751300
H -1.87873500 -2.45891400 -
2.89798300
H -1.72009800 -3.73247900 -
4.12624900
H -3.19795900 -3.61561000 -
3.15402400
C -2.94678500 -6.87516800
0.50140700
H -3.71988000 -6.23199900
0.95001300
H -3.46679100 -7.61316500 -
0.13053500
H -2.44853400 -7.42298400
1.31403000
C 1.79100200 -5.60224300 -
0.49534000
H 2.45452900 -5.61811900 -
1.36839600
H 2.14332000 -4.79616300
0.16984600
H 1.94013700 -6.54656600
0.04700500
H 0.89525700 -2.66599900 -
0.02021200

TS1_1b”

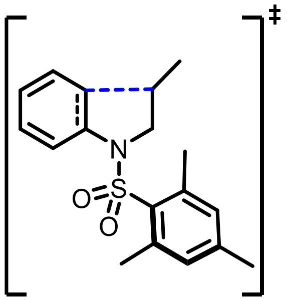

E (UωB97XD sbs) = -1301.37126
E (UωB97XD) = -1302.53935
E (UB3LYP) =-1302.90668
E (UPBE1PBE) = -1301.69209
E (UM062X) = -1302.42603
G_{corr} = 0.312163
$G_{corr(GoodVibes)}$ = 0.31646508

0 2
N 2.11037800 1.49842700 -
1.11515700
C 0.89557200 2.06315600 -
0.46554800
H 0.93317300 3.16139600 -
0.55192300

H 0.86962300 1.81703800
0.60800800
C -0.30843200 1.48241800 -
1.16115800
H -1.14795700 1.23915200 -
0.50174900
C -0.67677600 1.98845400 -
2.51904200
H -1.43134200 1.34359900 -
2.99430400
H -1.10456200 3.00621000 -
2.45437700
H 0.19685200 2.04323900 -
3.18754600
C 1.81628100 0.32399600 -
1.86095600
C 2.36005400 0.07238000 -
3.11049800
C 0.68353800 -0.40121800 -
1.38847000
C 1.81482600 -0.93942500 -
3.91339900
H 3.18216100 0.68964600 -
3.47752000
C 0.13902400 -1.40743300 -
2.22761600
H 0.52831900 -0.48815400 -
0.30946100
C 0.68856300 -1.65066900 -
3.47931600
H 2.25135400 -1.14619400 -
4.89260900
H -0.70008100 -2.00820300 -
1.86949300
H 0.25765400 -2.42411100 -
4.11971000
S 3.51561100 1.54666200 -
0.19437900
O 3.63551500 0.31776800
0.57778900
O 3.46143300 2.80411300
0.53861500
C 4.78935500 1.62496500 -
1.45165000
C 4.84012400 2.79067100 -
2.25541600
C 5.82186900 2.86020700 -
3.24272400
C 6.74273600 1.83144400 -
3.45411900
C 6.65627300 0.70031300 -
2.64724500
H 5.86670500 3.75388500 -
3.87127900
H 7.36232300 -0.11912000 -
2.80540300
C 5.69406800 0.55825800 -
1.63904700
C 3.88272800 3.94684400 -
2.12582200
H 3.95986900 4.42083700 -
1.13910000
H 4.09056100 4.69715000 -
2.89986200
H 2.84196900 3.60951300 -
2.23892100
C 7.80341600 1.95957400 -
4.51191800
H 7.38636100 2.35606800 -
5.44962100
H 8.59099300 2.65770700 -
4.18496300
H 8.28118300 0.99371100 -
4.72704100
C 5.70000000 -0.73571600 -
0.86483300
H 5.86253200 -0.57048700
0.20759300
H 4.74348300 -1.26742200 -
0.95754600
H 6.49904400 -1.38563300 -
1.24572000

TS1_1b"_b

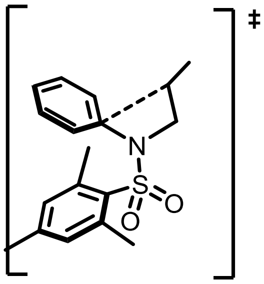

E (UωB97XD sbs) = -1301.36293
E (UωB97XD) = -1302.53232
E (UB3LYP) =-1302.89772
E (UPBE1PBE) = -1301.68236
E (UM062X) = -1302.41685
G_{corr} = 0.313222

0 2
N 2.52905900 1.05906800 -
1.70766500
C 1.84692300 2.14732400 -
2.38964600
H 2.52113200 2.73948200 -
3.02592900
H 1.31652100 2.83010500 -
1.70059600
C 0.88133200 1.24173900 -
3.12474200
H 1.29109200 0.80553700 -
4.04263400
C -0.59014000 1.48174900 -
3.12553900
H -0.85893300 2.27085800 -
3.85158800
H -1.13481800 0.57054200 -
3.41710300
H -0.95624500 1.80192900 -
2.13851800
C 1.57789000 -0.03738200 -
1.72888500
C 1.97428500 -1.27024700 -
2.33967600
C 0.56338000 -0.10647900 -
0.71410800
C 1.25061600 -2.42801100 -
2.12034900
H 2.84223100 -1.25817600 -
3.00112100
C -0.14906200 -1.27284700 -
0.51101100
H 0.33772900 0.78966500 -
0.13177600
C 0.17201100 -2.43894000 -
1.22291100

H 1.54052400 -3.34875900 -
2.63215600
H -0.94900700 -1.29228400
0.23305900
H -0.38727900 -3.36028100 -
1.04812300
S 3.79534900 1.19445200 -
0.64598400
O 4.46786900 2.42997300 -
1.01802600
O 4.53140200 -0.05678000 -
0.69616000
C 3.05692500 1.36158400
0.99157500
C 2.68320500 2.64539700
1.45405300
C 2.01540600 2.73095700
2.68031400
C 1.71111000 1.61038900
3.44968400
C 2.10161600 0.36324300
2.96492300
H 1.72521000 3.72011300
3.04456600
H 1.87420500 -0.52892600
3.55465100
C 2.77240200 0.19867100
1.74974300
C 2.97168200 3.94898800
0.74750100
H 4.05186700 4.13637100
0.69017200
H 2.60233400 3.96987100 -
0.28496600
H 2.50076500 4.77561400
1.29636400
C 1.01170900 1.73684200
4.77483000
H 0.25249600 0.95116700
4.90335100
H 1.73191100 1.63261500
5.60261800
H 0.52012100 2.71392900
4.88265800
C 3.13821400 -1.21451000
1.36498100
H 4.22748200 -1.35032600
1.34725000
H 2.70734200 -1.91391100
2.09394000
H 2.77316900 -1.49240400
0.36820100

INT1_1b”

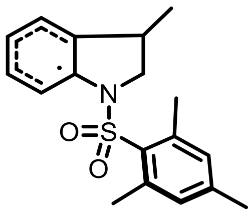

E (UωB97XD sbs) = -1301.41202
E (UωB97XD) = -1302.57828
E (UB3LYP) =-1302.93804
E (UPBE1PBE) = -1301.72864
E (UM062X) = -1302.46259
G_{corr} = 0.317375

0 2
N 1.94825700 -0.12259000 -
1.09432900
C 1.37602500 1.10468200 -
1.66713300
H 1.99804800 1.46709500 -
2.50217400
H 1.32018800 1.89442200 -
0.90248000
C -0.00666400 0.64379100 -
2.14613900
H -0.34426300 1.27444900 -
2.98156700
C -1.04515200 0.66229900 -
1.03019400
H -1.21187000 1.68778200 -
0.66675300
H -2.00915500 0.26519000 -
1.38089700
H -0.72582700 0.04667200 -
0.17419500
C 1.31792700 -1.26703400 -
1.60013700
C 0.33177400 -0.78472300 -
2.63259400
C 1.42834700 -2.58232900 -
1.23603900
C -0.76349800 -1.76785300 -
2.89615500
H 0.90624700 -0.65177700 -
3.57930000
C 0.48200100 -3.51120100 -
1.74161000
H 2.19895900 -2.90154600 -
0.53679400
C -0.62956600 -3.07199900 -
2.50913000
H -1.62472100 -1.44113300 -
3.48522200
H 0.57019900 -4.56391300 -
1.46706000
H -1.40105400 -3.79683800 -
2.78280100
S 3.41191600 -0.02033000 -
0.28766300
O 4.33520100 0.68897700 -
1.15752200
O 3.76452900 -1.35510000
0.16444300
C 2.98783900 1.01078100
1.12038500
C 3.53223600 2.30493900
1.26789500
C 3.14003200 3.04423600
2.39235100
C 2.25213800 2.55539100
3.34562100
C 1.73938900 1.26877200
3.16457000
C 2.08708700 0.47404600
2.07404100
H 3.55343000 4.04807600
2.51898900
H 1.04243600 0.86374000
3.90346200
C 4.50092700 2.96976300
0.32076200
H 4.76295600 3.96462100
0.70488100
H 5.42237900 2.38605300
0.20195500

H 4.07792200 3.08668100 -
0.68585200
C 1.85703100 3.37147300
4.54484800
H 0.76189000 3.44537100
4.62830700
H 2.22184900 2.90134900
5.47179300
H 2.26725600 4.38959300
4.49745800
C 1.48889300 -0.90624400
1.98815200
H 0.96416200 -1.07450600
1.03785000
H 2.26570400 -1.67857900
2.06396500
H 0.76751800 -1.05347500
2.80233800

INT1_1b”_b

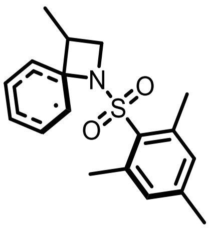

E (UωB97XD sbs) = -1301.37947
E (UωB97XD) = -1302.54774
E (UB3LYP) =-1302.90678
E (UPBE1PBE) = -1301.69494
E (UM062X) = -1302.42965
G_{corr} = 0.316608

0 2
N 2.57288800 1.07641400 -
1.73877800
C 1.98144300 2.19203800 -
2.46605900
H 2.66227100 2.68684600 -
3.17227400
H 1.50860700 2.95228400 -
1.81873400
C 0.96495500 1.19037500 -
3.04792400
H 1.32823600 0.81155600 -
4.01416900
C -0.49430300 1.57099300 -
3.14440700
H -0.64180000 2.31441700 -
3.94335300
H -1.11027500 0.69141500 -
3.38612500
H -0.87421400 2.00371700 -
2.20747900
C 1.42766800 0.15258700 -
1.92333500
C 1.78678900 -1.20120900 -
2.43805800
C 0.47913100 0.10079500 -
0.76396900
C 1.14035200 -2.33312100 -
2.02919200
H 2.57929700 -1.24048600 -
3.18904300
C -0.14121600 -1.05450300 -
0.38162900
H 0.27133000 1.03772000 -
0.24138200
C 0.14815700 -2.28650500 -
1.01809100
H 1.40989300 -3.29551900 -
2.47111200
H -0.85205100 -1.03263800
0.44820000
H -0.35463300 -3.20127300 -
0.69926500
S 3.80194600 1.06887000 -
0.64243100
O 4.58326900 2.25959900 -
0.94319600
O 4.43986800 -0.23438900 -
0.72173400
C 3.03682600 1.23464400
0.98360500
C 2.70375700 2.52713700
1.45881200
C 2.02126200 2.62260700
2.67311900
C 1.66247800 1.50258300
3.42414500
C 2.01228300 0.25017100
2.92903500
H 1.76263000 3.61715400
3.04725700
H 1.74224000 -0.64103200
3.50167000
C 2.69587400 0.07647300
1.71925400
C 3.05284700 3.82452000
0.76935500
H 2.58732500 4.66302700
1.30475100
H 4.13951100 3.98003200
0.74928700
H 2.72011000 3.85875500 -
0.27490300
C 0.94325800 1.65716400
4.73570700
H 1.62634100 2.04544700
5.50841300
H 0.10925100 2.37015200
4.65196200
H 0.54280000 0.69873900
5.09447000
C 3.00958300 -1.34429200
1.31956700
H 4.09158500 -1.53113100
1.34495500
H 2.51639100 -2.03708800
2.01479900
H 2.67941300 -1.58327200
0.30193900

INT2_1b”

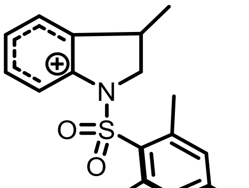

E (UωB97XD sbs) = -1301.24661
E (UωB97XD) = -1302.41308

E (UB3LYP) =-1302.77466
E (UPBE1PBE) = -1301.56302
E (UM062X) = -1302.29481
G_{corr} = 0.323432

1 1
N 1.90767500 -0.08451400 -
1.22345800
C 1.10774800 1.10448200 -
1.58311300
H 1.59299900 1.61480700 -
2.42992400
H 1.05365200 1.79281500 -
0.72880300
C -0.24873300 0.49720800 -
1.96829600
H -0.73846700 1.11633600 -
2.73138800
C -1.17145900 0.31041500 -
0.76895200
H -1.44746500 1.28630800 -
0.34515100
H -2.09838700 -0.20477200 -
1.05913500
H -0.69110900 -0.28000000
0.02808000
C 1.42269600 -1.19220200 -
1.76089700
C 0.22117000 -0.84269000 -
2.57509400
C 1.84824500 -2.53963000 -
1.63511000
C -0.71798300 -1.95791200 -
2.83932300
H 0.66411900 -0.61528800 -
3.57555900
C 0.96561300 -3.50417400 -
2.04470200
H 2.79270600 -2.79101300 -
1.15905600
C -0.33617600 -3.23070700 -
2.60532800
H -1.67890000 -1.72458200 -
3.30422000
H 1.25293600 -4.55058900 -
1.91115800
H -0.98974200 -4.06887300 -
2.84960700
S 3.44931700 0.14385600 -
0.38743600
O 4.18799200 0.99127800 -
1.29301300
O 3.92511900 -1.18659200 -
0.08128200
C 2.91260800 1.00566700
1.06329800
C 3.24553400 2.37028100
1.24377800
C 2.78947400 2.98051800
2.41677600
C 2.03958100 2.30741500
3.37906300
C 1.73040500 0.96281100
3.15430000
C 2.15292700 0.27927700
2.01784200
H 3.03941400 4.03148600
2.58070100
H 1.14310900 0.42042100
3.89979800
C 4.05397800 3.21998000
0.29613000
H 4.15418600 4.23097800
0.71005800
H 5.06053700 2.81098400
0.13900400
H 3.58620900 3.29990100 -
0.69438700
C 1.59412100 2.99181900
4.63813200
H 2.22024000 2.66931400
5.48568400
H 1.67512300 4.08399900
4.55778500
H 0.55415700 2.73392300
4.88550100
C 1.77445600 -1.17419300
1.88982000
H 1.21944500 -1.38271300
0.96387600
H 2.65999000 -1.82300400
1.89582200
H 1.12826500 -1.46320800
2.72788100

2b''

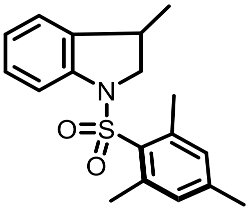

E (UωB97XD sbs) = -1300.86342
E (UωB97XD) = -1302.0294
E (UB3LYP) =-1302.38993
E (UPBE1PBE) = -1301.18193
E (UM062X) = -1301.92184
G_{corr} = 0.308888

0 1
N 1.82166800 -0.09730800 -
1.12353400
C 1.01495000 1.01158000 -
1.65275200
H 1.49918900 1.43074300 -
2.54973800
H 0.91816800 1.81057800 -
0.90209200
C -0.33723300 0.35225800 -
1.98832800
H -0.73726000 0.77531500 -
2.92169800
C -1.37169800 0.52350000 -
0.87346600
H -1.63809500 1.58368100 -
0.74191300
H -2.29216400 -0.03395900 -
1.10262500
H -0.97972400 0.14801900
0.08507800
C 1.32570800 -1.32411000 -
1.61032700
C 0.05790000 -1.09876600 -
2.16764400
C 1.88652400 -2.59914500 -
1.58544700

```
C -0.66503200 -2.15191700 -2.70727600
C 1.14434500 -3.64888700 -2.13632300
H 2.87421700 -2.76652700 -1.16153400
C -0.11497600 -3.43838700 -2.69476000
H -1.65374900 -1.97579000 -3.13911500
H 1.57285400 -4.65390300 -2.13115000
H -0.67013800 -4.27489400 -3.12415700
S 3.31249800 0.23724300 -0.46803700
O 3.98866600 1.16291900 -1.36191100
O 3.95817200 -1.02787200 -0.15290600
C 2.86834800 1.08100000 1.05600100
C 3.30662300 2.39778700 1.31332300
C 2.92069000 2.98013400 2.52855000
C 2.14299300 2.31510400 3.47139600
C 1.74203900 1.00824100 3.18478800
C 2.08798600 0.36473800 1.99796600
H 3.25236400 4.00022800 2.73914800
H 1.13917200 0.46302200 3.91640700
C 4.15889300 3.24018800 0.39595800
H 4.38930200 4.19508400 0.88686400
H 5.10227300 2.74247700 0.13857800
H 3.65151200 3.45158800 -0.55464000
C 1.75482400 2.96451500 4.77071600
H 2.23859700 2.45509400 5.61925300
H 2.04851100 4.02283500 4.79983900
H 0.66783800 2.90456000 4.93346200
C 1.59189100 -1.04390600 1.79158400
H 0.78403700 -1.07730600 1.04550800
H 2.38694800 -1.71152600 1.43803200
H 1.19357200 -1.44332100 2.73342000
```

1c_r

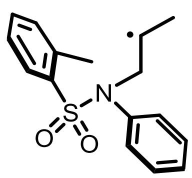

E (UωB97XD sbs) = -1222.83842

E (UωB97XD) = -1223.92596
E (UB3LYP) =-1224.2628
E (UPBE1PBE) = -1223.1441
E (UM062X) = -1223.83185
G_{corr} = 0.257606
$G_{corr(Goodvibes)}$ = 0.26246487

```
0 2
N -1.84089500 -1.62479000 -0.96210400
C -1.03410500 -0.77294800 -1.84234400
H -1.74372900 -0.12395000 -2.38490900
H -0.36704100 -0.10018700 -1.26742100
C -0.23736300 -1.59406100 -2.79556300
H -0.74176200 -2.44484800 -3.26340100
C 1.08518500 -1.13421100 -3.28931200
H 1.74041200 -0.81733300 -2.45821500
H 1.60485200 -1.92286800 -3.85207300
H 0.99851900 -0.25826900 -3.96433900
C -1.17003700 -2.63284300 -0.19980900
C -1.69120800 -3.92877300 -0.16665900
C -0.00353600 -2.33398200 0.51257700
C -1.05469900 -4.91459800 0.58316700
H -2.60149600 -4.14503900 -0.72592300
C 0.63783800 -3.32832700 1.24759600
H 0.40059500 -1.31977900 0.49888300
C 0.11206800 -4.61911600 1.28829600
H -1.46933500 -5.92476200 0.60812200
H 1.54990700 -3.08903700 1.79889900
H 0.61348300 -5.39692200 1.86852500
S -3.23526200 -0.97624400 -0.28757300
C -2.66637100 -0.07870000 1.15826900
C -2.26424500 1.27168900 1.12195300
C -2.57363600 -0.84119700 2.32682300
C -1.75536200 1.80412200 2.31466500
C -2.06024000 -0.28007600 3.49067500
H -2.91094700 -1.87821400 2.31367300
C -1.64617400 1.04872700 3.47930700
H -1.44166600 2.85086400 2.32461800
H -1.98816900 -0.88090100 4.39908700
```

H -1.24116500 1.50698100
4.38435100
O -3.78470600 -0.05515800 -
1.26910100
O -4.03482800 -2.09132700
0.18957900
C -2.38167500 2.17003200 -
0.08225400
H -1.82351200 1.78642900 -
0.94647300
H -3.42746900 2.26545900 -
0.40328800
H -1.99315200 3.16919900
0.15658500

TS1_1c

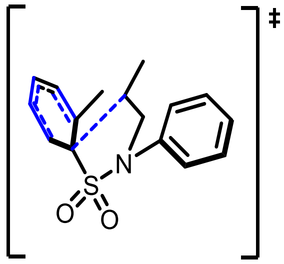

E (UωB97XD sbs) = -1222.8229
E (UωB97XD) = -1223.90728
E (UB3LYP) =-1224.24459
E (UPBE1PBE) = -1223.12907
E (UM062X) = -1223.81235
G_{corr} = 0.262556
$G_{corr(Goodvibes)}$ = 0.2651711

0 2
N -0.85300100 -1.75274800
0.25913000
C -1.32231700 -1.53235800
1.62700900
H -2.35447400 -1.13503100
1.60289800
H -1.35247900 -2.50136400
2.15244800
C -0.42013300 -0.57473200
2.35795600
H -0.62138700 0.48164100
2.15168300
C 0.00834000 -0.88827000
3.75021900
H 0.80296100 -0.20197600
4.08052500
H -0.83444700 -0.77562400
4.45751900
H 0.37970500 -1.92083800
3.84264500
C -1.61213800 -2.52920200 -
0.65367700
C -2.08689700 -1.98216800 -
1.85075300
C -1.90327100 -3.86009200 -
0.33620200
C -2.82474200 -2.77143400 -
2.72946200
H -1.87259400 -0.93917300 -
2.09038100
C -2.66650600 -4.63398700 -
1.20683500
H -1.52254700 -4.28999700
0.59249200
C -3.12375500 -4.09501200 -
2.40911100
H -3.18068100 -2.34030000 -
3.66776600
H -2.89170200 -5.67149300 -
0.94974900
H -3.71256000 -4.70668300 -
3.09602100
S 0.34585100 -0.73994200 -
0.30478800
C 1.41229200 -0.67518800
1.14622400
C 1.98650700 0.60397100
1.38958100
C 2.10950500 -1.85434900
1.57038600
C 3.09367400 0.73110100
2.20719800
H 1.50798400 1.48067600
0.95064400
C 3.21589400 -1.67653600
2.39730200
C 3.70749000 -0.41144900
2.73216200
H 3.49731700 1.72325100
2.41989600
H 3.72990600 -2.56520700
2.77308600
H 4.58655300 -0.32146600
3.37339700
O -0.17000400 0.60733400 -
0.53353800
O 1.00272300 -1.41962500 -
1.41153500
C 1.64829300 -3.24156800
1.22227800
H 0.69696200 -3.48354300
1.72327400
H 1.48669800 -3.35579800
0.14137300
H 2.39314200 -3.98228100
1.54327600

INT1_1c

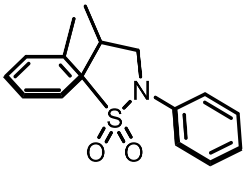

E (UωB97XD sbs) = -1222.8599
E (UωB97XD) = -1223.94063
E (UB3LYP) =-1224.27199
E (UPBE1PBE) = -1223.16121
E (UM062X) = -1223.84421
G_{corr} = 0.265479

0 2
N -1.48713500 -1.22015600 -
0.99784800
C -0.27649800 -0.69139000 -
1.60003900
H 0.60948000 -0.91604600 -
0.97849600
H -0.11615200 -1.15403000 -
2.58694500

C -0.45574700 0.82118500 -
1.74846900
H -1.14745500 0.98487500 -
2.58825400
C 0.85113700 1.54332600 -
2.03780400
H 1.30594300 1.15897300 -
2.96345200
H 0.68399900 2.62285400 -
2.16562500
H 1.57426300 1.41068300 -
1.21877200
C -1.71250700 -2.59173100 -
0.80354900
C -0.65217800 -3.50277200 -
0.91519600
C -3.00074300 -3.07687600 -
0.52328300
C -0.87790000 -4.86675900 -
0.73577100
H 0.35653700 -3.15375000 -
1.13856600
C -3.20604800 -4.43795900 -
0.32925700
H -3.84601000 -2.39043700 -
0.46404800
C -2.15047300 -5.34439200 -
0.43504200
H -0.03908400 -5.56072800 -
0.82848100
H -4.21413500 -4.79416100 -
0.10511300
H -2.32161400 -6.41282900 -
0.28978600
S -2.49942200 -0.04182100 -
0.32262200
C -1.15723500 1.33391200 -
0.46725800
C -0.34068000 1.18408700
0.76890700
C -1.84858800 2.66230300 -
0.59525700
C -0.40370100 2.07935200
1.79660800
C -1.88145400 3.51024400
0.48830400
C -1.20386600 3.23929700
1.69413600
H 0.17767000 1.89872900
2.70343300
H -2.42752300 4.45245700
0.39195700
H -1.26418900 3.94376000
2.52525800
O -2.79796500 -0.38431000
1.05965900
O -3.62488600 0.21803900 -
1.21696700
C -2.48356900 3.04951200 -
1.89734300
H -3.16736100 2.26773800 -
2.25887400
H -3.05127500 3.98325800 -
1.78626800
H -1.72401700 3.21425100 -
2.68076700
H 0.27686400 0.28875100
0.86830400

TS2_1c

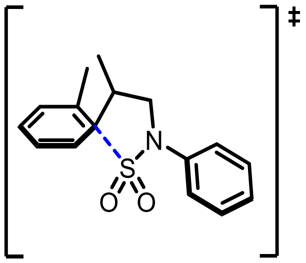

E (UωB97XD sbs) = -1222.85031
E (UωB97XD) = -1223.92995
E (UB3LYP) =-1224.26555
E (UPBE1PBE) = -1223.15087
E (UM062X) = -1223.83413
G_{corr} = 0.266415

0 2
N -0.83808700 -1.77389300
0.09043200
C 0.41268000 -1.19801000 -
0.37989200
H 1.11596200 -1.12857800
0.46508300
H 0.87054100 -1.87251500 -
1.11430800
C 0.14496800 0.18728100 -
1.00030800
H -0.43899600 -0.00685100 -
1.91340900
C 1.42256100 0.89715400 -
1.43390000
H 1.90405900 0.33301500 -
2.24732200
H 1.20574800 1.90776300 -
1.80978800
H 2.15239200 0.98379600 -
0.61792900
C -1.59740000 -2.67958900 -
0.69034200
C -1.42439500 -2.80630100 -
2.07463900
C -2.53299500 -3.49844400 -
0.04280100
C -2.16986600 -3.74132600 -
2.79159500
H -0.70838300 -2.17775500 -
2.60669900
C -3.28958100 -4.41059800 -
0.76966100
H -2.65847400 -3.41511100
1.03760000
C -3.10949000 -4.54222400 -
2.14718600
H -2.01751600 -3.83290600 -
3.86950000
H -4.01800500 -5.03655500 -
0.24938800
H -3.69683400 -5.26785400 -
2.71364200
S -1.63111000 -0.56328900
1.00172300
C -0.80117000 1.03386200 -
0.10625900
C -0.25891300 1.92231700
0.94389600
C -1.96355800 1.55600300 -
0.82981400

C -0.97290000 3.05383700
1.29759100
C -2.62390800 2.69283200 -
0.44539900
C -2.14817400 3.44967400
0.64230400
H -0.59080900 3.67121700
2.11512700
H -3.51193300 3.01537300 -
0.99291100
H -2.67338300 4.35238000
0.95974800
O -1.06178700 -0.56526800
2.34646700
O -3.07354200 -0.69411400
0.83558400
C 1.01308500 1.60803900
1.68241300
H 1.87114300 2.14008000
1.23905200
H 0.93359900 1.93429200
2.72889900
H 1.24610300 0.53745300
1.69548600
H -2.33358000 0.97141500 -
1.67558100

INT2_1c

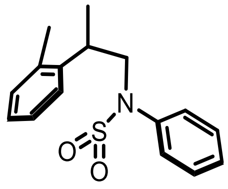

E (UωB97XD sbs) = -1222.87824
E (UωB97XD) = -1223.95709
E (UB3LYP) =-1224.29449
E (UPBE1PBE) = -1223.17205
E (UM062X) = -1223.86144
G_{corr} = 0.264555

0 2
N -1.17211300 -1.61328200
0.63254100
C 0.21167200 -1.20513000
0.41073000
H 0.60967900 -0.85899800
1.37188700
H 0.78098400 -2.11072100
0.14985800
C 0.40508800 -0.15909100 -
0.70340500
H 0.06306100 -0.64494100 -
1.63150300
C 1.89534600 0.13587300 -
0.89141500
H 2.42300300 -0.77002100 -
1.22867300
H 2.04591600 0.91406600 -
1.65377900
H 2.37977300 0.47081800
0.03607100
C -1.86988200 -2.31531800 -
0.39268300
C -3.03917400 -1.81452500 -
0.97431000
C -1.35345700 -3.54090200 -
0.82870500
C -3.68752000 -2.54025500 -
1.97052600
H -3.44458700 -0.85405800 -
0.65300900
C -1.99551000 -4.24867000 -
1.84114400
H -0.45465100 -3.94721200 -
0.36113700
C -3.16652000 -3.75413300 -
2.41411300
H -4.60431700 -2.14202200 -
2.41054800
H -1.58257800 -5.20389300 -
2.17267000
H -3.67299600 -4.31505700 -
3.20221300
S -2.07785700 -0.75592400
1.79200300
C -0.48972800 1.06370500 -
0.53250000
C -0.35018700 2.02910900
0.49121400
C -1.54147400 1.20849100 -
1.44922700
C -1.28733400 3.06980000
0.55970300
C -2.45889700 2.25237600 -
1.37110400
C -2.33384600 3.18893400 -
0.34996600
H -1.18685200 3.81024200
1.35785200
H -3.26600100 2.32882700 -
2.10322300
H -3.04494500 4.01334900 -
0.26044100
O -1.12829700 -0.34953000
2.83726800
O -3.24631800 -1.57708300
2.12973500
C 0.77230300 2.02527300
1.49881500
H 1.70949300 2.38692500
1.04691600
H 0.53459500 2.69209300
2.33881700
H 0.96199600 1.03207500
1.92290900
H -1.64383700 0.46904800 -
2.24800800

TS3_1c

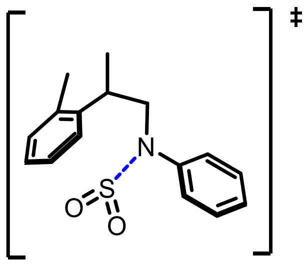

E (UωB97XD sbs) = -1222.87387
E (UωB97XD) = -1223.94854
E (UB3LYP) =-1224.29192
E (UPBE1PBE) = -1223.16655
E (UM062X) = -1223.85399

G_{corr} = 0.263773

0 2
N -1.30171100 -1.54560300
0.62358300
C 0.11040300 -1.22798000
0.49985500
H 0.43479100 -0.84590900
1.47572600
H 0.64738900 -2.17626900
0.33997300
C 0.48942300 -0.23076200 -
0.62653600
H 0.21723600 -0.71322500 -
1.57630200
C 2.00987900 -0.05792400 -
0.65855900
H 2.48674500 -0.99231400 -
0.99407300
H 2.29974100 0.73556600 -
1.36263600
H 2.43267500 0.18636700
0.32496900
C -1.95955500 -2.24403500 -
0.38790200
C -3.36781000 -2.37601700 -
0.40062000
C -1.22552800 -2.89227300 -
1.41036100
C -3.99870700 -3.10329000 -
1.40112400
H -3.96323900 -1.90247900
0.37623200
C -1.87267500 -3.63149700 -
2.39110100
H -0.13805100 -2.84831600 -
1.43026100
C -3.26361500 -3.73635400 -
2.40288200
H -5.08802900 -3.17905700 -
1.38984600
H -1.27758400 -4.13191300 -
3.15799200
H -3.76817100 -4.31021700 -
3.18259700
S -2.20598600 -0.31190400
1.69614000
C -0.34889300 1.03982000 -
0.58523600
C -0.16307100 2.10418800
0.32501000
C -1.42142700 1.10653900 -
1.48772300
C -1.05994200 3.18033900
0.28377000
C -2.30391500 2.18222400 -
1.51347700
H -1.56970000 0.27990000 -
2.18870200
C -2.12029100 3.22975800 -
0.61674500
H -0.92189800 4.00206400
0.99155300
H -3.12988000 2.19653900 -
2.22791600
H -2.80089700 4.08424200 -
0.61284400
O -1.18672700 0.07022000
2.68371300
O -3.38897200 -1.01175200
2.22163700
C 0.94999100 2.14363400
1.34057800
H 1.03533300 1.19999400
1.89485400
H 1.92116100 2.35501200
0.86681500
H 0.76454900 2.93505000
2.07956200

2c_r

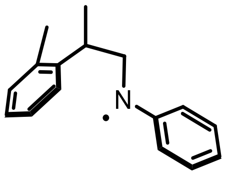

E (UωB97XD sbs) = -674.571284
E (UωB97XD) = -675.287687
E (UB3LYP) =-675.555487
E (UPBE1PBE) = -674.734077
E (UM062X) = -675.212822
G_{corr} = 0.2567

0 2
N 0.78027400 -1.34636000 -
0.85442400
C 0.12461400 -0.40184700 -
1.54775800
C -1.22327700 -0.48363100 -
2.02339200
C 0.85305000 0.79267900 -
1.84399700
C -1.78112200 0.55988900 -
2.74326400
H -1.82197000 -1.37329900 -
1.82400800
C 0.27951100 1.82709000 -
2.56023300
H 1.88088700 0.85062800 -
1.48003200
C -1.04169800 1.71853300 -
3.01627100
H -2.81014400 0.47818200 -
3.10140100
H 0.85702000 2.72967400 -
2.77265000
H -1.49507400 2.53444000 -
3.58337500
C 0.12969500 -2.58676300 -
0.54211600
H 0.58540100 -2.97749700
0.38148100
H -0.95317000 -2.48583400 -
0.34757200
C 0.32479700 -3.62501500 -
1.67969600
H -0.14390700 -3.17406900 -
2.56967600
C 1.80036600 -3.83202000 -
2.03260000
H 2.20778500 -2.91837700 -
2.48871200
H 2.42070900 -4.05259600 -
1.15356800
H 1.91757300 -4.65561100 -
2.75335800

C -0.47735400 -4.88954800 -
1.39676100
C -1.67973000 -5.04750300 -
2.10194200
C -0.11517900 -5.88064900 -
0.45624100
C -2.51872800 -6.14141600 -
1.90925300
H -1.96244500 -4.28383900 -
2.83252400
C -0.97158900 -6.97565100 -
0.27347100
C -2.16016400 -7.11655500 -
0.98412800
H -3.44568400 -6.22845500 -
2.48065000
H -0.69187600 -7.74229300
0.45455500
H -2.80099300 -7.98454700 -
0.81273800
C 1.16317300 -5.84514800
0.34639400
H 2.02679400 -6.14713600 -
0.26676700
H 1.38520300 -4.84993400
0.75351900
H 1.10491900 -6.54331200
1.19332200

2c

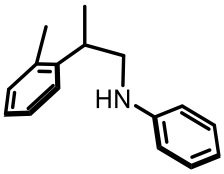

E (UωB97XD sbs) = -675.229445
E (UωB97XD) = -675.944708
E (UB3LYP) =-676.208829
E (UPBE1PBE) = -675.38619
E (UM062X) = -675.868728
G_{corr} =0.270561

0 1
C 0.20868400 -0.32722800 -
1.34265200
C -0.92761900 -0.38762200 -
2.17201300
C 1.04235000 0.80616400 -
1.44805600
C -1.21244700 0.64911200 -
3.05890000
H -1.59456100 -1.24861500 -
2.12698800
C 0.74559100 1.83156500 -
2.33670800
H 1.93191400 0.87250900 -
0.81479500
C -0.38554000 1.76637800 -
3.15378900
H -2.10235400 0.57411300 -
3.68926100
H 1.41182200 2.69632000 -
2.39275800
H -0.61516700 2.57273200 -
3.85291300
C -0.08112600 -2.62499200 -
0.40886100
H 0.22247800 -3.10986800
0.53255700
H -1.17918500 -2.53809900 -
0.36146200
C 0.31579100 -3.55007100 -
1.58585000
H -0.07118600 -3.09021100 -
2.50807900
C 1.83561300 -3.62534900 -
1.72982100
H 2.23982000 -2.64669200 -
2.02763500
H 2.33122900 -3.91166000 -
0.78780300
H 2.12728800 -4.35470300 -
2.50021100
C -0.36307700 -4.89915000 -
1.41204500
C -1.66271000 -5.13209500 -
1.91446500
C 0.27429200 -5.92282800 -
0.70005300
C -2.26197700 -6.37766700 -
1.69204400
C -0.33696100 -7.15580600 -
0.48158900
C -1.61433700 -7.38638300 -
0.98254800
H -3.26489300 -6.55873500 -
2.08850100
H 0.18844700 -7.93409700
0.07679800
H -2.10757000 -8.34839100 -
0.82516000
H 1.27934600 -5.75835900 -
0.30587700
C -2.41948500 -4.07747000 -
2.68187600
H -2.64274900 -3.20030300 -
2.05414000
H -1.85508400 -3.71740200 -
3.55616000
H -3.37927500 -4.47029000 -
3.04476300
N 0.51245000 -1.31240800 -
0.42869500
H 1.41981200 -1.23012000
0.00953900

1c'_react

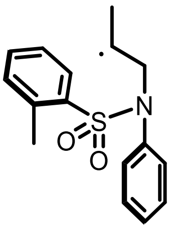

E (UωB97XD sbs) = -1222.84007
E (UωB97XD) = -1223.92506
E (UB3LYP) =-1224.26166
E (UPBE1PBE) = -1223.14246
E (UM062X) = -1223.83208
G_{corr} = 0.260833

0 2

N -1.81394100 -1.74439400 -
1.82098700
C -2.89943700 -1.56008100 -
2.81335200
H -3.02697100 -2.55834000 -
3.27082600
H -3.84493500 -1.31456600 -
2.30246800
C -2.58703200 -0.55561800 -
3.86866800
H -3.31743500 0.23270600 -
4.06857600
C -1.41197600 -0.70407200 -
4.76568500
H -1.69957800 -1.13249900 -
5.74631800
H -0.93601000 0.26813500 -
4.97559200
H -0.65077200 -1.36809000 -
4.32770600
C -1.75016300 -3.03516700 -
1.19765800
C -2.47717400 -3.32710100 -
0.03979000
C -0.98655000 -4.03223400 -
1.80774800
C -2.42077900 -4.60472900
0.51153100
H -3.07844200 -2.54730500
0.43179600
C -0.94717300 -5.31425300 -
1.26328800
H -0.42868800 -3.79455200 -
2.71622900
C -1.65956900 -5.60104800 -
0.09940100
H -2.98136700 -4.82394700
1.42291200
H -0.34969200 -6.09033500 -
1.74679800
H -1.62165700 -6.60343800
0.33273500
S -1.51172000 -0.41420400 -
0.82523300
C -0.57067600 0.68894700 -
1.86876400
C -1.17925700 1.89913000 -
2.20008300
C 0.72768400 0.36130900 -
2.30025800
C -0.50339200 2.81904200 -
2.99487500
C 1.37947600 1.30859800 -
3.09851200
C 0.77976000 2.51759700 -
3.44529600
H -0.97866200 3.76580800 -
3.25829900
H 2.38797400 1.08589200 -
3.45552600
H 1.32265300 3.22955900 -
4.07107600
O -0.66135600 -0.87660300
0.26068600
O -2.77460000 0.24650500 -
0.51063300
C 1.41409000 -0.92871700 -
1.95015100
H 1.57750600 -1.00511200 -
0.86630100
H 0.80162900 -1.79553000 -
2.23583300
H 2.38268000 -1.00195400 -
2.46222500
H -2.18612100 2.10210300 -
1.83369100

TS1_1c'_b

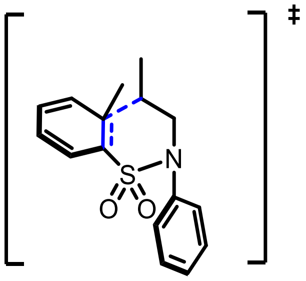

E (UPBE1PBE) = - 1223.12306
E (UM062X) = - 1223.80683
E (UB3LYP) =-1224.239
E (UωB97XD sbs) = -1222.81705
E (UωB97XD) = -1223.90126
G_{corr} = 0.263615

0 2
N -1.16125600 -1.70951600 -
1.42757300
C -0.15484000 -1.10650400 -
2.31086200
H 0.62511200 -1.84067000 -
2.57730400
H -0.68204100 -0.87048900 -
3.25173600
C 0.44440600 0.16625200 -
1.76949100
H 1.37109000 0.45387600 -
2.27964600
C -0.50124300 1.28335700 -
1.46979700
H -1.30937500 0.95695100 -
0.79717500
H 0.00998400 2.14146500 -
1.01160700
H -0.97610100 1.64242300 -
2.40225000
C -2.32417700 -2.29024100 -
2.01943100
C -2.44677400 -3.67176000 -
2.19615100
C -3.34279100 -1.43956700 -
2.45735500
C -3.59130200 -4.19440400 -
2.79428200
H -1.64575300 -4.33241000 -
1.85935100
C -4.47269200 -1.96633500 -
3.07811600
H -3.23954200 -0.36352100 -
2.30126600
C -4.60241000 -3.34551500 -
3.24262100
H -3.68661700 -5.27521000 -
2.91995900
H -5.26381300 -1.29583000 -
3.42130900

H -5.49399600 -3.75913600 -
3.71895000
S -0.58074300 -2.41235100 -
0.00808200
C 0.18760500 -0.98703400
0.71061700
C 1.37705100 -0.43163600
0.12641900
C -0.55836500 -0.31645900
1.67204000
C 1.79878700 0.80766000
0.70842100
C -0.12021100 0.90686100
2.17802300
C 1.06696400 1.46281000
1.67908300
H 2.74012400 1.23924500
0.35821600
H -0.69437400 1.41745100
2.95277200
H 1.43094100 2.41339300
2.07641500
O -1.74249400 -2.79726700
0.77647800
O 0.42492700 -3.42504100 -
0.31709800
C 2.48359300 -1.27914500 -
0.47490500
H 3.20993900 -0.63491400 -
0.99027000
H 3.01211900 -1.79914800
0.33920900
H 2.13423400 -2.05168500 -
1.16543800
H -1.48851600 -0.76710000
2.02235900

TS1_1c’

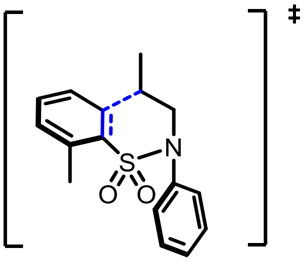

E (UωB97XD sbs) = -1222.82115
E (UωB97XD) = -1223.90557
E (UB3LYP) =-1224.24362
E (UPBE1PBE) = -1223.12748
E (UM062X) = -1223.81098
G_{corr} = 0.263545
$G_{corr(Goodvibes)}$ = 0.26618052

0 2
N -1.66581800 -2.62240700 -
2.22665800
C -0.67516700 -3.41548500 -
2.97274400
H -0.35918400 -4.29318700 -
2.38219700
H -1.20401200 -3.80398900 -
3.86081800
C 0.51587500 -2.61348200 -
3.42803300
H 1.37272400 -3.23741700 -
3.70820200
C 0.25834800 -1.43402200 -
4.30744100
H -0.51021700 -0.77302900 -
3.87825900
H 1.17125000 -0.84481400 -
4.47690200
H -0.10897200 -1.76522900 -
5.29713000
C -3.04838600 -2.93635300 -
2.41074700
C -3.72318000 -3.81191400 -
1.55488700
C -3.71720400 -2.37310400 -
3.50015600
C -5.06502400 -4.10724400 -
1.78427600
H -3.19438200 -4.25317500 -
0.70776300
C -5.05267800 -2.68996300 -
3.73808800
H -3.17807500 -1.68419500 -
4.15412500
C -5.73067900 -3.55309000 -
2.87725600
H -5.59128100 -4.78300200 -
1.10648700
H -5.57021800 -2.24893500 -
4.59296900
H -6.78099200 -3.79259200 -
3.05743300
S -1.17392700 -2.16627400 -
0.67572700
C 0.18178900 -1.11131600 -
1.11078800
C 1.32542500 -1.83205200 -
1.56256600
C 0.07939800 0.28064900 -
1.22739200
C 2.47880400 -1.08996900 -
1.92626500
H 1.44695900 -2.86223700 -
1.22234900
C 1.22244800 0.96252400 -
1.67692100
C 2.40692500 0.28736900 -
2.00389000
H 3.40366100 -1.61855600 -
2.16547400
H 1.18527400 2.05142200 -
1.75507200
H 3.28394600 0.86325400 -
2.30907100
O -2.27496100 -1.43990500 -
0.06765700
O -0.62458800 -3.32866200
0.01769000
C -1.16119300 1.06736600 -
0.89624500
H -2.02775900 0.71198200 -
1.47156700
H -1.43018900 0.96728300
0.16388300
H -1.00325300 2.13107600 -
1.11955500

INT1_1c’

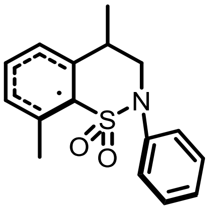

E (UωB97XD sbs) = -1222.85554
E (UωB97XD) = -1223.93829
E (UB3LYP) =-1224.26876
E (UPBE1PBE) = -1223.15839
E (UM062X) = -1223.84131
G_{corr} = 0.26644

0 2
N -1.28870400 -2.99506600 -
2.08488500
C -1.26073100 -2.98445900 -
0.61743700
H -1.31814500 -1.94829100 -
0.23678900
H -2.16358100 -3.51089400 -
0.27843500
C -0.01828000 -3.69298700 -
0.07537600
H -0.03658100 -3.56650000
1.01993800
C -0.02871600 -5.18201300 -
0.40629400
H -0.02727100 -5.34682900 -
1.49454200
H 0.85035000 -5.69208900
0.01428800
H -0.92777000 -5.66412400
0.00780300
C -2.56062600 -2.98321600 -
2.73609500
C -3.13550700 -1.79975800 -
3.20934000
C -3.24896300 -4.19227000 -
2.86484300
C -4.38614500 -1.83531900 -
3.82244900
H -2.59716700 -0.85640600 -
3.10040600
C -4.50971300 -4.21735500 -
3.45592800
H -2.78101600 -5.10962200 -
2.50094800
C -5.07857900 -3.03957700 -
3.94132300
H -4.82598700 -0.91005700 -
4.20119800
H -5.04421000 -5.16516200 -
3.55158600
H -6.06230400 -3.06117800 -
4.41541500
S -0.03897400 -2.12882100 -
2.81049900
C 1.34023700 -2.97442800 -
2.09599500
C 1.27542100 -2.97964800 -
0.58574100
C 2.36884400 -3.51907400 -
2.83558000
C 2.50485600 -3.55550400
0.03642900
C 3.45727800 -4.09208800 -
2.12186100
C 3.51571300 -4.08348600 -
0.70562900
H 2.55585700 -3.55207500
1.12872600
H 4.27972900 -4.53079100 -
2.69032000
H 4.39315200 -4.50592200 -
0.21016900
O -0.15919500 -2.32587400 -
4.24498700
O -0.01570900 -0.76339300 -
2.28640600
C 2.42691400 -3.53265600 -
4.34283200
H 1.56376000 -4.05822600 -
4.77215000
H 2.41188800 -2.51541700 -
4.75738500
H 3.34660200 -4.02936100 -
4.67949500
H 1.18397900 -1.92595600 -
0.25278600

INT2_1c’

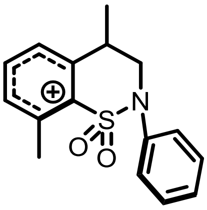

E (UωB97XD sbs) = -1222.64661
E (UωB97XD) = -1223.73336
E (UB3LYP) =-1224.0667
E (UPBE1PBE) = -1222.95397
E (UM062X) = -1223.63253
G_{corr} = 0.270697

1 1
N -1.03397400 -1.88250100 -
1.43671100
C 0.04000600 -1.25551600 -
2.20613500
H 0.89040900 -1.94845400 -
2.31273300
H -0.35473300 -1.05597000 -
3.21046100
C 0.48855200 0.06594800 -
1.57553300
H 1.37736500 0.40620600 -
2.12774600
C -0.59228100 1.14011600 -
1.62807600
H -1.47158400 0.86288600 -
1.02674600
H -0.22221500 2.11076600 -
1.26828500
H -0.92495700 1.28095800 -
2.66666000
C -2.23707600 -2.35718700 -
2.05658900
C -2.41395600 -3.71994200 -
2.30337500
C -3.21125400 -1.43485200 -
2.44079400
C -3.58039400 -4.15896100 -
2.92474600

H -1.64045300 -4.43261300 -2.00991700
C -4.36564600 -1.87942900 -3.08083000
H -3.05992600 -0.37323000 -2.23834100
C -4.55356500 -3.24097600 -3.31776600
H -3.72406900 -5.22511400 -3.11085600
H -5.12661200 -1.15895000 -3.38706300
H -5.46277400 -3.58834400 -3.81265000
S -0.58527700 -2.44094300 0.05285900
C -0.04700600 -0.86548400 0.74523700
C 0.97733000 -0.19076400 -0.08442400
C -0.49879400 -0.36479600 1.94663100
C 1.55367300 1.02496500 0.48544200
H 1.81669100 -0.91545400 -0.19360200
C 0.11213600 0.84533500 2.37686400
C 1.12161900 1.52829200 1.68219900
H 2.33312300 1.53249900 -0.08947600
H -0.22159500 1.25709100 3.33410700
H 1.54827700 2.43937000 2.10244800
O -1.75123900 -2.96048200 0.72768900
O 0.64505500 -3.21093400 -0.02326800
C -1.53745400 -1.00720400 2.82389100
H -2.50819200 -1.05596000 2.31211500
H -1.26123200 -2.03737600 3.08203100
H -1.65594100 -0.43383100 3.75164100

2c’

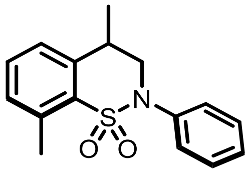

E (UωB97XD sbs) = -1222.30435
E (UωB97XD) = -1223.38734
E (UB3LYP) =-1223.71802
E (UPBE1PBE) = -1222.60962
E (UM062X) = -1223.29955
G_{corr} = 0.258862

0 1
N -1.30095600 -1.62983700 -1.36980500
C -0.10654100 -1.13613900 -2.05292900
H 0.73474800 -1.83907900 -1.91673100
H -0.32907300 -1.10026300 -3.12738000
C 0.25077600 0.25289300 -1.53643900
H 1.21027500 0.53525900 -1.99863300
C -0.80322000 1.29364000 -1.92890400
H -1.77694000 1.04614400 -1.47990200
H -0.51783300 2.29669100 -1.58020000
H -0.92080600 1.33644200 -3.02300100
C -2.34943600 -2.26713100 -2.09756500
C -2.61710600 -3.63291000 -1.95845100
C -3.10589000 -1.49944900 -2.98855100
C -3.64995900 -4.21408200 -2.69098800
H -2.01741200 -4.23724200 -1.27627800
C -4.11625000 -2.09451000 -3.73935300
H -2.90011900 -0.43173900 -3.08390200
C -4.39723000 -3.45223500 -3.58748100
H -3.86034900 -5.27872900 -2.56738800
H -4.69952700 -1.48722000 -4.43517100
H -5.19874600 -3.91545000 -4.16686400
S -1.05456200 -2.08191400 0.21747600
C -0.10712100 -0.69040000 0.83617800
C 0.46040300 0.25580000 -0.03208400
C 0.09537500 -0.64876300 2.23302700
C 1.22848500 1.28180900 0.52973400
C 0.86671000 0.39856300 2.73995100
C 1.42903900 1.35652000 1.90056100
H 1.67943100 2.02595900 -0.13165500
H 1.03396300 0.45481400 3.81810200
H 2.03540200 2.16135000 2.32161800
O -2.35966600 -2.14332700 0.85518400
O -0.18922600 -3.25801400 0.29413300
C -0.48425100 -1.66944100 3.17707700
H -1.57881800 -1.58218100 3.22914500
H -0.25786300 -2.69369300 2.84848300
H -0.07342600 -1.53208500 4.18590200

1c”_react

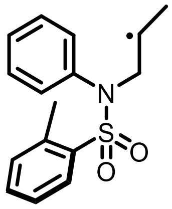

E (UωB97XD sbs) = -1222.84021
E (UωB97XD) = -1223.92764
E (UB3LYP) =-1224.26425
E (UPBE1PBE) = -1223.14563
E (UM062X) = -1223.83385
G_{corr} = 0.259572

0 2
N -2.17153400 -1.86071600
0.57650600
C -2.42280800 -1.82962000
2.02308700
H -2.88714100 -0.85166100
2.23802100
H -3.16003100 -2.59922400
2.32938200
C -1.15621800 -1.99076300
2.78864400
H -0.28048100 -1.44454100
2.42537400
C -1.14391900 -2.62601400
4.13059500
H -1.70122500 -3.57956400
4.13407600
H -0.11910100 -2.82734300
4.47452200
H -1.62282200 -1.98645100
4.90015800
C -1.45659600 -2.97821700
0.04343000
C -1.76023400 -4.28405800
0.44311300
C -0.45159800 -2.75536900 -
0.90140600
C -1.06698600 -5.35869400 -
0.10922500
H -2.54808800 -4.46258700
1.17734500
C 0.22814800 -3.83453500 -
1.45998300
H -0.22746600 -1.73120700 -
1.19990600
C -0.07693800 -5.13770900 -
1.06619500
H -1.30915200 -6.37639200
0.20484600
H 1.00704000 -3.65489200 -
2.20423100
H 0.46106300 -5.98214400 -
1.50257700
S -3.35221100 -1.18485500 -
0.41253100
C -4.66018400 -2.41321700 -
0.45331400
C -5.60977200 -2.29645600
0.56686100
C -4.70584000 -3.46518600 -
1.39132600
C -6.62259000 -3.24051800
0.69464500
H -5.55343400 -1.44850400
1.25080600
C -5.74101900 -4.39725600 -
1.23019300
C -6.68079200 -4.29893000 -
0.20785900
H -7.36271800 -3.14313200
1.49116400
H -5.80958100 -5.22474200 -
1.94060000
H -7.46874300 -5.05076900 -
0.12384400
O -3.85782000 -0.02266500
0.30137700
O -2.78291500 -1.03139900 -
1.73868700
C -3.75586900 -3.62898000 -
2.54848700
H -4.01186300 -4.53315800 -
3.11672800
H -3.80402100 -2.76307600 -
3.22216500

TS1_1c”

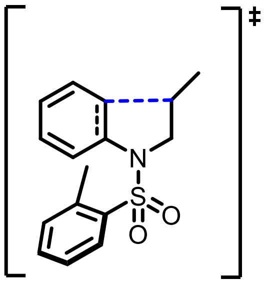

E (UωB97XD sbs) = -1222.81791
E (UωB97XD) = -1223.905
E (UB3LYP) =-1224.24293
E (UPBE1PBE) = -1223.12763
E (UM062X) = -1223.80928
G_{corr} = 0.262477
$G_{corr(Goodvibes)}$ = 0.26508955

0 2
N 2.33294800 0.73652600 -
1.51641100
C 1.40754500 1.85361700 -
1.81625900
H 1.94911100 2.59673300 -
2.42222200
H 1.07756700 2.34981000 -
0.88627000
C 0.22306800 1.27786000 -
2.55044300
H -0.74419200 1.71248300 -
2.27794100
C 0.38283000 0.89123400 -
3.98568400
H -0.46731900 0.28395900 -
4.33150000
H 0.43371600 1.78822900 -
4.63036800
H 1.30702500 0.31640500 -
4.15352300
C 1.71939800 -0.53457700 -
1.60680400

C 2.32835900 -1.64331000 -
2.17531900
C 0.31382900 -0.50039000 -
1.36401200
C 1.55476300 -2.76764700 -
2.48792600
H 3.39680000 -1.61401500 -
2.39031000
C -0.44691700 -1.64858300 -
1.70777100
H -0.06906700 0.14993400 -
0.57152400
C 0.16766300 -2.75176600 -
2.28307000
H 2.03407100 -3.64677600 -
2.92350900
H -1.51733500 -1.66623000 -
1.49013800
H -0.42851800 -3.62775700 -
2.54968200
S 3.70807300 1.05576500 -
0.64059200
O 4.22745300 2.30800700 -
1.16650300
O 4.52846600 -0.14272700 -
0.64758900
C 3.13539500 1.38401400
1.02945500
C 3.04440100 2.73248600
1.38238400
C 2.56829900 3.09663200
2.63830900
C 2.19002900 2.10289400
3.53584300
C 2.29569000 0.76043100
3.17805400
H 2.49944300 4.15149100
2.91075500
H 2.00708800 -0.01008400
3.89723700
H 3.36092000 3.48897800
0.66332400
H 1.81501900 2.37000100
4.52637100
C 2.76869900 0.35682000
1.92303700
C 2.87944400 -1.11302900
1.61695900
H 2.28528600 -1.39732900
0.73711100
H 3.91910700 -1.39245800
1.39974200
H 2.52346200 -1.70432000
2.47104800

INT1_1c”

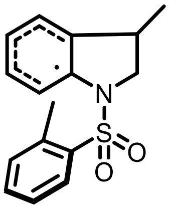

E (UωB97XD sbs) = -1222.85887
E (UωB97XD) = -1223.9433
E (UB3LYP) =-1224.27313
E (UPBE1PBE) = -1223.16307
E (UM062X) = -1223.84518
G_{corr} = 0.266061

0 2
N 2.04076600 -0.05435900 -
1.24751800
C 1.32904000 1.13891700 -
1.73209400
H 1.83689200 1.55435400 -
2.61719900
H 1.30137500 1.91409200 -
0.95122700
C -0.06250200 0.59065300 -
2.07300300
H -0.52419800 1.20166400 -
2.86233000
C -0.98023600 0.53727500 -
0.85676900
H -1.17985900 1.54893900 -
0.47197800
H -1.94497900 0.07301500 -
1.10913400
H -0.53304300 -0.05319300 -
0.04122700
C 1.43231000 -1.23548500 -
1.69177500
C 0.31389100 -0.81031200 -
2.60854700
C 1.66892800 -2.54446900 -
1.36782800
C -0.73850100 -1.86062700 -
2.76750300
H 0.77527700 -0.63633300 -
3.60892300
C 0.73499800 -3.52873700 -
1.78392700
H 2.53059400 -2.81993300 -
0.76264100
C -0.47991200 -3.15671900 -
2.41879200
H -1.67746300 -1.58530500 -
3.25528400
H 0.92173500 -4.57611400 -
1.54007900
H -1.22817600 -3.92824300 -
2.61935400
S 3.49788500 0.14453000 -
0.45966800
O 4.28978700 1.07974000 -
1.24226300
O 4.02452000 -1.17387900 -
0.15451700
C 3.02746100 0.98430800
1.04909400
C 3.47962600 2.29596800
1.19537500
C 3.15662700 3.01626500
2.34168600
C 2.38292600 2.41372800
3.32944100
C 1.94117300 1.10104100
3.17270700
C 2.25019600 0.34673600
2.03538000
H 3.50941600 4.04270800
2.45783700
H 1.33783000 0.63837000
3.95757700
H 2.11937200 2.96720300
4.23348500

H 4.08257100 2.74094300
0.40298300
C 1.76636700 -1.07191300
1.92014900
H 2.61259900 -1.77320300
1.90286000
H 1.11872700 -1.32639300
2.76937700
H 1.19633500 -1.23879000
0.99501300

INT2_1c”

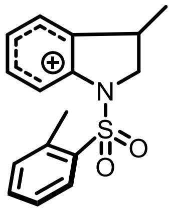

E (UωB97XD sbs) = -1222.69095
E (UωB97XD) = -1223.77534
E (UB3LYP) =-1224.10682
E (UPBE1PBE) = -1222.99463
E (UM062X) = -1223.67487
G_{corr} = 0.271864

1 1
N 2.03308200 -0.05821300 -
1.26124600
C 1.32495400 1.16086200 -
1.70933700
H 1.82806100 1.55228900 -
2.60704300
H 1.35088800 1.92580700 -
0.92094100
C -0.08478400 0.63472900 -
2.01186700
H -0.55155400 1.23287500 -
2.80534100
C -0.97646800 0.60084500 -
0.77642500
H -1.14817100 1.61956200 -
0.40059400
H -1.95594700 0.15762000 -
1.00535600
H -0.52014200 0.01311200
0.03689300
C 1.44273600 -1.16419900 -
1.69255000
C 0.26462300 -0.77912500 -
2.52358800
C 1.74450200 -2.52608900 -
1.44421700
C -0.76482900 -1.82900100 -
2.70313500
H 0.70988600 -0.65889700 -
3.54169100
C 0.77837700 -3.44199400 -
1.77208800
H 2.66535100 -2.81875500 -
0.94674700
C -0.49385800 -3.10668600 -
2.36290300
H -1.70229100 -1.55383500 -
3.19220100
H 0.97363000 -4.49372000 -
1.54570100
H -1.21660100 -3.90345000 -
2.54177200
S 3.57087100 0.10744500 -
0.42814300
O 4.32338100 1.00747500 -
1.27055800
O 4.02486400 -1.23989200 -
0.16838100
C 3.07312900 0.94004900
1.05007400
C 3.52596500 2.25577800
1.18493600
C 3.20213300 2.97234400
2.33092800
C 2.42954900 2.36699800
3.31889700
C 1.98517100 1.05480500
3.16617200
C 2.29493700 0.29563000
2.03344100
H 3.55654800 3.99751500
2.44858300
H 1.38299300 0.59395900
3.95244800
H 2.16871300 2.92042500
4.22331100
C 1.80293700 -1.12102900
1.92312200
H 1.19255900 -1.27583300
1.02065500
H 2.64045200 -1.83113100
1.88184400
H 1.17951200 -1.37334200
2.79008100
H 4.13033100 2.70712000
0.39710100

2c”

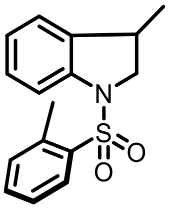

E (UωB97XD sbs) = -1222.31036
E (UωB97XD) = -1223.39485
E (UB3LYP) =-1223.72528
E (UPBE1PBE) = -1222.61664
E (UM062X) = -1223.30477
G_{corr} = 0.256612

0 1
N 2.09151200 -0.07442000 -
1.27991000
C 1.34929800 1.08600000 -
1.79367900
H 1.77563900 1.39965800 -
2.75958300
H 1.41932000 1.93250500 -
1.09431500
C -0.09523600 0.56521400 -
1.94052400
H -0.54175000 0.95923700 -
2.86539800
C -0.98142700 0.94196100 -
0.75103500

H	-1.12021300	2.03252800	-0.69170100
H	-1.97419400	0.47535000	-0.83633800
H	-0.52965900	0.60392200	0.19518900
C	1.40273900	-1.26553700	-1.58472700
C	0.11477200	-0.93269300	-2.02862200
C	1.82497800	-2.59083800	-1.51443500
C	-0.77326900	-1.93104100	-2.40083400
C	0.91583800	-3.58436500	-1.89095000
H	2.83078700	-2.83848800	-1.18152000
C	-0.36812200	-3.26853100	-2.33225200
H	-1.77858300	-1.67300100	-2.74402300
H	1.23039800	-4.62961200	-1.84499800
H	-1.05538100	-4.06362100	-2.62860100
S	3.52537500	0.14517100	-0.48412700
O	4.29911400	1.11179500	-1.24563200
O	4.08086400	-1.16730300	-0.19622100
C	3.04621800	0.94788100	1.04521500
C	3.53693500	2.23812500	1.24740200
C	3.21911500	2.92658700	2.41463000
C	2.41398700	2.31320300	3.36995300
C	1.93291400	1.02233700	3.15782900
C	2.23211200	0.30186100	1.99603800
H	3.60303300	3.93604700	2.57337200
H	1.30540800	0.55001200	3.91773400
H	2.15692900	2.84047300	4.29136500
C	1.66974000	-1.08007300	1.80642800
H	0.88066100	-1.08573400	1.03860300
H	2.44216200	-1.78890600	1.48083300
H	1.22644000	-1.44603700	2.74185600
H	4.16715000	2.69128300	0.48133100

8. References

[1] Yuan, K.; Soulé, J. F.; Dorce, V.; Doucet, H. Palladium-Catalyzed Cascade sp^2 C–H Bond Functionalizations Allowing One-Pot Access to 4-Aryl-1,2,3,4-tetrahydroquinolines from *N*-Allyl-*N*-arylsulfonamides. *ACS Catal*. **2016**, *6* (12), 8121-8126. DOI: 10.1021/acscatal.6b02586.
[2] Liu, J.; Zhang, B.; Hu, J.; Qiu, Z.; Chen, X.; Tian, X.; Wang, Q.; Zheng, G.; Yuan, M. Radical Arylaminoformylation of Activated Alkenes to Amides Containing All-Carbon Quaternary Stereocenters. *Eur. J. Org. Chem*. **2023**, *26* (6), e202201378. DOI: 10.1002/ejoc.202201378.
[3] Wang, L.-C.; Chen, B.; Wu, X. -F. Cobalt-Catalyzed Direct Aminocarbonylation of Ethers: Efficient Access to α-Amide Substituted Ether Derivatives. *Angew. Chem. Int. Ed.* **2022**, *61* (23), e202203797. DOI: 10.1002/anie.202203797.
[4] Zhao, H.; Leonori, D. Minimization of Back-Electron Transfer Enables the Elusive sp^3 CH Functionalization of Secondary Anilines. *Angew. Chem. Int. Ed*. **2021**, *60* (14), 7669-7674.
DOI: 10.1002/anie.202100051.
[5] Lunic, D.; Vystavkin, N.; Qin, J.; Teskey, C. J.; Dual-Catalytic Structural Isomerisation as a Route to *α*-Arylated Ketones. *Angew. Chem. Int. Ed*. **2024**, e202409388.
DOI: 10.1002/anie.202409388.
[6] Nakagawa, M.; Matsuki, Y.; Nagao, K.; Ohmiya, H. A Triple Photoredox/Cobalt/Brønsted Acid Catalysis Enabling Markovnikov Hydroalkoxylation of Unactivated Alkenes. *J. Am. Chem. Soc.* **2022**, *144* (18), 7953-7959. DOI: 10.1021/jacs.2c00527.
[7] Qin, Y.; Sun, R.; Gianoulis, N. P.; Nocera, D. G. Photoredox Nickel-Catalyzed C-S Cross-Coupling: Mechanism, Kinetics, and Generalization. *J. Am. Chem. Soc.* **2021**, *143* (4), 2005-2015. DOI: 10.1021/jacs.0c11937.
[8] Bergamaschi, E.; Mayerhofer, V. J.; Teskey, C. J. Light-Driven Cobalt Hydride Catalyzed Hydroarylation of Styrenes. *ACS Catal*. **2022**, *12* (24), 14806-14611.
DOI: 10.1021/acscatak.2c05109.
[9] Gaussian 16, Revision C.01, Frisch, M. J., Trucks, G. W., Schlegel, H. B., Scuseria, G. E., Robb, M. A., Cheeseman, J. R., Scalmani, G., Barone, V., Petersson, G. A., Nakatsuji, H., Li, X., Caricato, M., Marenich, A. V., Bloino, J., Janesko, B. G., Gomperts, R., Mennucci, B., Hratchian, H. P., Ortiz, J. V., Izmaylov, A. F., Sonnenberg, J. L., Williams-Young, D., Ding, F., Lipparini, F., Egidi, F., Goings, J., Peng, B., Petrone, A., Henderson, T., Ranasinghe, D., Zakrzewski, V. G., Gao, J., Rega, N., Zheng, G., Liang, W., Hada, M., Ehara, M., Toyota, K., Fukuda, R., Hasegawa, J., Ishida, M., Nakajima, T., Honda, Y., Kitao, O., Nakai, H., Vreven, T., Throssell, K., Montgomery, J. A., Jr., Peralta, J. E., Ogliaro, F., Bearpark, M. J., Heyd, J. J., Brothers, E. N., Kudin, K. N., Staroverov, V. N., Keith, T. A., Kobayashi, R., Normand, J., Raghavachari, K., Rendell, A. P., Burant, J. C., Iyengar, S. S., Tomasi, J., Cossi, M., Millam, J. M., Klene, M., Adamo, C., Cammi, R., Ochterski, J. W., Martin, R. L., Morokuma, K., Farkas, O., Foresman, J. B., Fox, D. J. Gaussian, Inc., Wallingford CT, **2016**.
[10] Chai, J.-D.; Head-Gordon, M. Long-range corrected hybrid density functionals with damped atom-atom dispersion corrections. *Phys. Chem.* **2008**, *10* (44), 6615-6620. DOI: 10.1039/B810189B.
[11] Weigend, F.; Ahlrichs, R. Balanced basis sets of split valence, triple zeta valence and quadruple zeta valence quality for H to Rn: Design and assessment of accuracy. *Phys. Chem.* **2005**, *7* (18), 3297-3305. DOI: 10.1039/B508541A.

[12] Marenich, A. V.; Cramer, C. J.; Truhlar, D. G. Universal solvation model based on solute electron density and a continuum model of the solvent defined by the bulk dielectric constant and atomic surface tensions. *J. Phys. Chem. B*. **2009**, *113* (18), 6378-6396. DOI: 10.1021/jp810292n.
[13] Becke, A.D. Density-functional thermochemistry. III. The role of exact exchange. *J. Chem. Phys*. **1993**, *98*, 5648-5652. DOI: 10.1063/1.464913.
[14] Lee, C.; Yang, W.; Parr, R.G. Development of the Colle-Salvetti correlation-energy formula into a functional of the electron density. *Phys. Rev. B*. **1988**, *37* (2), 785-789S. DOI: 10.1103/PhysRevB.37.785.
[15] Grimme, S.; Antony, J.; Ehrlich, S.; Krieg, H. A consistent and accurate ab initio parameterization of density functional dispersion correction (DFT-D) for the 94 elements H-Pu. *J. Chem. Phys.* **2010**, *132* (15), 154104. DOI: 10.1063/1.3382344.
[16] Adamo, C.; and Barone, V. Toward reliable density functional methods without adjustable parameters: The PBE0 model. *J. Chem. Phys.* **1999**, *110* (13), 6158-6170. DOI: 10.1063/1.478522.
[17] Zhao, Y.; Truhlar, D. G. The M06 suite of density functionals for main group thermochemistry, thermochemical kinetics, noncovalent interactions, excited states, and transition elements: two new functionals and systematic testing of four M06-class functionals and 12 other functionals. *Theor. Chem. Acc*. **2008**, *120*, 215-241. DOI: 10.1007/s00214-007-0310-x.
[18] Grimme, S. Supramolecular Binding Thermodynamics by Dispersion-Corrected Density Functional Theory. *Chem. Eur. J.* **2012**, *18* (32), 9955–9964. DOI: 10.1002/chem.201200497.
[19] Luchini, G.; Alegre-Requena, J.V.; Funes-Ardoiz, I.; Paton, R.S.; GoodVibes: automated thermochemistry for heterogeneous computational chemistry data. *F1000Research*. **2020**, *9* (291), 291. DOI: 10.12688/f1000research.22758.1.
[20] CYLview1.0; Legault, C. Y., Université de Sherbrooke, **2020** (http://www.cylview.org).

9. NMR Spectra

N-allyl-*N*-phenylbenzenesulfonamide (1a)

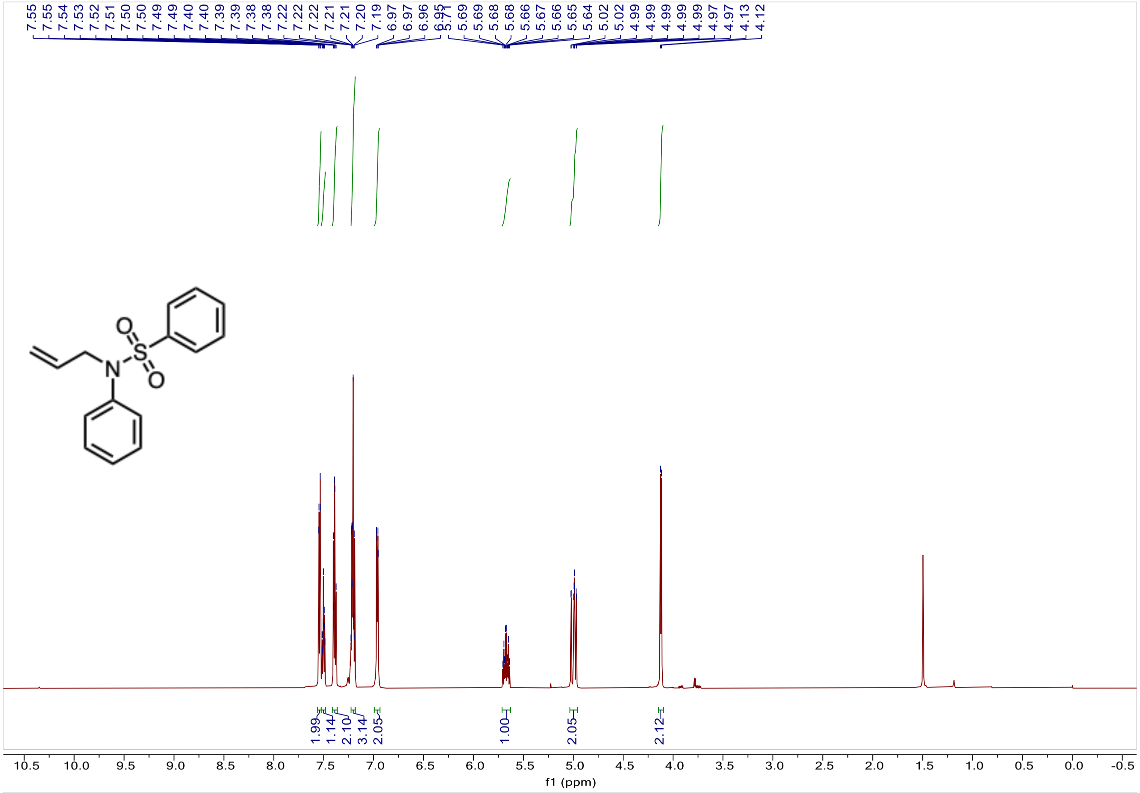

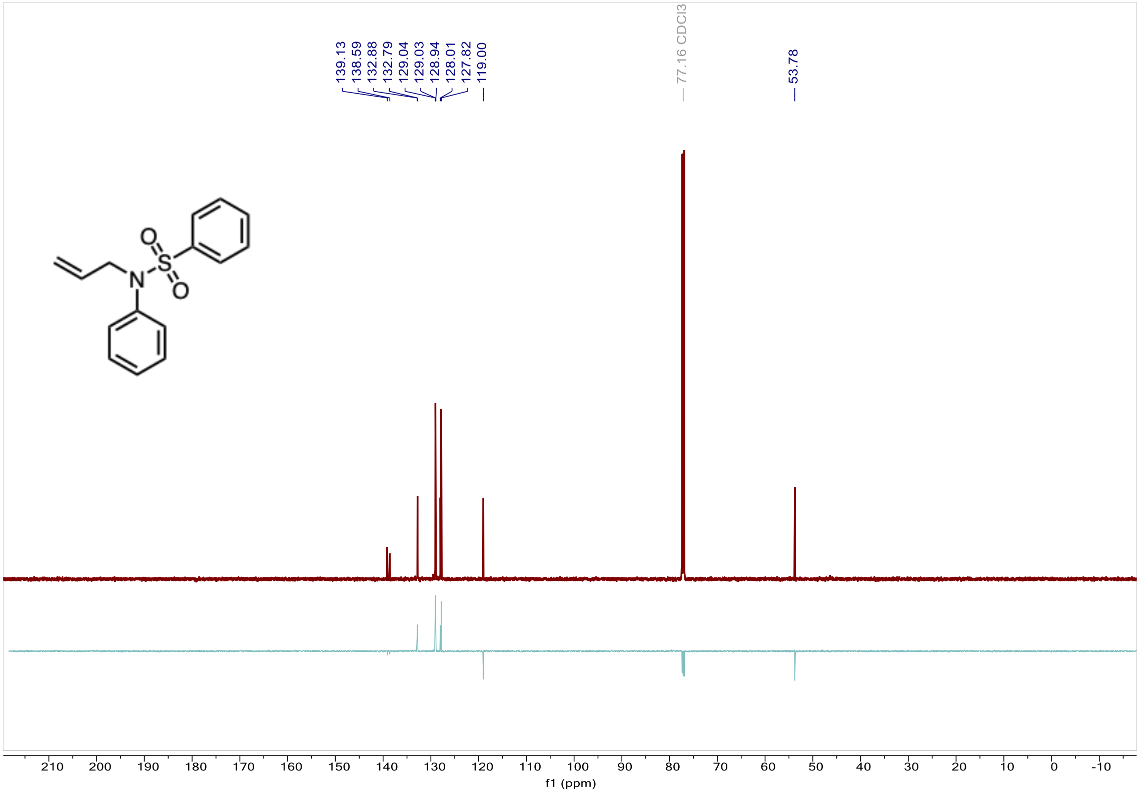

_N_-allyl-2,4,6-trimethyl-_N_-phenylbenzenesulfonamide (1b)

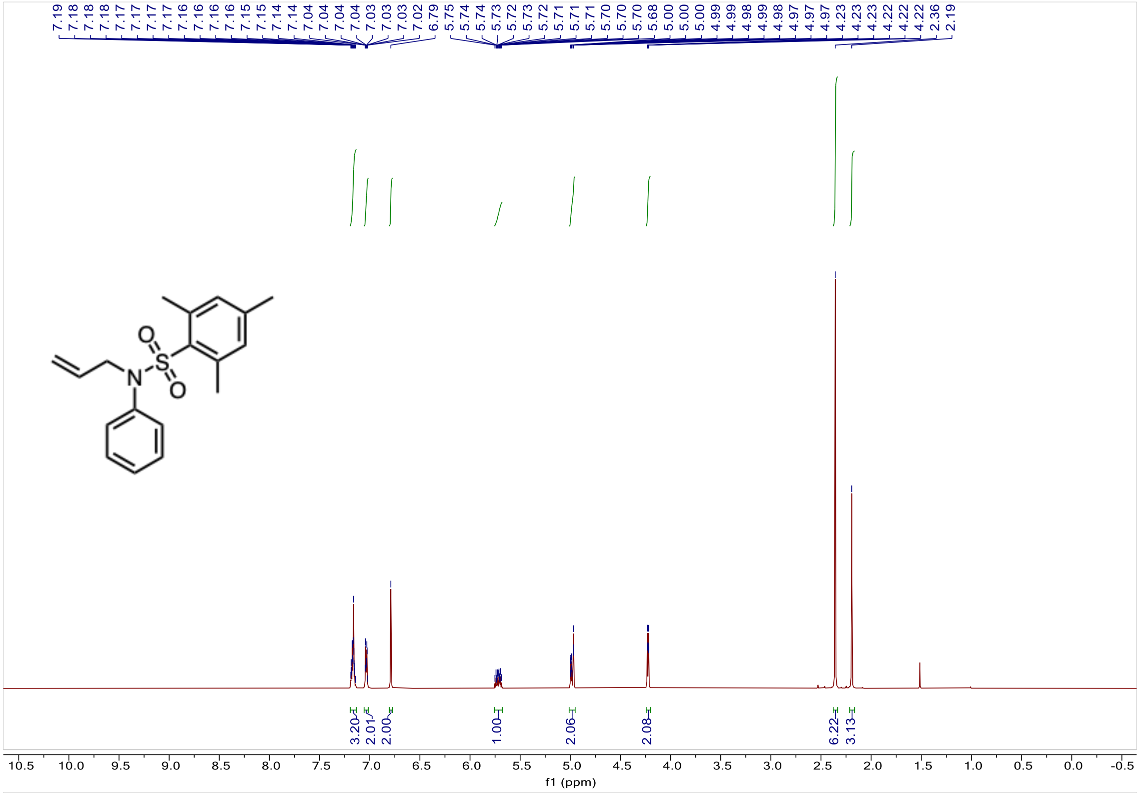

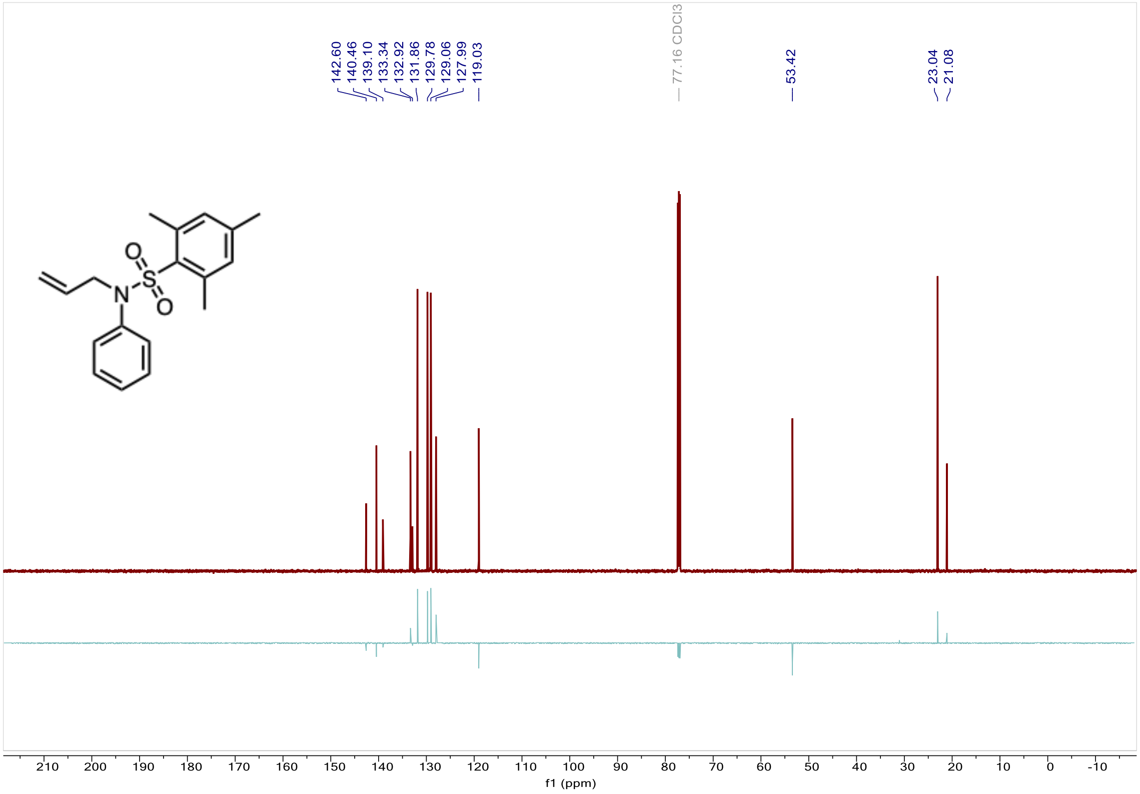

N-allyl-2-methyl-*N*-phenylbenzenesulfonamide (1c)

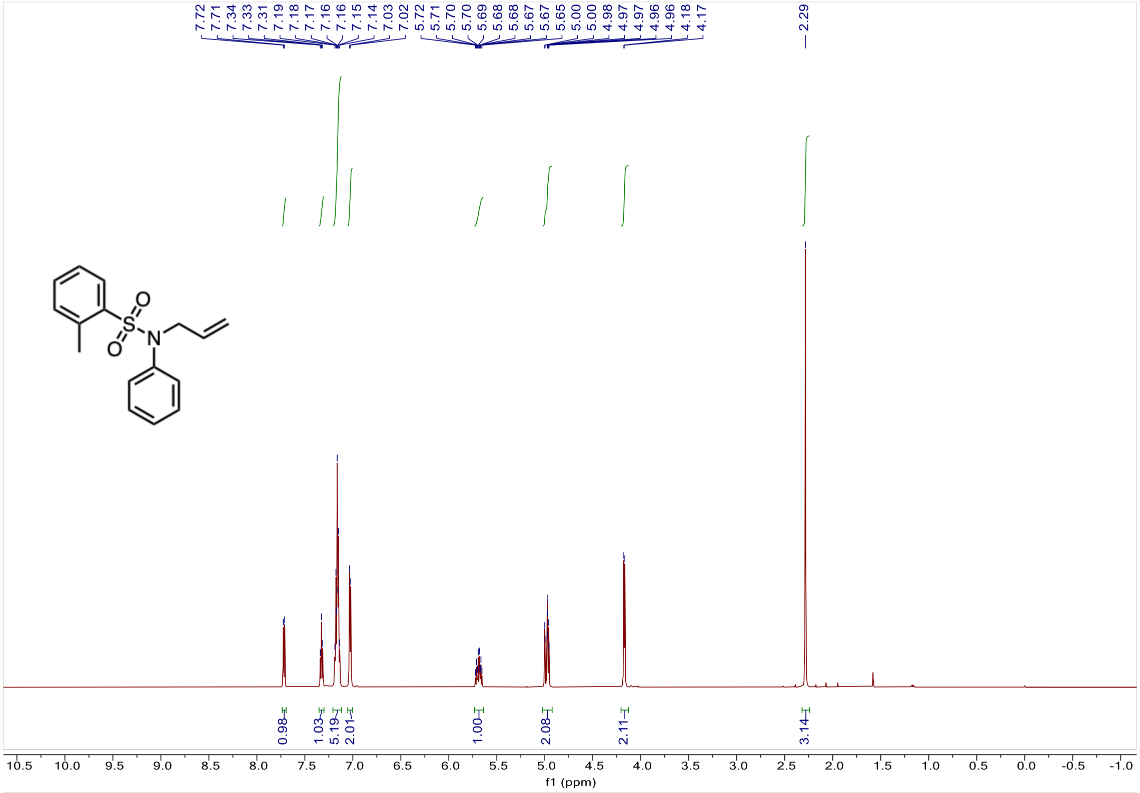

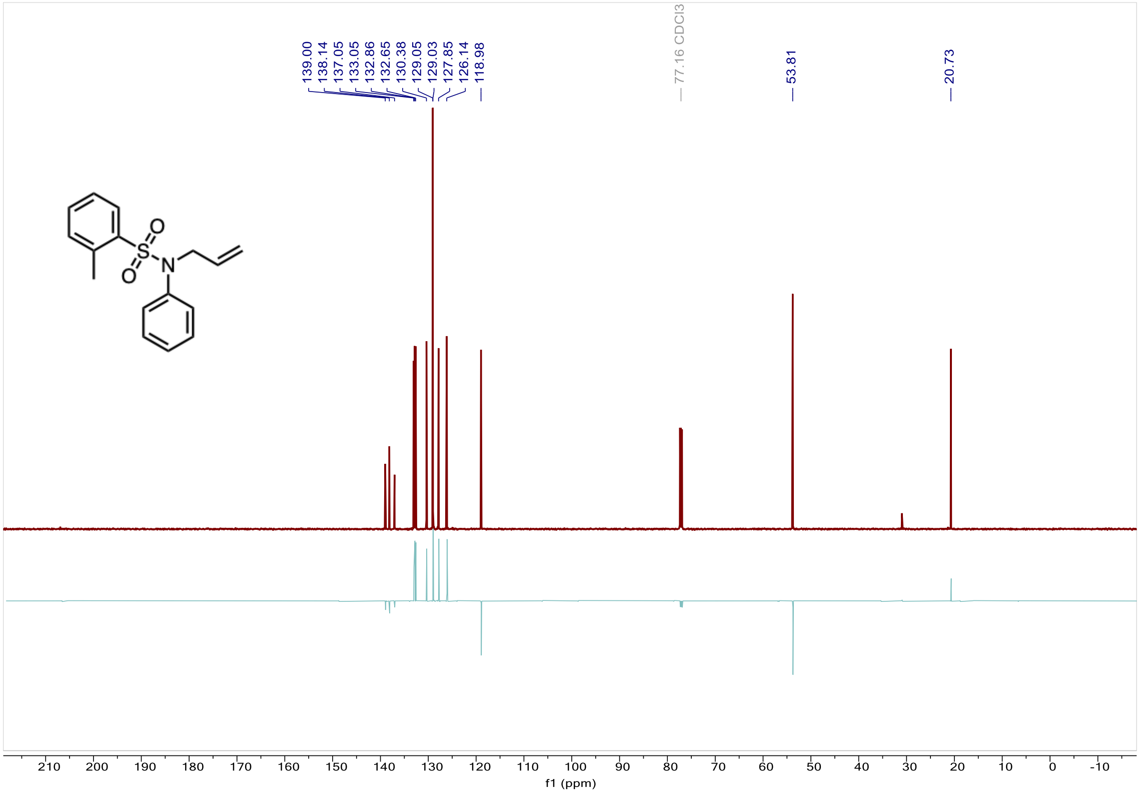

N-allyl-2-methoxy-N-phenylbenzenesulfonamide (1d)

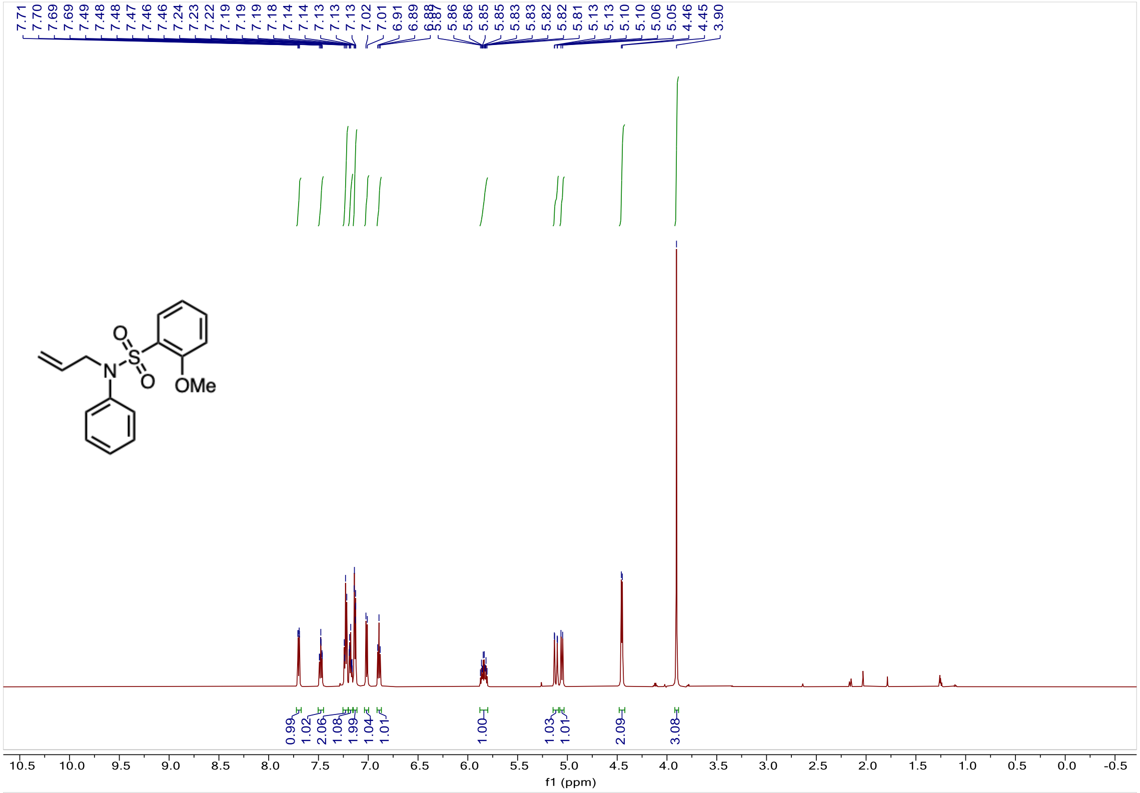

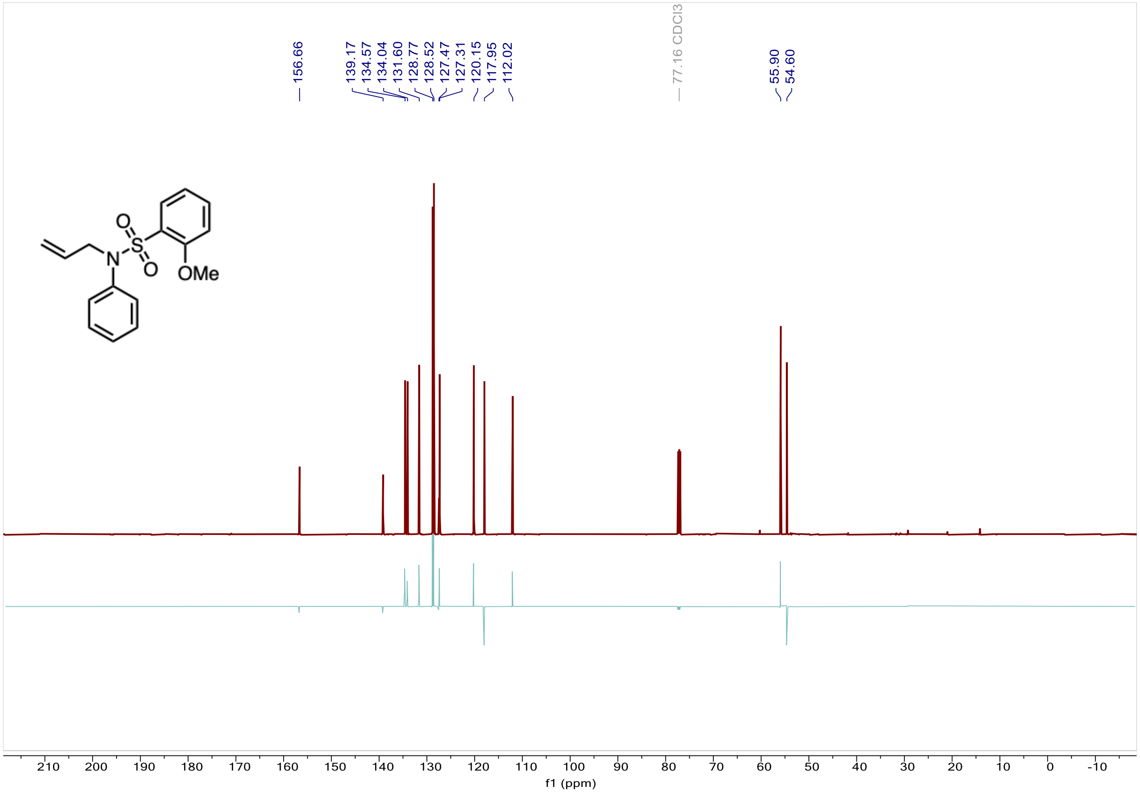

N-allyl-2-chloro-*N*-phenylbenzenesulfonamide (1e)

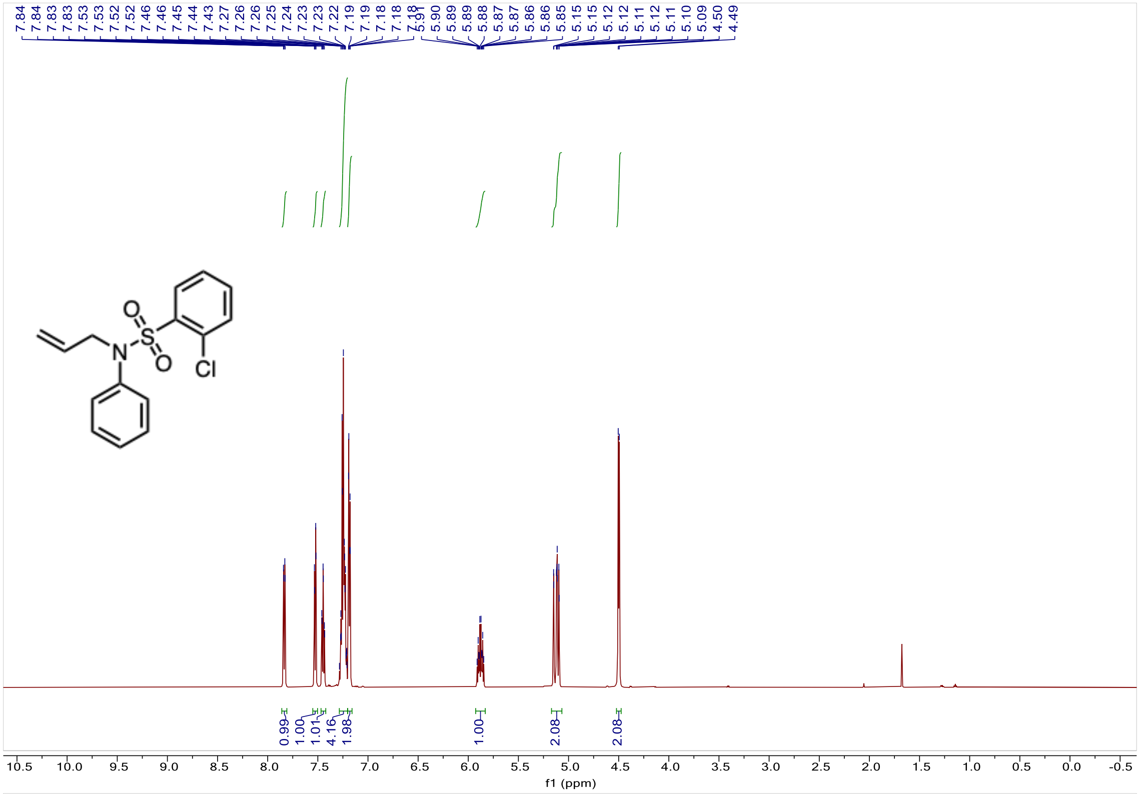

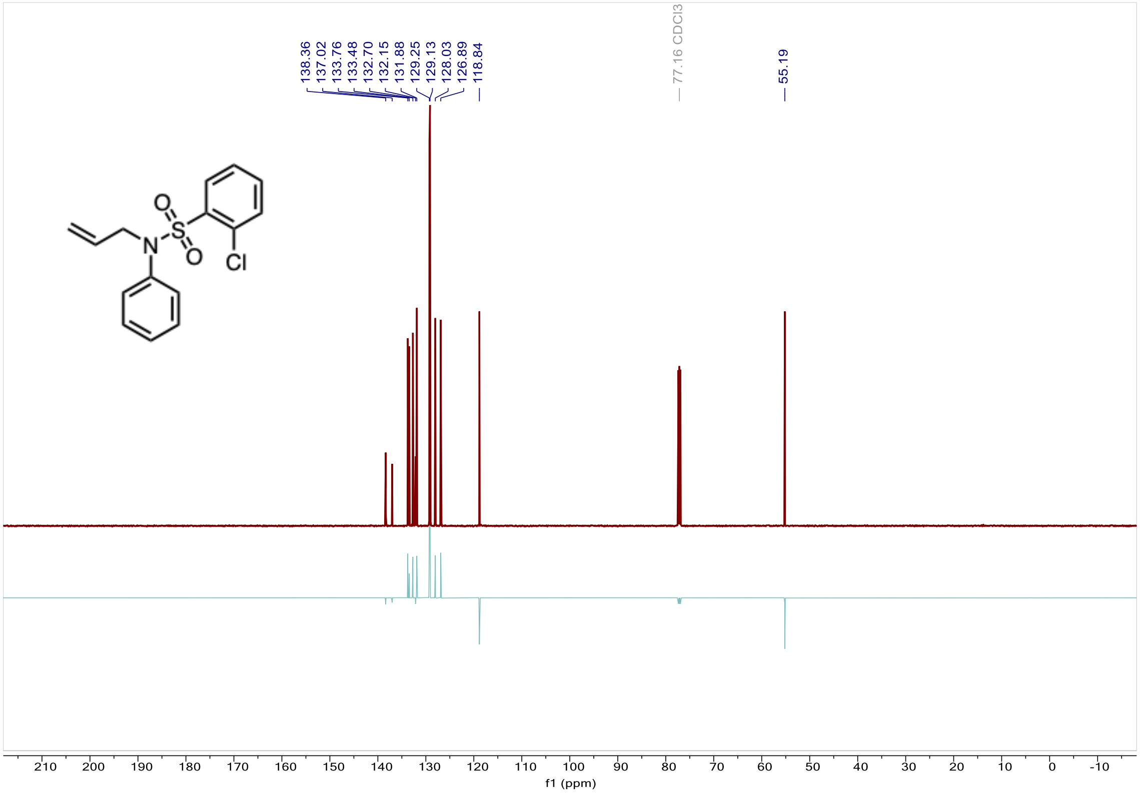

N-allyl-2-bromo-*N*-phenylbenzenesulfonamide (1f)

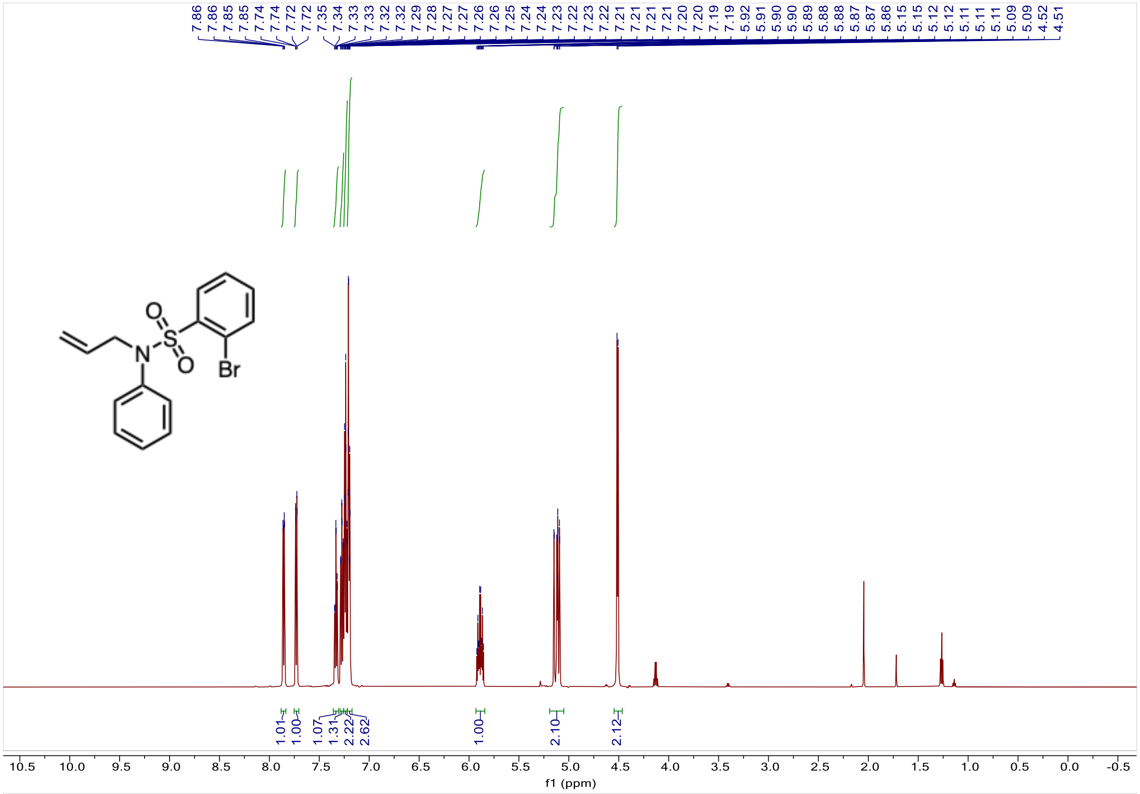

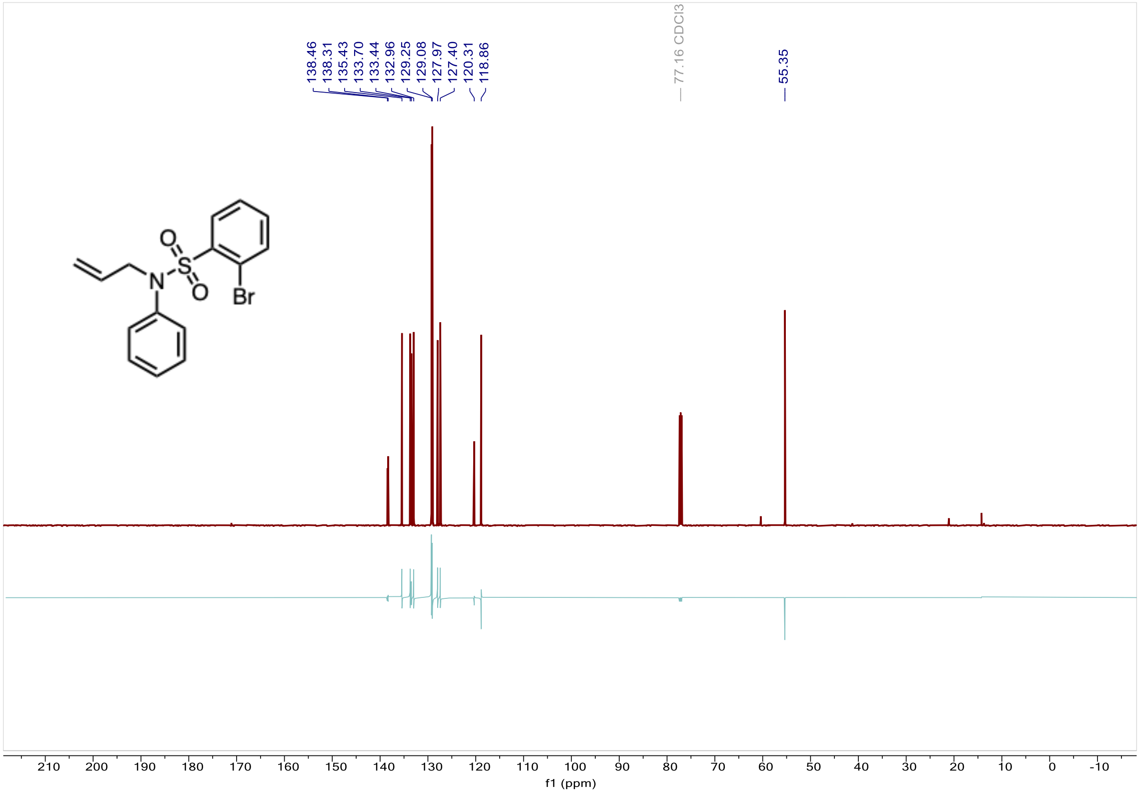

N-allyl-*N*-phenyl-2-(trifluoromethyl) benzenesulfonamide (1g)

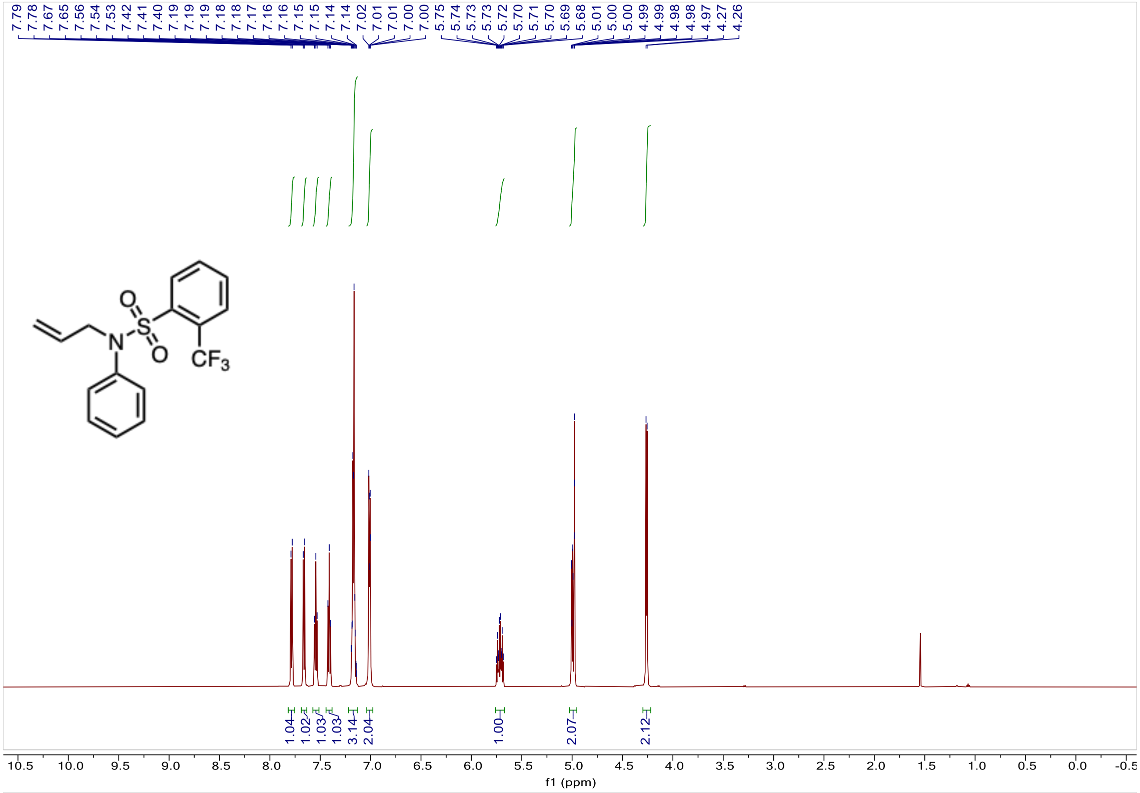

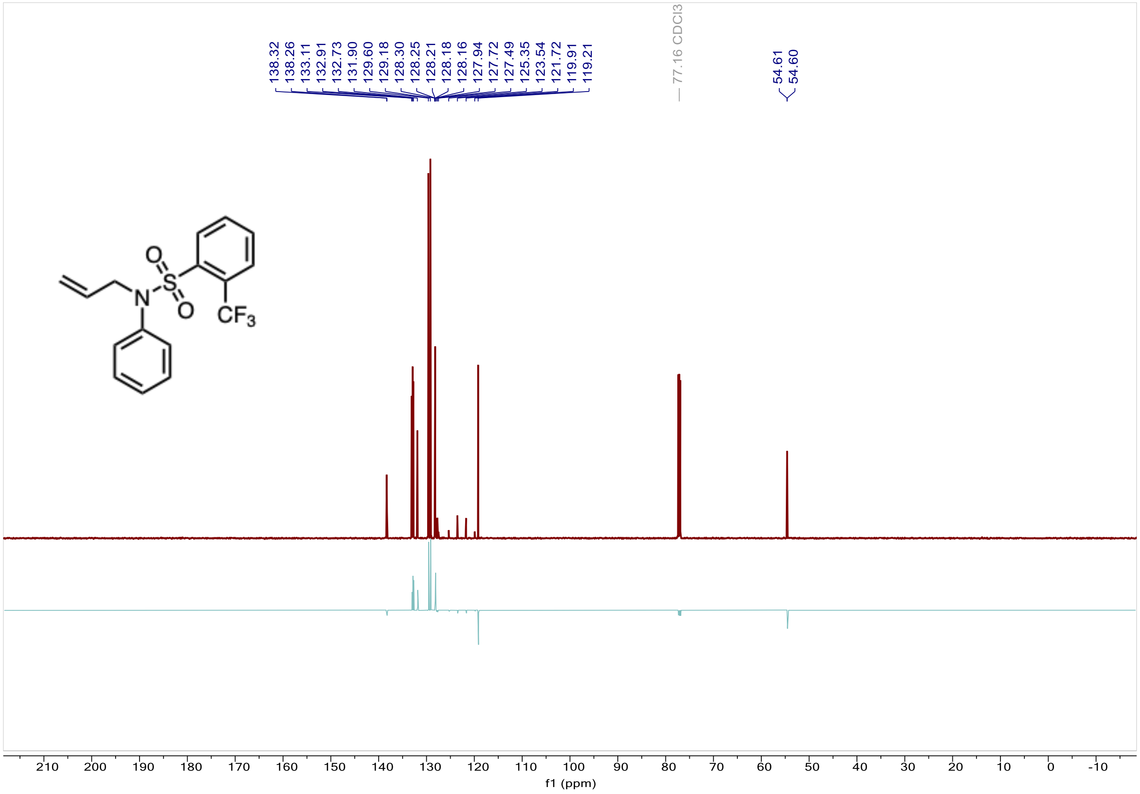

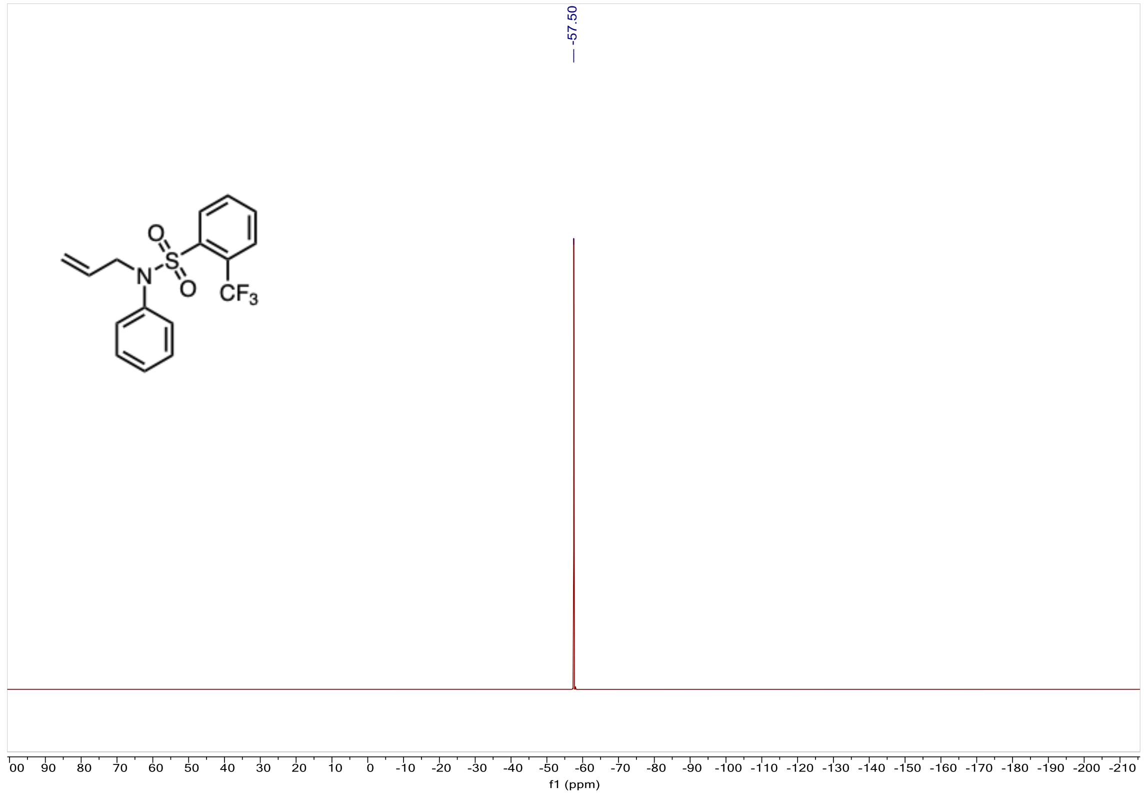

methyl 2-(*N*-allyl-*N*-phenylsulfamoyl) benzoate (1h)

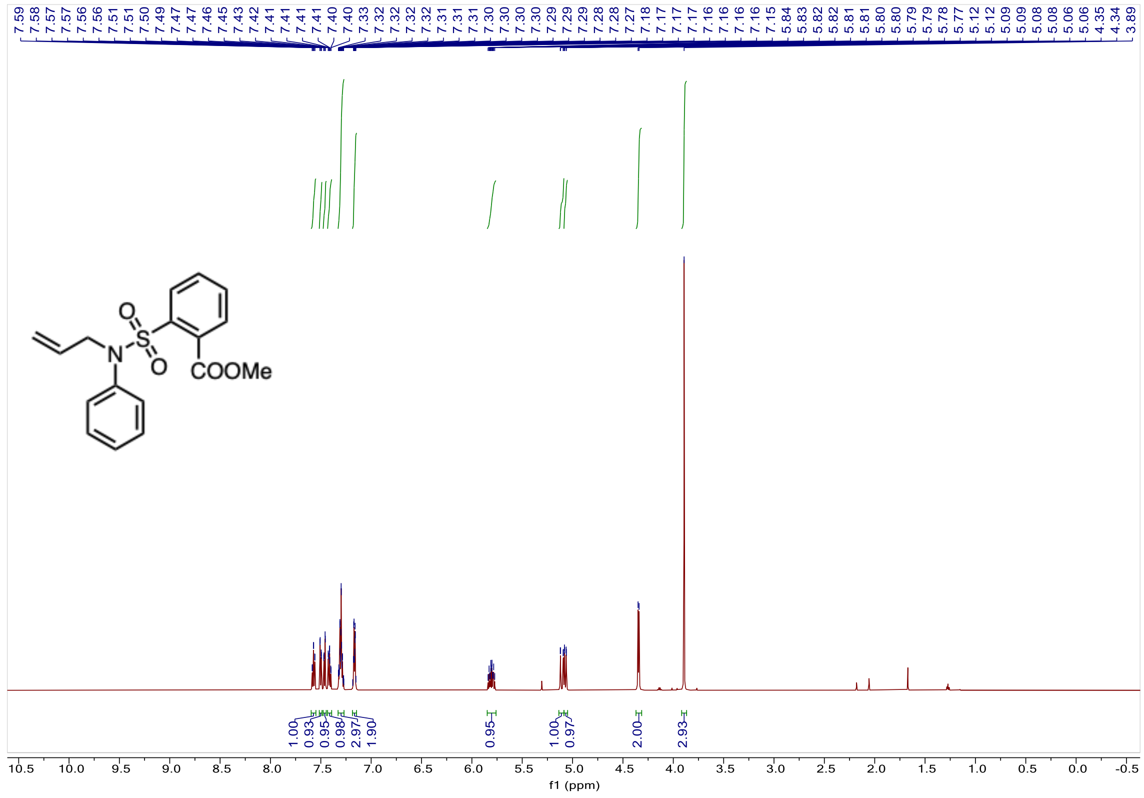

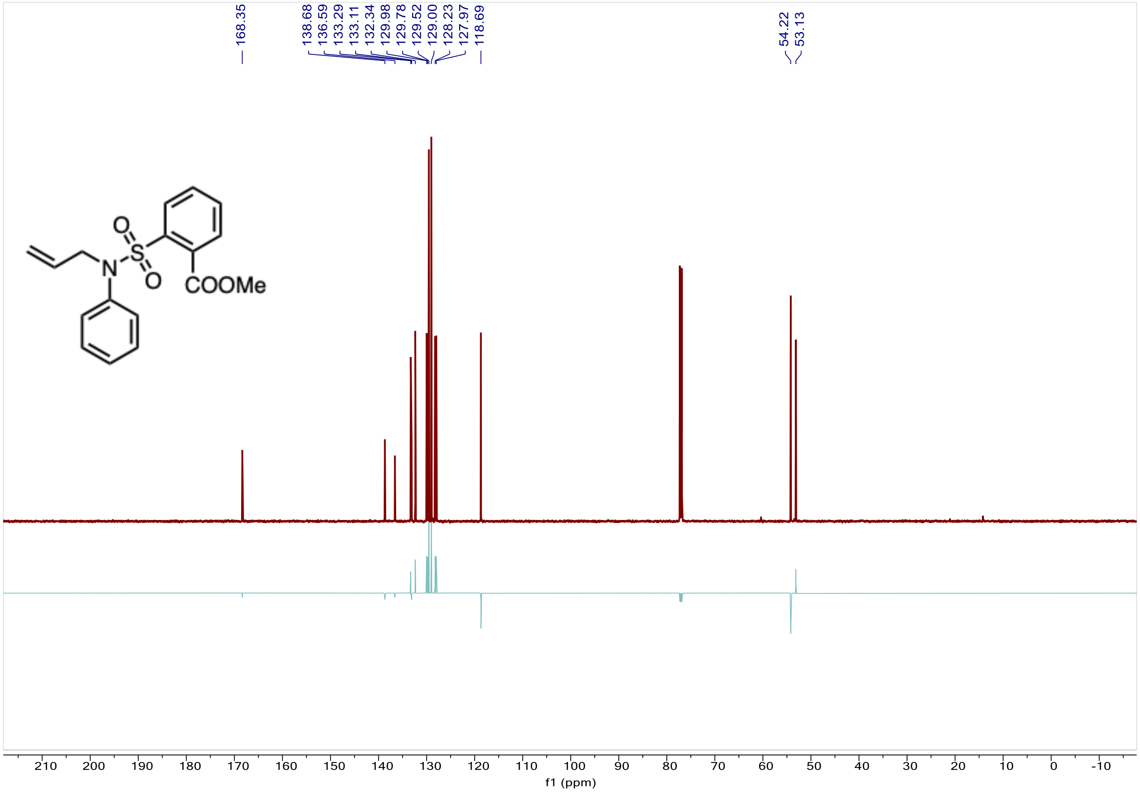

N-allyl-2,6-dimethoxy-*N*-phenylbenzenesulfonamide (1i)

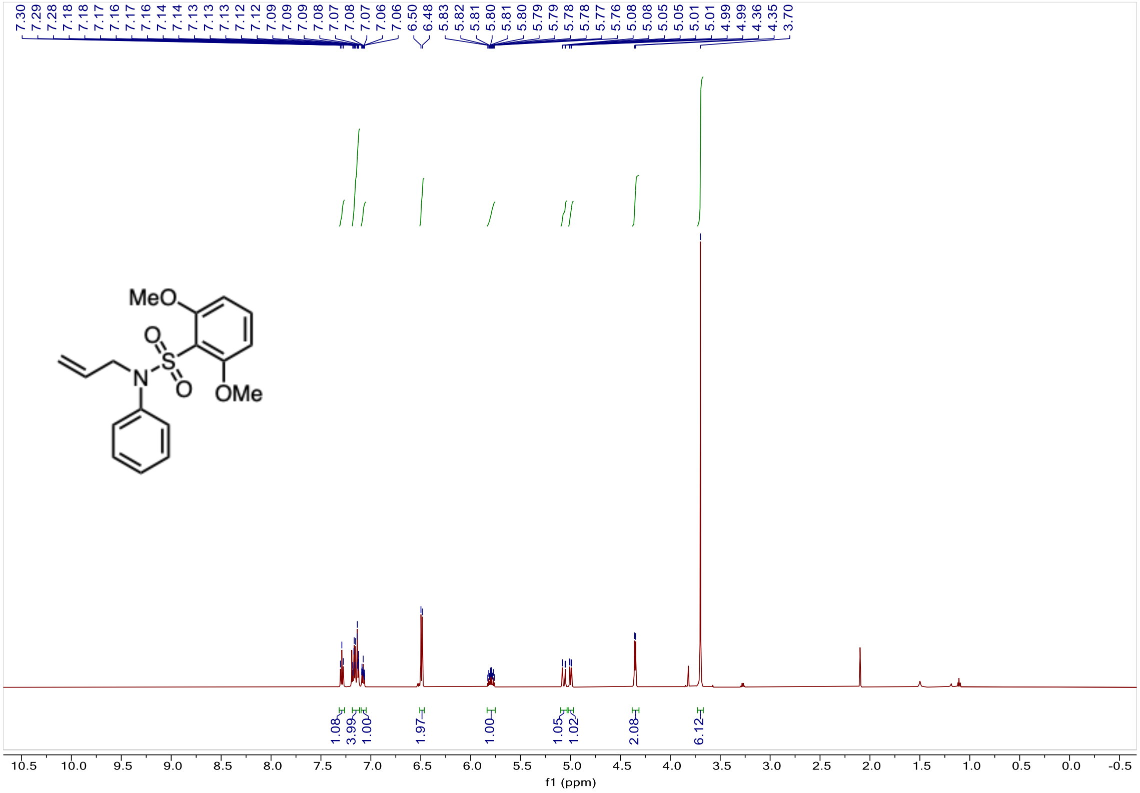

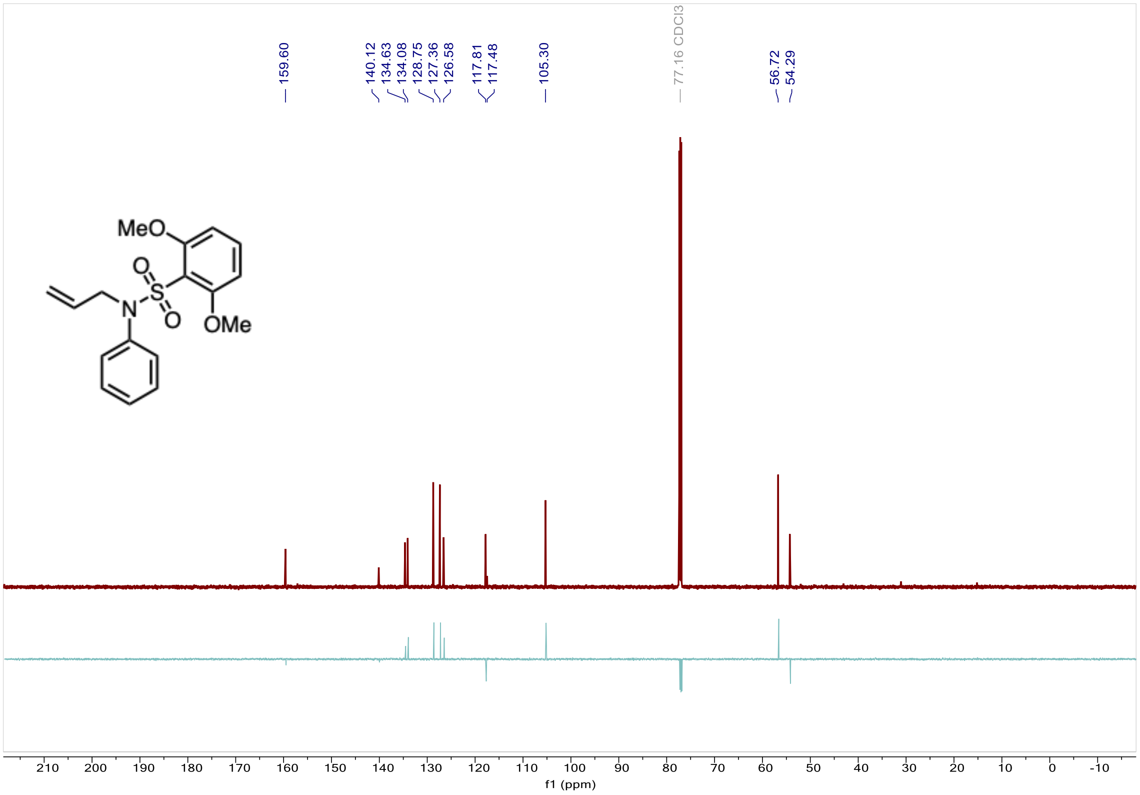

N-allyl-2,6-dichloro-*N*-phenylbenzenesulfonamide (1j)

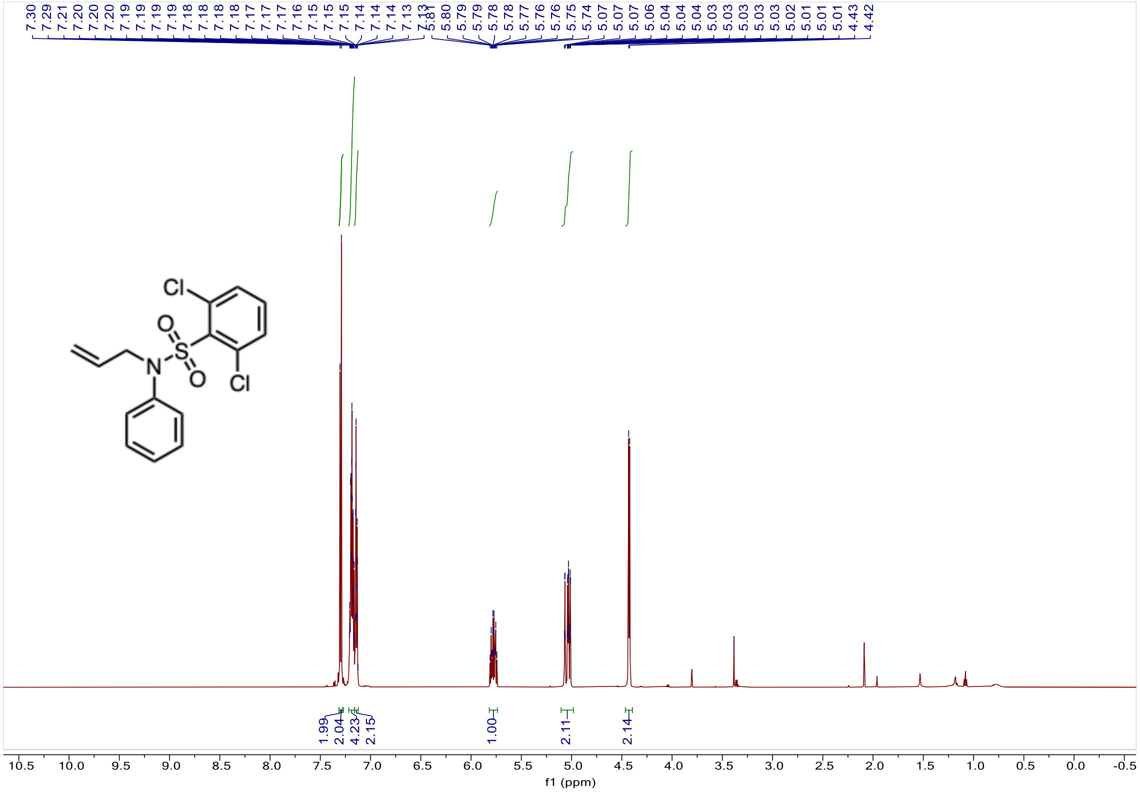

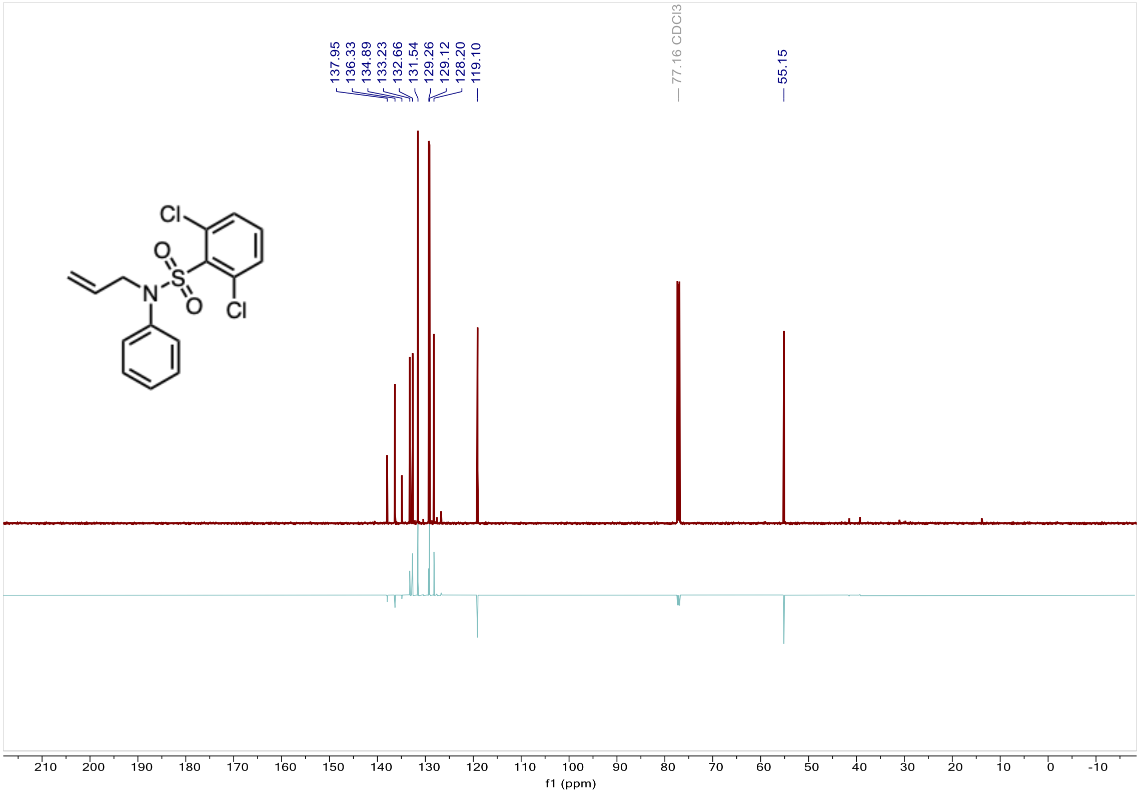

N-allyl-2,5-dimethyl-*N*-phenylbenzenesulfonamide (1k)

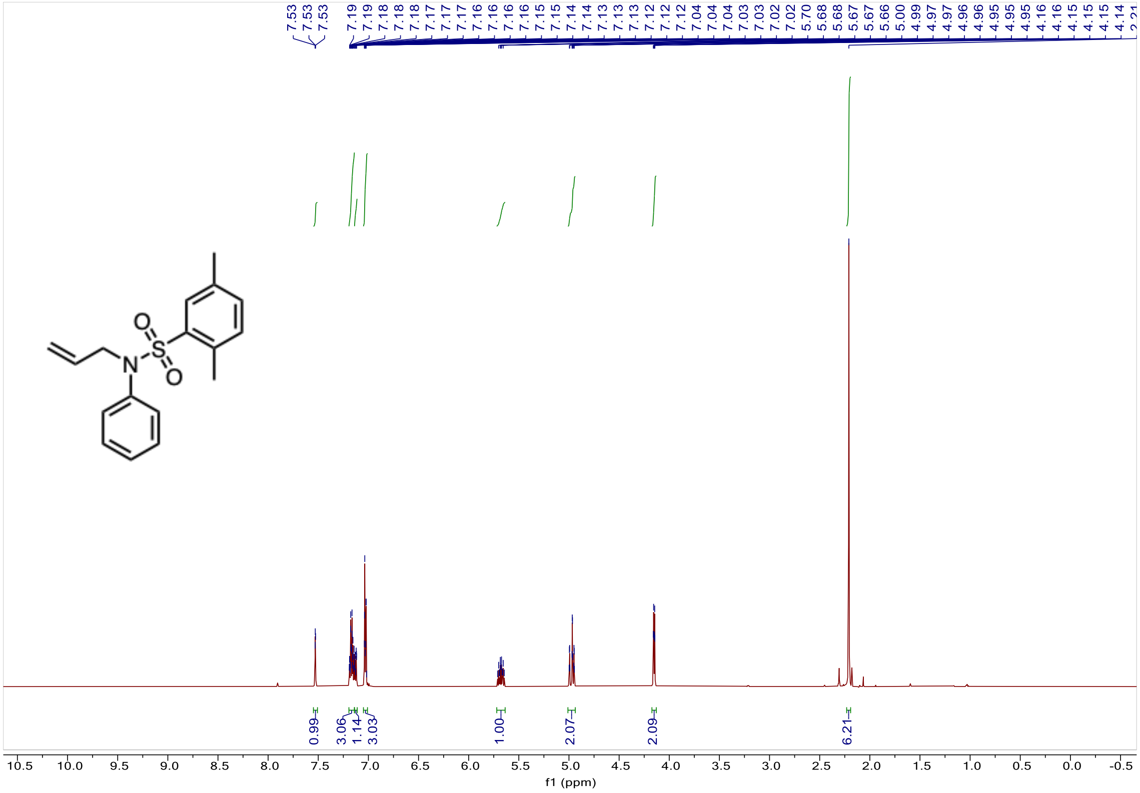

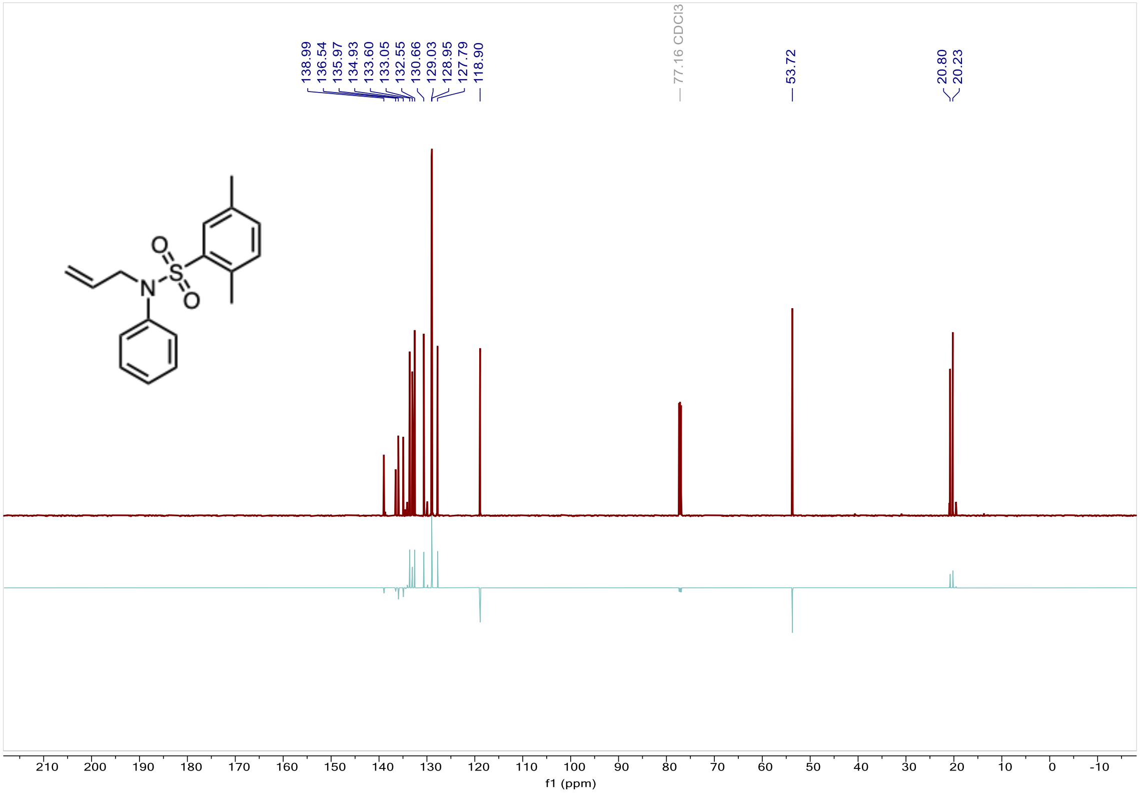

N-allyl-2,4-dimethyl-*N*-phenylbenzenesulfonamide (1l)

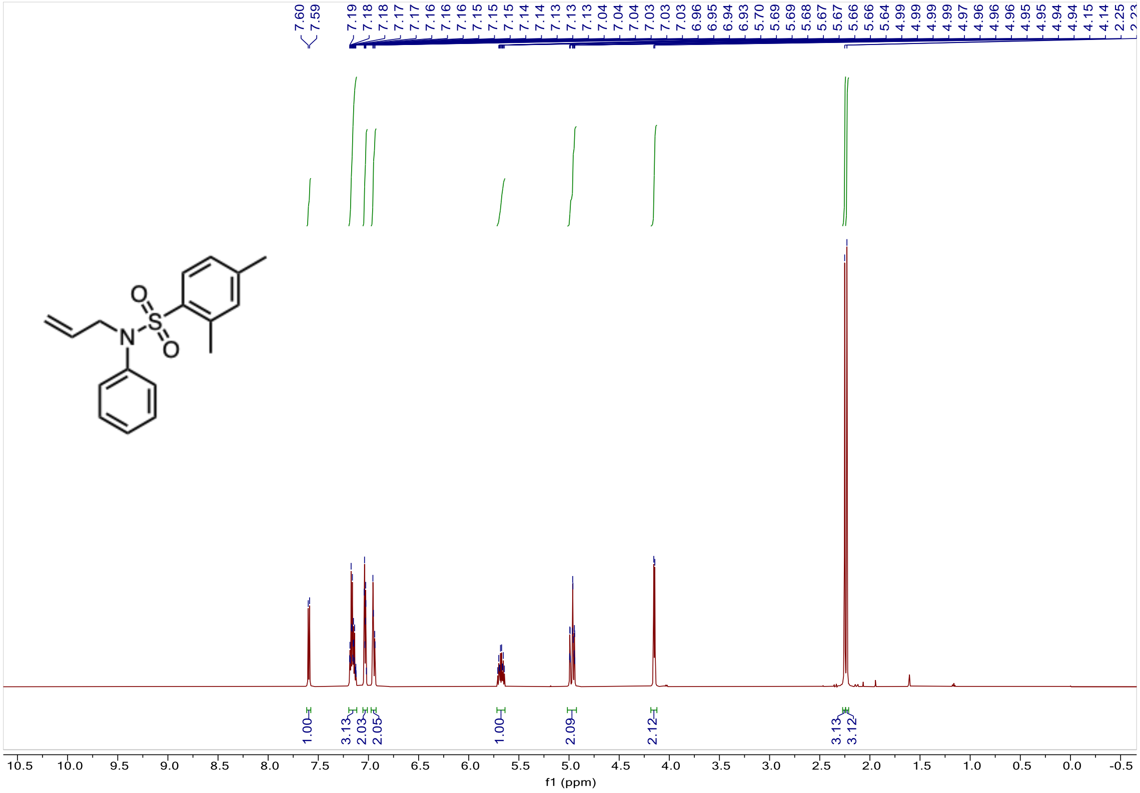

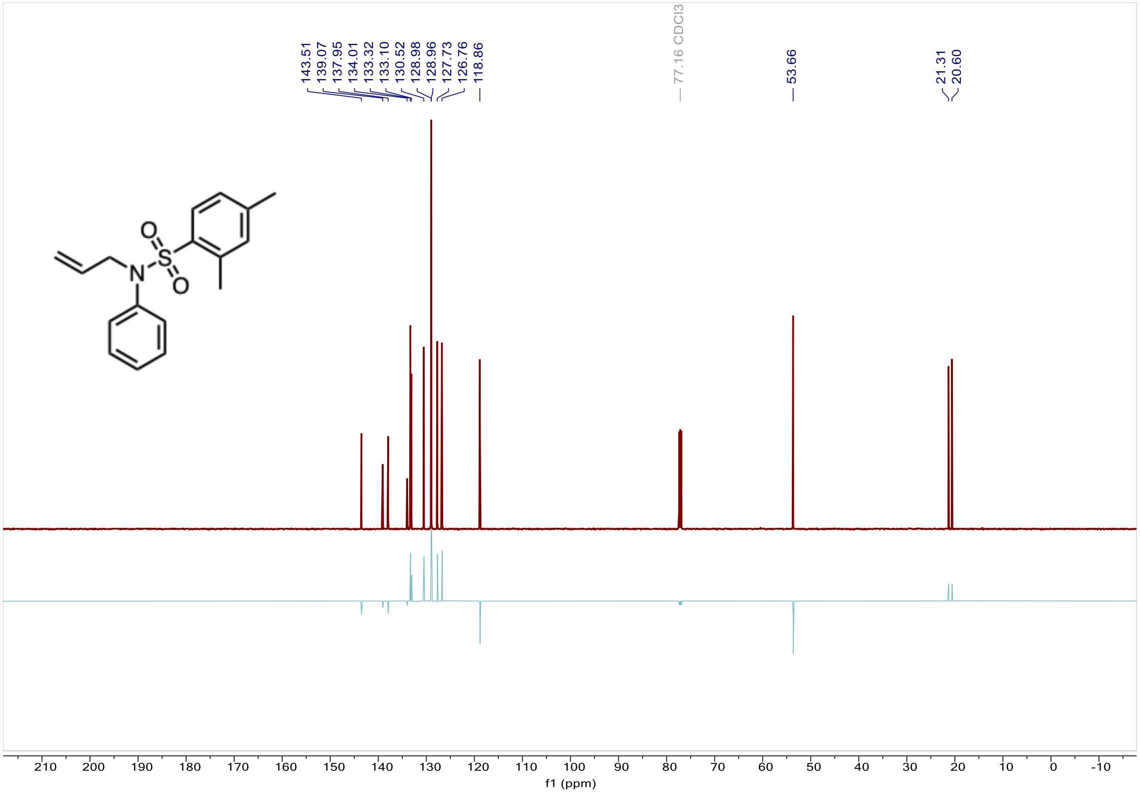

N-allyl-2,3,4,5,6-pentamethyl-*N*-phenylbenzenesulfonamide (1m)

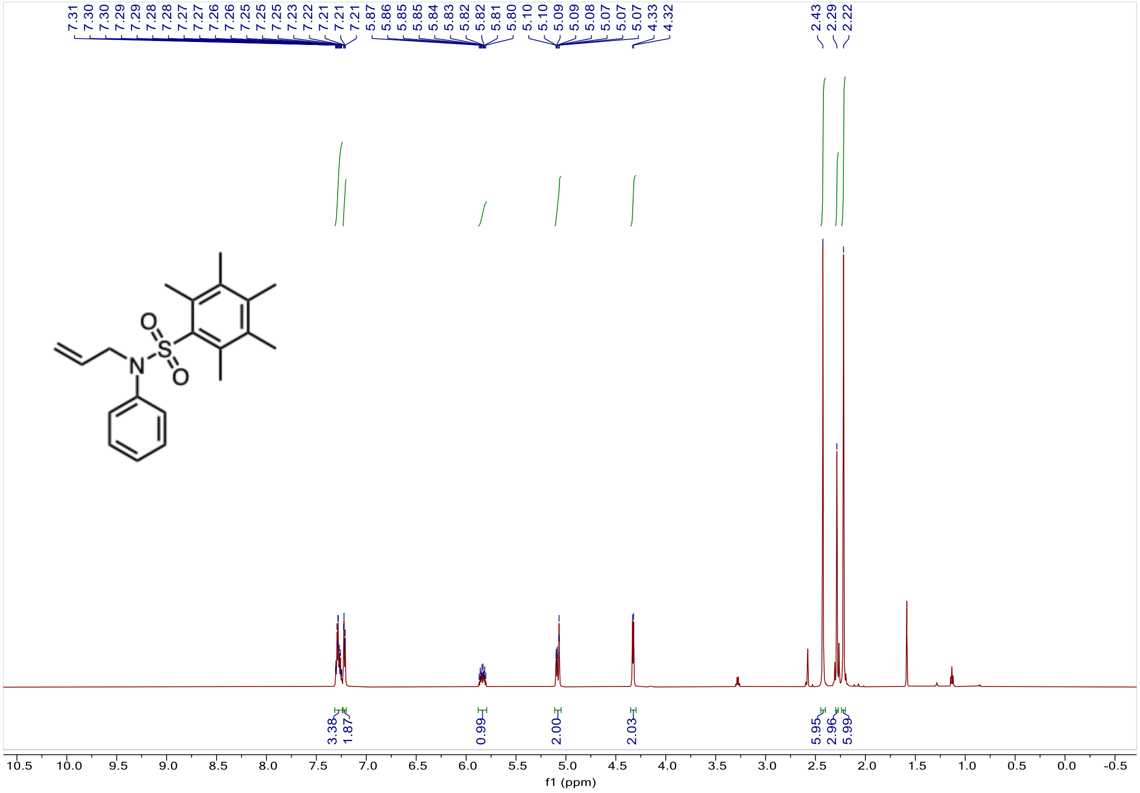

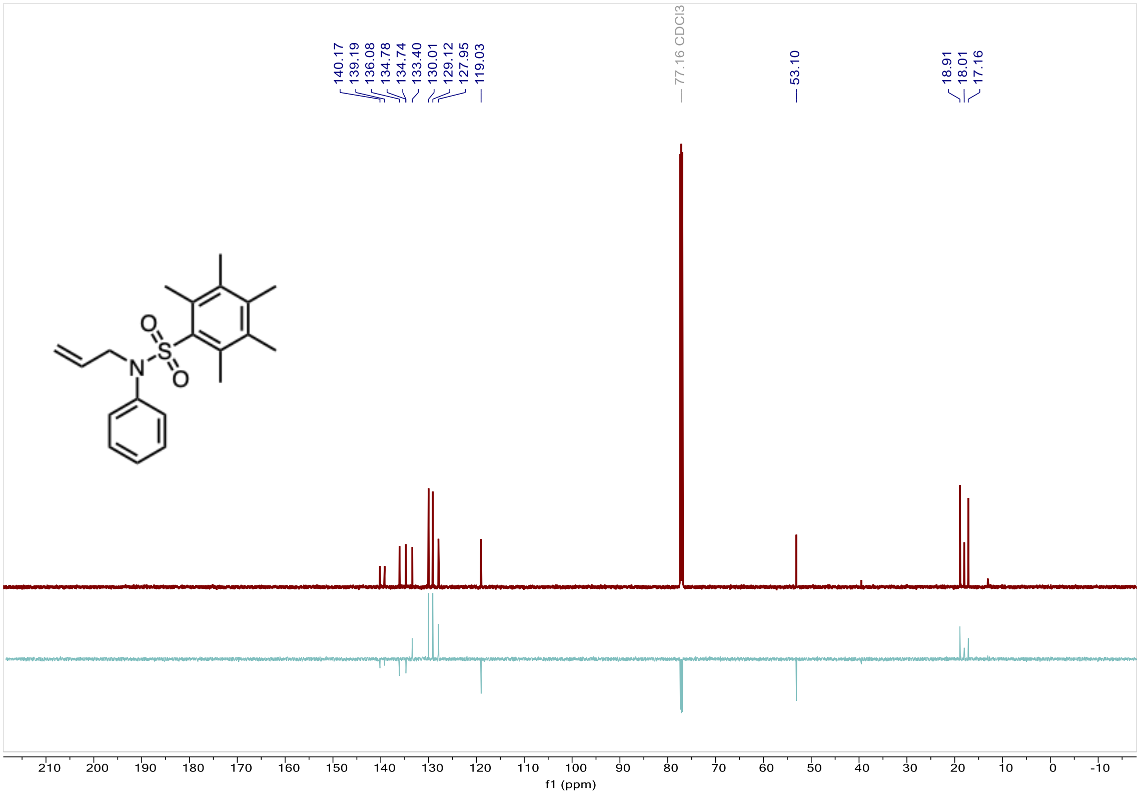

N-allyl-2,4,6-triisopropyl-*N*-phenylbenzenesulfonamide (1n)

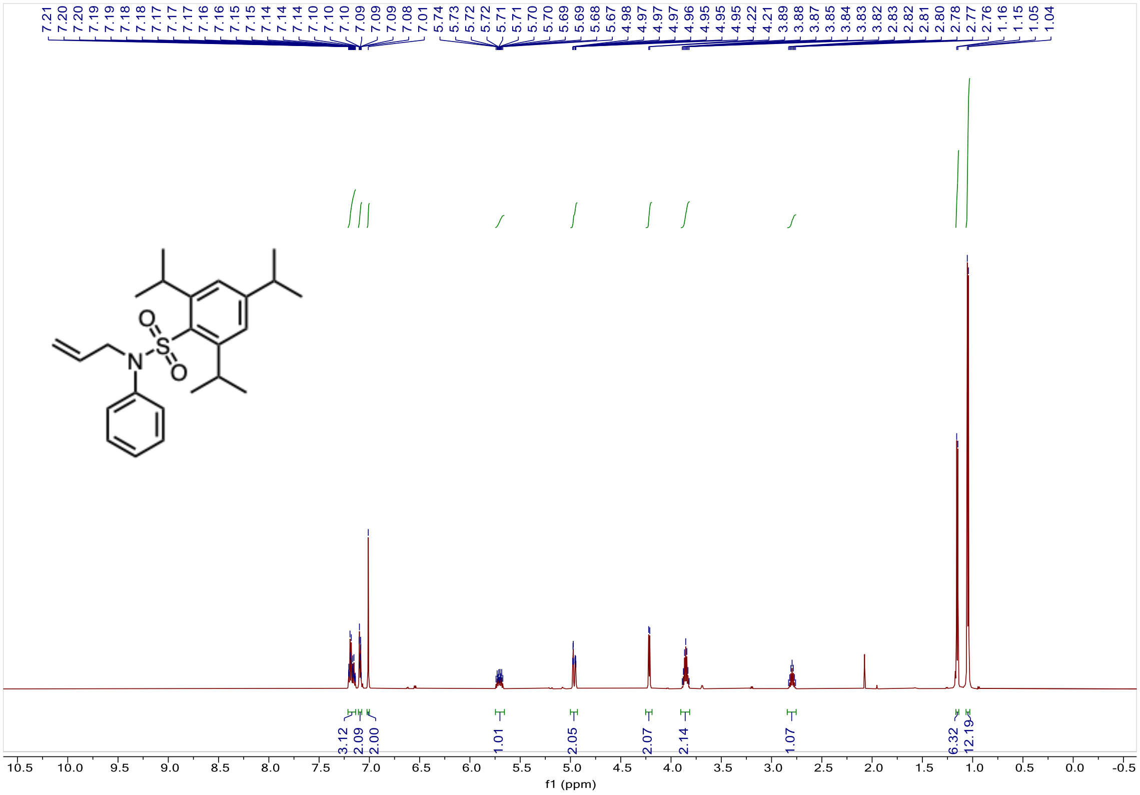

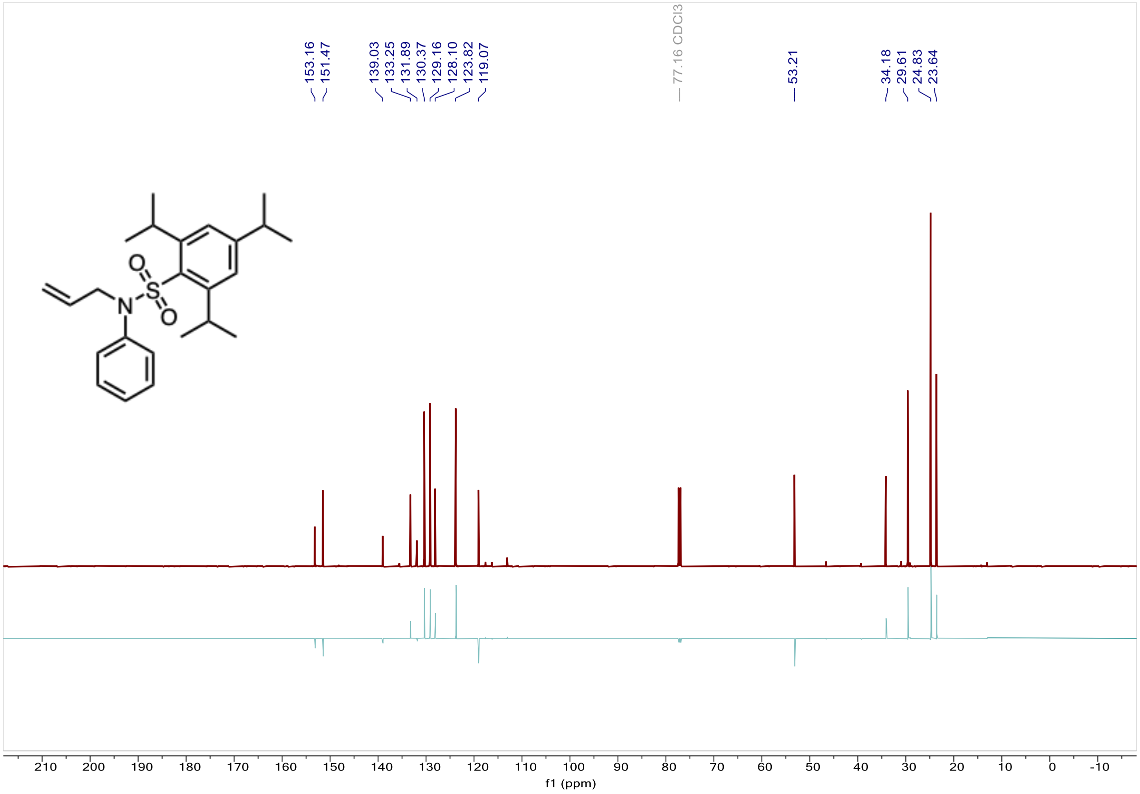

N-allyl-4-methoxy-*N*-phenylbenzenesulfonamide (1o)

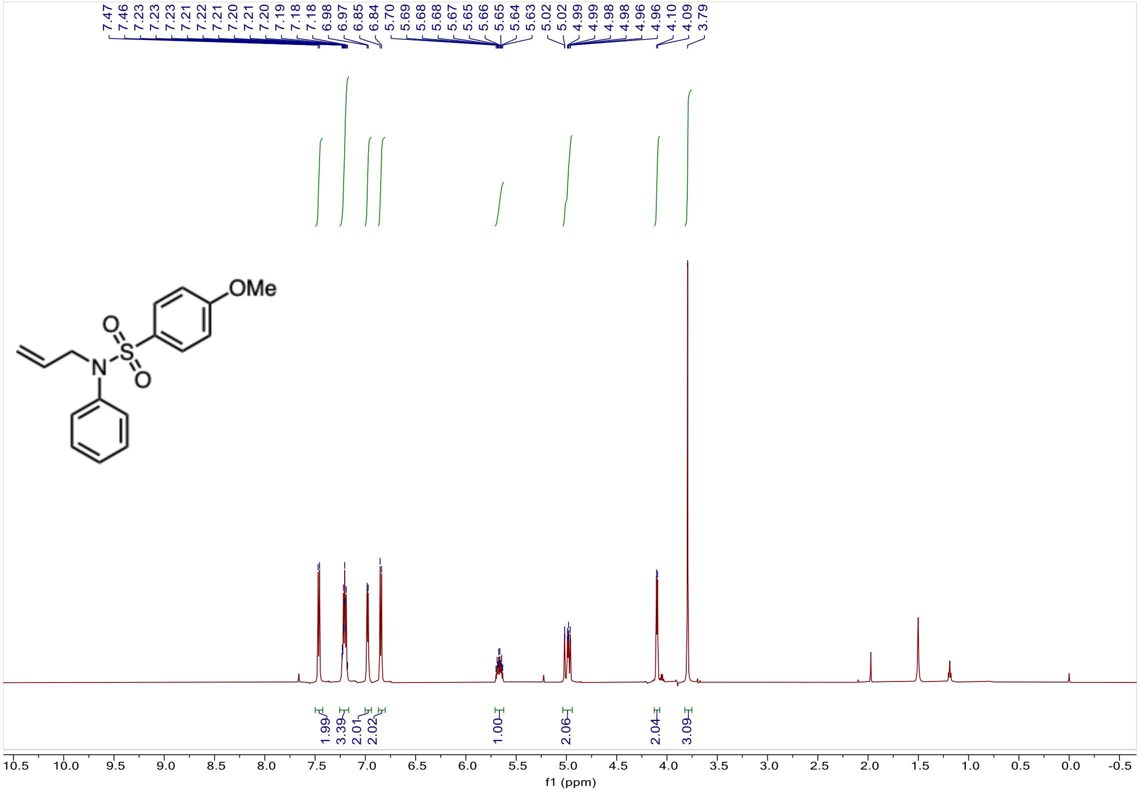

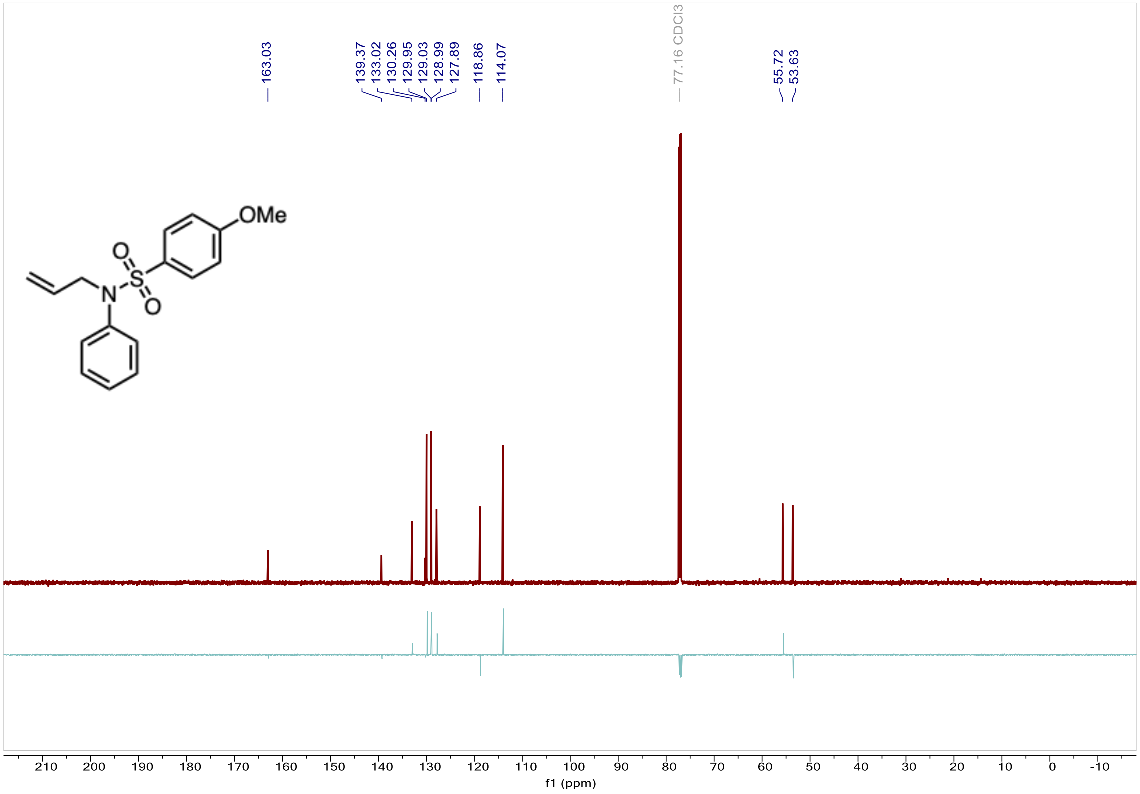

N-allyl-*N*-phenyl-4-(trifluoromethyl)benzenesulfonamide (1p)

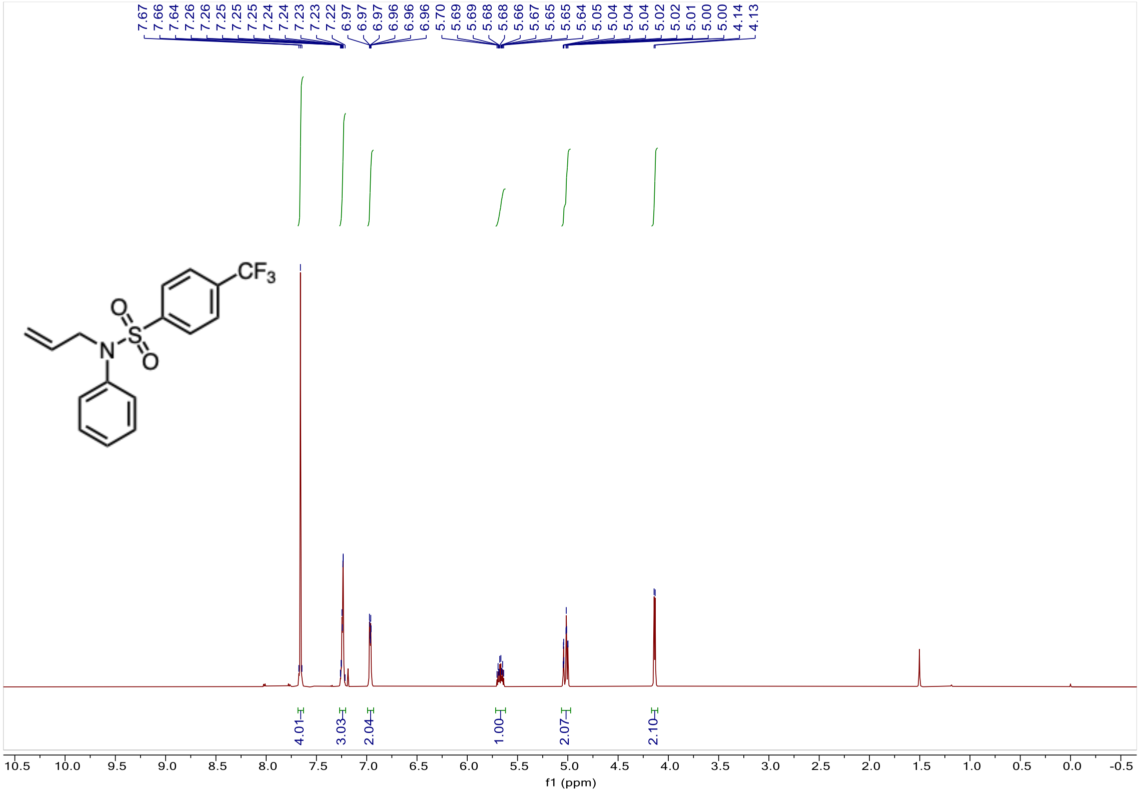

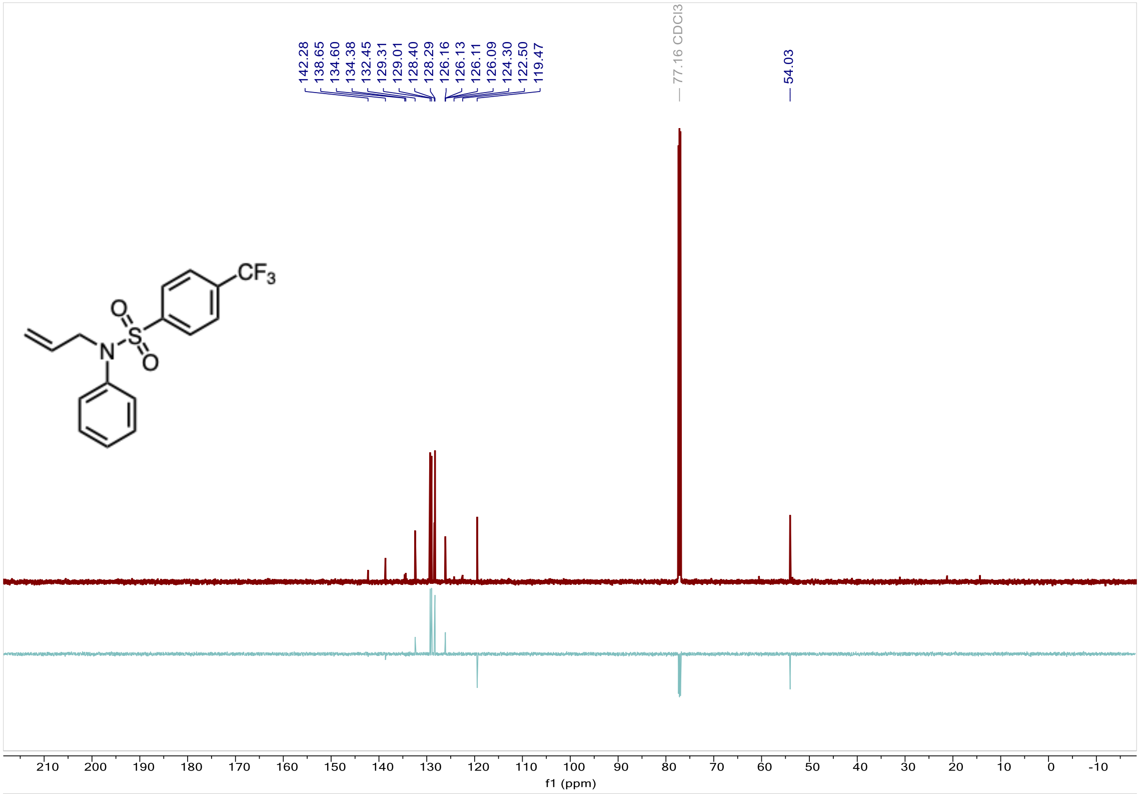

142.28
138.65
134.60
134.38
132.45
129.31
129.01
128.40
128.29
126.16
126.13
126.11
126.09
124.30
122.50
119.47
77.16 CDCl3
54.03
CF3
O
S
O
N
210
200
190
180
170
160
150
140
130
120
110
100
90
80
70
60
50
40
30
20
10
0
-10
f1 (ppm)

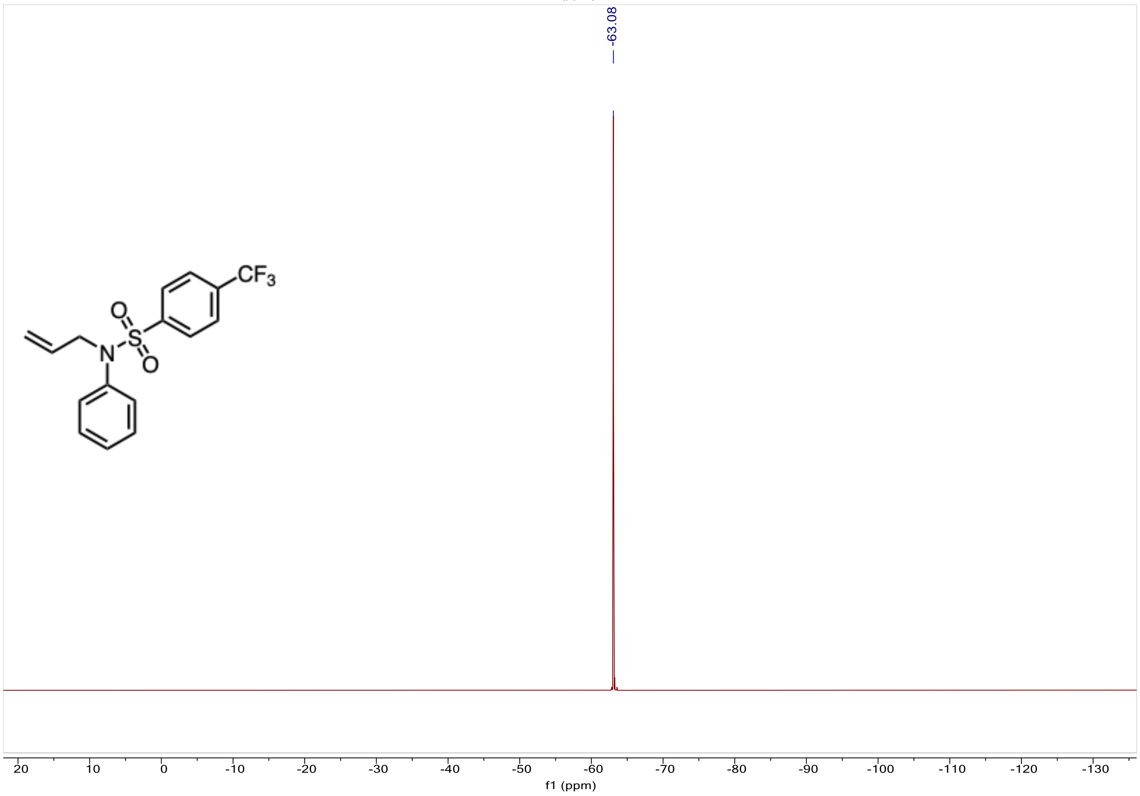

-63.08
CF3
O
S
O
N
20
10
0
-10
-20
-30
-40
-50
-60
-70
-80
-90
-100
-110
-120
-130
f1 (ppm)

N-allyl-3-methyl-*N*-phenylbenzenesulfonamide (1q)

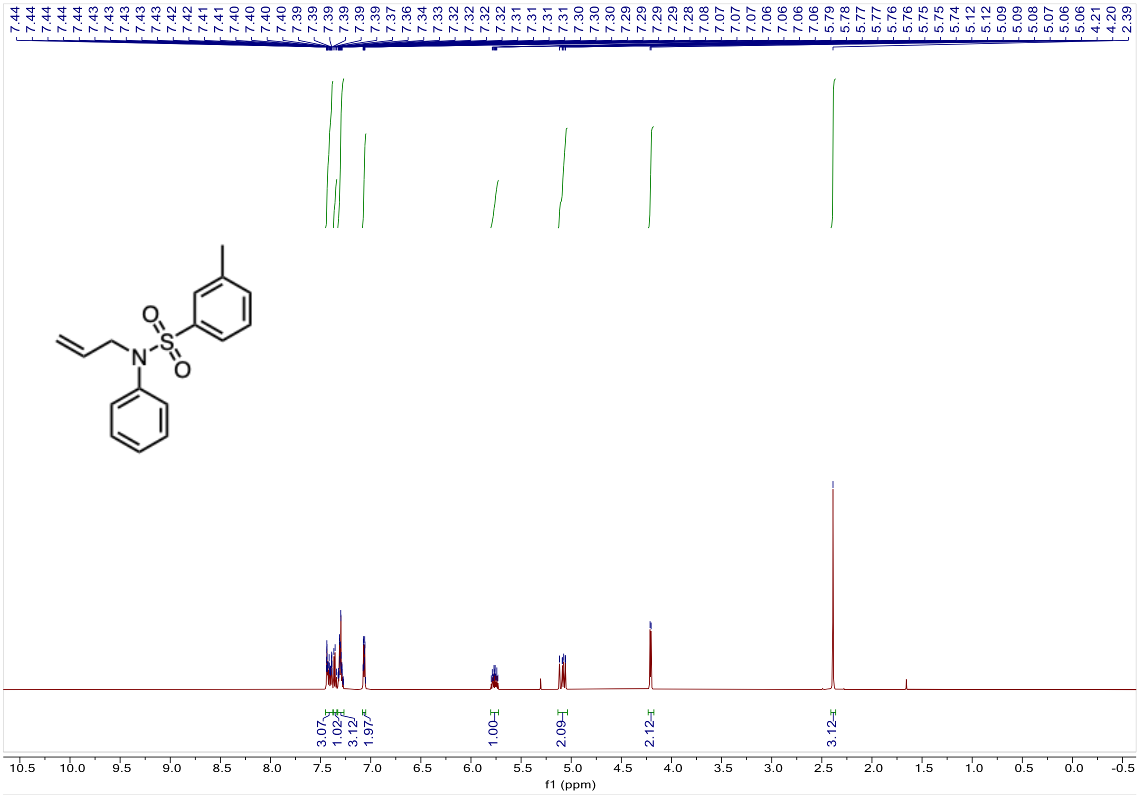

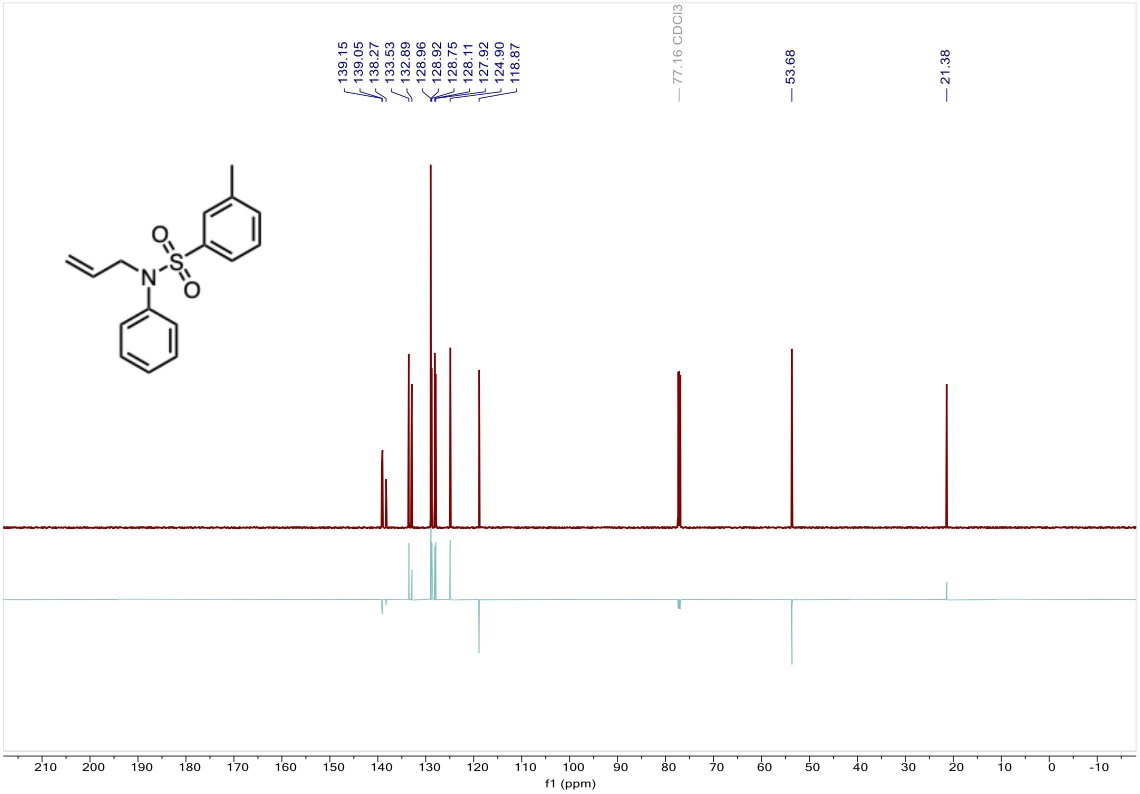

N-allyl-*N*-phenylnaphthalene-1-sulfonamide (1r)

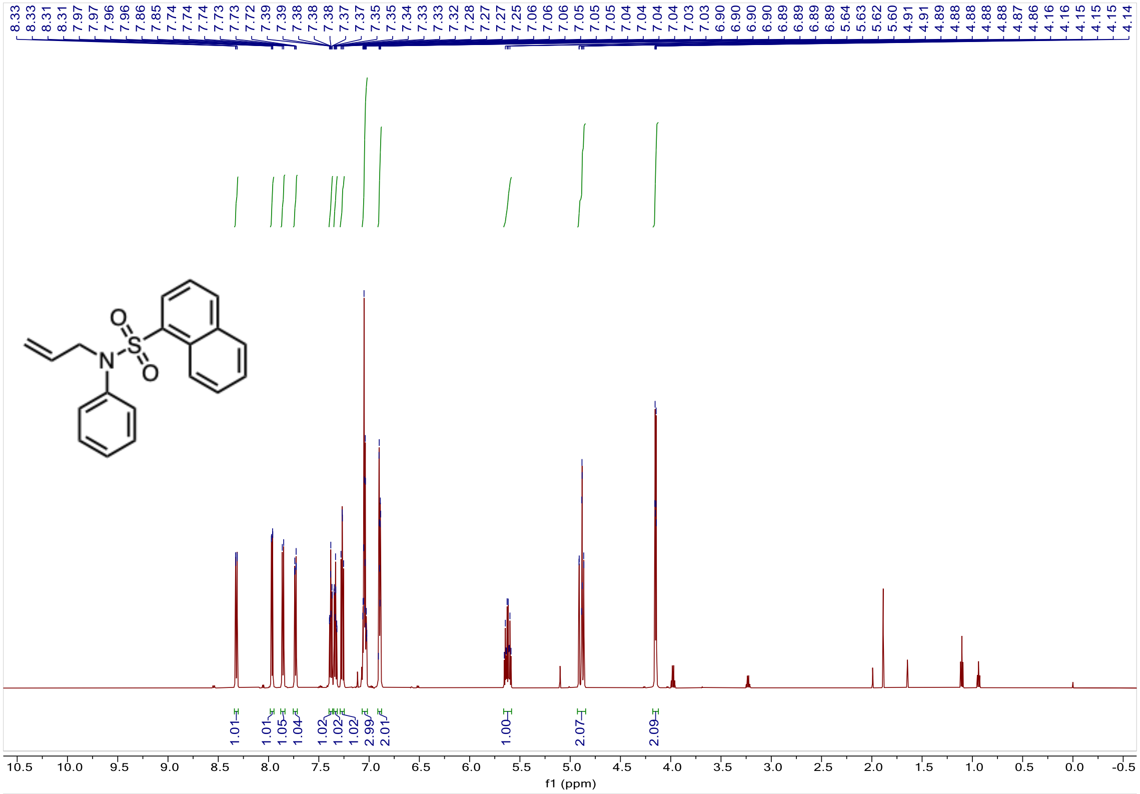

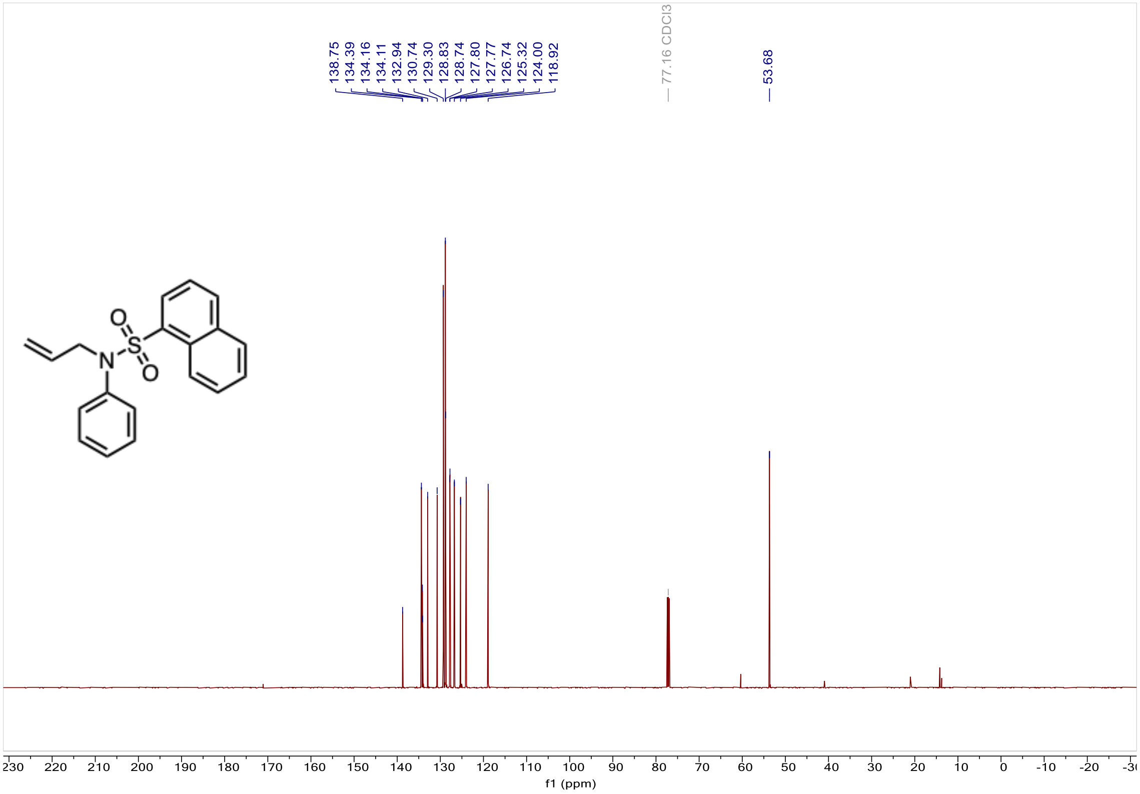

***N*-allyl-*N*-phenylquinoline-8-sulfonamide (1s)**

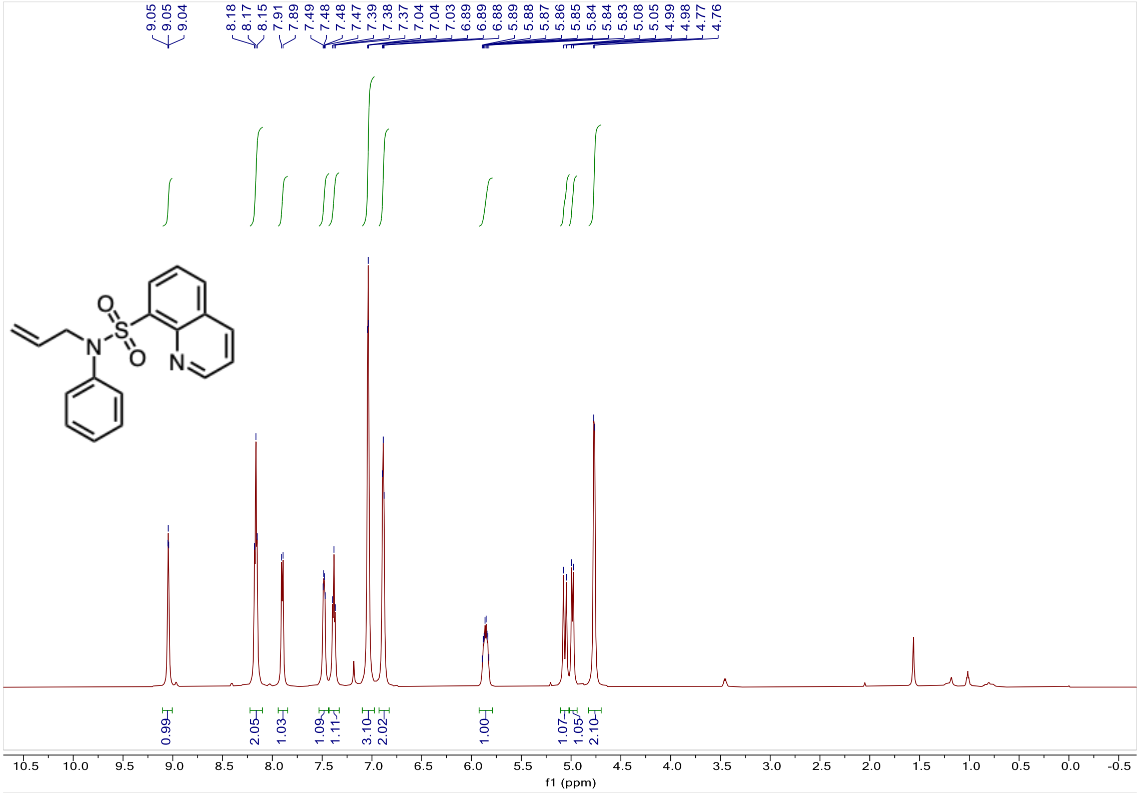

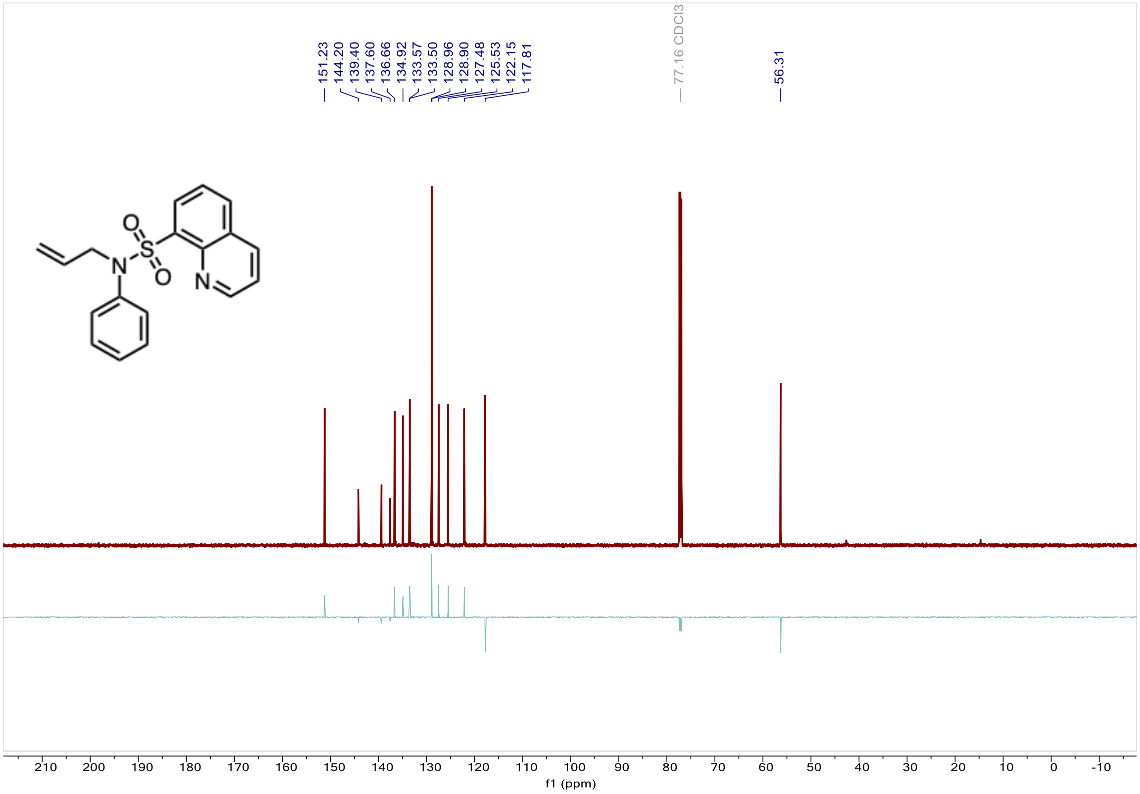

N-allyl-2,4,6-trimethyl-N-(p-tolyl)benzenesulfonamide (1t)

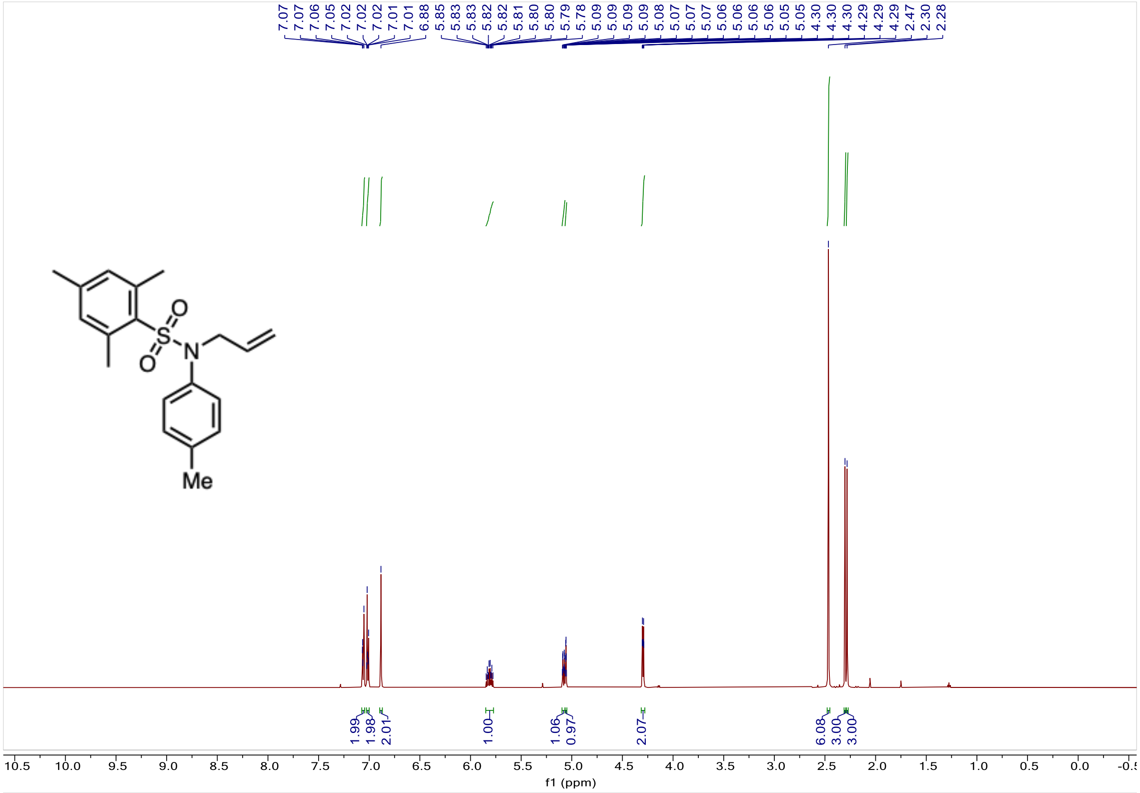

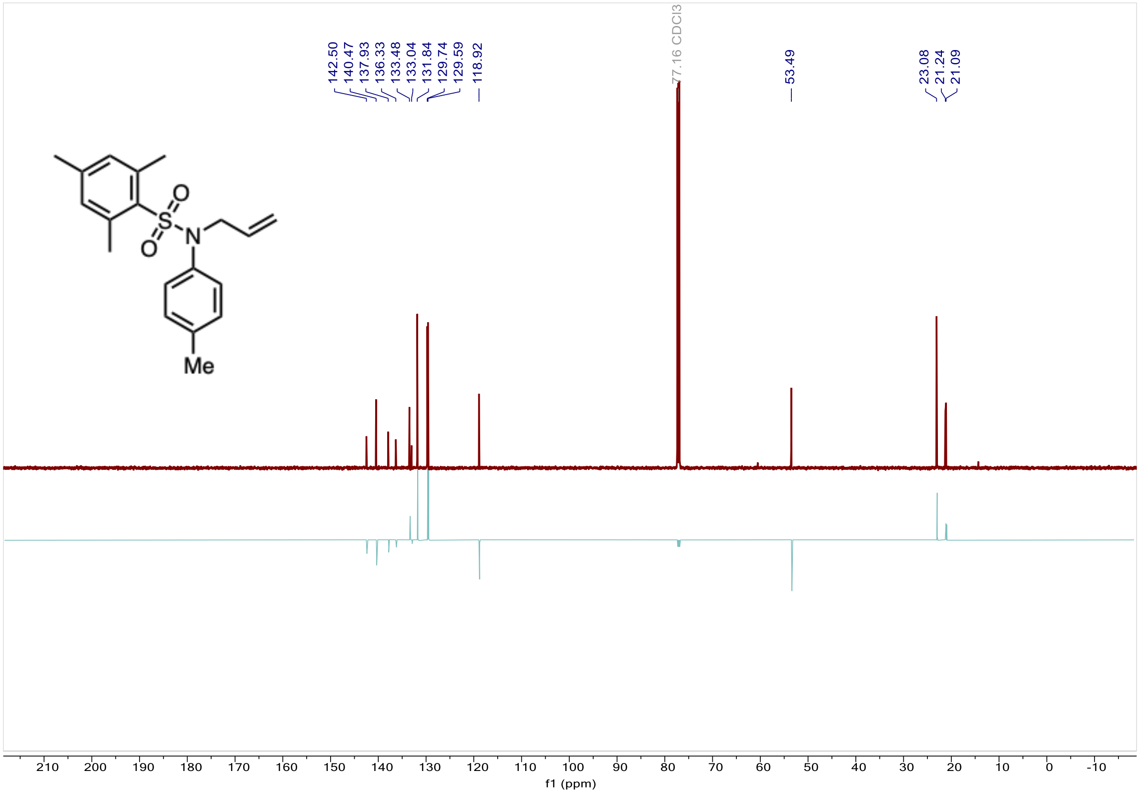

N-allyl-*N*-(4-methoxyphenyl)-2,4,6-trimethylbenzenesulfonamide (1u)

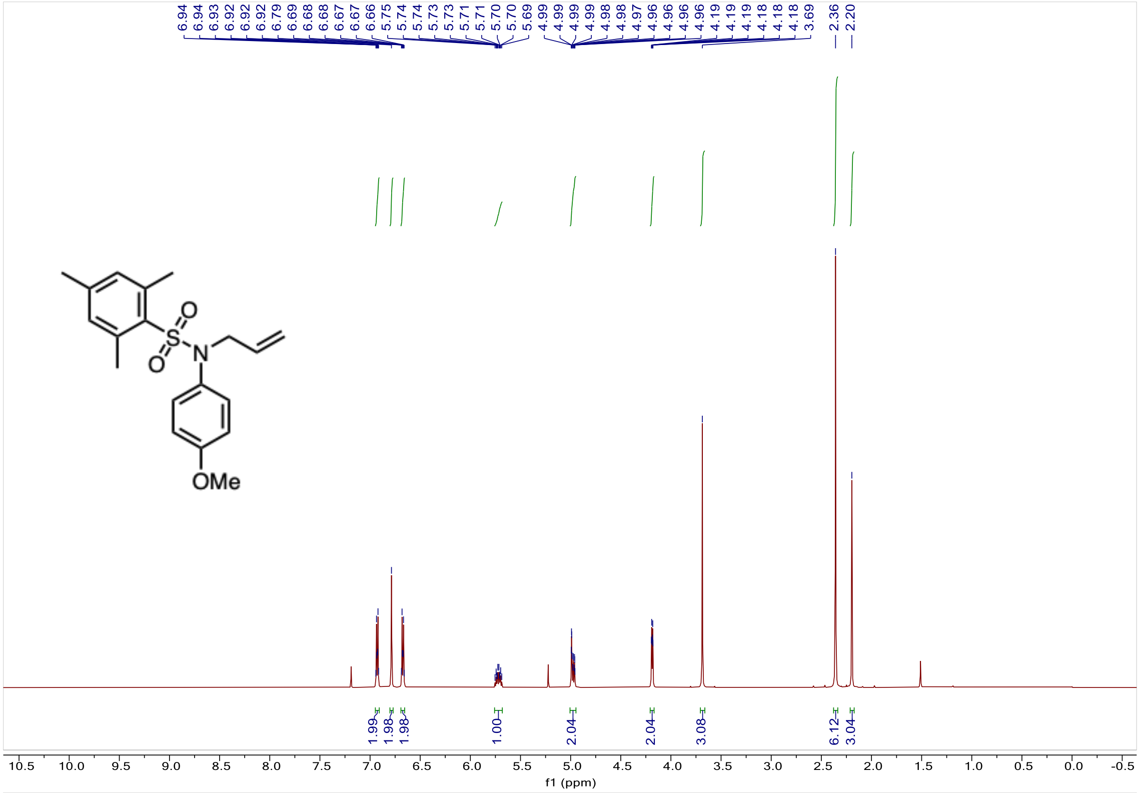

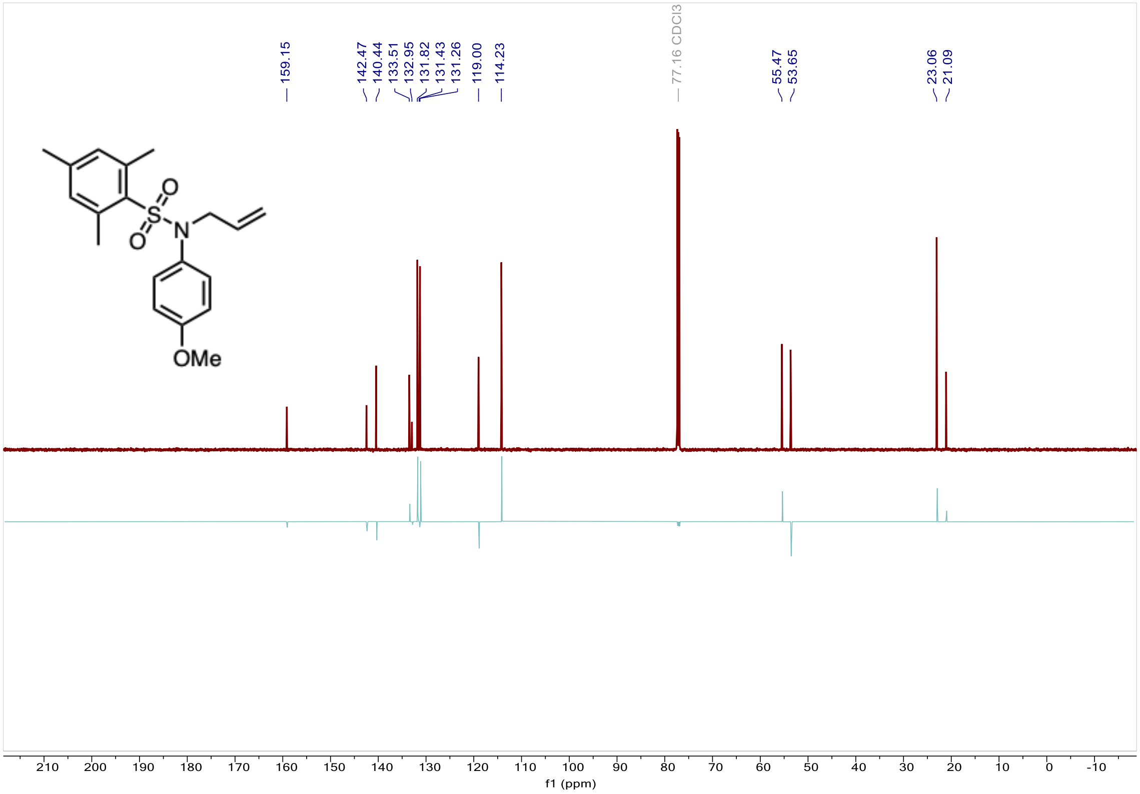

N-allyl-*N*-(4-chlorophenyl)-2,4,6-trimethylbenzenesulfonamide (1v)

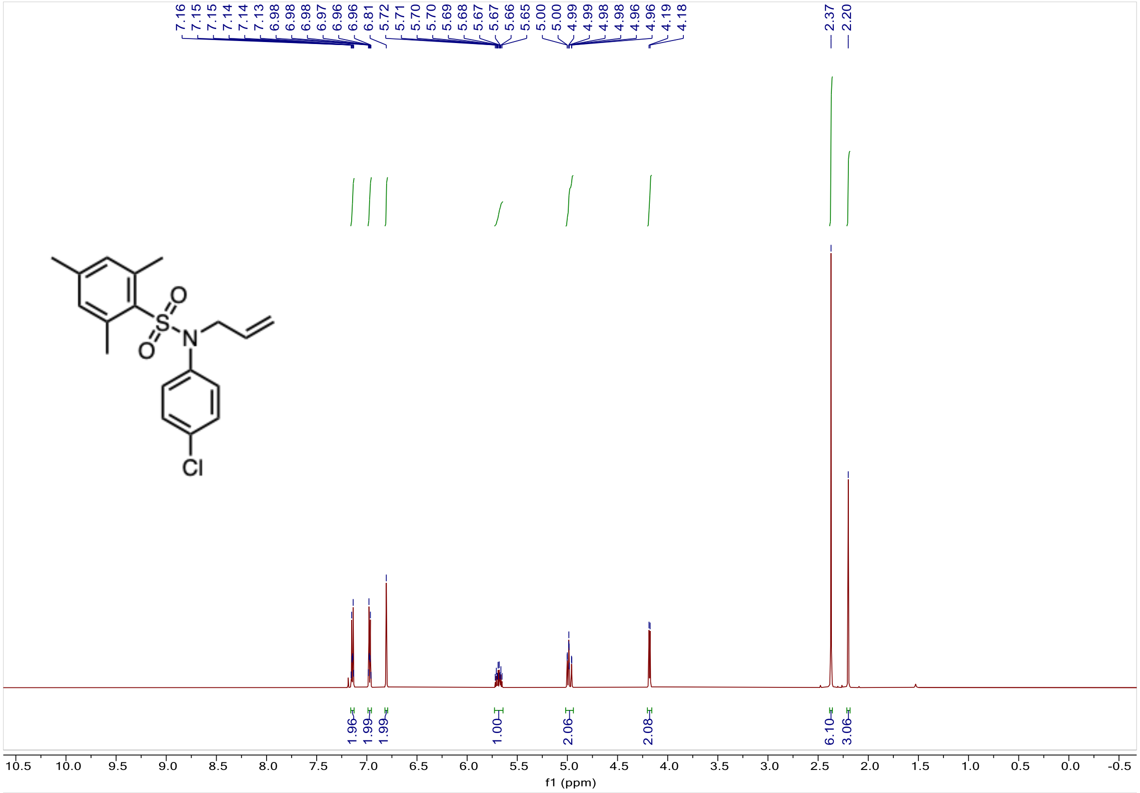

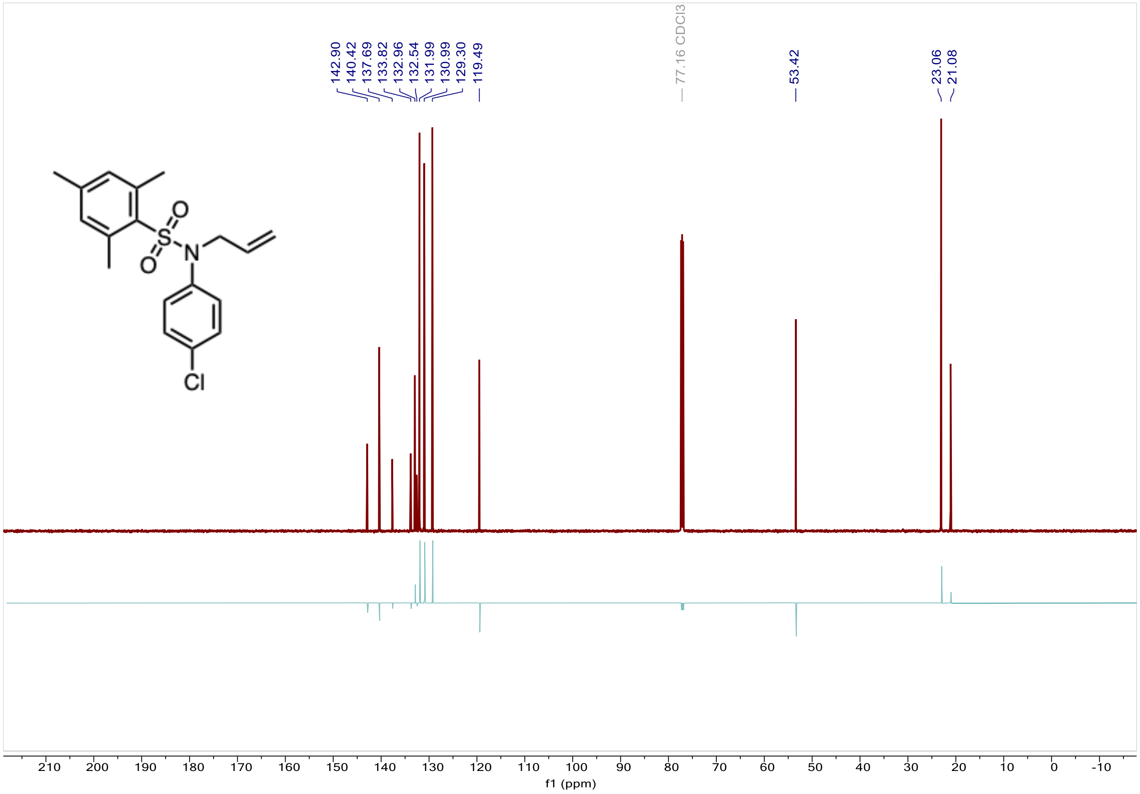

_N_-allyl-_N_-(4-bromophenyl)-2,4,6-trimethylbenzenesulfonamide (1w)

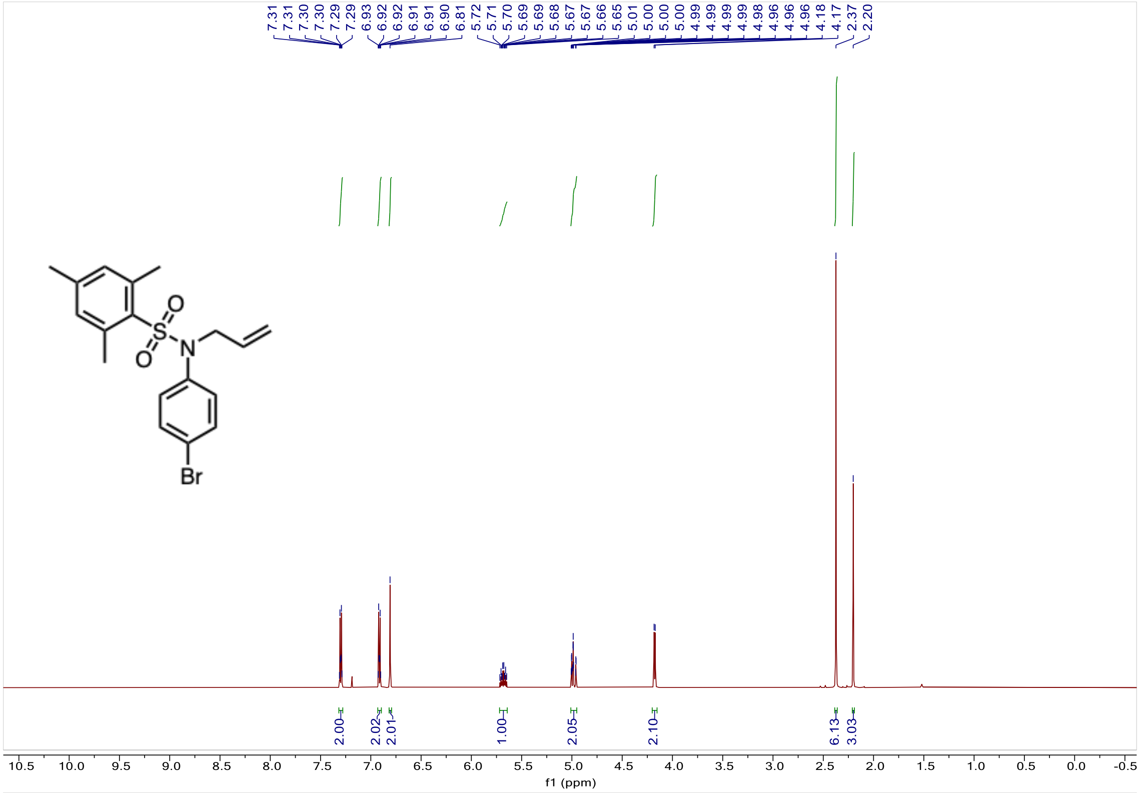

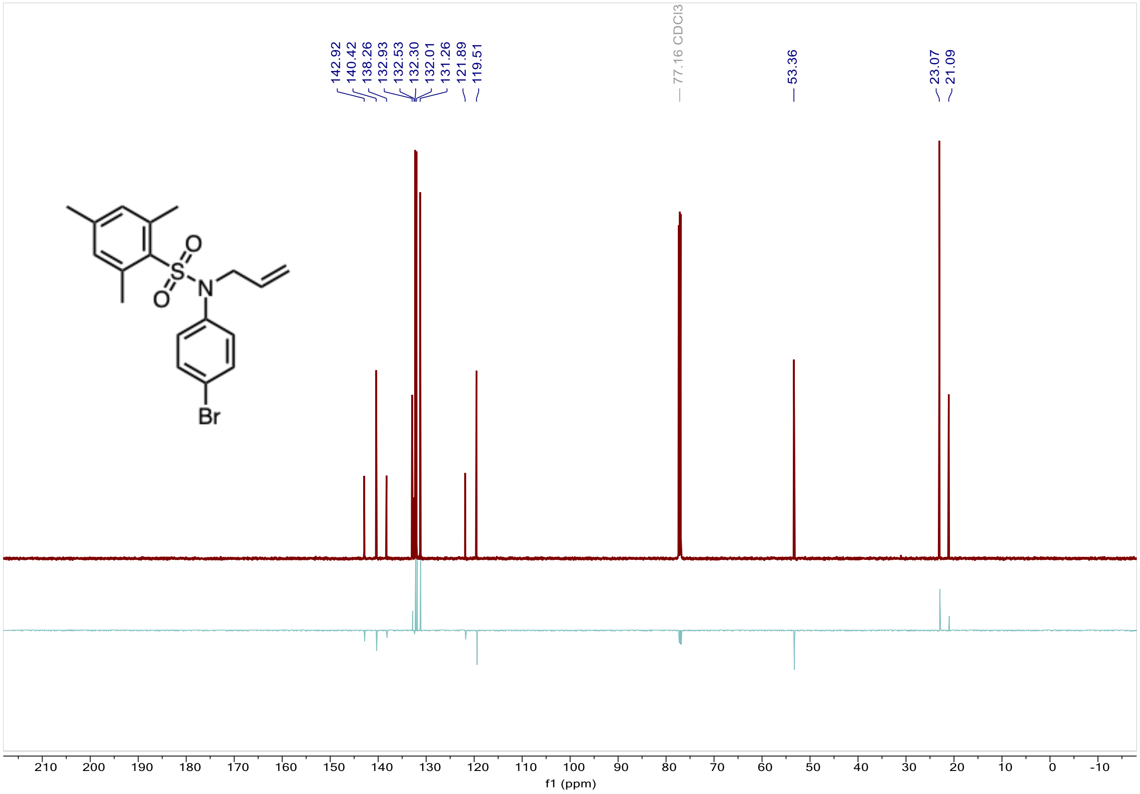

N-allyl-N-(4-fluorophenyl)-2,4,6-trimethylbenzenesulfonamide (1x)

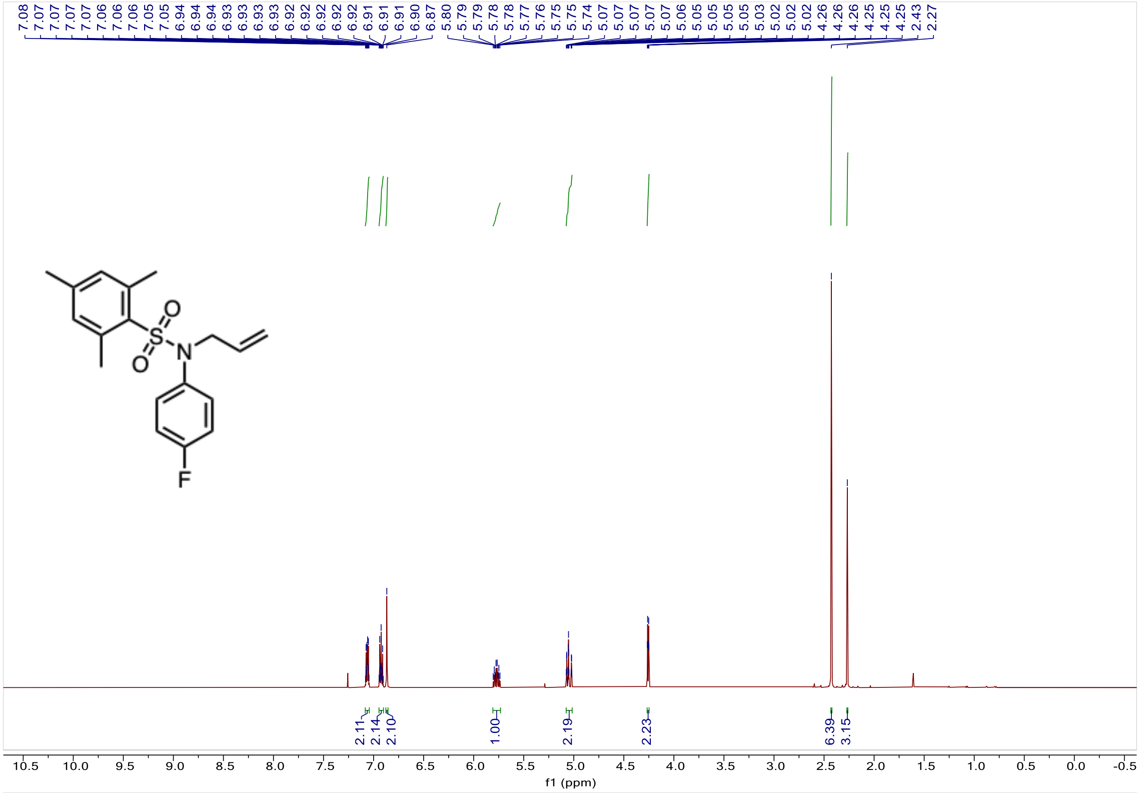

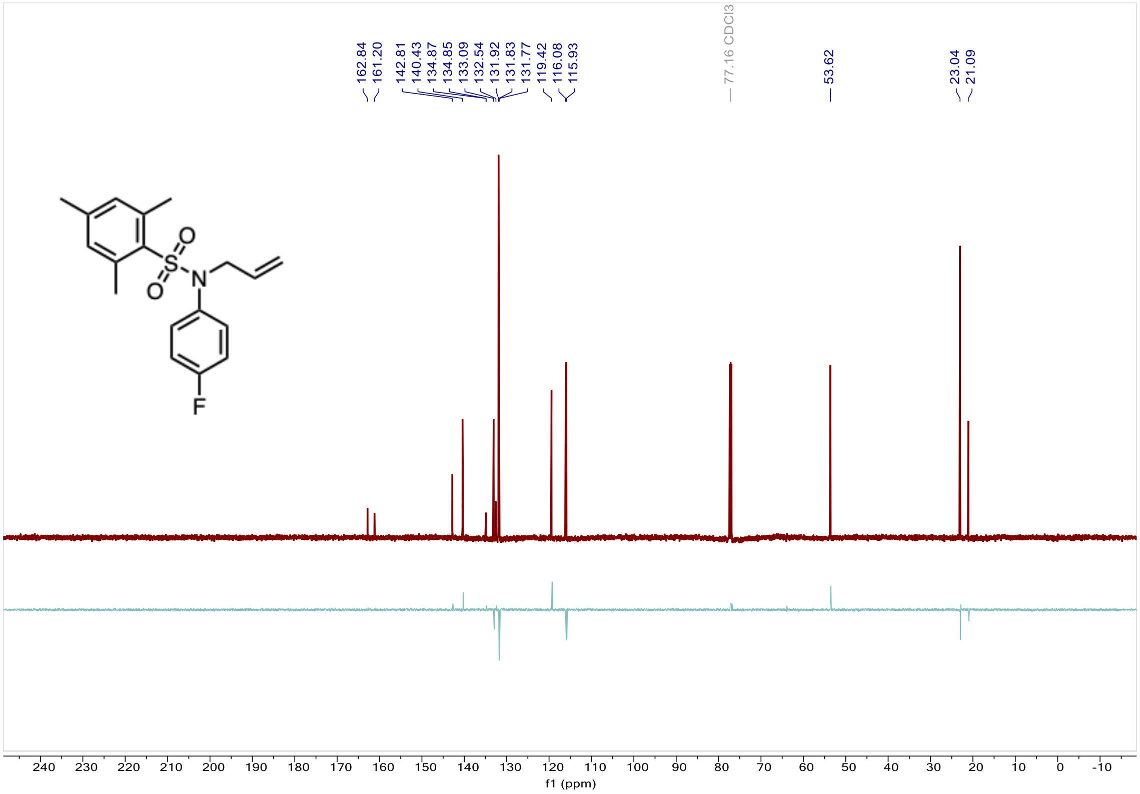

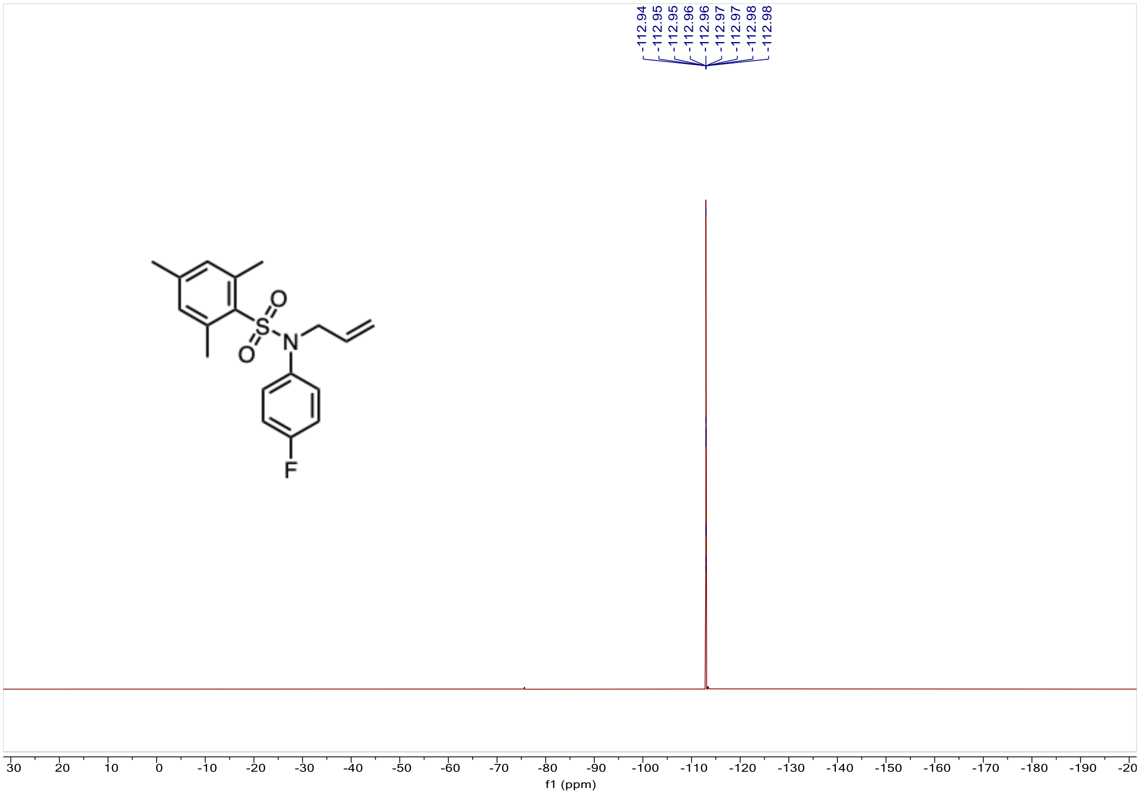

N-allyl-2,4,6-trimethyl-*N*-(4-(trifluoromethyl)phenyl)benzenesulfonamide (1y)

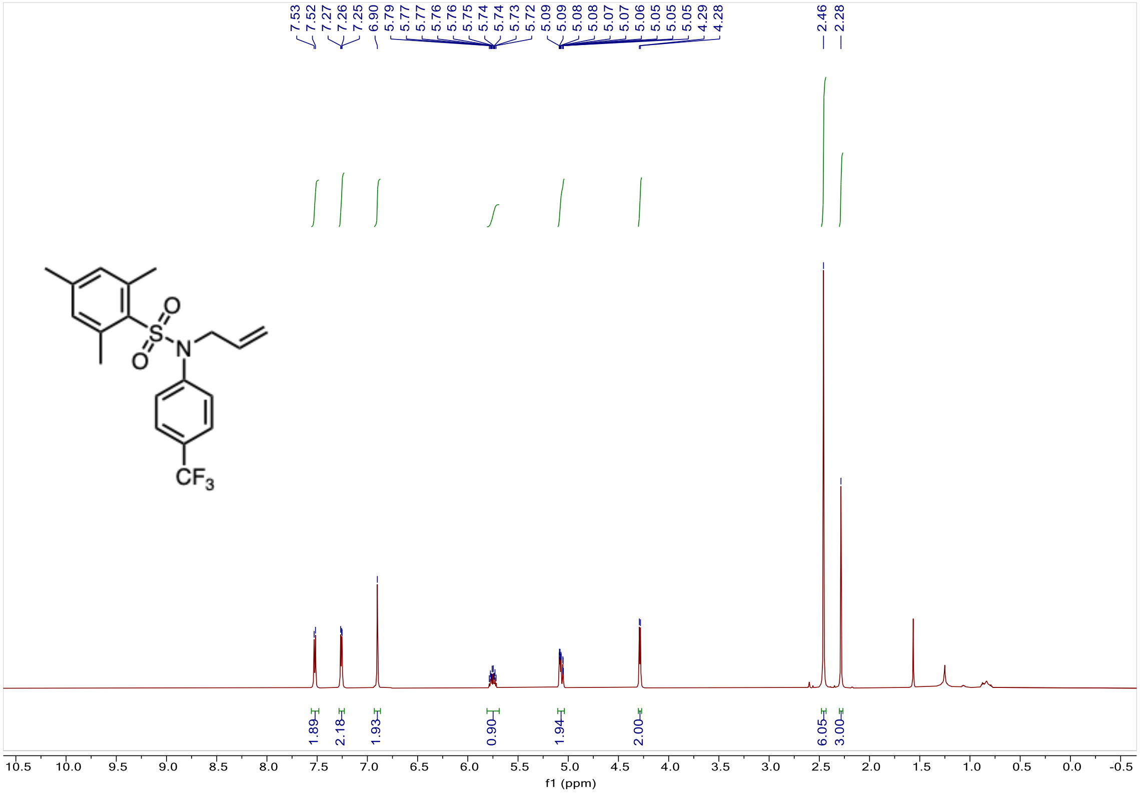

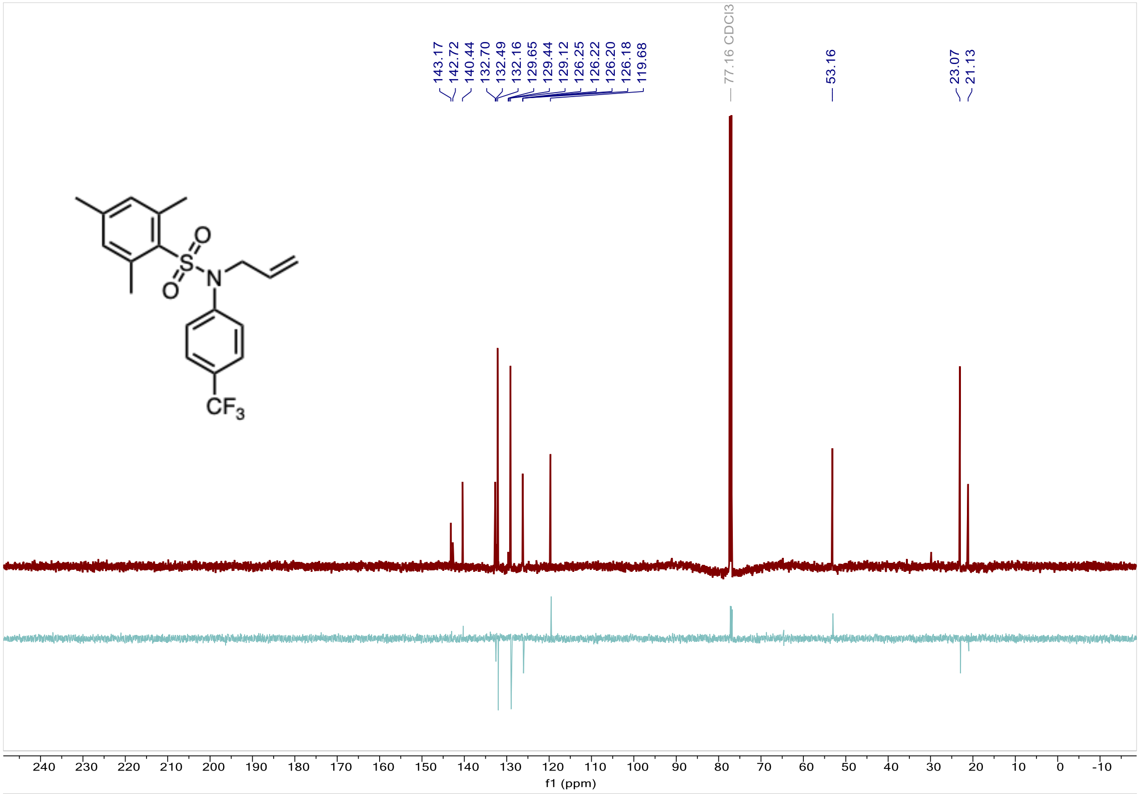
143.17
142.72
140.44
132.70
132.49
132.16
129.65
129.44
129.12
126.25
126.22
126.20
126.18
119.68
77.16 CDCl3
53.16
23.07
21.13
CF3
240 230 220 210 200 190 180 170 160 150 140 130 120 110 100 90 80 70 60 50 40 30 20 10 0 -10
f1 (ppm)

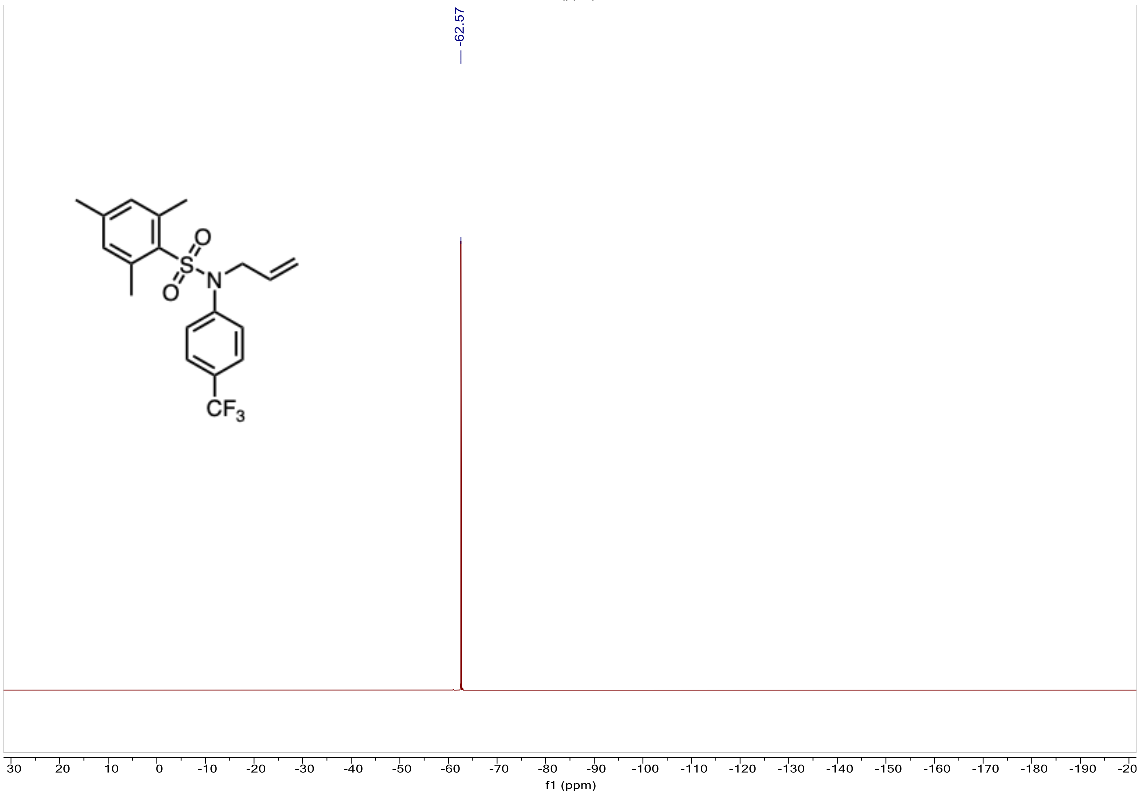
-62.57
CF3
30 20 10 0 -10 -20 -30 -40 -50 -60 -70 -80 -90 -100 -110 -120 -130 -140 -150 -160 -170 -180 -190 -20
f1 (ppm)

N-allyl-2,4,6-trimethyl-*N*-(4-phenoxyphenyl)benzenesulfonamide (1z)

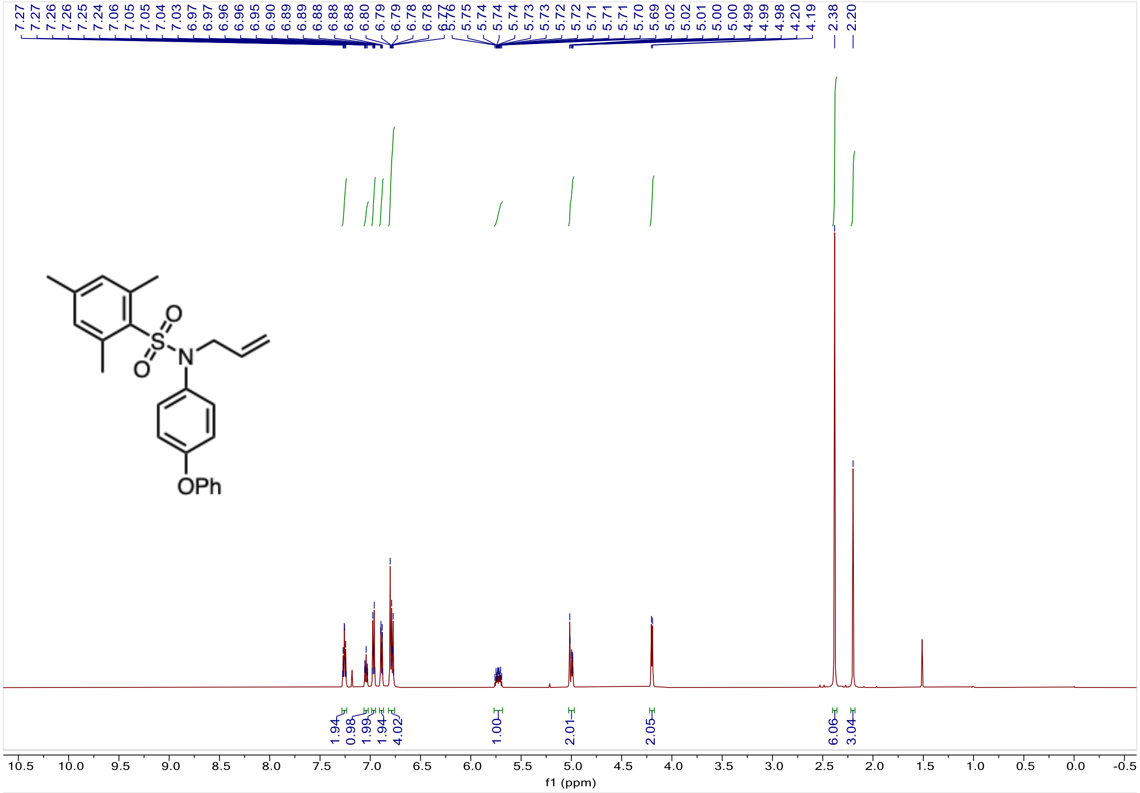

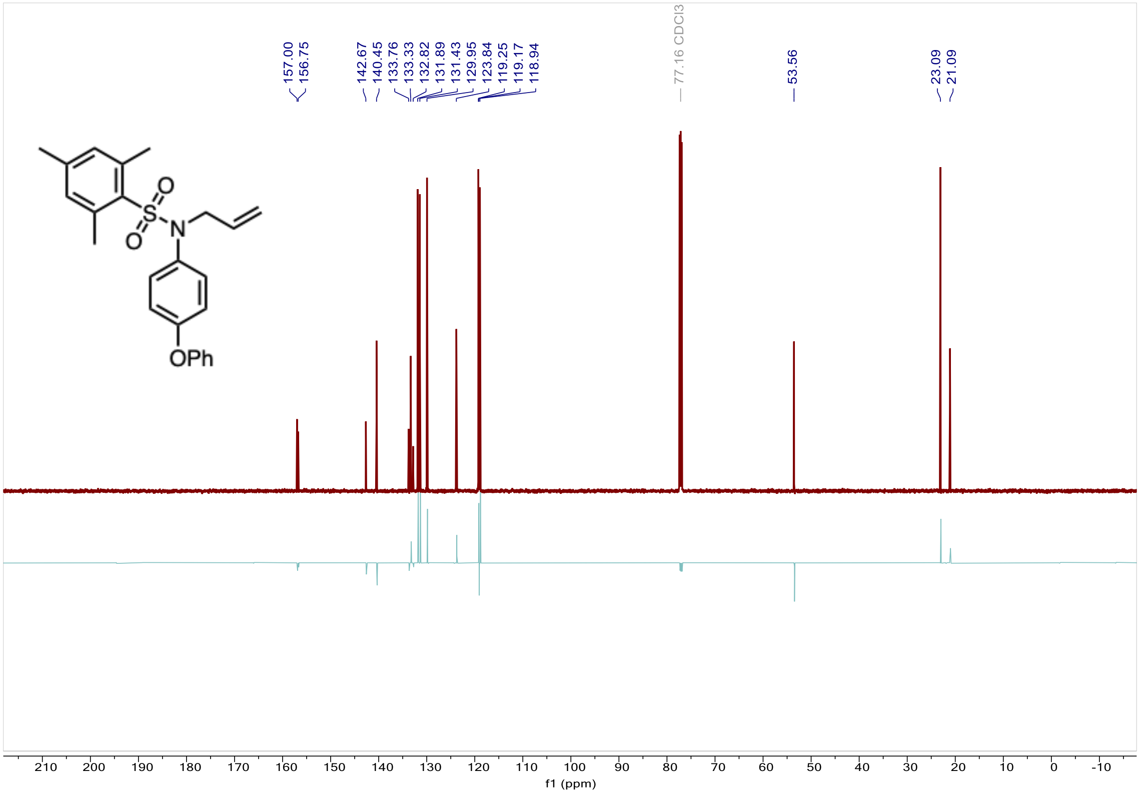

***N*-allyl-2,4,6-trimethyl-*N*-(*m*-tolyl)benzenesulfonamide (1aa)**

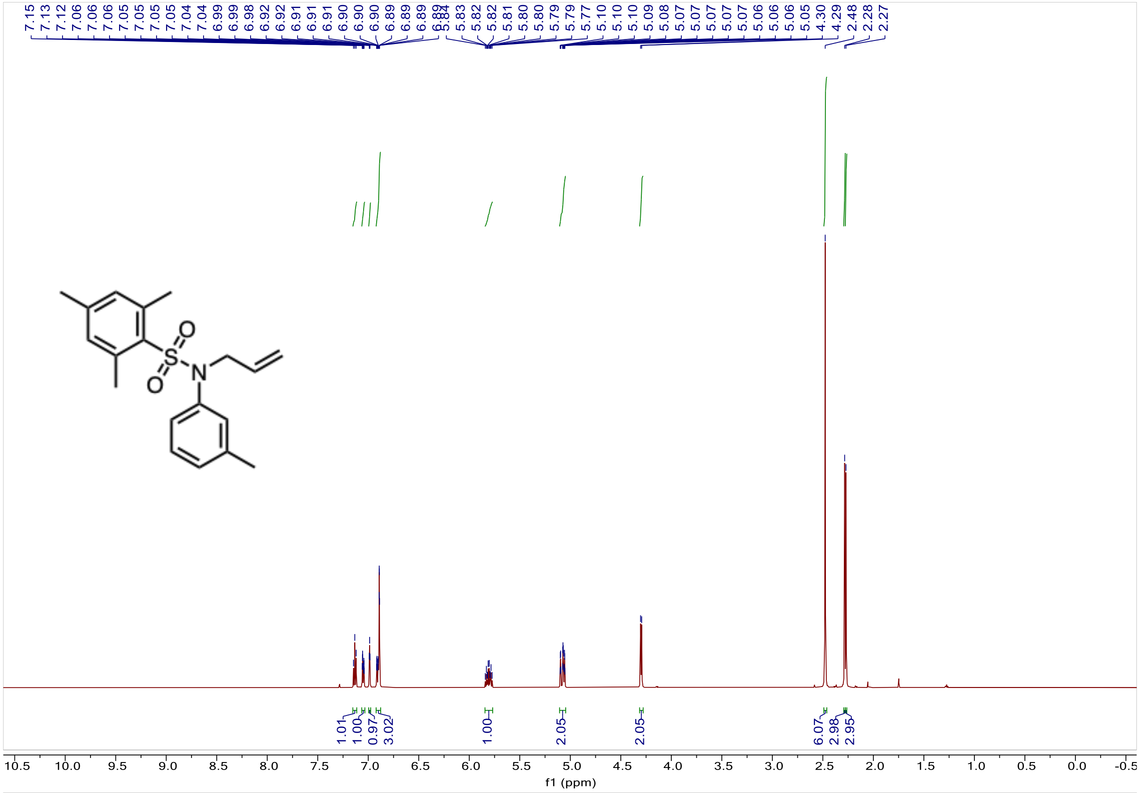

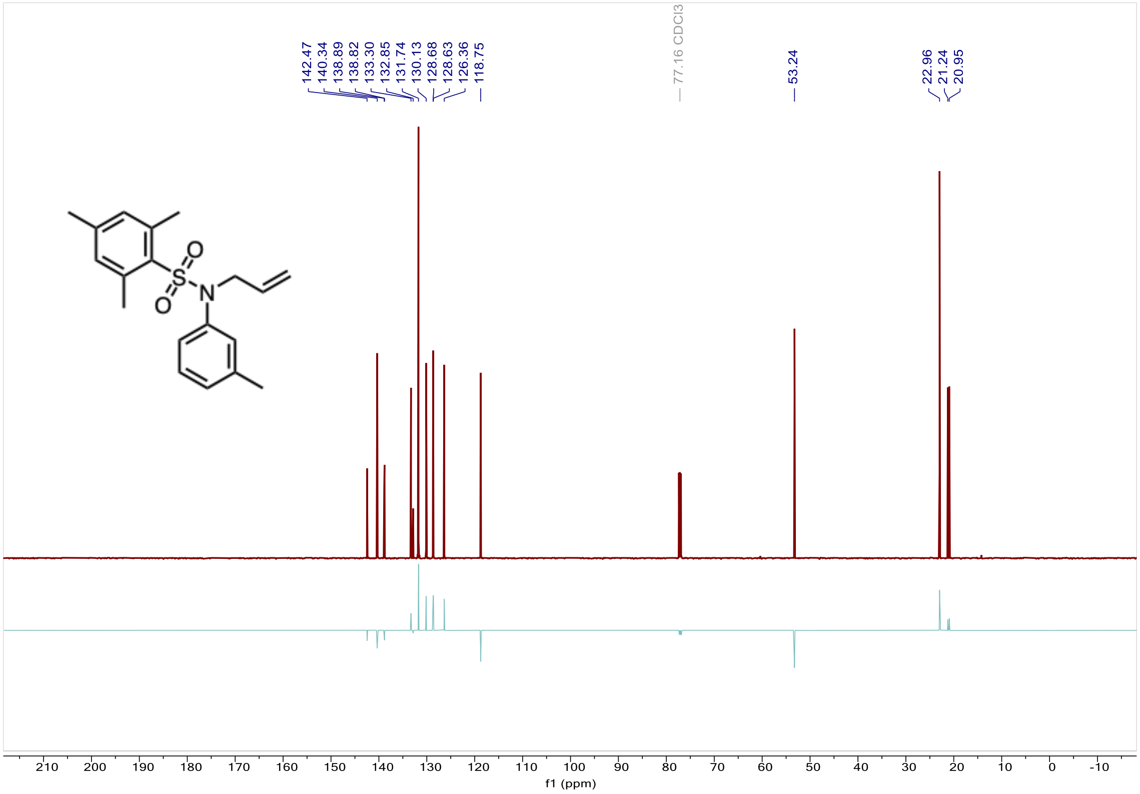

N-allyl-*N*-(3-methoxyphenyl)-2,4,6-trimethylbenzenesulfonamide (1ab)

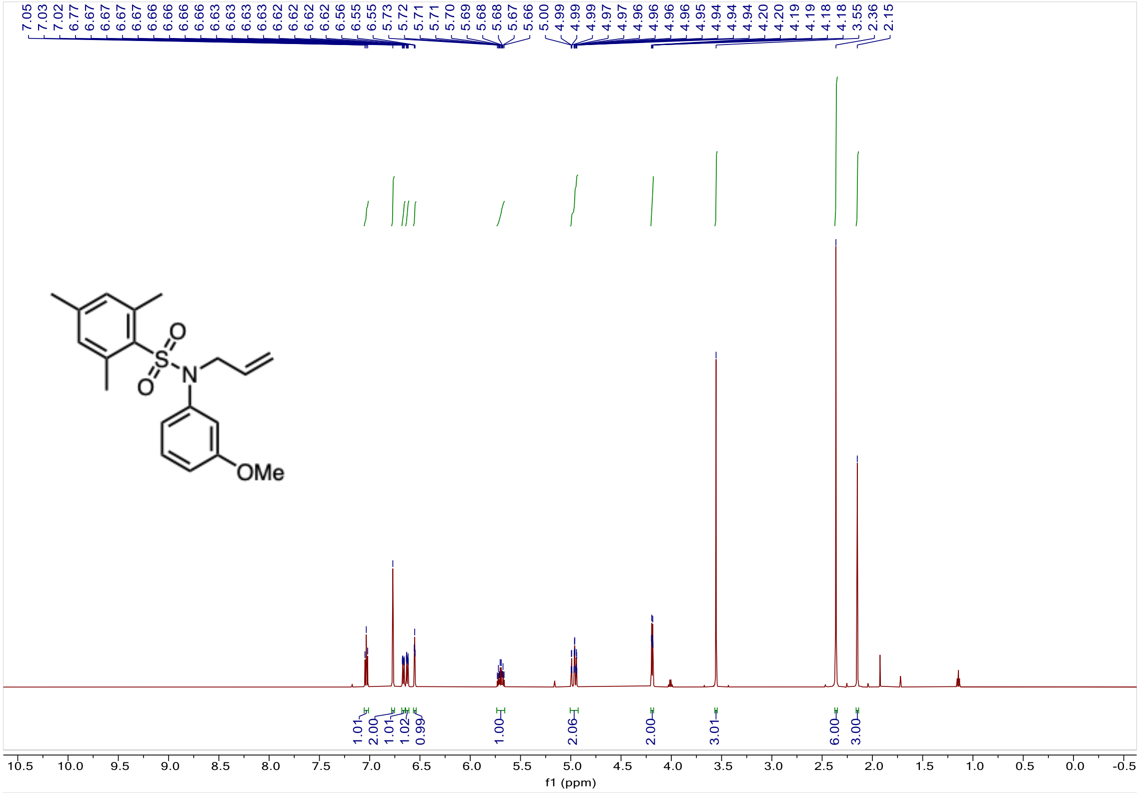

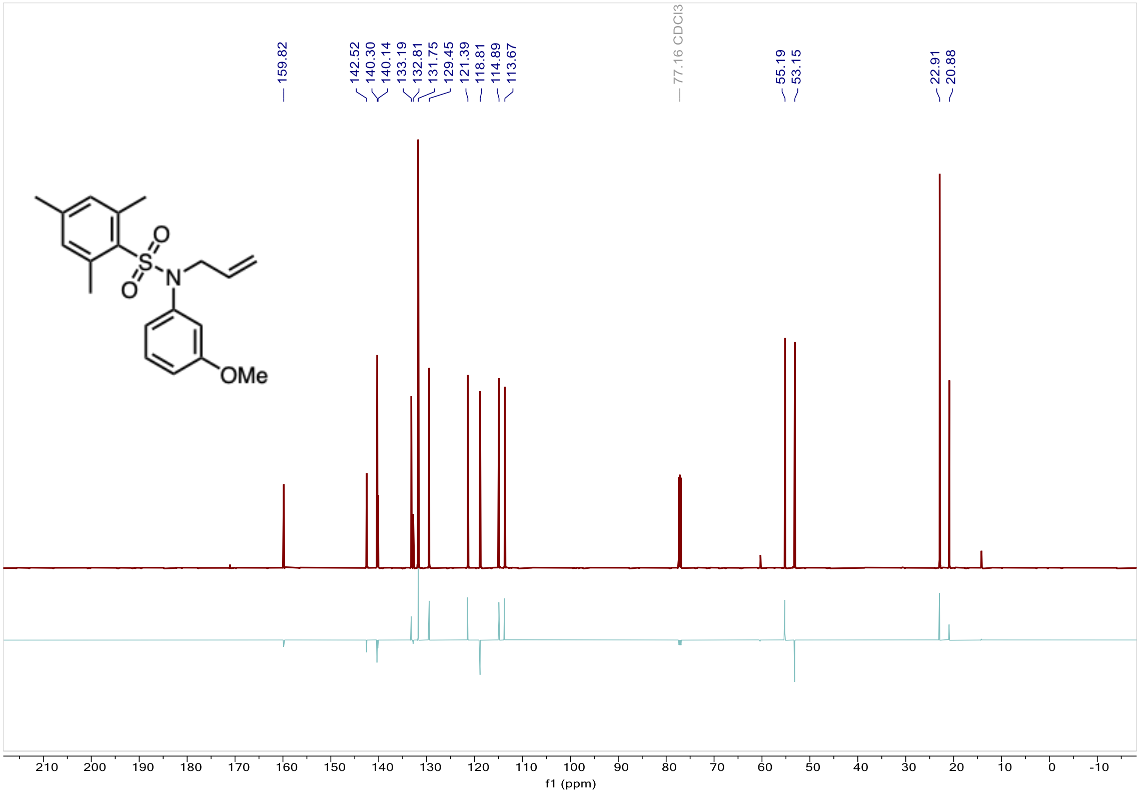

N-allyl-*N*-(3-bromophenyl)-2,4,6-trimethylbenzenesulfonamide (1ac)

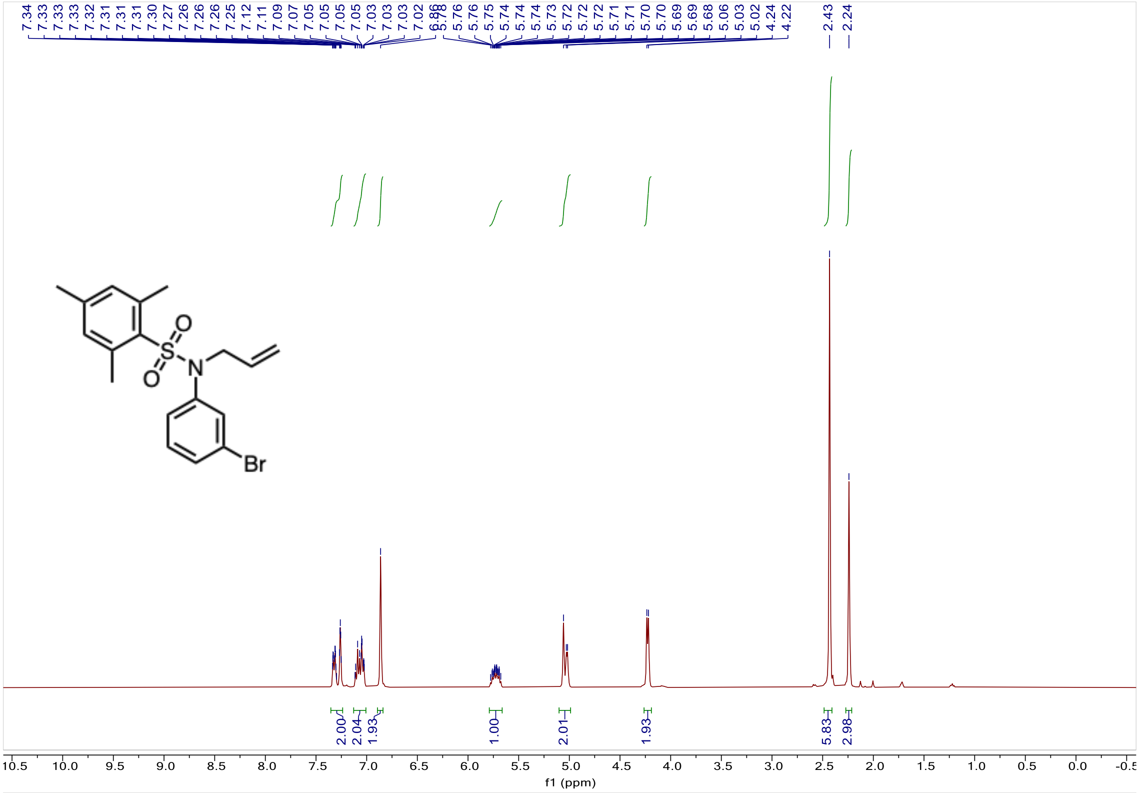

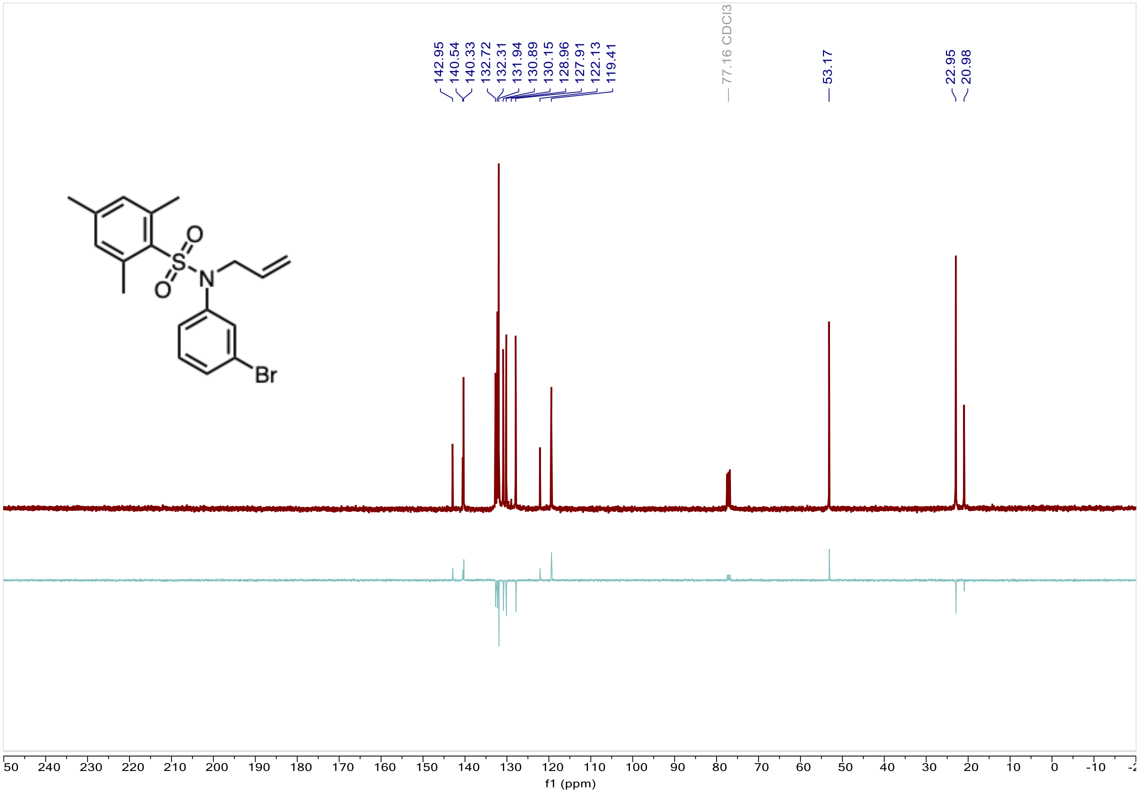

N-allyl-*N*-(3,5-dimethylphenyl)-2,4,6-trimethylbenzenesulfonamide (1ad)

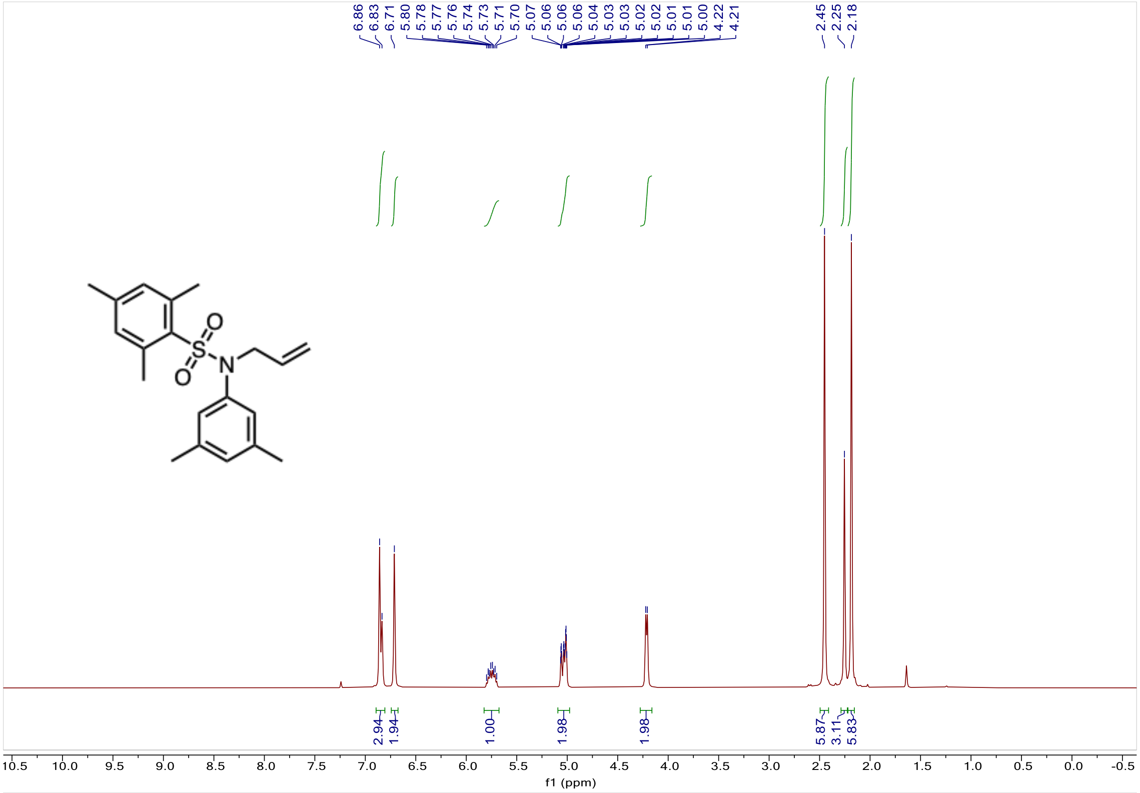

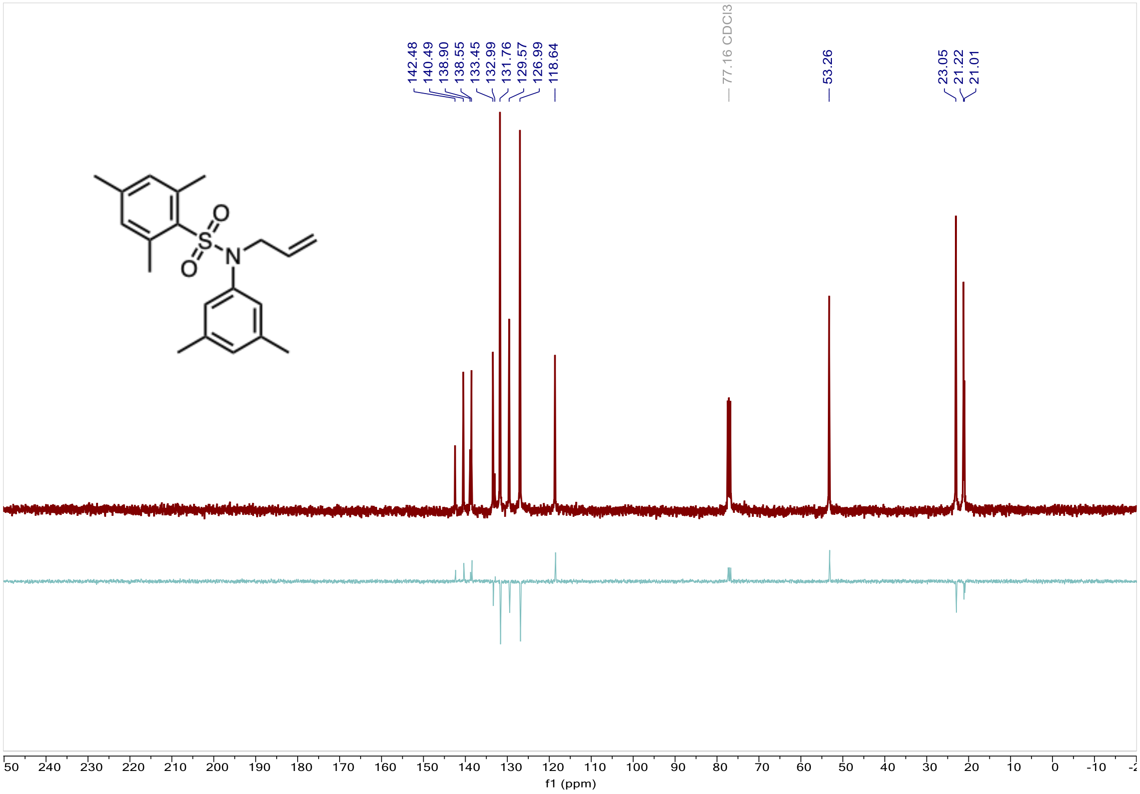

N-allyl-2,4,6-trimethyl-*N*-(*o*-tolyl)benzenesulfonamide (1ae)

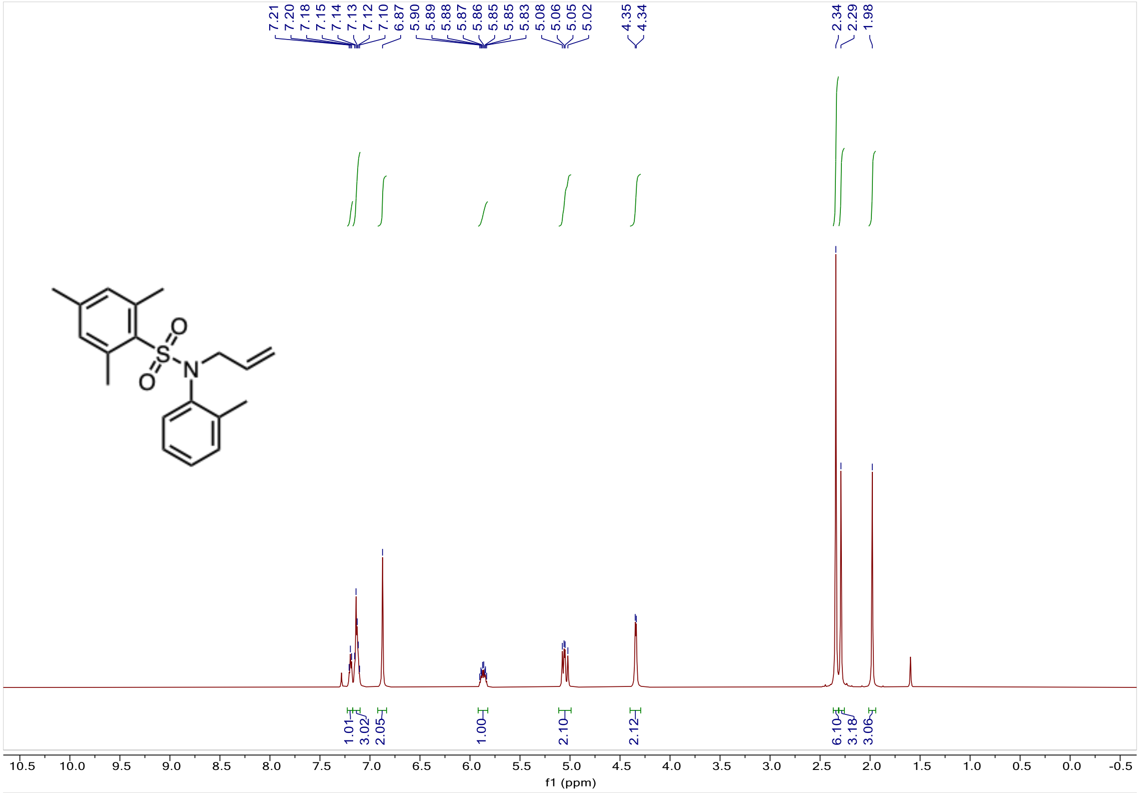

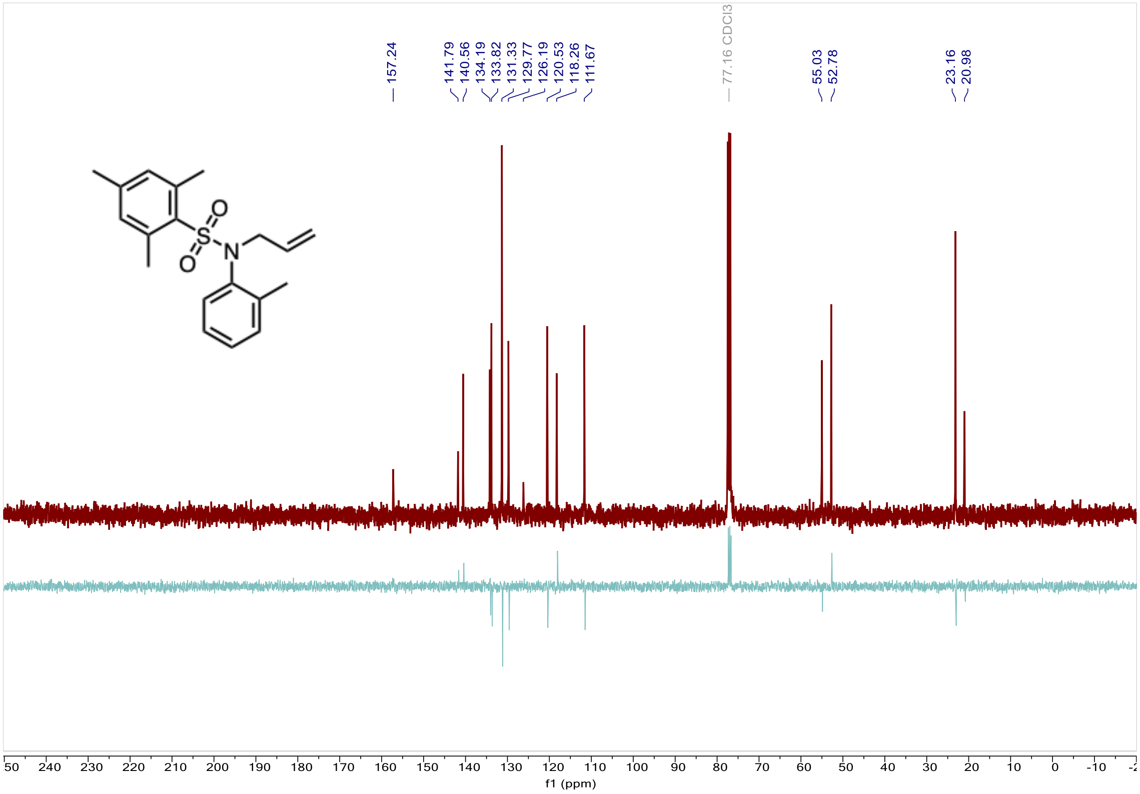

N-allyl-*N*-(2-methoxyphenyl)-2,4,6-trimethylbenzenesulfonamide (1af)

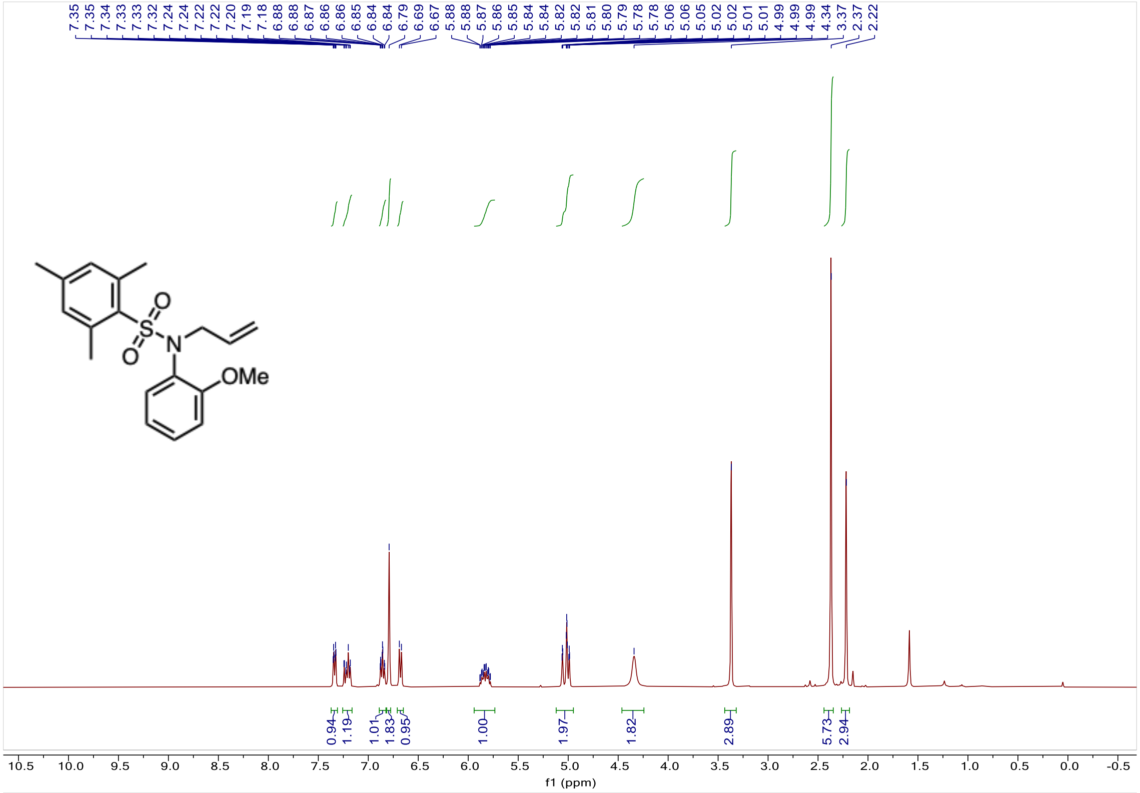

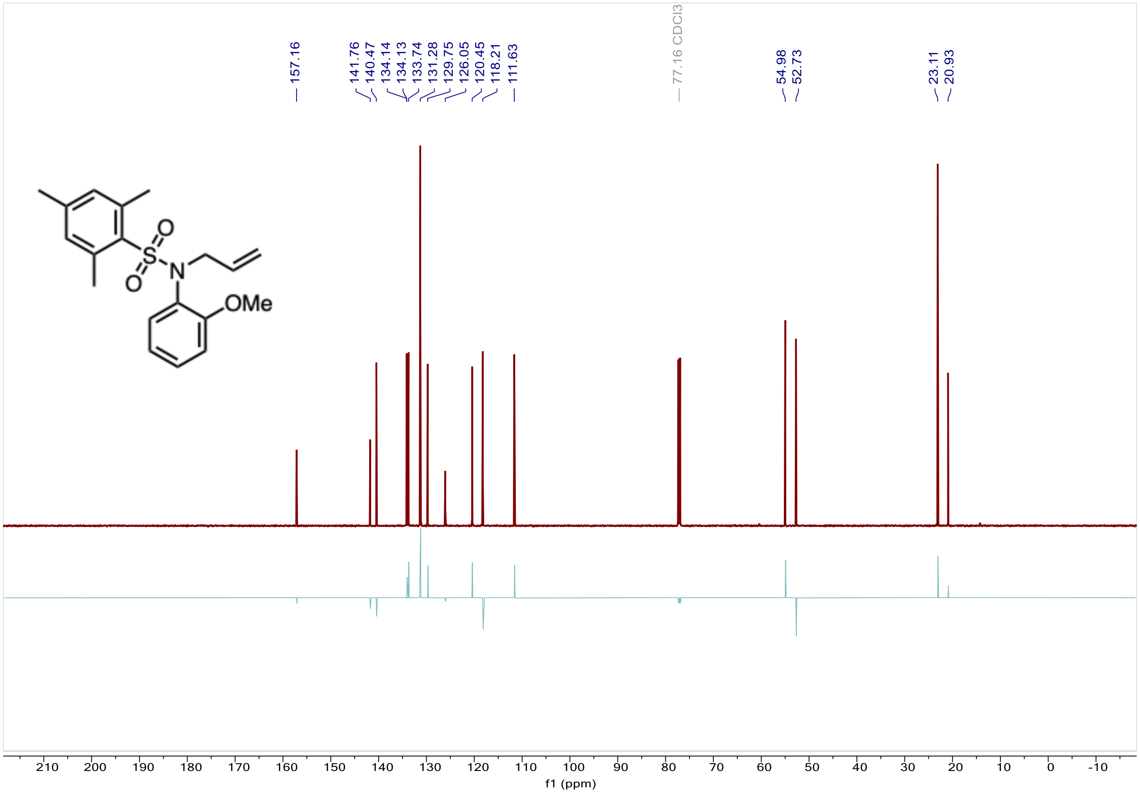

N-allyl-*N*-(2-bromophenyl)-2,4,6-trimethylbenzenesulfonamide (1ag)

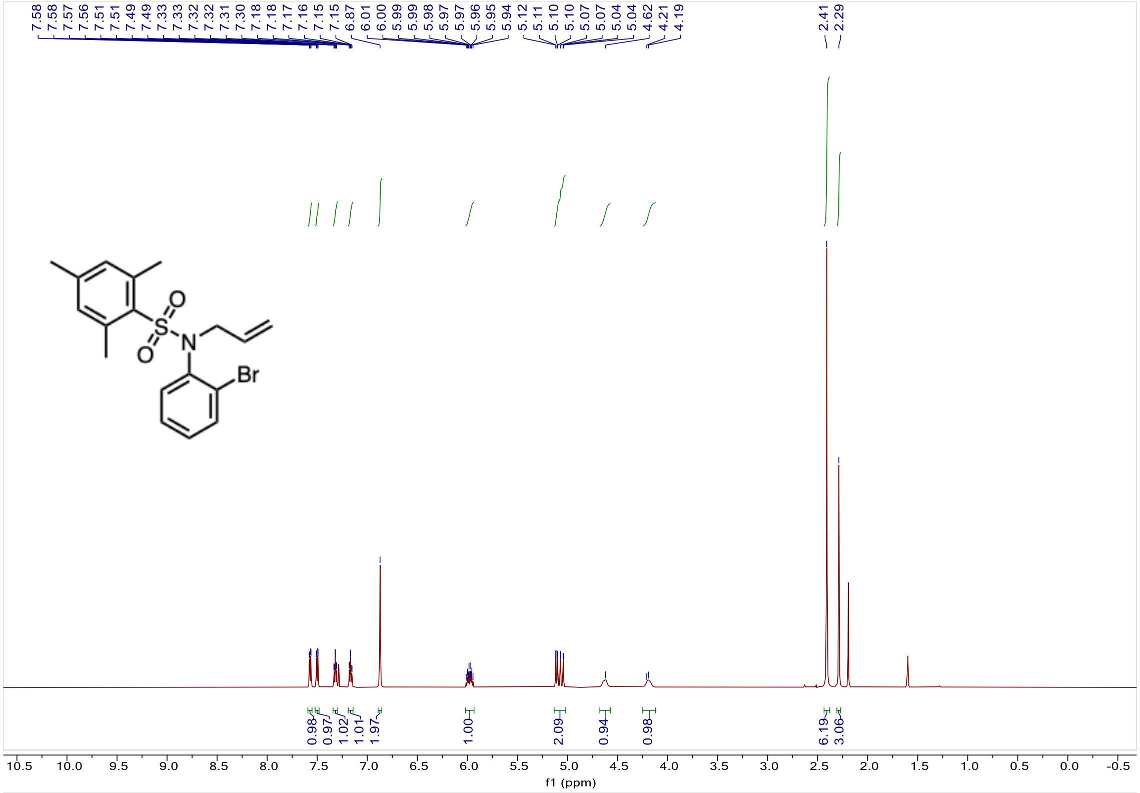

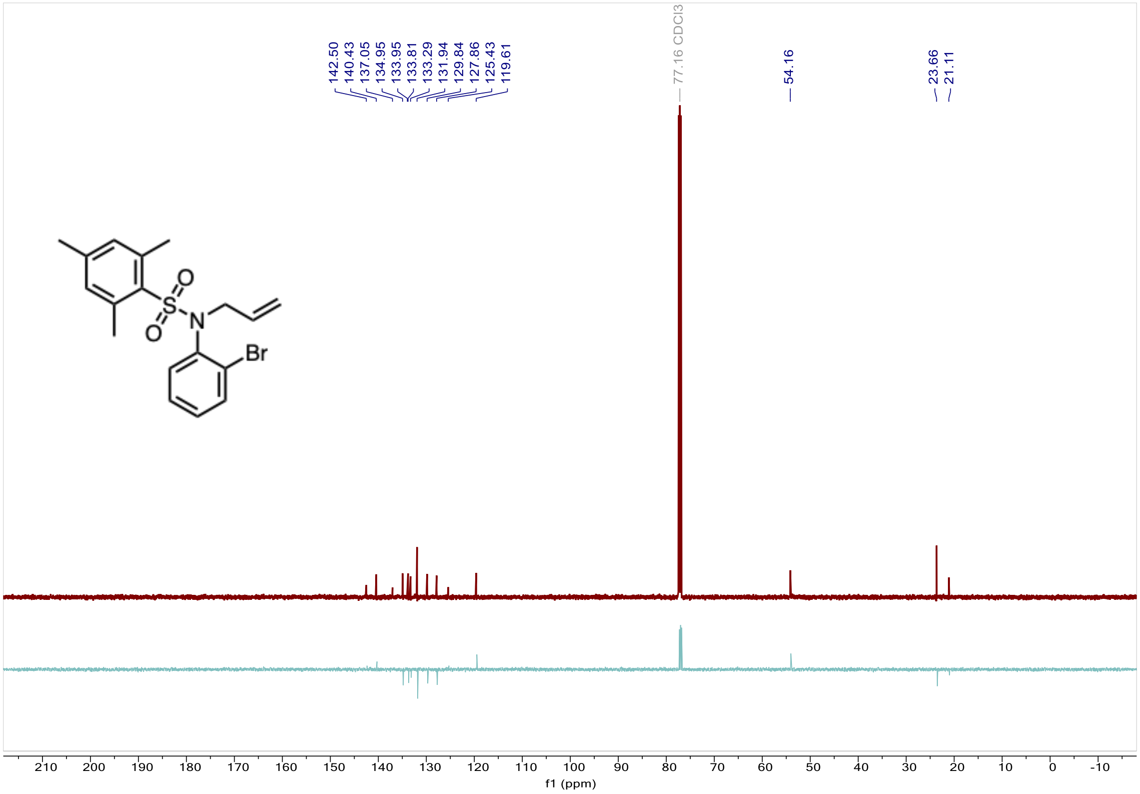

N-allyl-*N*-(benzo[*d*][1,3]dioxol-5-yl)-2,4,6-trimethylbenzenesulfonamide (1ah)

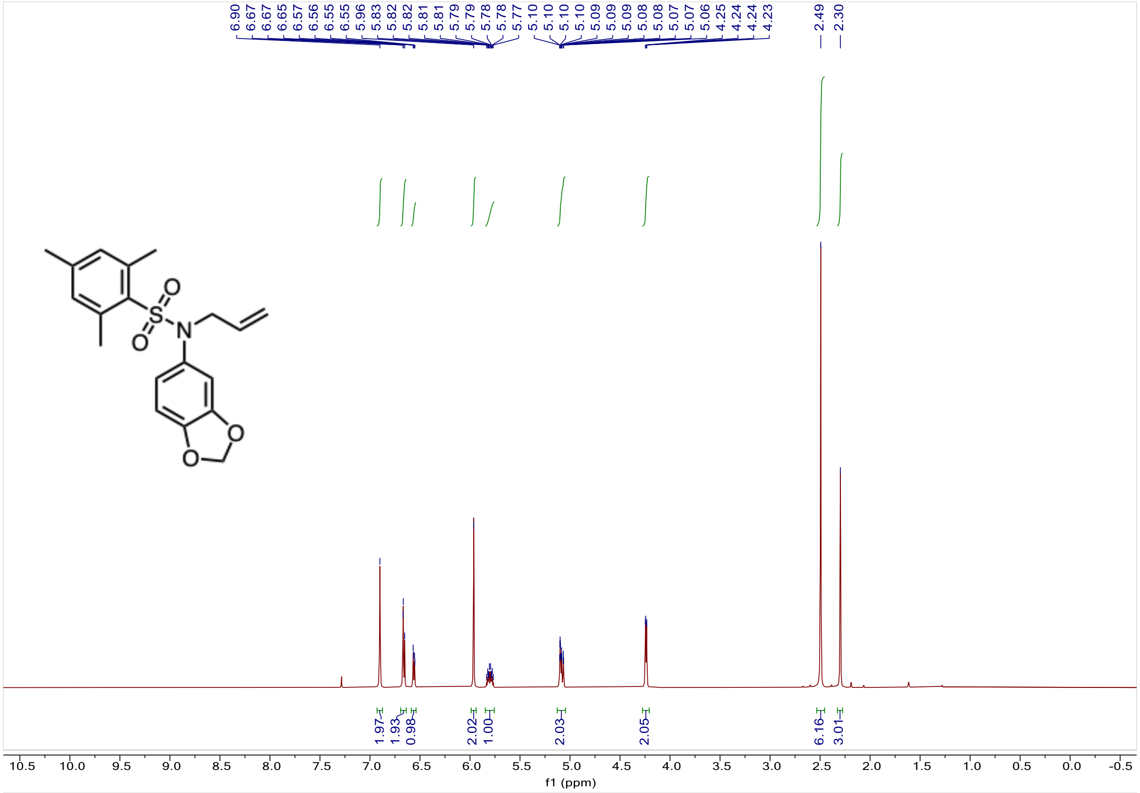

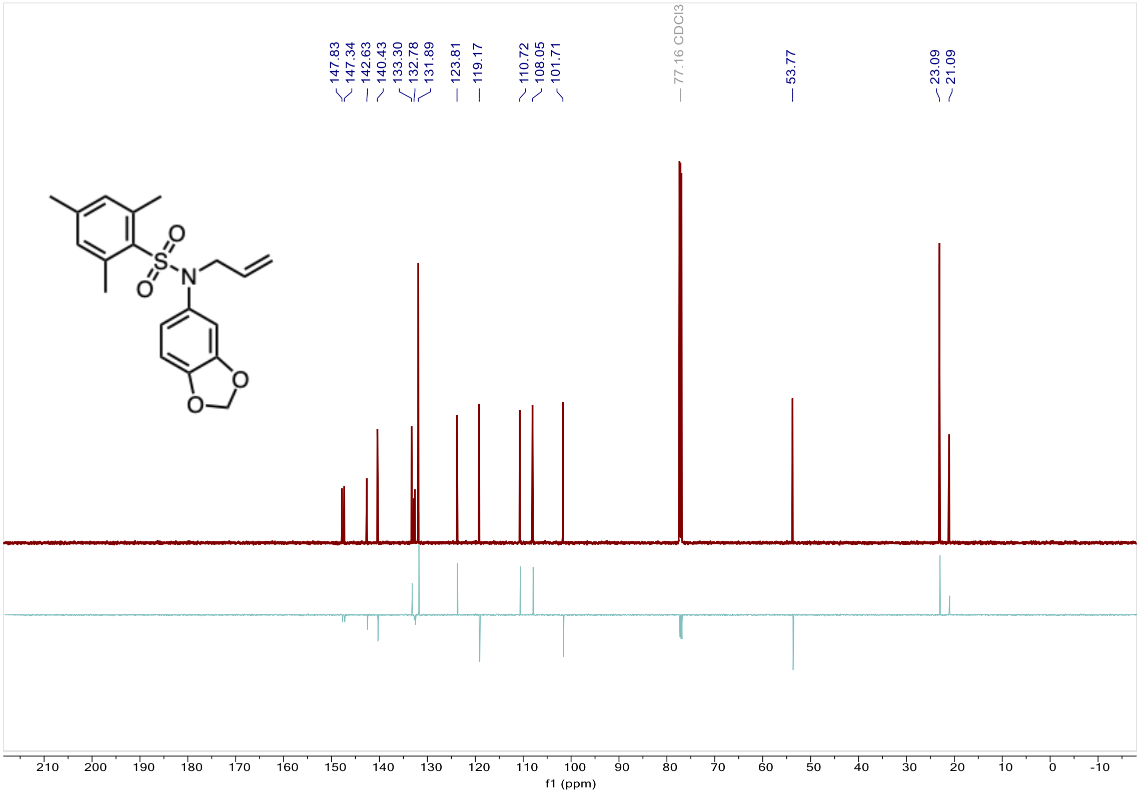

Butyl 4-((*N*-allyl-2,4,6-trimethylphenyl)sulfonamido)benzoate (1ai)

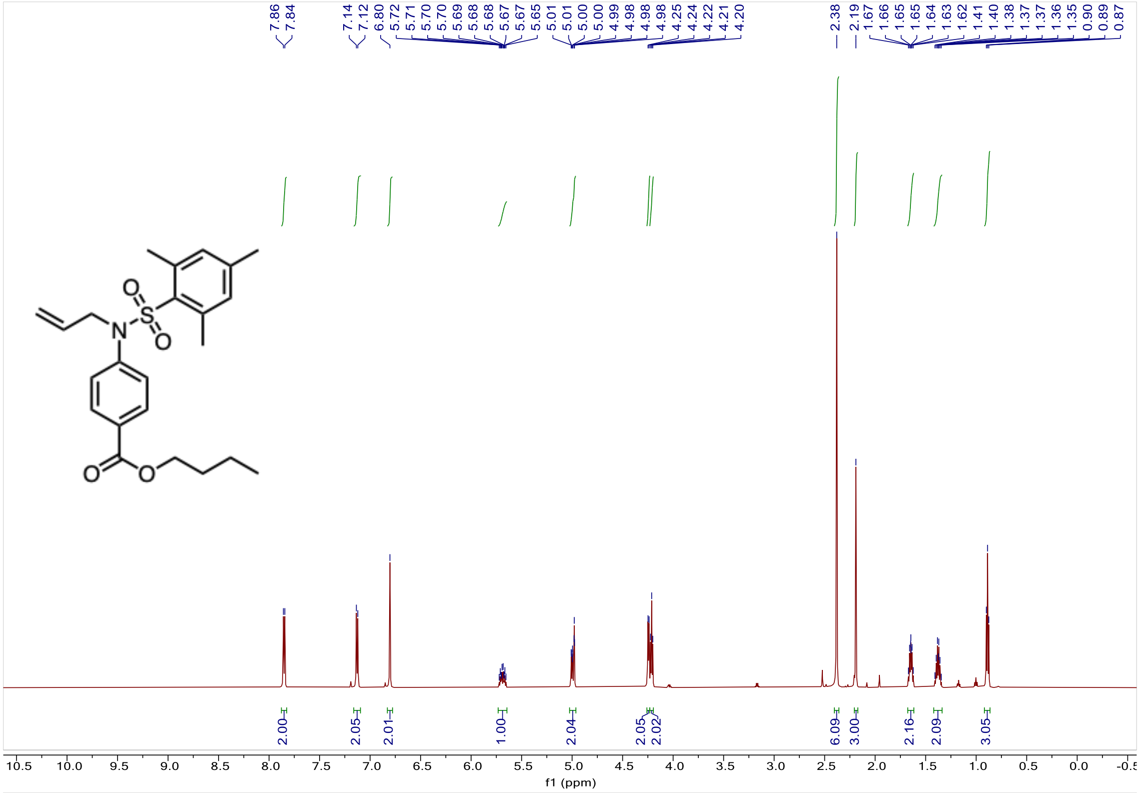

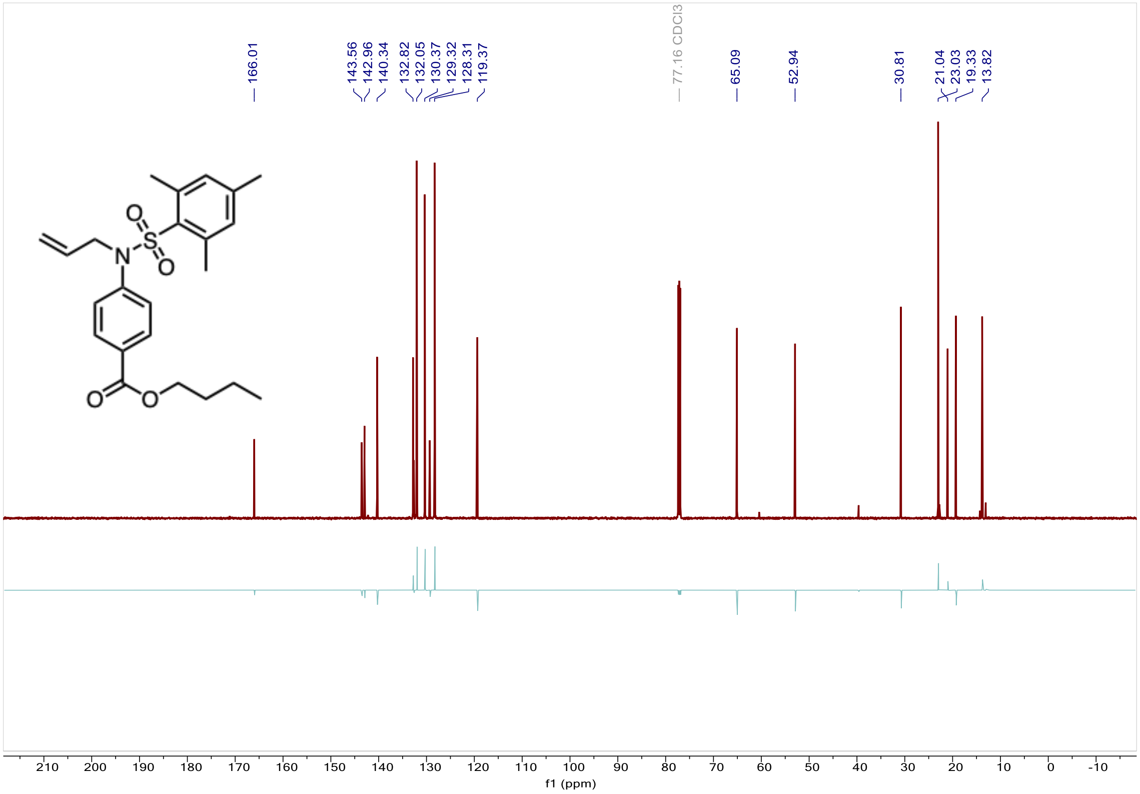

(1*R*,2*S*,5*R*)-2-isopropyl-5-methylcyclohexyl4-((*N*-allyl-2,4,6-trimethylphenyl) sulfonamido) benzoate (1aj)

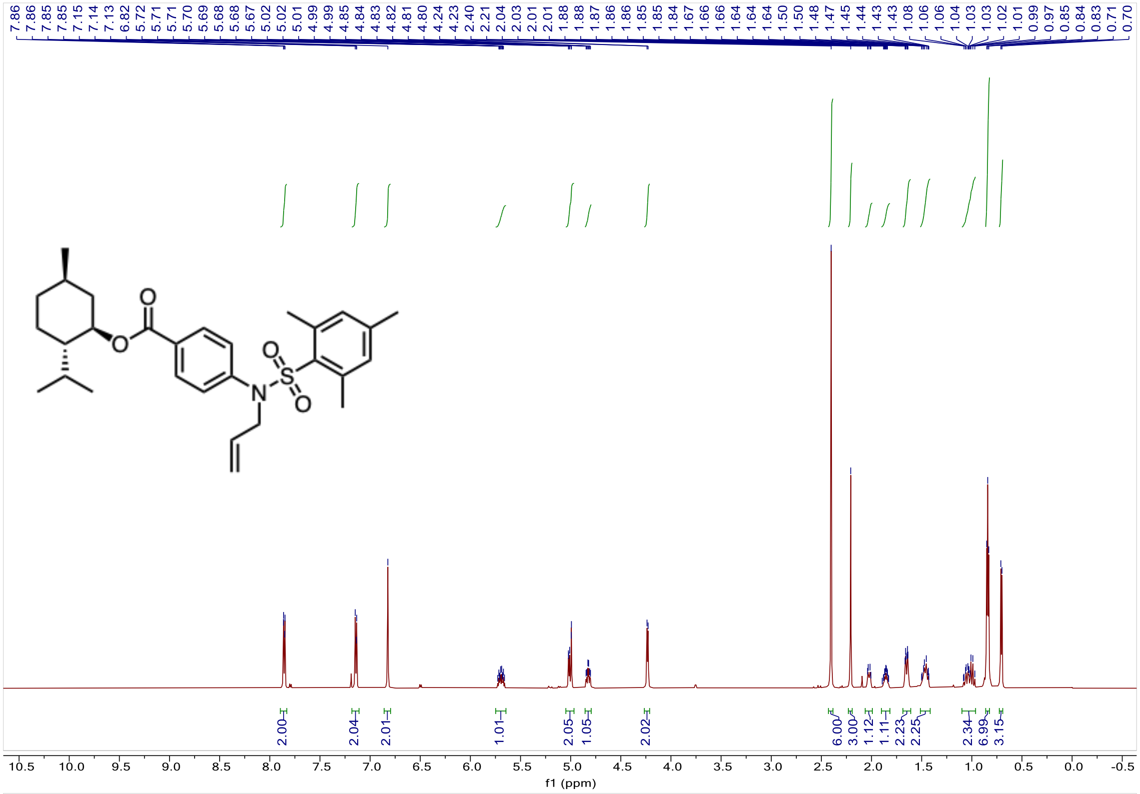

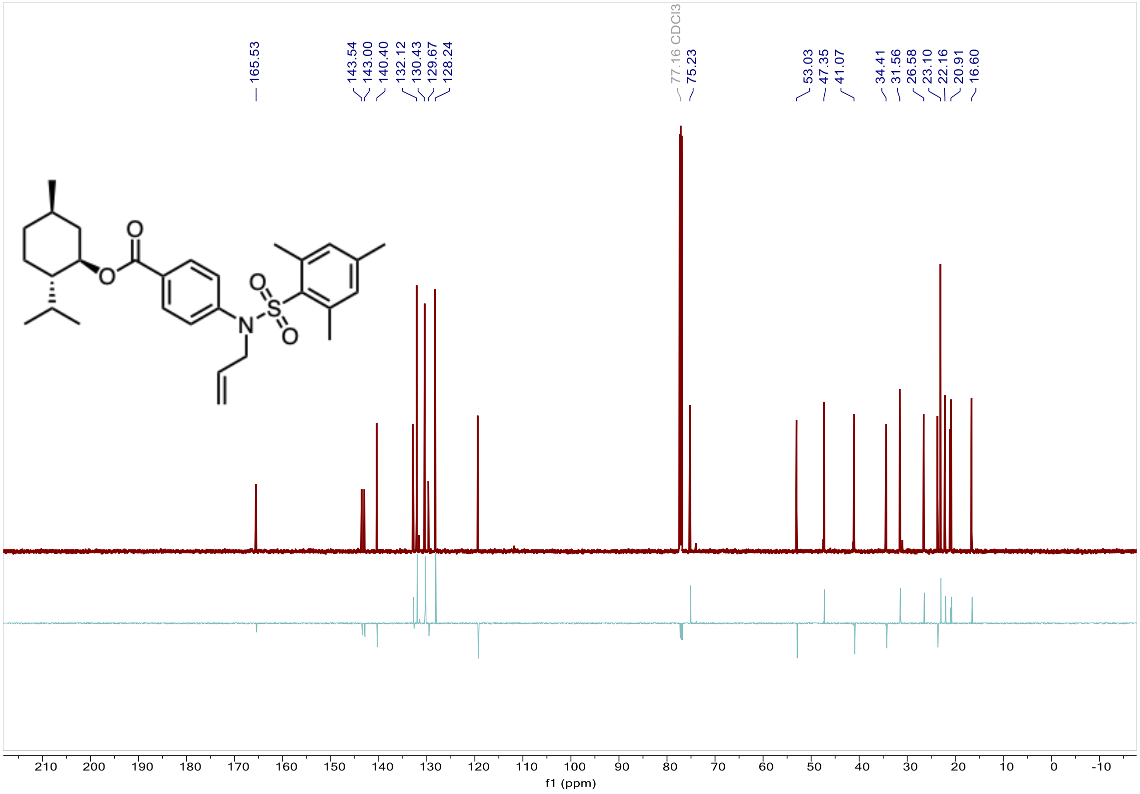

(*R*)-2,8-dimethyl-2-((4*R*,8*R*)-4,8,12-trimethyltridecyl)chroman-6-yl 4-((*N*-allyl-2,4,6-trimethylphenyl)sulfonamido)-2-methylbenzoate (1ak)

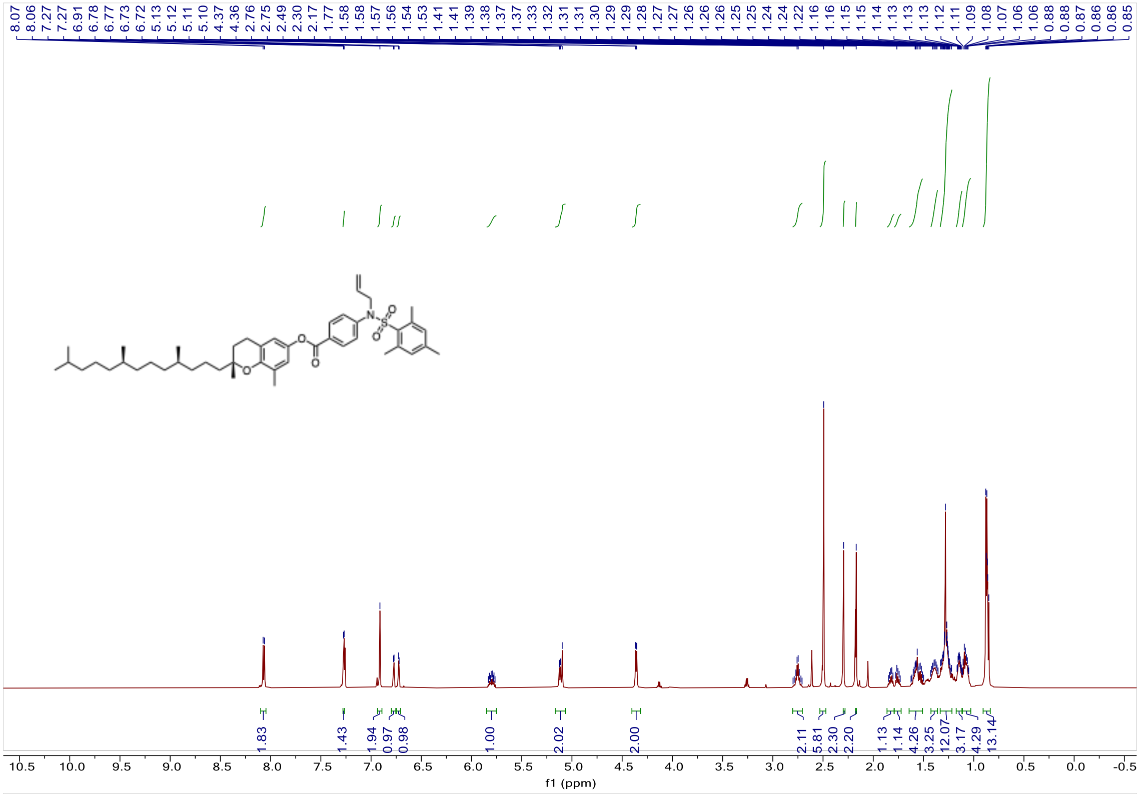

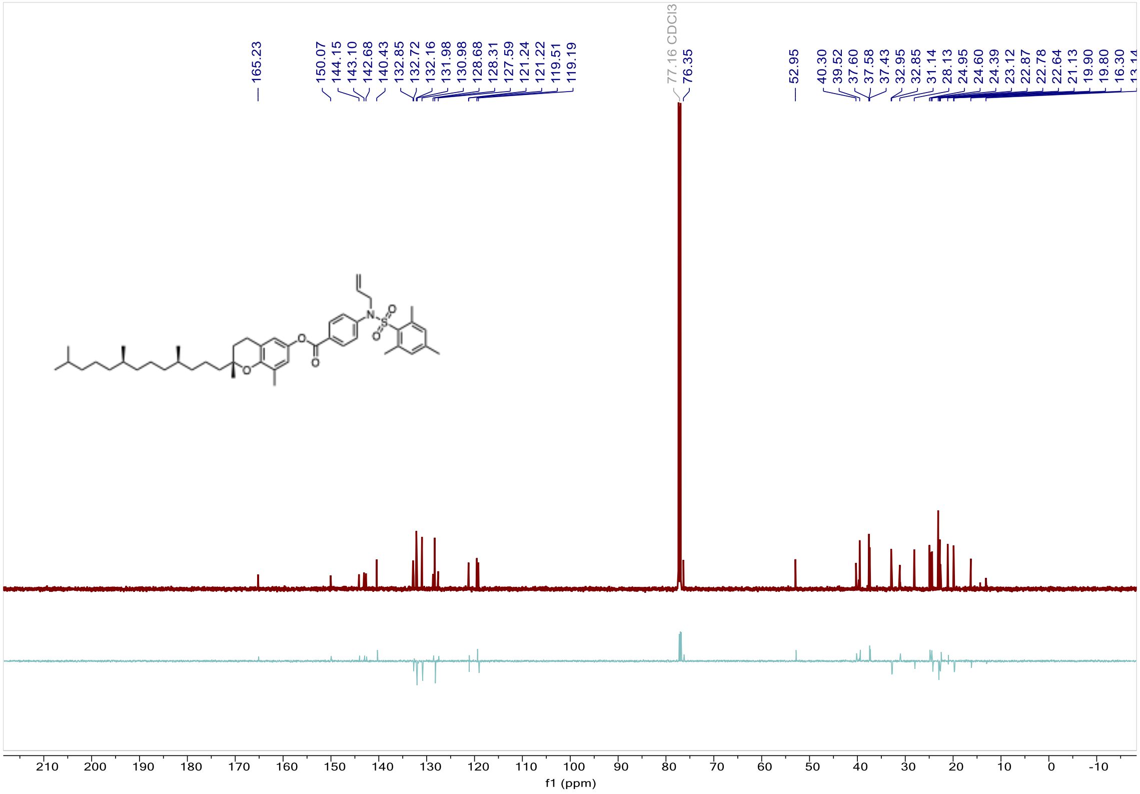

(*E*)-*N*-(but-2-en-1-yl)-2,4,6-trimethyl-*N*-phenylbenzenesulfonamide (1al)

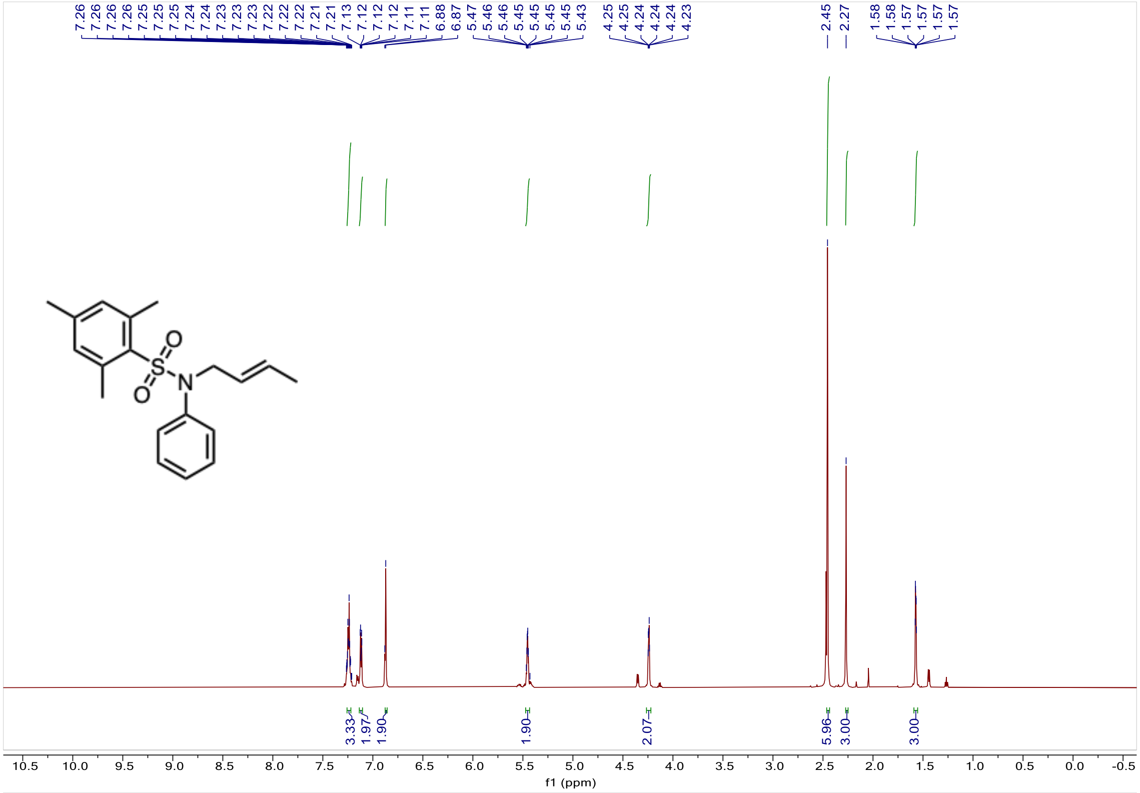

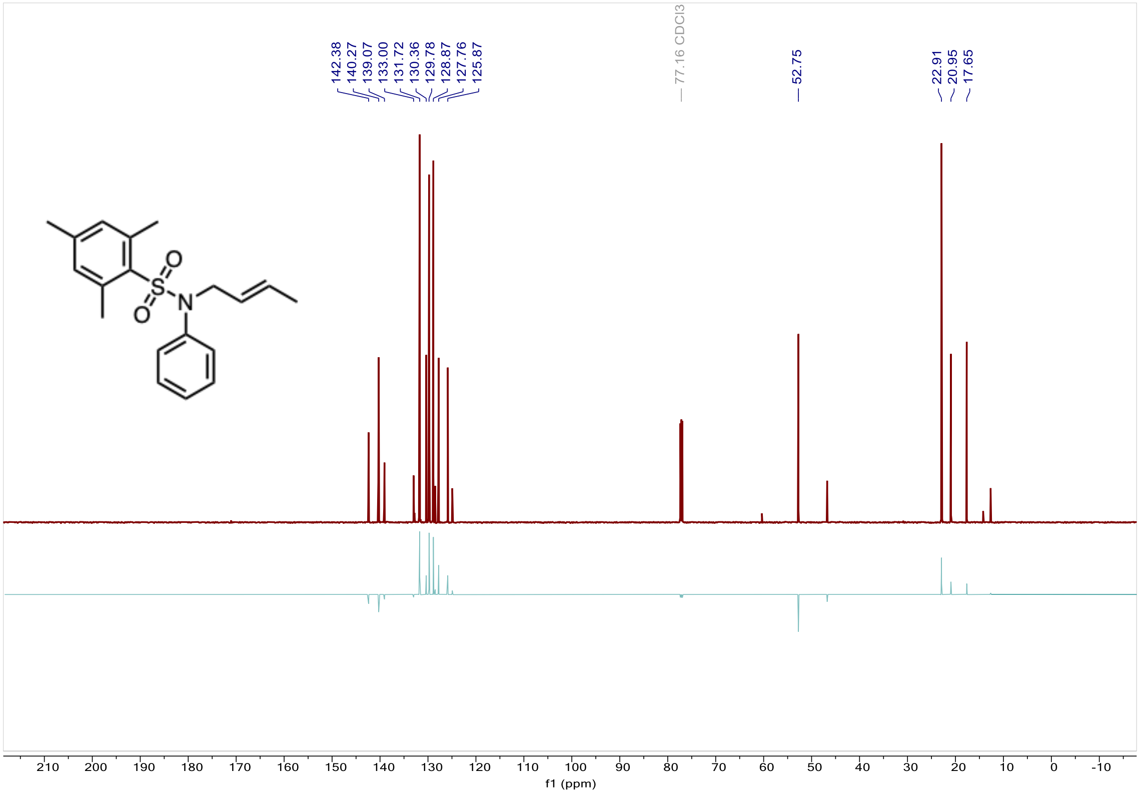

***N*-(cyclohex-2-en-1-yl)-2,4,6-trimethyl-*N*-phenylbenzenesulfonamide(1am)**

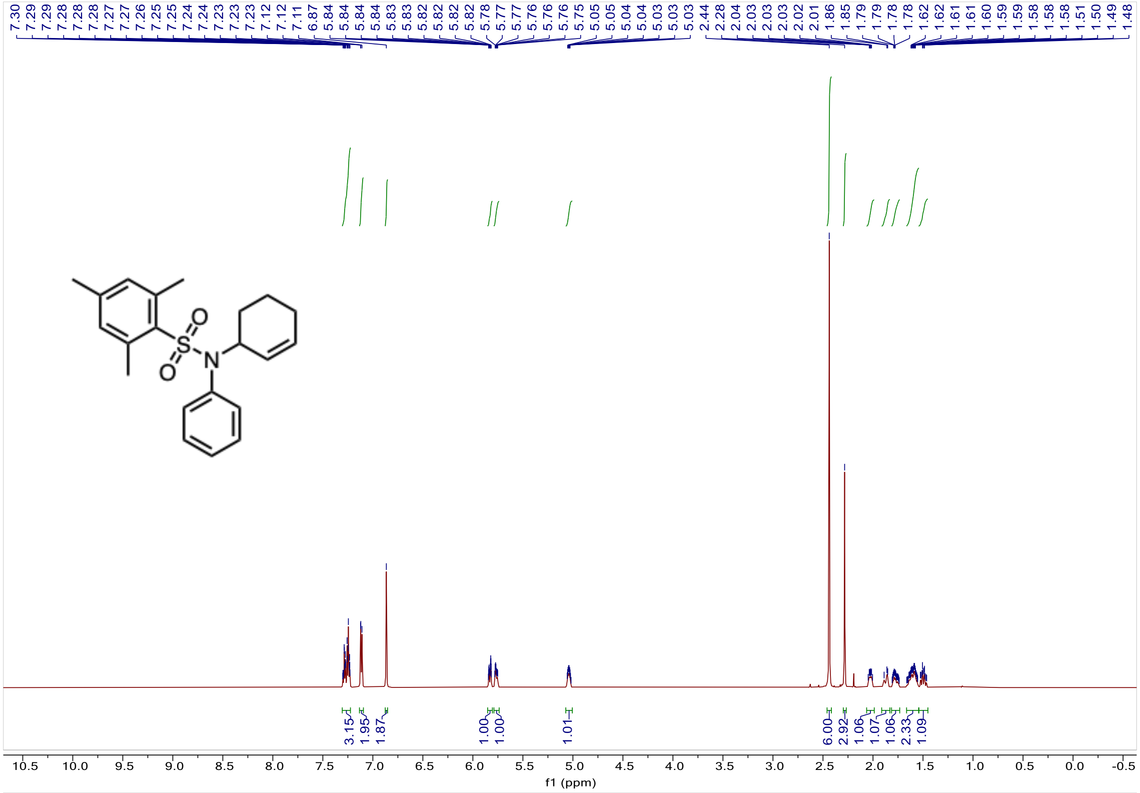

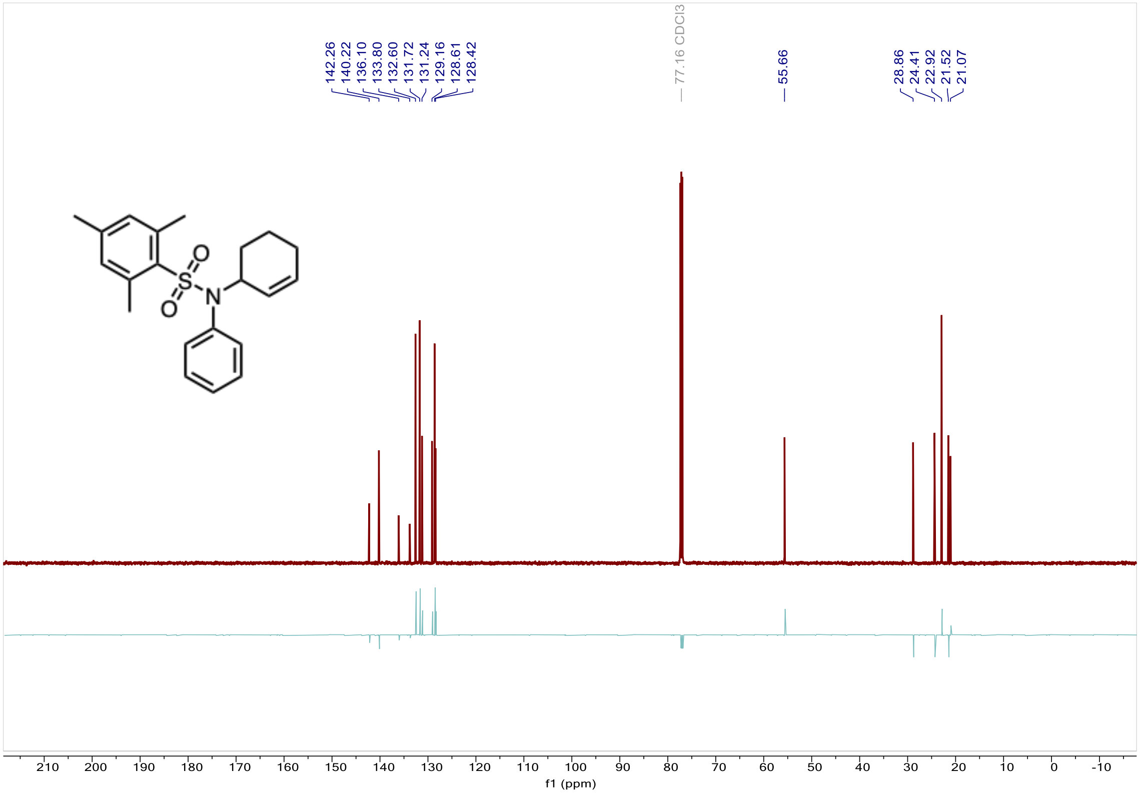

***N*-(mesitylsulfonyl)-*N*-phenylacrylamide (1an)**

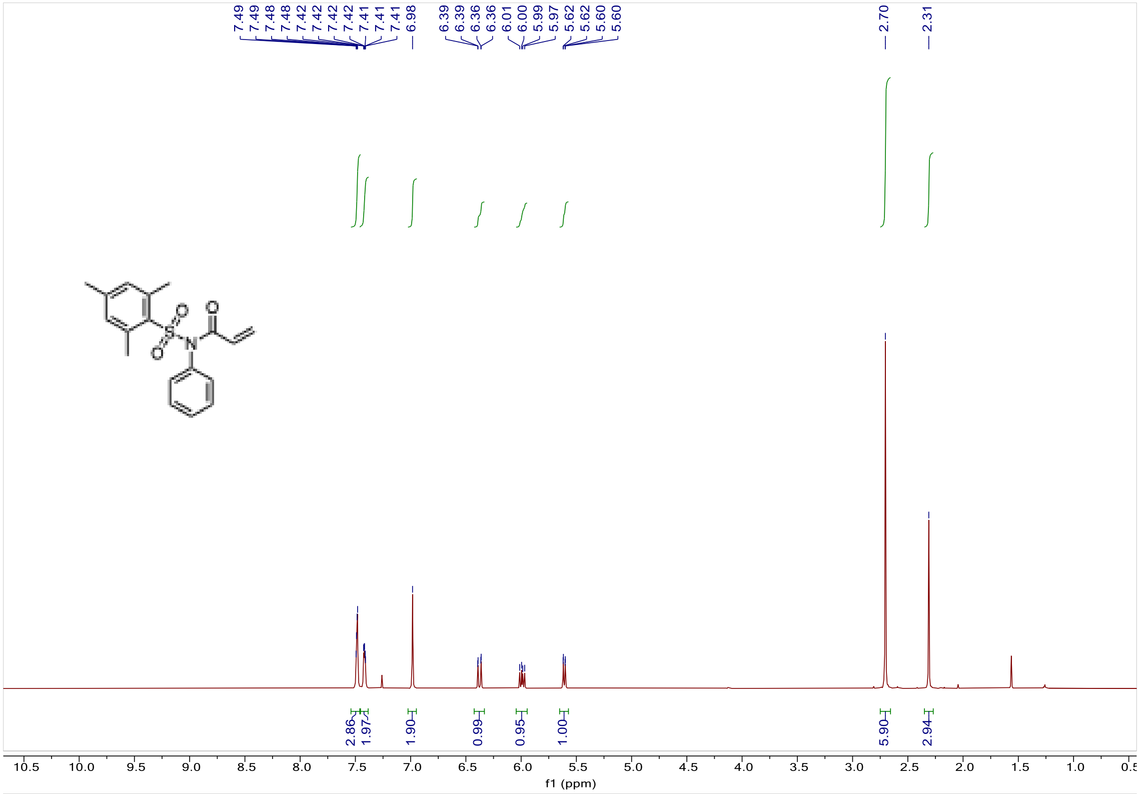

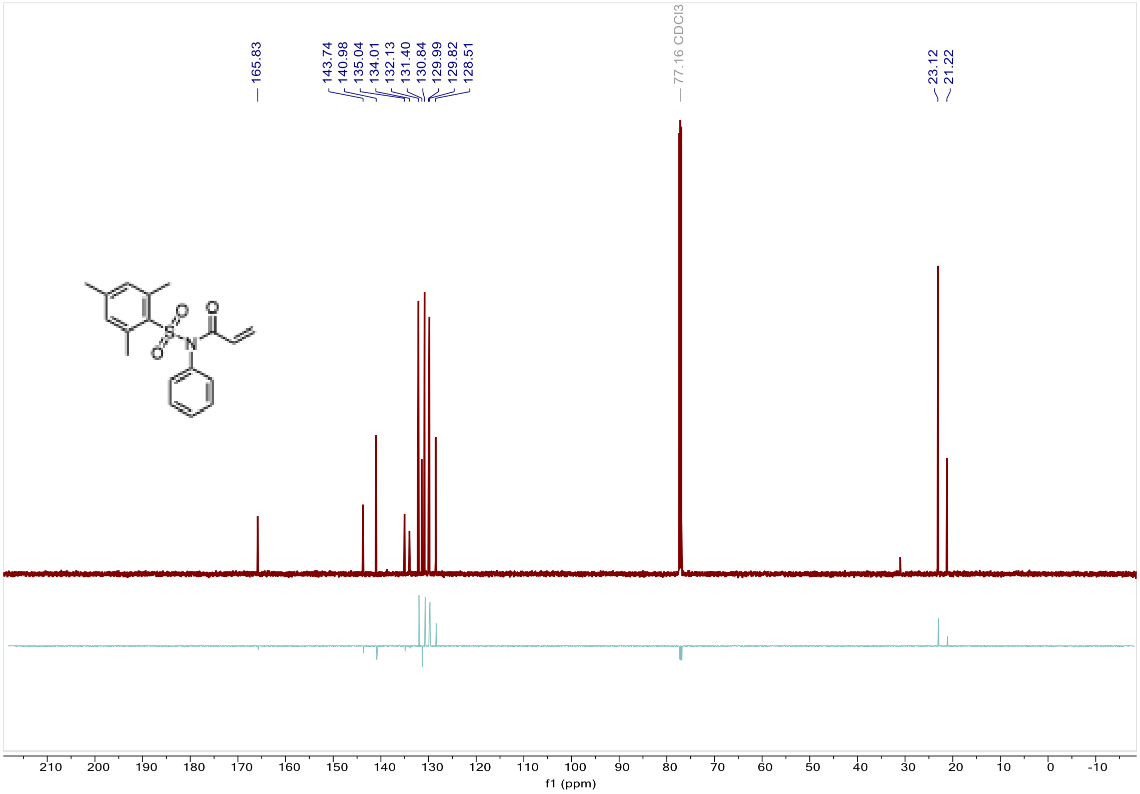

N-(2-phenylpropyl)aniline (2a)

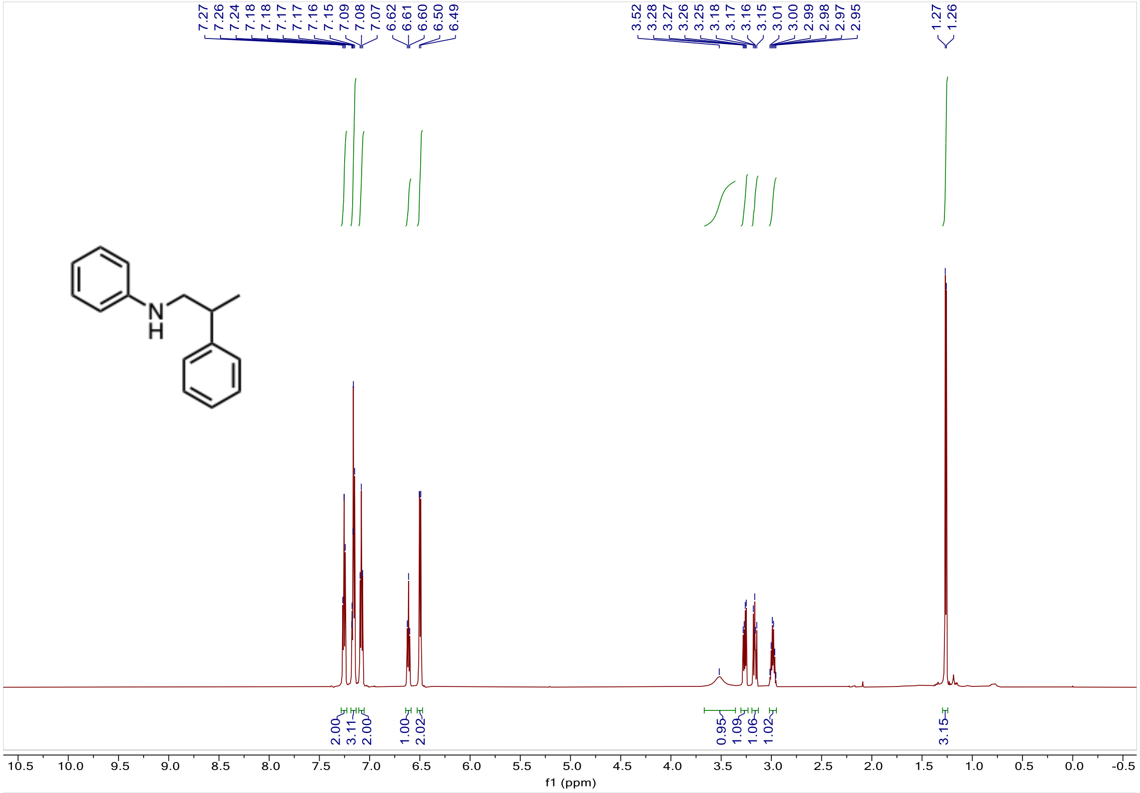

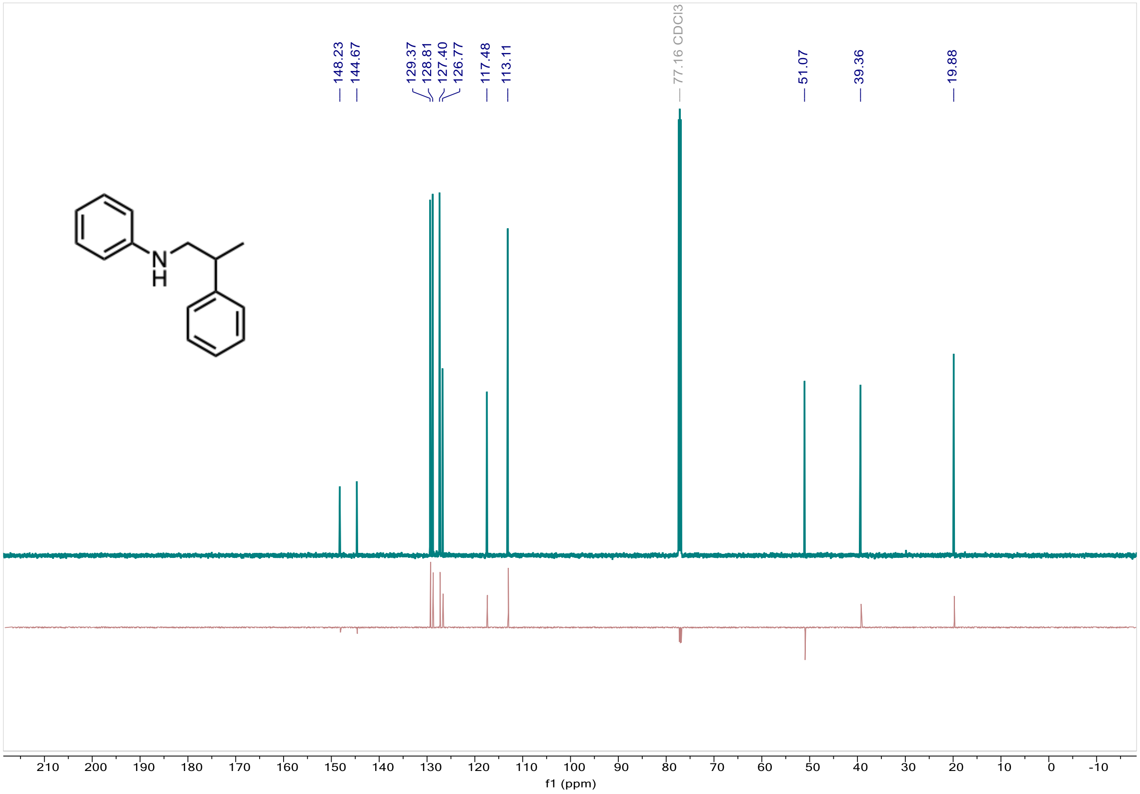

N-(2-mesitylpropyl)aniline (2b)

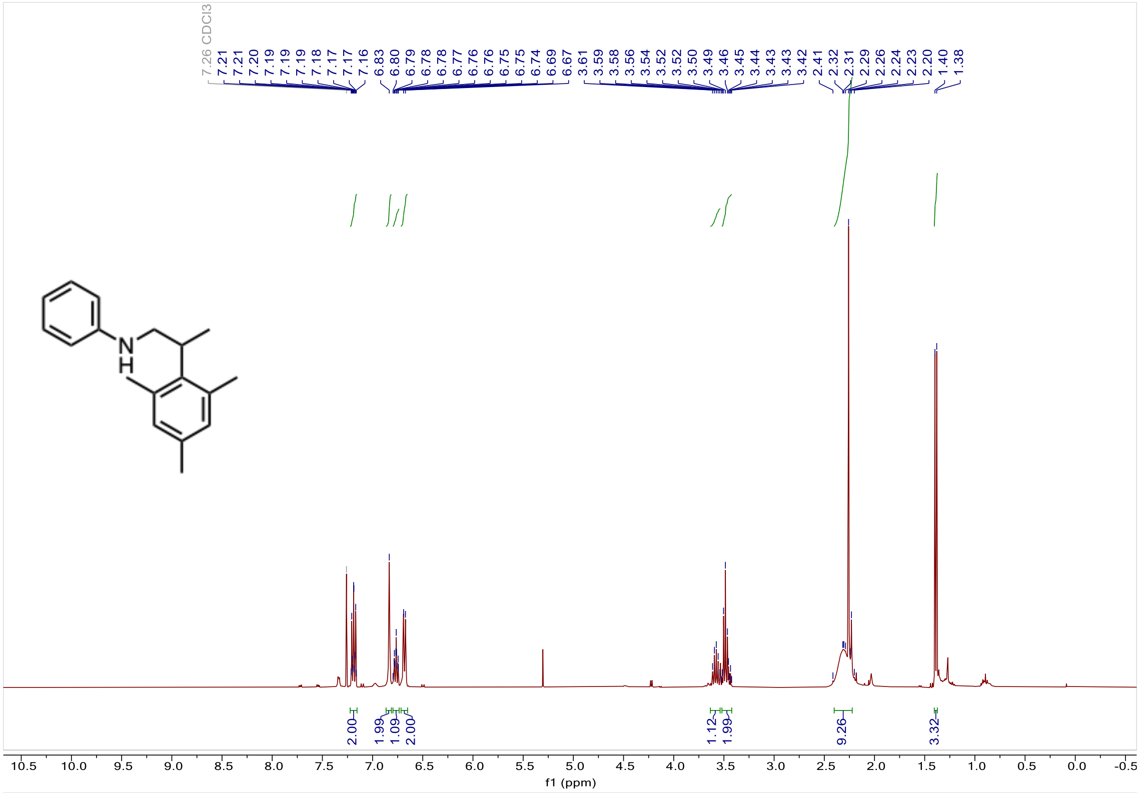

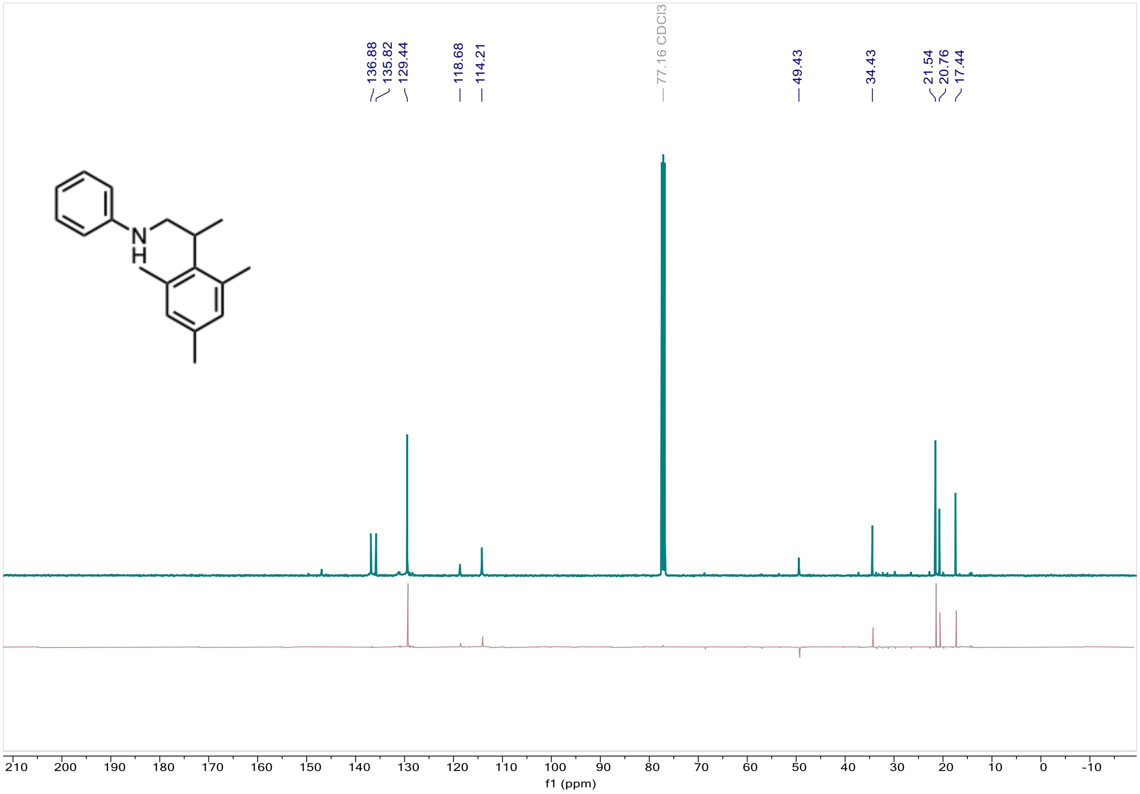

N-(2-(*o*-tolyl)propyl)aniline (2c)

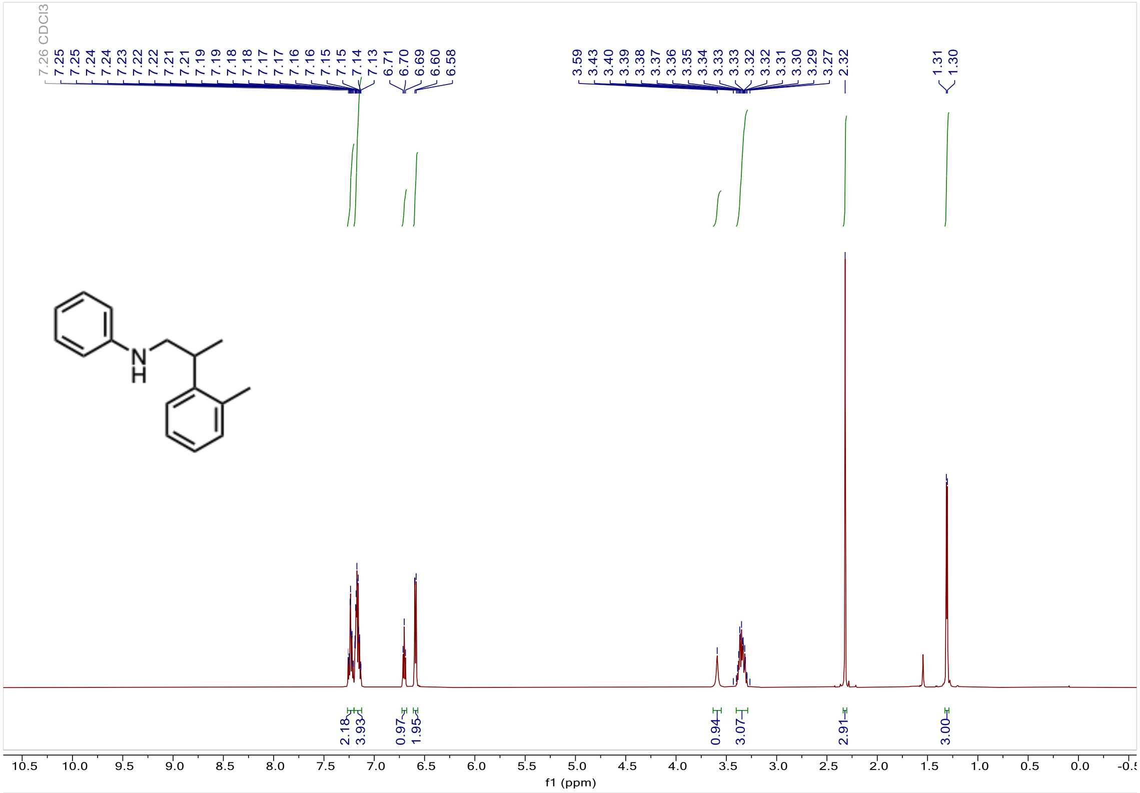

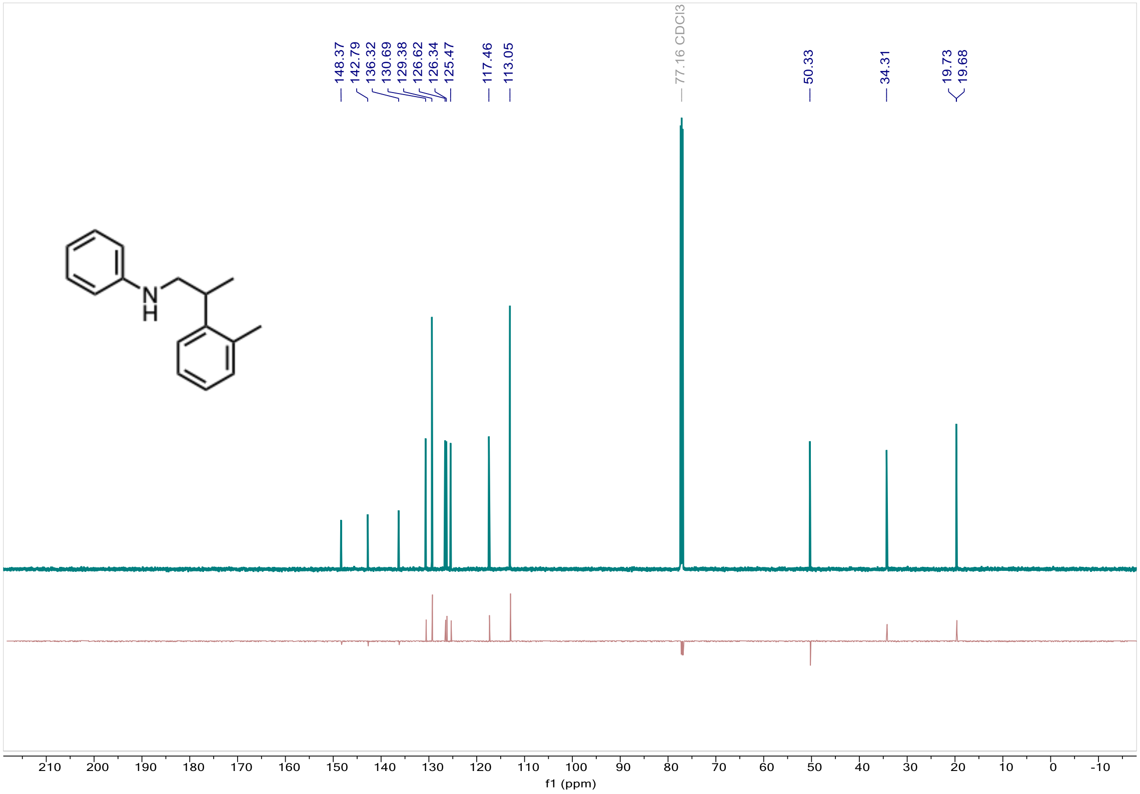

N-(2-(2-methoxyphenyl)propyl)aniline (2d)

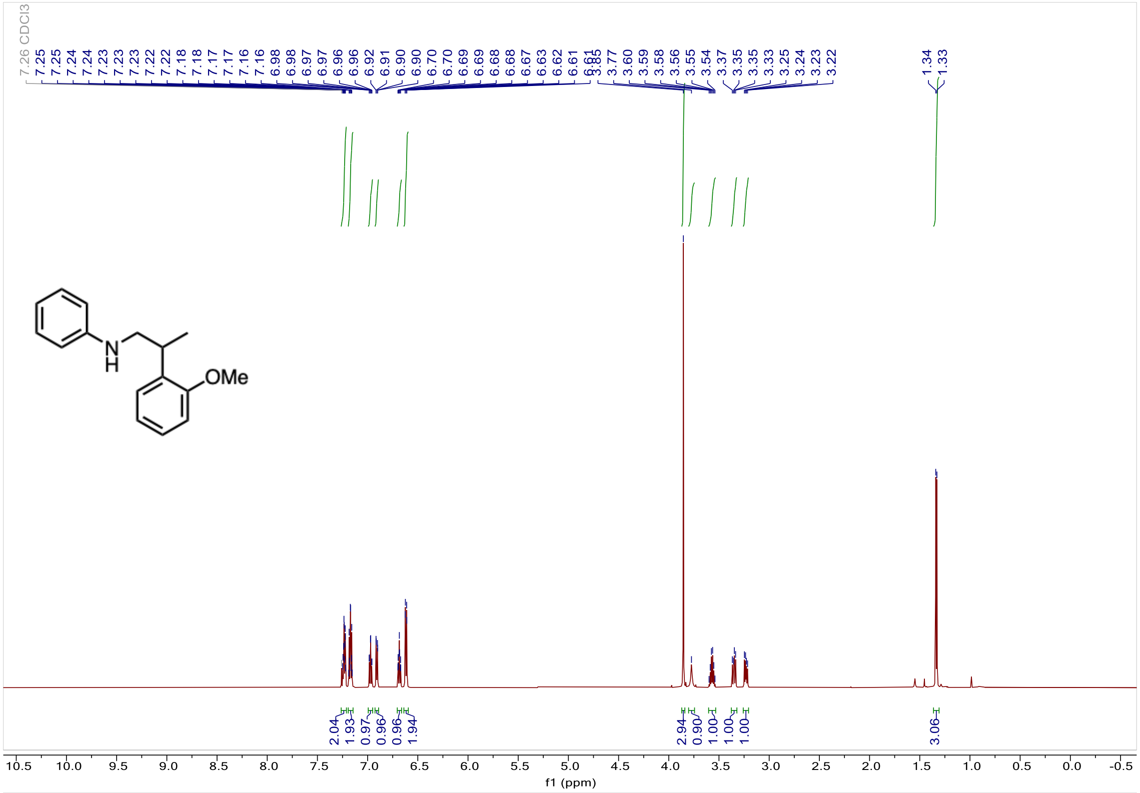

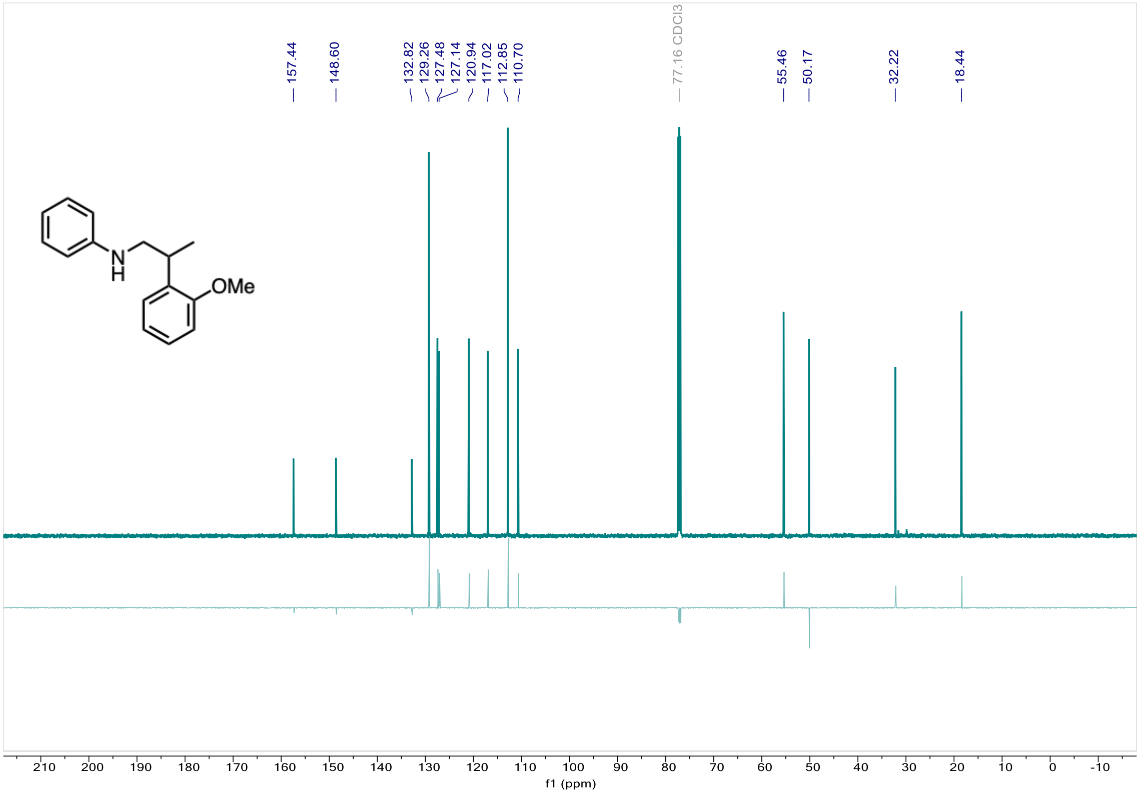

***N*-(2-(2-chlorophenyl)propyl)aniline (2e)**

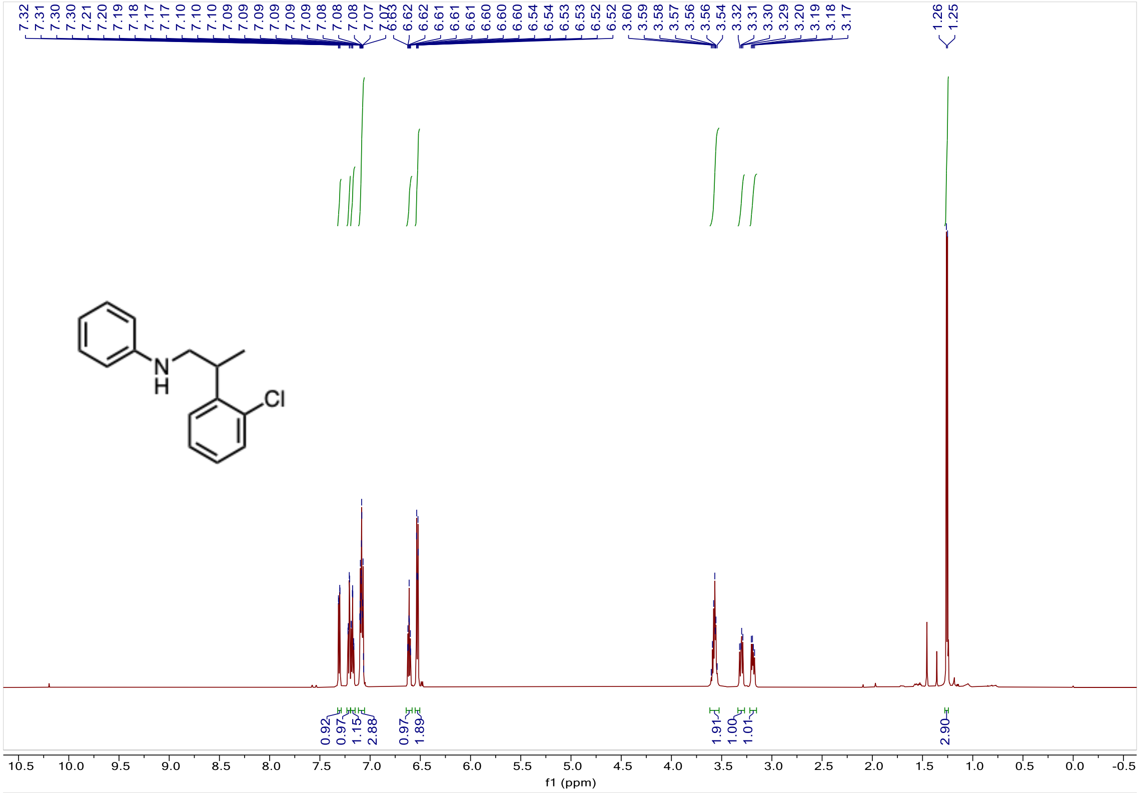

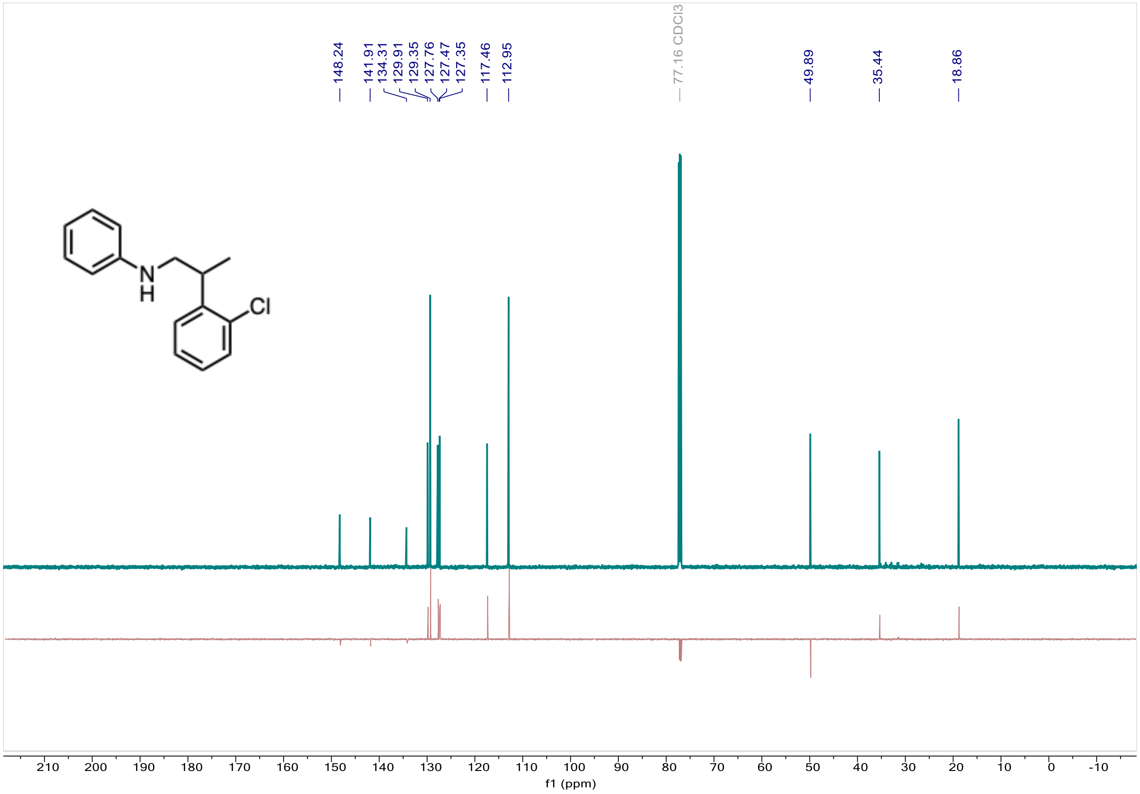

N-(2-(2-bromophenyl)propyl)aniline (2f)

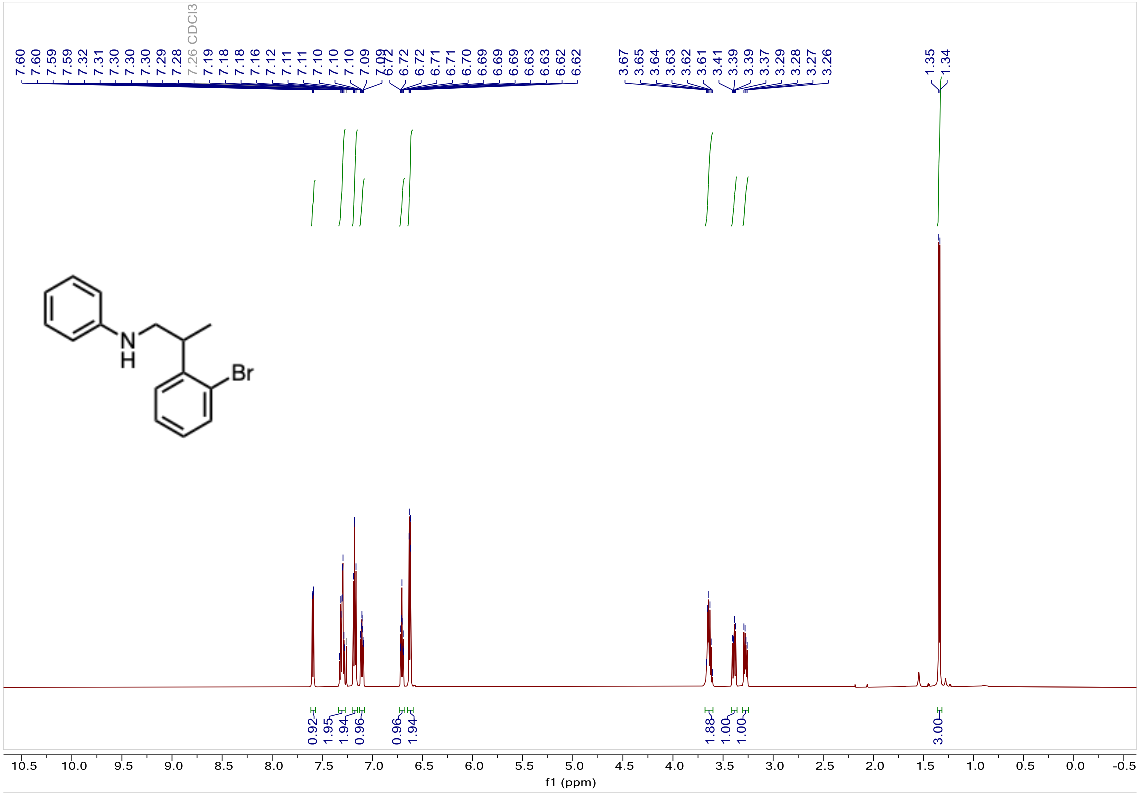

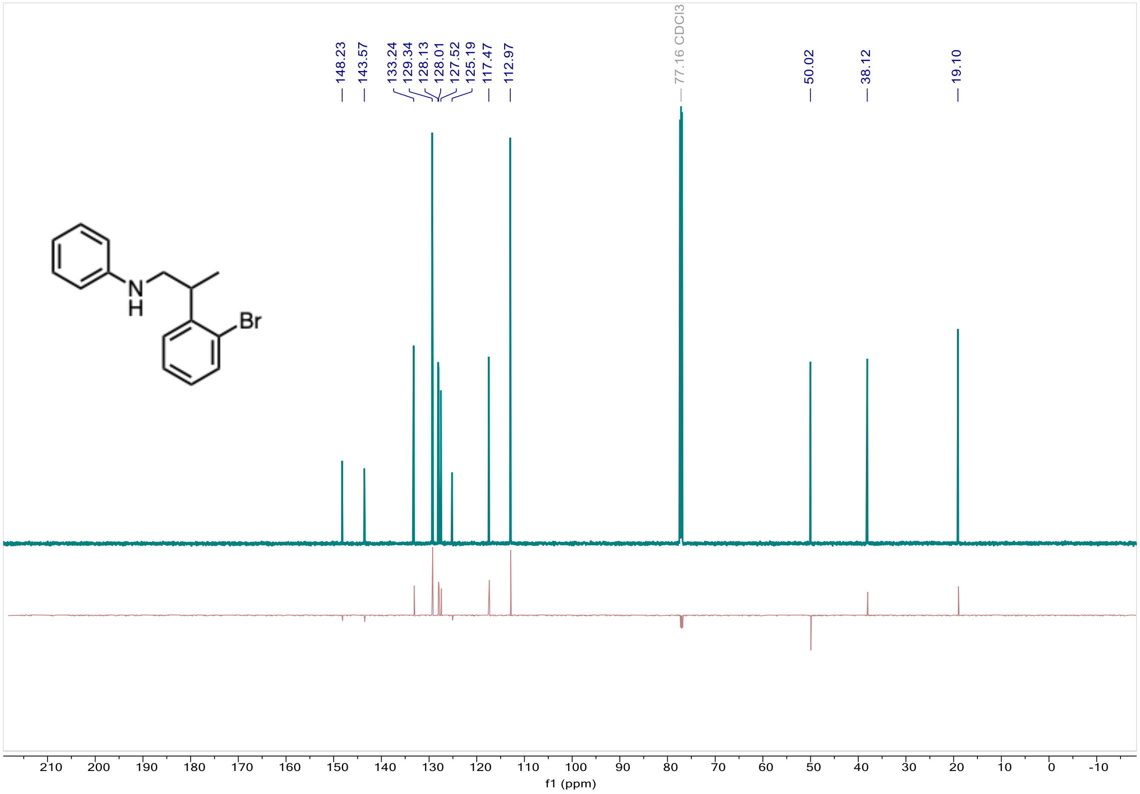

N-(2-(2-(trifluoromethyl)phenyl)propyl)aniline (2g)

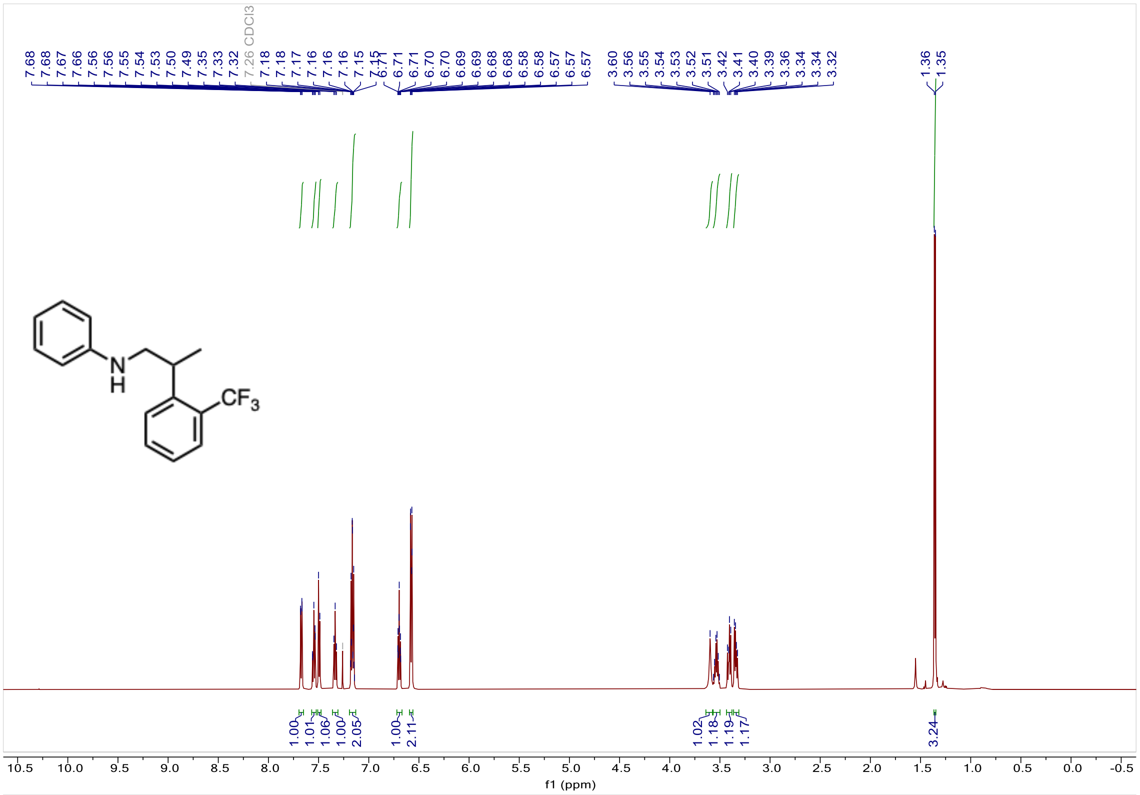

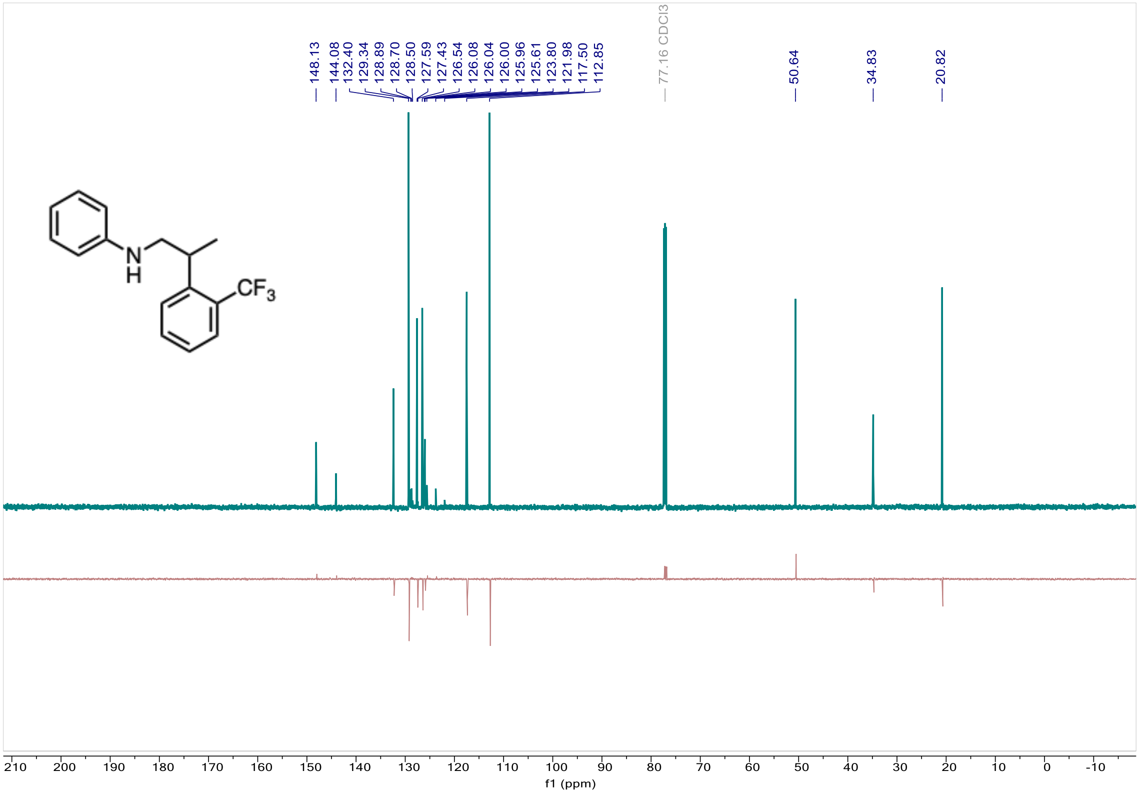

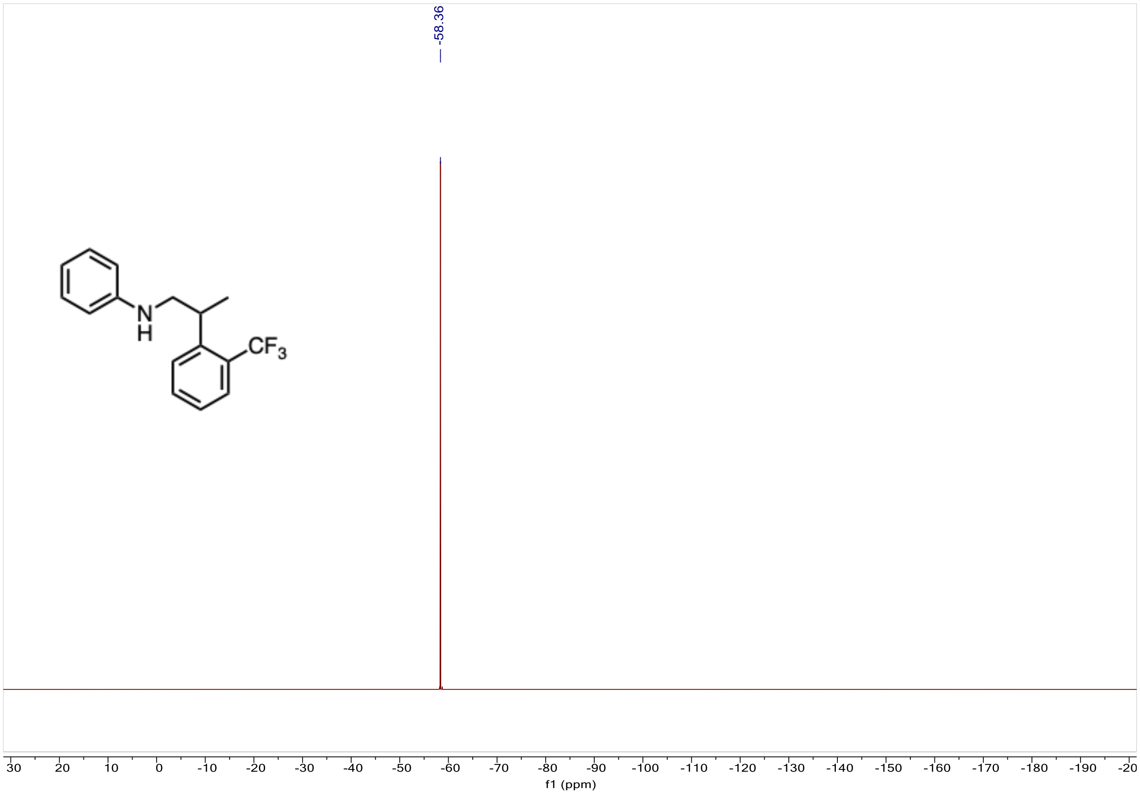

Methyl 2-(1-(phenylamino)propan-2-yl)benzoate (2h)

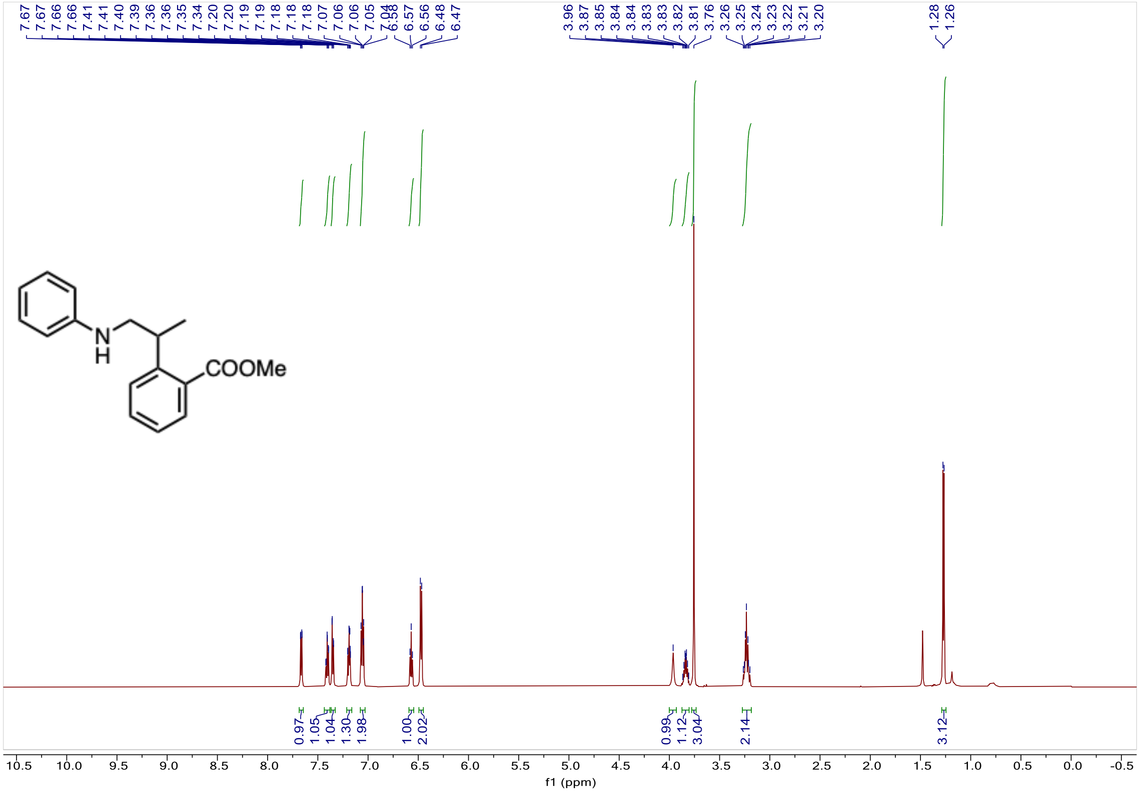

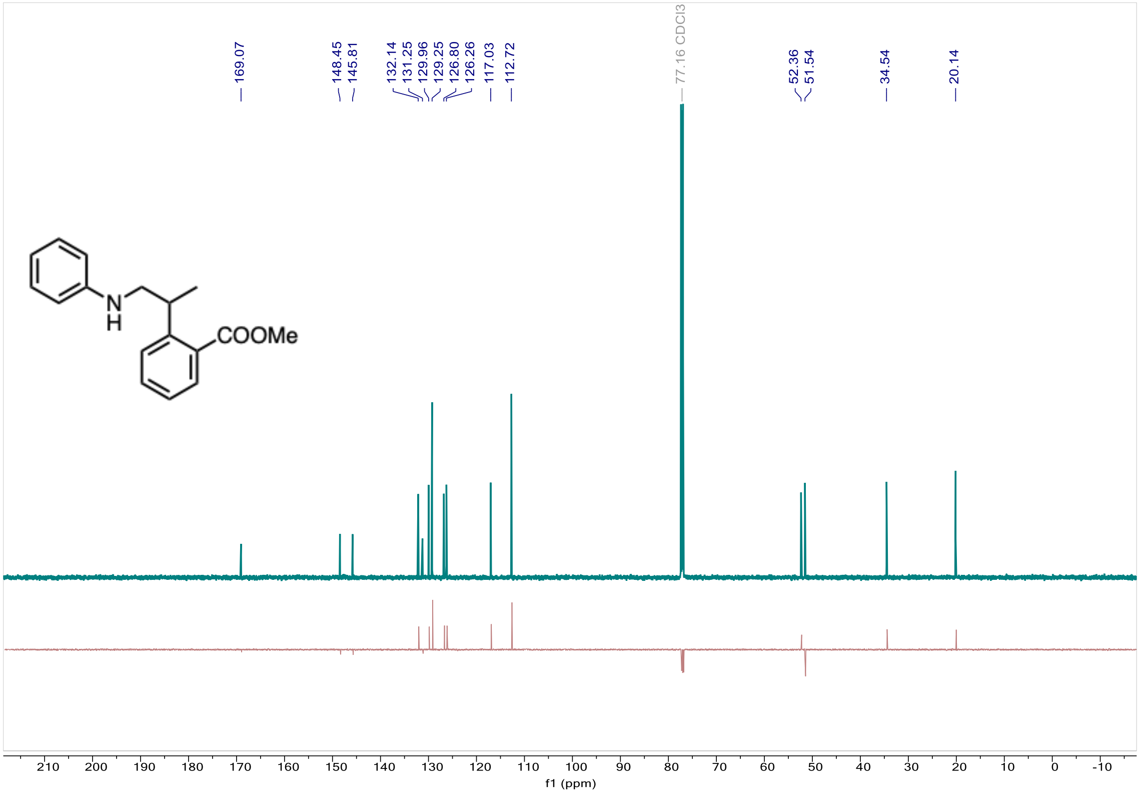

N-(2-(2,6-dimethoxyphenyl)propyl)aniline (2i)

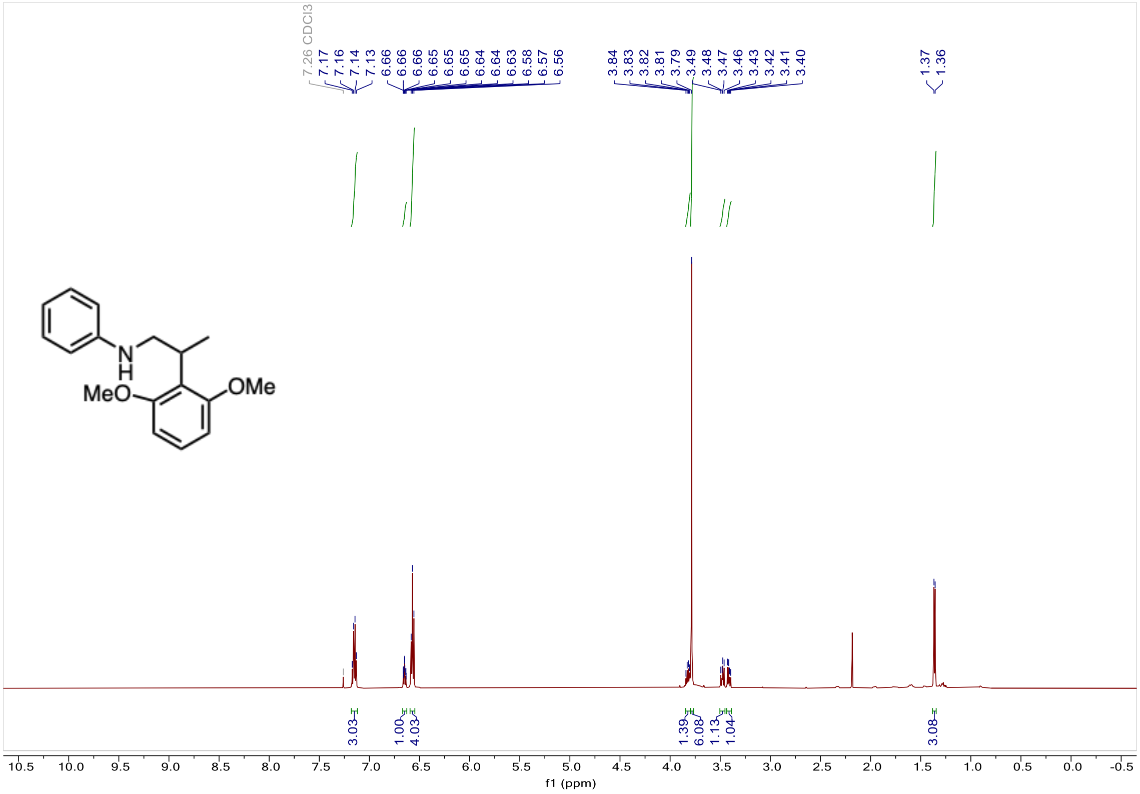

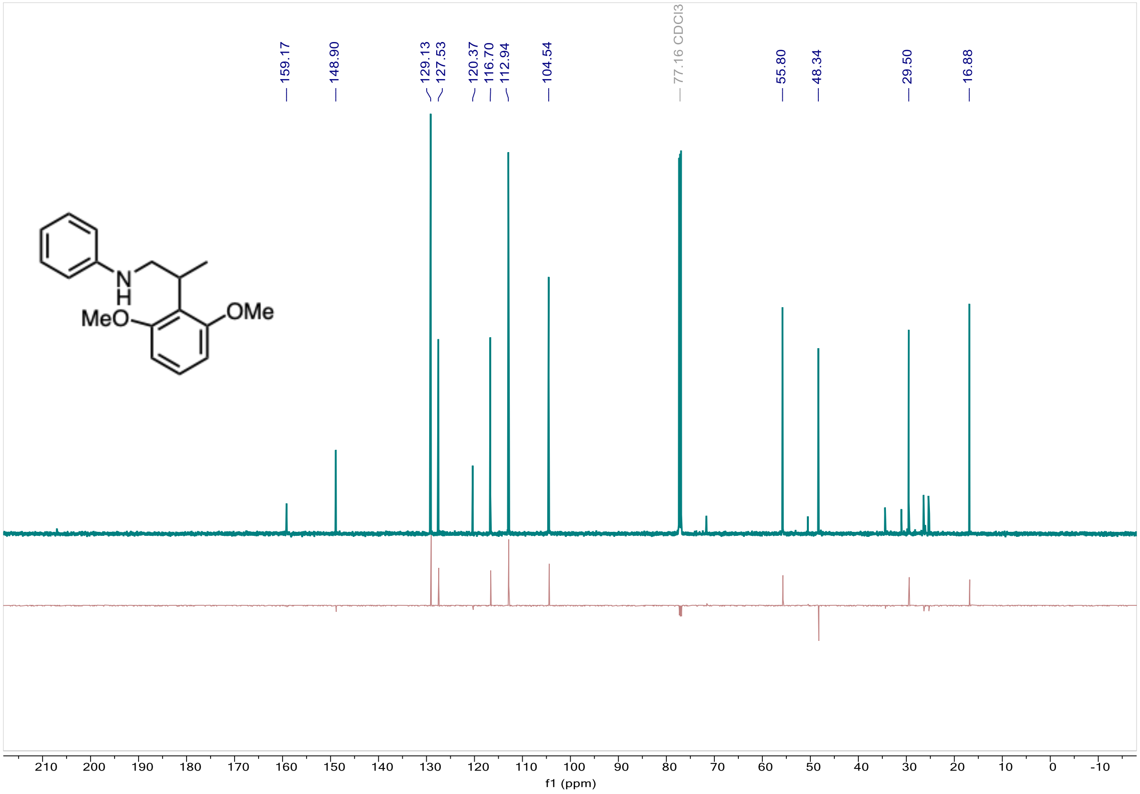

N-(2-(2,6-dichlorophenyl)propyl)aniline (2j)

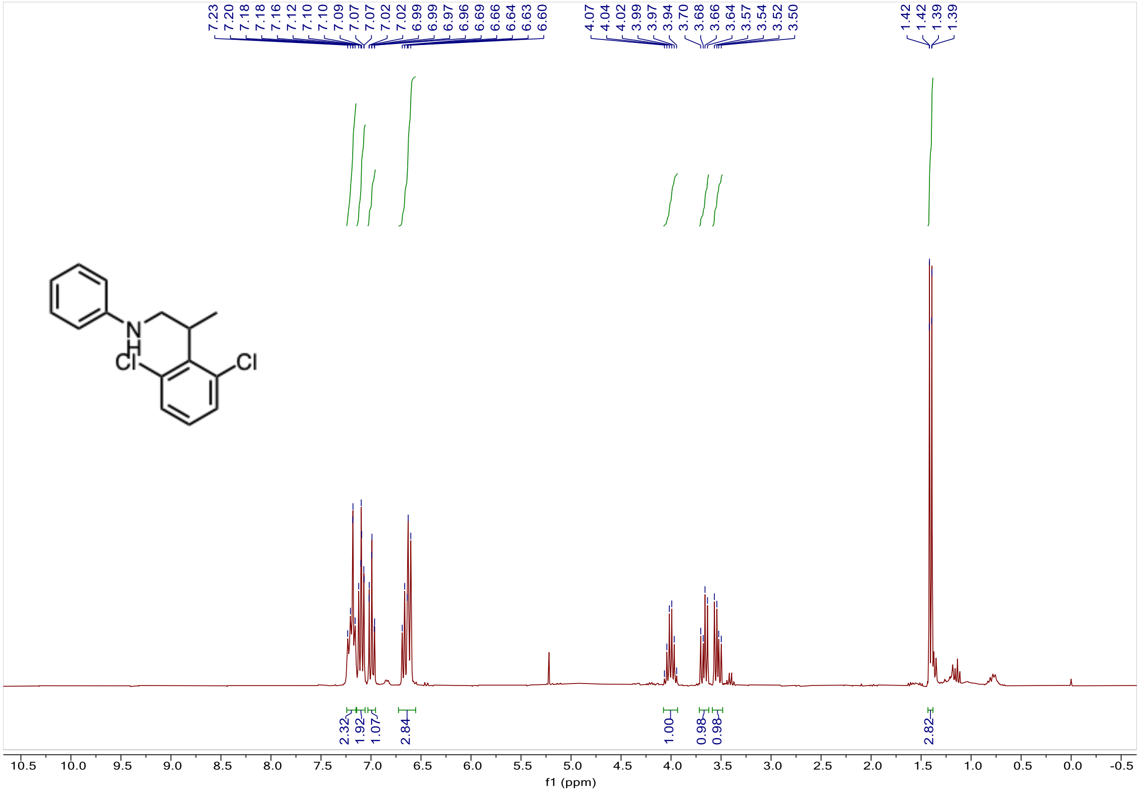

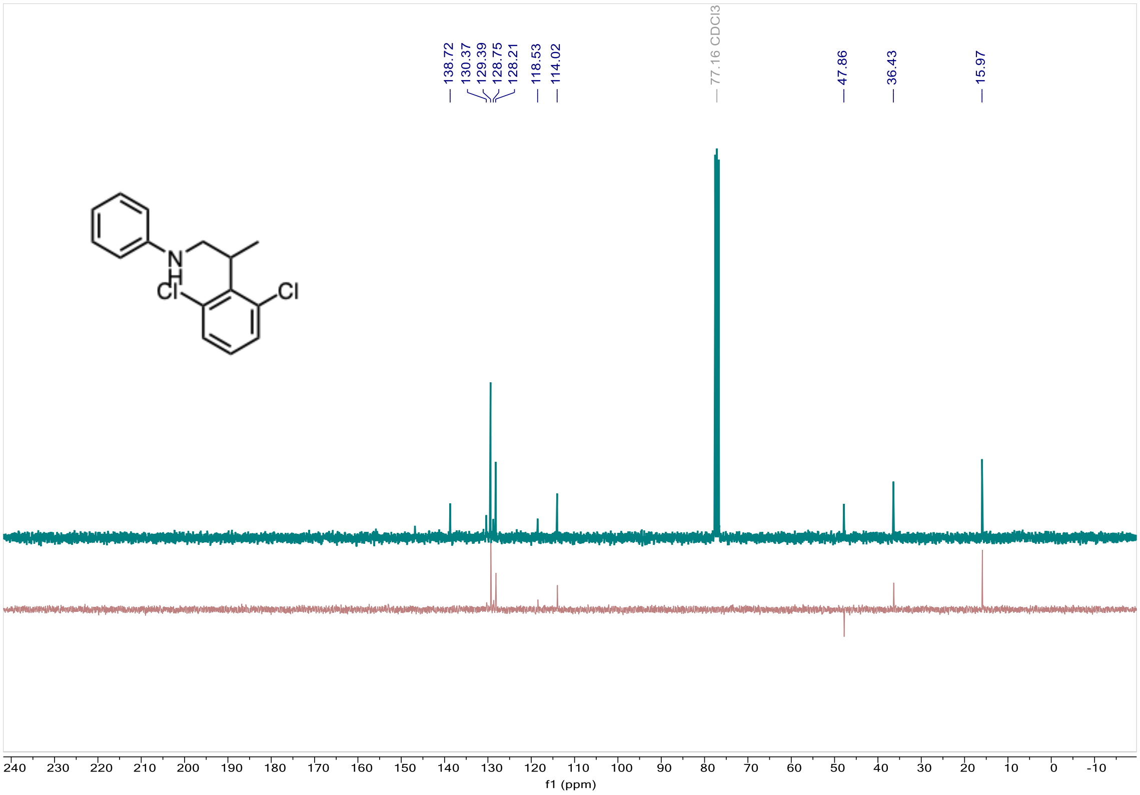

N-(2-(4-(trifluoromethyl)phenyl)propyl)aniline (2k)

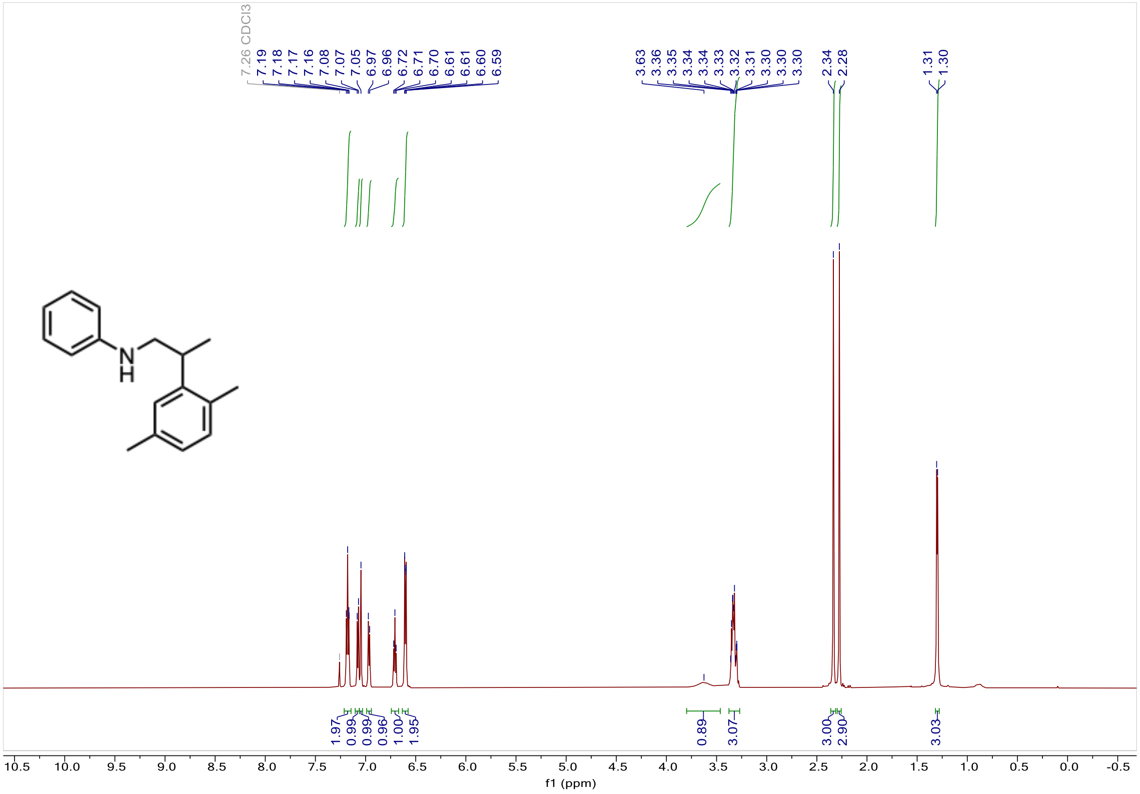

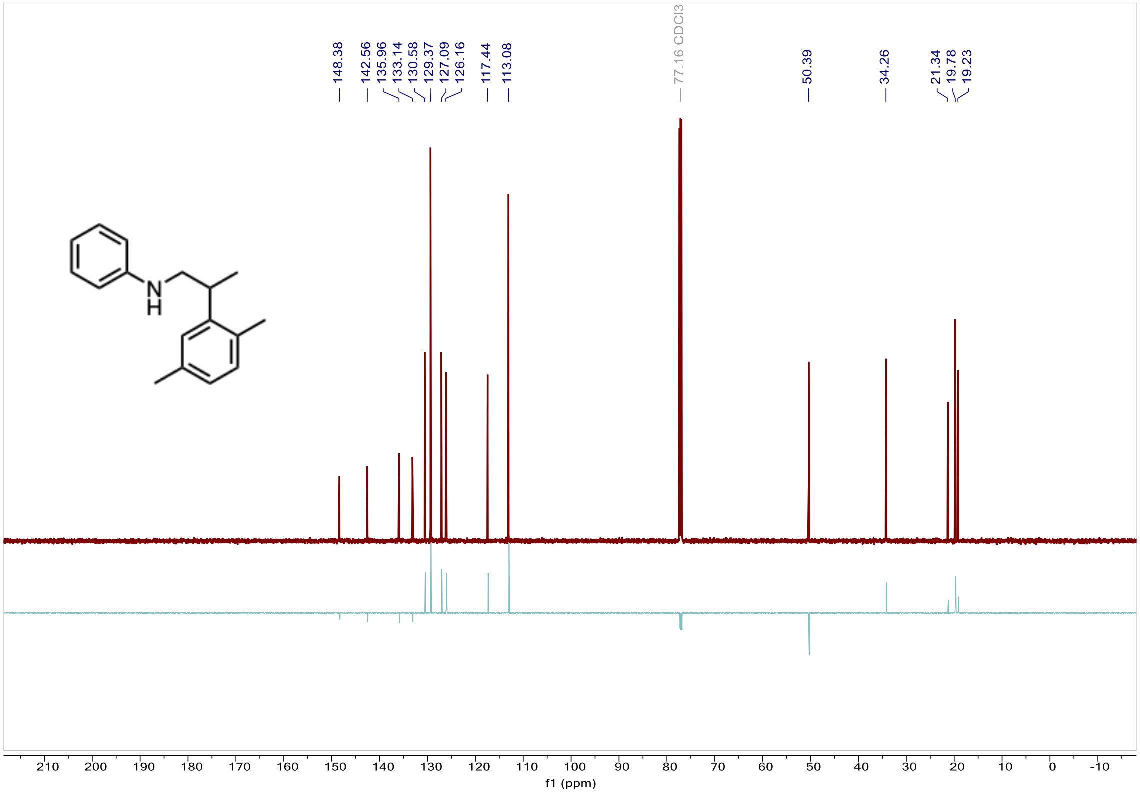

N-(2-(2,4-dimethylphenyl)propyl)aniline (2l)

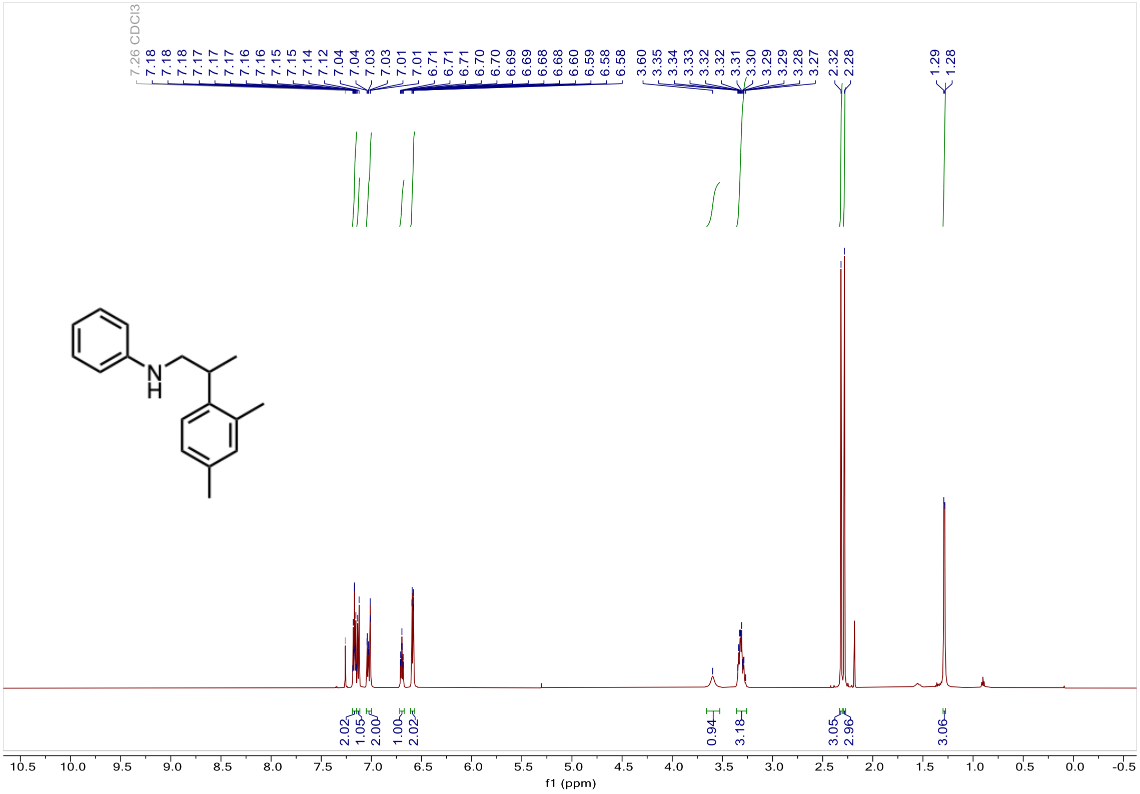

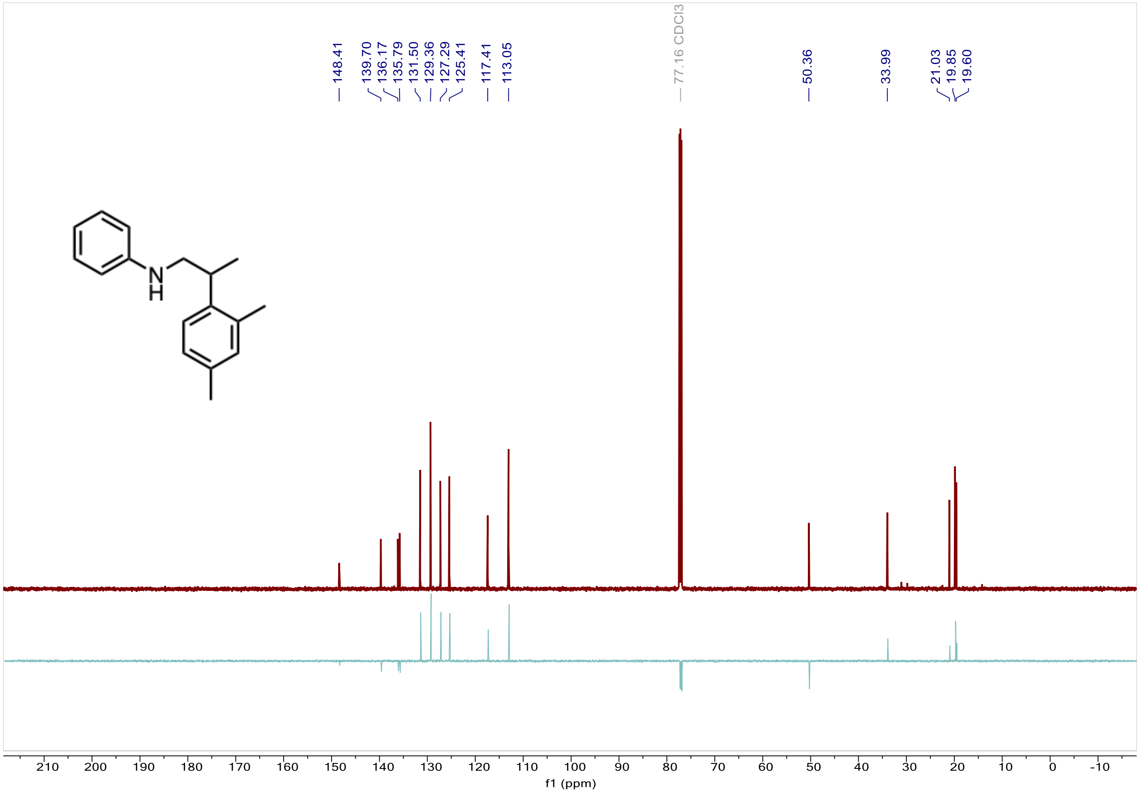

N-(2-(2,3,4,5,6-pentamethylphenyl)propyl)aniline (2m)

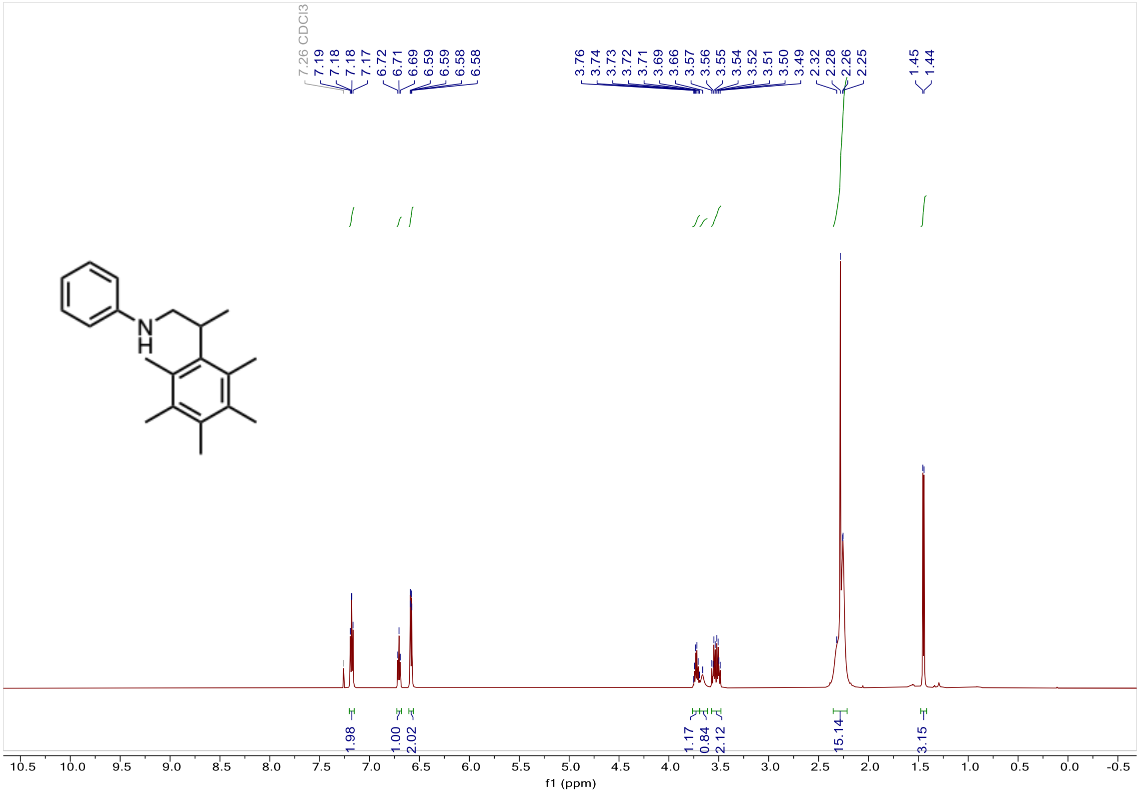

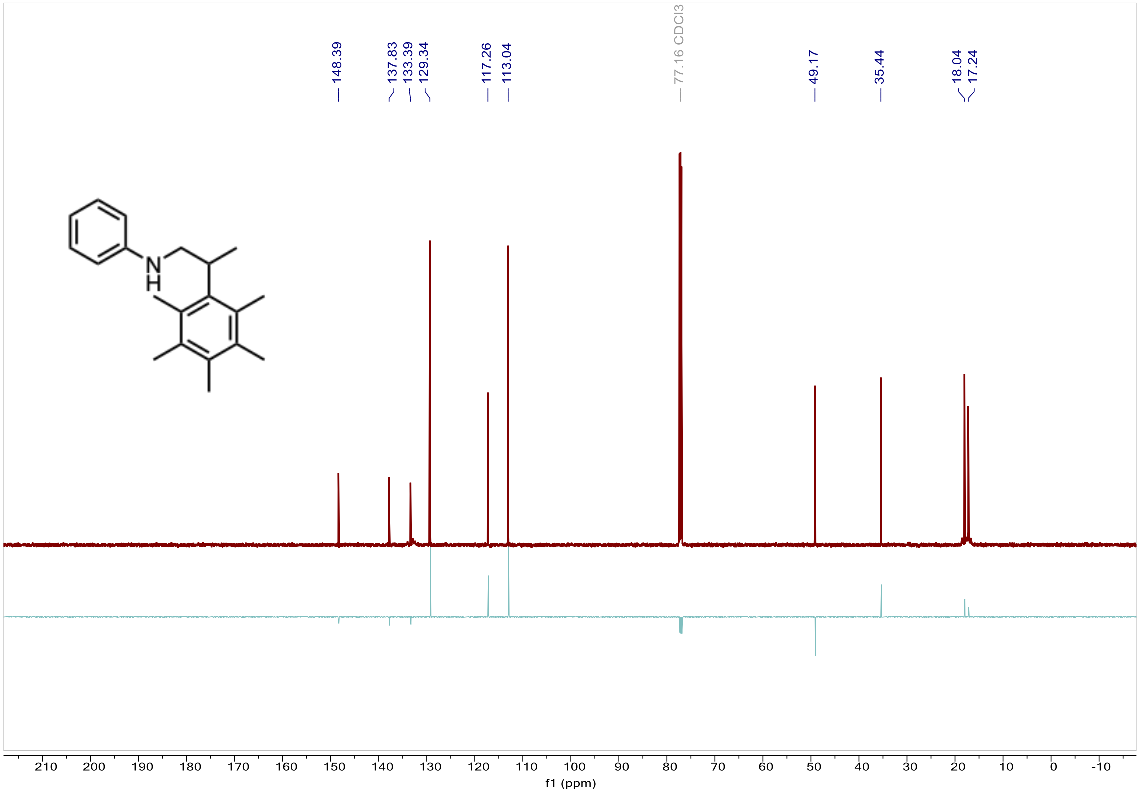

N-(2-(2,4,6-triisopropylphenyl)propyl)aniline (2n)

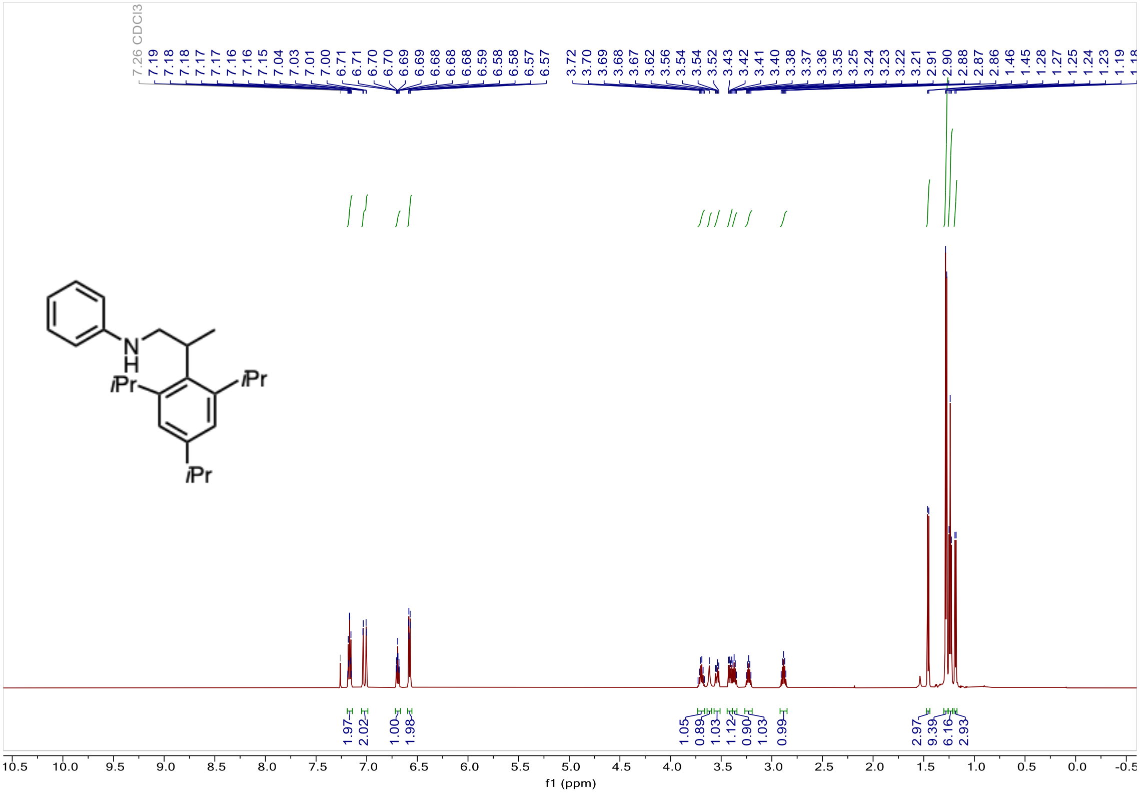

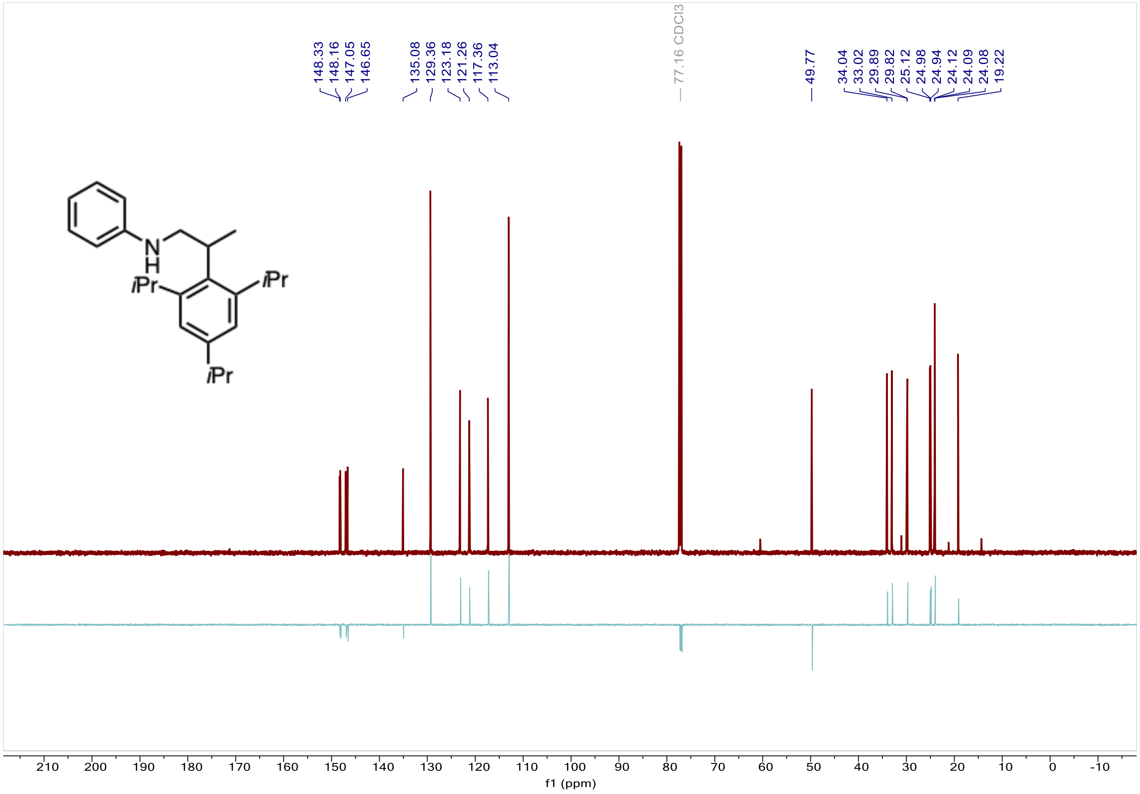

N-(2-(4-methoxyphenyl)propyl)aniline (2o)

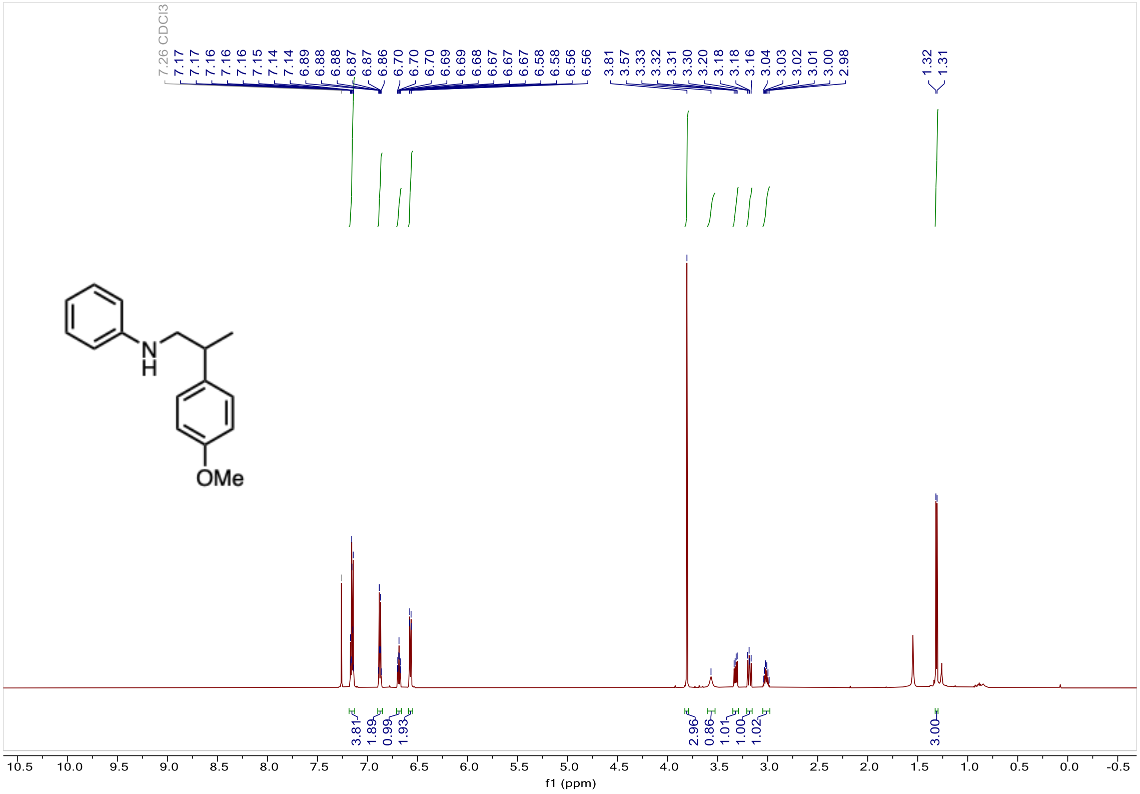

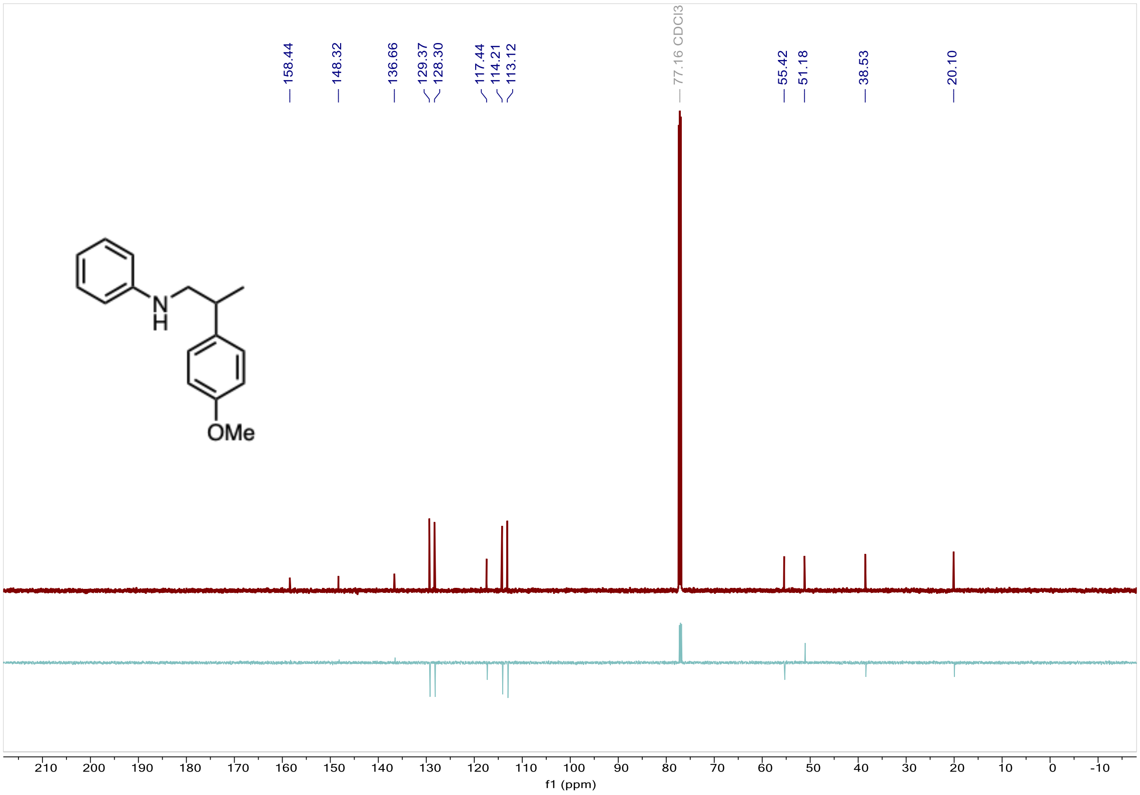

_N_-(2-(4-(trifluoromethyl)phenyl)propyl)aniline (2p)

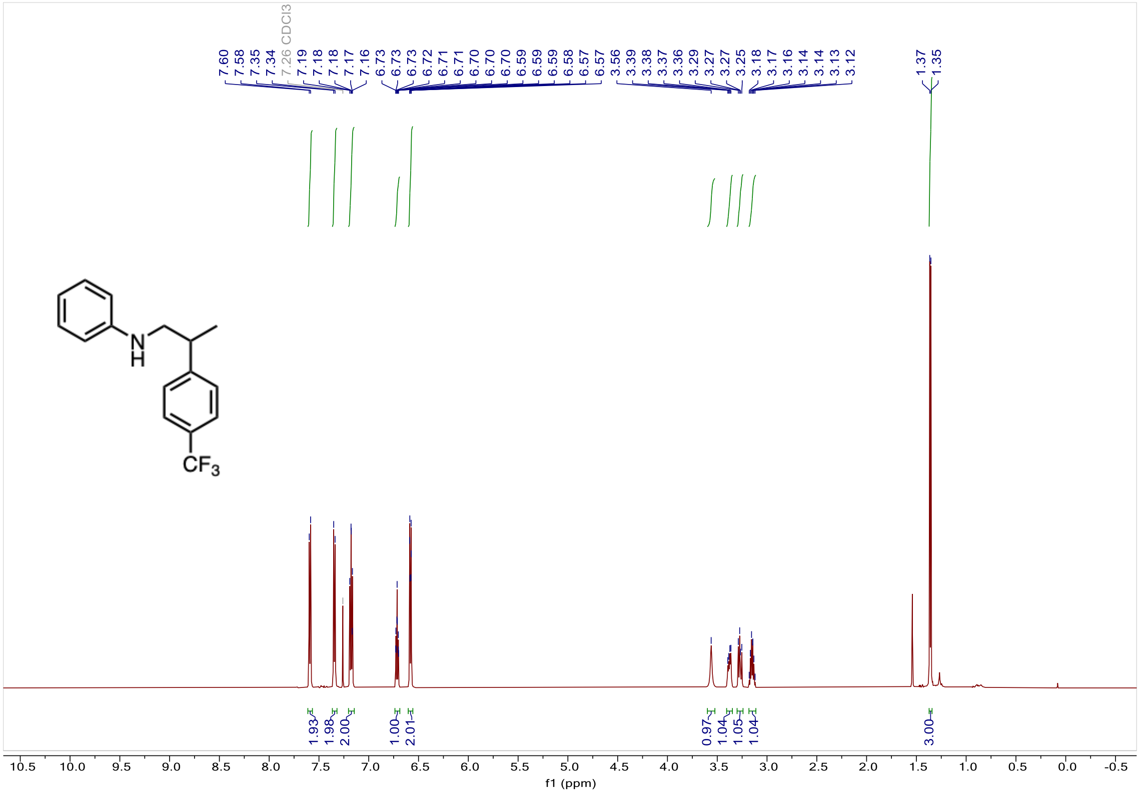

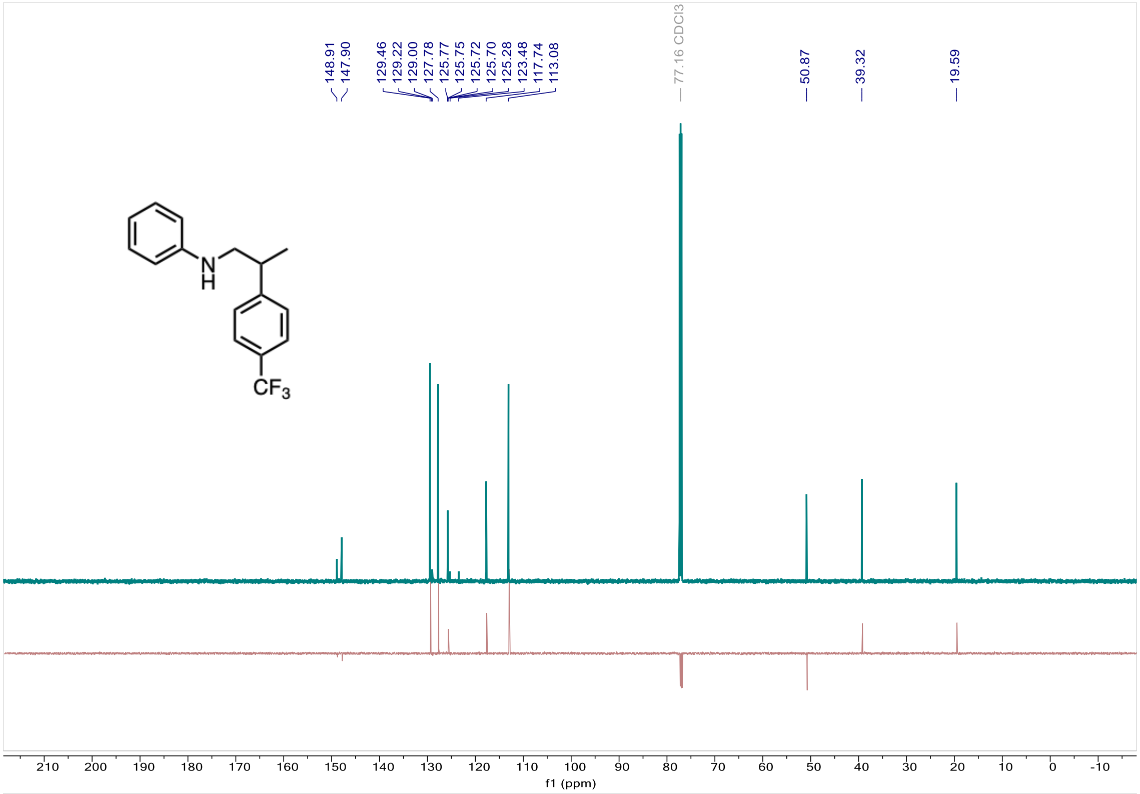
148.91
147.90
129.46
129.22
129.00
127.78
125.77
125.75
125.72
125.70
125.28
123.48
117.74
113.08
77.16 CDCl3
50.87
39.32
19.59
N
H
CF3
210 200 190 180 170 160 150 140 130 120 110 100 90 80 70 60 50 40 30 20 10 0 -10
f1 (ppm)

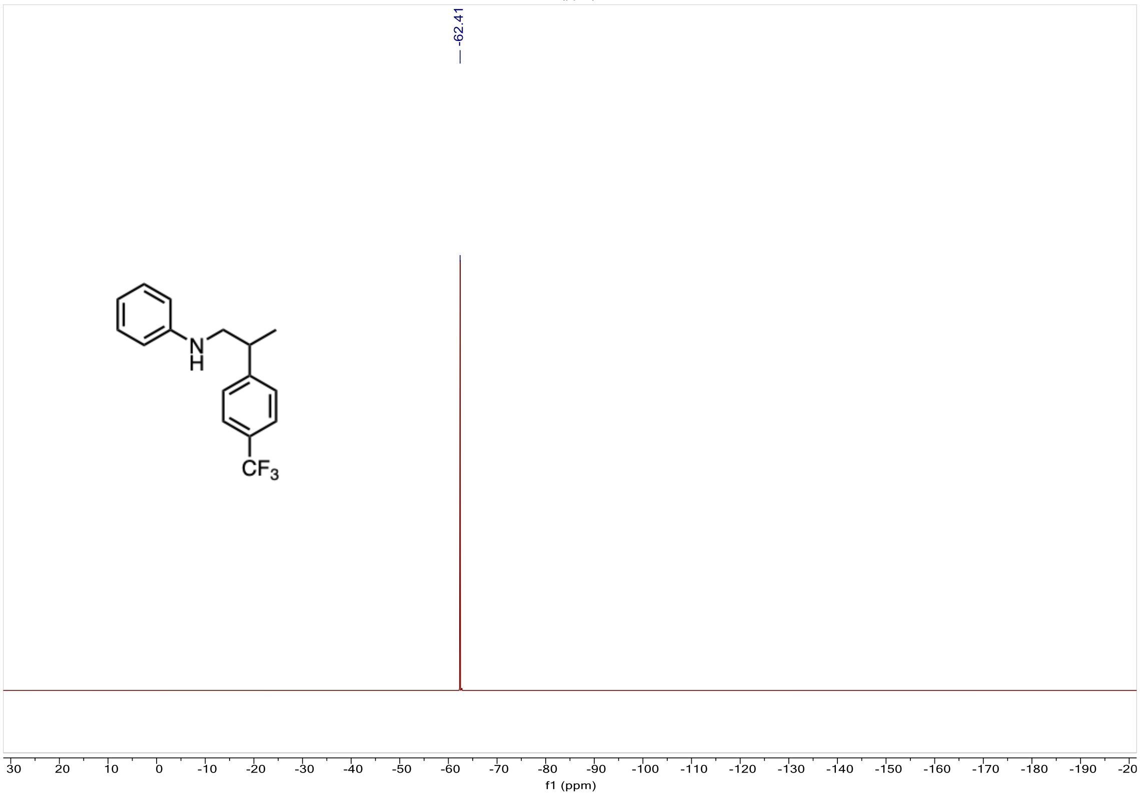
-62.41
N
H
CF3
30 20 10 0 -10 -20 -30 -40 -50 -60 -70 -80 -90 -100 -110 -120 -130 -140 -150 -160 -170 -180 -190 -20
f1 (ppm)

N-(2-(4-(trifluoromethyl)phenyl)propyl)aniline (2q)

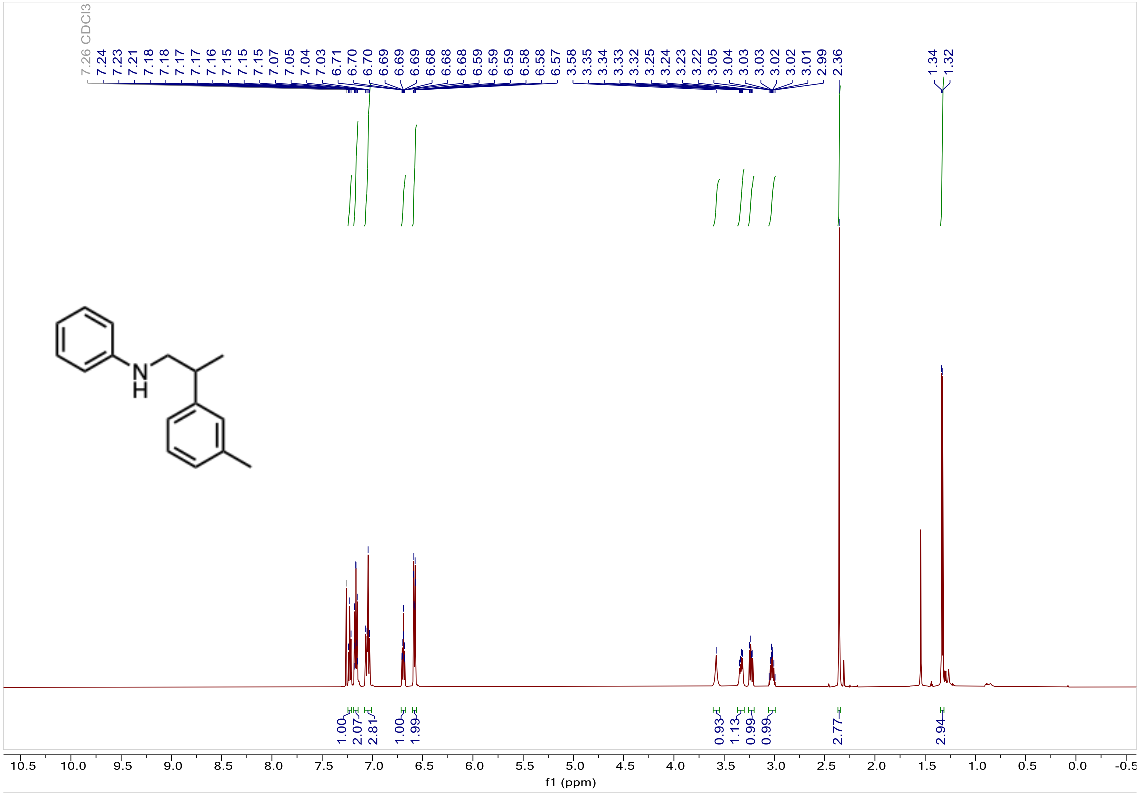

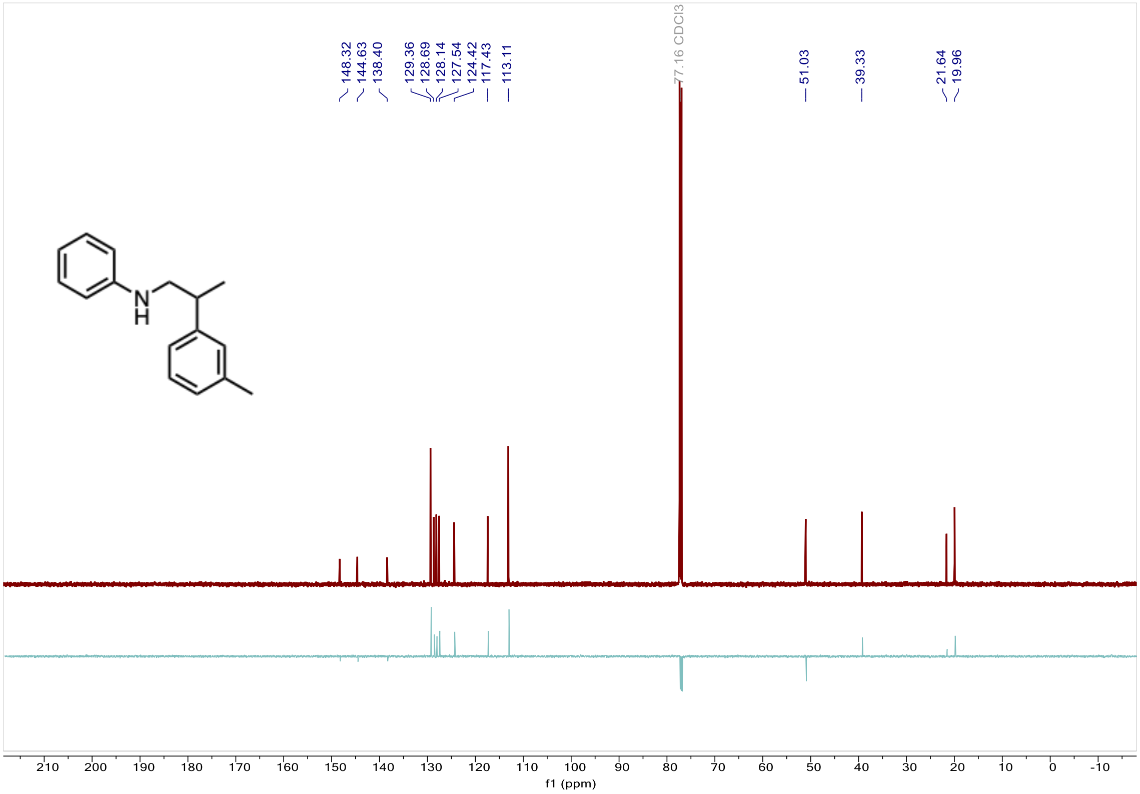

N-(2-(naphthalen-1-yl)propyl)aniline (2r)

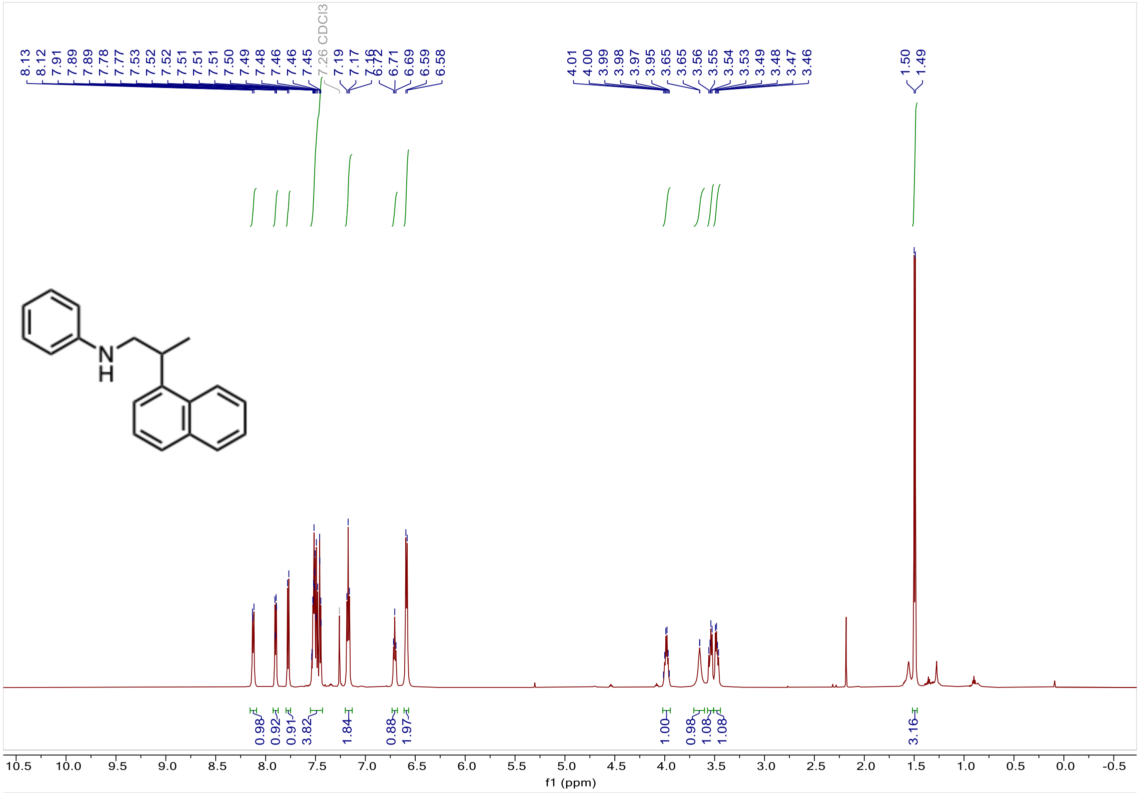

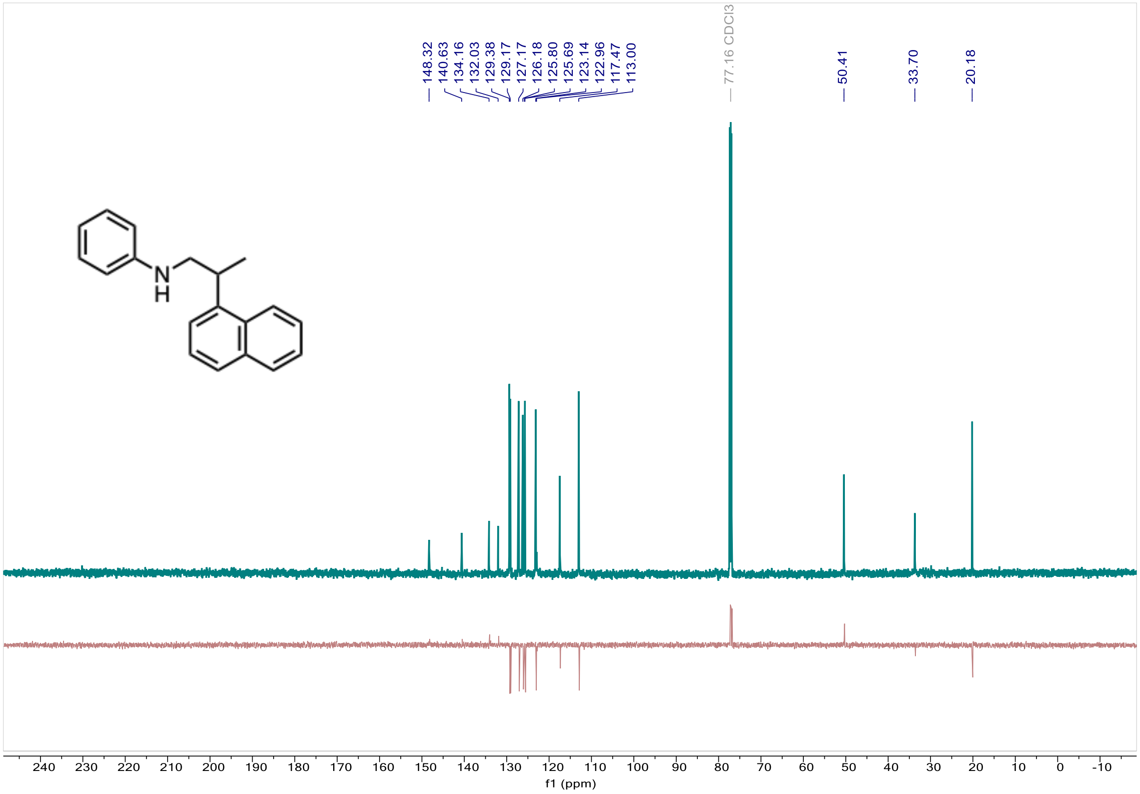

N-(2-(isoquinolin-5-yl)propyl)aniline (2s)

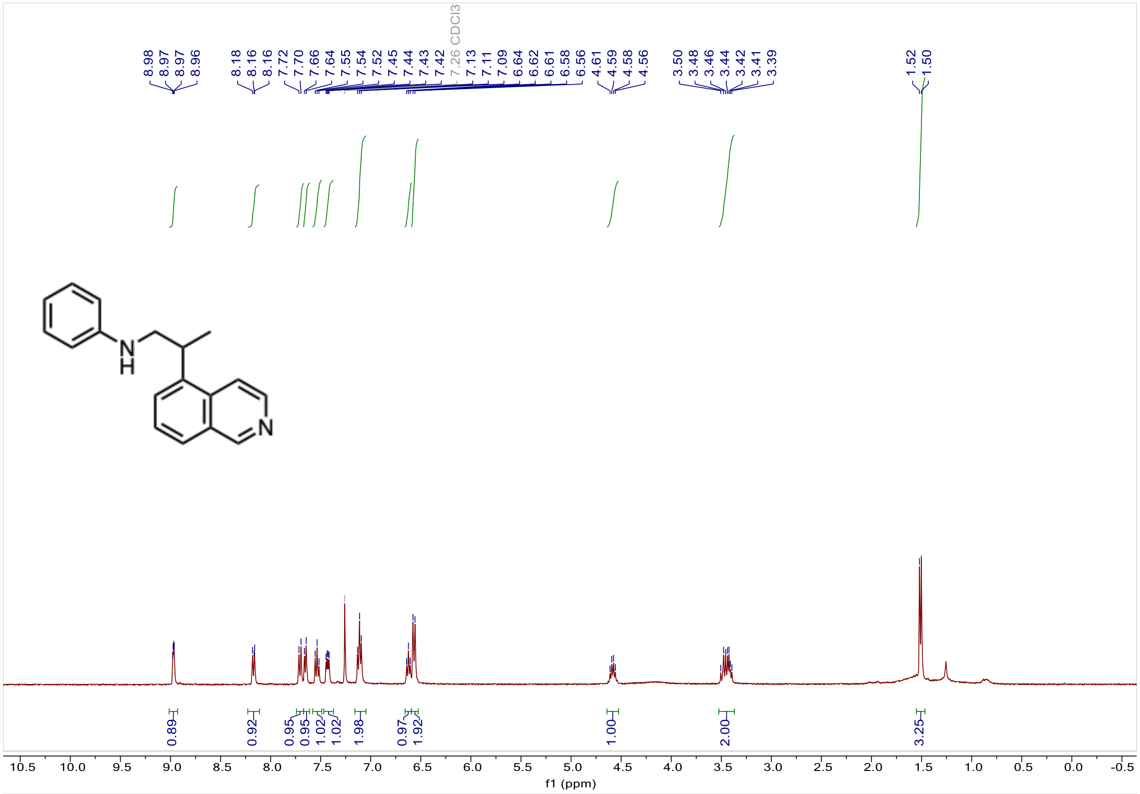

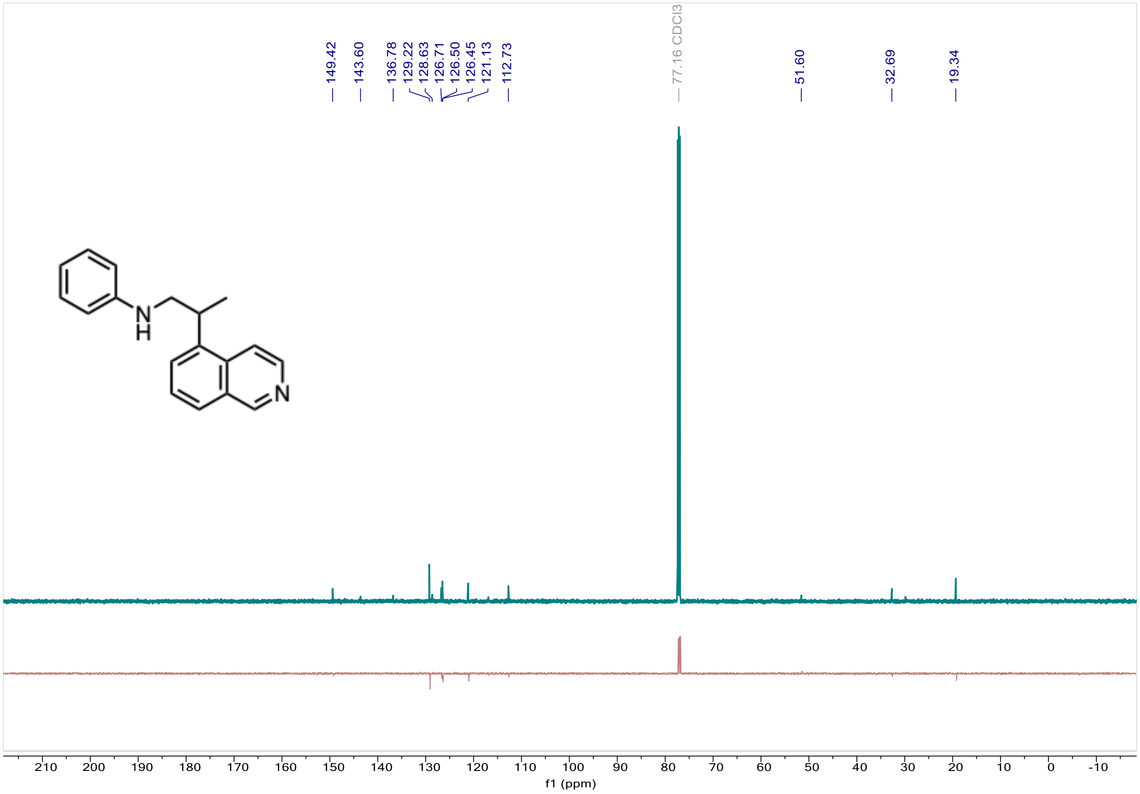

N-(2-mesitylpropyl)-4-methylaniline (2t)

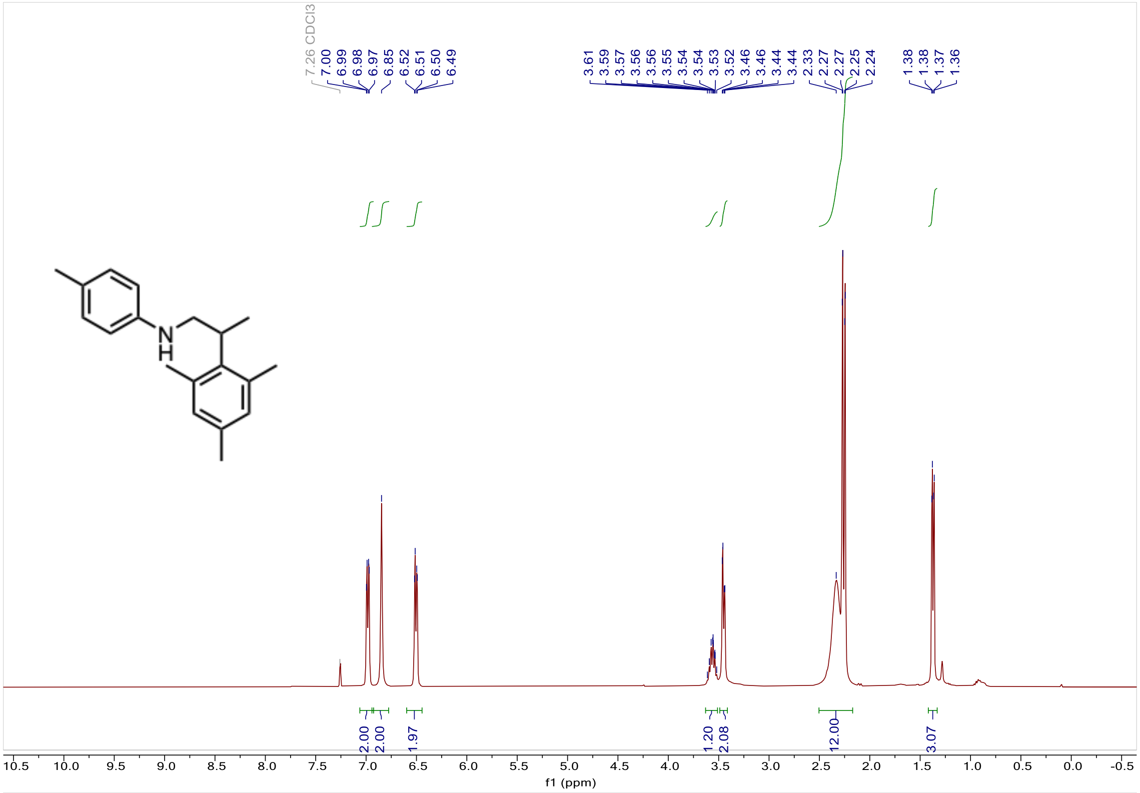

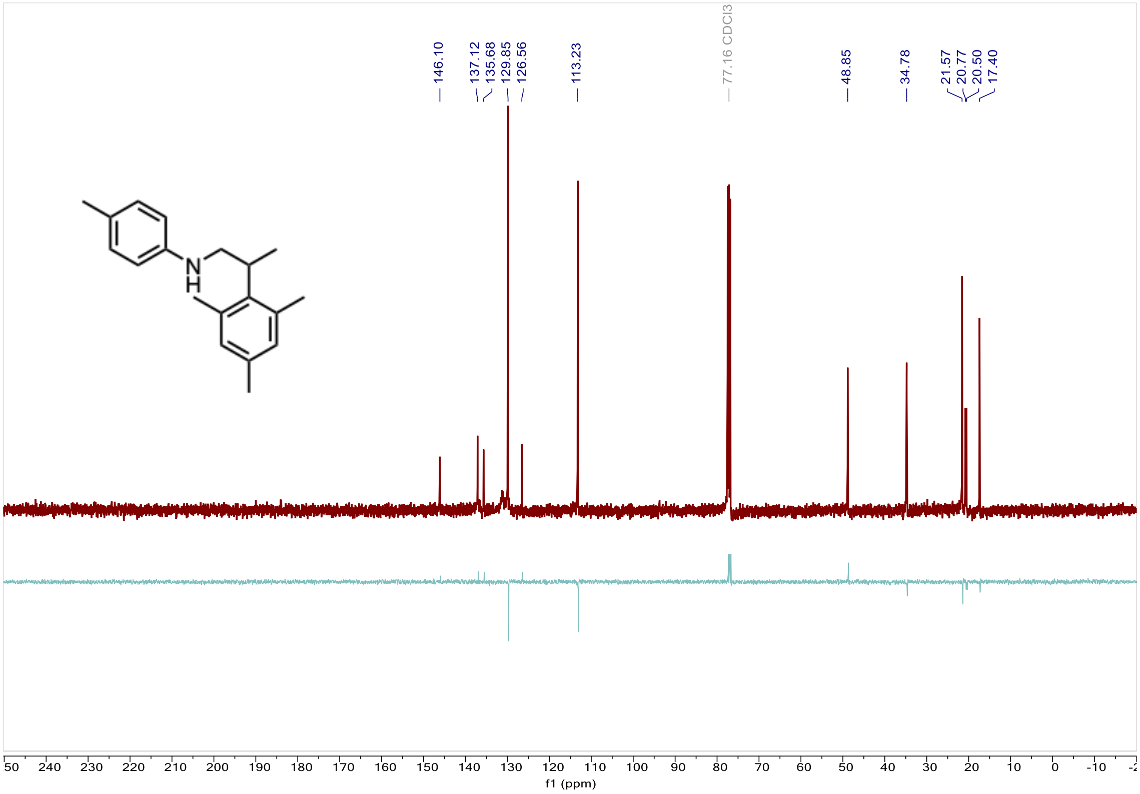

N-(2-mesitylpropyl)-4-methoxyaniline (2u)

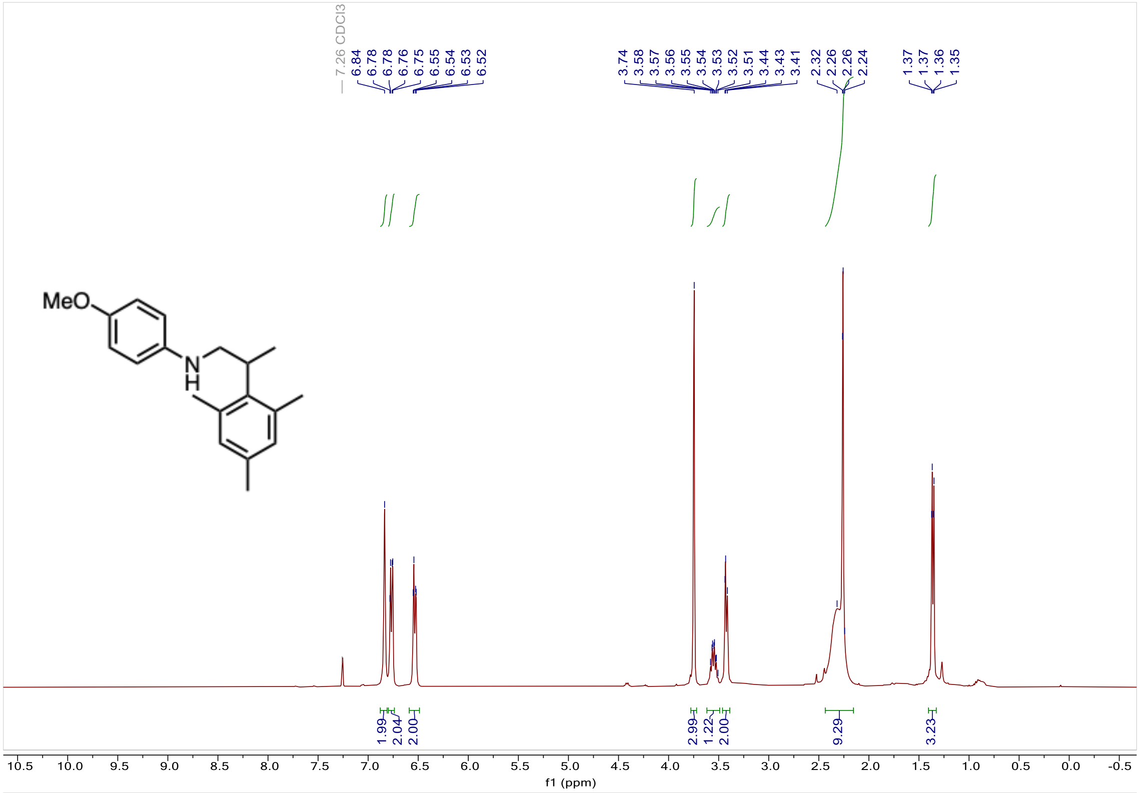

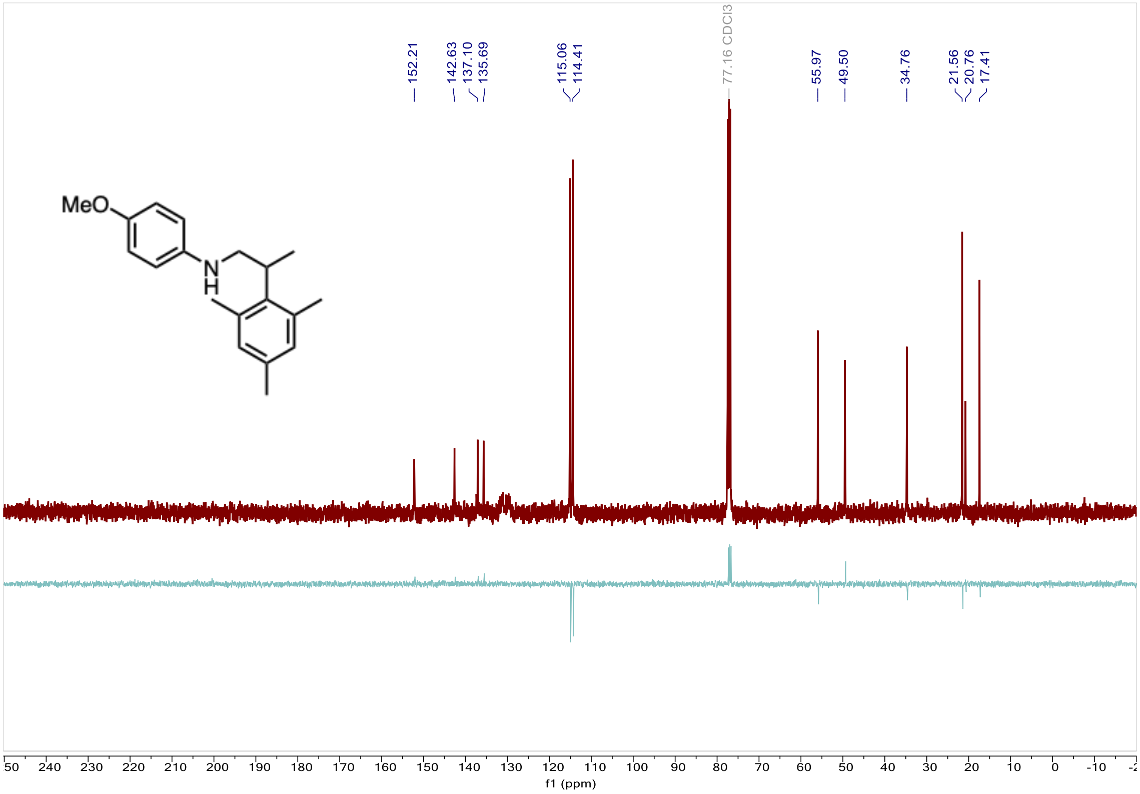

4-chloro-*N*-(2-mesitylpropyl)aniline (2v)

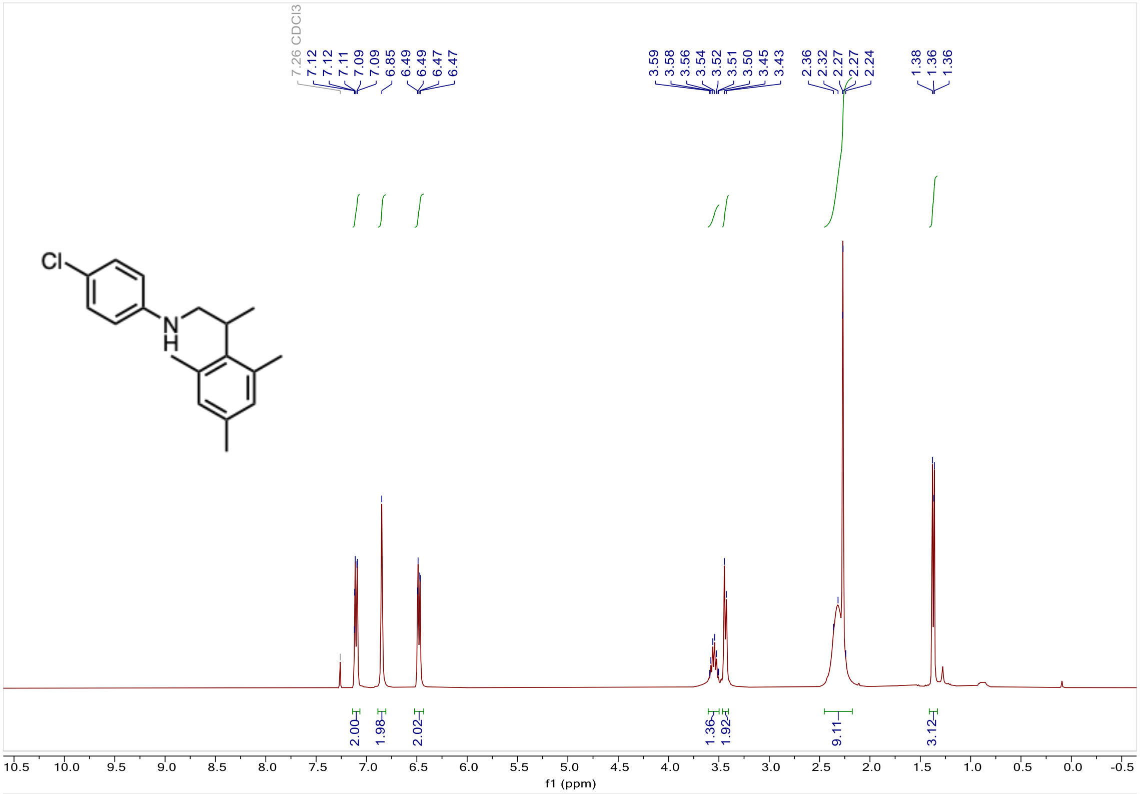

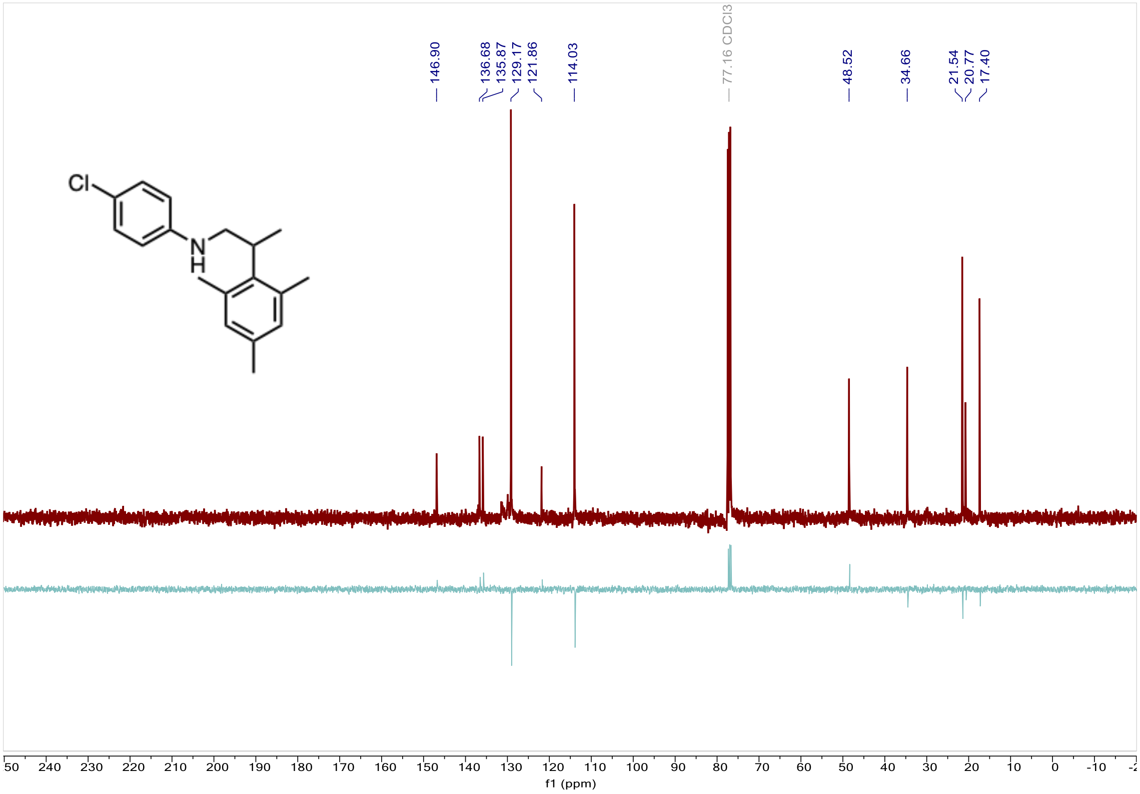

4-bromo-*N*-(2-mesitylpropyl)aniline (2w)

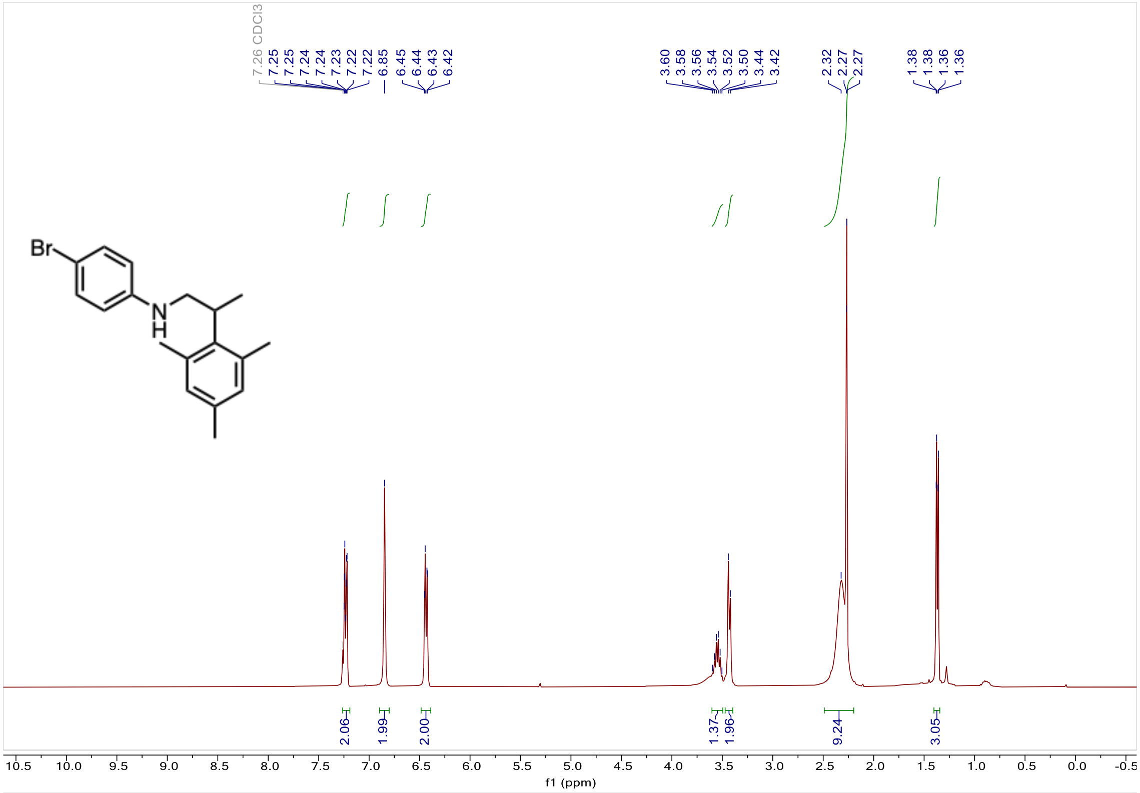

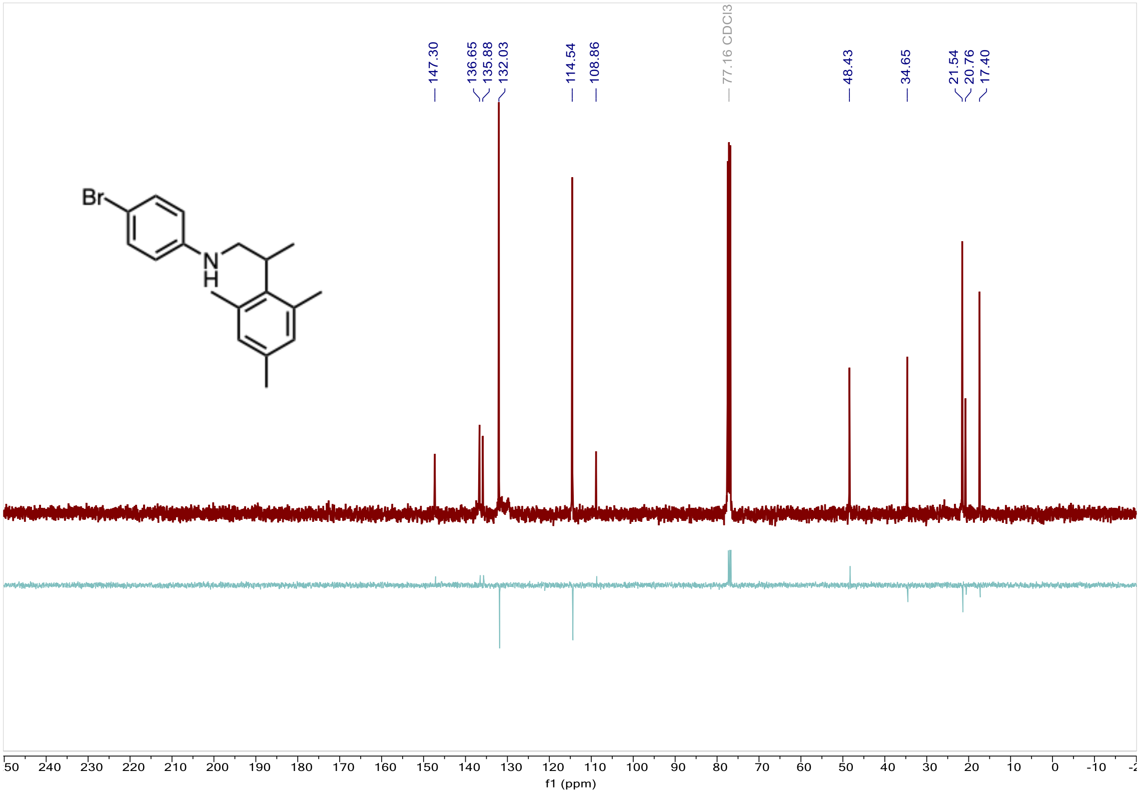

4-fluoro-*N*-(2-mesitylpropyl)aniline (2x)

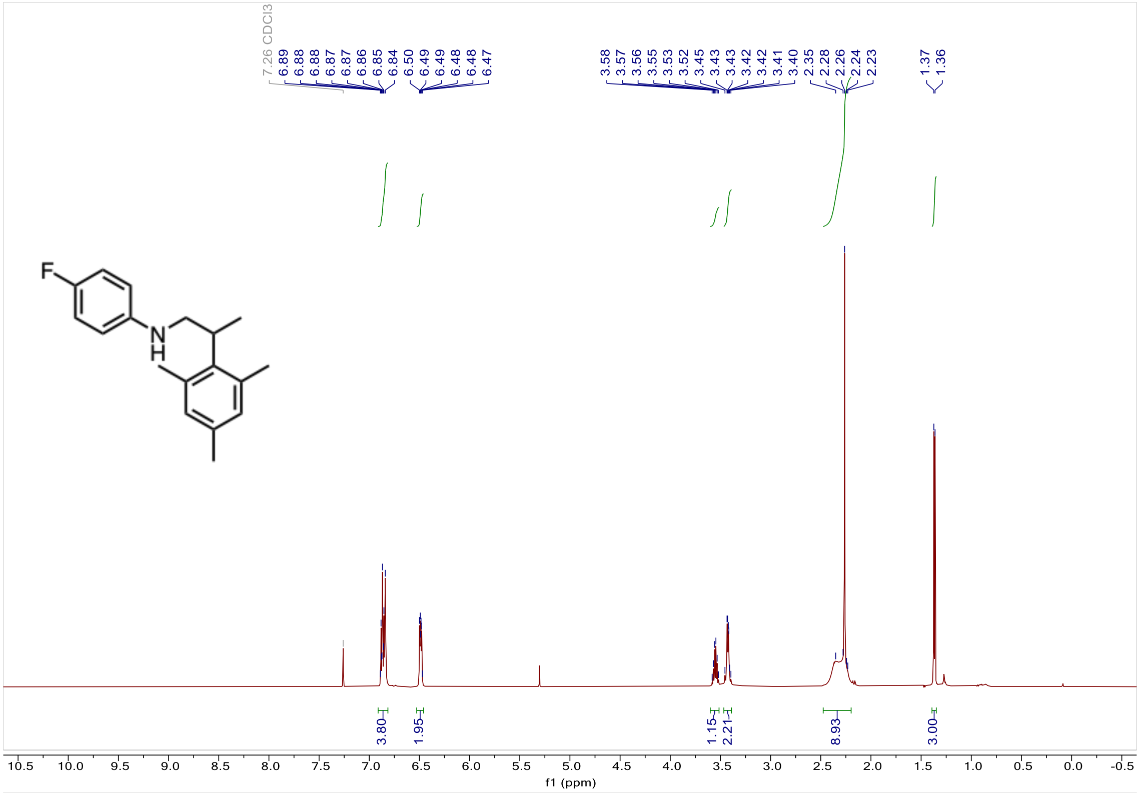

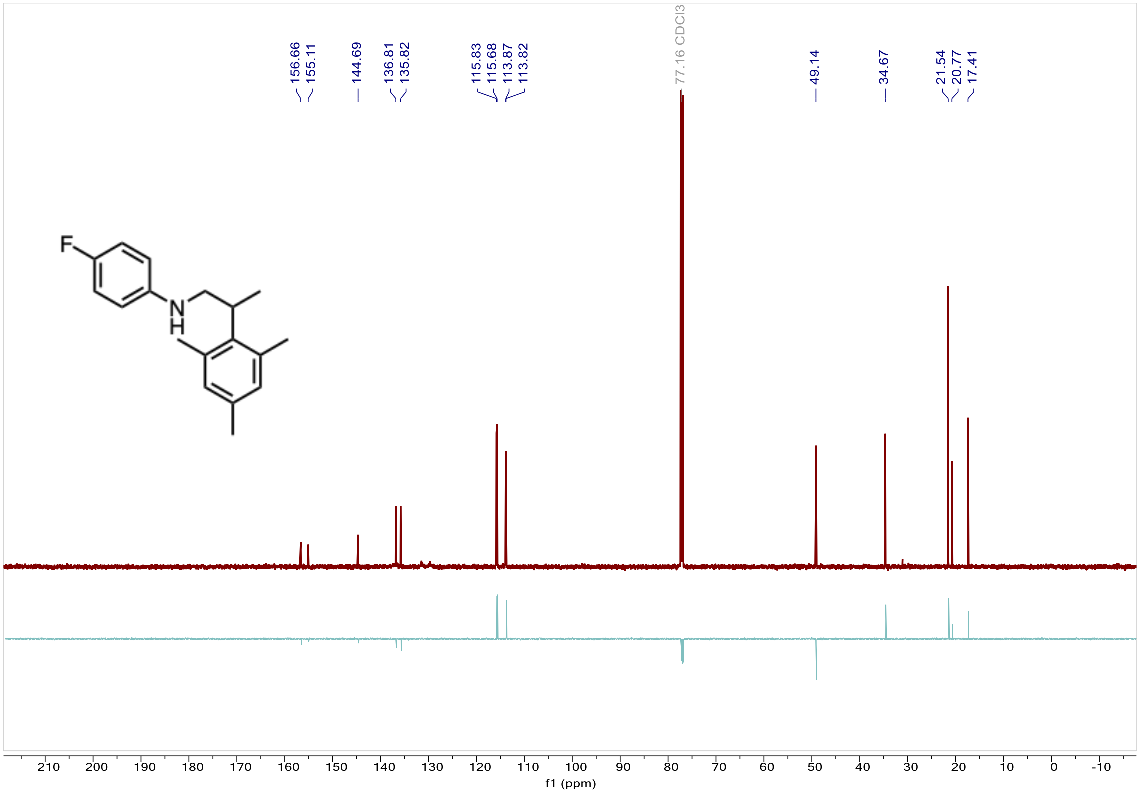

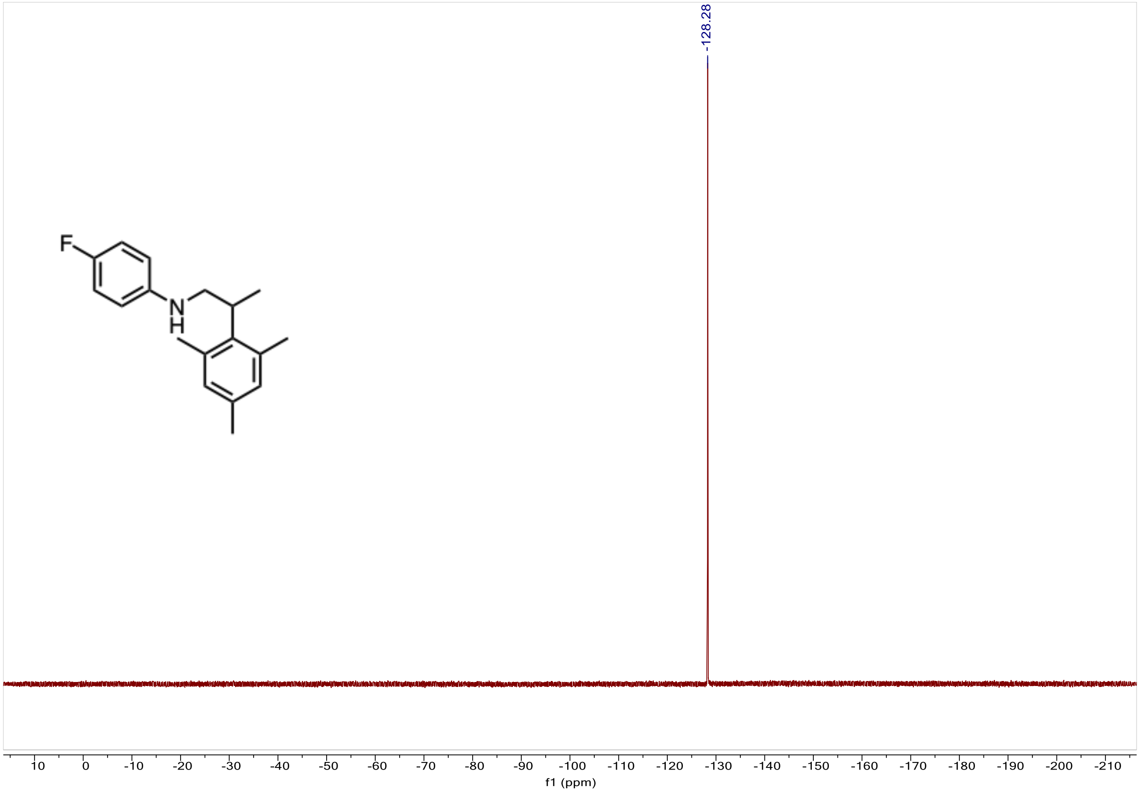

N-(2-mesitylpropyl)-4-(trifluoromethyl)aniline (2y)

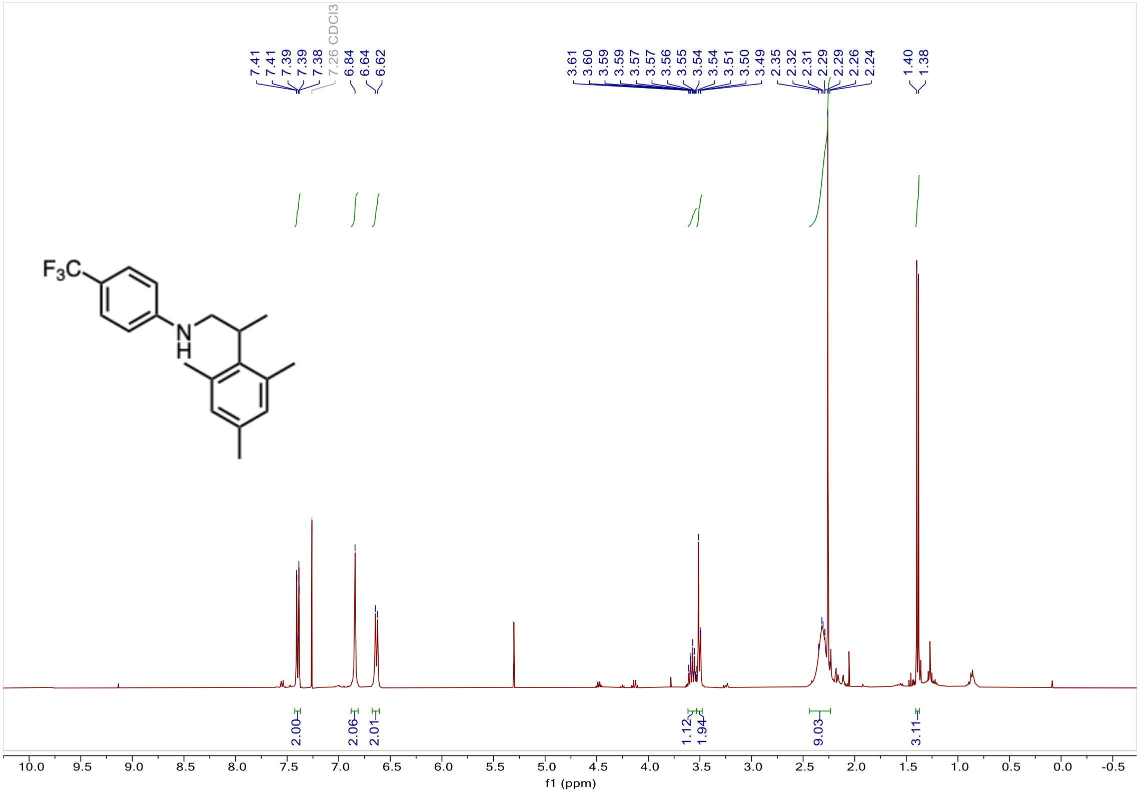

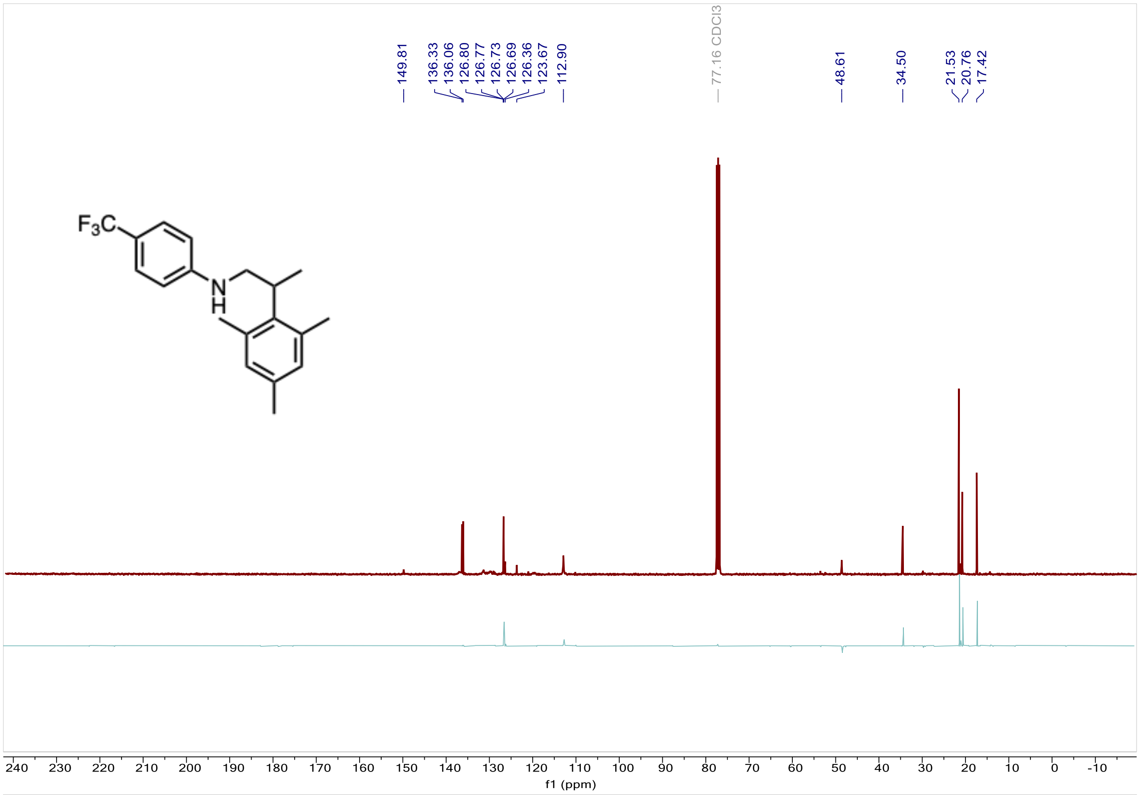
149.81
136.33
136.06
126.80
126.77
126.73
126.69
126.36
123.67
112.90
77.16 CDCl3
48.61
34.50
21.53
20.76
17.42
F3C
N
H
240 230 220 210 200 190 180 170 160 150 140 130 120 110 100 90 80 70 60 50 40 30 20 10 0 -10
f1 (ppm)

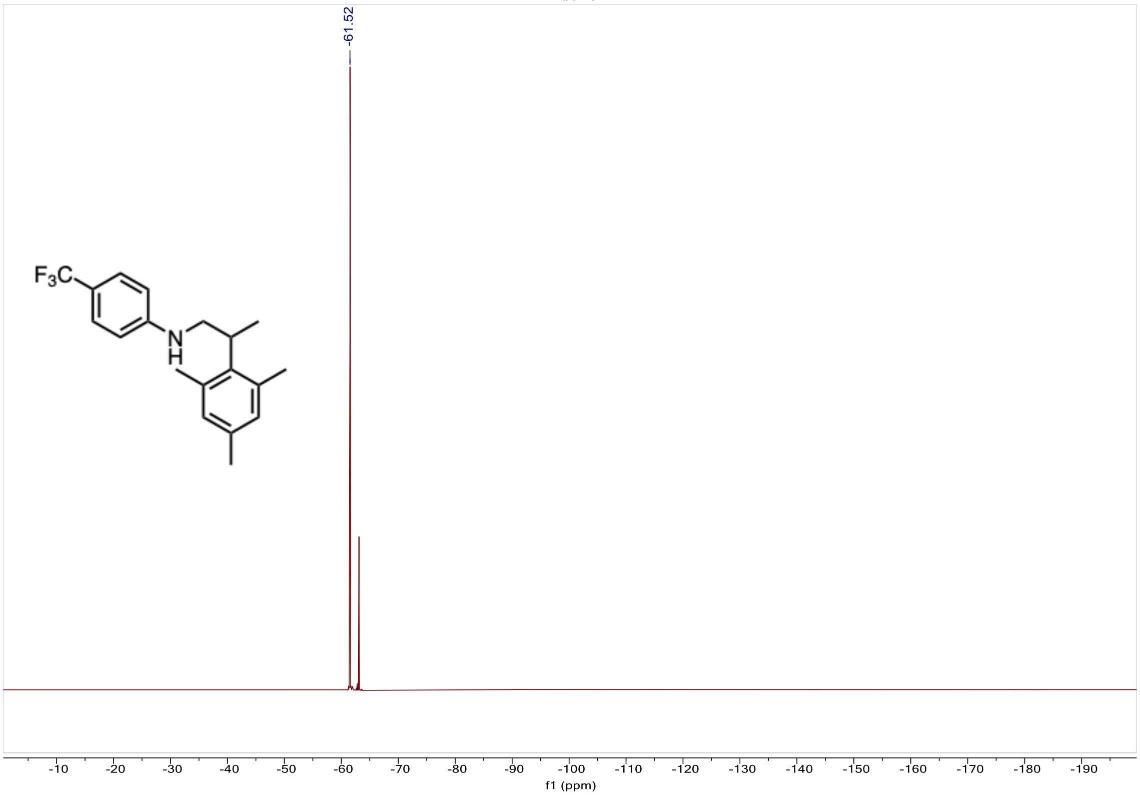
-61.52
F3C
N
H
-10 -20 -30 -40 -50 -60 -70 -80 -90 -100 -110 -120 -130 -140 -150 -160 -170 -180 -190
f1 (ppm)

N-(2-mesitylpropyl)-4-phenoxyaniline (2z)

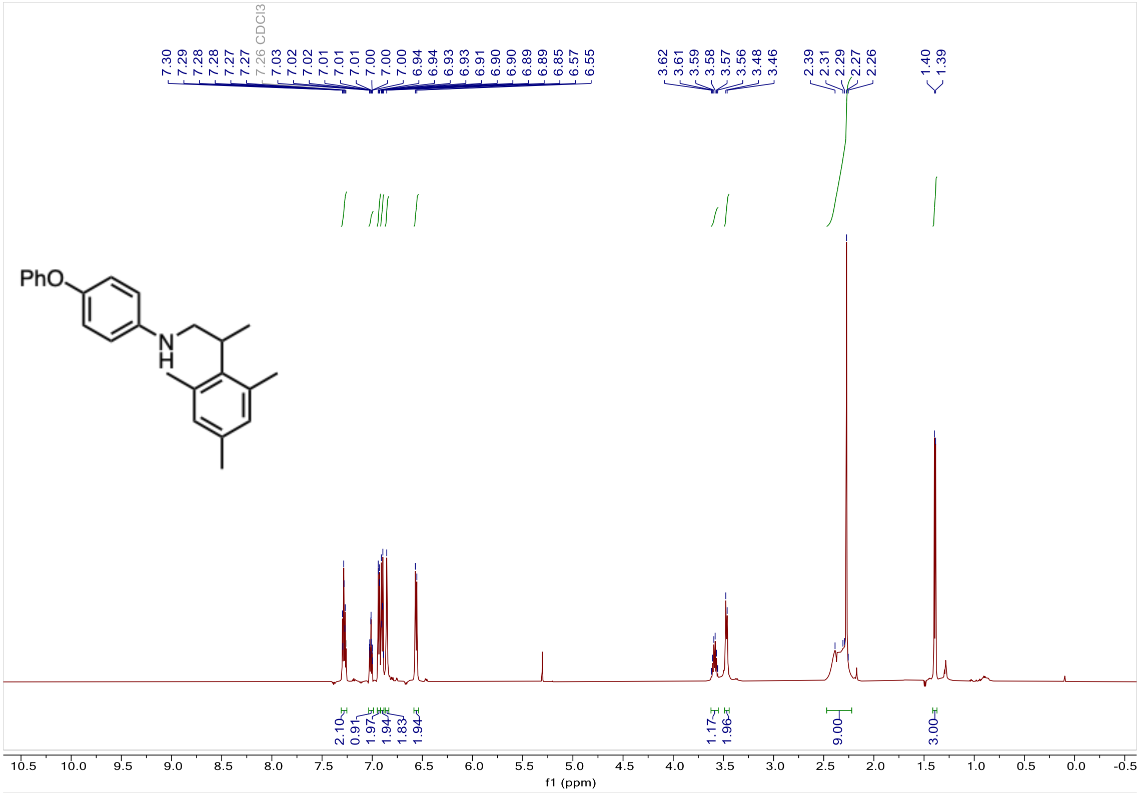

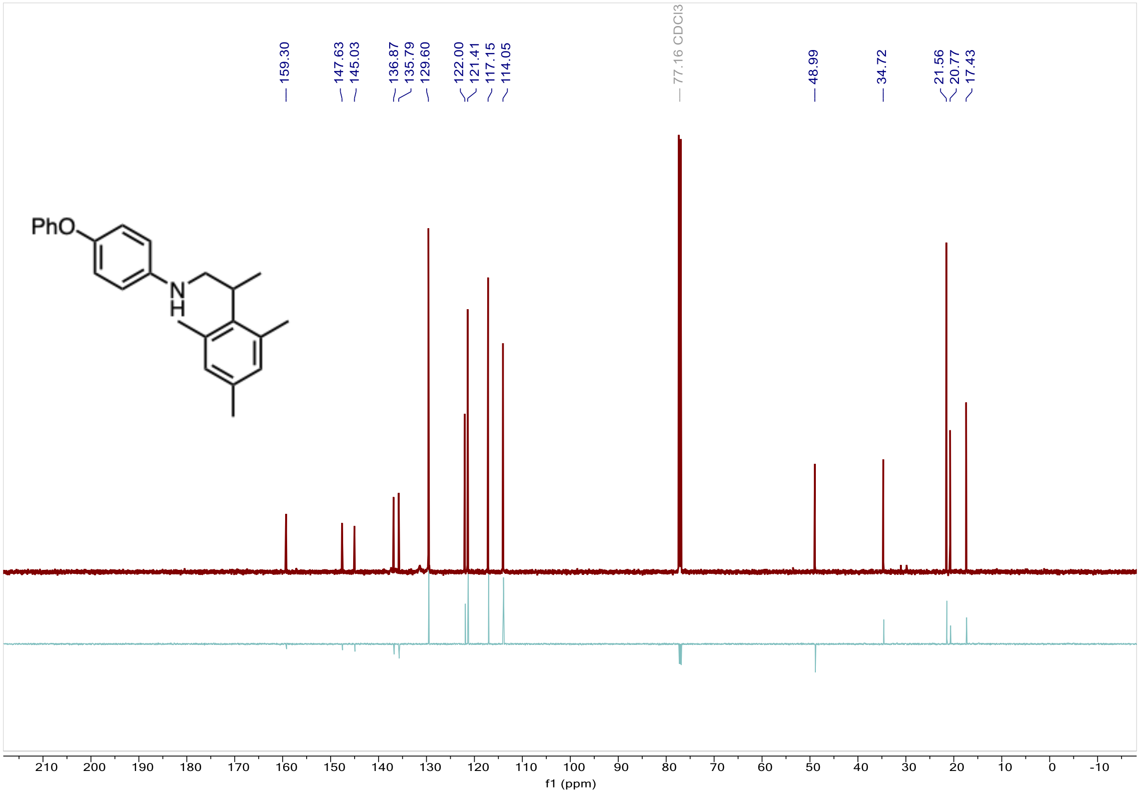

_N_-(2-mesitylpropyl)-3-methylaniline (2aa)

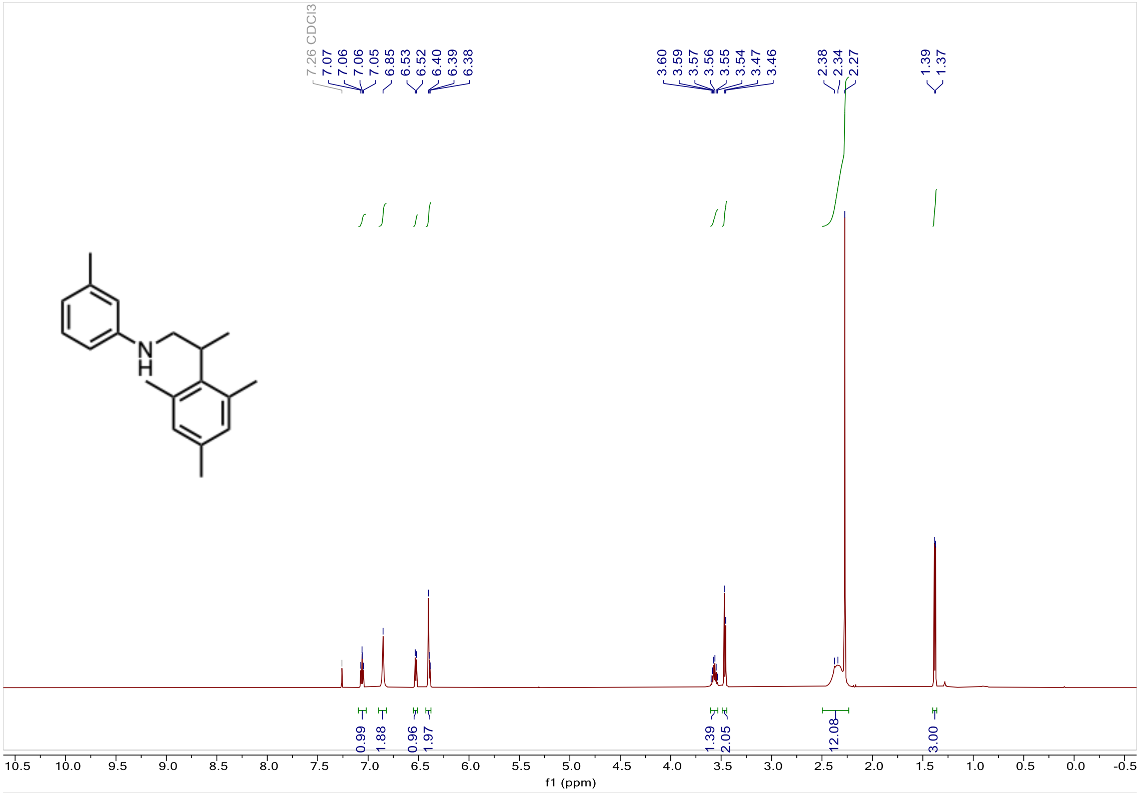

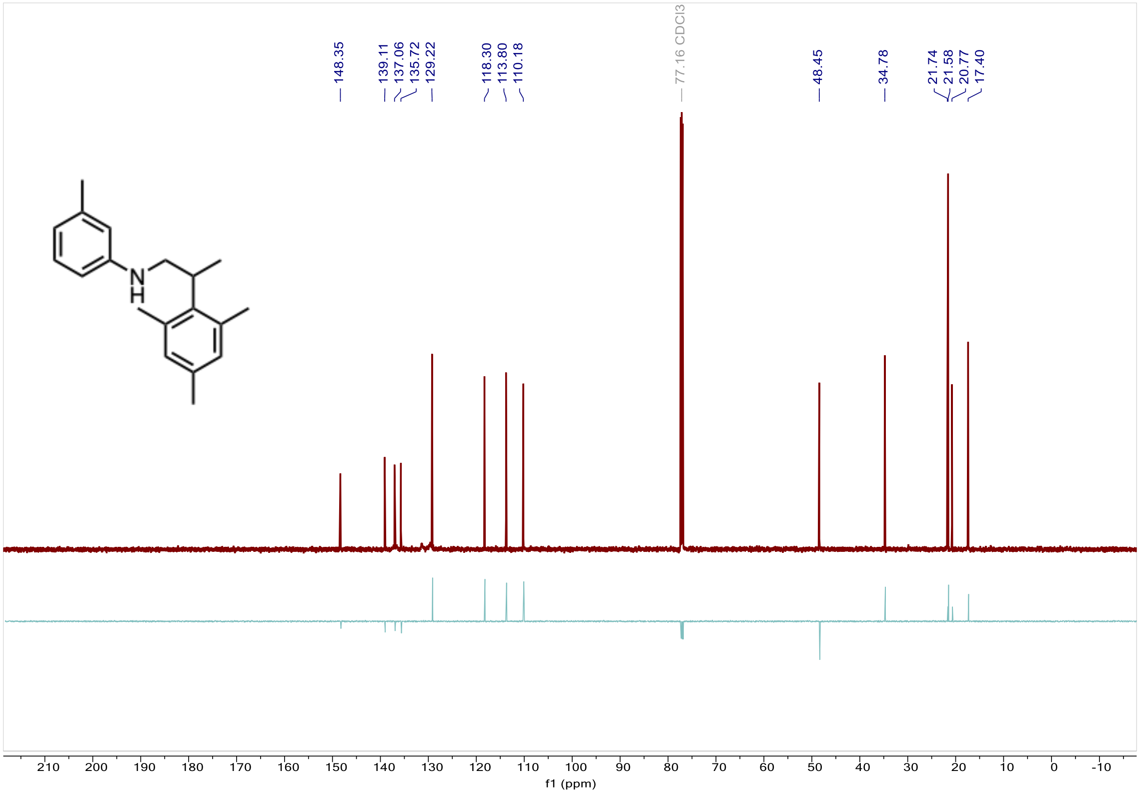

N-(2-mesitylpropyl)-3-methoxyaniline (2ab)

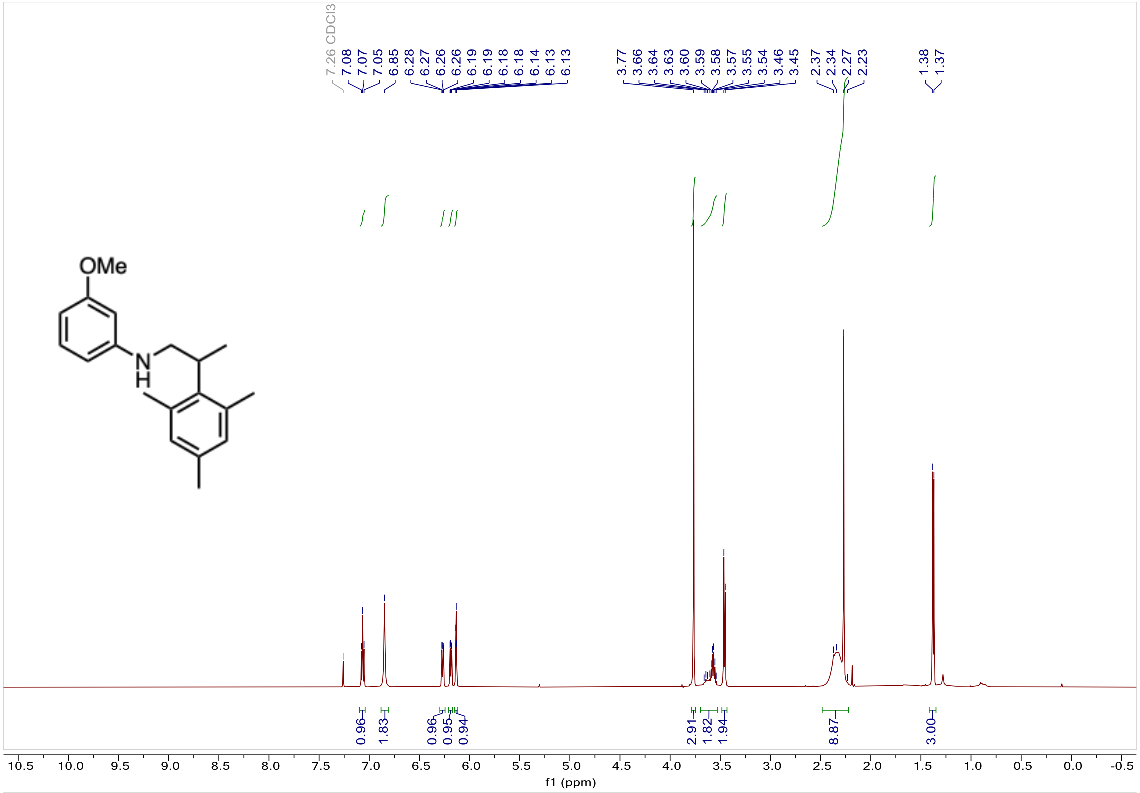

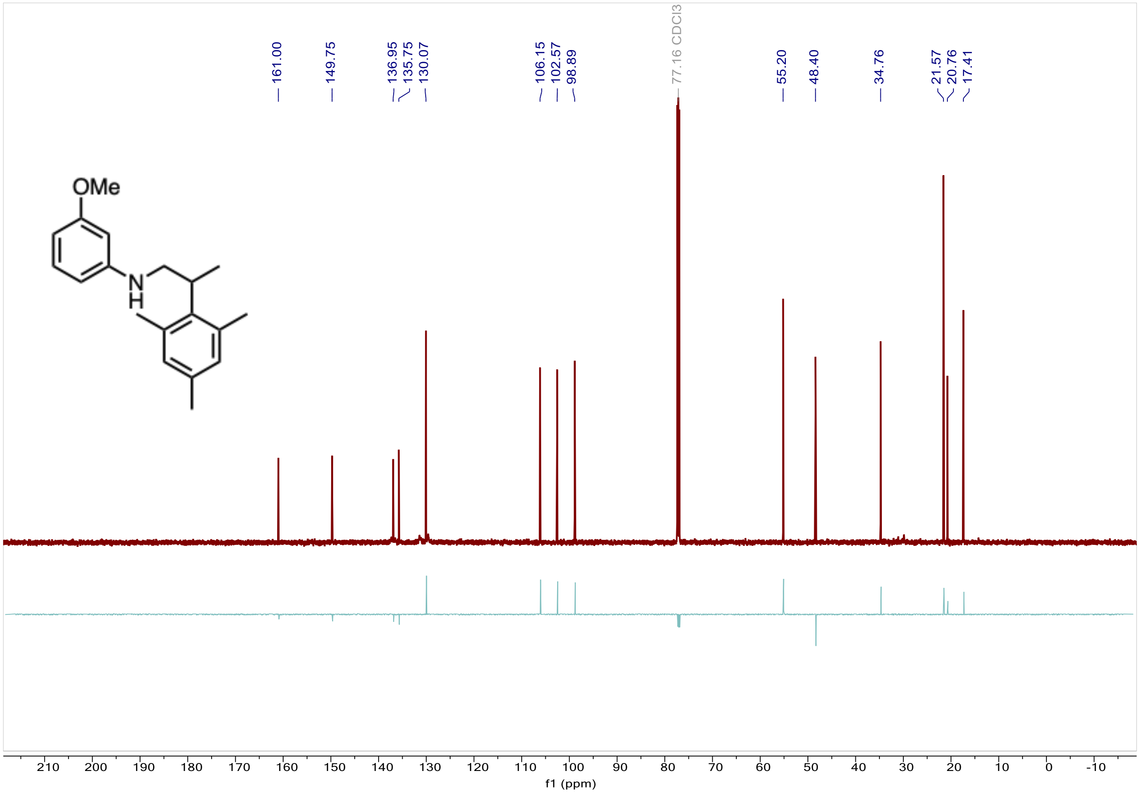

3-bromo-*N*-(2-mesitylpropyl)aniline (2ac)

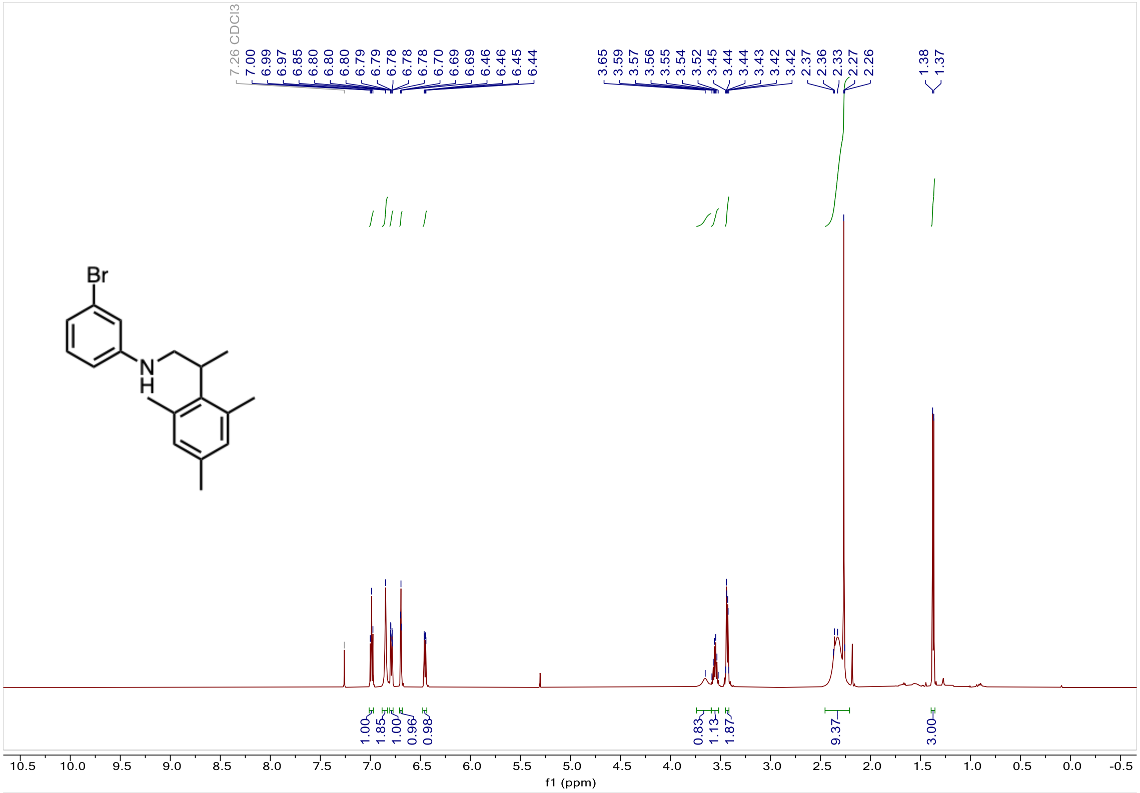

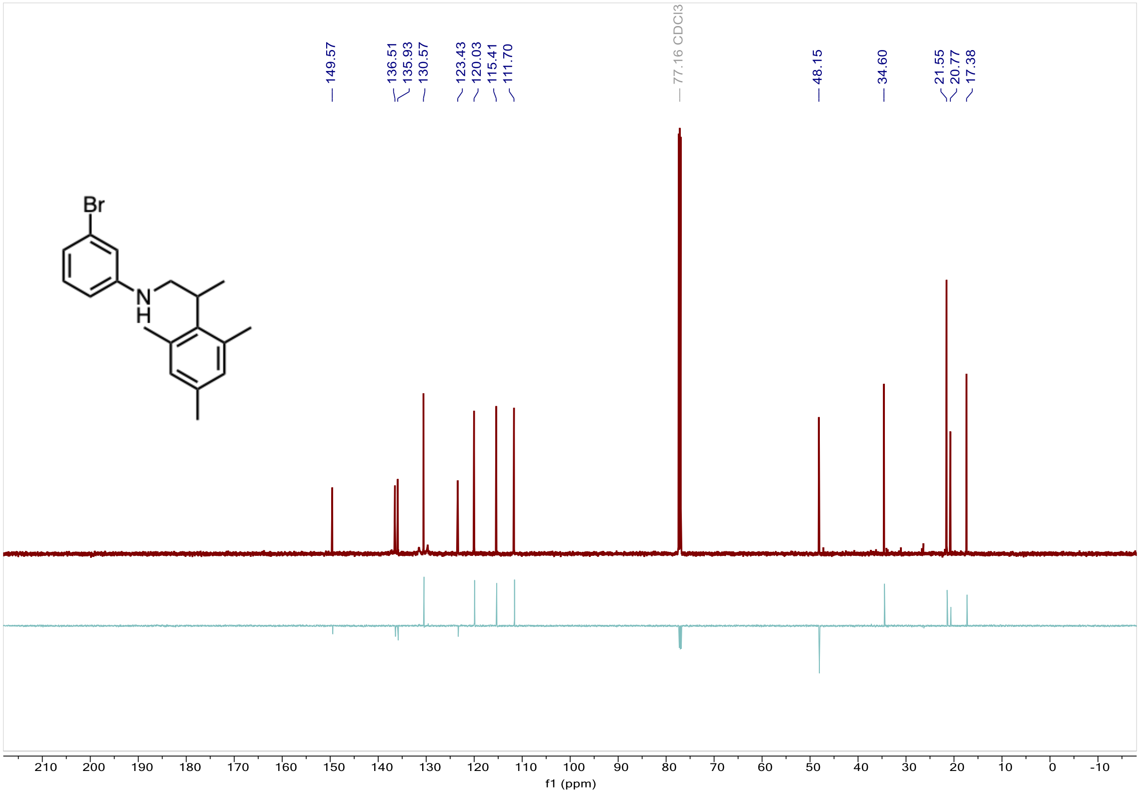

3-bromo-*N*-(2-mesitylpropyl)aniline (2ad)

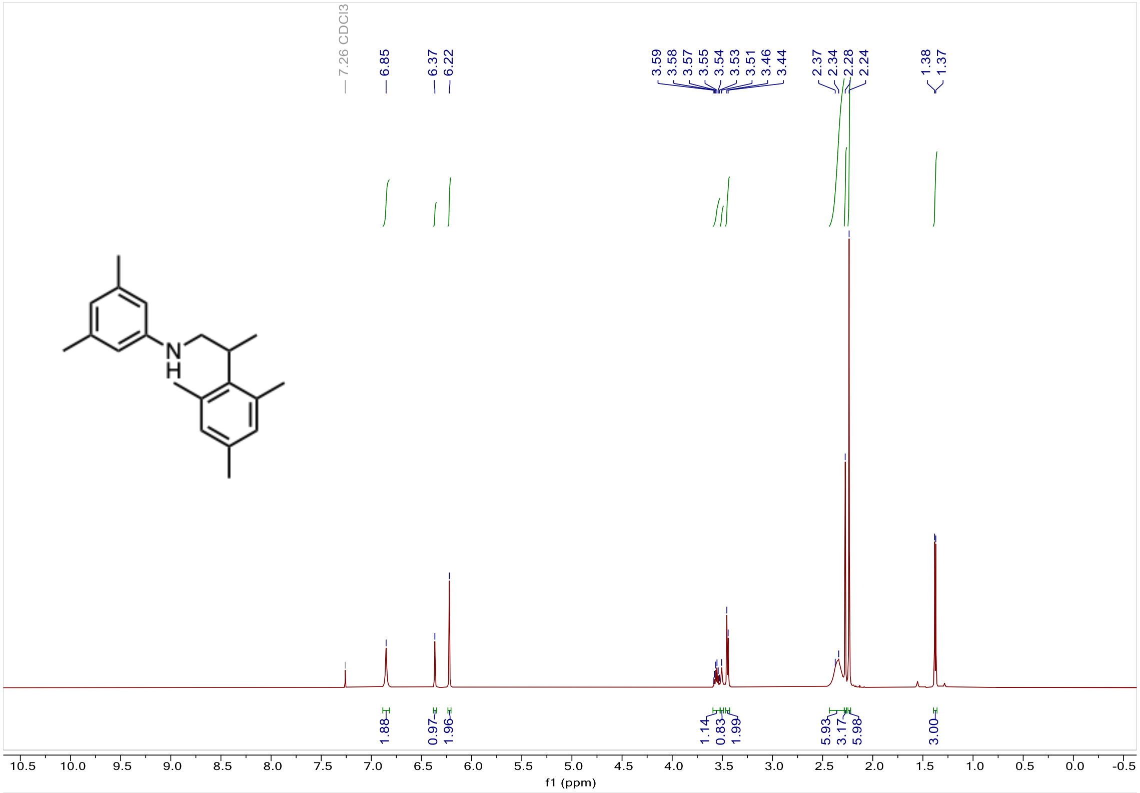

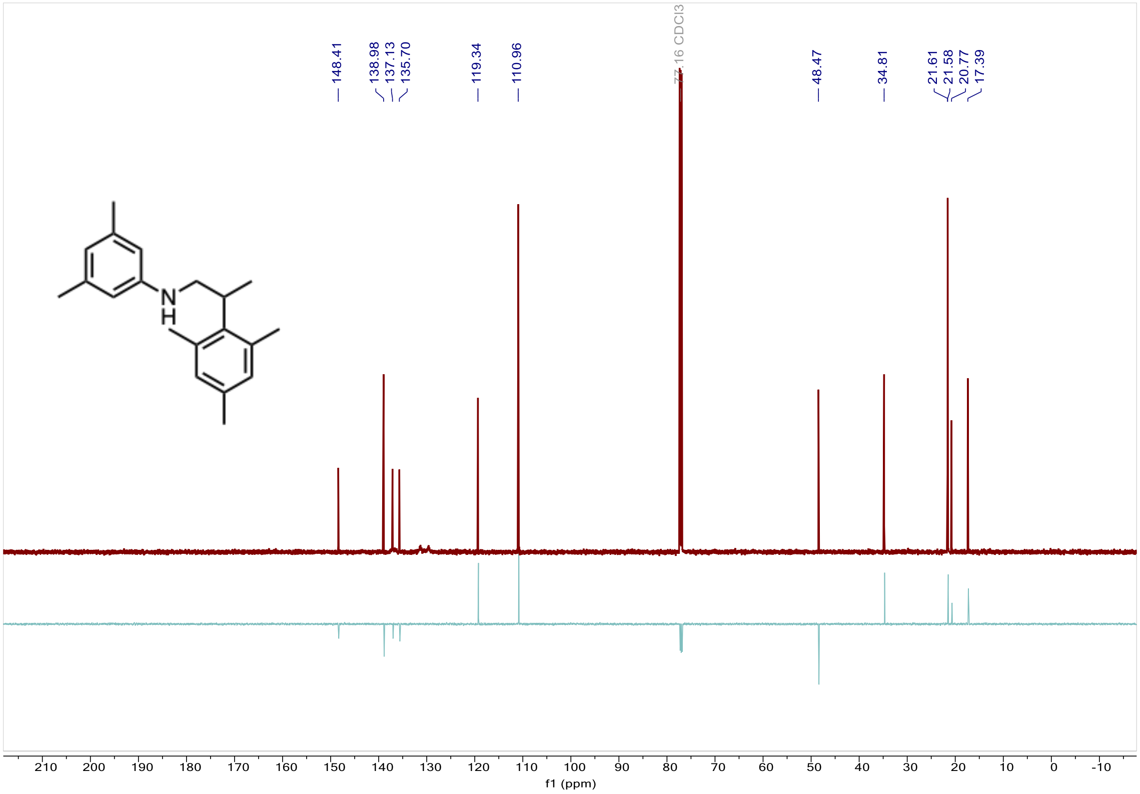

N-(2-mesitylpropyl)-2-methylaniline (2ae)

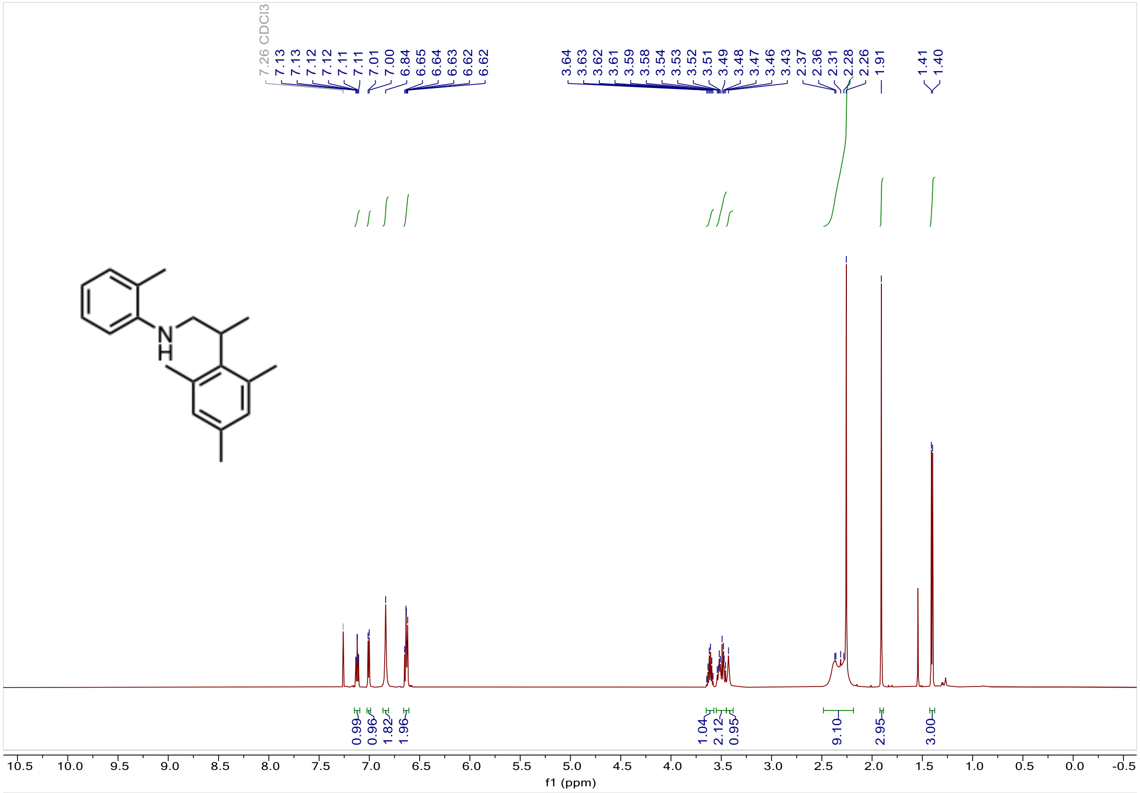

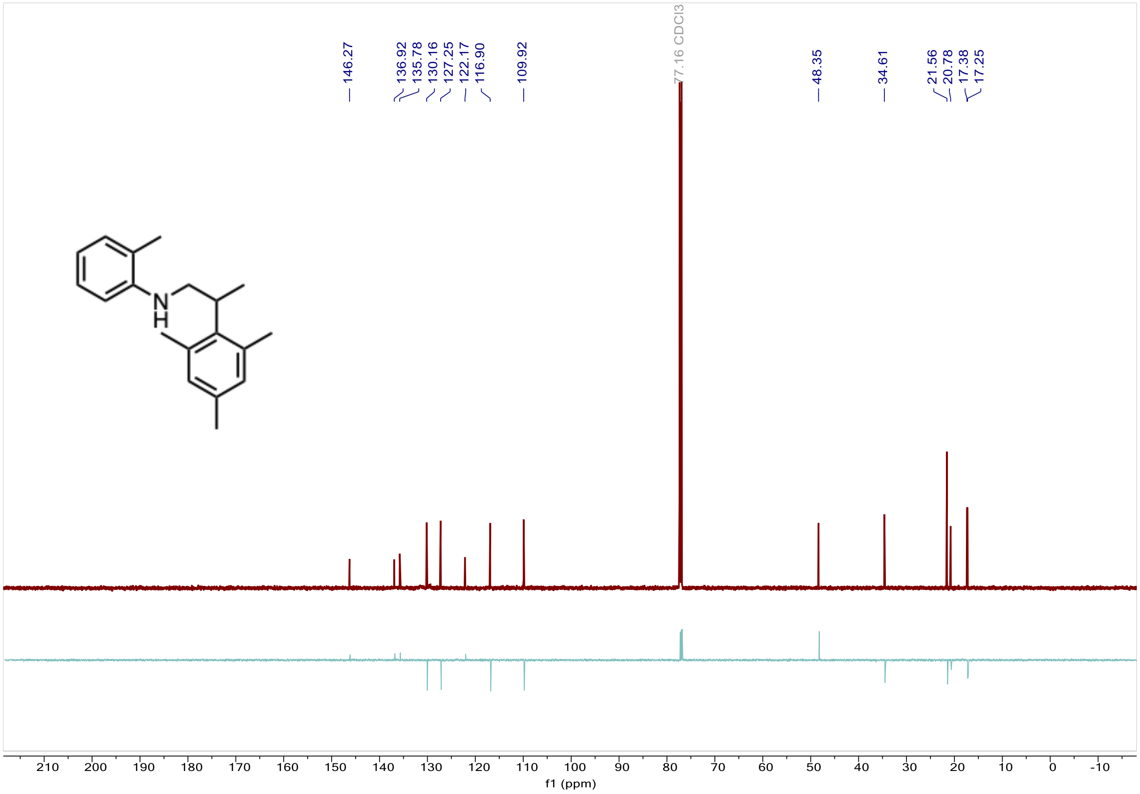

N-(2-mesitylpropyl)-2-methoxyaniline (2af)

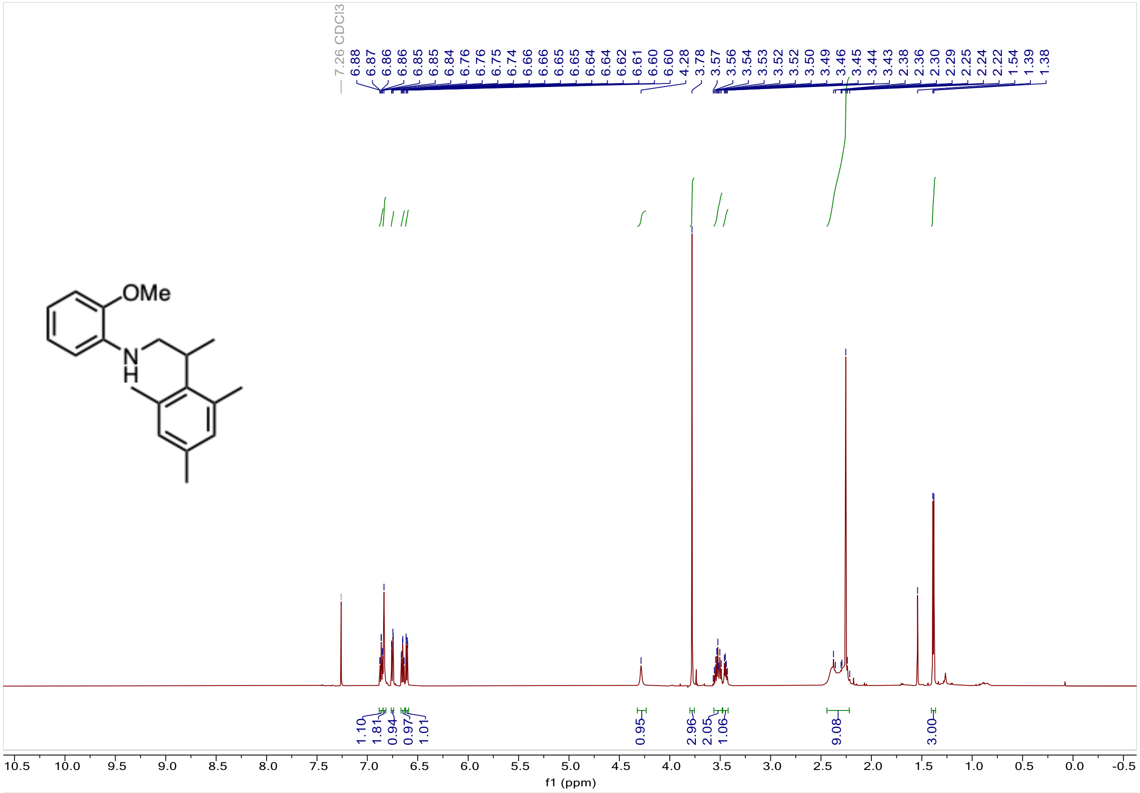

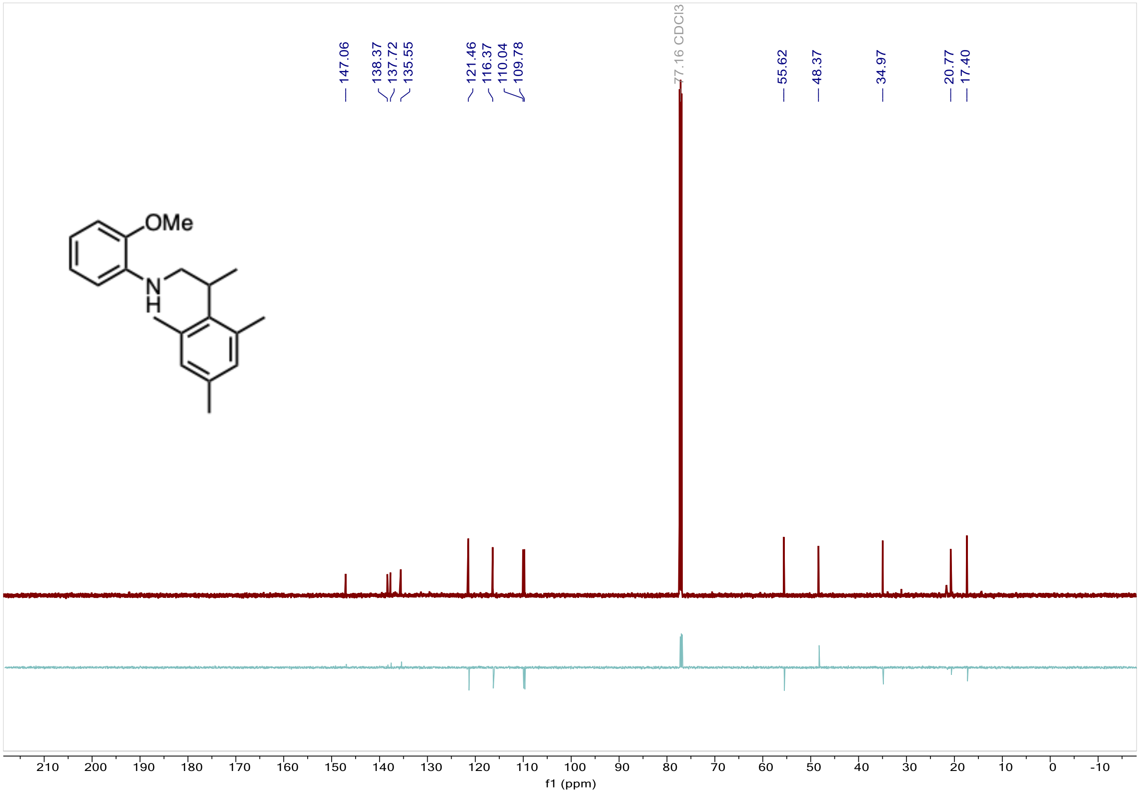

2-bromo-*N*-(2-mesitylpropyl)aniline (2ag)

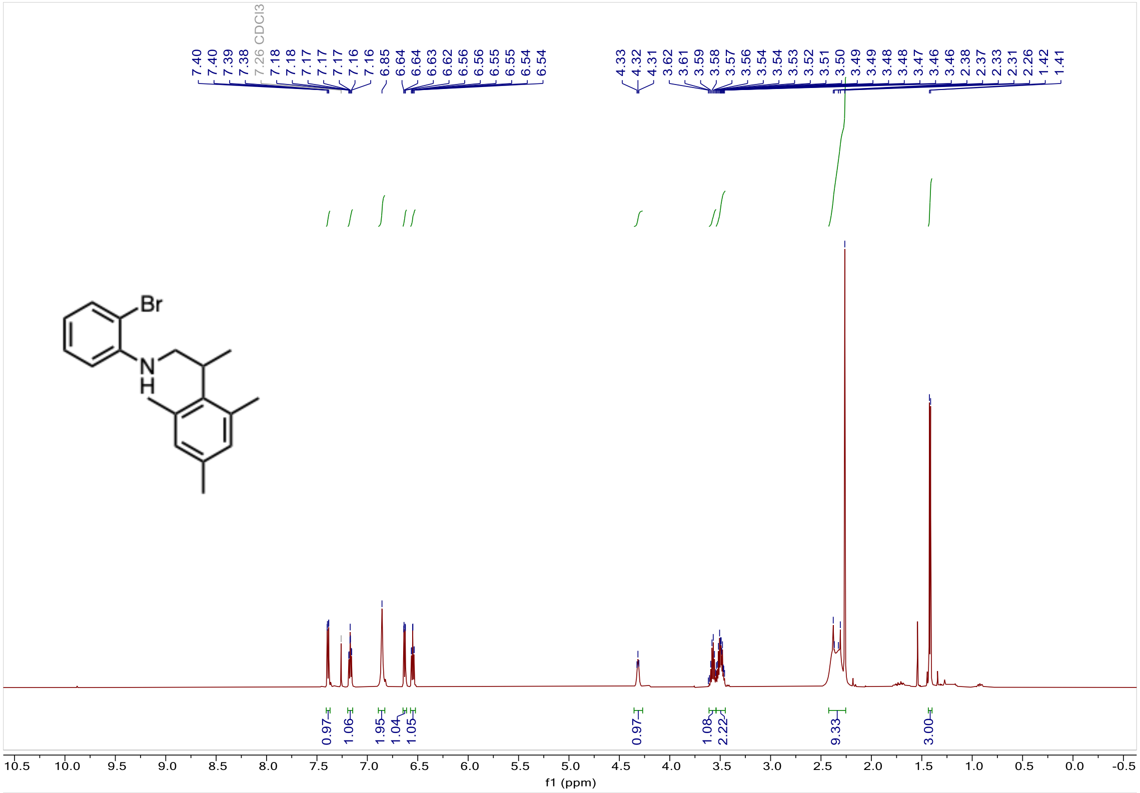

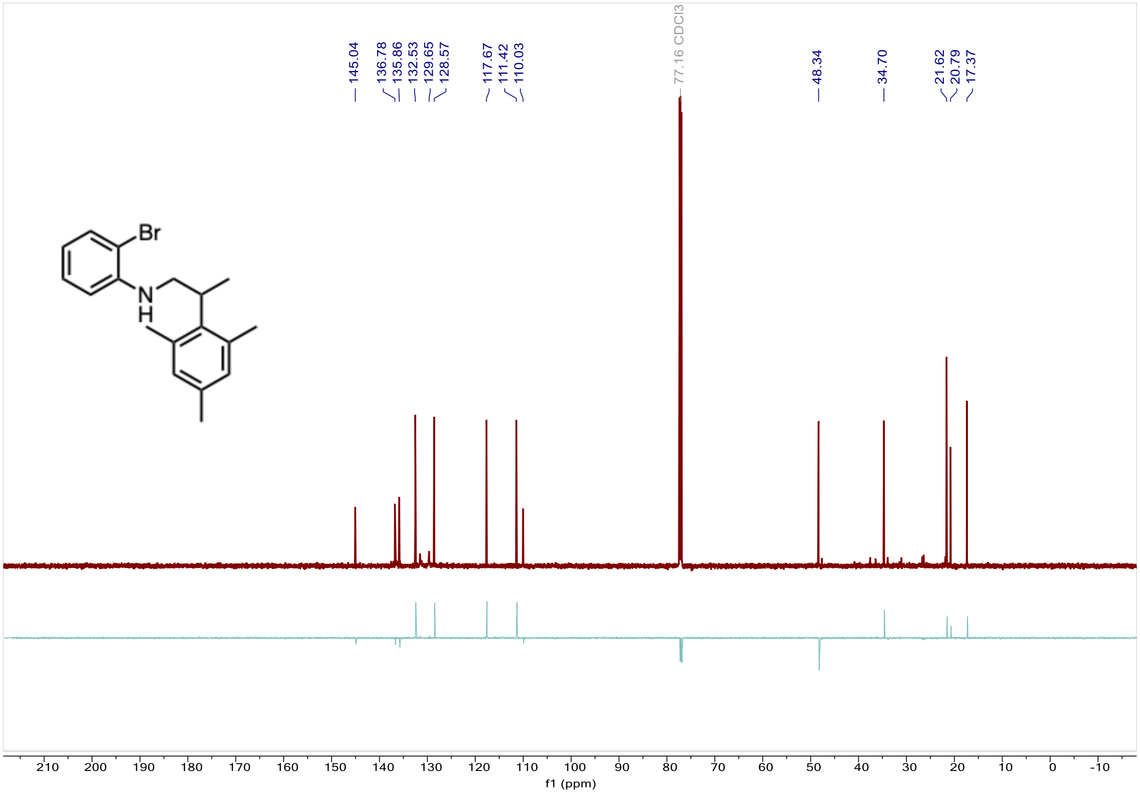

N-(2-mesitylpropyl)benzo[*d*][1,3]dioxol-5-amine (2ah)

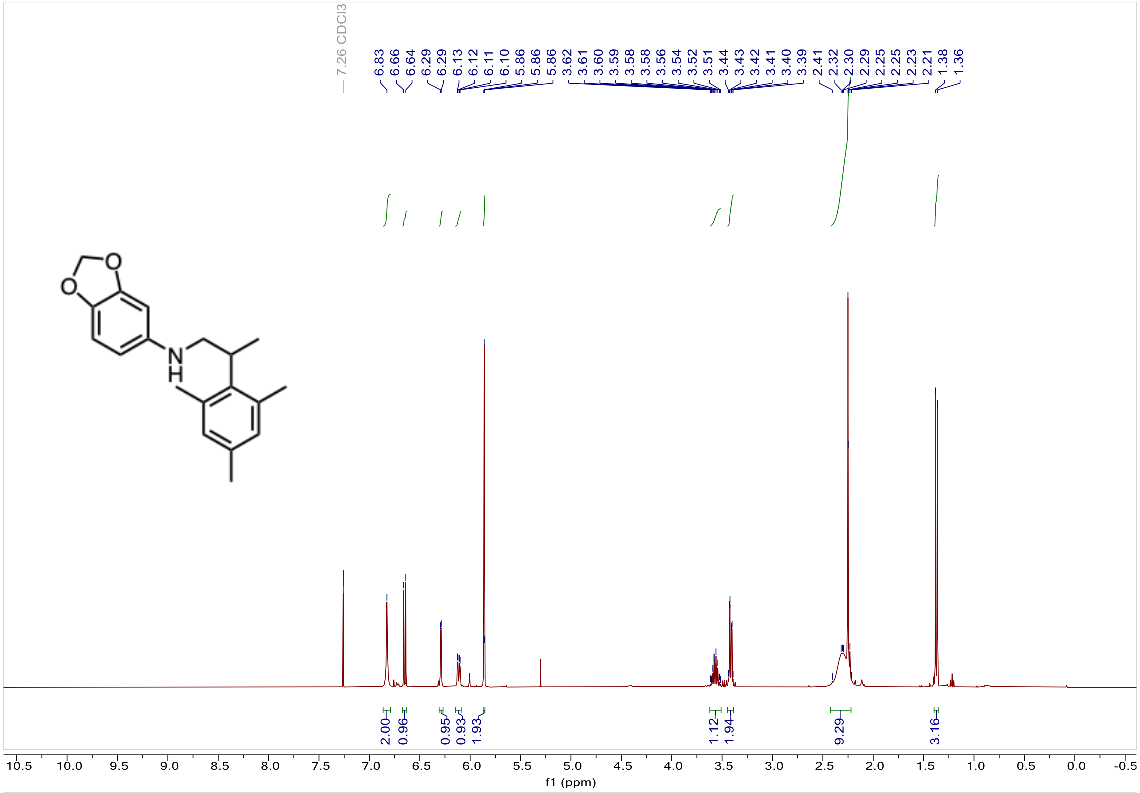

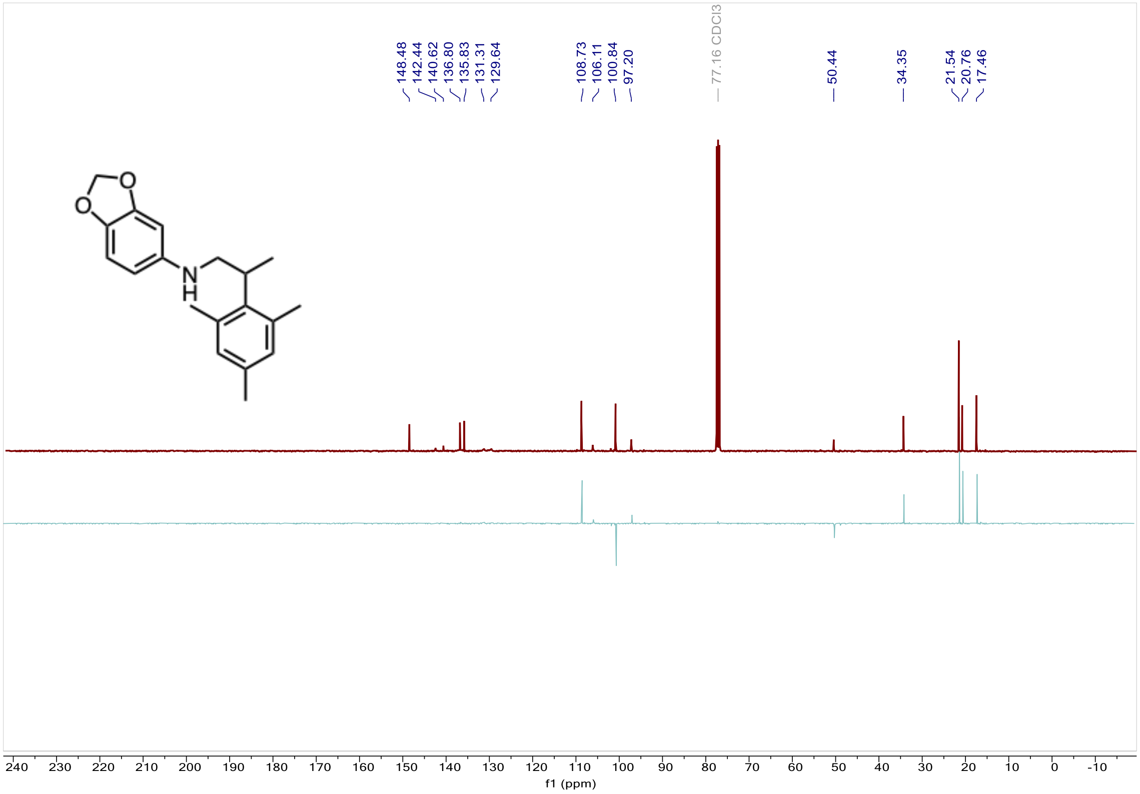

Butyl 4-((2-mesitylpropyl)amino)benzoate (2ai)

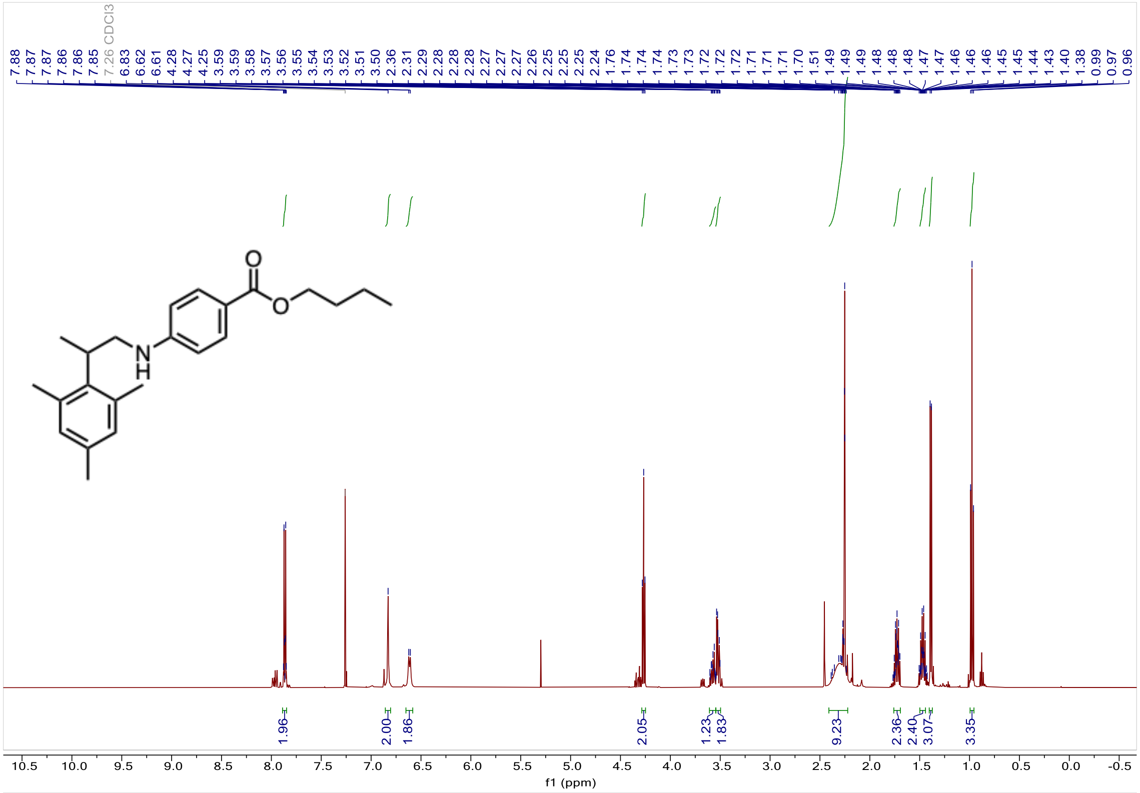

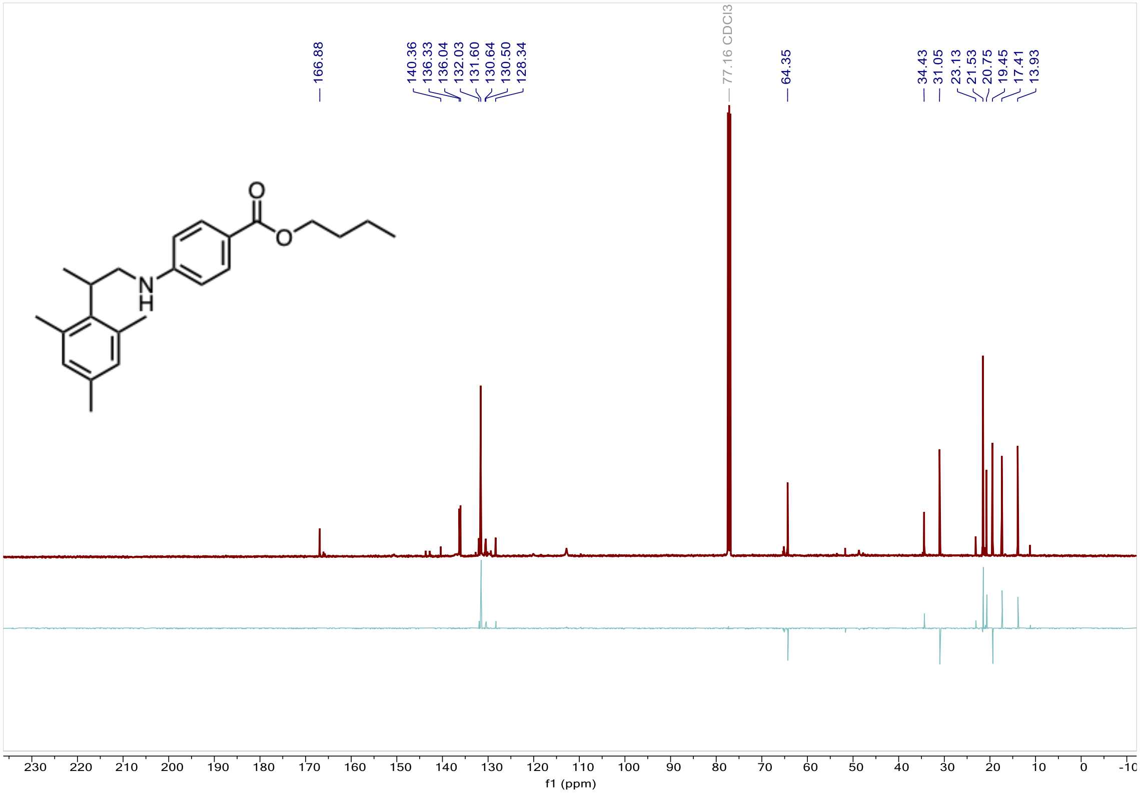

(1*R*,2*S*,5*R*)-2-isopropyl-5-methylcyclohexyl 4-((2-mesitylpropyl)amino)benzoate (2aj)

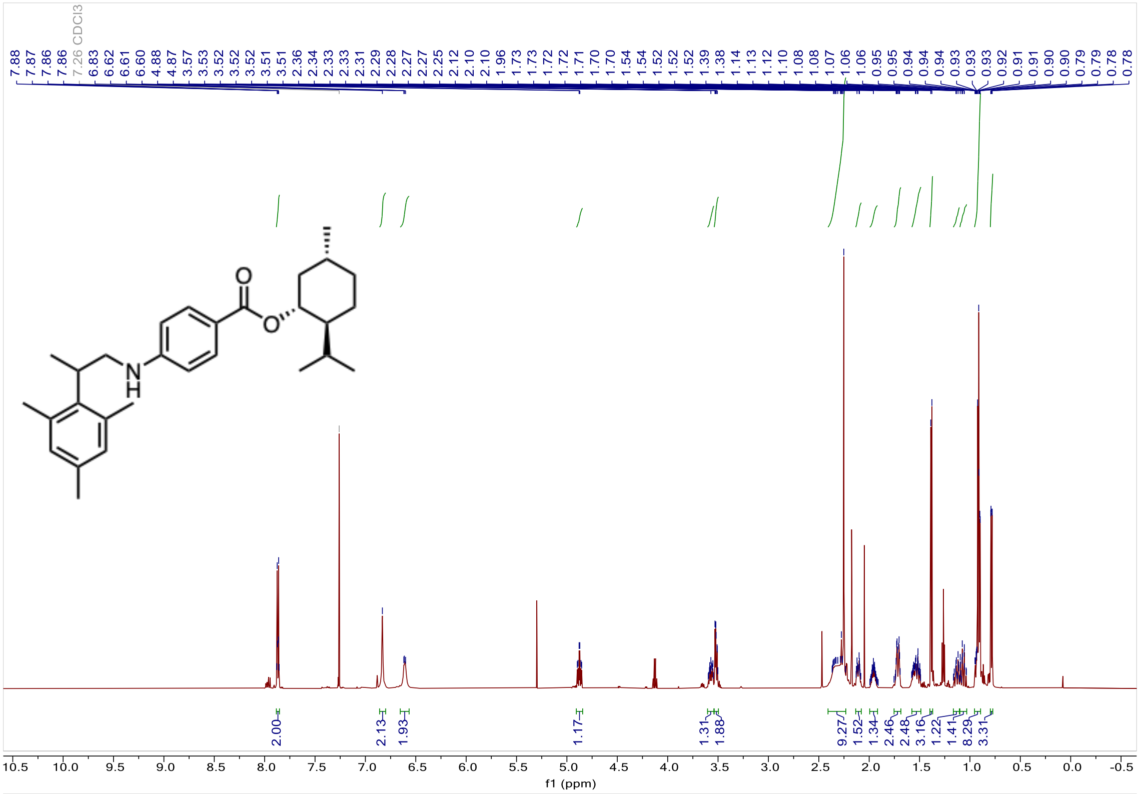

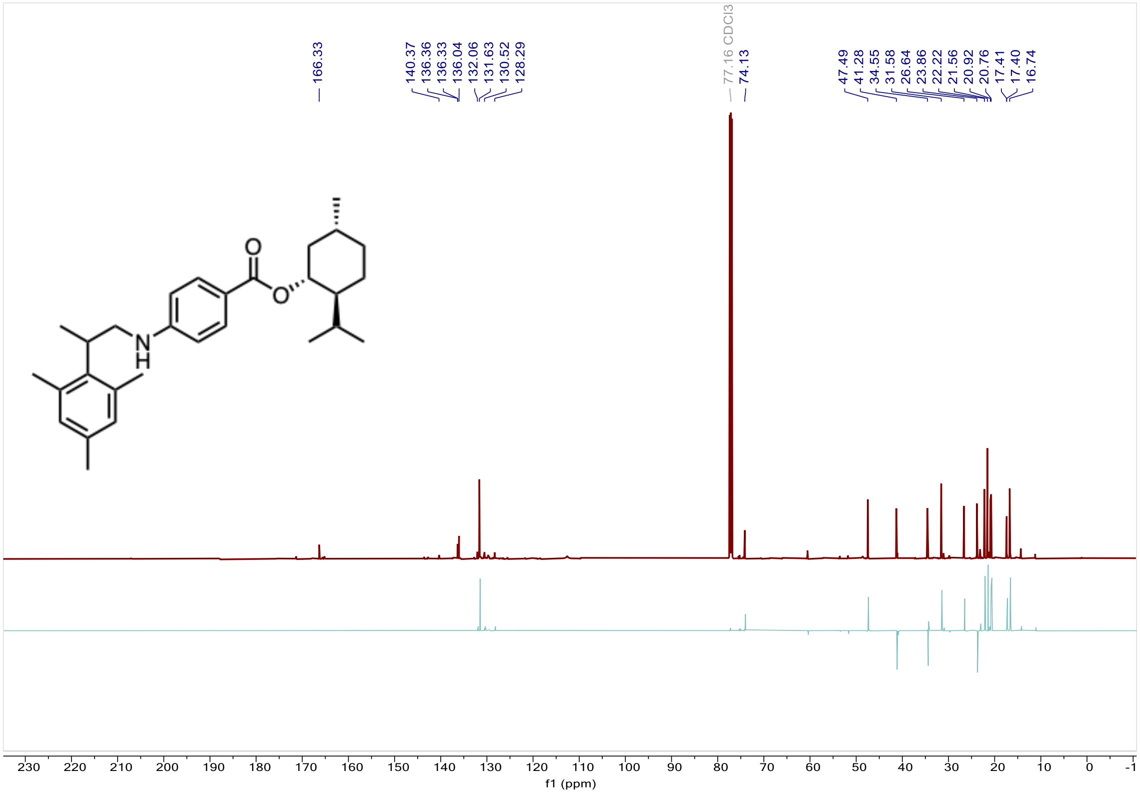

(*R*)-2,8-dimethyl-2-((4*R*,8*R*)-4,8,12-trimethyltridecyl)chroman-6-yl4-((2-mesityl propyl)amino)benzoate (2ak)

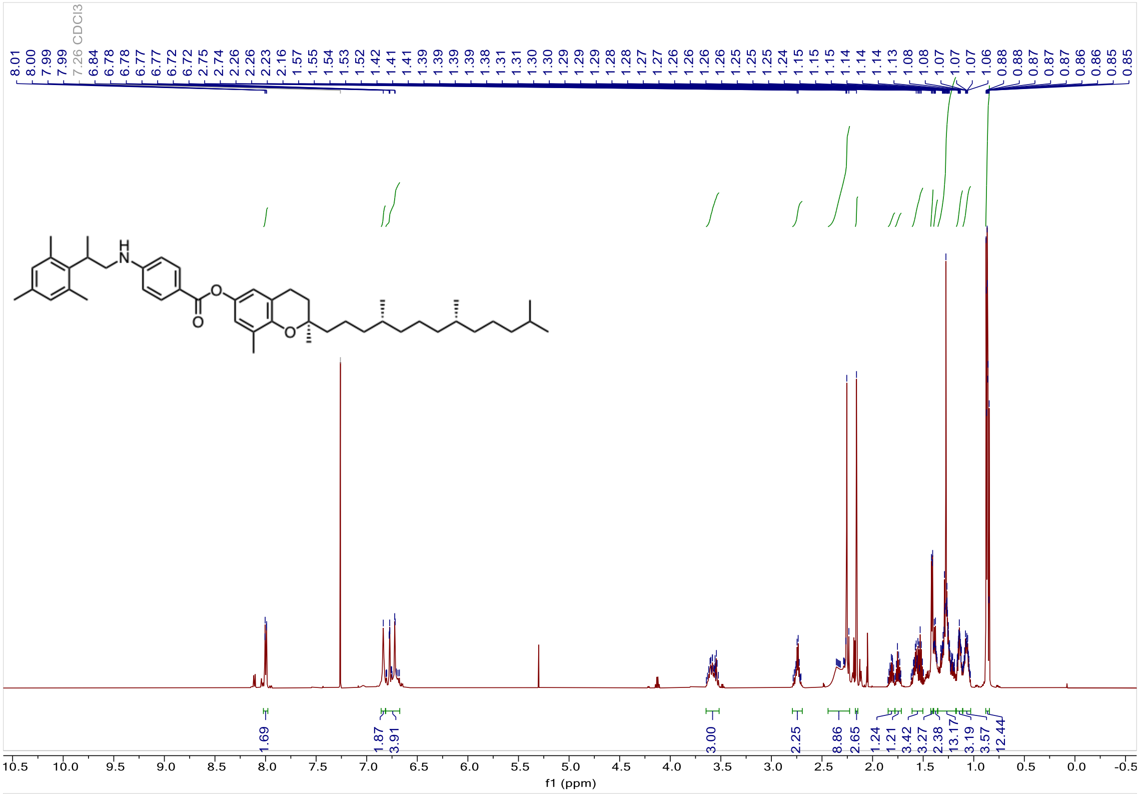

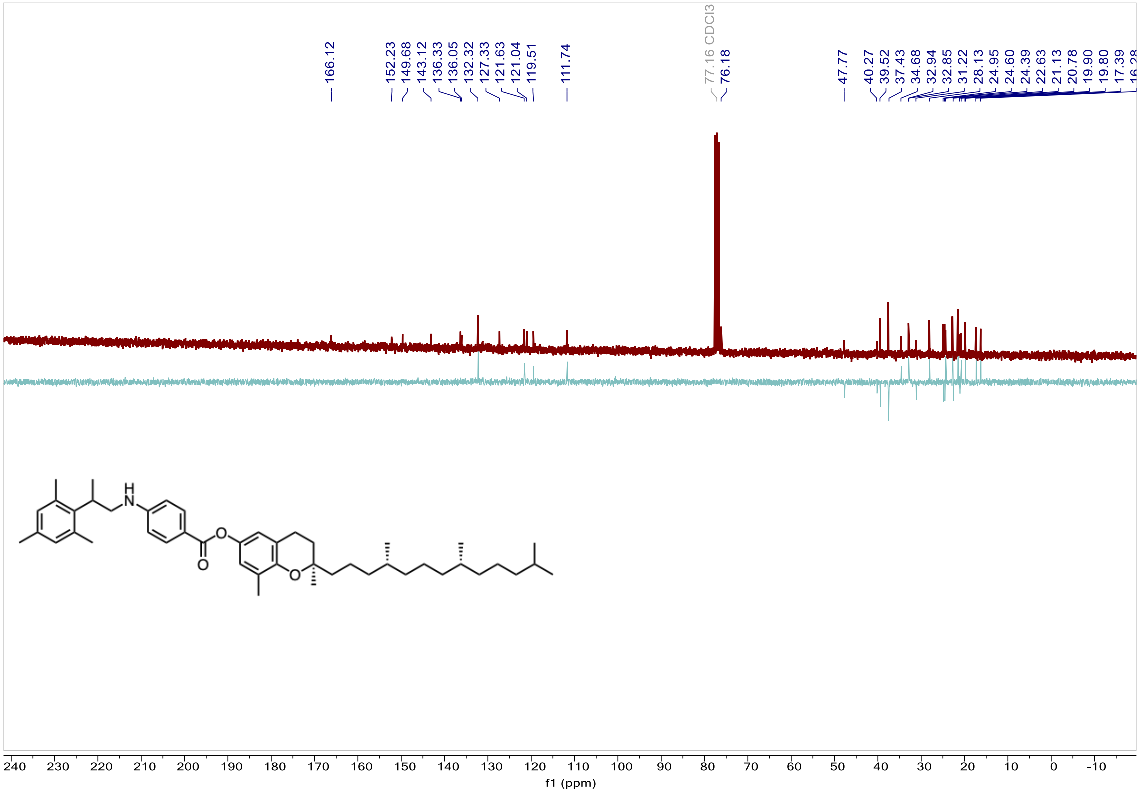

_N_-(2-mesitylbutyl)aniline (2al)

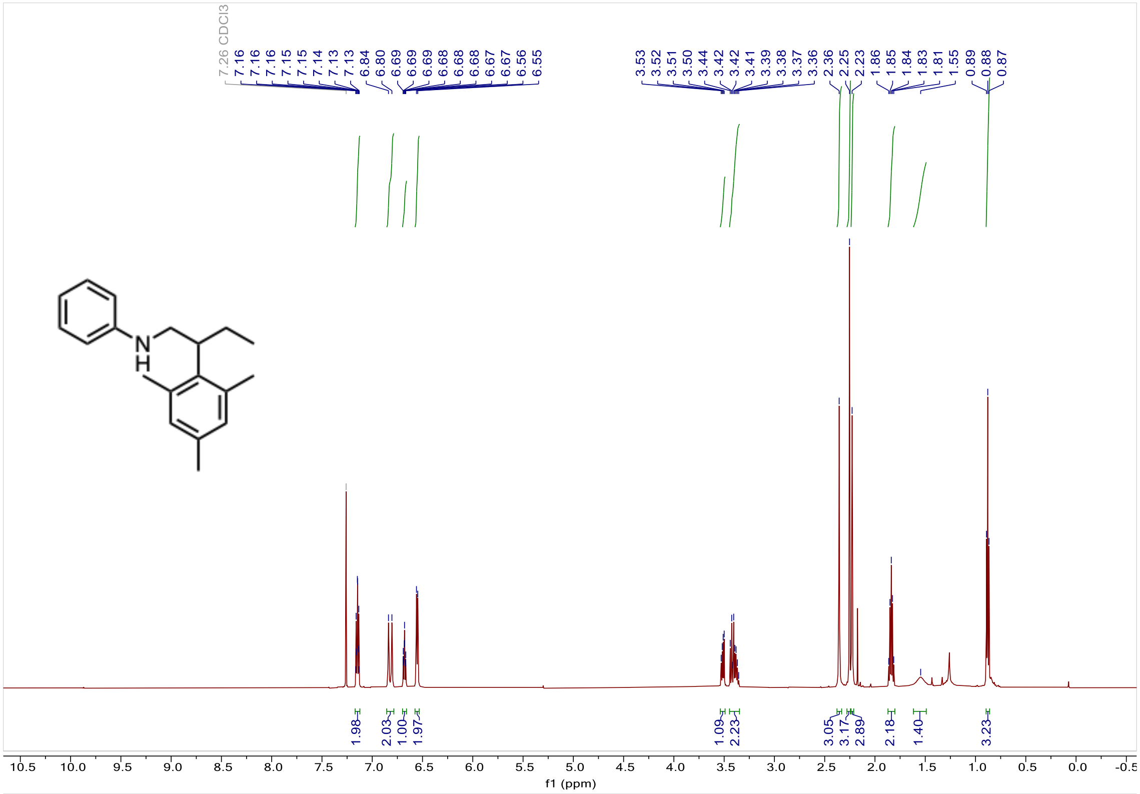

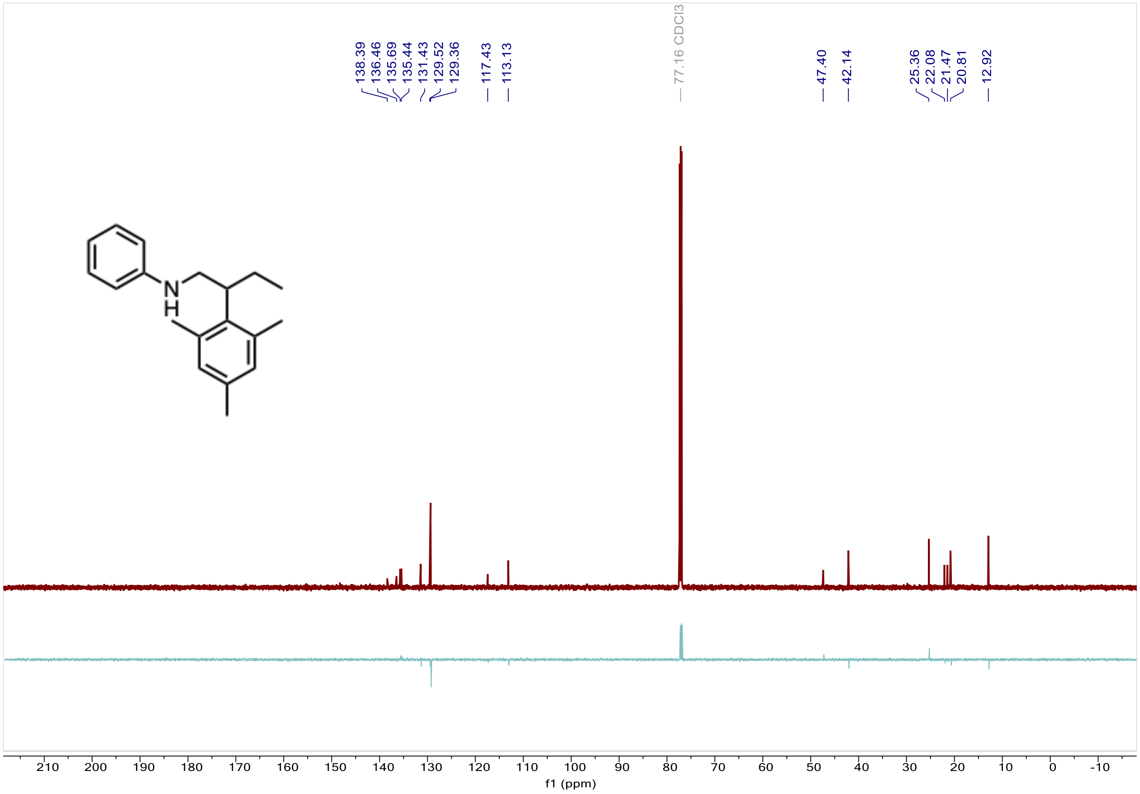

N-(2-mesitylbutyl)aniline (2am)

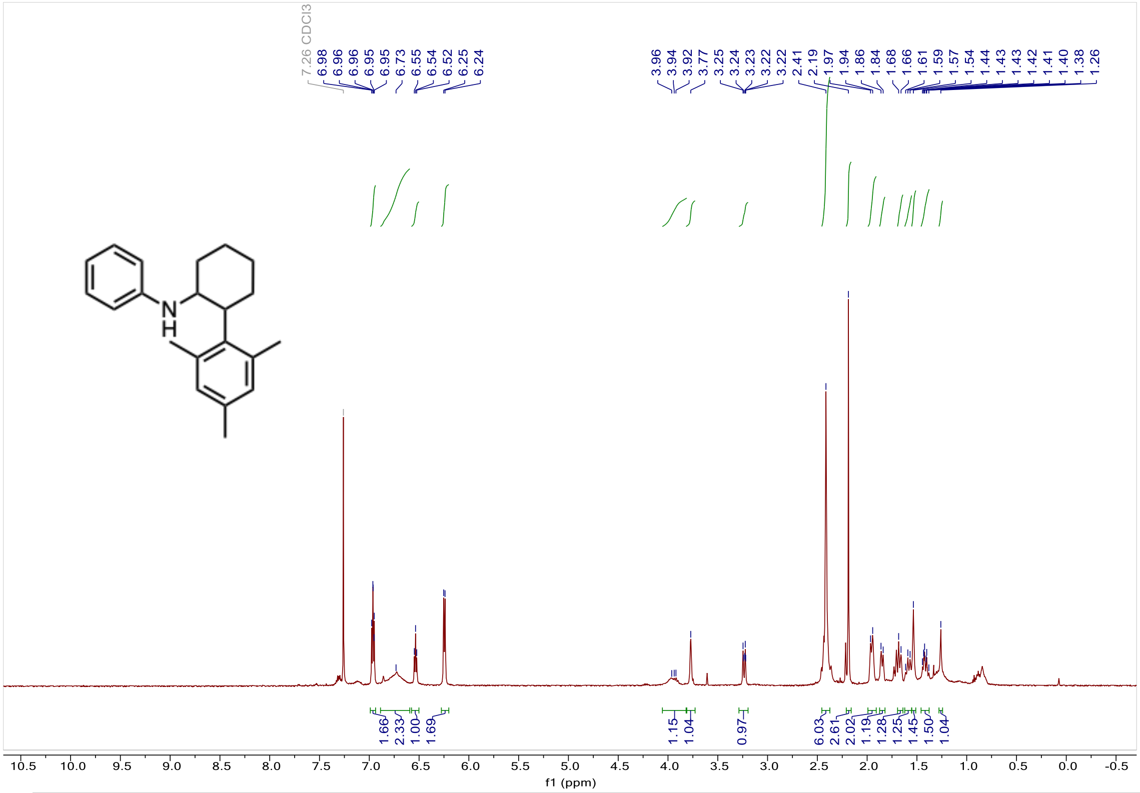

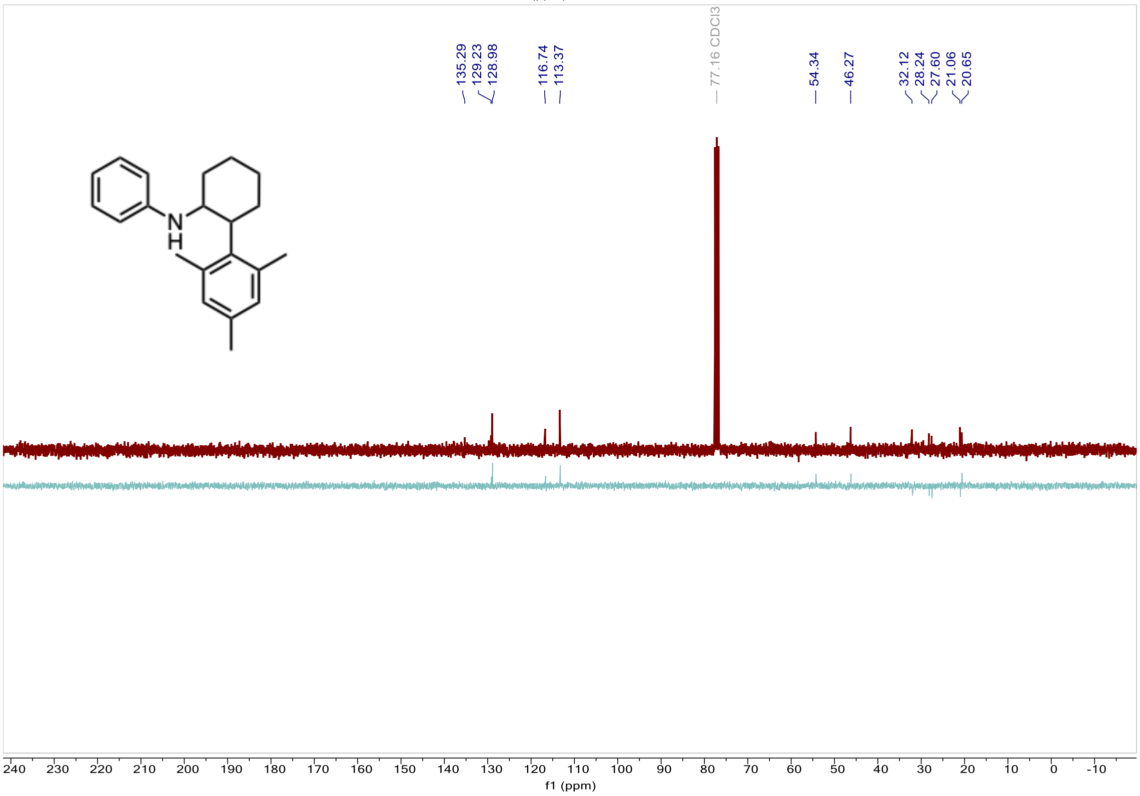

2-mesityl-*N*-phenylpropanamide (2an)

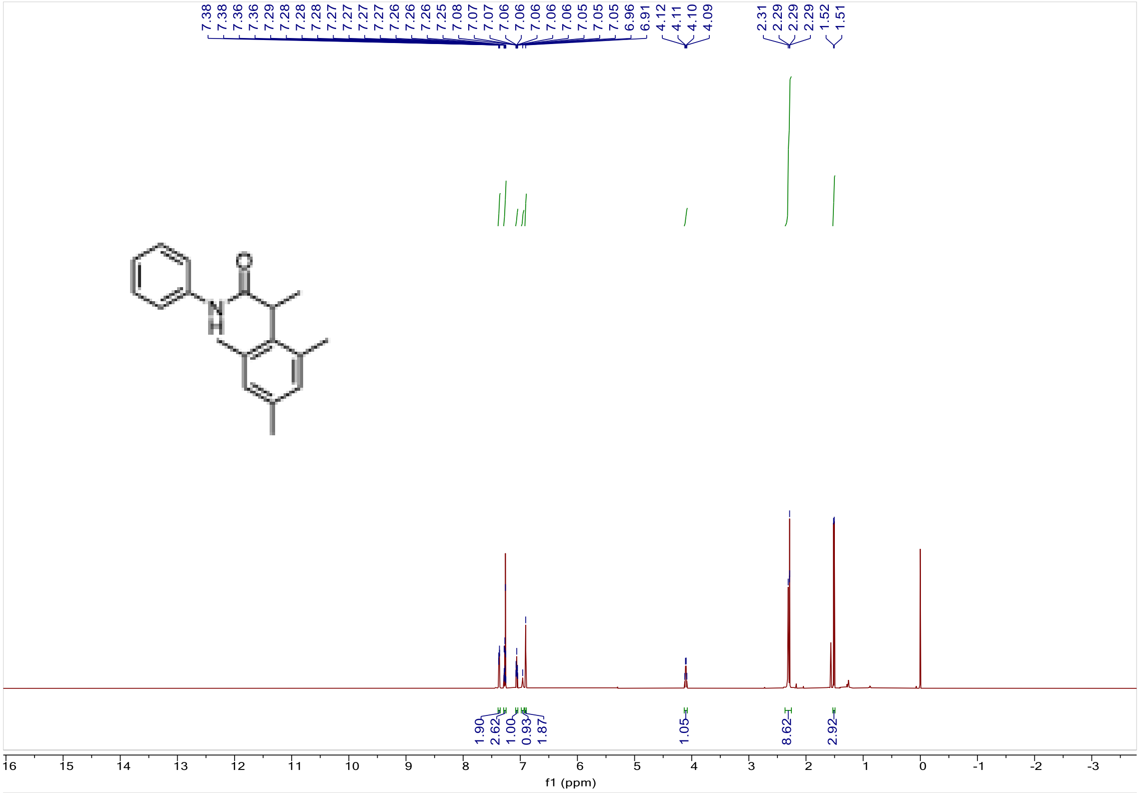

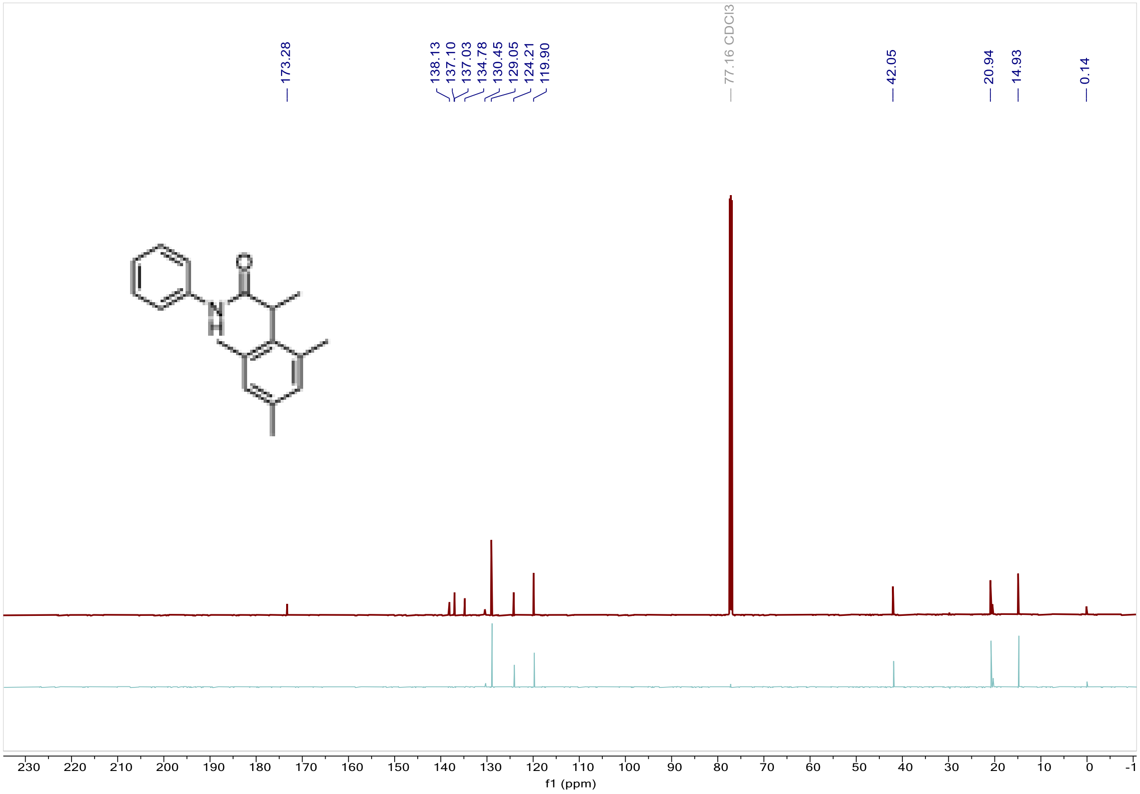

3-cyclohexyl-2-mesityl-*N*-phenylpropanamide (2an‘)

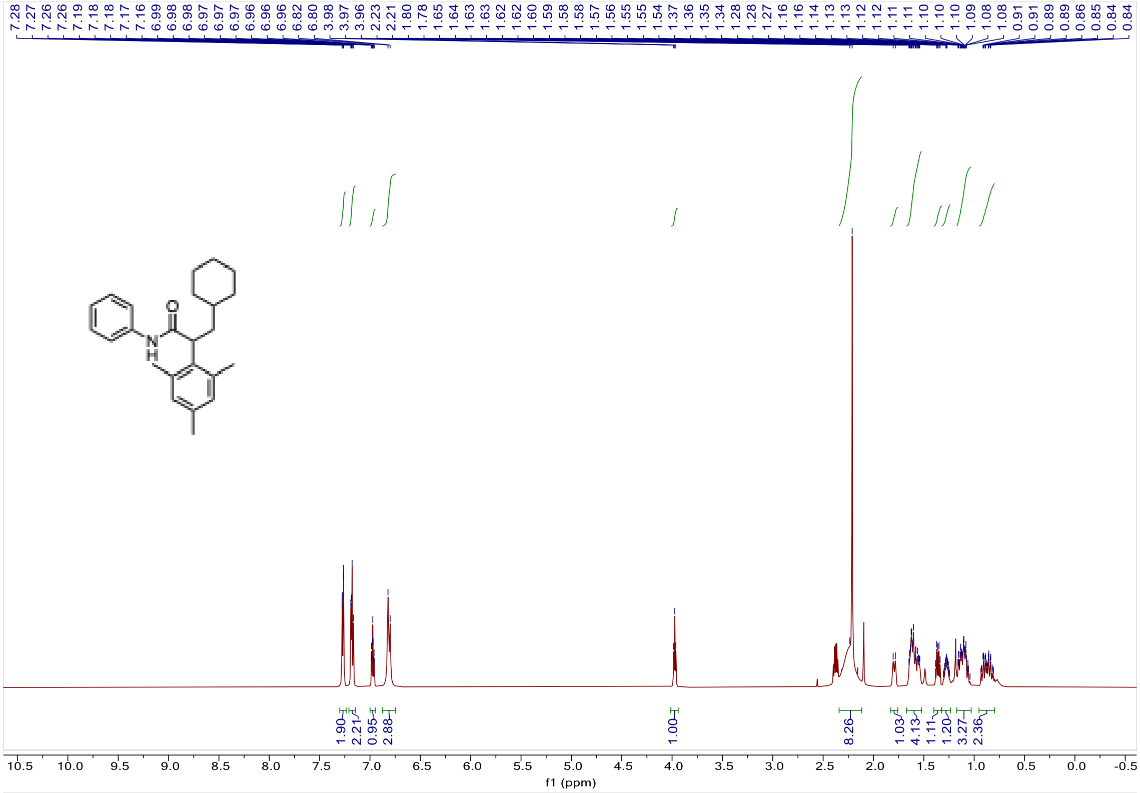

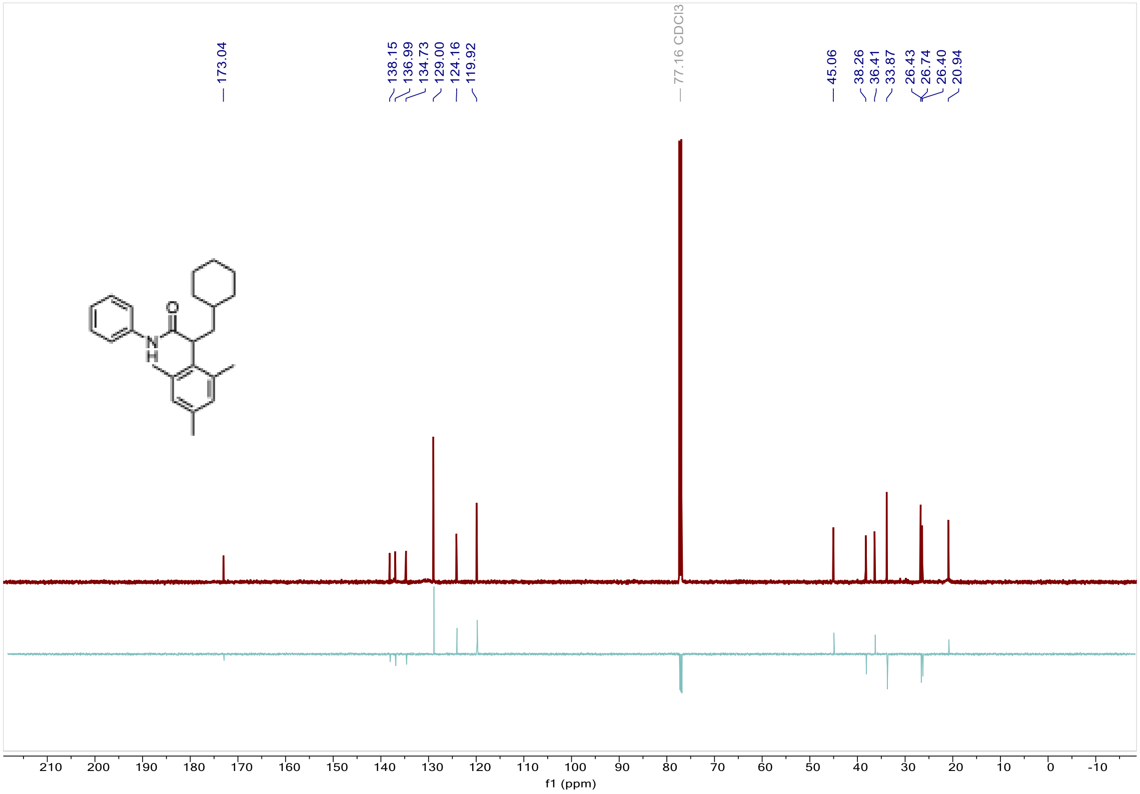

4-(2-mesityl-1-(phenylamino)propyl)benzonitrile (3)

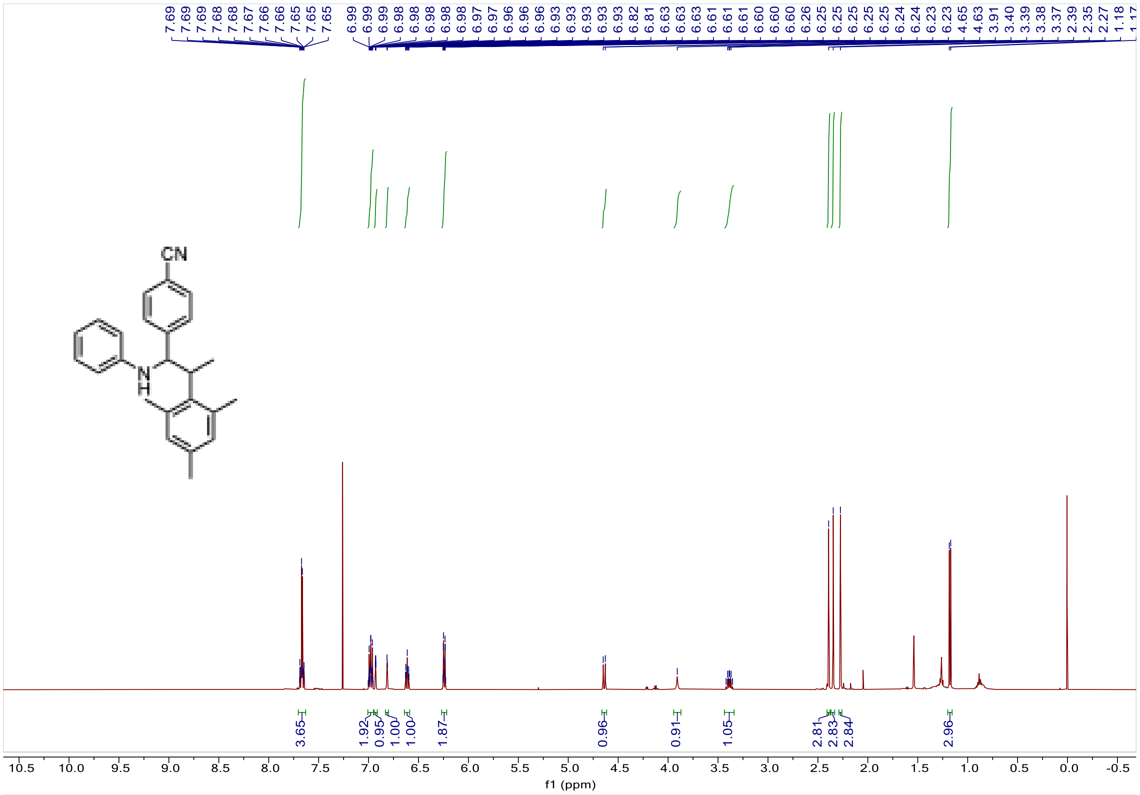

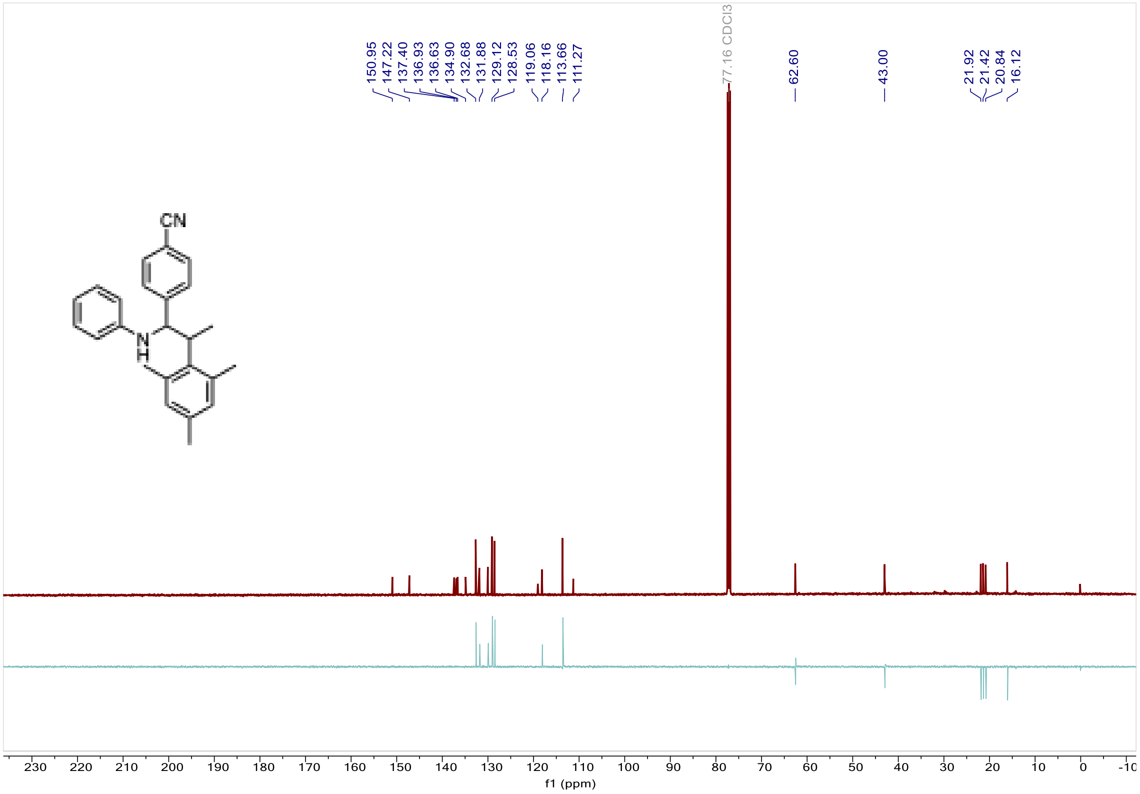

Supplement: Supplementary file 1 — Supporting Information [file ANIE-64-e202418869-s001.pdf]
